# Larvicidal Activity, GC–MS Profiling and Multitarget Molecular Docking of Essential Oils from *Cymbopogon citratus*, *Tanacetum cinerariifolium* and *Salvia rosmarinus* Against *Anopheles arabiensis*

**DOI:** 10.3390/molecules31183346

**Published:** 2026-09-21

**Authors:** Eric Kibagendi Osoro, Bernard Mwiti Kithetu, Alex Muthengi, Njogu M. Kimani, Bulelwa Audrey Ngcangatha, Fidelis Ngugi, Mojeed Adedoyin Agoro, Nicholas Rono

**Affiliations:** 1 Basic Sciences Department, Tharaka University, Marimanti P.O Box 193-60215, Kenya; kithetumwiti@gmail.com (B.M.K.); alex.mugwiria@tharaka.ac.ke (A.M.); fidelis.ngugi@tharaka.ac.ke (F.N.); 2 Department of Physical Sciences, University of Embu, Embu P.O. Box 6-60100, Kenya; mark.njogu@gmail.com; 3 Department of Agriculture Eastern Cape Province, Dohne Agricultural Development Institute, Private Bag X15, Stutterheim 4930, South Africa; bulelwa.ngcangatha@ecagriculture.gov.za; 4 Institute of Technology, University of Fort Hare, Private Bag X1314, Alice 5700, South Africa; magoro@ufh.ac.za

**Keywords:** juvenile hormone-binding protein, sterol carrier protein-2, *Anopheles*, mosquitoes, larvicidal, essential oil, multitarget inhibition

## Abstract

Malaria remains a major public health challenge, particularly in sub-Saharan Africa, necessitating the development of environmentally sustainable alternatives to conventional insecticides. This study evaluated the larvicidal efficacy of essential oils extracted from *Cymbopogon citratus*, *Tanacetum cinerariifolium* and *Salvia rosmarinus* against *Anopheles arabiensis* larvae. The phytochemical composition of the essential oils was characterized using gas chromatography–mass spectrometry (GC–MS), while their potential molecular mechanisms of action were investigated through molecular docking against selected mosquito larval target proteins. All essential oils exhibited concentration-dependent larvicidal activity, with *T. cinerariifolium* (LC_50_ = 511.0 mg/L; 95% CI: 473.3–547.1 mg/L) and *C. citratus* (LC_50_ = 602.7 mg/L; 95% CI: 559.3–648.5 mg/L) demonstrating the greatest potency. GC–MS analysis identified sesquiterpene hydrocarbons as the predominant constituents of *T. cinerariifolium*, whereas oxygenated monoterpenes dominated the oils of *C. citratus* and *S. rosmarinus*. Hierarchical molecular docking followed by MM-GBSA analysis against mosquito juvenile hormone-binding protein (JHBP) and sterol carrier protein-2 (SCP-2) revealed that 87.13% and 77.23% of the identified phytochemicals, respectively, exhibited favorable binding affinities toward these targets, indicating that a substantial proportion of the essential oil constituents have the potential to interact with proteins critical for mosquito development and survival. Farnesol emerged as the most promising multitarget phytochemical, displaying consistently favorable docking scores and binding free energies against both proteins, while 1,10-di-epi-cubenol and (5Z,9Z)-farnesyl acetone exhibited the strongest target-specific interactions with JHBP and SCP-2, respectively. In addition, the lead compounds satisfied Tice’s insecticide-likeness criteria, supporting their suitability as insecticidal leads. Collectively, these findings demonstrate that the larvicidal activity of the investigated essential oils is likely mediated through the synergistic action of multiple phytochemicals acting on complementary molecular targets, highlighting *T. cinerariifolium* and *C. citratus* as promising sources of eco-friendly botanical larvicides for mosquito vector control.

## 1. Introduction

Malaria remains one of the most important vector-borne diseases worldwide which continues to pose a major public health challenge, particularly in sub-Saharan Africa, where it accounts for a substantial burden of morbidity and mortality [1]. The disease is caused by *Plasmodium* parasites transmitted through the bites of infected female *Anopheles* mosquitoes, with the African region accounting for approximately 94% of global malaria cases due to the prevalence of highly efficient vector species and the dominance of *Plasmodium falciparum* [2]. Given the critical role of mosquito vectors in malaria transmission, effective vector control remains one of the most important strategies for reducing disease incidence and transmission. Among the available vector management strategies, larval control has gained considerable attention because it targets mosquitoes before they emerge as disease-transmitting adults, thereby reducing vector populations and interrupting disease transmission. Consequently, the development of effective and eco-friendly larvicidal agents has become increasingly important, particularly in the face of growing insecticide resistance and the environmental concerns associated with synthetic insecticides.

The growing concerns surrounding the extensive use of synthetic insecticides, including their toxicity to non-target organisms, environmental persistence, bioaccumulation and the emergence of resistant insect populations, have intensified the search for safer and more sustainable alternatives [3,4]. Natural products represent a rich source of bioactive compounds and have been successfully utilized in pest management owing to their biodegradability, reduced ecological footprint, target specificity and lower propensity to induce resistance [5]. Among the natural products, essential oils have attracted considerable attention as potential mosquito larvicides due to their rich composition of bioactive secondary metabolites, including terpenes, terpenoids and phenylpropanoids. Essential oils are generally biodegradable, environmentally friendly and possess diverse modes of action that may reduce the likelihood of resistance development [4]. Moreover, numerous plant essential oils have demonstrated significant larvicidal activity against mosquito vectors, highlighting their potential as effective and sustainable tools for malaria vector control.

Among the plant species considered in this study are *Cymbopogon citratus* (Poaceae), *Tanacetum cinerariifolium* (Asteraceae) and *Salvia rosmarinus* (Lamiaceae), all of which are recognized for producing essential oils with insecticidal properties. *C. citratus* has demonstrated larvicidal activity against *Anopheles arabiensis* in extract form [6], while *T. cinerariifolium* is renowned as the natural source of pyrethrins, potent insecticidal compounds widely used in mosquito control [7]. Similarly, the essential oils of *S. rosmarinus* have been reported to exhibit insecticidal, repellent, and larvicidal activities against several mosquito species [8]. Despite these promising bioactivities, information on the larvicidal efficacy of the essential oils of these plants against *An. arabiensis* remains limited. This knowledge gap is particularly important given the increasing reports of insecticide resistance in *An. arabiensis* populations, which threaten the effectiveness of conventional vector control interventions [9]. Therefore, evaluating the larvicidal activity of these plant-derived essential oils against *An. arabiensis*, alongside *Azadirachta indica* oil, a well-established botanical insecticide used as a positive control [10], may provide valuable comparative evidence and insights into the potential of these essential oils as sustainable alternatives for malaria vector management.

Recent advances in computational tools have accelerated the discovery of novel insecticidal agents by enabling rapid screening of bioactive compounds against key molecular targets. Molecular docking is widely used to predict the binding affinities and interactions of candidate compounds with proteins involved in insect growth, development, and survival [11]. In this study, docking was employed to evaluate the interactions of essential oil constituents with two mosquito larval targets: juvenile hormone-binding protein (JHBP) and sterol carrier protein-2 (SCP-2). These proteins regulate critical processes including growth and metamorphosis and lipid transport, respectively [12,13]. Accordingly, this study evaluated the larvicidal activity of essential oils from *C. citratus*, *T. cinerariifolium* and *S. rosmarinus* against *An. arabiensis* and integrated GC–MS characterization with molecular docking analyses to identify putative bioactive constituents and elucidate their interactions with key mosquito target proteins.

## 2. Results and Discussion

### 2.1. Larvicidal Activity of the Essential Oils Against Anopheles arabiensis Larvae

**Figure 1:**
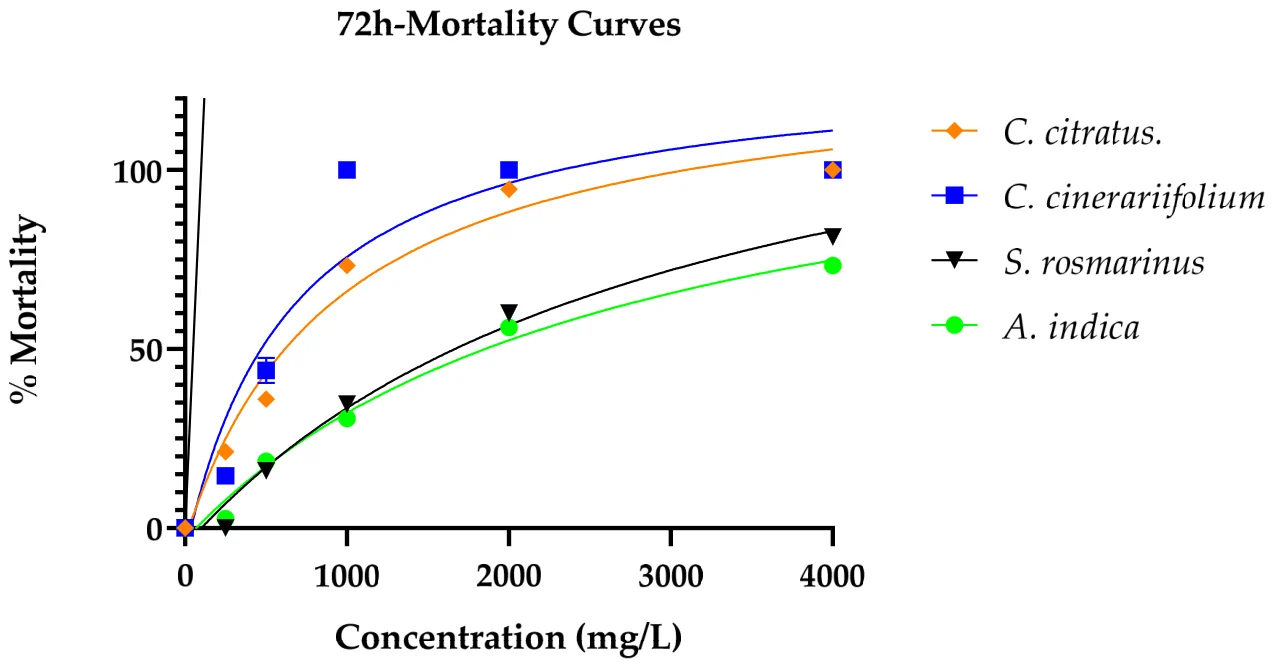
Nonlinear concentration–response curves of the tested essential oils and *Azadirachta indica* against *An. arabiensis* larvae after 72 h of exposure.

The larvicidal activities of essential oils obtained from *C. citratus*, *T. cinerariifolium* and *S. rosmarinus* were assessed against *An. arabiensis* larvae under controlled laboratory conditions. The results, summarized in Table 1 and Figure 1, were compared with those of *A. indica*, a well-established botanical insecticide used as a positive control [10]. The bioassay was conducted at multiple concentrations to investigate concentration-dependent larvicidal effects, and mortality rates were assessed over a 72-h exposure period. Essential oils from all the plant species exhibited concentration-dependent larvicidal activity against *An. arabiensis* larvae. Mortality increased progressively with increasing concentration and exposure duration, with *T. cinerariifolium* and *C. citratus* showing the greatest efficacy, achieving complete larval mortality at 2000–4000 mg/L and 4000 mg/L, respectively, after 72 h of exposure. Based on the LC_50_ values, *T. cinerariifolium* (511.0 mg/L; 95% confidence interval (CI): 473.3–547.1 mg/L) and *C. citratus* (602.7 mg/L; 95% CI: 559.3–648.5 mg/L) exhibited the highest larvicidal activity against *An. arabiensis* larvae, with *T. cinerariifolium* showing greater potency than *C. citratus*.

As shown in Table 2 and Figure 1, both *T. cinerariifolium* and *C. citratus* exhibited significantly greater larvicidal activity against *An. arabiensis* larvae than the positive control (*A. indica*) at most concentrations tested (*p* < 0.05), corroborating their lower LC_50_ values of 511.0 mg/L and 602.7 mg/L, respectively. In contrast, the activity of *S. rosmarinus* was generally comparable to that of *A. indica*, with no significant differences observed at most concentrations (*p* > 0.05). Direct comparison between *T. cinerariifolium* and *C. citratus* revealed significantly higher activity for *T. cinerariifolium* at 250, 1000, and 2000 mg/L, whereas no significant difference was detected at 500 mg/L. In addition, both essential oils achieved complete larval mortality at 4000 mg/L. These findings indicate that the essential oils of *T. cinerariifolium* and *C. citratus* exhibited the greatest larvicidal efficacy among the tested treatments and showed higher potency than the positive control, *A. indica*. The lower LC_50_ value of *T. cinerariifolium* indicates greater potency than *C. citratus*, suggesting that the former may be particularly promising for further investigation as a botanical larvicide. The strong concentration-dependent activity observed for both oils, together with their relatively low LC_50_ values, highlights their potential for mosquito larval control.

To further characterize the concentration–mortality relationships, nonlinear concentration–response regression was performed to estimate the 72-h LC_50_ values and assess the goodness of fit of the fitted models. The nonlinear concentration–response analysis showed a strong fit for all treatments, with R^2^ values ranging from 0.9845 to 0.9905 (Table 1). These high coefficients of determination indicate that the fitted concentration–mortality models explained approximately 98.45–99.05% of the variation in larval mortality, supporting the reliability of the estimated concentration–response relationships. The highest goodness of fit was observed for *S. rosmarinus* (R^2^ = 0.9905), followed by *C. citratus* (R^2^ = 0.9897), *A. indica* (R^2^ = 0.9887) and *T. cinerariifolium* (R^2^ = 0.9845). Although the R^2^ value for *T. cinerariifolium* was comparatively lower, it still indicated an excellent fit, demonstrating that concentration was strongly associated with larval mortality. The Hill slope values ranged from 1.33 to 3.80 indicating differences in the steepness of the concentration–response relationships among the treatments. *T. cinerariifolium* exhibited the highest Hill slope (3.80), indicating a particularly steep response around its LC_50_. Thus, relatively small changes in concentration around the LC_50_ were associated with pronounced changes in mortality. This steep response is consistent with the rapid increase in mortality observed between 500 and 1000 mg/L, where mortality increased from 44% to 100% at 72 h. *C. citratus* also showed a relatively steep concentration–response relationship (Hill slope = 1.96), although less pronounced than that of *T. cinerariifolium*. In contrast, *S. rosmarinus* (1.59) and *A. indica* (1.33) exhibited lower Hill slopes, indicating more gradual changes in mortality with increasing concentration. Importantly, the Hill slope reflects the steepness of the concentration–response curve rather than overall larvicidal potency; potency is more appropriately interpreted from the LC_50_ values. Overall, the combination of high R^2^ values and biologically interpretable Hill slopes supports the adequacy of the nonlinear regression models for describing the concentration-dependent larvicidal effects.

The larvicidal activity observed in the present study is consistent with previous reports demonstrating the larvicidal potential of these essential oils against diverse mosquito species. *C. citratus* essential oil has shown pronounced larvicidal activity against several *Anopheles* species, including *An. sinensis*, *An. gambiae* and *An. funestus*, as well as *Aedes* species [14,15,16,17,18]. Similarly, *S. rosmarinus* essential oil has demonstrated activity against *Ae. aegypti* and *Ae. albopictus* [19]. *T. cinerariifolium* has a well-established insecticidal history against mosquitoes through its pyrethrin constituents, but relatively little information is available specifically on its chemically characterized essential oil against *Anopheles* larvae [20,21,22]. In their mechanisms of action, constituents of *C. citratus* essential oil have been associated with inhibition of acetylcholinesterase activity while *S. rosmarinus* essential oil with acetylcholinesterase and α-amylase activities [14,19]. These findings, together with the activity observed against *An. arabiensis* in the present study, suggest that the tested essential oils possess broad-spectrum mosquitocidal potential. Differences in activity among studies may reflect variations in mosquito species and larval susceptibility, developmental stage, plant material, essential-oil composition, exposure duration and assay conditions.

### 2.2. Chemical Composition of the Essential Oils

GC–MS analysis of the essential oil extracted from *C. citratus* resulted in the identification of 50 constituents. The distribution of these compounds according to their chemical classes is summarized in Table 3. The essential oil was predominantly composed of oxygenated monoterpenes (68.56%), with geranial (28.00%), neral (19.01%), 1,8-Cineole (eucalyptol) (11.76%) and verbenone (2.60%) identified as the major constituents. The predominance of the citral isomers, geranial and neral, which together accounted for 47.01% of the total oil composition, indicates a citral-rich chemotype characteristic of *C. citratus* essential oil [23]. Citral, a mixture of the geometric isomers geranial (trans-citral) and neral (cis-citral), is widely recognized as the principal bioactive constituent of lemongrass oil and is largely responsible for its characteristic lemon aroma and diverse biological activities. The high abundance of these compounds in the present study is consistent with previous reports identifying citral as the dominant constituent of *C. citratus* essential oil [24]. Monoterpene hydrocarbons represented the second largest chemical class (18.81%), predominantly comprising α-pinene, limonene, camphene, and myrcene. Oxygenated sesquiterpenes occurred only in trace amounts (0.90%), and no phenylpropanoid constituents were detected. The proposed constituents were assigned using an integrated assessment of GC–MS spectral matches, similarity index (SI) scores, and retention indices (RI). Most experimental RIs closely matched the reported literature values, including 1,8-cineole (1032 vs. 1032), α-pinene (933 vs. 933), limonene (1029 vs. 1030), and caryophyllene oxide (1581 vs. 1587), providing additional confidence in the compound identifications [25,26].

GC–MS analysis of the essential oil from *T. cinerariifolium* resulted in the identification of 66 constituents and their classification into various chemical classes presented in Table 4. The oil was predominantly composed of sesquiterpene hydrocarbons (79.0%), with γ-muurolene (30.98%), β-farnesene (27.73%), α-farnesene (4.42%), bicyclogermacrene (3.52%), and δ-cadinene (2.78%) identified as the major constituents. This chemical profile is consistent with previously reported constituents of pyrethrum (*T. cinerariifolium*) essential oil, which is typically dominated by sesquiterpene hydrocarbons [27,28]. Oxygenated sesquiterpenes constituted the second most abundant class (approximately 12.7%), represented mainly by nerolidol (3.40%), sesquilavandulol (2.09%), cadin-4-en-10-ol (1.03%), and T-muurolol (0.98%). Monoterpenes were present only in trace amounts, with oxygenated monoterpenes and monoterpene hydrocarbons accounting for 0.76% and 0.07% of the total oil composition, respectively. Experimental retention indices were interpreted alongside GC–MS assignments and spectral similarity scores to strengthen compound identification. The experimental RIs generally showed close agreement with literature values, including β-farnesene (1452 vs. 1452), γ-muurolene (1481 vs. 1478), α-farnesene (1502 vs. 1504) and isogermacrene D (1444 vs. 1447) among others, supporting the proposed identifications based on the combined GC–MS, SI, and RI evidence [25,26].

GC–MS analysis of the essential oil extracted from *S. rosmarinus* resulted in the identification of 57 constituents. The GC–MS data presented in Table 5, revealed that oxygenated monoterpenes constituted the predominant fraction of the *S. rosmarinus* essential oil, accounting for 56.5% of the total composition, followed by monoterpene hydrocarbons (33.0%). Sesquiterpenes (0.82%) and phenylpropanoids (0.45%) were present in relatively low proportions, while other compounds and unidentified constituents comprised 9.2% of the oil. This distribution highlights the predominance of monoterpenoid compounds, a characteristic feature of rosemary essential oils, and is indicative of a verbenone-rich chemotype [29]. The major constituents contributing to these chemical classes were α-pinene (21.96%), eucalyptol (1,8-cineole) (20.04%), verbenone (13.42%), geraniol (5.60%), borneol (4.94%) and camphor (3.14%). The dominant constituents and the overall predominance of monoterpenes are consistent with the characteristic chemical profile of *S. rosmarinus* essential oil. Although the oil exhibited a comparatively high verbenone content and a relatively low levels of camphor, such variations fall within the range of natural chemical diversity reported among rosemary populations [30]. Differences in the relative abundance of these constituents have been attributed to factors such as genotype, geographical origin, environmental conditions, developmental stage, and harvesting season, highlighting the considerable chemotypic variability that exists within the species [31]. The proposed constituents were supported by the combined GC–MS, SI, and RI evidence, with close experimental and literature RI values agreement for major compounds such as 1,8-cineole (1031 vs. 1032), α-pinene (933 vs. 933), and verbenone (1206 vs. 1208).

### 2.3. Molecular Docking Studies

To elucidate the molecular mechanisms underlying the observed larvicidal activity, molecular docking studies were employed to evaluate the binding interactions of the essential oil’s major constituents with two critical mosquito larval targets: juvenile hormone-binding protein (JHBP) and sterol carrier protein-2 (SCP-2). These proteins were selected due to their indispensable roles in larval survival and development. Specifically, JHBP is responsible for the hemolymph transport of juvenile hormone, a vital regulator of insect growth and metamorphosis [12,32] and SCP-2 mediates intracellular lipid trafficking, which is essential for energy storage and membrane synthesis in rapidly developing larvae [13]. Consequently, disruption of these molecular targets by bioactive essential oil constituents may interfere with endocrine regulation, lipid metabolism, and cellular signaling pathways, ultimately leading to impaired larval development and mortality. The molecular docking approach therefore provided valuable insights into the potential modes of action through which the essential oil exerts its larvicidal effects.

#### 2.3.1. Juvenile Hormone Binding Protein (PDB ID: 5V13)

**Figure 2:**
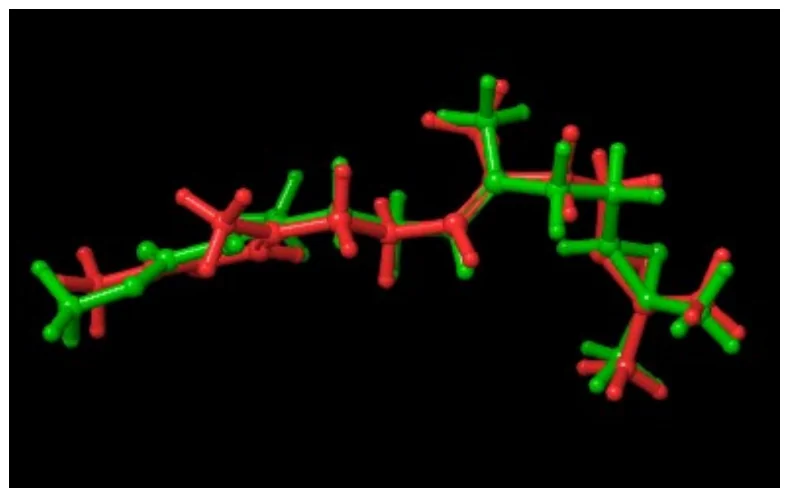
Superposition of the 5V13 co-crystallized ligand and redocked ligand within the binding site. The co-crystallized ligand is shown in green, while the redocked ligand is shown in red.

Following protein preparation, the receptor grid for PDB ID: 5V13 was generated by centering the grid box on the co-crystallized ligand, yielding grid coordinates of x = 252.28, y = 8.95, and z = 352.45. The docking protocol was validated by re-docking the co-crystallized ligand into the active site of the target protein. As shown in Figure 2, the superimposition of the co-crystallized and re-docked ligand poses yielded a root-mean-square deviation (RMSD) of 2.1 Å, indicating close agreement between the experimental and predicted binding poses [33]. Although the RMSD was marginally above the commonly applied 2.0 Å criterion, the observed structural agreement provided supportive evidence for the applicability of the docking protocol to subsequent screening of ligands against JHBP, while recognizing the limitation of the redocking validation. A total of 101 compounds identified by GC–MS were initially screened against the mosquito juvenile hormone-binding protein (JHBP; PDB ID: 5V13) using HTVS, resulting in the selection of 88 promising lead compounds. These leads were subsequently evaluated using the Glide Standard Precision (SP) docking protocol, followed by Extra Precision (XP) docking to improve the accuracy and selectivity of binding pose prediction. This hierarchical virtual screening workflow identified the 15 highest-ranked candidate compounds, whose docking scores and MM-GBSA binding free energy values are presented in Table 6. The top-ranked phytochemicals exhibited docking scores ranging from −8.261 to −9.482 kcal/mol, indicating favorable binding affinities toward JHBP. Although none of the compounds surpassed the co-crystallized ligand (docking score = −9.985 kcal/mol; ΔG_bind = −78.10 kcal/mol), several displayed comparable binding affinities, suggesting their potential to bind within the JHBP ligand-binding pocket and potentially interfere with juvenile hormone transport.

Among the screened phytochemicals, 1,10-di-epi-cubenol, a sesquiterpene alcohol identified in *C. citratus*, exhibited the most favorable docking score (−9.482 kcal/mol). The other the remaining top-ranked compounds, including farnesol (−8.840 kcal/mol), α-zingiberene, (E)-α-bisabolene and β-bisabolene, were identified in *T. cinerariifolium*. Despite ranking third based on docking score, farnesol exhibited the most favorable MM-GBSA binding free energy (ΔG_bind = −69.29 kcal/mol), approaching that of the co-crystallized ligand (−78.10 kcal/mol), suggesting the formation of a highly stable protein–ligand complex. Similarly, nerolidol (−55.02 kcal/mol), α-zingiberene (−52.87 kcal/mol), (E)-α-bisabolene (−44.17 kcal/mol) and β-bisabolene (−42.14 kcal/mol) also exhibited favorable binding free energies, indicating stable interactions with JHBP. In contrast, α-muurolol (ΔG_bind = 6.33 kcal/mol) and globulol (ΔG_bind = −7.89 kcal/mol) exhibited relatively weak binding free energies despite their favorable docking scores, highlighting the importance of MM-GBSA analysis in refining docking predictions by providing a more realistic estimate of ligand–protein binding stability. Collectively, these findings identify 1,10-di-epi-cubenol from *C. citratus* and farnesol, nerolidol, α-zingiberene and (E)-α-bisabolene from *T. cinerariifolium* as the most promising phytochemical candidates for targeting mosquito juvenile hormone-binding protein.

**Figure 3:**
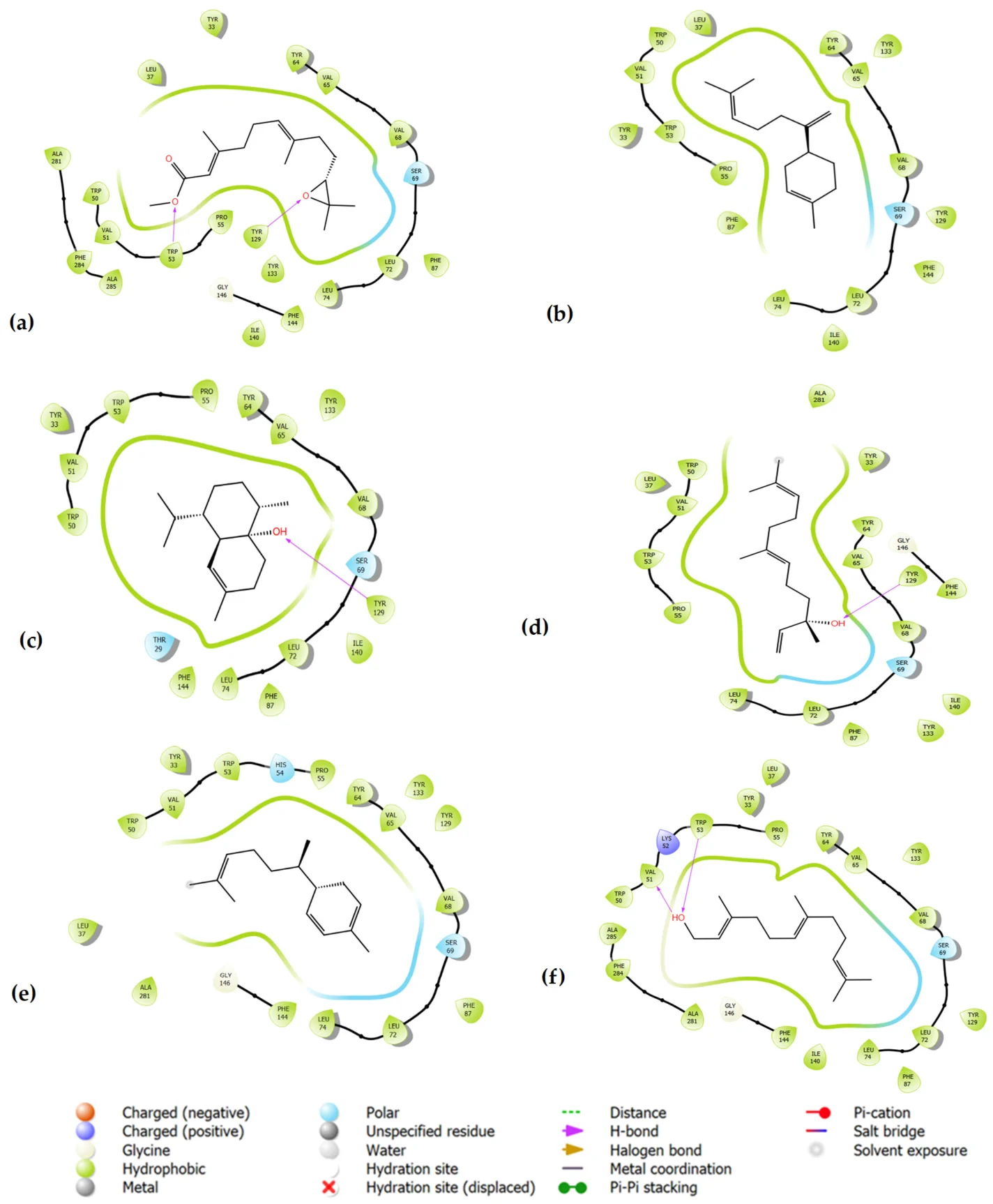
Two-dimensional (2D) visualization of protein–ligand interactions for top-ranked compounds and co-crystalized ligand within the 5V13 binding pocket: (**a**) co-crystalized ligand, (**b**) β-bisabolene, (**c**) 1,10-di-epi-cubenol, (**d**) nerolidol, (**e**) α-zingiberene and (**f**) farnesol.

Two-dimensional (2D) interaction analysis of the top-ranked phytochemicals and the co-crystallized ligand within the juvenile hormone-binding protein (JHBP) binding pocket revealed a highly conserved binding mode characterized predominantly by hydrophobic interactions (Figure 3), consistent with the hydrophobic ligand-binding cavity reported for insect JHBP, which facilitates the binding and transport of the lipophilic juvenile hormone [34]. The co-crystallized ligand was stabilized by extensive hydrophobic contacts with TRP50, VAL51, TRP53, PRO55, TYR64, VAL65, VAL68, LEU72, LEU74, PHE87 TYR129, TYR133, PHE144, and ILE140, together with hydrogen bonds involving TRP53 and TYR129. Similarly, the top-ranked phytochemicals occupied the same hydrophobic cavity and shared many of these key interacting residues, suggesting a common binding mechanism. β-Bisabolene and α-zingiberene, which are non-polar sesquiterpene hydrocarbons, interacted almost exclusively through hydrophobic contacts. In contrast, the hydroxyl-containing sesquiterpenes formed additional hydrogen-bonding interactions while maintaining extensive hydrophobic contacts with surrounding residues. Specifically, 1,10-di-epi-cubenol formed a hydrogen bond with TRP50, nerolidol with TYR129 and farnesol formed hydrogen bonds with TRP53 and VAL51. The combination of hydrogen bonding and hydrophobic contacts likely contributes to the enhanced binding stability observed for these oxygenated compounds, particularly farnesol, which exhibited the most favorable MM-GBSA binding free energy [35,36]. The recurrence of residues TRP50, TRP53, VAL51, VAL65, VAL68, LEU72, LEU74, TYR64, TYR129, TYR133, PHE87 and PHE144 across the interaction profiles highlights their critical role in ligand recognition and stabilization within the JHBP binding pocket. Collectively, these findings indicate that the identified phytochemicals mimic the binding mode of the native ligand and support their potential to competitively occupy the juvenile hormone-binding cavity, thereby potentially disrupting juvenile hormone transport and mosquito larval development.

#### 2.3.2. Sterol Carrier Protein-2 (PDB ID:1PZ4)

**Figure 4:**
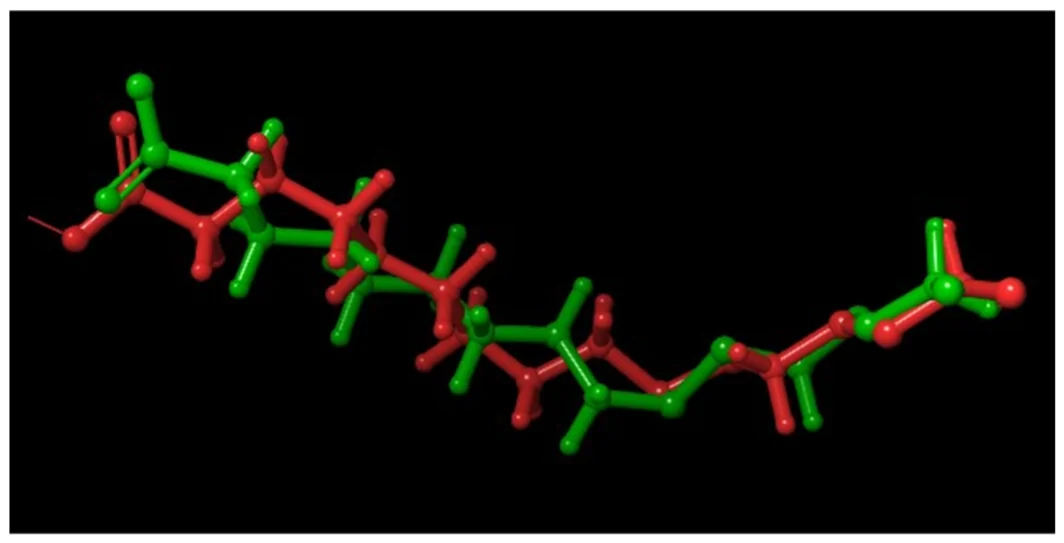
Superposition of the 1PZ4 co-crystallized ligand and redocked ligand within the binding site. The co-crystallized ligand is shown in green, while the redocked ligand is shown in red.

Following the target protein preparation, the generated grid box coordinates for PDB ID 1PZ4 were x = 24.65, y = 29.57, z = 55.94. Re-docking of the co-crystallized ligand into the receptor grid-defined active site demonstrated a close agreement between the experimental crystallographic pose and the predicted binding conformation, yielding a root-mean-square deviation (RMSD) of 1.846 Å (Figure 4). This RMSD value falls below the widely accepted validation threshold of 2.0 Å, indicating that the docking protocol accurately reproduced the native binding mode and is therefore reliable for predicting ligand binding within the sterol carrier protein-2 (SCP-2) active site. A total of 101 GC–MS-identified compounds were screened against sterol carrier protein-2 (SCP-2) using a hierarchical molecular docking workflow comprising High-Throughput Virtual Screening (HTVS), which identified 78 promising lead compounds. These lead compounds were subsequently evaluated using Glide Standard Precision (SP) docking, followed by Extra Precision (XP) docking to improve the accuracy and selectivity of binding pose prediction. The 15 highest-ranked compounds, together with the co-crystallized ligand, are presented in Table 7. The selected phytochemicals exhibited docking scores ranging from −7.338 to −10.091 kcal/mol, indicating favorable binding affinities toward the SCP-2 binding pocket. Notably, (5Z,9Z)-farnesyl acetone, a sesquiterpene constituent of *T. cinerariifolium*, achieved the most favorable docking score (−10.091 kcal/mol), followed by β-bisabolene and the co-crystallized ligand (−8.285 kcal/mol). These results indicate that (5Z,9Z)-farnesyl acetone and the other top phytochemicals exhibit favorable predicted binding interaction with the SCP-2 active site and highlights their potential as a promising inhibitor of the target protein.

As previously noted, (5Z,9Z)-farnesyl acetone exhibited the most favorable docking score (−10.091 kcal/mol), followed by the co-crystallized ligand (−8.285 kcal/mol), and was associated with a favorable MM-GBSA binding free energy (ΔG_bind = −70.23 kcal/mol), suggesting a stable predicted interaction with the SCP-2 binding site. Although the co-crystallized ligand exhibited the lowest binding free energy (ΔG_bind = −79.03 kcal/mol), tridecyl methyl ketone (docking score = −8.018 kcal/mol; ΔG_bind = −72.93 kcal/mol) and farnesol (−7.860 kcal/mol; ΔG_bind = −65.47 kcal/mol) also demonstrated favorable predicted binding. Similarly, β-bisabolene (−8.375 kcal/mol; ΔG_bind = −52.37 kcal/mol), α-zingiberene (−8.166 kcal/mol; ΔG_bind = −51.64 kcal/mol) and sesquilavandulol (−7.722 kcal/mol; ΔG_bind = −54.51 kcal/mol) displayed favorable docking scores that were generally supported by their MM-GBSA binding free energies, whereas α-copaene, cyclosativene, isogermacrene D and δ-elemene exhibited comparatively weak MM-GBSA binding free energies despite favorable docking scores. Collectively, these findings illustrate the complementary value of MM-GBSA calculations in refining docking-based predictions by providing additional insight into the relative stability of predicted protein–ligand complexes [36]. Collectively, the combined docking and MM-GBSA analyses identify (5Z,9Z)-farnesyl acetone, tridecyl methyl ketone, farnesol, β-bisabolene, α-zingiberene and sesquilavandulol as the most promising phytochemical candidates for targeting mosquito sterol carrier protein-2, supporting their potential to disrupt sterol transport and lipid metabolism essential for mosquito growth and development.

**Figure 5:**
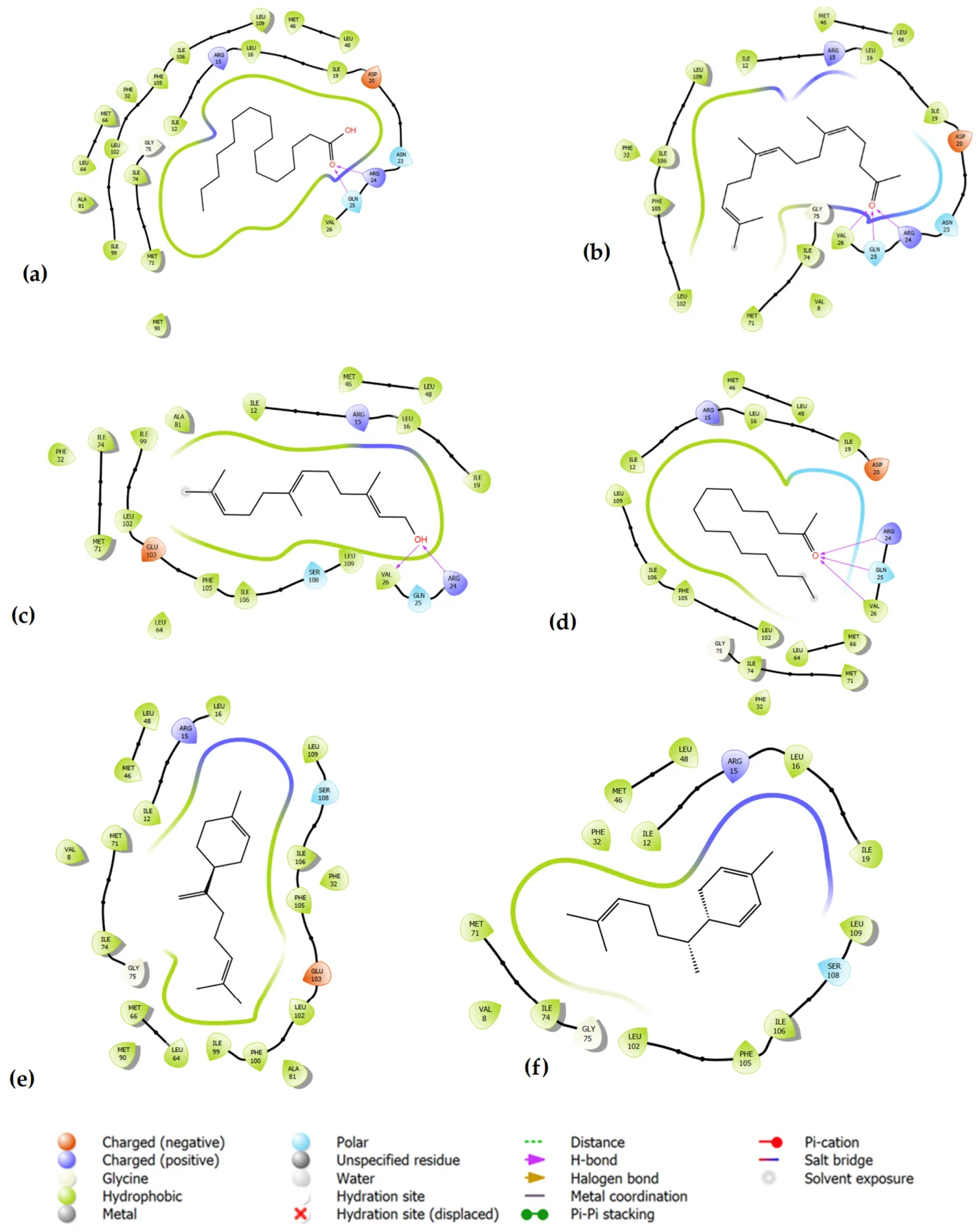
Two-dimensional (2D) visualization of protein–ligand interactions for top-ranked compounds and co-crystalized ligand within the 1PZ4 binding pocket: (**a**) co-crystalized ligand, (**b**) (5Z,9Z)-farnesyl acetone, (**c**) farnesol, (**d**) tridecyl methyl ketone, (**e**) β-bisabolene and (**f**) α-Zingiberene.

Two-dimensional (2D) interaction analysis within the sterol carrier protein-2 pocket (Figure 5) revealed that the top-ranked phytochemicals occupied the same binding cavity as the co-crystallized ligand within SCP-2 and were stabilized predominantly by hydrophobic interactions, reflecting the lipophilic nature of the sterol-binding pocket [37]. The co-crystallized ligand formed hydrogen bonds with ARG24 and GLN25, together with hydrophobic contacts involving LEU16, ILE19, LEU48, LEU64, MET46, MET66, MET71, ILE74, ALA81, ILE99, LEU102, PHE105, ILE106 and LEU109. A similar binding mode was observed for (5Z,9Z)-farnesyl acetone, tridecyl methyl ketone and farnesol, which each formed hydrogen bonds with ARG24 and GLN25 in addition to extensive hydrophobic interactions. These interaction profiles are consistent with their favorable MM-GBSA binding free energies, particularly for (5Z,9Z)-farnesyl acetone (−70.23 kcal/mol), tridecyl methyl ketone (−72.93 kcal/mol) and farnesol (−65.47 kcal/mol.). In contrast, the hydrocarbon sesquiterpenes β-bisabolene and α-zingiberene, which lack polar functional groups, interacted almost exclusively through hydrophobic contacts with residues including LEU16, LEU48, MET46, MET66, MET71, ILE74, LEU64, ILE99, LEU102, PHE105, ILE106 and LEU109, reflecting the predominantly hydrophobic nature of the SCP-2 binding pocket. Collectively, these findings indicate that the identified phytochemicals mimic the binding mode of the co-crystallized ligand, with hydrophobic interactions serving as the principal driving force for binding and hydrogen bonds providing additional stabilization for oxygenated compounds [35]. This interaction pattern supports the potential of these phytochemicals to competitively occupy the SCP-2 sterol-binding cavity and interfere with sterol transport, a process that is essential for mosquito growth, development, and survival.

The molecular docking analyses demonstrated that the phytochemicals identified from *C. citratus*, *T. cinerariifolium* and *S. rosmarinus* exhibited favorable binding affinities toward both juvenile hormone-binding protein (JHBP) and sterol carrier protein-2 (SCP-2), suggesting that these compounds may contribute to larvicidal activity through a multitarget mechanism. Several phytochemicals displayed favorable interactions with both proteins, although variations in docking scores and MM-GBSA binding free energies indicate differing degrees of target preference and binding stability. Notably, the majority of the top-ranked compounds were identified in the essential oil of *T. cinerariifolium*, suggesting that its sesquiterpene-rich phytochemical profile may have contributed to its superior larvicidal activity. Only a few high-ranking compounds originated from the other essential oils, including 1,10-di-epi-cubenol, tridecyl methyl ketone and geranic acid from *C. citratus* and thymol, p-cymene and carvone from *S. rosmarinus*. The predominance of *T. cinerariifolium*-derived phytochemicals among the highest-ranked ligands is consistent with its superior larvicidal activity observed in the bioassays, suggesting that its sesquiterpene-rich composition may underpin its enhanced inhibitory potential against multiple mosquito molecular targets.

Although the highest-ranked docking candidates were not always the most abundant constituents, analysis of the major phytochemicals identified by GC–MS provides additional insight into the larvicidal potential of each essential oil (Table 8). In *C. citratus*, the predominant constituents geranial (28.00%), neral (19.01%), α-pinene (11.83%) and 1,8-Cineole (11.76%) all exhibited moderate binding affinities toward both juvenile hormone-binding protein (JHBP) and sterol carrier protein-2 (SCP-2) (Table 7). Likewise, the sesquiterpene-rich essential oil of *T. cinerariifolium* was dominated by γ-muurolene (30.98%) and β-farnesene (27.73%). Of these, γ-muurolene displayed relatively strong binding to JHBP (−8.169 kcal/mol) and SCP-2 (−7.218 kcal/mol), whereas β-farnesene exhibited only moderate binding despite its high abundance. In *S. rosmarinus*, the major constituents α-pinene (21.96%), verbenone (13.42%), geraniol (5.60%), and borneol (4.94%) also showed moderate docking scores with both JHBP and SCP-2. Overall, these findings demonstrate that favorable protein–ligand interactions were not restricted to the most abundant constituents but were distributed among both major and minor phytochemicals. This observation suggests that the larvicidal potential of the investigated essential oils is influenced by their overall phytochemical composition rather than by the abundance of a single dominant constituent, providing a mechanistic basis for the differences in larvicidal activity observed among the three essential oils [38,39].

Hierarchical virtual screening revealed that a remarkably high proportion of the GC–MS-identified phytochemicals exhibited favorable affinity toward the selected mosquito targets. Of the 101 compounds screened, 88 (87.13%) and 78 (77.23%) progressed beyond HTVS against juvenile hormone-binding protein (JHBP) and sterol carrier protein-2 (SCP-2), respectively, indicating that most constituents possess the structural features required for productive interactions with these proteins. This widespread target recognition suggests that the larvicidal activity of the investigated essential oils is unlikely to be mediated by a single dominant constituent, but rather by the collective action of multiple phytochemicals with varying binding affinities and target preferences. Such a multitarget mode of action is characteristic of essential oils, whose biological activities arise from the additive or synergistic interactions of numerous constituents rather than from individual compounds acting alone [38,39]. The ability of a large proportion of the identified phytochemicals to interact with two distinct proteins involved in juvenile hormone transport and sterol metabolism further supports the hypothesis that these essential oils interfere with complementary physiological pathways essential for mosquito growth and development. This broad spectrum of molecular interactions may contribute to the superior larvicidal activity observed for *T. cinerariifolium* and *C. citratus*, while also reducing the likelihood of resistance development compared with single-target insecticides [40].

Farnesol, β-bisabolene, α-zingiberene, and sesquilavandulol consistently ranked among the highest-scoring compounds against both JHBP and SCP-2. In particular, farnesol achieved docking scores of −8.840 and −7.860 kcal/mol, with corresponding MM-GBSA binding free energies of −69.29 and −65.47 kcal/mol against JHBP and SCP-2, respectively. Two-dimensional interaction analysis further revealed that farnesol formed hydrogen bonds and extensive hydrophobic interactions within both binding pockets, contributing to the stability of the protein–ligand complexes. Its ability to interact strongly with two proteins involved in juvenile hormone transport and sterol homeostasis suggests a multitarget mode of action that could simultaneously disrupt complementary physiological pathways essential for mosquito larval development. Such multitarget activity is particularly advantageous in botanical insecticides, where the combined action of compounds against complementary biological pathways may enhance efficacy while reducing the likelihood of resistance development compared with single-target insecticides [40,41]. Given its consistent performance across both targets and its favorable binding energies, farnesol represents one of the most promising lead phytochemicals for further investigation as a mosquito larvicidal agent.

### 2.4. Insecticide-Likeness

Evaluation of the physicochemical properties of the lead phytochemicals according to Tice’s insecticide-likeness criteria demonstrated that all four compounds, farnesol, β-bisabolene, α-zingiberene, and sesquilavandulol, satisfied the recommended thresholds without any rule violations, indicating favorable characteristics for insecticidal activity (Table 9). All compounds possessed relatively low molecular weights (204.19–222.20 g/mol), which are advantageous for diffusion through the insect cuticle and access to intracellular targets [32,42]. Likewise, their lipophilicity (logP = 4.42–5.21) fell within the acceptable range, suggesting an appropriate balance between membrane permeability and aqueous solubility. Farnesol and sesquilavandulol each contained one hydrogen bond donor and one hydrogen bond acceptor, with moderate topological polar surface areas (20.23 Å^2^), potentially enabling additional hydrogen-bonding interactions with target proteins while maintaining good permeability [42]. In contrast, the hydrocarbon sesquiterpenes β-bisabolene and α-zingiberene lacked hydrogen-bonding functionalities (TPSA = 0 Å^2^) but retained favorable insecticide-likeness owing to their hydrophobic nature, which may facilitate penetration through the lipid-rich insect cuticle. Collectively, these results indicate that the identified lead compounds possess physicochemical properties consistent with those of successful insecticidal molecules, supporting their potential for further development as botanical larvicidal agents. Their favorable insecticide-likeness, together with their strong docking affinities and binding stability, further reinforces their suitability as multitarget inhibitors of mosquito proteins.

## 3. Materials and Methods

### 3.1. Reagents and Materials

All reagents and solvents used were of analytical grade obtained from Sigma-Aldrich. Petroleum ether was of b.p. 40–60 °C range, and diethyl ether was peroxide-free. *An. arabiensis* mosquito colonies were obtained from Kenya Medical Research Institute (KEMRI) and their larvae were reared at the University of Nairobi Insectary. *C. citratus* and *S. rosmarinus* samples were obtained from Tharaka Nithi County, Kenya whereas *T. cinerariifolium* samples were obtained from the neighboring Meru County. *A. indica* seeds were obtained from Tharaka University’s main campus grounds. The leaf samples were identified by a taxonomist from the National Museums of Kenya, and a voucher specimen was deposited in the Tharaka University Herbarium.

### 3.2. Sample Preparation and Extraction

Essential oils were extracted from the plant samples by hydrodistillation at 40–60 °C for 2–3 h. The extraction procedure was repeated three times per batch to ensure adequate oil recovery. For *A. indica* oil, the lean, air-dried seeds were first dehulled and ground into powder. The powder was further air-dried between 40–50 °C to remove residual moisture prior to extraction. The sample was then subjected to solvent extraction by maceration. Briefly, the seed powder was soaked in petroleum ether at a solvent-to-sample ratio of 1:5 (*w*/*v*). The mixture was kept at room temperature for 24 h with intermittent shaking to enhance extraction efficiency. The extraction procedure was repeated thrice for the same sample. After exhaustive maceration, the mixture was filtered using Whatman No. 1 filter paper to remove solid residues. The filtrate was then concentrated under reduced pressure using a rotary evaporator at 40–50 °C to recover the solvent and obtain the crude fixed oil. The extracted oil was left to dry further in a fume chamber to remove residual solvent and stored in amber vials at 4 °C until analysis.

### 3.3. Larvicidal Assay

The larvicidal activity was tested based WHO guidelines for laboratory and field-testing of larvicides [43]. All the oil samples were tested at 4000, 2000, 1000, 500 and 250 mg/L to get the activity range. Briefly, batches of twenty-five third- and fourth-instar larvae were transferred into 100 mL beakers containing an appropriate concentration of the test solution and distilled water. *A. indica* (neem) seed oil was used as a positive control while ethanol in distilled water was used as a negative control. *A. indica* oil was used as the positive control owing to its well-documented larvicidal activity against mosquito vectors, including *Anopheles* species, and its widespread use as a reference botanical insecticide in larvicidal bioassays [44,45]. The experiment was conducted for 72 h period under optimal conditions (a temperature between 24–32 °C and 85% relative humidity) in the insectary. Each experiment was conducted in triplicate. The number of dead larvae was counted after a 24-, 48- and 72-h exposure and the mortality rate was calculated per the replicates.

### 3.4. GC-MS Analysis

Each essential oil sample was diluted to 0.2% (*v*/*v*) in 100% hexane and filtered through a 0.22 µm PTFE syringe filter into a clean autosampler vial prior to analysis. A 1 µL aliquot of each sample was injected into the GC–MS system. Compound separation was achieved using an SH-Rxi-5Sil MS capillary column (30 m × 0.25 mm i.d., 0.25 µm film thickness). Ultrapure helium served as the carrier gas at a flow rate of 0.7 mL/min. The injector temperature was maintained at 280 °C, and samples were introduced in split mode at a ratio of 10:1. The oven temperature program was set from 50 °C (0 min hold) to 330 °C at a rate of 3 °C/min, resulting in a total run time of 93 min. Mass spectra were acquired in full-scan mode over a mass range of 50–500 m/z, with a solvent cut time of 3.8 min. The ion source and interface temperatures were maintained at 200 °C and 250 °C, respectively. Identification of the eluted compounds was performed by comparing the acquired mass spectra with reference spectral data. For *C. citratus*, *T. cinerariifolium*, and *S. rosmarinus*, compound assignments were based on comparison with the FFNSC-3 (Flavors and Fragrances, Natural and Synthetic Compounds) library. Chemical constituents were assigned using an integrated assessment of GC–MS spectral matches, similarity index (SI) scores, and retention indices (RI). The complete GC–MS dataset for the three essential oils, including retention times, retention indices, similarity indices, relative abundances, and compound identifications, together with the corresponding total ion chromatograms (TICs), is provided in Supplementary Materials.

### 3.5. Molecular Docking Analyses

#### 3.5.1. Target Protein Preparation

The three-dimensional crystal structures of: juvenile hormone-binding protein (pdb: 5V13) and sterol carrier protein-2 (pdb:1PZ4) were retrieved from the protein data bank (https://www.rcsb.org). Protein preparation step for each target was performed using the Protein Preparation Wizard module in the Schrödinger Suite (Release 2021-2). The retrieved structure was pre-processed by assigning proper bond orders, adding missing hydrogen atoms, and correcting incomplete side chains and missing residues. Missing loops and side chains were also reconstructed using the Prime module. Water molecules located beyond 5 Å from the co-crystallized ligand or not involved in critical bridging interactions were removed. The hydrogen-bonding network was subsequently optimized at physiological pH of 7.4 using the PROPKA empirical prediction algorithm. Finally, restrained energy minimization was carried out with an OPLS4 force field.

#### 3.5.2. Receptor Grid Generation

Receptor grid generation was accomplished using the Receptor Grid Generation panel, with the binding site defined by co-crystallized ligand coordinates. The Grid boxes were centered on the identified binding sites with dimensions of 20 × 20 × 20 Å to accommodate ligand flexibility while maintaining computational efficiency. The Van der Waals scaling factors were set at 1.0 for receptor atoms and at 0.8 for ligand atoms to account for minor conformational adjustments during docking.

#### 3.5.3. Ligand Preparation

Compounds identified through GC–MS analysis of the essential oils from each plant species were tabulated in Microsoft Excel, annotated with their corresponding Simplified Molecular Input Line Entry System (SMILES) strings, and exported as a CSV file for subsequent in silico analyses. The compounds together with the corresponding co-crystallized ligands of the respective protein targets were prepared using the LigPrep module of the Schrödinger Suite. Ionization states, protonation patterns, and tautomeric forms were generated at physiological conditions (pH = 7.4 ± 0.5) using Epik. Stereoisomer generation was permitted where applicable, yielding a total of 101 energetically minimized three-dimensional ligand conformations from the original candidate pool.

#### 3.5.4. Ligand Docking

Initially, all the prepared 101 ligand conformations obtained after LigPrep processing were subjected to High-Throughput Virtual Screening (HTVS). Compounds that demonstrated favorable binding characteristics during the HTVS phase were subsequently advanced to Standard Precision (SP) docking, and later to Extra Precision (XP) docking. The co-crystallized ligand of each protein target was concurrently docked under the same XP docking conditions to serve as a benchmark for comparative binding assessment.

#### 3.5.5. MM-GBSA Binding Free Energy Rescoring

To improve the accuracy of binding affinity prediction beyond docking scores alone, the top XP-docked protein–ligand complexes alongside the co-crystallized ligand of each protein target were subjected to Molecular Mechanics-Generalized Born Surface Area (MM-GBSA) binding free energy calculations using the Prime MM-GBSA module in Schrödinger. Calculations were performed using the OPLS4 force field in combination with the VSGB solvation model. The binding free energy (${∆G}_{bind})$ was estimated according to the following equation:

$${∆G}_{bind}=G_{complex}-\left(G_{receptor}+G_{ligand}\right)$$

where $G_{complex}$, $G_{receptor}$, and $G_{ligand}$ represent the minimized free energies of the protein–ligand complex, the isolated receptor, and the unbound ligand, respectively.

#### 3.5.6. Docking Protocol Validation

Validation of the docking protocol was carried out by extracting, preparing and re-docking of the co-crystalized ligand into the original active site of each of the target proteins to validate the reliability of the docking procedure. This was done for every protein target.

#### 3.5.7. Pesticide-Likeness Analysis (CoPLA)

Physicochemical properties of each of the top compounds were evaluated in-silico using the AdmetLab 3.0 web server [46]. The canonical SMILES representations of all selected compounds were individually submitted to the platform to assess their compliance with Tice’s rules of insecticide-likeness [47]. Six key molecular descriptors were evaluated to determine their desirability, including molecular weight (MW), calculated lipophilicity (clogP), number of hydrogen bond donors (HBD), number of hydrogen bond acceptors (HBA), number of rotatable bonds (RB), and topological polar surface area (TPSA).

### 3.6. Statistical Analysis

All experiments were done in triplicates, and mortality rates reported as mean values. The results were expressed as mean ± SE (standard error). The results were subjected to one-way ANOVA using Minitab 22.4.0. Median lethal concentration (LC_50_) values were estimated by nonlinear regression analysis, and concentration–response curves were generated using GraphPad Prism 11.0.2.

## 4. Conclusions

This study demonstrates that all essential oils exhibited concentration-dependent larvicidal activity, with *T. cinerariifolium* (LC_50_ = 511.0 mg/L; 95% CI: 473.3–547.1 mg/L) and *C. citratus* (LC_50_ = 602.7 mg/L; 95% CI: 559.3–648.5 mg/L) demonstrating the greatest potency. GC–MS analysis revealed distinct phytochemical profiles among the investigated essential oils, with sesquiterpene hydrocarbons predominating in *T. cinerariifolium* and oxygenated monoterpenes dominating *C. citratus* and *S*. *rosmarinus*. Hierarchical molecular docking and MM-GBSA analyses demonstrated that a substantial proportion of the identified phytochemicals exhibited favorable binding affinities toward mosquito juvenile hormone-binding protein (JHBP) and sterol carrier protein-2 (SCP-2), supporting a multitarget mode of action. Among the identified compounds, farnesol emerged as the most promising multitarget phytochemical, exhibiting consistently favorable binding affinities and binding free energies against both proteins, while 1,10-di-epi-cubenol and (5Z,9Z)-farnesyl acetone showed strong target-specific interactions with JHBP and SCP-2, respectively. Furthermore, the lead phytochemicals satisfied Tice’s insecticide-likeness criteria, indicating favorable physicochemical properties for insecticidal development. Collectively, these findings suggest that the larvicidal activity of the investigated essential oils is likely mediated by the combined action of multiple phytochemicals acting on complementary molecular targets involved in juvenile hormone transport and sterol metabolism. The integration of experimental bioassays with phytochemical profiling and computational analyses provides a mechanistic basis for the observed biological activity and highlights *T. cinerariifolium* and *C. citratus* as promising sources of environmentally sustainable botanical larvicides. Future studies should focus on isolating the most active constituents, validating their inhibitory activity through biochemical and in vivo assays, and investigating potential synergistic interactions among the constituent compounds to facilitate the development of effective mosquito control agents.

## Figures and Tables

**Table 1:** Mean percent larval mortality rate of *An. arabiensis* following 72 h exposure to essential oils from *C. citratus*, *T. cinerariifolium* and *S. rosmarinus*.

Essential Oil	Concentration (mg/L)	% Mortality		72 h-LC_50_ (mg/L)	95% CI (mg/L)	Hillslope	R^2^
		24 h	72 h				
*T. cinerariifolium*	250	5.33 ± 1.33	14.67 ± 1.33	511.0	473.3–547.1	3.80	0.9845
	500	14.67 ± 2.67	44 ± 6.11				
	1000	64.00 ± 6.11	100 ± 0				
	2000	100 ± 0.00	100 ± 0.00				
	4000	100 ± 0.00	100 ± 0.00				
*C. citratus*	250	8.00 ± 2.31	21.33 ± 1.33	602.7	559.3–648.5	1.96	0.9897
	500	14.66 ± 1.33	36.00 ± 2.31				
	1000	33.33 ± 2.67	73.33 ± 1.33				
	2000	42.67 ± 4.81	94.67 ± 1.33				
	4000	96.00 ± 2.31	100 ± 0.00				
*S. rosmarinus*	250	0.00	0.00	1537	1442–1638	1.59	0.9905
	500	5.33 ± 1.33	16.00 ± 2.31				
	1000	10.67 ± 3.53	34.67 ± 3.53				
	2000	8.00 ± 2.31	60.00 ± 2.31				
	4000	26.67 ± 3.53	81.33 ± 2.67				
Positive control (*A. indica*)	250	1.33 ± 1.33	2.67 ± 1.33	1763	1645–1892	1.33	0.9887
	500	5.33 ± 1.33	18.67 ± 1.33				
	1000	12.00 ± 00	30.67 ± 3.53				
	2000	25.33 ± 1.33	56.00 ± 2.32				
	4000	32.33 ± 2.31	73.33 ± 1.33				
**Negative control (water + ethanol)**		0.00	0.00				

**Table 2:** ANOVA results on the mortality of *An. arabiensis* mosquito larvae treated with various concentrations of *C. citratus*, *T. cinerariifolium, S. rosmarinus* and *A. indica* oils after 72 h exposure.

Variable Compared		F-Computed	*p*-Value
Concentration	Essential Oil		
250 mg/L	*T. cinerariifolium* vs. *A.indica*	40.5	0.003 *
	*C. citratus* vs. *A. indica*	98.00	0.001 *
	*S. rosmarinus* vs. *A. indica*	4.00	0.116
	*T. cinerariifolium* vs. *C. citratus*	12.50	0.024 *
500 mg/L	*T. cinerariifolium* vs. *A.indica*	16.41	0.015 *
	*C. citratus* vs. *A. indica*	42.25	0.003 *
	*S. rosmarinus* vs. *A. indica*	1.00	0.374
	*T. cinerariifolium* vs. *C. citratus*	1.50	0.228
1000 mg/L	*T. cinerariifolium* vs. *A.indica*	386.29	<0.001 *
	*C. citratus* vs. *A. indica*	128.00	<0.001 *
	*S. rosmarinus* vs. *A. indica*	0.64	0.468
	*T. cinerariifolium* vs. *C. citratus*	400.00	<0.001 *
2000 mg/L	*T. cinerariifolium* vs. *A.indica*	363.00	<0.001 *
	*C. citratus* vs. *A. indica*	210.25	<0.001 *
	*S. rosmarinus* vs. *A. indica*	1.50	0.288
	*T. cinerariifolium* vs. *C. citratus*	16.00	0.016 *
4000 mg/L	*T. cinerariifolium* vs. *A.indica*	400.00	<0.001 *
	*C. citratus* vs. *A. indica*	400.00	<0.001 *
	*S. rosmarinus* vs. *A. indica*	7.20	0.055
	*T. cinerariifolium* vs. *C. citratus*	-	-

At 0.05 level of significance, * implies that there is a significant difference.

**Table 3:** Chemical class composition of *C. citratus* essential oils identified by GC–MS Analysis.

Compound Class	Representative Identified Constituents			Total Relative Abundance (%)
	RI		Compound	
	Obs.	Lit.		
Oxygenated monoterpenes	1032	1032	1,8-Cineole (eucalyptol)	68.56
	1146	1149	Camphor	
	1171	1173	Borneol	
	1180	1184	Terpinen-4-ol	
	1205	1208	Verbenone	
	1225	1232	Citronellol	
	1237	1238	Neral	
	1267	1268	Geranial	
	1347	1342	Geranic acid	
Monoterpene hydrocarbons	933	933	α-Pinene	18.81
	949	953	Camphene	
	988	991	Myrcene	
	1006	1007	α-Phellandrene	
	1017	1018	α-Terpinene	
	1024	1025	p-Cymene	
	1029	1030	Limonene	
	1057	1058	γ-Terpinene	
	1085	1086	Terpinolene	
Oxygenated sesquiterpenes	1581	1587	Caryophyllene oxide	0.90
	1622	1614	1,10-Di-epi-cubenol	
	1663	1668	Intermedeol	
Other and unidentified compounds	1492	1495	Tridecan-2-one	11.59
	1965	1967	Tridecyl methyl ketone	

**Table 4:** Chemical class composition of *T. cinerariifolium* essential oils identified by GC–MS Analysis.

Compound Class	Representative Identified Constituents			Total Relative Abundance (%)
	RI		Compound	
	Obs.	Lit.		
Sesquiterpene hydrocarbons	1452	1452	β-Farnesene	79.0
	1481	1478	γ-Muurolene	
	1502	1504	α-Farnesene	
	1444	1447	Isogermacrene D	
	1376	1375	α-Copaene	
	1430	1433	β-copaene	
	1495	1497	Bicyclogermacrene	
	1539	1540	α-Bisabolene	
	1518	1518	δ-Cadinene	
	1512	1512	γ-Cadinene	
	1489	1496	α-Zingiberene	
	1507	1508	β-Bisabolene	
	1332	1335	δ-Elemene	
	1369	1367	Cyclosativene	
Oxygenated sesquiterpenes	1559	1569	Nerolidol	12.7
	1585	1592	Globulol,	
	1645	1651	α-Muurolol	
	1532	1536	*trans*-Cadina-1,4-diene	
	1654	1659	Cadin-4-en-10-ol	
	1725	1725	Farnesol	
	1623	1633	Sesquilavandulol	
	1642	1645	T-Muurolol	
Oxygenated monoterpenes	1154	1155	*trans*-Chrysanthemol	0.8
	1171	1173	Borneol	
	1275	1266	Chrysanthemyl acetate	
Monoterpene hydrocarbons	933	933	α-Pinene	0.07
Other and unidentified compounds	2498	2500	Pentacosane <n->	7.4

**Table 5:** Chemical class composition of *S. rosmarinus* essential oils identified by GC–MS Analysis.

Compound Class	Representative Identified Constituents			Total Relative Abundance (%)
	RI		Compound	
	Obs.	Lit.		
Oxygenated monoterpenes	1031	1032	1,8-Cineole (eucalyptol)	56.5
	1206	1208	Verbenone	
	1248	1255	Geraniol	
	1145	1149	Camphor,	
	1170	1173	Borneol	
	1179	1184	Terpinen-4-ol	
	1224	1232	Citronellol	
	1235	1238	Neral	
	1265	1268	Geranial	
	1069	1069	Sabinene hydrate	
	1241	1246	Carvone	
	1160	1164	Pinocarvone	
	1173	1176	Pinocamphone	
	1295	1293	Thymol	
Monoterpene hydrocarbons	933	933	α-Pinene	33.0
	987	991	Myrcene	
	1006	1007	α-Phellandrene	
	1057	1058	γ-Terpinene	
	1084	1086	Terpinolene	
	1088	1093	p-Cymene	
	922	923	Tricyclene	
	925	927	α-Thujene	
	952	953	Thuja-2,4(10)-diene	
Sesquiterpenes	1419	1424	(E)-Caryophyllene,	0.82
	1455	1454	α-Humulene,	
	1580	1587	Caryophyllene oxide	
Phenylpropanoids	1349	1357	Eugenol	0.45
	1396	1403	Methyl eugenol	
Others and unidentified compounds	798	797	Isobutenyl methyl ketone	9.2
	981	986	Hept-5-en-2-one <6-methyl->	

**Table 6:** Docking scores and MM-GBSA binding free energy results for the top 15 candidate compounds and the co-crystalized ligand, on Mosquito Juvenile Hormone-binding protein.

Compound	Docking Score (kcal/mol)	ΔG_bind_ (kcal/mol)
1,10-di-epi-Cubenol	−9.48	−34.31
Farnesol	−8.84	−69.29
α-Copaene	−8.77	−37.05
Globulol	−8.75	−7.89
β-Copaene	−8.64	−39.22
α-Muurolol	−8.57	6.33
(E)-α-Bisabolene	−8.49	−44.17
Sesquilavandulol	−8.43	−37.04
*trans*-Cadina-1,4-diene	−8.42	−35.04
β-Bisabolene	−8.34	−42.14
α-Zingiberene	−8.34	−52.87
Cyclosativene	−8.31	−18.65
δ-Cadinene	−8.28	−17.75
α-Cadinene	−8.26	−25.80
Nerolidol	−8.14	−55.07
Co-crystalized ligand	−9.99	−78.10

**Table 7:** Docking scores and MM-GBSA binding free energy results for the top 15 candidate compounds and the co-crystalized ligand, on Sterol Carrier Protein-2.

Compound	Docking Score (kcal/mol)	ΔG_bind_ (kcal/mol)
(5Z,9Z)-Farnesyl acetone	−10.09	−70.23
β-Bisabolene	−8.38	−52.37
α-Zingiberene	−8.17	−51.64
Tridecyl methyl ketone	−8.02	−72.93
β-Copaene	−7.94	−28.51
Cyclosativene	−7.90	−18.26
δ-Elemene	−7.86	−24.48
Farnesol	−7.86	−65.47
Carvone	−7.75	−45.40
Sesquilavandulol	−7.72	−54.51
Isogermacrene D	−7.67	−23.25
α-Copaene	−7.50	−9.08
Thymol	−7.45	−39.05
Geranic acid	−7.34	−46.63
p-Cymene	−7.31	−39.45
Co-crystalized ligand	−8.29	−79.03

**Table 8:** Relative abundance and molecular docking scores of the major phytochemical constituents identified by GC–MS from the essential oils of *C. citratus*, *T. cinerariifolium*, and *S. rosmarinus* against JHBP (5V13) and SCP-2 (1PZ4).

Essential Oil Source	Major Compound(s)	Abundance (%)	Docking Score (kcal/mol)	
			5V13	1PZ4
*C. citratus*	Geranial	28.00	−6.78	−6.74
	Neral	19.01	−7.09	−6.88
	α-Pinene	11.83	−7.01	−6.02
	1,8-Cineole (Eucalyptol)	11.76	−7.05	−6.67
*T. cinerariifolium*	Muurolene (gamma)	30.98	−8.17	−7.22
	β-Farnesene	27.73	−6.48	−6.64
	α-Farnesene	4.42	−7.02	−7.03
	Nerolidol	3.40	−8.15	−6.52
*S. rosmarinus*	α-Pinene	21.96	−7.01	−6.02
	Verbenon	13.42	−6.77	−6.27
	Geraniol	5.60	−7.21	−6.46
	Borneol	4.94	−6.94	−6.26

**Table 9:** Physicochemical properties and insecticide-likeness assessment of the lead phytochemicals based on Tice’s insecticide-likeness criteria.

Compound	MW (g/mol)	nHA	nHD	nRB	TPSA (Å^2^)	LogP	Tice Rule Violations
Farnesol	222.20	1	1	7	20.23	5.21	0
β-bisabolene	204.19	0	0	4	0.00	5.14	0
α-zingiberene	204.19	0	0	4	0.00	4.51	0
Sesquilavandulol	222.20	1	1	7	20.23	4.42	0

## Data Availability

The original contributions presented in this study are included in the article/Supplementary Material. Further inquiries can be directed to the corresponding authors.

## Supplementary Materials

The following supporting information can be downloaded at: https://www.mdpi.com/article/10.3390/molecules31183346/s1. Complete GC–MS dataset (File S1) and TIC chromatograms for *C. citratus* (Figure S1), *T. cinerariifolium* (Figure S2), and *S. rosmarinus* (Figure S3) essential oils.

## Abbreviations

Abbreviation	Full term
ANOVA	Analysis of variance
CSV	Comma-separated values
ΔG	Gibbs free energy
F	F-statistic
GC–MS	Gas chromatography–mass spectrometry
HBA	Hydrogen-bond acceptor
HBD	Hydrogen-bond donor
HTVS	High-throughput virtual screening
CI	Confidence interval
JHBP	Juvenile hormone-binding protein
LC_50_	Median lethal concentration
MM-GBSA	Molecular mechanics/generalized Born surface area
PDB	Protein Data Bank
*p*-value	Probability value
RB	Rotatable bond
RMSD	Root-mean-square deviation
R^2^	Coefficient of determination
SCP-2	Sterol carrier protein-2
SMILES	Simplified Molecular Input Line Entry System
SP	Standard precision
TPSA	Topological polar surface area
WHO	World Health Organization
XP	Extra precision
