# Supplementary material for: Larvicidal Activity, GC–MS Profiling and Multitarget Molecular Docking of Essential Oils from Cymbopogon citratus, Tanacetum cinerariifolium and Salvia rosmarinus Against Anopheles arabiensis
Source: Molecules. 2026 Sep 21;31(18):3346. doi: 10.3390/molecules31183346 (PMC13610486; doi:10.3390/molecules31183346)

Qualitative Analysis Report

Sample Information

Analyzed : 21/07/2025
C:\GCMSsolution\Data\Project1\21-07-25_LG304.qgd
Injection Volume : 1.00

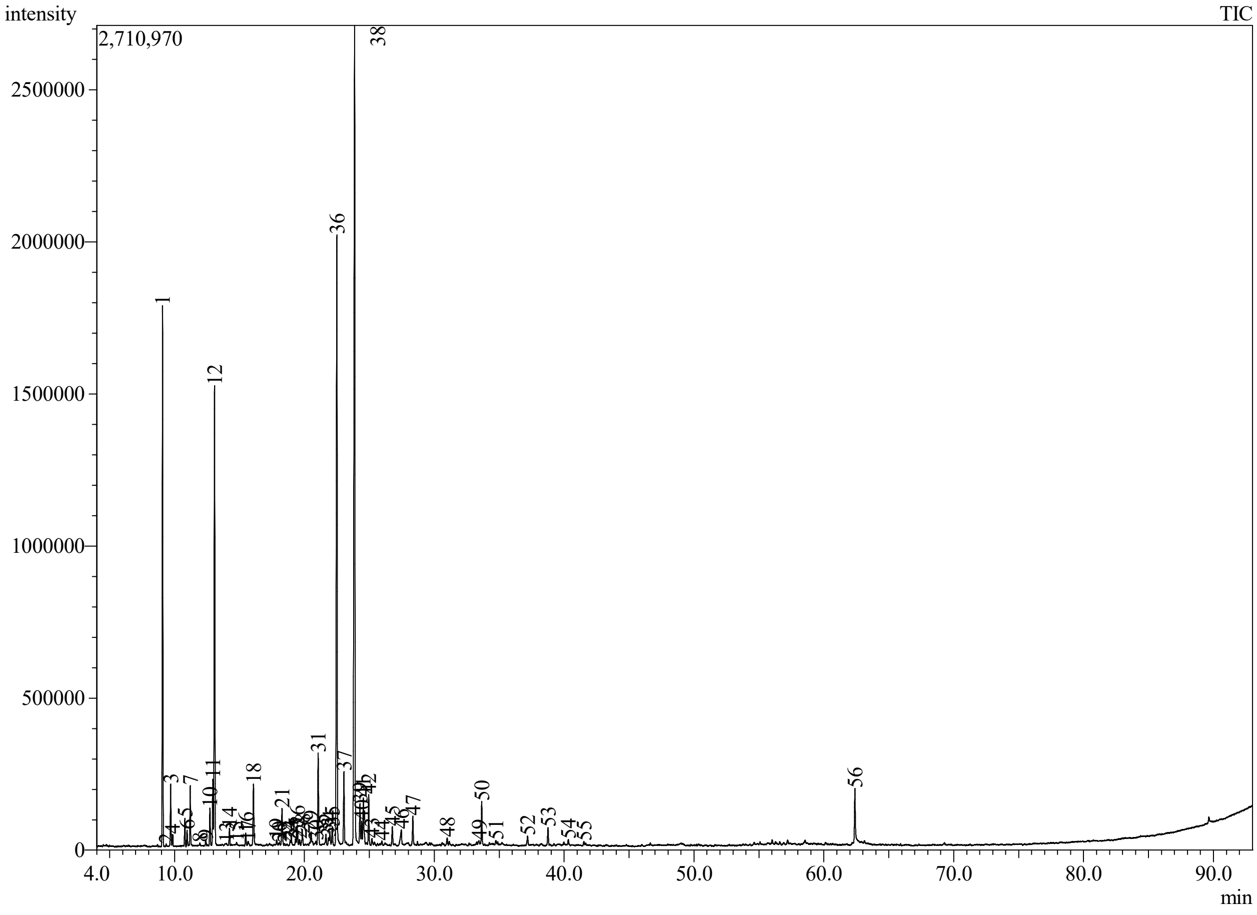

Qualitative Analysis Report

Library

<< Target >>
Line#:1 R.Time:9.075(Scan#:1219) Retention Index:933 MassPeaks:270
RawMode:Averaged 9.071-9.079(1218-1220) BasePeak:93.10(475483)
BG Mode:Calc. from Peak Group 1 - Event 1 Scan

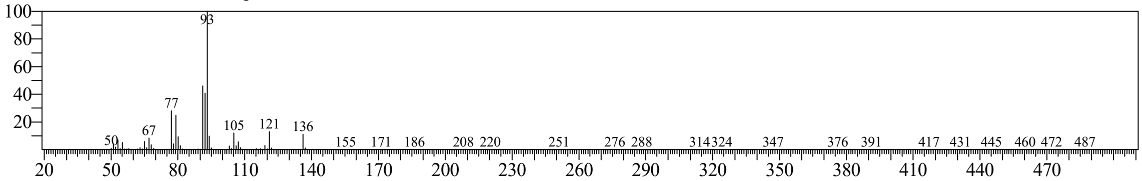

Hit#:1 Entry:817 Library:FFNSC 3.lib
SI:98 Formula:C10 H16 CAS:80-56-8 MolWeight:136 RetIndex:933
CompName:Pinene <alpha->

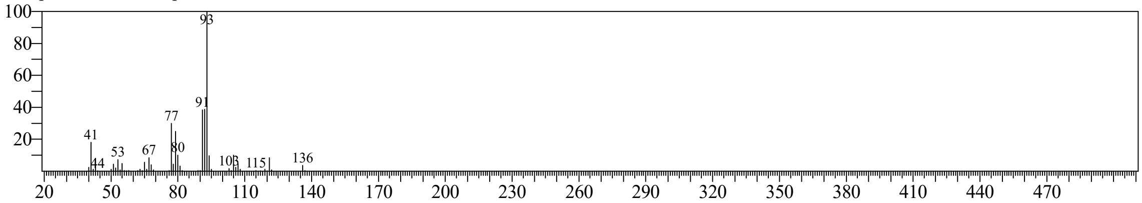

Hit#:2 Entry:986 Library:FFNSC 3.lib
SI:93 Formula:C10 H16 CAS:508-32-7 MolWeight:136 RetIndex:923
CompName:Tricyclene

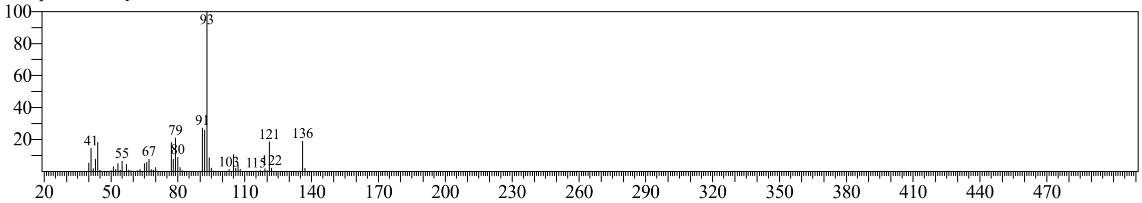

Hit#:3 Entry:3281 Library:FFNSC 3.lib
SI:93 Formula:C10 H16 CAS:4889-83-2 MolWeight:136 RetIndex:927
CompName:3-Methylapopinene

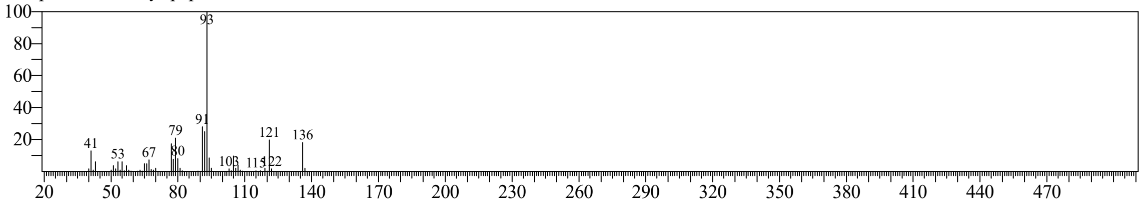

Hit#:4 Entry:24214 Library:NIST17-1.lib
SI:93 Formula:C10H16N2 CAS:87143-58-6 MolWeight:164 RetIndex:0
CompName:3,5-Methanocyclopentapyrazole, 3,3a,4,5,6,6a-hexahydro-3a,4,4-trimethyl-

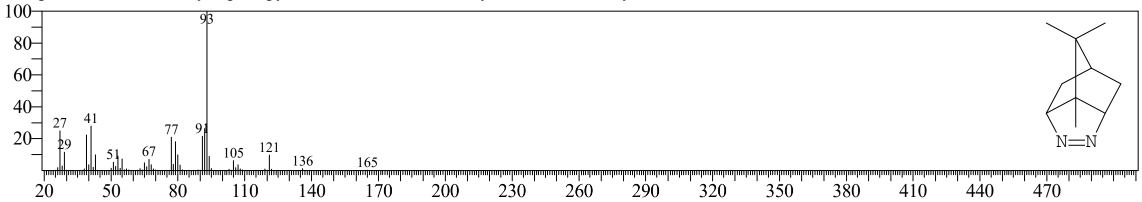

Hit#:5 Entry:957 Library:FFNSC 3.lib
SI:93 Formula:C10 H16 CAS:2867-05-2 MolWeight:136 RetIndex:927
CompName:Thujene <alpha->

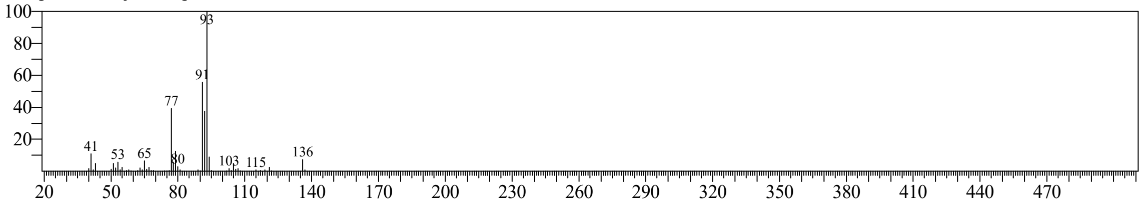

Qualitative Analysis Report

<< Target >>
Line#:2 R.Time:9.354(Scan#:1286) Retention Index:940 MassPeaks:234
RawMode:Averaged 9.350-9.358(1285-1287) BasePeak:55.05(1542)
BG Mode:Calc. from Peak Group 1 - Event 1 Scan

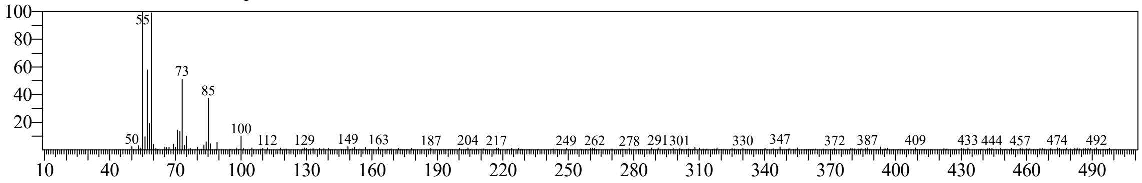

Hit#:1 Entry:46813 Library:NIST17-1.lib
SI:89 Formula:C3H4F5NOS CAS:80409-43-4 MolWeight:197 RetIndex:0
CompName:Acrylamidosulfur pentafluoride

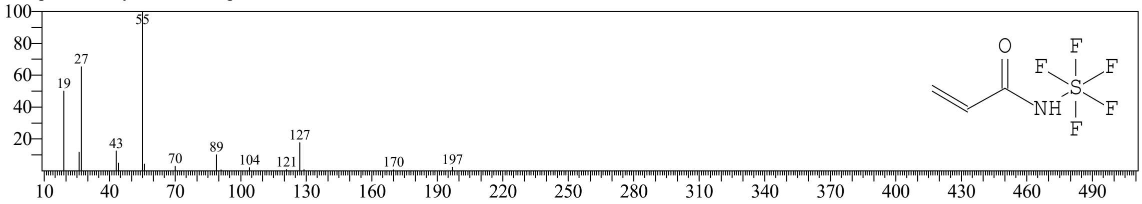

Hit#:2 Entry:5237 Library:NIST17-1.lib
SI:87 Formula:C6H14O2 CAS:82360-67-6 MolWeight:118 RetIndex:942
CompName:RS-2,3-hexanediol $$ 2,3-Hexanediol # $$

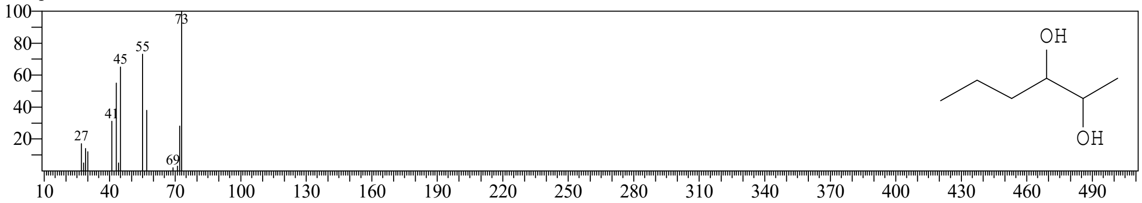

Hit#:3 Entry:5238 Library:NIST17-1.lib
SI:86 Formula:C6H14O2 CAS:22520-19-0 MolWeight:118 RetIndex:942
CompName:(SS)- or (RR)-2,3-hexanediol $$ 2,3-Hexanediol # $$

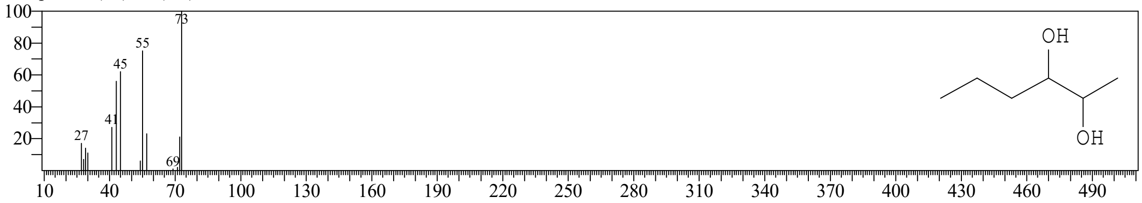

Hit#:4 Entry:659 Library:NIST17-1.lib
SI:84 Formula:C2H4N4 CAS:16681-77-9 MolWeight:84 RetIndex:0
CompName:1H-Tetrazole, 1-methyl- $$ N-Methyltetrazole $$ 1-Methyl-1H-tetrazole $$ 1-Methyltetrazole $$ 1-Methyl-1H-tetraazole # $$

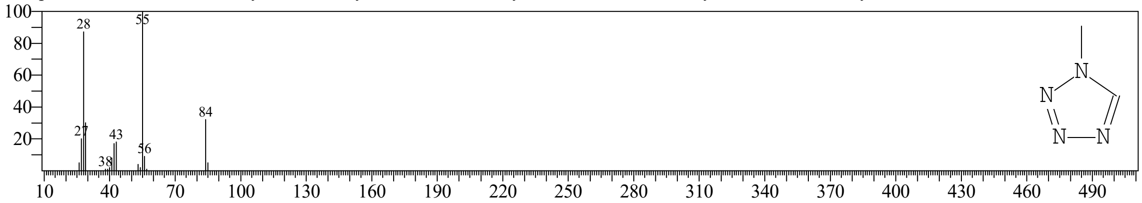

Hit#:5 Entry:87 Library:NIST17-1.lib
SI:84 Formula:C3H5N CAS:624-79-3 MolWeight:55 RetIndex:0
CompName:Ethyl isocyanide $$ Ethyl carbylamine $$ Ethane, isocyano- $$ Isocyanoethane $$ C2H5NC $$ Carbylamine, ethyl- $$ Ethylisokyanid $$ Ethyl

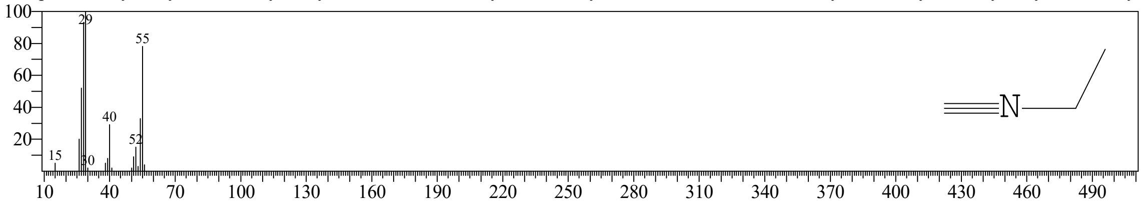

Qualitative Analysis Report

<< Target >>
Line#:3 R.Time:9.700(Scan#:1369) Retention Index:949 MassPeaks:254
RawMode:Averaged 9.696-9.704(1368-1370) BasePeak:93.10(40228)
BG Mode:Calc. from Peak Group 1 - Event 1 Scan

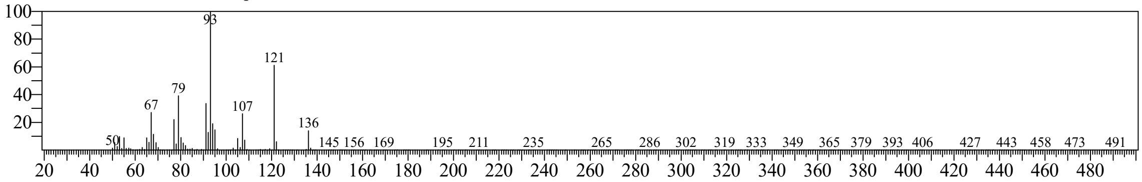

Hit#:1 Entry:952 Library:FFNSC 3.lib
SI:99 Formula:C10 H16 CAS:79-92-5 MolWeight:136 RetIndex:953
CompName:Camphene

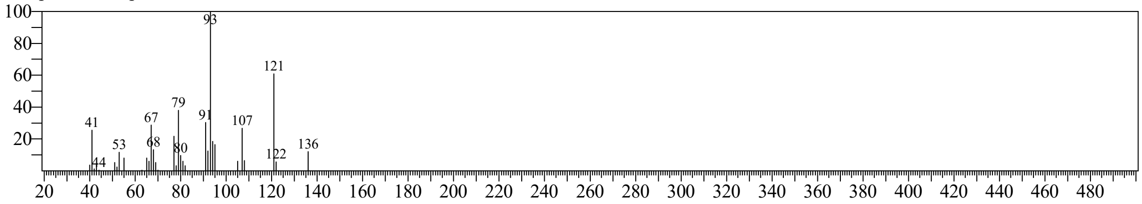

Hit#:2 Entry:3282 Library:FFNSC 3.lib
SI:99 Formula:C10 H16 CAS:497-32-5 MolWeight:136 RetIndex:954
CompName:2,2-Dimethyl-5-methylene norbornane

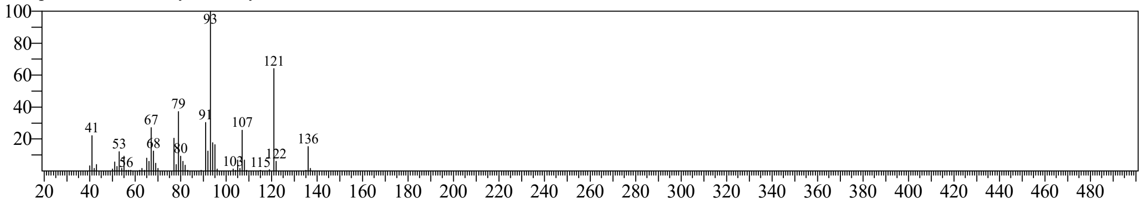

Hit#:3 Entry:10395 Library:NIST17-1.lib
SI:96 Formula:C10H16 CAS:5794-04-7 MolWeight:136 RetIndex:943
CompName:Bicyclo[2.2.1]heptane, 2,2-dimethyl-3-methylene-, (1S)- $$ 2,2-Dimethyl-3-methylenebicyclo[2.2.1]heptane $$

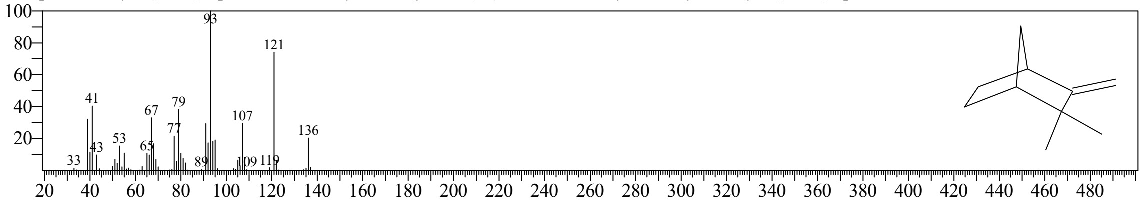

Hit#:4 Entry:10401 Library:NIST17-1.lib
SI:92 Formula:C10H16 CAS:1727-69-1 MolWeight:136 RetIndex:956
CompName:Cyclopentene, 4-ethenyl-1,5,5-trimethyl- $$ 1,5,5-Trimethyl-4-vinyl-1-cyclopentene # $$

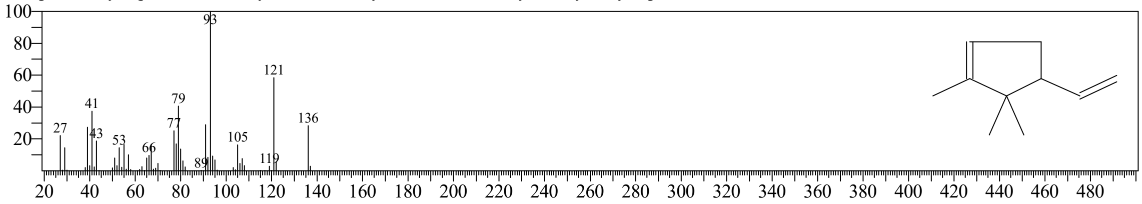

Hit#:5 Entry:10373 Library:NIST17-1.lib
SI:91 Formula:C10H16 CAS:471-84-1 MolWeight:136 RetIndex:943
CompName:Bicyclo[2.2.1]heptane, 7,7-dimethyl-2-methylene- $$ Norbornane, 7,7-dimethyl-2-methylene- $$ .alpha.-Fenchene $$ Fenchene $$ 7,7-Dimethy

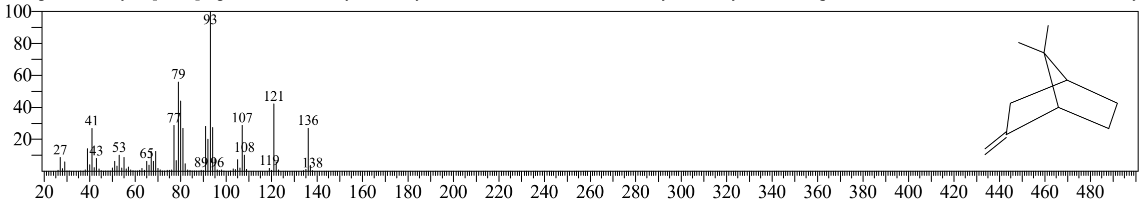

Qualitative Analysis Report

<< Target >>
Line#:4 R.Time:9.833(Scan#:1401) Retention Index:953 MassPeaks:222
RawMode:Averaged 9.829-9.838(1400-1402) BasePeak:91.10(10874)
BG Mode:Calc. from Peak Group 1 - Event 1 Scan

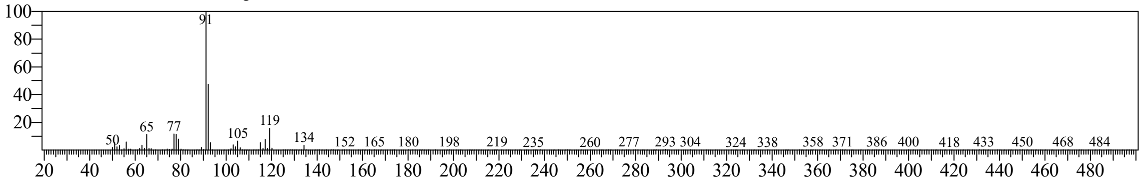

Hit#:1 Entry:556 Library:FFNSC 3.lib
SI:98 Formula:C10 H14 CAS:36262-09-6 MolWeight:134 RetIndex:953
CompName:Thuja-2,4(10)-diene

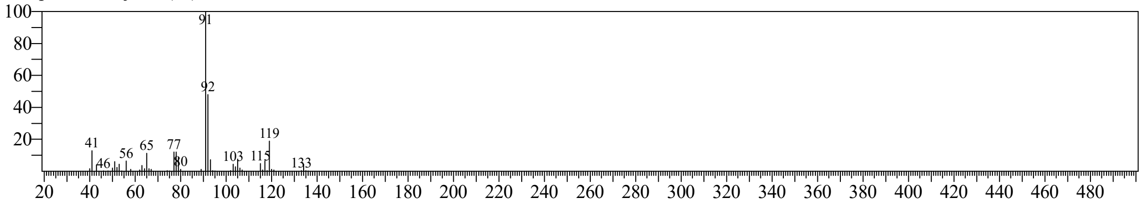

Hit#:2 Entry:231901 Library:NIST17-1.lib
SI:92 Formula:C13H14Fe2O4S2 CAS:12215-56-4 MolWeight:410 RetIndex:0
CompName:Iron, [(2,3,5,6-.eta.)-bicyclo[2.2.1]hepta-2,5-diene]tetracarbonylbis[.mu.-(methanethiolato)]di-, (Fe-Fe)

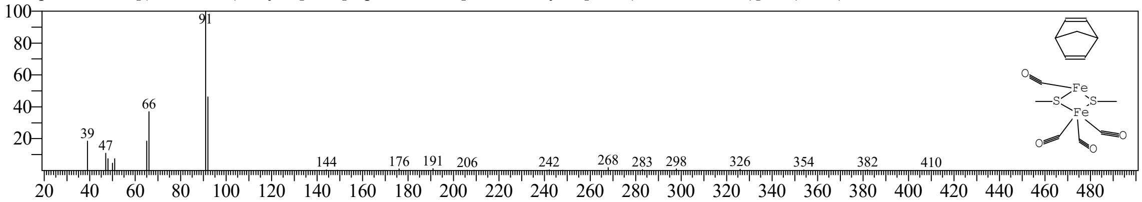

Hit#:3 Entry:5580 Library:NIST17-1.lib
SI:89 Formula:C9H12 CAS:17634-51-4 MolWeight:120 RetIndex:946
CompName:1,3,5-Cycloheptatriene, 7-ethyl- $$ 7-Ethyl-1,3,5-cycloheptatriene $$ 7-Ethylcycloheptatriene $$

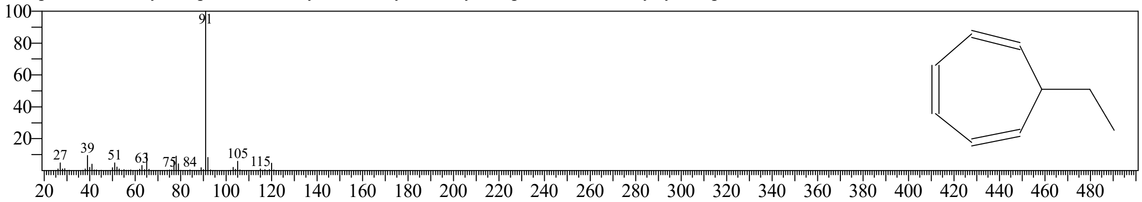

Hit#:4 Entry:93293 Library:NIST17-1.lib
SI:89 Formula:C10H10FeO4 CAS:32716-70-4 MolWeight:250 RetIndex:0
CompName:Iron, tricarbonyl[(2,3,4,5-.eta.)-2,4-cycloheptadien-1-ol]-

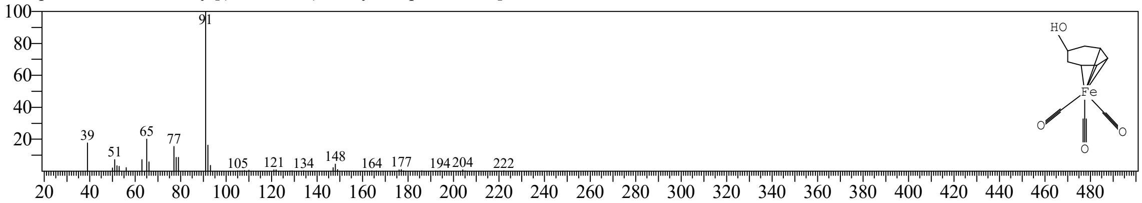

Hit#:5 Entry:168210 Library:NIST17-1.lib
SI:89 Formula:C13H7N7O4 CAS:1394125-25-7 MolWeight:325 RetIndex:0
CompName:12-Benzyl-4,9,15-trioxa-3,5,8,10,12,14,16-heptaazatetracyclo[11.3.0.0(2,6).0(7,11)]hexadeca-1(16),2,5,7,10,13-hexaen-3-one

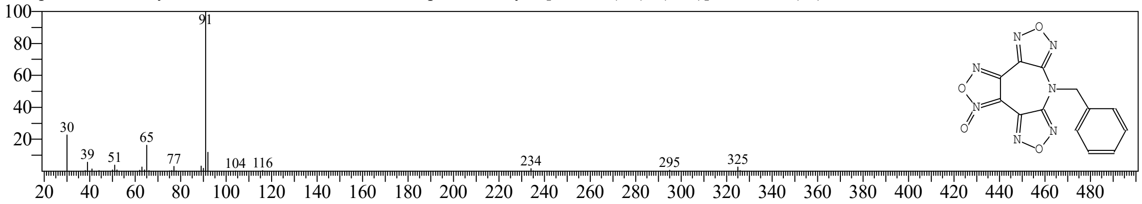

Qualitative Analysis Report

<< Target >>
Line#:5 R.Time:10.788(Scan#:1630) Retention Index:977 MassPeaks:290
RawMode:Averaged 10.783-10.792(1629-1631) BasePeak:93.10(24359)
BG Mode:Calc. from Peak Group 1 - Event 1 Scan

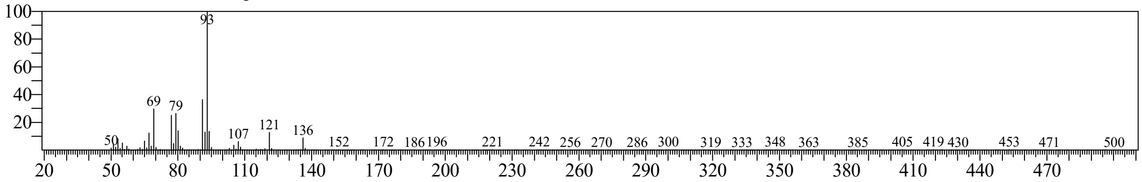

Hit#:1 Entry:837 Library:FFNSC 3.lib
SI:96 Formula:C10 H16 CAS:127-91-3 MolWeight:136 RetIndex:978
CompName:Pinene <beta->

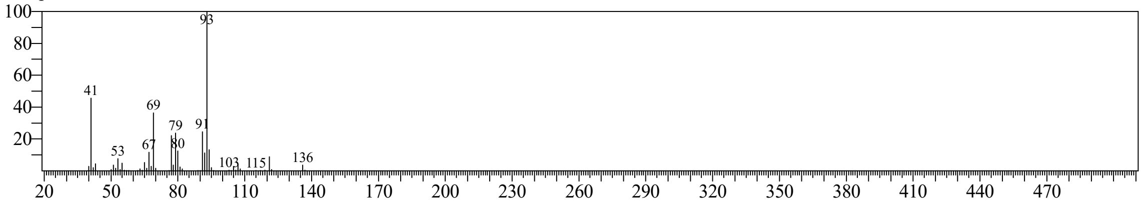

Hit#:2 Entry:958 Library:FFNSC 3.lib
SI:94 Formula:C10 H16 CAS:3387-41-5 MolWeight:136 RetIndex:972
CompName:Sabinene

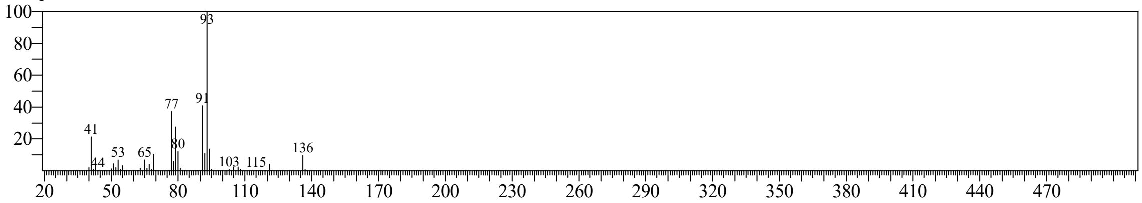

Hit#:3 Entry:10357 Library:NIST17-1.lib
SI:92 Formula:C10H16 CAS:3779-61-1 MolWeight:136 RetIndex:976
CompName:trans-.beta.-Ocimene $$ 1,3,6-Octatriene, 3,7-dimethyl-, (E)- $$ .beta.-trans-Ocimene $$ trans-3,7-Dimethyl-1,3,6-Octatriene $$ Ocimene, trans

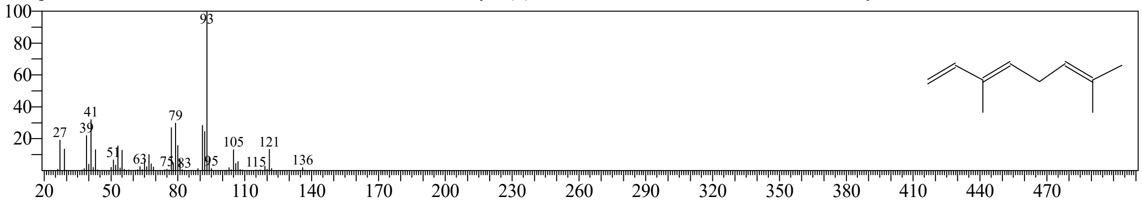

Hit#:4 Entry:10358 Library:NIST17-1.lib
SI:91 Formula:C10H16 CAS:3338-55-4 MolWeight:136 RetIndex:976
CompName:1,3,6-Octatriene, 3,7-dimethyl-, (Z)- $$ .beta.-cis-Ocimene $$ cis-.beta.-Ocimene $$ cis-3,7-Dimethyl-1,3,6-octatriene $$ Ocimene, cis-.beta.- $

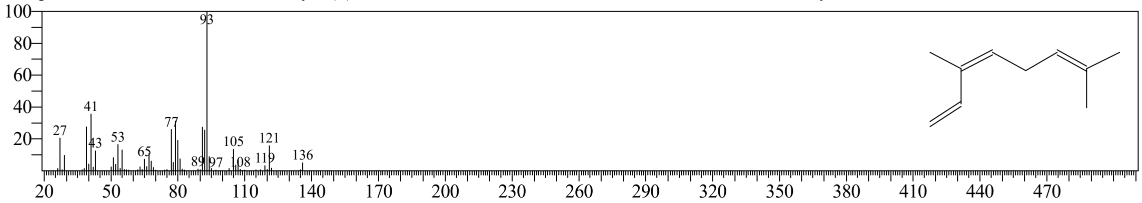

Hit#:5 Entry:24214 Library:NIST17-1.lib
SI:91 Formula:C10H16N2 CAS:87143-58-6 MolWeight:164 RetIndex:0
CompName:3,5-Methanocyclopentapyrazole, 3,3a,4,5,6,6a-hexahydro-3a,4,4-trimethyl-

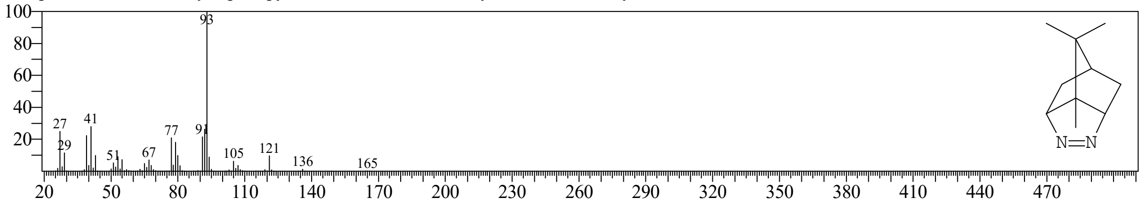

Qualitative Analysis Report

<< Target >>
Line#:6 R.Time:10.963(Scan#:1672) Retention Index:982 MassPeaks:341
RawMode:Averaged 10.958-10.967(1671-1673) BasePeak:108.10(7660)
BG Mode:Calc. from Peak Group 1 - Event 1 Scan

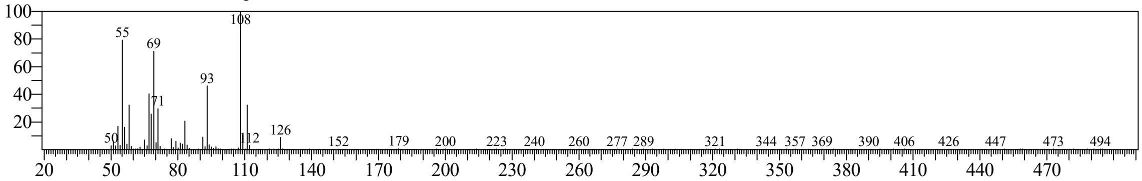

Hit#:1 Entry:675 Library:FFNSC 3.lib
SI:96 Formula:C8 H14 O CAS:110-93-0 MolWeight:126 RetIndex:986
CompName:Hept-5-en-2-one <6-methyl->

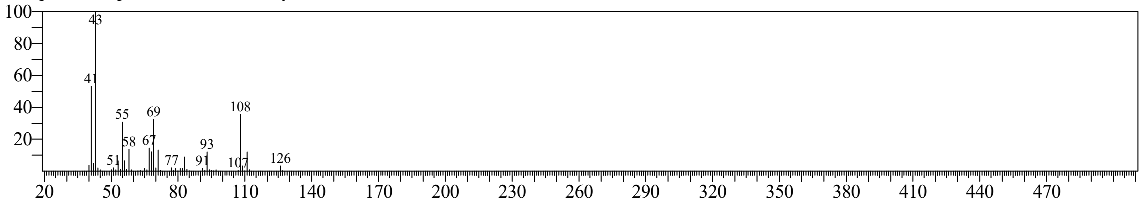

Hit#:2 Entry:110970 Library:NIST17-1.lib
SI:85 Formula:C14H16CrO2 CAS:57820-92-5 MolWeight:268 RetIndex:0
CompName:Chromium, bis(anisole)-

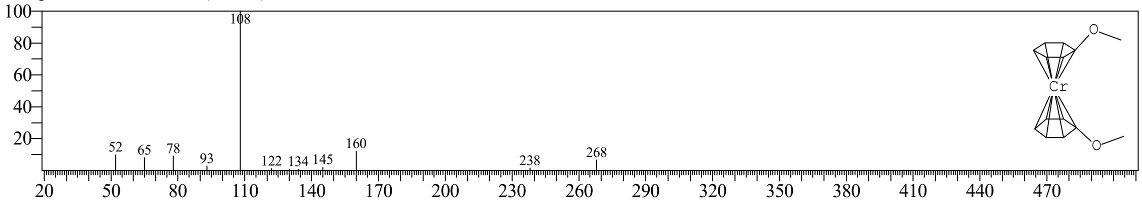

Hit#:3 Entry:2939 Library:NIST17-1.lib
SI:83 Formula:C6H8Si CAS:63878-65-9 MolWeight:108 RetIndex:0
CompName:Silabenzene,1-methyl- $$ 1-Methyl-1-silabenzene $$ 1-Methylsiline # $$

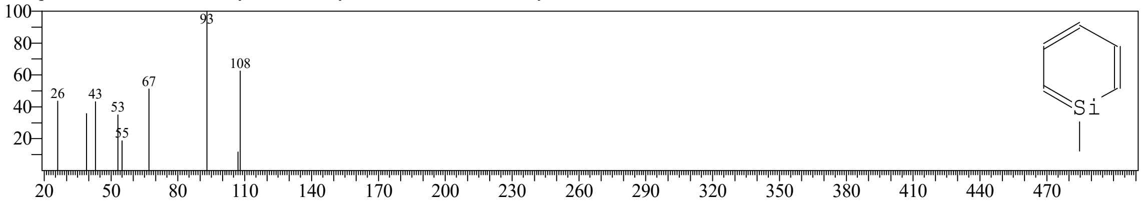

Hit#:4 Entry:6536 Library:NIST17-1.lib
SI:82 Formula:C7H11NO CAS:0-00-0 MolWeight:125 RetIndex:0
CompName:Pyridinium, 1-ethyl-, hydroxide

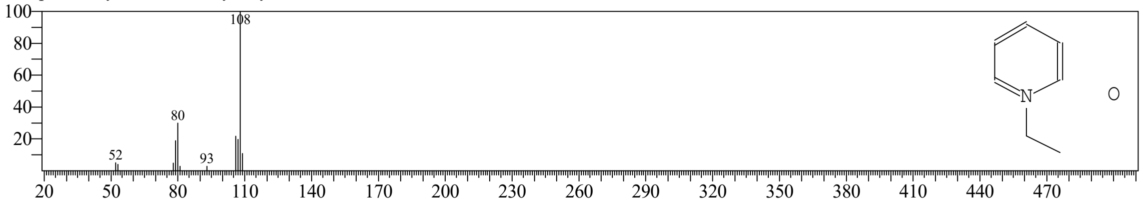

Qualitative Analysis Report

<< Target >>
Line#:7 R.Time:11.204(Scan#:1730) Retention Index:988 MassPeaks:220
RawMode:Averaged 11.200-11.208(1729-1731) BasePeak:93.10(54829)
BG Mode:Calc. from Peak Group 1 - Event 1 Scan

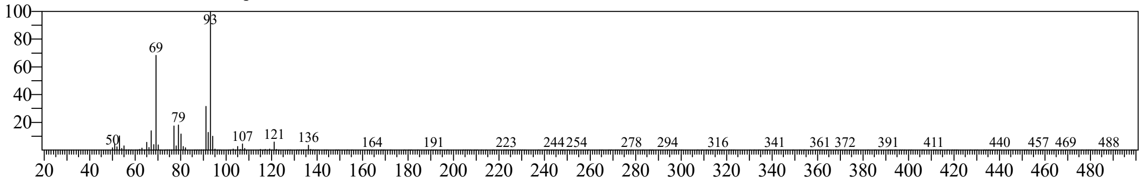

Hit#:1 Entry:628 Library:FFNSC 3.lib
SI:97 Formula:C10 H16 CAS:123-35-3 MolWeight:136 RetIndex:991
CompName:Myrcene

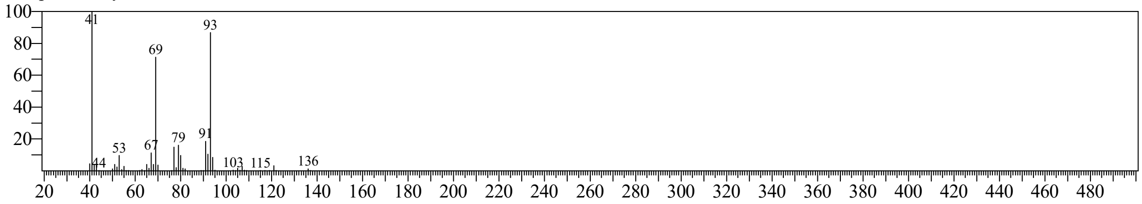

Hit#:2 Entry:837 Library:FFNSC 3.lib
SI:94 Formula:C10 H16 CAS:127-91-3 MolWeight:136 RetIndex:978
CompName:Pinene <beta->

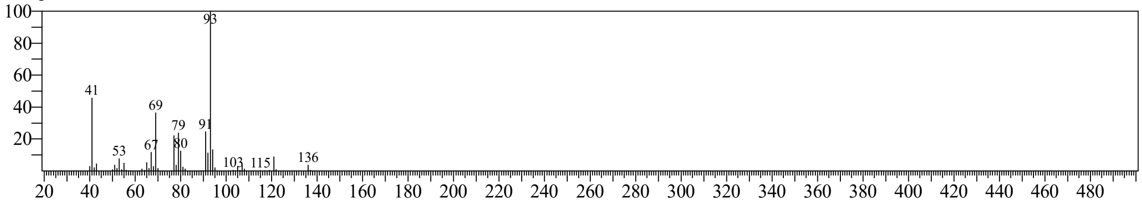

Hit#:3 Entry:24214 Library:NIST17-1.lib
SI:90 Formula:C10H16N2 CAS:87143-58-6 MolWeight:164 RetIndex:0
CompName:3,5-Methanocyclopentapyrazole, 3,3a,4,5,6,6a-hexahydro-3a,4,4-trimethyl-

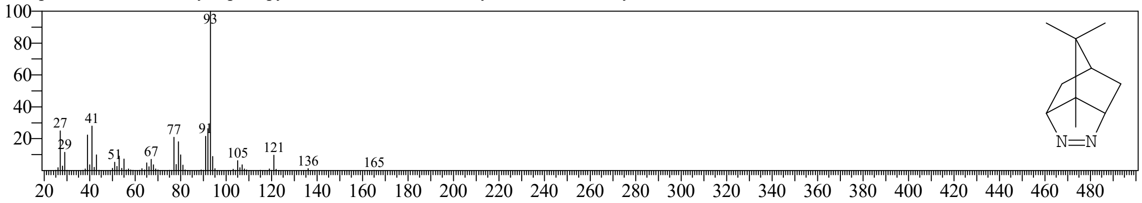

Hit#:4 Entry:10367 Library:NIST17-1.lib
SI:88 Formula:C10H16 CAS:99-84-3 MolWeight:136 RetIndex:993
CompName:Cyclohexene, 4-methylene-1-(1-methylethyl)- $$ p-Mentha-1(7),3-diene $$ .beta.-Terpinen $$ .beta.-Terpinene $$ Beta terpinene $$ 1-Isopropy

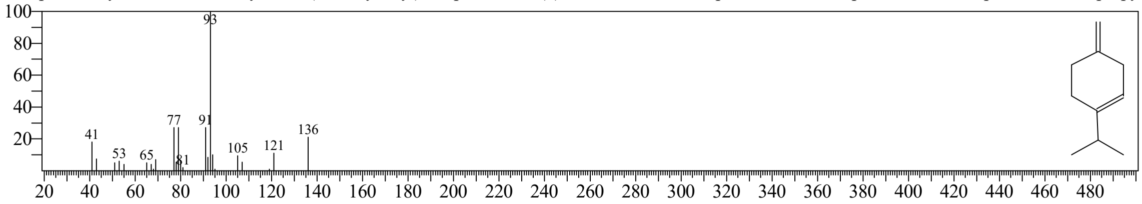

Hit#:5 Entry:10127 Library:NIST17-1.lib
SI:88 Formula:C8H12N2 CAS:109746-10-3 MolWeight:136 RetIndex:0
CompName:1,4-Methano-1H-Cyclopropa[d]pyridazine, 4,4a,5,5a-tetrahydro-6,6-dimethyl-, (1.alpha.,4.alpha.,4a.alpha.,5a.alpha.)-

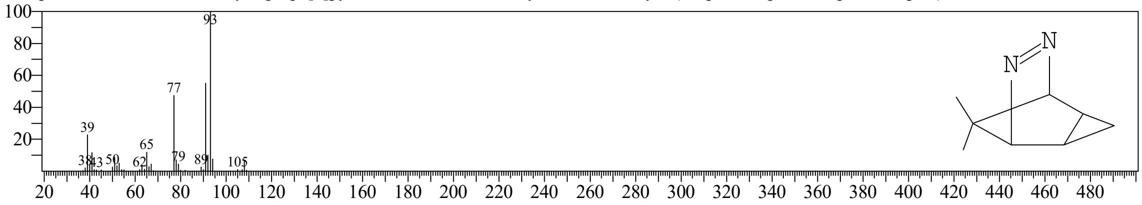

Qualitative Analysis Report

<< Target >>
Line#:8 R.Time:11.942(Scan#:1907) Retention Index:1006 MassPeaks:255
RawMode:Averaged 11.938-11.946(1906-1908) BasePeak:93.10(2083)
BG Mode:Calc. from Peak Group 1 - Event 1 Scan

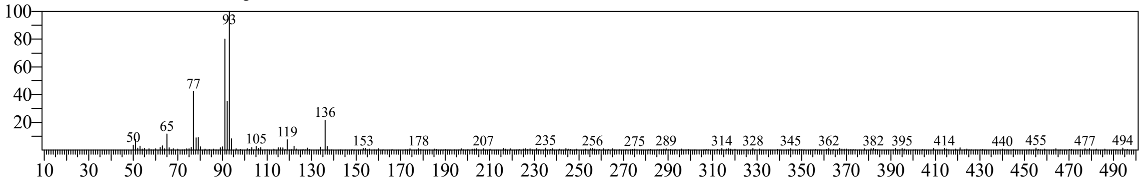

Hit#:1 Entry:648 Library:FFNSC 3.lib
SI:93 Formula:C10 H16 CAS:99-83-2 MolWeight:136 RetIndex:1007
CompName:Phellandrene <alpha->

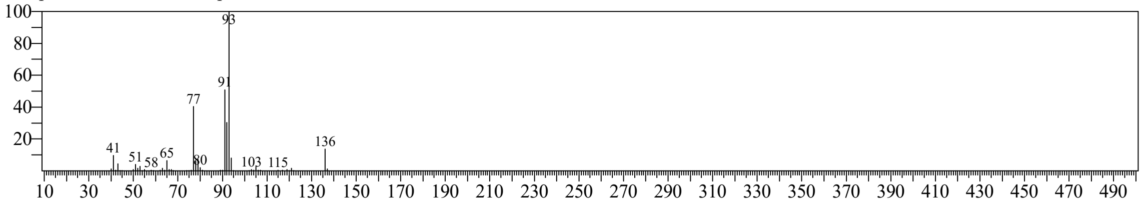

Hit#:2 Entry:10127 Library:NIST17-1.lib
SI:92 Formula:C8H12N2 CAS:109746-10-3 MolWeight:136 RetIndex:0
CompName:1,4-Methano-1H-Cyclopropa[d]pyridazine, 4,4a,5,5a-tetrahydro-6,6-dimethyl-, (1.alpha.,4.alpha.,4a.alpha.,5a.alpha.)-

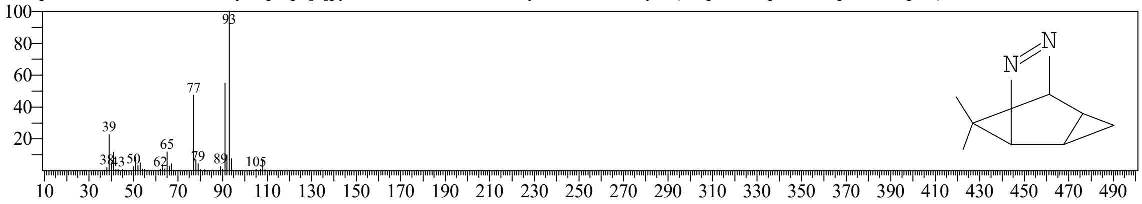

Hit#:3 Entry:10391 Library:NIST17-1.lib
SI:88 Formula:C10H16 CAS:99-85-4 MolWeight:136 RetIndex:998
CompName:.gamma.-Terpinene $$ 1,4-Cyclohexadiene, 1-methyl-4-(1-methylethyl)- $$ .gamma.-Terpinen $$ p-Mentha-1,4-diene $$ Crithmene $$ Moslene

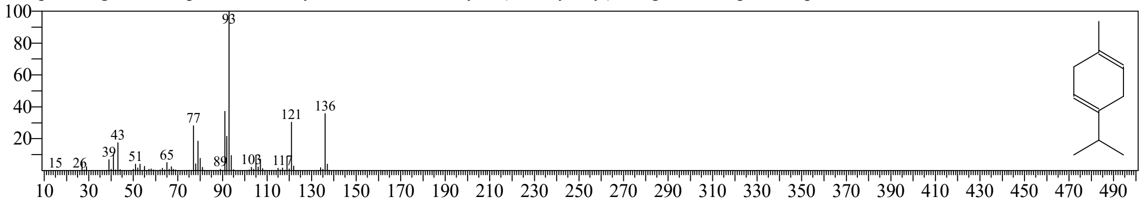

Hit#:4 Entry:186931 Library:NIST17-1.lib
SI:84 Formula:C14H16N8O3 CAS:0-00-0 MolWeight:344 RetIndex:0
CompName:1H-[1,2,3]Triazole-4-carboxylic acid, 1-(4-aminofurazan-3-yl)-5-[[(pyridin-2-ylmethyl)amino]methyl]-, ethyl ester

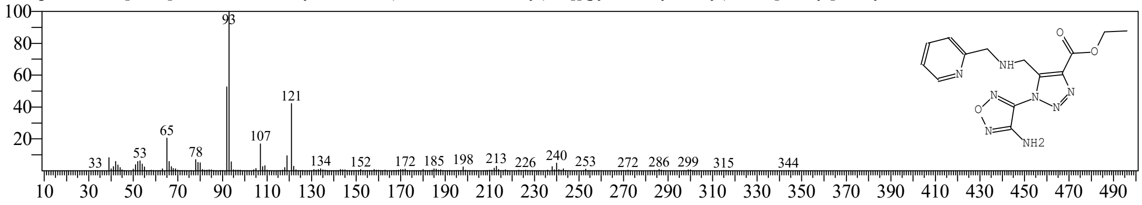

Hit#:5 Entry:1124 Library:FFNSC 3.lib
SI:84 Formula:C10 H16 CAS:13466-78-9 MolWeight:136 RetIndex:1009
CompName:Carene <delta-3->

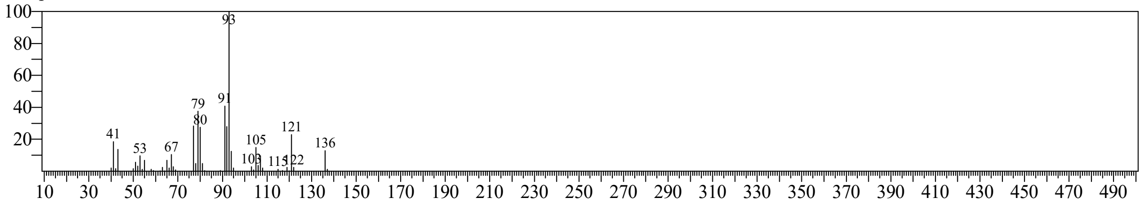

Qualitative Analysis Report

<< Target >>
Line#:9 R.Time:12.408(Scan#:2019) Retention Index:1017 MassPeaks:226
RawMode:Averaged 12.404-12.413(2018-2020) BasePeak:93.10(2731)
BG Mode:Calc. from Peak Group 1 - Event 1 Scan

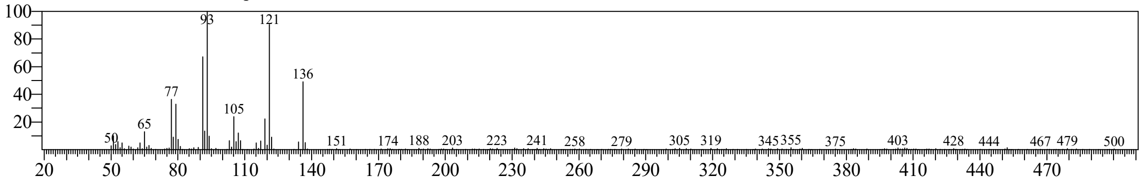

Hit#:1 Entry:277 Library:FFNSC 3.lib
SI:96 Formula:C10 H16 CAS:99-86-5 MolWeight:136 RetIndex:1018
CompName:Terpinene <alpha->

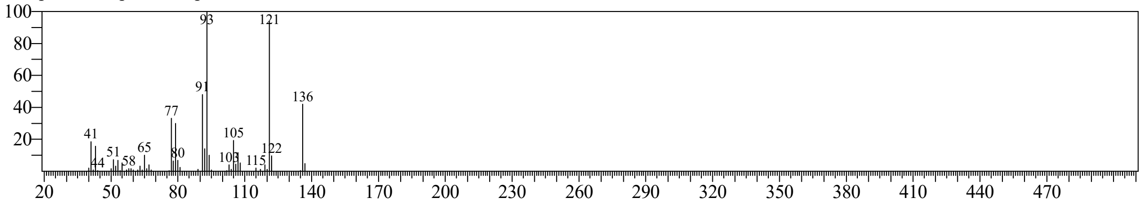

Hit#:2 Entry:10402 Library:NIST17-1.lib
SI:94 Formula:C10H16 CAS:99805-90-0 MolWeight:136 RetIndex:1023
CompName:Cyclohexene, 4-methyl-3-(1-methylethylidene)- $$ 4-Methyl-3-(1-methylethylidene)-1-cyclohexene # $$

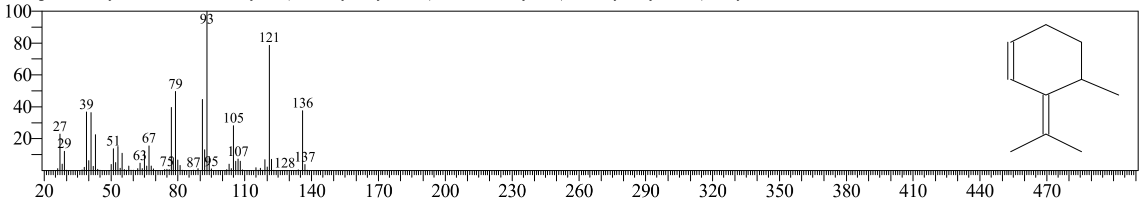

Hit#:3 Entry:10409 Library:NIST17-1.lib
SI:93 Formula:C10H16 CAS:586-63-0 MolWeight:136 RetIndex:1023
CompName:Cyclohexene, 3-methyl-6-(1-methylethylidene)- $$ p-Mentha-2,4(8)-diene $$ Isoterpinolene $$ 3-Methyl-6-(1-methylethylidene)-1-cyclohexene

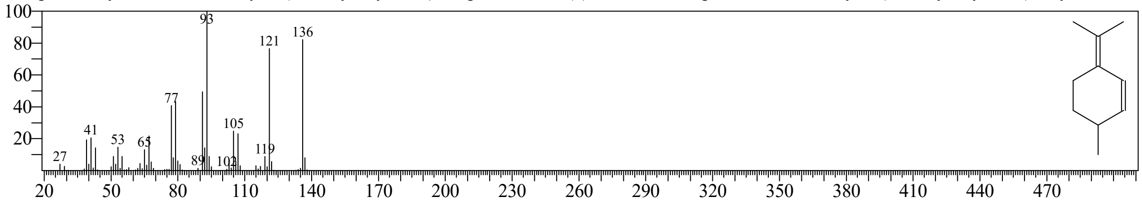

Hit#:4 Entry:10127 Library:NIST17-1.lib
SI:92 Formula:C8H12N2 CAS:109746-10-3 MolWeight:136 RetIndex:0
CompName:1,4-Methano-1H-Cyclopropa[d]pyridazine, 4,4a,5,5a-tetrahydro-6,6-dimethyl-, (1.alpha.,4.alpha.,4a.alpha.,5a.alpha.)-

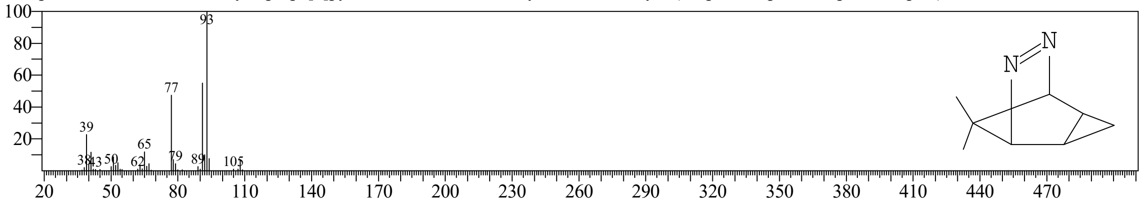

Hit#:5 Entry:2330 Library:FFNSC 3.lib
SI:88 Formula:C10 H16 CAS:499-03-6 MolWeight:136 RetIndex:1011
CompName:Sylvestrene <iso->

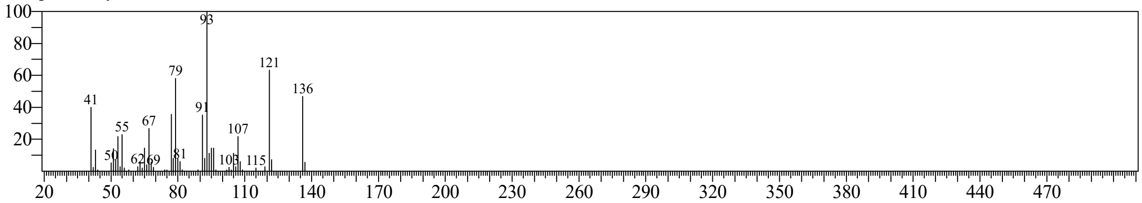

Qualitative Analysis Report

<< Target >>
Line#:10 R.Time:12.721(Scan#:2094) Retention Index:1024 MassPeaks:250
RawMode:Averaged 12.717-12.725(2093-2095) BasePeak:119.10(43426)
BG Mode:Calc. from Peak Group 1 - Event 1 Scan

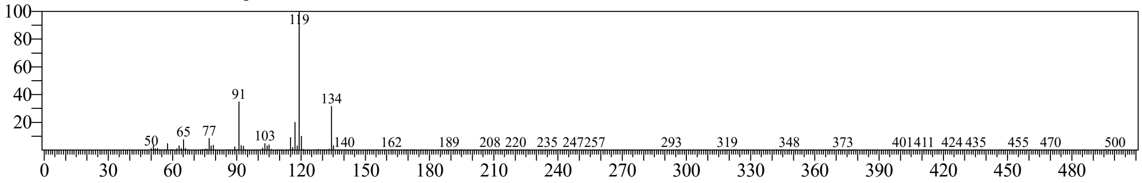

Hit#:1 Entry:317 Library:FFNSC 3.lib
SI:97 Formula:C10 H14 CAS:99-87-6 MolWeight:134 RetIndex:1025
CompName:Cymene <para->

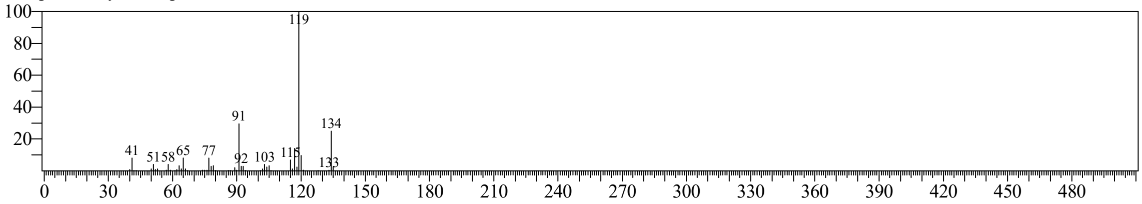

Hit#:2 Entry:5389 Library:NIST17-1.lib
SI:91 Formula:C7H5NO CAS:103-71-9 MolWeight:119 RetIndex:0
CompName:Benzene, isocyanato- $$ Isocyanic acid, phenyl ester $$ Carbanil $$ Isocyanatobenzene $$ Mondur P $$ Phenyl carbonimide $$ Phenyl isocyan

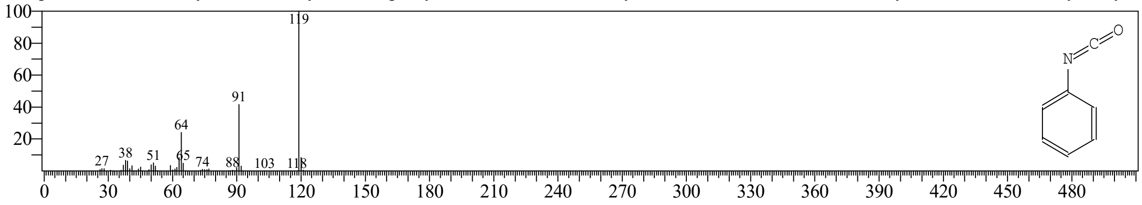

Hit#:3 Entry:1735 Library:FFNSC 3.lib
SI:91 Formula:C10 H14 CAS:527-84-4 MolWeight:134 RetIndex:1024
CompName:Cymene <ortho->

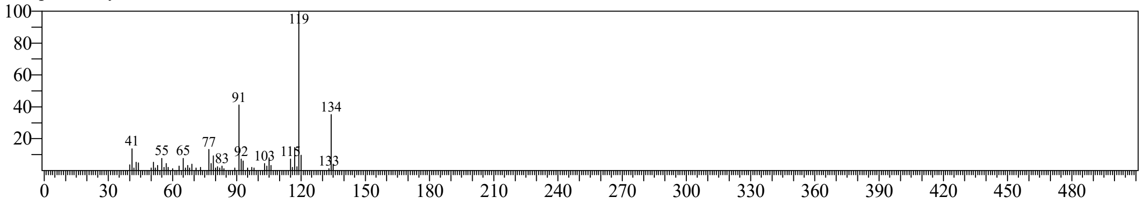

Hit#:4 Entry:32530 Library:NIST17-1.lib
SI:90 Formula:C10H11NS CAS:206559-59-3 MolWeight:177 RetIndex:0
CompName:3,4-Dimethylbenzyl isothiocyanate $$ 4-(Isothiocyanatomethyl)-1,2-dimethylbenzene # $$

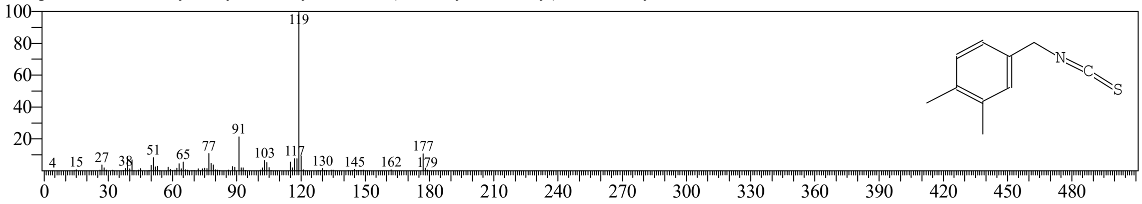

Hit#:5 Entry:242784 Library:NIST17-1.lib
SI:89 Formula:C19H20BrNO4S CAS:0-00-0 MolWeight:437 RetIndex:0
CompName:(S)-(7-Bromo-3-isopropyl-1,1-dioxido-3,4-dihydro-2H-benzo[b][1,4,5]oxathiazepin-2-yl)(m-tolyl)methanone $$ SMR001788266 $$ MLS0027

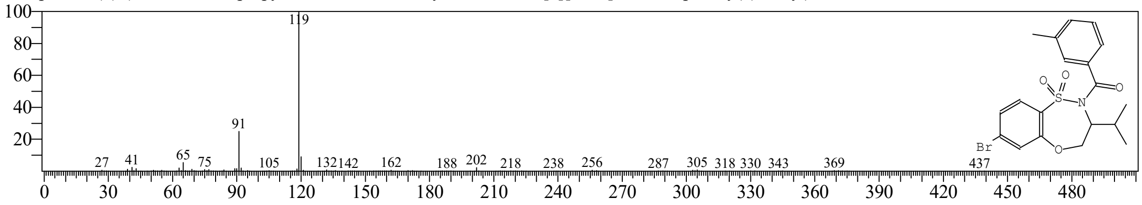

Qualitative Analysis Report

<< Target >>
Line#:11 R.Time:12.946(Scan#:2148) Retention Index:1029 MassPeaks:254
RawMode:Averaged 12.942-12.950(2147-2149) BasePeak:68.10(29082)
BG Mode:Calc. from Peak Group 1 - Event 1 Scan

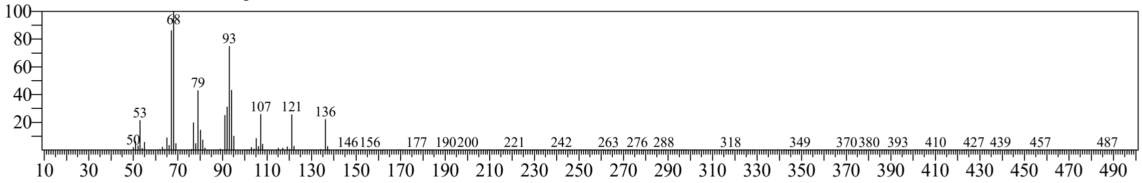

Hit#:1 Entry:980 Library:NIST17-1.lib
SI:100 Formula:F3NO CAS:13847-65-9 MolWeight:87 RetIndex:0
CompName:Trifluoroamine oxide $$ Nitrogen fluoride oxide (NF3O) $$ AMOX $$ Nitrogen Fluoride oxide (F3NO) $$ Trifluoramine oxide $$ NOF3 $$ N

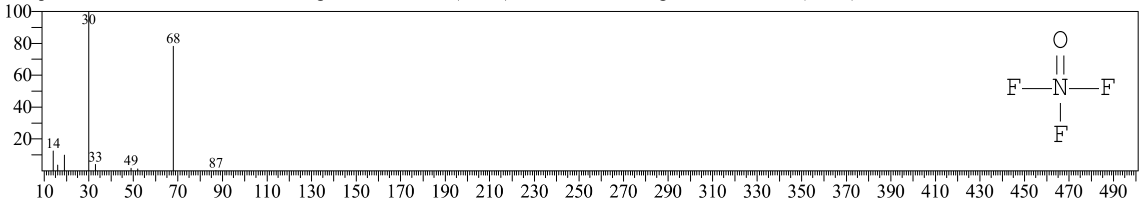

Hit#:2 Entry:443 Library:FFNSC 3.lib
SI:94 Formula:C10 H16 CAS:138-86-3 MolWeight:136 RetIndex:1030
CompName:Limonene

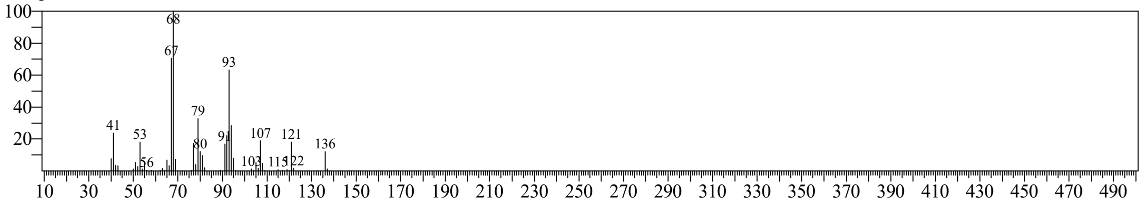

Hit#:3 Entry:206 Library:NIST17-1.lib
SI:94 Formula:C3O2 CAS:504-64-3 MolWeight:68 RetIndex:0
CompName:1,2-Propadiene-1,3-dione $$ Carbon suboxide $$ Carbon oxide (C3O2) $$ C3O2 $$ 1,3-Allenedione # $$

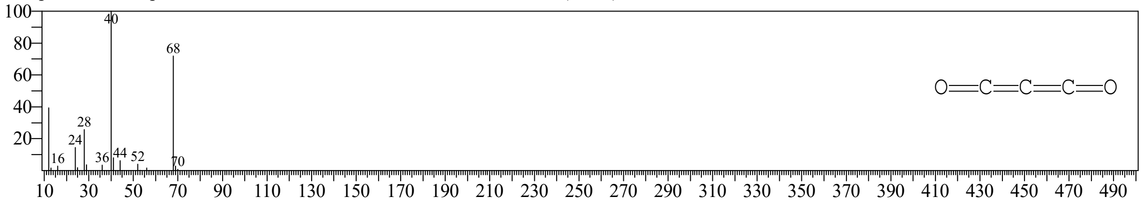

Hit#:4 Entry:8816 Library:NIST17-1.lib
SI:91 Formula:C5H8O2S CAS:1193-10-8 MolWeight:132 RetIndex:0
CompName:2,5-Dihydro-3-methyl-thiophene 1,1-dioxide $$ 3-Methylsulfolene $$ Thiophene, 2,5-dihydro-3-methyl-, 1,1-dioxide $$ 1,3-Butadiene, 2-methy

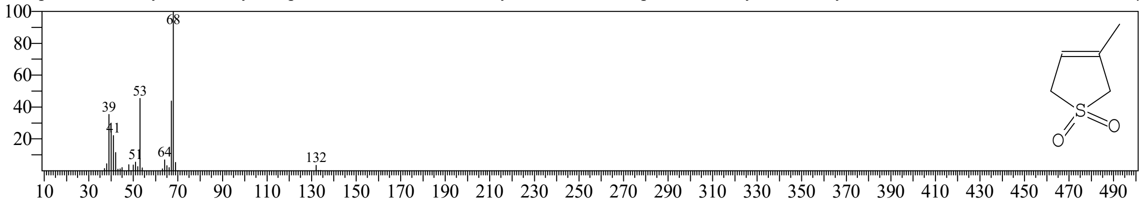

Hit#:5 Entry:2251 Library:FFNSC 3.lib
SI:90 Formula:C10 H16 CAS:1461-27-4 MolWeight:136 RetIndex:1031
CompName:Sylvestrene

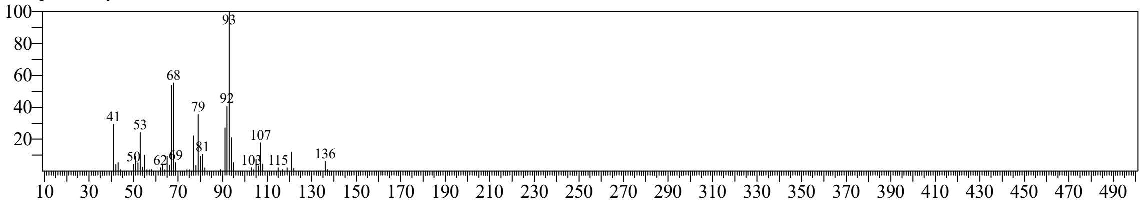

Qualitative Analysis Report

<< Target >>
Line#:12 R.Time:13.075(Scan#:2179) Retention Index:1032 MassPeaks:301
RawMode:Averaged 13.071-13.079(2178-2180) BasePeak:81.10(128288)
BG Mode:Calc. from Peak Group 1 - Event 1 Scan

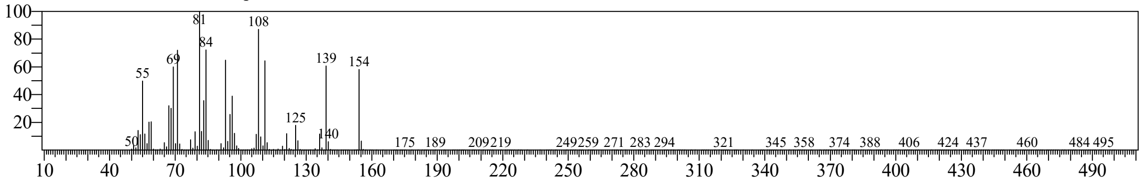

Hit#:1 Entry:621 Library:FFNSC 3.lib
SI:96 Formula:C10 H18 O CAS:470-82-6 MolWeight:154 RetIndex:1032
CompName:Eucalyptol

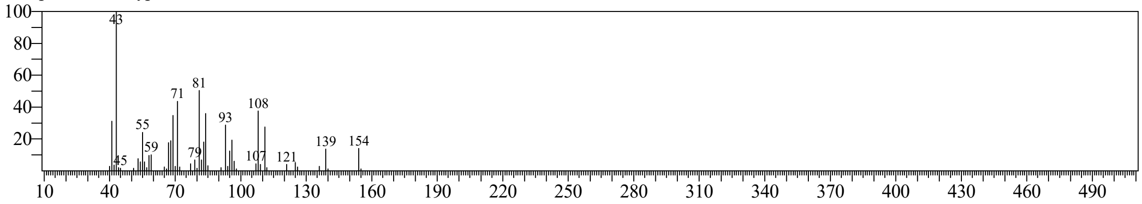

Hit#:2 Entry:6389 Library:NIST17-1.lib
SI:86 Formula:C9H16 CAS:3618-18-6 MolWeight:124 RetIndex:1026
CompName:Methylenecyclooctane $$ Cyclooctane, methylene- $$

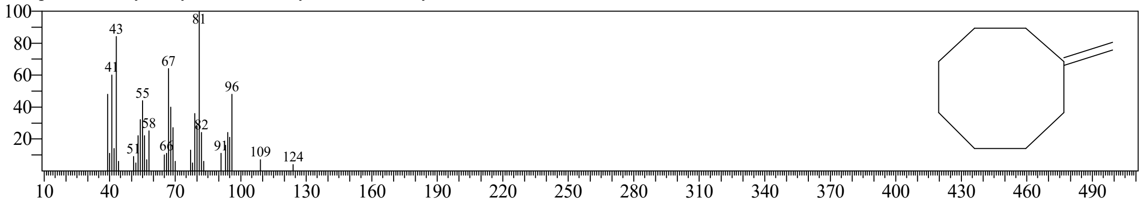

Hit#:3 Entry:158522 Library:NIST17-1.lib
SI:85 Formula:C11H5N7O5 CAS:0-00-0 MolWeight:315 RetIndex:0
CompName:12-(Furan-2-ylmethyl)-4,9,15-trioxa-3,5,8,10,12,14,16-heptaazatetracyclo[11.3.0.0(2,6).0(7,11)]hexadeca-1(16),2,5,7,10,13-hexaen-3-one

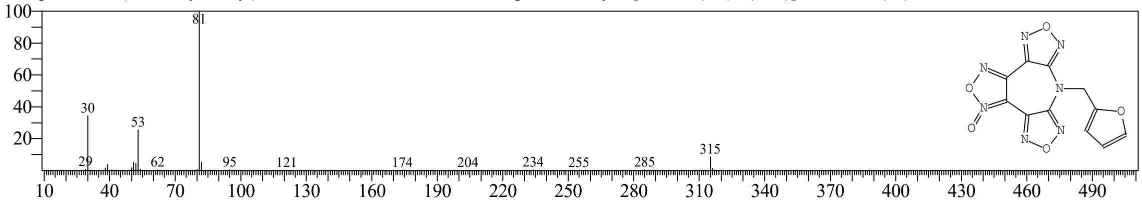

Hit#:4 Entry:11988 Library:NIST17-1.lib
SI:84 Formula:C9H16O CAS:0-00-0 MolWeight:140 RetIndex:1031
CompName:7-Methyl-8-oxabicyclo[4.3.0]nonane $$ 1-Methyloctahydro-2-benzofuran # $$

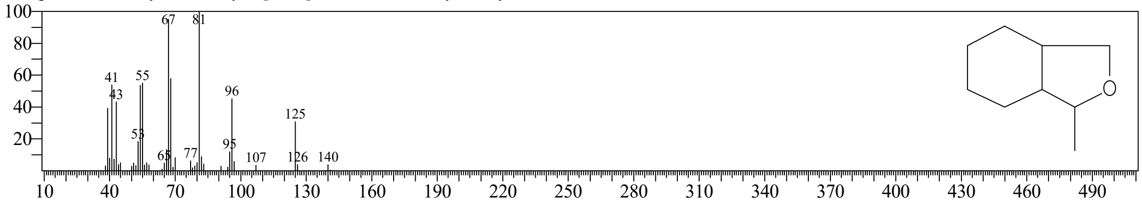

Hit#:5 Entry:1380 Library:NIST17-1.lib
SI:84 Formula:C2H6FOP CAS:753-70-8 MolWeight:96 RetIndex:0
CompName:Dimethylphosphinic fluoride

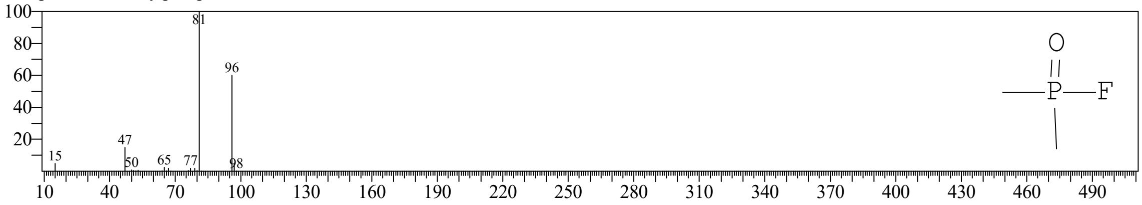

Qualitative Analysis Report

<< Target >>
Line#:13 R.Time:13.992(Scan#:2399) Retention Index:1052 MassPeaks:255
RawMode:Averaged 13.988-13.996(2398-2400) BasePeak:82.05(2234)
BG Mode:Calc. from Peak Group 1 - Event 1 Scan

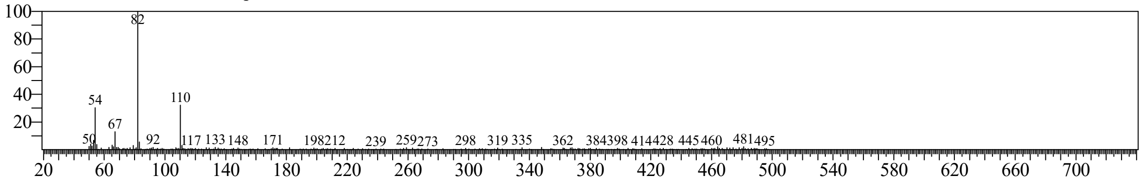

Hit#:1 Entry:406 Library:FFNSC 3.lib
SI:97 Formula:C7 H10 O CAS:1193-18-6 MolWeight:110 RetIndex:1055
CompName:Seudenone

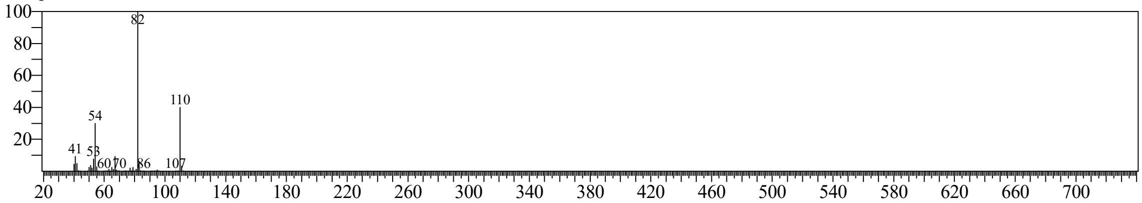

Hit#:2 Entry:3497 Library:NIST17-1.lib
SI:90 Formula:C2H6FO2P CAS:353-88-8 MolWeight:112 RetIndex:0
CompName:Methyl methylphosphonofluoridate $$ Phosphonofluoridic acid, methyl-, methyl ester $$ Methylphoshonic acid, fluoroanhydride, methyl ester $

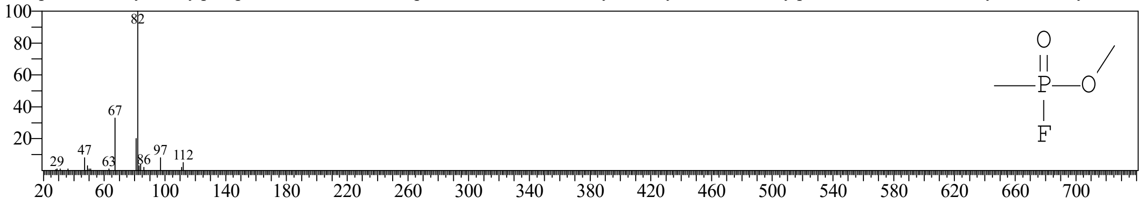

Hit#:3 Entry:55340 Library:NIST17-1.lib
SI:87 Formula:CAgF3S CAS:811-68-7 MolWeight:208 RetIndex:0
CompName:Methanethiol, trifluoro-, silver(1+) salt $$ Silver trifluoromethanethiolate $$

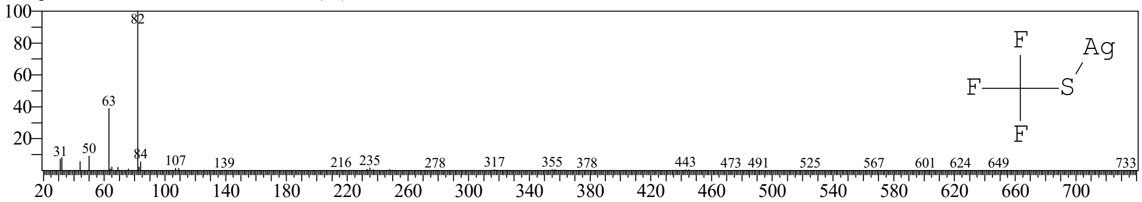

Hit#:4 Entry:3136 Library:NIST17-1.lib
SI:85 Formula:C2H7O3P CAS:6779-09-5 MolWeight:110 RetIndex:0
CompName:Ethylphosphonic acid $$ Ethanephosphonic acid $$ Phosphonic acid, ethyl- $$

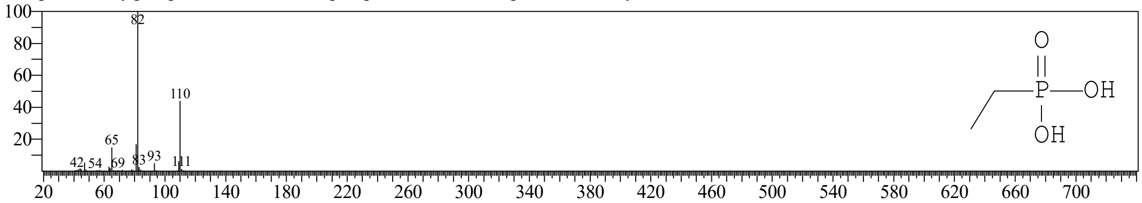

Hit#:5 Entry:71428 Library:NIST17-1.lib
SI:84 Formula:C6H10Cl2Ge CAS:5764-64-7 MolWeight:226 RetIndex:0
CompName:Germacyclopent-3-ene, 1,1-dichloro-3,4-dimethyl- $$ 1H-Germole, 1,1-dichloro-2,5-dihydro-3,4-dimethyl- $$ 1,1-Dichloro-3,4-dimethyl-2,5-di

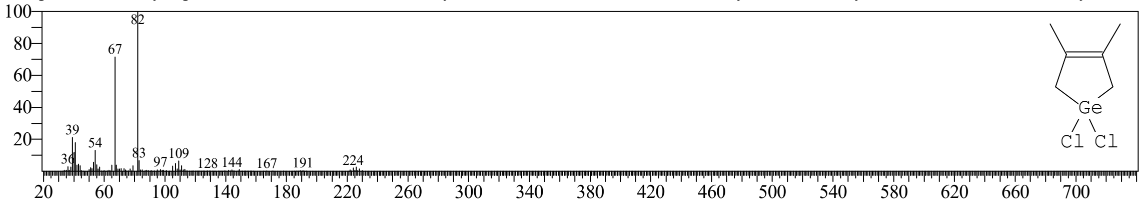

Qualitative Analysis Report

<< Target >>
Line#:14 R.Time:14.225(Scan#:2455) Retention Index:1057 MassPeaks:243
RawMode:Averaged 14.221-14.229(2454-2456) BasePeak:93.10(11594)
BG Mode:Calc. from Peak Group 1 - Event 1 Scan

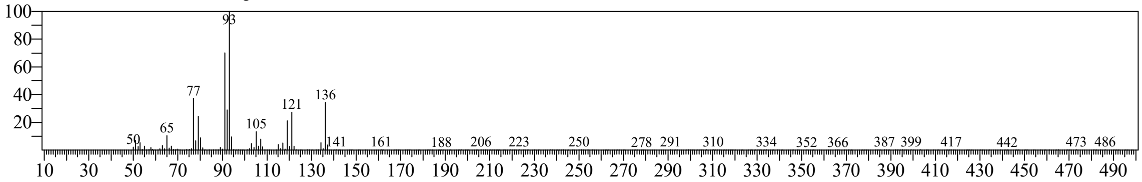

Hit#:1 Entry:818 Library:FFNSC 3.lib
SI:94 Formula:C10 H16 CAS:99-85-4 MolWeight:136 RetIndex:1058
CompName:Terpinene <gamma->

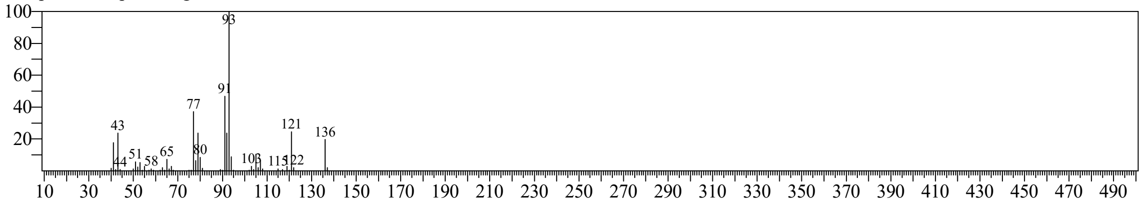

Hit#:2 Entry:10127 Library:NIST17-1.lib
SI:91 Formula:C8H12N2 CAS:109746-10-3 MolWeight:136 RetIndex:0
CompName:1,4-Methano-1H-Cyclopropa[d]pyridazine, 4,4a,5,5a-tetrahydro-6,6-dimethyl-, (1.alpha.,4.alpha.,4a.alpha.,5a.alpha.)-

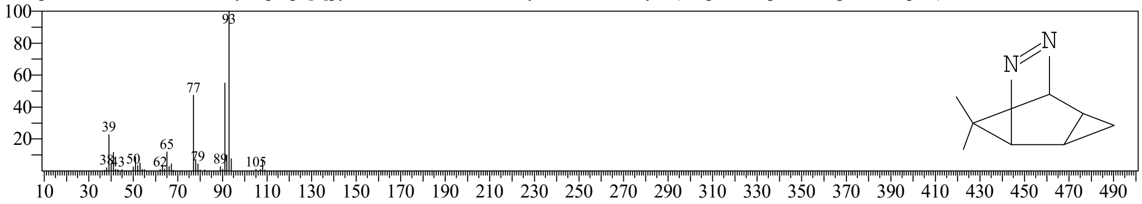

Hit#:3 Entry:10404 Library:NIST17-1.lib
SI:89 Formula:C10H16 CAS:586-62-9 MolWeight:136 RetIndex:1052
CompName:Cyclohexene, 1-methyl-4-(1-methylethylidene)- $$ p-Mentha-1,4(8)-diene $$ Terpinolene $$ Terpinolen $$ UN 2541 $$ .alpha.- Terpinolen $$

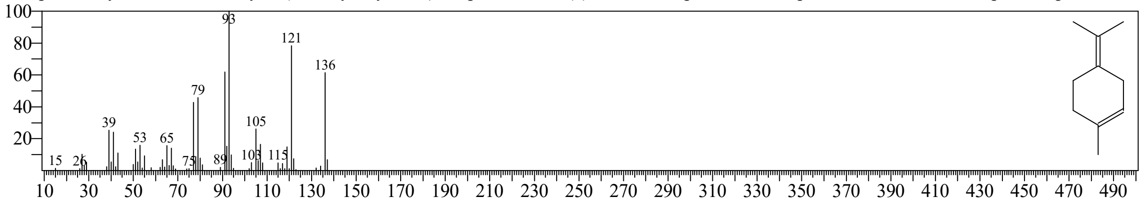

Hit#:4 Entry:10219 Library:NIST17-1.lib
SI:89 Formula:C9H12O CAS:29765-85-3 MolWeight:136 RetIndex:1060
CompName:2-Cyclopenten-1-one, 2,3,5-trimethyl-4-methylene- $$ 2,3,5-Trimethyl-4-methylene-2-cyclopenten-1-one # $$

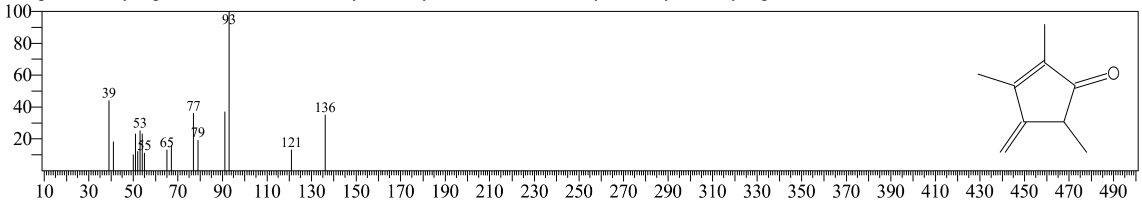

Hit#:5 Entry:186931 Library:NIST17-1.lib
SI:87 Formula:C14H16N8O3 CAS:0-00-0 MolWeight:344 RetIndex:0
CompName:1H-[1,2,3]Triazole-4-carboxylic acid, 1-(4-aminofurazan-3-yl)-5-[[(pyridin-2-ylmethyl)amino]methyl]-, ethyl ester

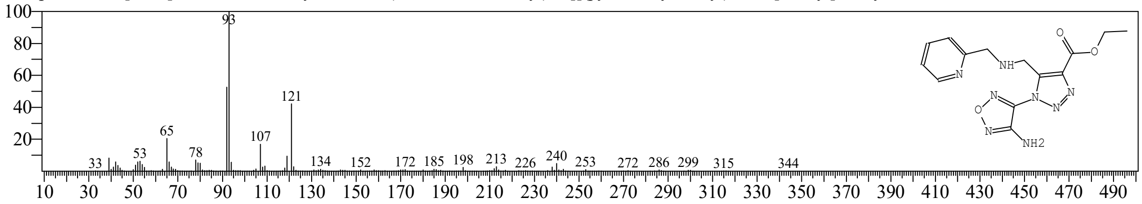

Qualitative Analysis Report

<< Target >>
Line#:15 R.Time:14.771(Scan#:2586) Retention Index:1069 MassPeaks:219
RawMode:Averaged 14.767-14.775(2585-2587) BasePeak:59.05(955)
BG Mode:Calc. from Peak Group 1 - Event 1 Scan

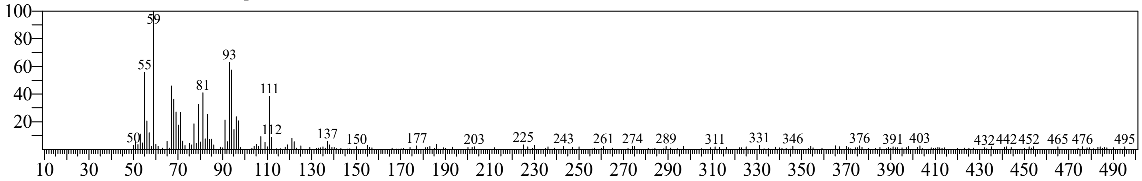

Hit#:1 Entry:27586 Library:NIST17-1.lib
SI:91 Formula:C4H12FN2PS CAS:36267-53-5 MolWeight:170 RetIndex:0
CompName:Phosphonofluoridothioic hydrazide, P-ethyl-2,2-dimethyl- $$ p-Ethyl-N',N'-dimethylphosphonohydrazidothioic fluoride # $$

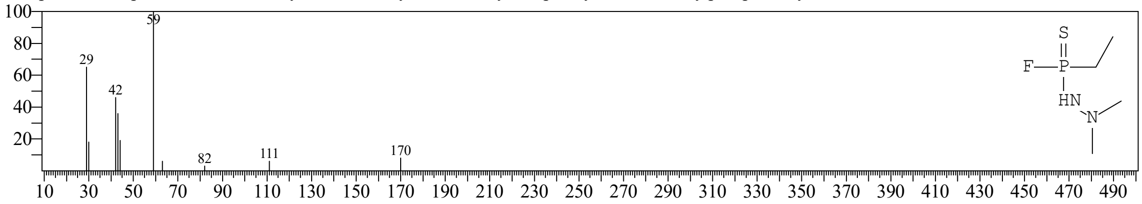

Hit#:2 Entry:988 Library:FFNSC 3.lib
SI:89 Formula:C10 H18 O2 CAS:5989-33-3 MolWeight:170 RetIndex:1069
CompName:Linalool oxide <cis->

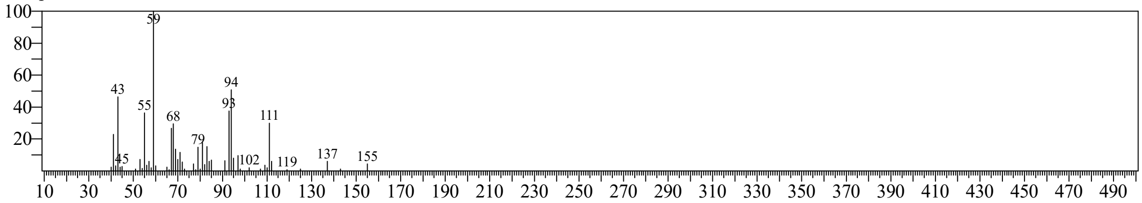

Hit#:3 Entry:953 Library:FFNSC 3.lib
SI:88 Formula:C10 H18 O CAS:15537-55-0 MolWeight:154 RetIndex:1069
CompName:Sabinene hydrate <cis->

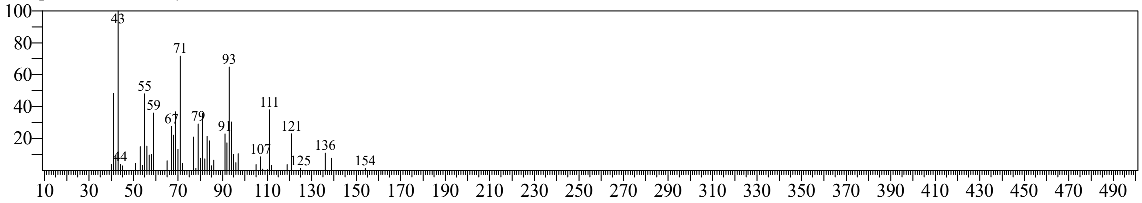

Hit#:4 Entry:19373 Library:NIST17-1.lib
SI:88 Formula:C3H10FN2PS CAS:36267-52-4 MolWeight:156 RetIndex:0
CompName:Phosphonofluoridothioic hydrazide, P,2,2-trimethyl- $$ N',N',p-Trimethylphosphonohydrazidothioic fluoride # $$

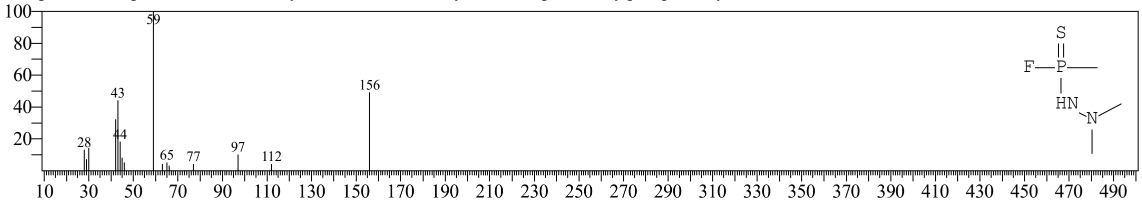

Hit#:5 Entry:27571 Library:NIST17-1.lib
SI:85 Formula:C4H4Cl2O3 CAS:0-00-0 MolWeight:170 RetIndex:1068
CompName:Methyl dichloropyruvate

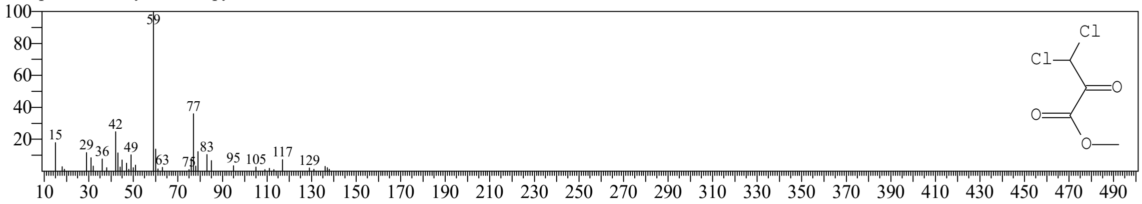

Qualitative Analysis Report

<< Target >>
Line#:16 R.Time:15.475(Scan#:2755) Retention Index:1085 MassPeaks:246
RawMode:Averaged 15.471-15.479(2754-2756) BasePeak:93.10(6785)
BG Mode:Calc. from Peak Group 1 - Event 1 Scan

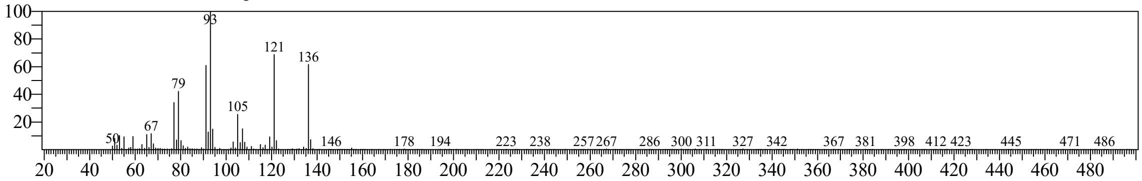

Hit#:1 Entry:603 Library:FFNSC 3.lib
SI:96 Formula:C10 H16 CAS:586-62-9 MolWeight:136 RetIndex:1086
CompName:Terpinolene

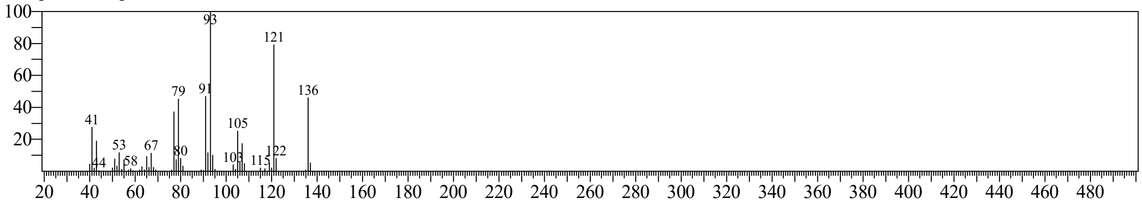

Hit#:2 Entry:10127 Library:NIST17-1.lib
SI:90 Formula:C8H12N2 CAS:109746-10-3 MolWeight:136 RetIndex:0
CompName:1,4-Methano-1H-Cyclopropa[d]pyridazine, 4,4a,5,5a-tetrahydro-6,6-dimethyl-, (1.alpha.,4.alpha.,4a.alpha.,5a.alpha.)-

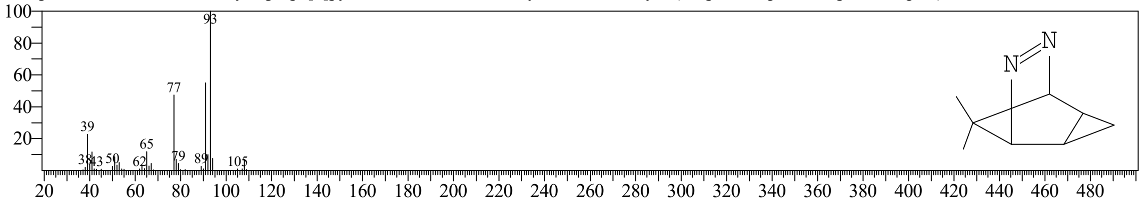

Hit#:3 Entry:24215 Library:NIST17-1.lib
SI:89 Formula:C10H16N2 CAS:0-00-0 MolWeight:164 RetIndex:0
CompName:2,3-Diazabicyclo[2.2.1]hept-2-ene, 5-ethenyl-4,7,7-trimethyl-, (1.alpha.,4.alpha.,5.beta.)- $$ 1,7,7-Trimethyl-6-vinyl-2,3-diazabicyclo[2.2.1]hep

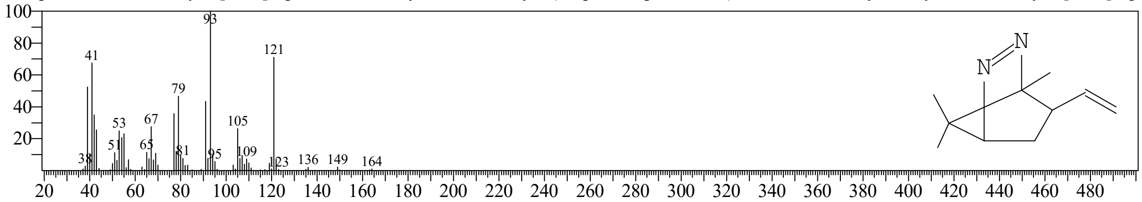

Hit#:4 Entry:10440 Library:NIST17-1.lib
SI:87 Formula:C10H16 CAS:14803-30-6 MolWeight:136 RetIndex:0
CompName:Cyclopropane, trimethyl(2-methyl-1-propenylidene)- $$ Propene, 2-methyl-1-(trimethylcyclopropylidene)- $$ 1,1,2-Trimethyl-3-(2-methyl-1-pro

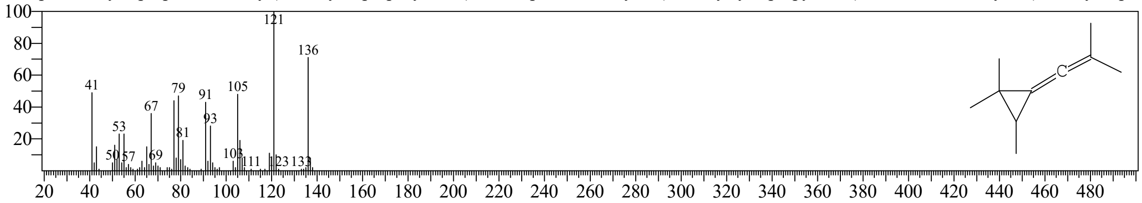

Hit#:5 Entry:10379 Library:NIST17-1.lib
SI:86 Formula:C10H16 CAS:15402-94-5 MolWeight:136 RetIndex:1095
CompName:Cycloheptene, 5-ethylidene-1-methyl- $$ (5E)-5-Ethylidene-1-methyl-1-cycloheptene # $$

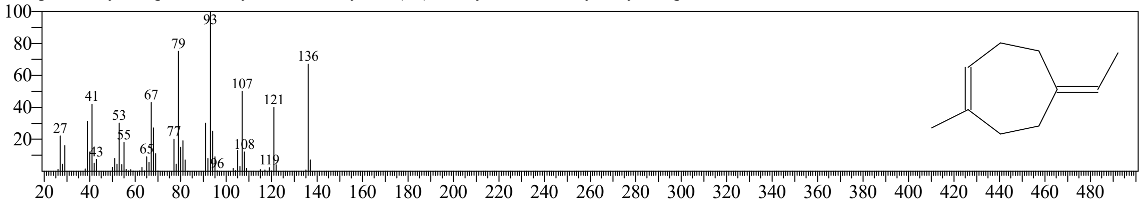

Qualitative Analysis Report

<< Target >>
Line#:17 R.Time:15.658(Scan#:2799) Retention Index:1089 MassPeaks:235
RawMode:Averaged 15.654-15.663(2798-2800) BasePeak:79.05(1517)
BG Mode:Calc. from Peak Group 1 - Event 1 Scan

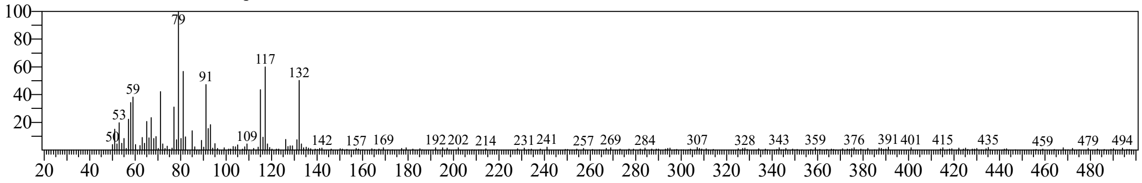

Hit#:1 Entry:2798 Library:FFNSC 3.lib
SI:90 Formula:C10 H16 O CAS:29414-55-9 MolWeight:152 RetIndex:1096
CompName:Epoxymyrcene<6,7->

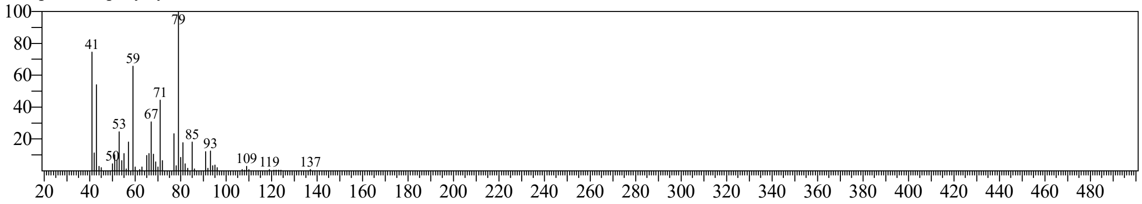

Hit#:2 Entry:5919 Library:NIST17-1.lib
SI:88 Formula:C9H14 CAS:1123-11-1 MolWeight:122 RetIndex:0
CompName:1,2-Cyclononadiene

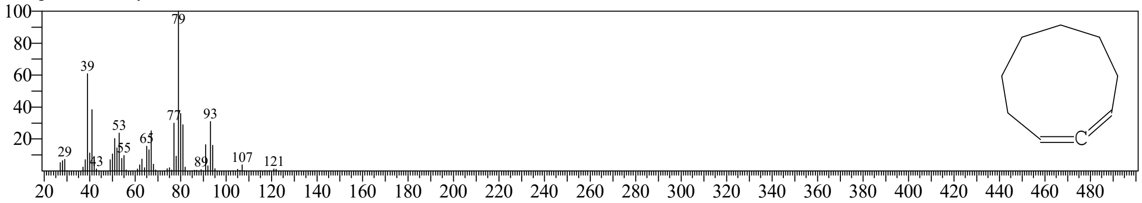

Hit#:3 Entry:10333 Library:NIST17-1.lib
SI:85 Formula:C10H16 CAS:36144-40-8 MolWeight:136 RetIndex:0
CompName:Cyclohexane, 1-butenylidene- $$ 1-Butenylidenecyclohexane # $$

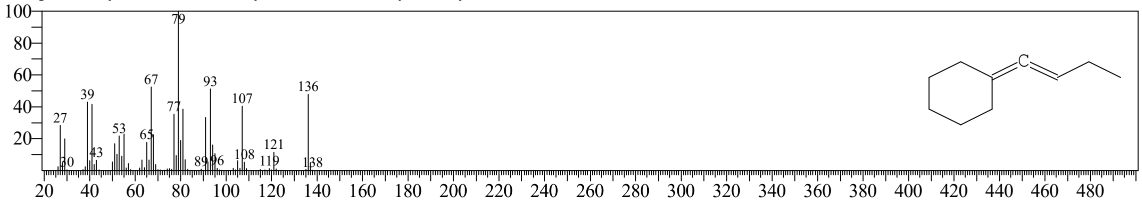

Hit#:4 Entry:242361 Library:NIST17-1.lib
SI:84 Formula:C12H18Br2Ni2 CAS:32877-98-8 MolWeight:436 RetIndex:0
CompName:Nickel, di-.mu.-bromobis[(1,2,3-.eta.)-2-cyclohexen-1-yl]di-

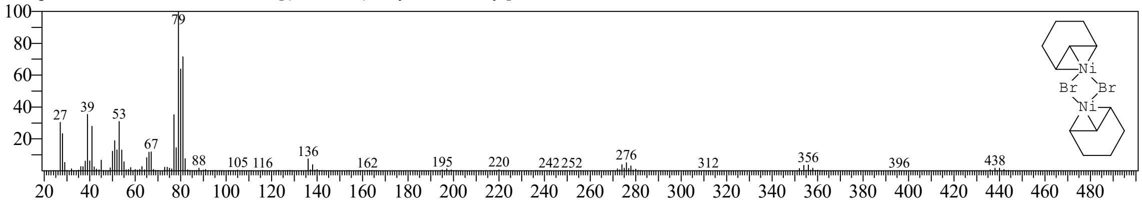

Hit#:5 Entry:5614 Library:NIST17-1.lib
SI:84 Formula:CH3N3O2S CAS:1516-70-7 MolWeight:121 RetIndex:0
CompName:Methanesulfonyl azide $$ Mesyl azide $$

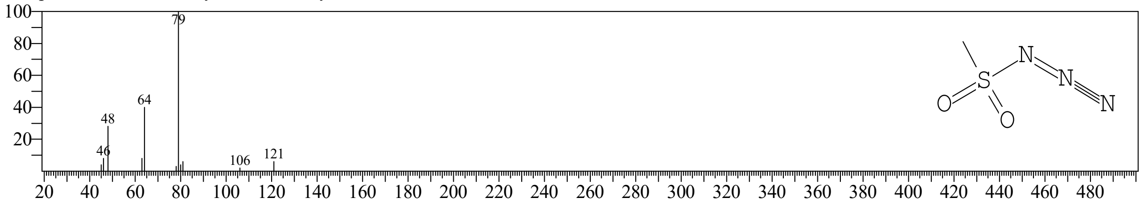

Qualitative Analysis Report

<< Target >>
Line#:18 R.Time:16.071(Scan#:2898) Retention Index:1098 MassPeaks:255
RawMode:Averaged 16.067-16.075(2897-2899) BasePeak:93.05(25787)
BG Mode:Calc. from Peak Group 1 - Event 1 Scan

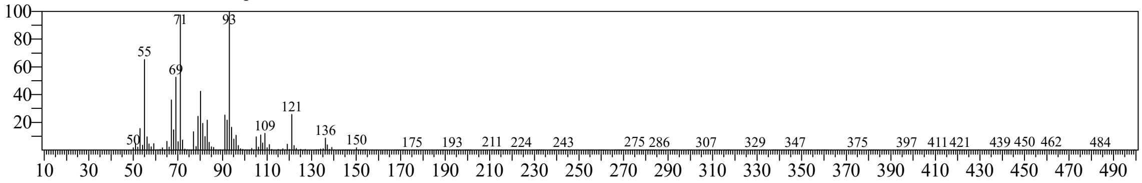

Hit#:1 Entry:3376 Library:FFNSC 3.lib
SI:94 Formula:C17 H23 N O2 CAS:7149-26-0 MolWeight:273 RetIndex:1104
CompName:Linalyl anthranilate

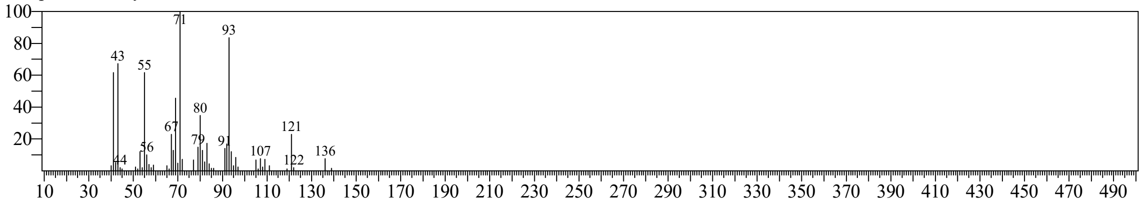

Hit#:2 Entry:365 Library:FFNSC 3.lib
SI:93 Formula:C10 H18 O CAS:78-70-6 MolWeight:154 RetIndex:1101
CompName:Linalool

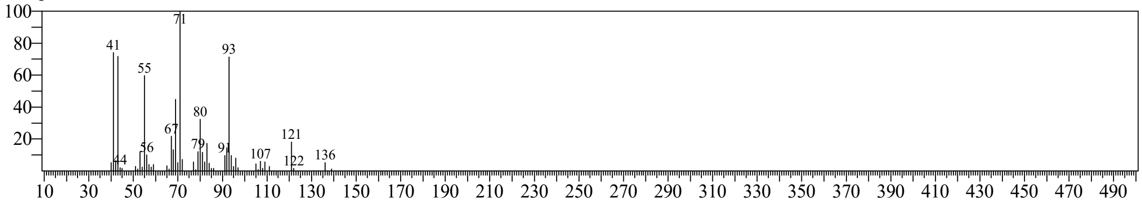

Hit#:3 Entry:93586 Library:NIST17-1.lib
SI:91 Formula:C12H17F3O2 CAS:0-00-0 MolWeight:250 RetIndex:1099
CompName:(-)-Isopinocampheol, trifluoroacetate

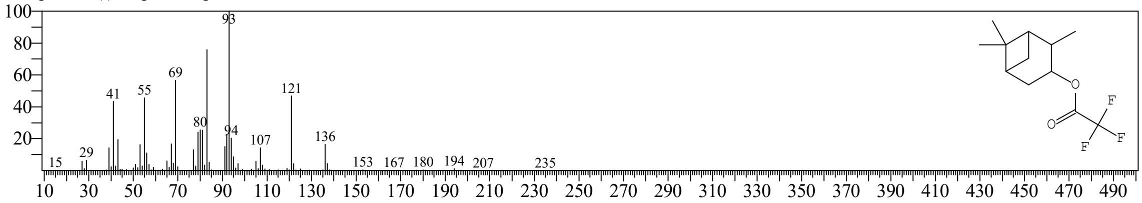

Hit#:4 Entry:954 Library:FFNSC 3.lib
SI:88 Formula:C10 H18 O CAS:17699-16-0 MolWeight:154 RetIndex:1099
CompName:Sabinene hydrate <trans->

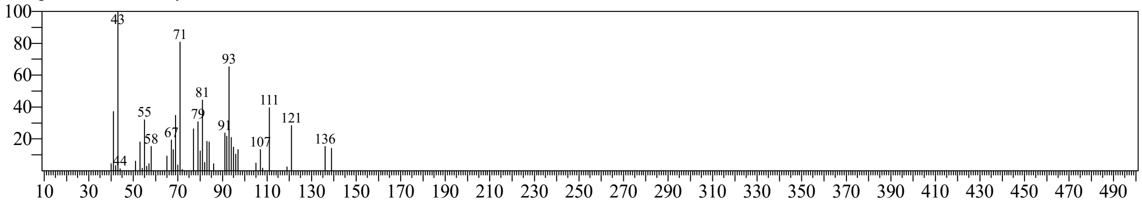

Hit#:5 Entry:2939 Library:NIST17-1.lib
SI:86 Formula:C6H8Si CAS:63878-65-9 MolWeight:108 RetIndex:0
CompName:Silabenzene,1-methyl- $$ 1-Methyl-1-silabenzene $$ 1-Methylsiline # $$

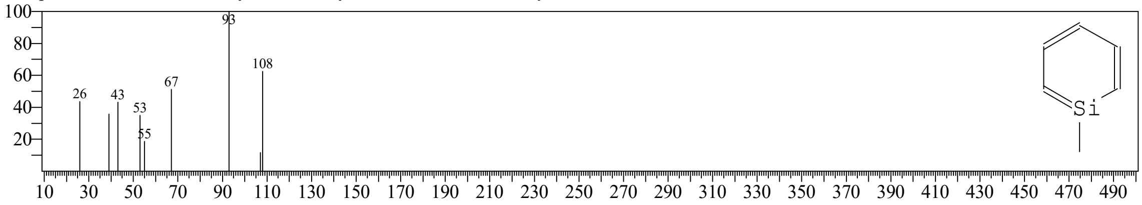

Qualitative Analysis Report

<< Target >>
Line#:19 R.Time:17.854(Scan#:3326) Retention Index:1137 MassPeaks:260
RawMode:Averaged 17.850-17.858(3325-3327) BasePeak:82.05(6274)
BG Mode:Calc. from Peak Group 1 - Event 1 Scan

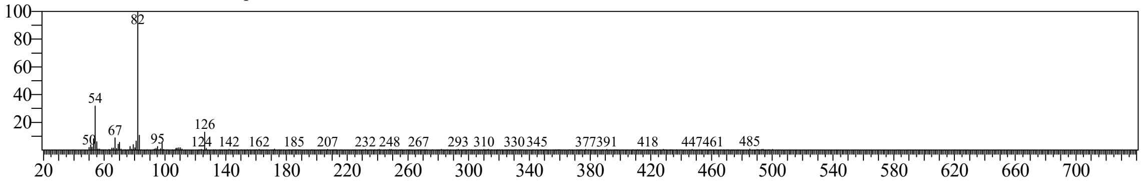

Hit#:1 Entry:3497 Library:NIST17-1.lib
SI:88 Formula:C2H6FO2P CAS:353-88-8 MolWeight:112 RetIndex:0
CompName:Methyl methylphosphonofluoridate $$ Phosphonofluoridic acid, methyl-, methyl ester $$ Methylphoshonic acid, fluoroanhydride, methyl ester $

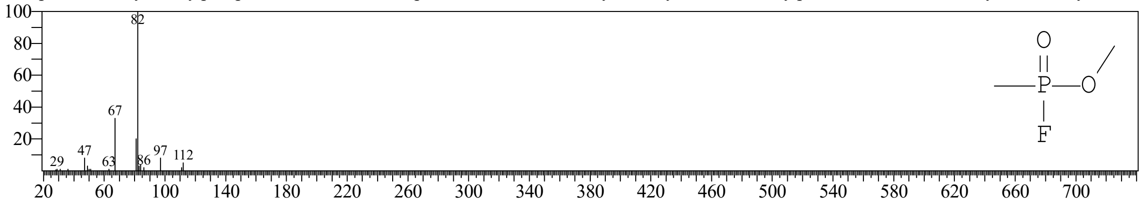

Hit#:2 Entry:55340 Library:NIST17-1.lib
SI:86 Formula:CAgF3S CAS:811-68-7 MolWeight:208 RetIndex:0
CompName:Methanethiol, trifluoro-, silver(1+) salt $$ Silver trifluoromethanethiolate $$

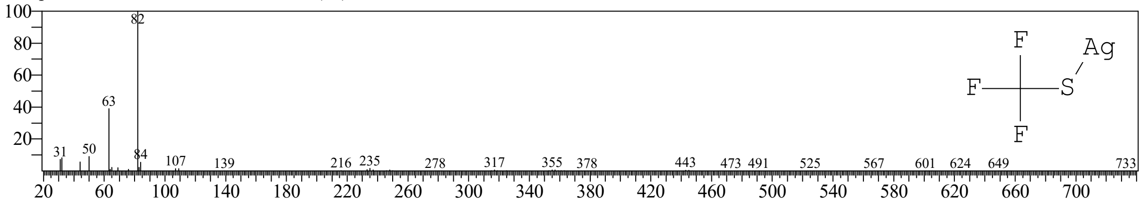

Hit#:3 Entry:173912 Library:NIST17-1.lib
SI:85 Formula:C18H33B3O3 CAS:28289-86-3 MolWeight:330 RetIndex:0
CompName:Boroxin, tricyclohexyl- $$ 2,4,6-Tricyclohexylboroxin # $$

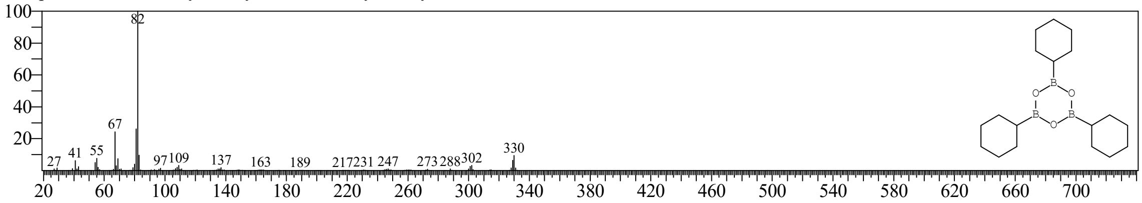

Hit#:4 Entry:18025 Library:NIST17-1.lib
SI:84 Formula:C9H20BN CAS:30430-68-3 MolWeight:153 RetIndex:0
CompName:2-Butyl-1-ethyl-1,2-azaborolidine

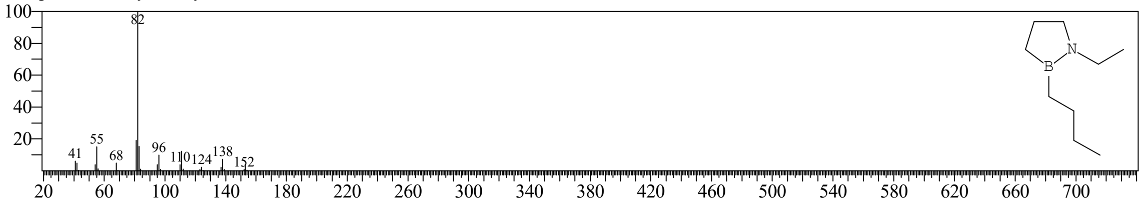

Hit#:5 Entry:71428 Library:NIST17-1.lib
SI:84 Formula:C6H10Cl2Ge CAS:5764-64-7 MolWeight:226 RetIndex:0
CompName:Germacyclopent-3-ene, 1,1-dichloro-3,4-dimethyl- $$ 1H-Germole, 1,1-dichloro-2,5-dihydro-3,4-dimethyl- $$ 1,1-Dichloro-3,4-dimethyl-2,5-d

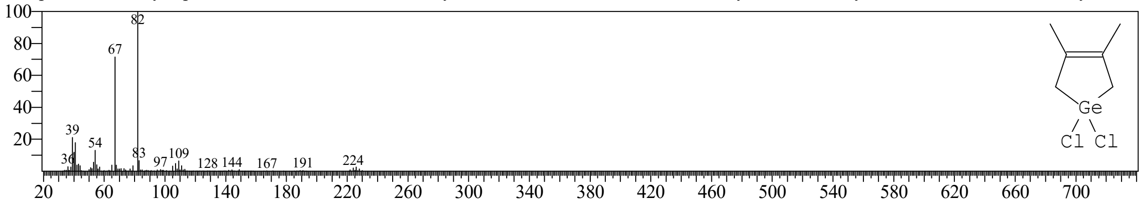

Qualitative Analysis Report

<< Target >>
Line#:20 R.Time:18.079(Scan#:3380) Retention Index:1142 MassPeaks:258
RawMode:Averaged 18.075-18.083(3379-3381) BasePeak:69.10(1330)
BG Mode:Calc. from Peak Group 1 - Event 1 Scan

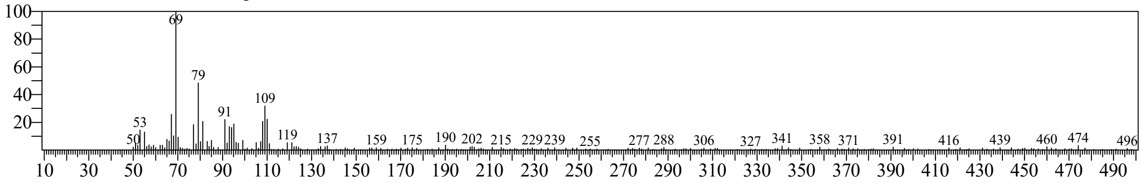

Hit#:1 Entry:228 Library:NIST17-1.lib
SI:99 Formula:C2H3N3 CAS:288-36-8 MolWeight:69 RetIndex:0
CompName:1H-1,2,3-Triazole $$ V-triazole $$ Osotriazole $$ Pyrrodiazole $$ Triazacyclopentadiene $$ 1,2,3-Triazole $$

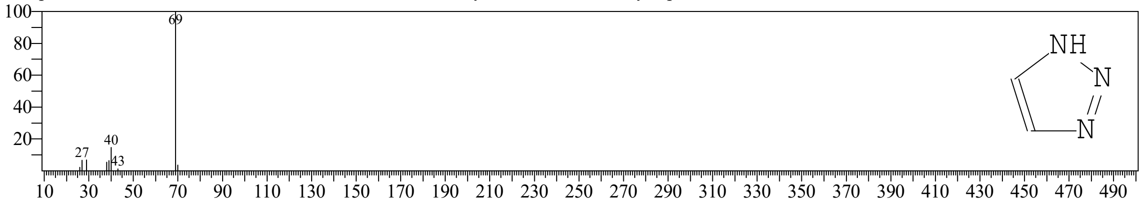

Hit#:2 Entry:10642 Library:NIST17-1.lib
SI:95 Formula:CF5P CAS:1112-04-5 MolWeight:138 RetIndex:0
CompName:Trifluoromethyldifluorophosphine $$ Trifluoromethylphosphonous difluoride # $$

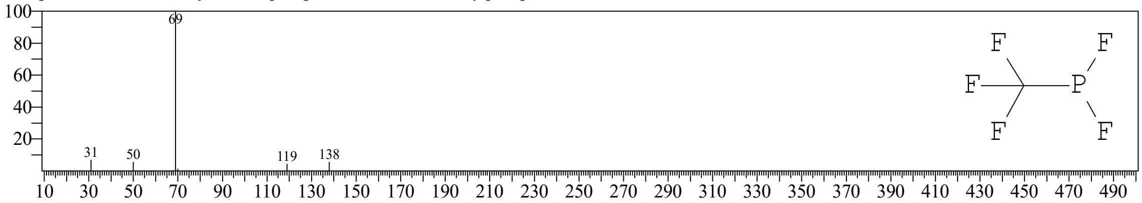

Hit#:3 Entry:981 Library:NIST17-1.lib
SI:95 Formula:CF4 CAS:75-73-0 MolWeight:88 RetIndex:0
CompName:Tetrafluoromethane $$ Carbon tetrafluoride $$ Methane, tetrafluoro- $$ Arcton 0 $$ Carbon fluoride (CF4) $$ Freon 14 $$ FC 14 $$ Perfluoro

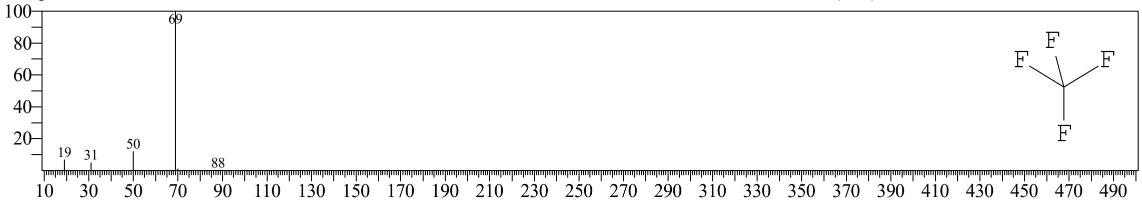

Hit#:4 Entry:10455 Library:NIST17-1.lib
SI:94 Formula:CClF4N CAS:13880-72-3 MolWeight:137 RetIndex:0
CompName:N-Chloro-N-fluoro-trifluoromethylamine $$ N-Chloro-N-tetrafluoromethanamine # $$

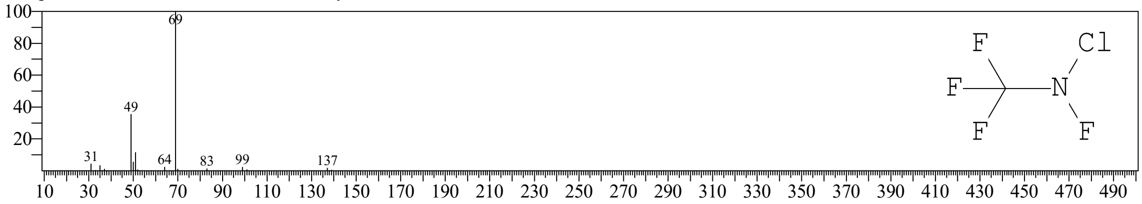

Hit#:5 Entry:9867 Library:NIST17-1.lib
SI:93 Formula:CF4OS CAS:812-12-4 MolWeight:136 RetIndex:0
CompName:Methanesulfinyl fluoride, trifluoro- $$ Trifluoromethanesulfinyl fluoride $$ Trifluoromethylsulfinyl fluoride $$

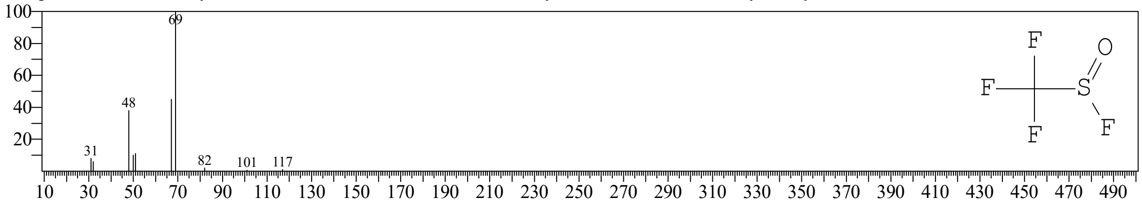

Qualitative Analysis Report

<< Target >>
Line#:21 R.Time:18.271(Scan#:3426) Retention Index:1146 MassPeaks:274
RawMode:Averaged 18.267-18.275(3425-3427) BasePeak:95.10(21165)
BG Mode:Calc. from Peak Group 1 - Event 1 Scan

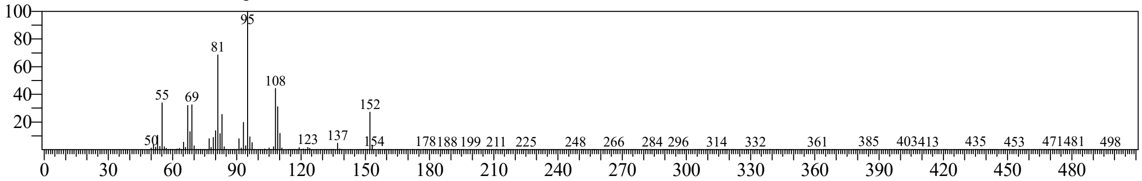

Hit#:1 Entry:298 Library:FFNSC 3.lib
SI:97 Formula:C10 H16 O CAS:76-22-2 MolWeight:152 RetIndex:1149
CompName:Camphor

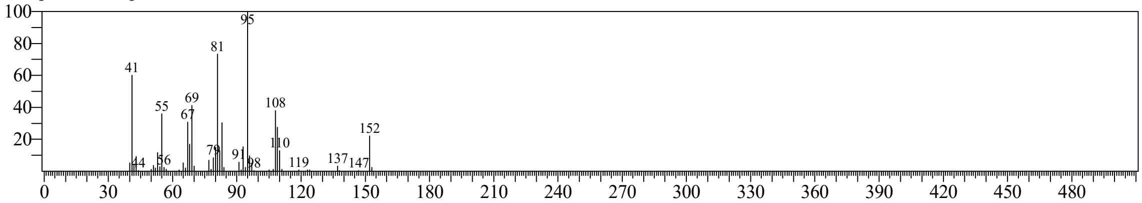

Hit#:2 Entry:34970 Library:NIST17-1.lib
SI:87 Formula:C12H20O CAS:19576-21-7 MolWeight:180 RetIndex:1155
CompName:1-Propanone, 1-[2-(1,1-dimethylethyl)-2-cyclopropen-1-yl]-2,2-dimethyl- $$ 1-Propanone, 1-(2-tert-butyl-2-cyclopropen-1-yl)-2,2-dimethyl- $$

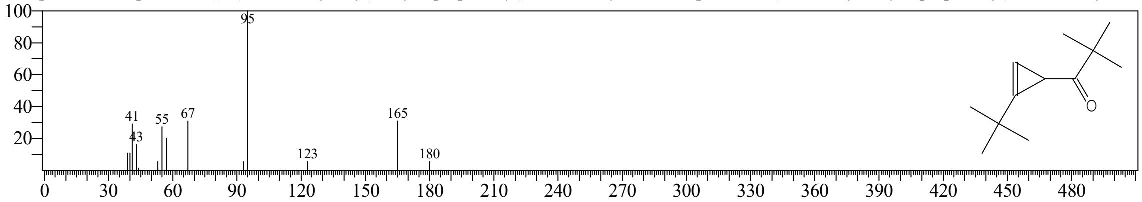

Hit#:3 Entry:30769 Library:NIST17-1.lib
SI:86 Formula:C10H19Cl CAS:80483-50-7 MolWeight:174 RetIndex:1143
CompName:2-Chloro-1-isopropyl-4-methylcyclohexane

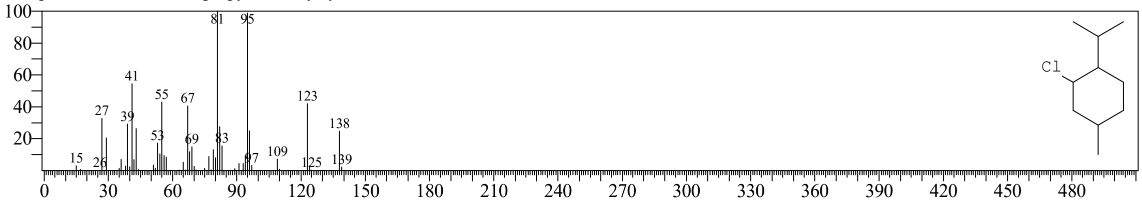

Hit#:4 Entry:17944 Library:NIST17-1.lib
SI:85 Formula:C8H11NS CAS:18530-33-1 MolWeight:153 RetIndex:0
CompName:Bicyclo[2.2.1]heptane, 2-isothiocyanato-, exo- $$ 2-Isothiocyanatobicyclo[2.2.1]heptane # $$

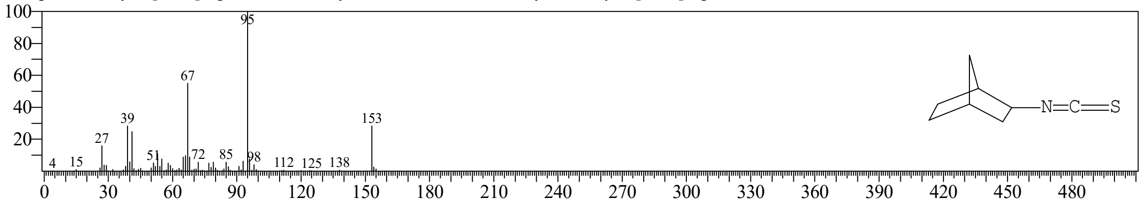

Hit#:5 Entry:11059 Library:NIST17-1.lib
SI:85 Formula:C9H14O CAS:697-37-0 MolWeight:138 RetIndex:1147
CompName:1-(1-Propynyl)cyclohexanol

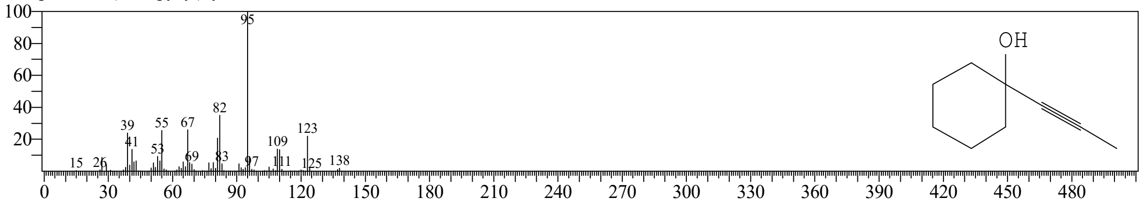

Qualitative Analysis Report

<< Target >>
Line#:22 R.Time:18.483(Scan#:3477) Retention Index:1150 MassPeaks:272
RawMode:Averaged 18.479-18.488(3476-3478) BasePeak:69.10(694)
BG Mode:Calc. from Peak Group 1 - Event 1 Scan

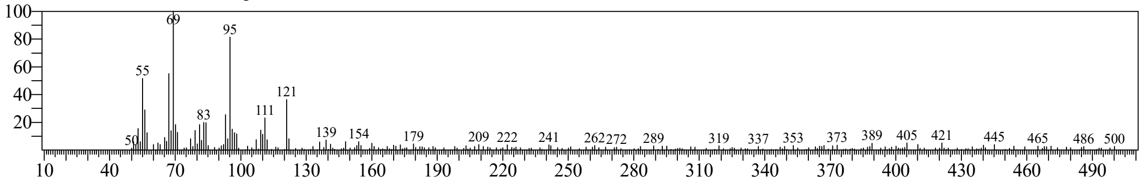

Hit#:1 Entry:228 Library:NIST17-1.lib
SI:97 Formula:C2H3N3 CAS:288-36-8 MolWeight:69 RetIndex:0
CompName:1H-1,2,3-Triazole $$ V-triazole $$ Osotriazole $$ Pyrrodiazole $$ Triazacyclopentadiene $$ 1,2,3-Triazole $$

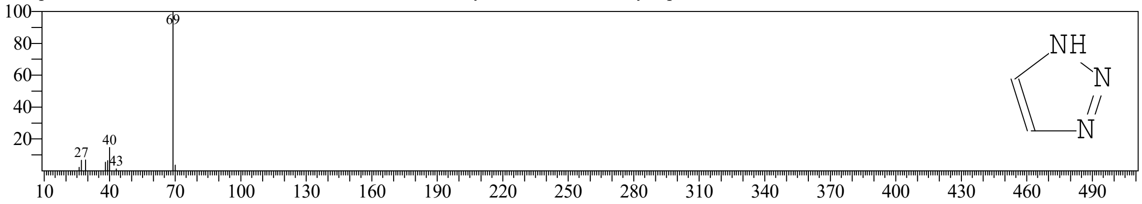

Hit#:2 Entry:9867 Library:NIST17-1.lib
SI:95 Formula:CF4OS CAS:812-12-4 MolWeight:136 RetIndex:0
CompName:Methanesulfinyl fluoride, trifluoro- $$ Trifluoromethanesulfinyl fluoride $$ Trifluoromethylsulfinyl fluoride $$

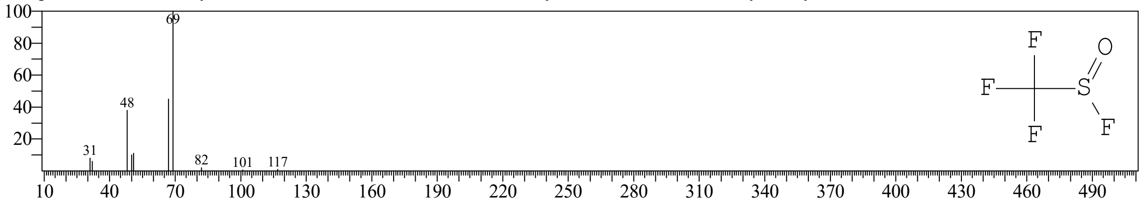

Hit#:3 Entry:1001 Library:FFNSC 3.lib
SI:94 Formula:C10 H18 O CAS:106-23-0 MolWeight:154 RetIndex:1152
CompName:Citronellal

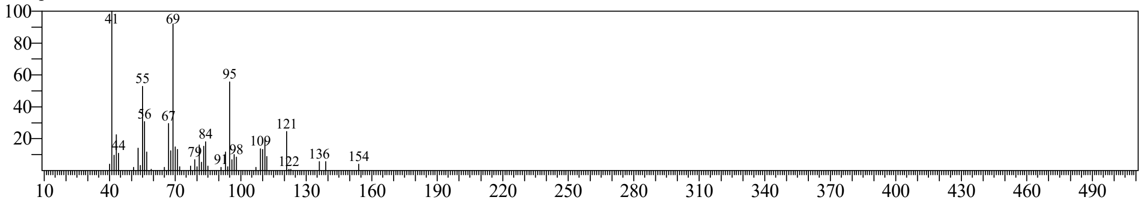

Hit#:4 Entry:73172 Library:NIST17-1.lib
SI:93 Formula:C2H6F4N2P2S CAS:27352-02-9 MolWeight:228 RetIndex:0
CompName:Phosphoramidothioic difluoride, [(dimethylamino)difluorophosphoranylidene]- $$ (Dimethylamino)(difluoro)phosphoranylidenephosphoramido

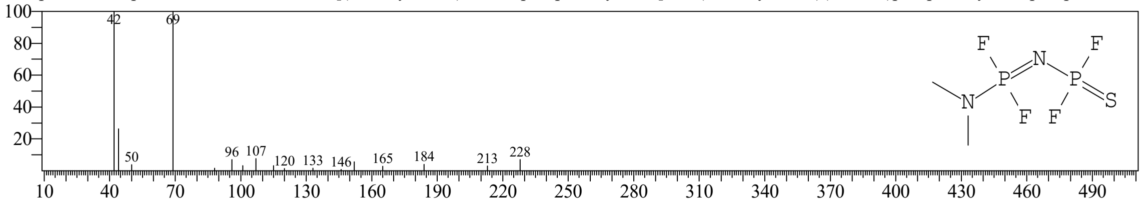

Hit#:5 Entry:981 Library:NIST17-1.lib
SI:93 Formula:CF4 CAS:75-73-0 MolWeight:88 RetIndex:0
CompName:Tetrafluoromethane $$ Carbon tetrafluoride $$ Methane, tetrafluoro- $$ Arcton 0 $$ Carbon fluoride (CF4) $$ Freon 14 $$ FC 14 $$ Perfluoro

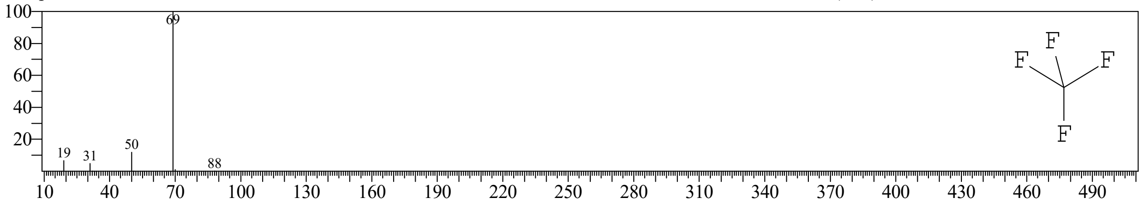

Qualitative Analysis Report

<< Target >>
Line#:23   R.Time:18.900(Scan#:3577) Retention Index:1159 MassPeaks:262
RawMode:Averaged 18.896-18.904(3576-3578) BasePeak:83.10(1745)
BG Mode:Calc. from Peak Group 1 - Event 1 Scan

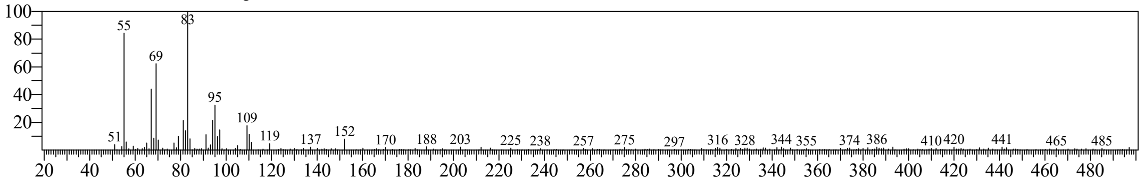

Hit#:1 Entry:3389 Library:NIST17-1.lib
SI:92 Formula:C3HN3S CAS:57352-02-0 MolWeight:111 RetIndex:0
CompName:5-Cyano-1,2,3-thiadiazole $$ 1,2,3-Thiadiazole-5-carbonitrile # $$

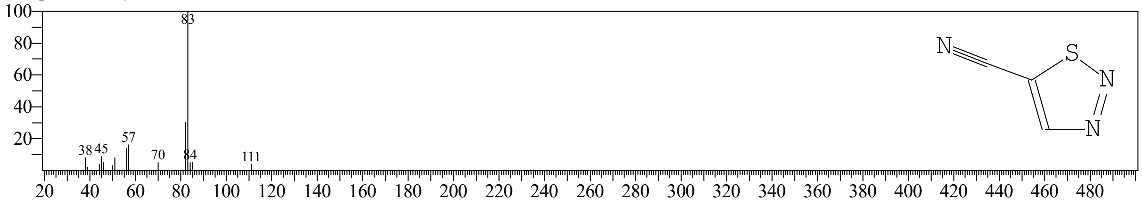

Hit#:2 Entry:1502 Library:FFNSC 3.lib
SI:91 Formula:C10 H16 O CAS:547-60-4 MolWeight:152 RetIndex:1160
CompName:Pinocamphone <trans->

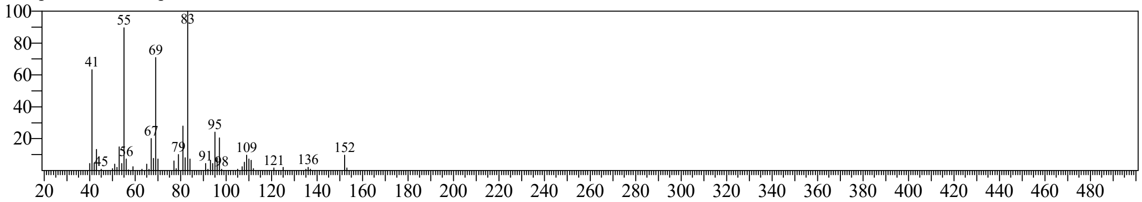

Hit#:3 Entry:26728 Library:NIST17-1.lib
SI:89 Formula:C10H16O2 CAS:90358-41-1 MolWeight:168 RetIndex:1153
CompName:(Z)-(E)-2-Methylbut-2-en-1-yl 2-methylbut-2-enoate $$ 2-Butenoic acid, 2-methyl-, (2E)-2-methyl-2-buten-1-yl ester, (2Z)- $$ 2-Butenoic acid,

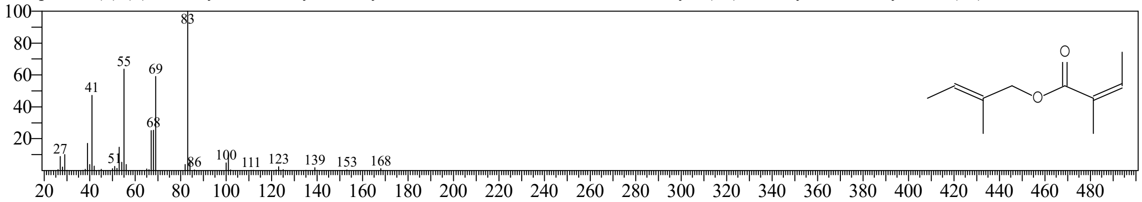

Hit#:4 Entry:29298 Library:NIST17-1.lib
SI:88 Formula:C8H16N2O2 CAS:108201-46-3 MolWeight:172 RetIndex:0
CompName:Diazene, 1-cyclohexyl-2-ethoxy-, 1-oxide $$ Cyclohexyl(ethoxy)diazene oxide # $$

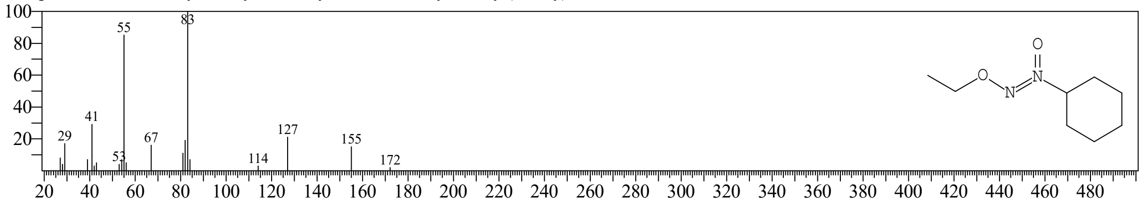

Hit#:5 Entry:20661 Library:NIST17-1.lib
SI:86 Formula:C7H14N2O2 CAS:72553-41-4 MolWeight:158 RetIndex:0
CompName:Diazene, 1-cyclohexyl-2-methoxy-, 1-oxide $$ 1-Cyclohexyl-2-methoxydiazene 1-oxide # $$

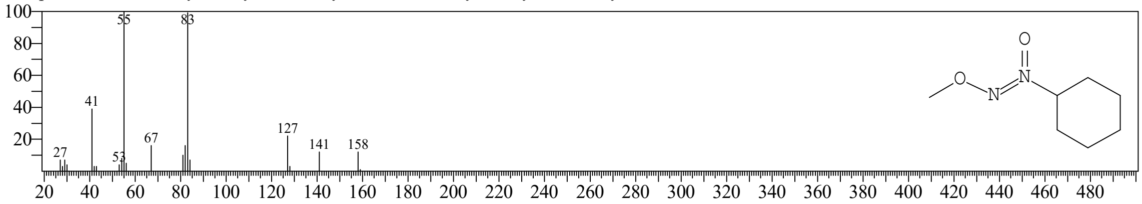

Qualitative Analysis Report

<< Target >>
Line#:24 R.Time:18.992(Scan#:3599) Retention Index:1161 MassPeaks:246
RawMode:Averaged 18.988-18.996(3598-3600) BasePeak:53.05(1106)
BG Mode:Calc. from Peak Group 1 - Event 1 Scan

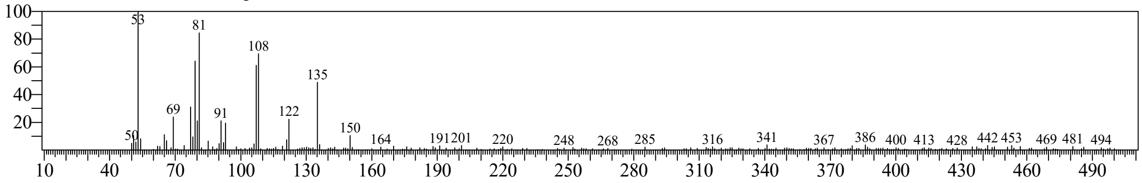

Hit#:1 Entry:73 Library:NIST17-1.lib
SI:98 Formula:F2HN CAS:10405-27-3 MolWeight:53 RetIndex:0
CompName:Difluoramine $$ Difluoroamine $$ HNF2 $$ Fluorimide $$ N,N-Difluoroamine # $$

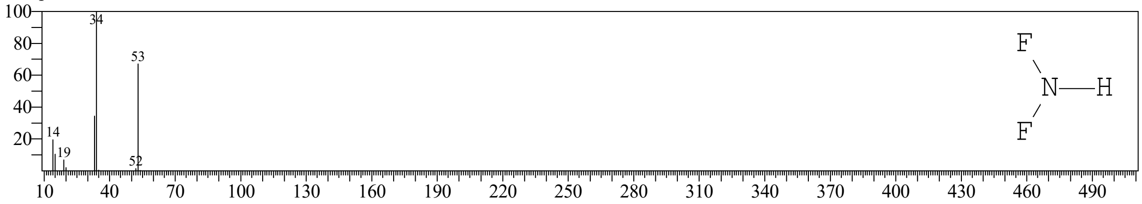

Hit#:2 Entry:1096 Library:FFNSC 3.lib
SI:91 Formula:C10 H14 O CAS:30460-92-5 MolWeight:150 RetIndex:1164
CompName:Pinocarvone

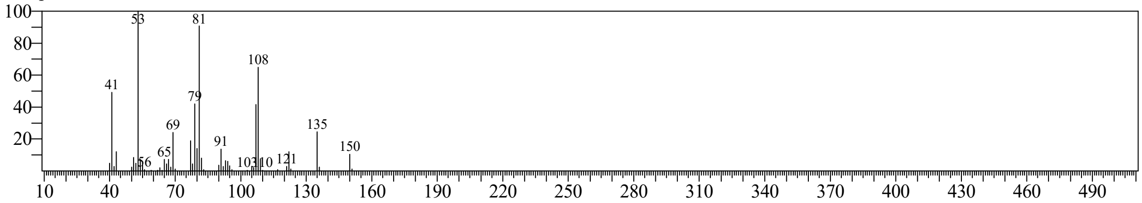

Hit#:3 Entry:574 Library:NIST17-1.lib
SI:84 Formula:C5H6O CAS:4009-55-6 MolWeight:82 RetIndex:0
CompName:3,4-Pentadienal

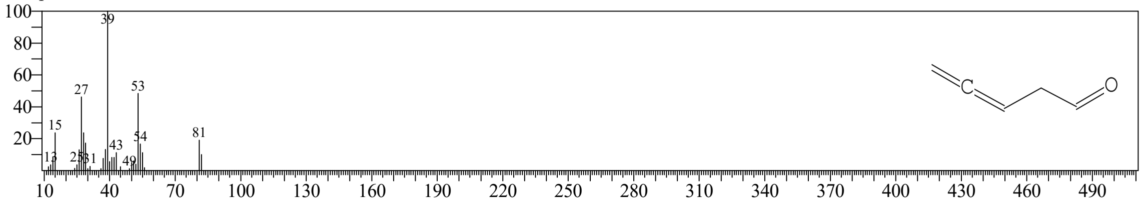

Hit#:4 Entry:33670 Library:NIST17-1.lib
SI:83 Formula:C7H9N5O CAS:0-00-0 MolWeight:179 RetIndex:0
CompName:6-Isopropoxytetrazolo[1,5-b]pyridazine

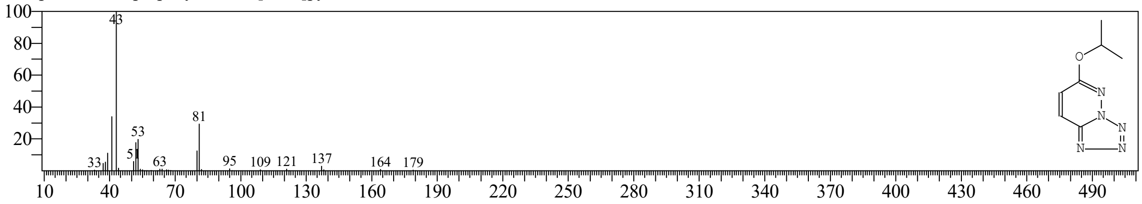

Hit#:5 Entry:1311 Library:NIST17-1.lib
SI:83 Formula:C7H10 CAS:3642-12-4 MolWeight:94 RetIndex:0
CompName:Hepta-1,2,6-heptatriene

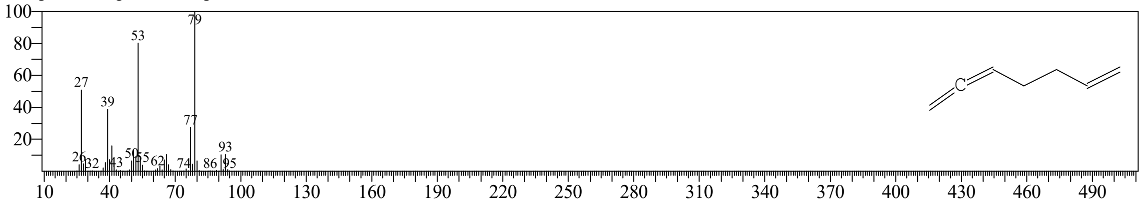

Qualitative Analysis Report

<< Target >>
Line#:25   R.Time:19.317(Scan#:3677) Retention Index:1168 MassPeaks:250
RawMode:Averaged 19.313-19.321(3676-3678) BasePeak:95.05(3580)
BG Mode:Calc. from Peak Group 1 - Event 1 Scan

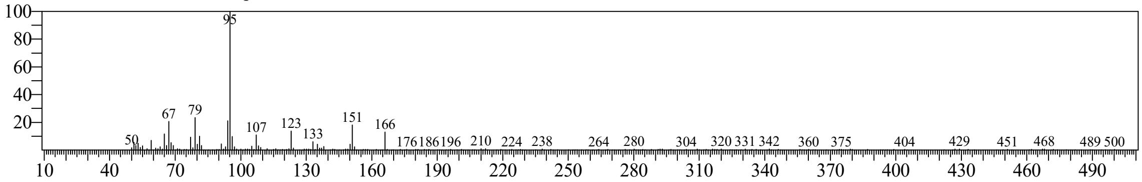

Hit#:1 Entry:1653 Library:FFNSC 3.lib
SI:95 Formula:C10 H14 O2 CAS:92356-06-4 MolWeight:166 RetIndex:1169
CompName:Rose furan oxide

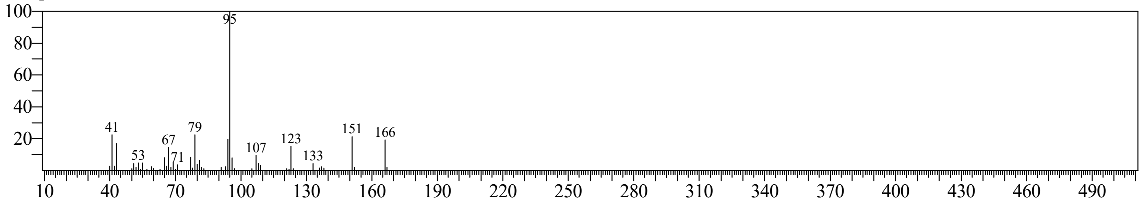

Hit#:2 Entry:67553 Library:NIST17-1.lib
SI:90 Formula:C7H11I CAS:34825-94-0 MolWeight:222 RetIndex:1177
CompName:Cyclohexene, 3-(iodomethyl)- $$ 3-(Iodomethyl)-1-cyclohexene # $$

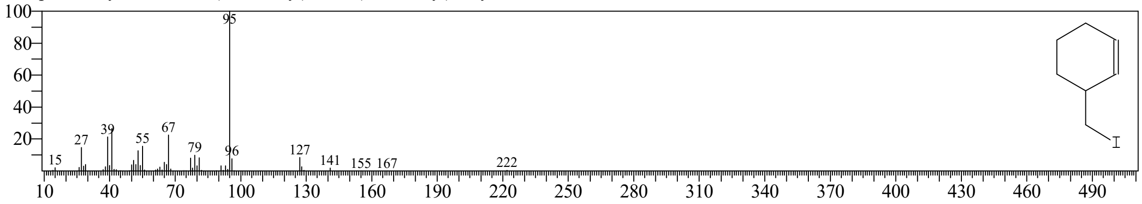

Hit#:3 Entry:25057 Library:NIST17-1.lib
SI:89 Formula:C7H12Cl2 CAS:32616-84-5 MolWeight:166 RetIndex:1172
CompName:Cycloheptane, 1,3-dichloro-, trans- $$ 1,3-Dichlorocycloheptane, (E)- $$

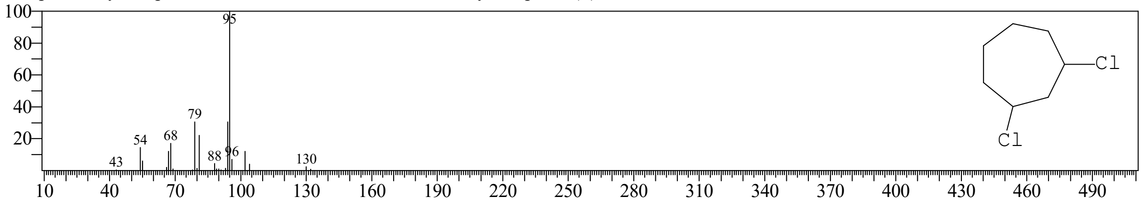

Hit#:4 Entry:11826 Library:NIST17-1.lib
SI:86 Formula:C8H12O2 CAS:0-00-0 MolWeight:140 RetIndex:1162
CompName:1,2-Dimethyl-cyclopent-2-enecarboxylic acid $$ 1,2-Dimethyl-2-cyclopentene-1-carboxylic acid # $$

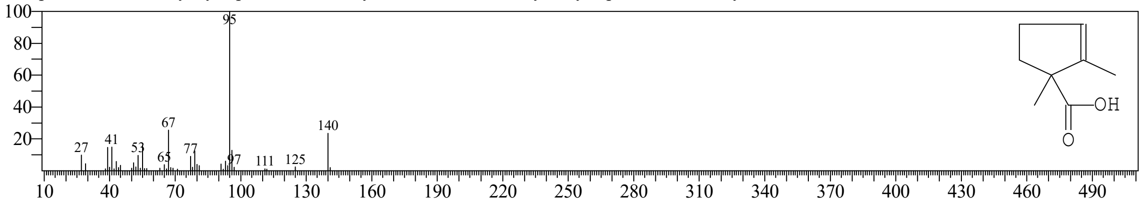

Hit#:5 Entry:25061 Library:NIST17-1.lib
SI:85 Formula:C7H12Cl2 CAS:32718-95-9 MolWeight:166 RetIndex:1172
CompName:Cycloheptane, 1,4-dichloro-, cis- $$ 1,4-Dichlorocycloheptane # $$

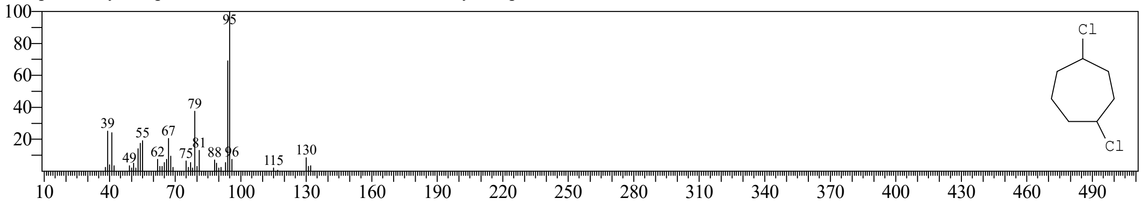

Qualitative Analysis Report

<< Target >>
Line#:26 R.Time:19.442(Scan#:3707) Retention Index:1171 MassPeaks:247
RawMode:Averaged 19.438-19.446(3706-3708) BasePeak:95.10(20691)
BG Mode:Calc. from Peak Group 1 - Event 1 Scan

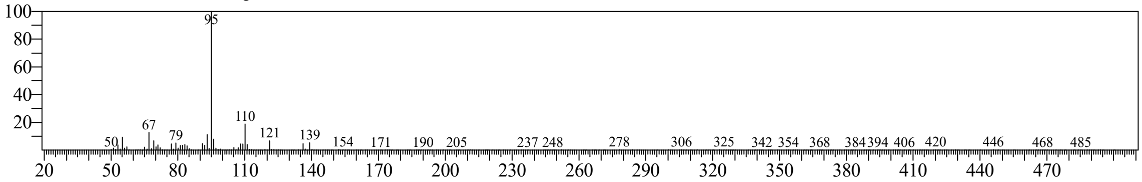

Hit#:1 Entry:1262 Library:FFNSC 3.lib
SI:96 Formula:C10 H18 O CAS:507-70-0 MolWeight:154 RetIndex:1173
CompName:Borneol

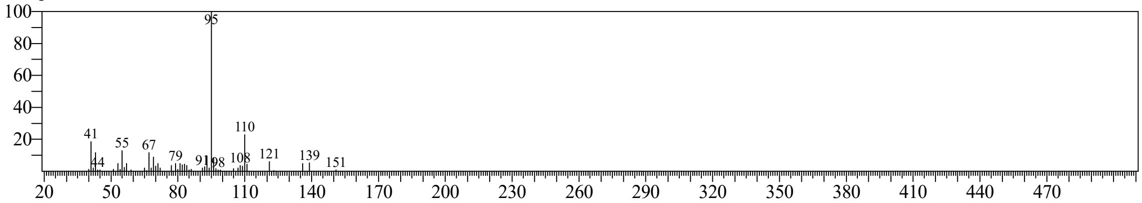

Hit#:2 Entry:687 Library:FFNSC 3.lib
SI:93 Formula:C10 H18 O CAS:10385-78-1 MolWeight:154 RetIndex:1165
CompName:Isoborneol

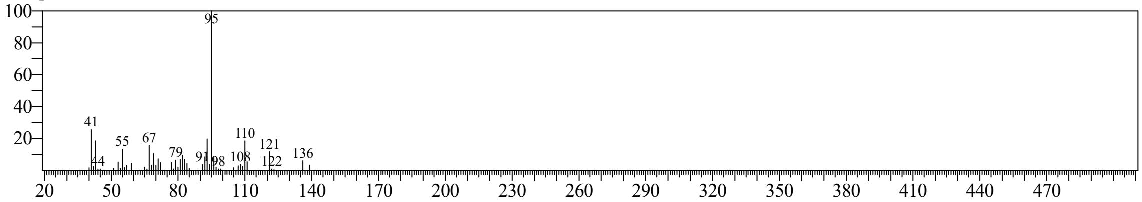

Hit#:3 Entry:26760 Library:NIST17-1.lib
SI:90 Formula:C10H16O2 CAS:5809-03-0 MolWeight:168 RetIndex:1172
CompName:2-Cyclopentene-1-carboxylic acid, 1,2-dimethyl-, ethyl ester $$ Ethyl 1,2-dimethyl-2-cyclopentene-1-carboxylate # $$

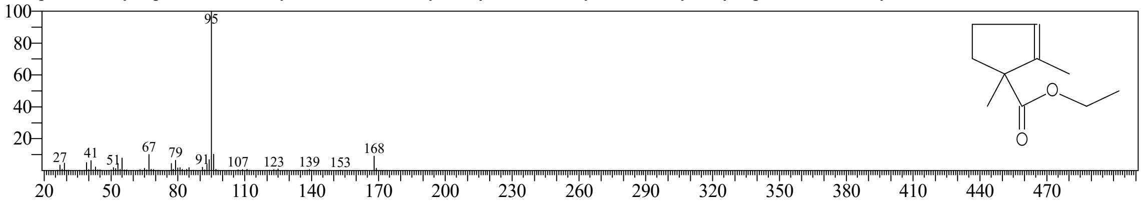

Hit#:4 Entry:36077 Library:NIST17-1.lib
SI:89 Formula:C10H14O3 CAS:0-00-0 MolWeight:182 RetIndex:1179
CompName:2-Furoic acid, 3-methylbut-2-yl ester

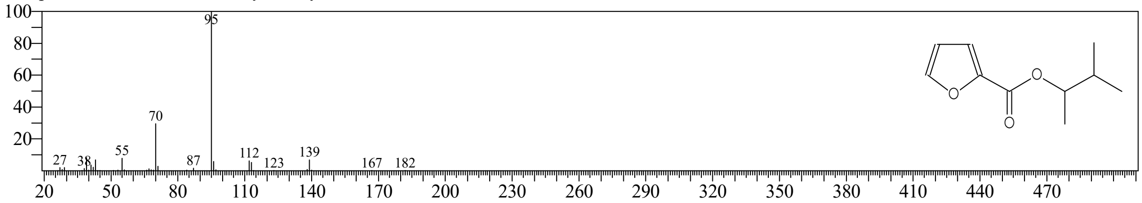

Hit#:5 Entry:11826 Library:NIST17-1.lib
SI:88 Formula:C8H12O2 CAS:0-00-0 MolWeight:140 RetIndex:1162
CompName:1,2-Dimethyl-cyclopent-2-enecarboxylic acid $$ 1,2-Dimethyl-2-cyclopentene-1-carboxylic acid # $$

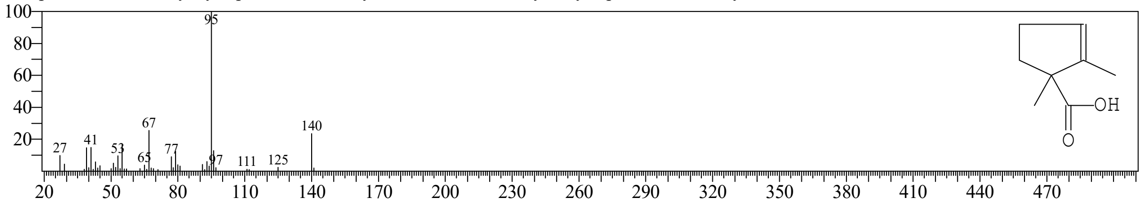

Qualitative Analysis Report

<< Target >>
Line#:27 R.Time:19.629(Scan#:3752) Retention Index:1175 MassPeaks:213
RawMode:Averaged 19.625-19.633(3751-3753) BasePeak:69.10(2727)
BG Mode:Calc. from Peak Group 1 - Event 1 Scan

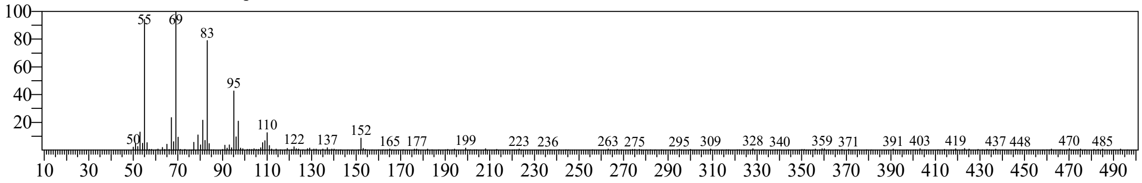

Hit#:1 Entry:228 Library:NIST17-1.lib
SI:99 Formula:C2H3N3 CAS:288-36-8 MolWeight:69 RetIndex:0
CompName:1H-1,2,3-Triazole $$ V-triazole $$ Osotriazole $$ Pyrrodiazole $$ Triazacyclopentadiene $$ 1,2,3-Triazole $$

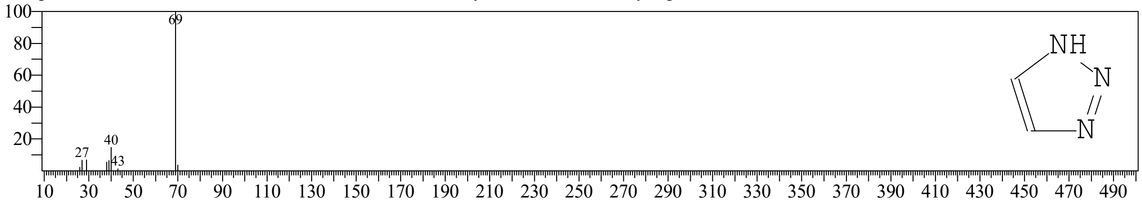

Hit#:2 Entry:1097 Library:FFNSC 3.lib
SI:96 Formula:C10 H16 O CAS:15358-88-0 MolWeight:152 RetIndex:1176
CompName:Pinocamphone <cis->

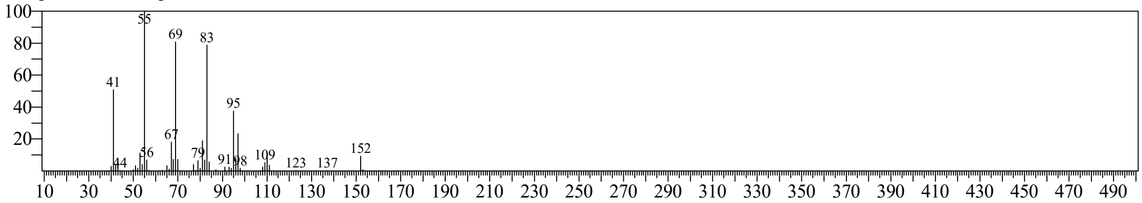

Hit#:3 Entry:981 Library:NIST17-1.lib
SI:95 Formula:CF4 CAS:75-73-0 MolWeight:88 RetIndex:0
CompName:Tetrafluoromethane $$ Carbon tetrafluoride $$ Methane, tetrafluoro- $$ Arcton 0 $$ Carbon fluoride (CF4) $$ Freon 14 $$ FC 14 $$ Perfluoro

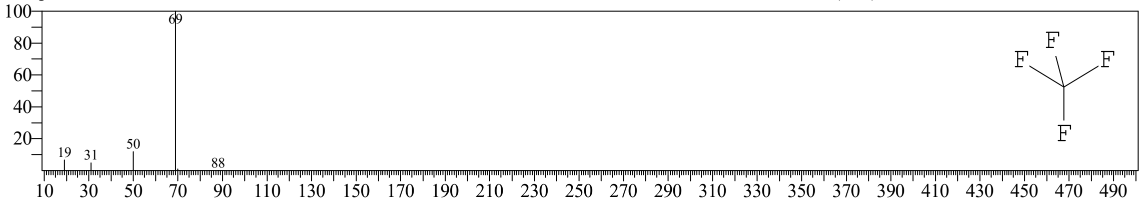

Hit#:4 Entry:10642 Library:NIST17-1.lib
SI:95 Formula:CF5P CAS:1112-04-5 MolWeight:138 RetIndex:0
CompName:Trifluoromethyldifluorophosphine $$ Trifluoromethylphosphonous difluoride # $$

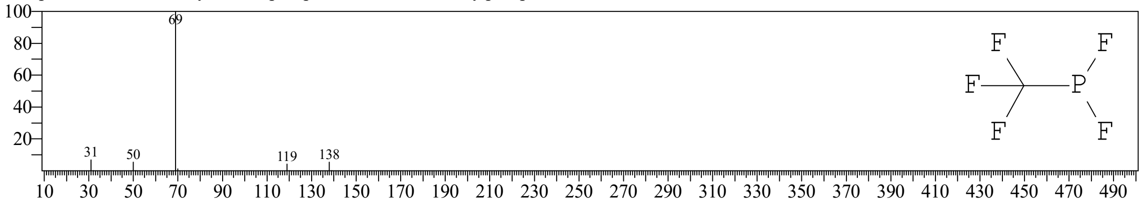

Hit#:5 Entry:9867 Library:NIST17-1.lib
SI:93 Formula:CF4OS CAS:812-12-4 MolWeight:136 RetIndex:0
CompName:Methanesulfinyl fluoride, trifluoro- $$ Trifluoromethanesulfinyl fluoride $$ Trifluoromethylsulfinyl fluoride $$

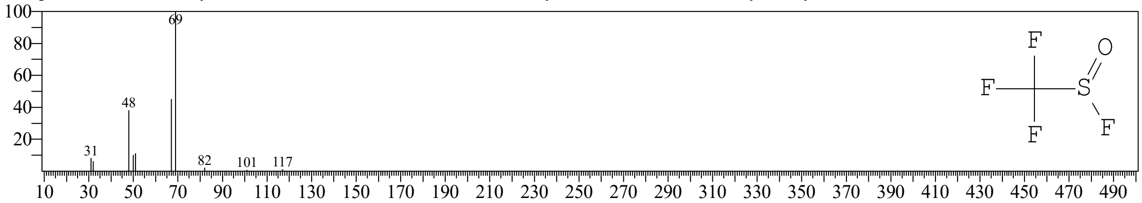

Qualitative Analysis Report

<< Target >>
Line#:28 R.Time:19.850(Scan#:3805) Retention Index:1180 MassPeaks:284
RawMode:Averaged 19.846-19.854(3804-3806) BasePeak:71.05(5315)
BG Mode:Calc. from Peak Group 1 - Event 1 Scan

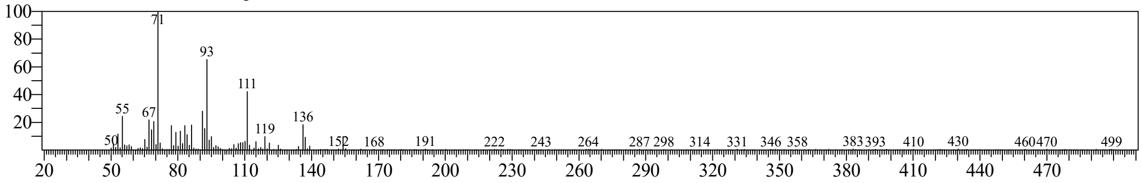

Hit#:1 Entry:1006 Library:FFNSC 3.lib
SI:95 Formula:C10 H18 O CAS:562-74-3 MolWeight:154 RetIndex:1184
CompName:Terpinen-4-ol

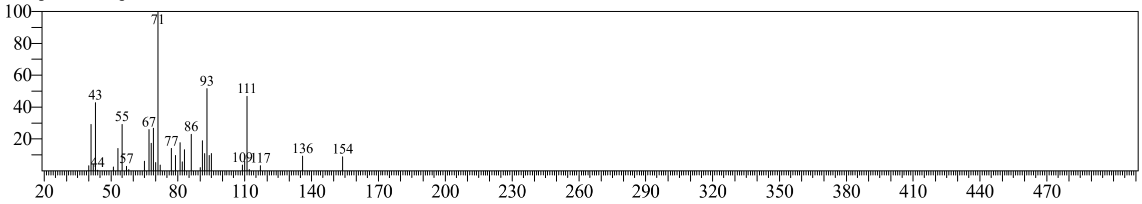

Hit#:2 Entry:223670 Library:NIST17-1.lib
SI:89 Formula:C24H48BNO2 CAS:0-00-0 MolWeight:393 RetIndex:0
CompName:N-Ethyl-2-amino-octade-4-en-1,3-diol buthaneboronate $$ N-(2-Butyl-4-[(1E)-1-pentadecenyl]-1,3,2-dioxaborinan-5-yl)-N-ethylamine # $$

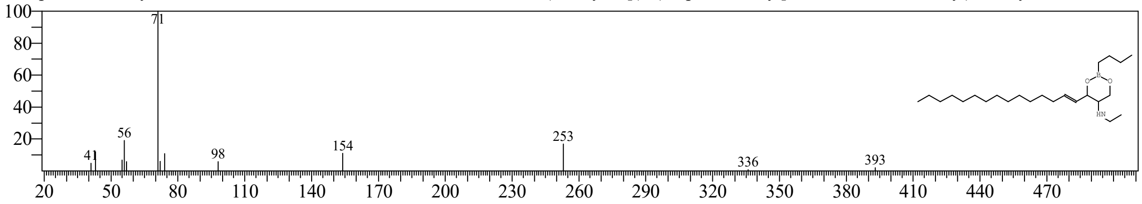

Hit#:3 Entry:13448 Library:NIST17-1.lib
SI:89 Formula:C4H4N2O2S CAS:22097-10-5 MolWeight:144 RetIndex:0
CompName:1,2,3-Thiadiazole-4-carboxylic acid, 5-methyl- $$ 5-Methyl-1,2,3-thiadiazole-4-carboxylic acid # $$

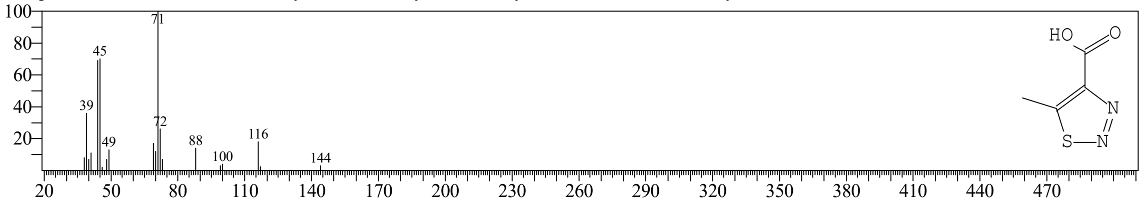

Hit#:4 Entry:233910 Library:NIST17-1.lib
SI:87 Formula:C26H44BNO2 CAS:0-00-0 MolWeight:413 RetIndex:0
CompName:N-Ethyl-2-amino-octade-4-en-1,3-diol benzeneboronate $$ N-Ethyl-4-[(1E)-1-pentadecenyl]-2-phenyl-1,3,2-dioxaborinan-5-amine # $$

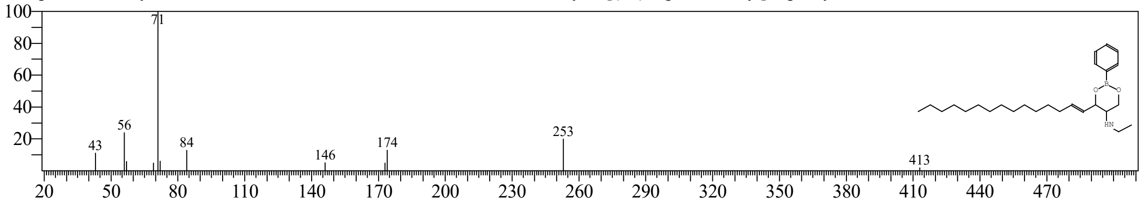

Hit#:5 Entry:50993 Library:NIST17-1.lib
SI:87 Formula:C12H26O2 CAS:0-00-0 MolWeight:202 RetIndex:1174
CompName:Acetaldehyde diisoamyl acetal

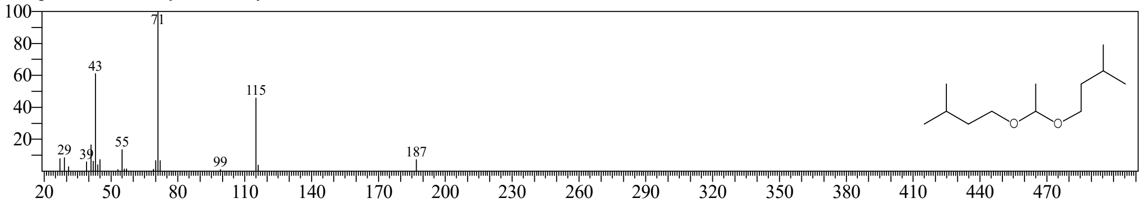

Qualitative Analysis Report

<< Target >>
Line#:29 R.Time:20.508(Scan#:3963) Retention Index:1194 MassPeaks:230
RawMode:Averaged 20.504-20.512(3962-3964) BasePeak:59.05(6059)
BG Mode:Calc. from Peak Group 1 - Event 1 Scan

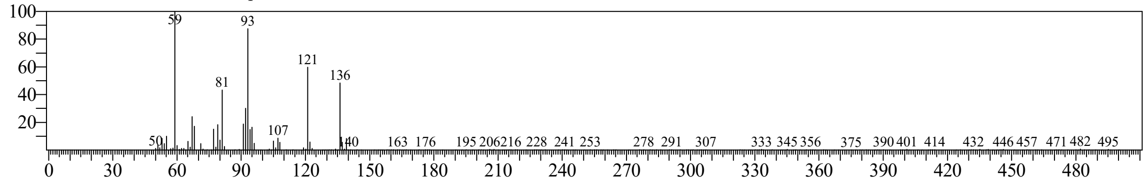

Hit#:1 Entry:120 Library:NIST17-1.lib
SI:97 Formula:CH5N3 CAS:113-00-8 MolWeight:59 RetIndex:0
CompName:Guanidine $$ Aminoformamidine $$ Aminomethanamidine $$ Carbamamidine $$ Carbamidine $$ Guanidin $$ Imidourea $$ Iminourea $$ (NI

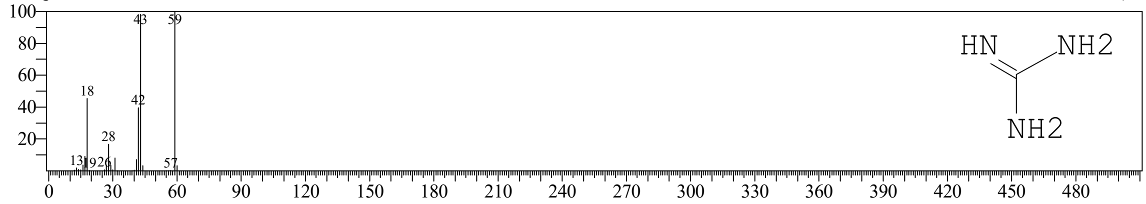

Hit#:2 Entry:497 Library:FFNSC 3.lib
SI:95 Formula:C10 H18 O CAS:98-55-5 MolWeight:154 RetIndex:1195
CompName:Terpineol <alpha->

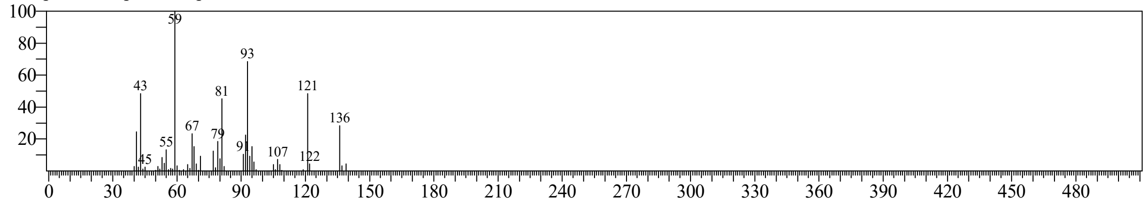

Hit#:3 Entry:27586 Library:NIST17-1.lib
SI:94 Formula:C4H12FN2PS CAS:36267-53-5 MolWeight:170 RetIndex:0
CompName:Phosphonofluoridothioic hydrazide, P-ethyl-2,2-dimethyl- $$ p-Ethyl-N',N'-dimethylphosphonohydrazidothioic fluoride # $$

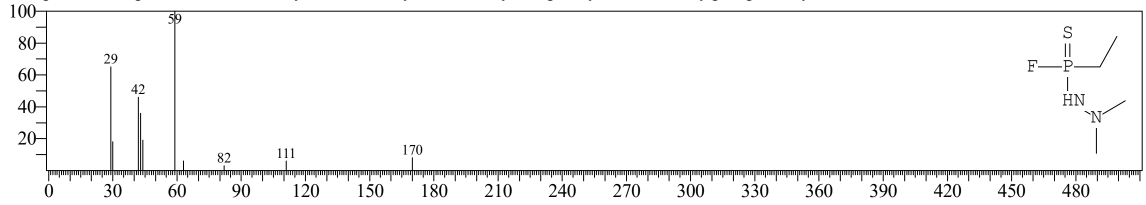

Hit#:4 Entry:20036 Library:NIST17-1.lib
SI:86 Formula:C10H20O CAS:16624-02-5 MolWeight:156 RetIndex:1191
CompName:Cycloheptanemethanol, .alpha.,.alpha.-dimethyl- $$ 2-Cycloheptyl-2-propanol # $$

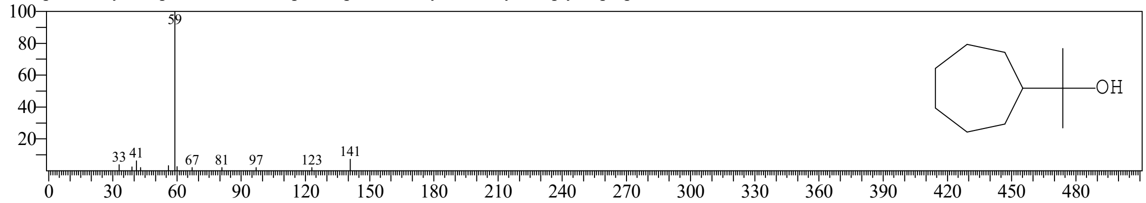

Hit#:5 Entry:144 Library:NIST17-1.lib
SI:86 Formula:C2H8Si CAS:1111-74-6 MolWeight:60 RetIndex:0
CompName:Silane, dimethyl- $$ Dimethylsilane $$ 2-Silapropane $$ (CH3)2SiH2 $$

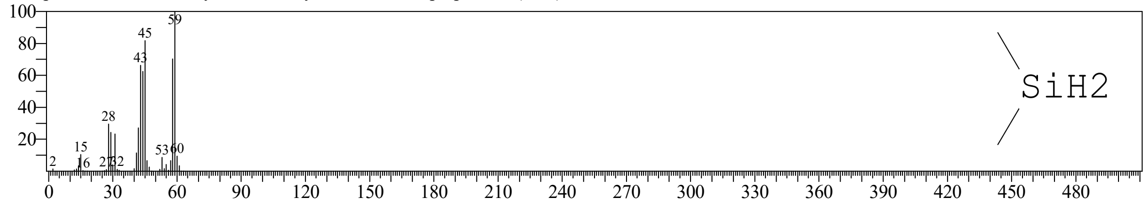

Qualitative Analysis Report

<< Target >>
Line#:30 R.Time:20.875(Scan#:4051) Retention Index:1202 MassPeaks:296
RawMode:Averaged 20.871-20.879(4050-4052) BasePeak:95.10(2510)
BG Mode:Calc. from Peak Group 1 - Event 1 Scan

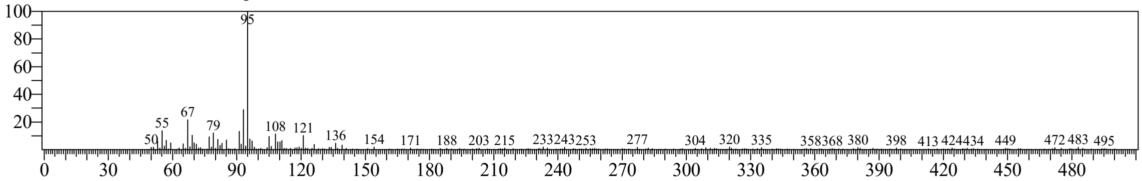

Hit#:1 Entry:11236 Library:NIST17-1.lib
SI:87 Formula:CH2BrNO2 CAS:563-70-2 MolWeight:139 RetIndex:0
CompName:Bromonitromethane $$ Methane, bromonitro- $$

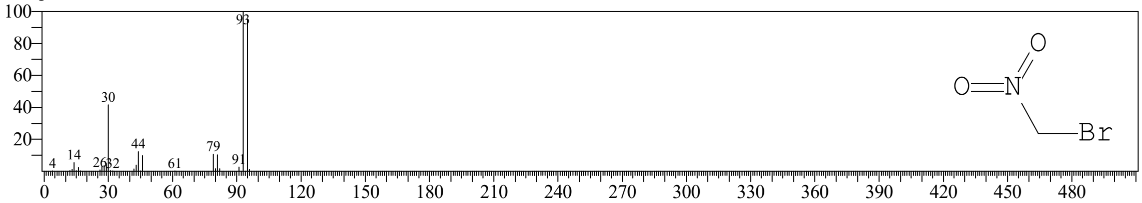

Hit#:2 Entry:6426 Library:NIST17-1.lib
SI:86 Formula:C2H8NO3P CAS:2697-42-9 MolWeight:125 RetIndex:0
CompName:Phosphoramidic acid, dimethyl ester $$ Dimethyl phosphoramidate $$ Dimethyl amidophosphate # $$

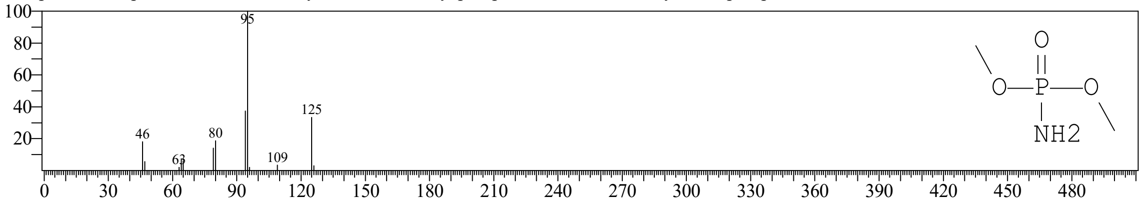

Hit#:3 Entry:25625 Library:NIST17-1.lib
SI:85 Formula:C11H18O CAS:19780-98-4 MolWeight:166 RetIndex:1197
CompName:2,6-Dimethyl-1-nonen-3-yn-5-ol

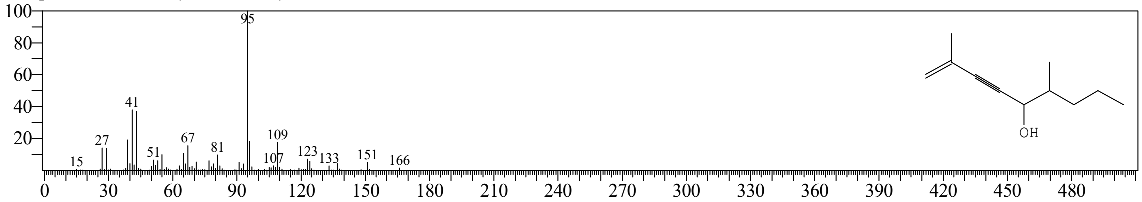

Hit#:4 Entry:10478 Library:NIST17-1.lib
SI:84 Formula:C5H7N5 CAS:0-00-0 MolWeight:137 RetIndex:0
CompName:4-(Azidomethyl)-1-methylpyrazole

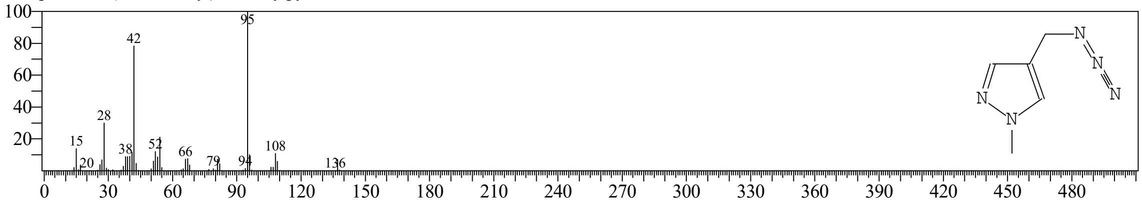

Hit#:5 Entry:36423 Library:NIST17-1.lib
SI:84 Formula:C12H22O CAS:77142-78-0 MolWeight:182 RetIndex:1203
CompName:5,7-Octadien-3-ol, 2,4,4,7-tetramethyl-, (E)- $$ (5E)-2,4,4,7-Tetramethyl-5,7-octadien-3-ol # $$

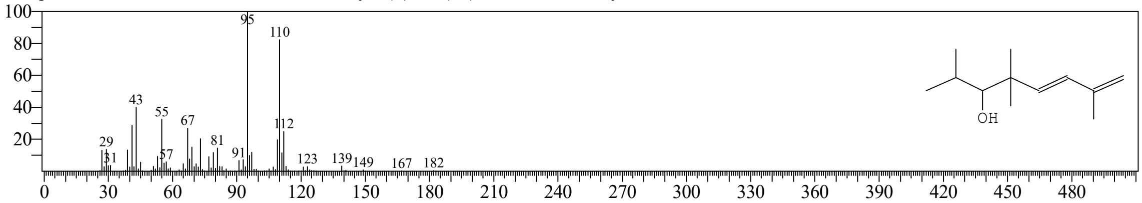

Qualitative Analysis Report

<< Target >>
Line#:31 R.Time:21.054(Scan#:4094) Retention Index:1205 MassPeaks:294
RawMode:Averaged 21.050-21.058(4093-4095) BasePeak:107.10(41442)
BG Mode:Calc. from Peak Group 1 - Event 1 Scan

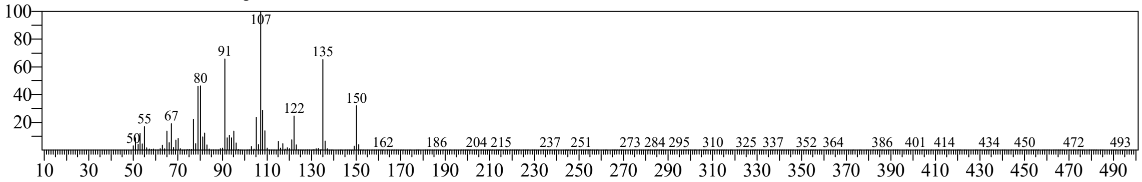

Hit#:1 Entry:820 Library:FFNSC 3.lib
SI:97 Formula:C10 H14 O CAS:80-57-9 MolWeight:150 RetIndex:1208
CompName:Verbenone

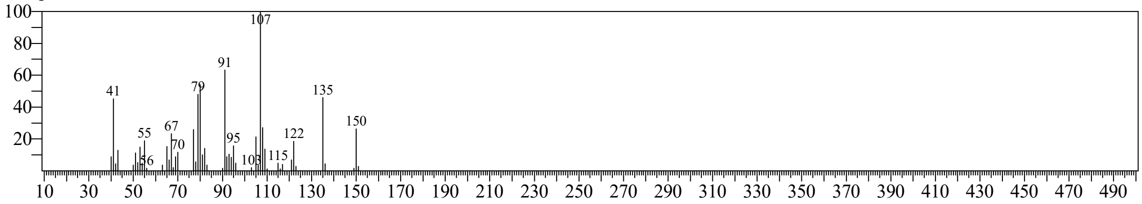

Hit#:2 Entry:7085 Library:NIST17-1.lib
SI:97 Formula:F5P CAS:7647-19-0 MolWeight:126 RetIndex:0
CompName:Phosphorus pentafluoride $$ Phosphorus(V) fluoride $$ PF5 $$ Phosphorane, pentafluoro- $$ UN 2198 $$ Phosphorus fluoride $$ Pentafluoro

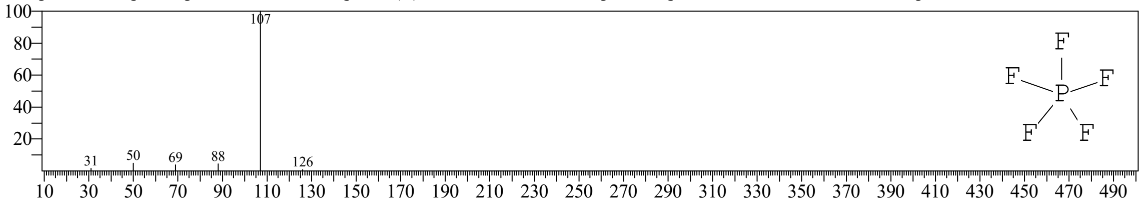

Hit#:3 Entry:9872 Library:NIST17-1.lib
SI:95 Formula:C2H5F4P CAS:0-00-0 MolWeight:136 RetIndex:0
CompName:Ethyltetrafluorophosphorane

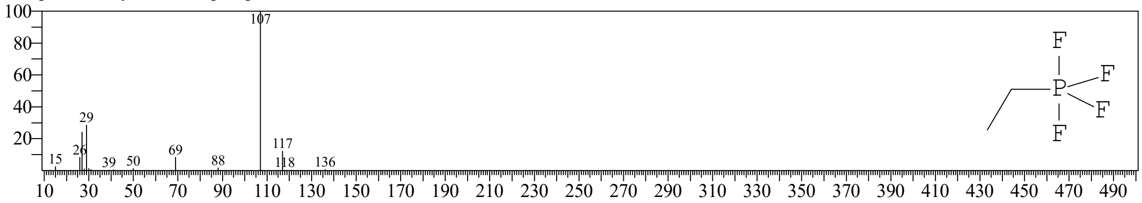

Hit#:4 Entry:33195 Library:NIST17-1.lib
SI:93 Formula:C10H14N2O CAS:14487-70-8 MolWeight:178 RetIndex:0
CompName:3-Diazo-1,7,7-trimethylbicyclo[2.2.1]heptan-2-one

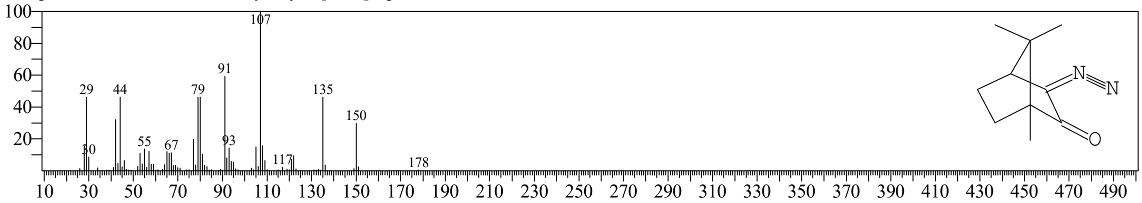

Hit#:5 Entry:9296 Library:NIST17-1.lib
SI:93 Formula:C2H3F4P CAS:3583-99-1 MolWeight:134 RetIndex:0
CompName:Tetrafluorovinylphosphorane

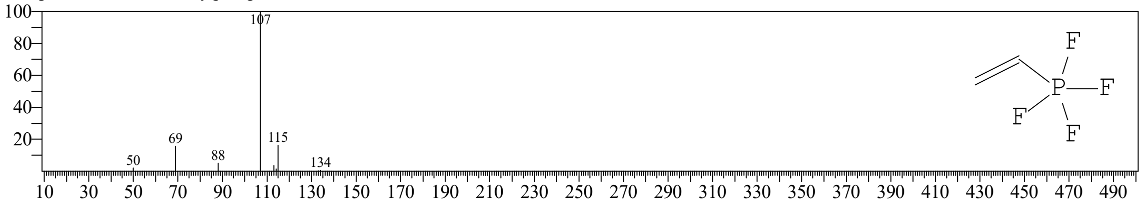

Qualitative Analysis Report

<< Target >>
Line#:32 R.Time:21.658(Scan#:4239) Retention Index:1219 MassPeaks:278
RawMode:Averaged 21.654-21.663(4238-4240) BasePeak:69.10(4450)
BG Mode:Calc. from Peak Group 1 - Event 1 Scan

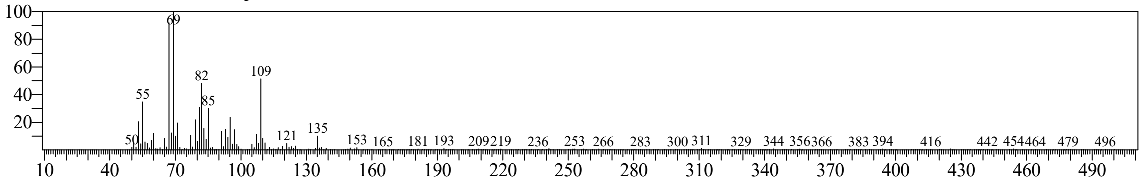

Hit#:1 Entry:228 Library:NIST17-1.lib
SI:99 Formula:C2H3N3 CAS:288-36-8 MolWeight:69 RetIndex:0
CompName:1H-1,2,3-Triazole $$ V-triazole $$ Osotriazole $$ Pyrrodiazole $$ Triazacyclopentadiene $$ 1,2,3-Triazole $$

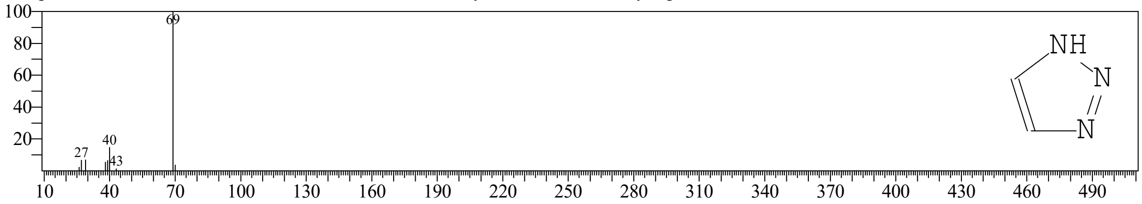

Hit#:2 Entry:981 Library:NIST17-1.lib
SI:95 Formula:CF4 CAS:75-73-0 MolWeight:88 RetIndex:0
CompName:Tetrafluoromethane $$ Carbon tetrafluoride $$ Methane, tetrafluoro- $$ Arcton 0 $$ Carbon fluoride (CF4) $$ Freon 14 $$ FC 14 $$ Perfluoro

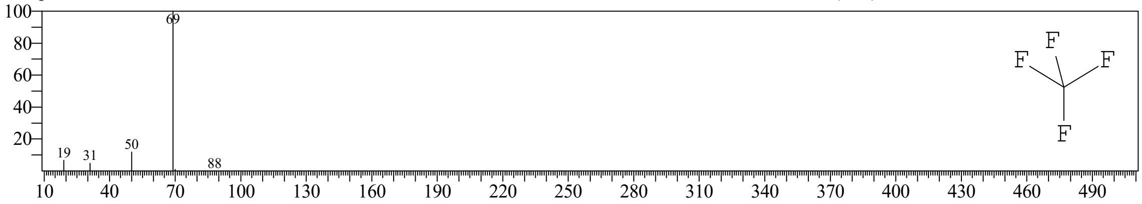

Hit#:3 Entry:10642 Library:NIST17-1.lib
SI:95 Formula:CF5P CAS:1112-04-5 MolWeight:138 RetIndex:0
CompName:Trifluoromethyldifluorophosphine $$ Trifluoromethylphosphonous difluoride # $$

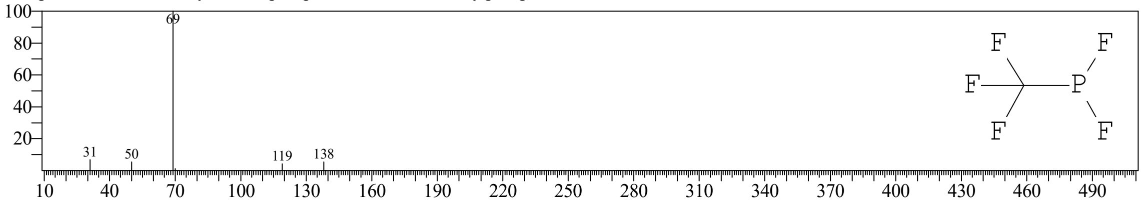

Hit#:4 Entry:10455 Library:NIST17-1.lib
SI:93 Formula:CClF4N CAS:13880-72-3 MolWeight:137 RetIndex:0
CompName:N-Chloro-N-fluoro-trifluoromethylamine $$ N-Chloro-N-tetrafluoromethanamine # $$

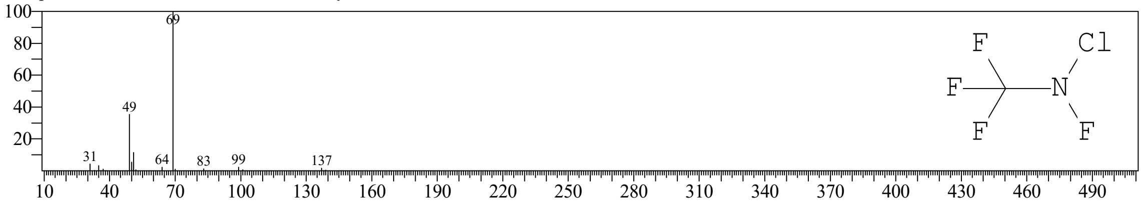

Hit#:5 Entry:216304 Library:NIST17-1.lib
SI:92 Formula:C7F14O2 CAS:42133-36-8 MolWeight:382 RetIndex:0
CompName:Acetic acid, trifluoro-, undecafluoropentyl ester $$ 1,1,2,2,3,3,4,4,5,5,5-Undecafluoropentyl trifluoroacetate # $$

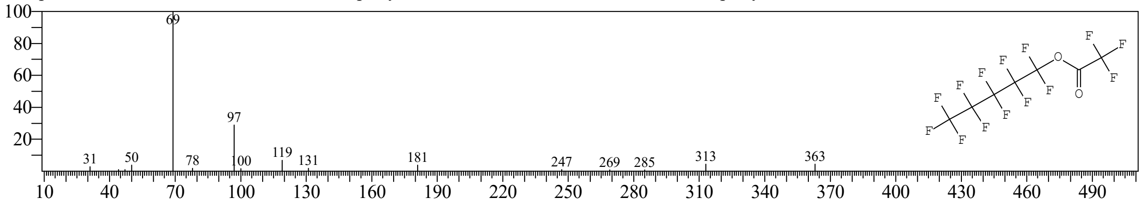

Qualitative Analysis Report

<< Target >>
Line#:33 R.Time:21.846(Scan#:4284) Retention Index:1223 MassPeaks:294
RawMode:Averaged 21.842-21.850(4283-4285) BasePeak:69.10(3139)
BG Mode:Calc. from Peak Group 1 - Event 1 Scan

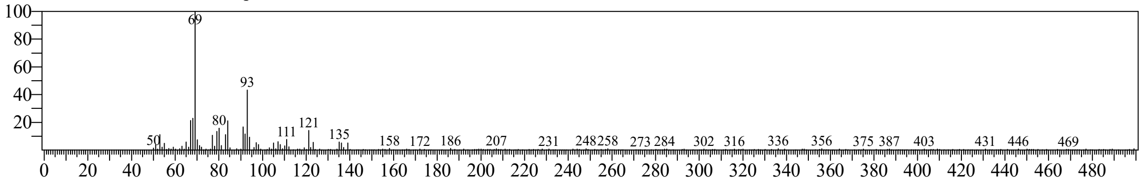

Hit#:1 Entry:228 Library:NIST17-1.lib
SI:99 Formula:C2H3N3 CAS:288-36-8 MolWeight:69 RetIndex:0
CompName:1H-1,2,3-Triazole $$ V-triazole $$ Osotriazole $$ Pyrrodiazole $$ Triazacyclopentadiene $$ 1,2,3-Triazole $$

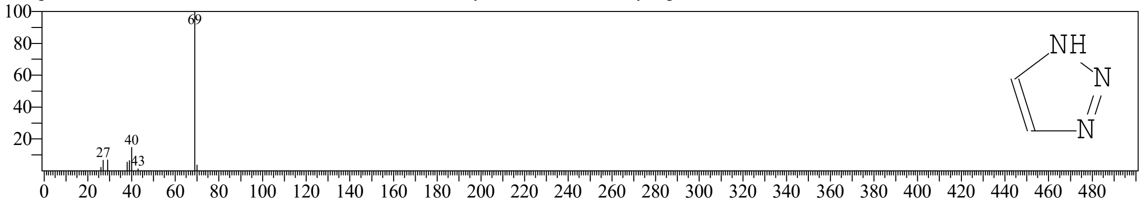

Hit#:2 Entry:981 Library:NIST17-1.lib
SI:95 Formula:CF4 CAS:75-73-0 MolWeight:88 RetIndex:0
CompName:Tetrafluoromethane $$ Carbon tetrafluoride $$ Methane, tetrafluoro- $$ Arcton 0 $$ Carbon fluoride (CF4) $$ Freon 14 $$ FC 14 $$ Perfluoro

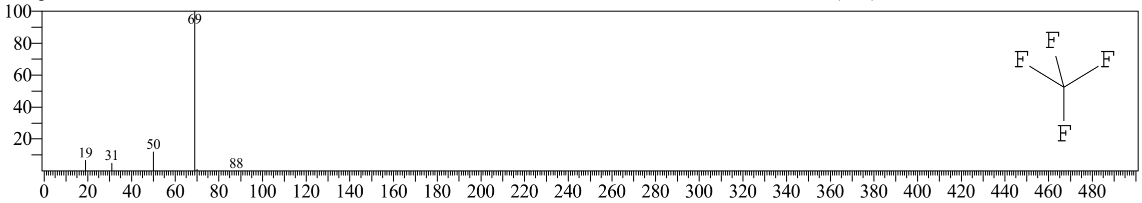

Hit#:3 Entry:10455 Library:NIST17-1.lib
SI:93 Formula:CClF4N CAS:13880-72-3 MolWeight:137 RetIndex:0
CompName:N-Chloro-N-fluoro-trifluoromethylamine $$ N-Chloro-N-tetrafluoromethanamine # $$

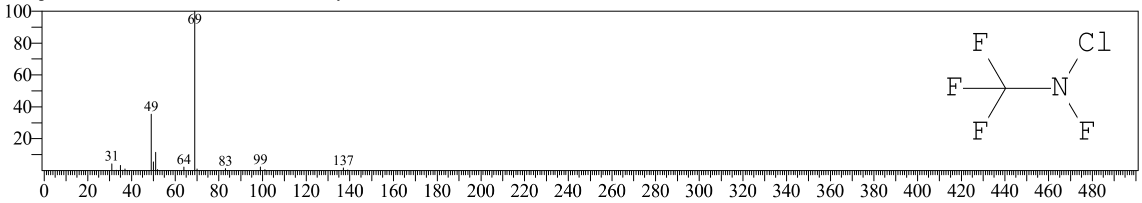

Hit#:4 Entry:10642 Library:NIST17-1.lib
SI:93 Formula:CF5P CAS:1112-04-5 MolWeight:138 RetIndex:0
CompName:Trifluoromethyldifluorophosphine $$ Trifluoromethylphosphonous difluoride # $$

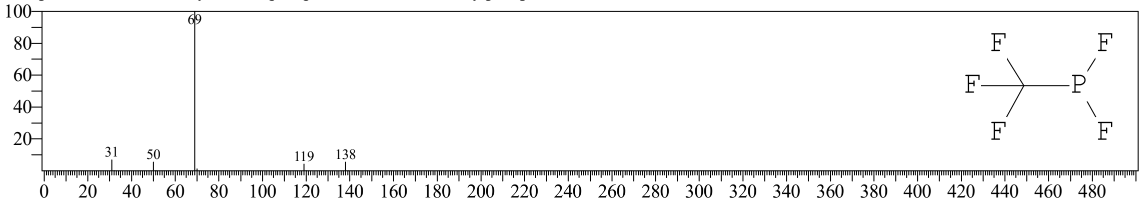

Hit#:5 Entry:1768 Library:NIST17-1.lib
SI:93 Formula:C6H15B CAS:97-94-9 MolWeight:98 RetIndex:0
CompName:Borane, triethyl- $$ Triethylborane $$ Triethylboron $$ (C2H5)3B $$ Triethylborine $$ Borethyl $$

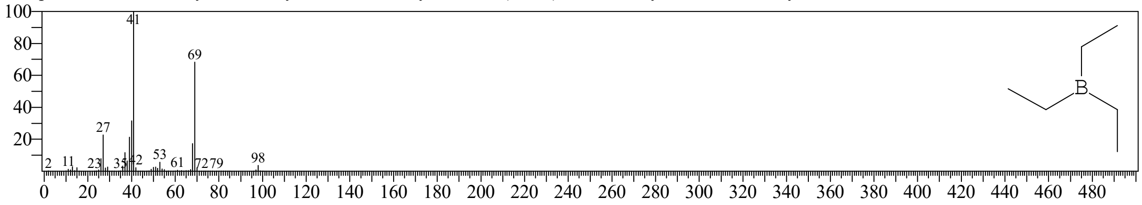

Qualitative Analysis Report

<< Target >>
Line#:34 R.Time:21.954(Scan#:4310) Retention Index:1225 MassPeaks:286
RawMode:Averaged 21.950-21.958(4309-4311) BasePeak:69.10(4019)
BG Mode:Calc. from Peak Group 1 - Event 1 Scan

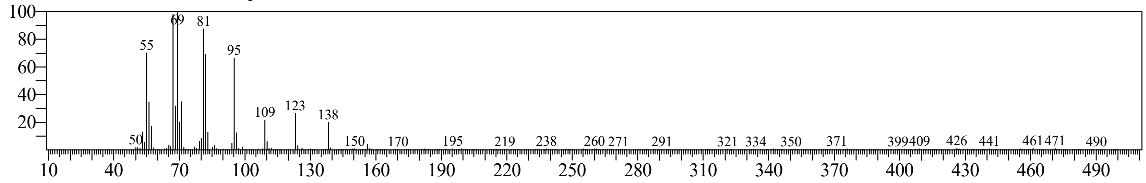

Hit#:1 Entry:228 Library:NIST17-1.lib
SI:96 Formula:C2H3N3 CAS:288-36-8 MolWeight:69 RetIndex:0
CompName:1H-1,2,3-Triazole $$ V-triazole $$ Osotriazole $$ Pyrrodiazole $$ Triazacyclopentadiene $$ 1,2,3-Triazole $$

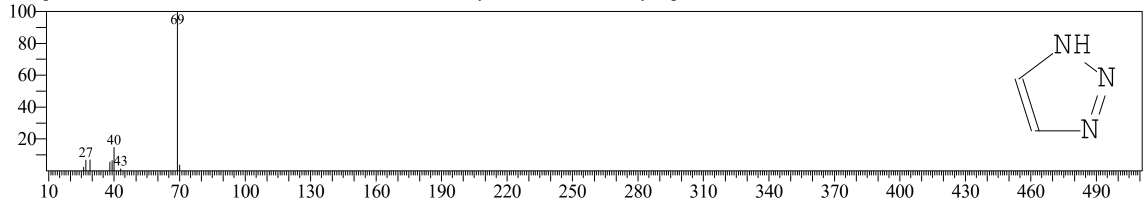

Hit#:2 Entry:981 Library:NIST17-1.lib
SI:94 Formula:CF4 CAS:75-73-0 MolWeight:88 RetIndex:0
CompName:Tetrafluoromethane $$ Carbon tetrafluoride $$ Methane, tetrafluoro- $$ Arcton 0 $$ Carbon fluoride (CF4) $$ Freon 14 $$ FC 14 $$ Perfluoro

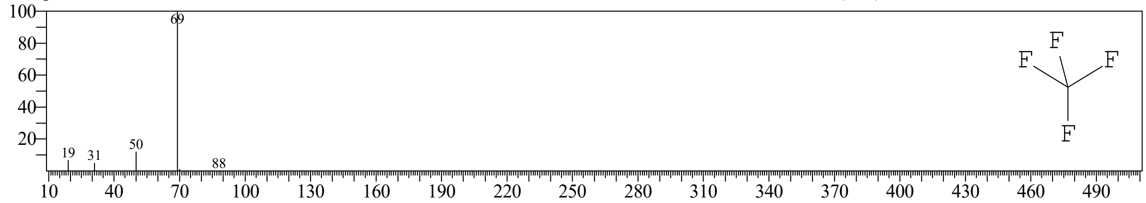

Hit#:3 Entry:922 Library:FFNSC 3.lib
SI:93 Formula:C10 H20 O CAS:106-22-9 MolWeight:156 RetIndex:1232
CompName:Citronellol

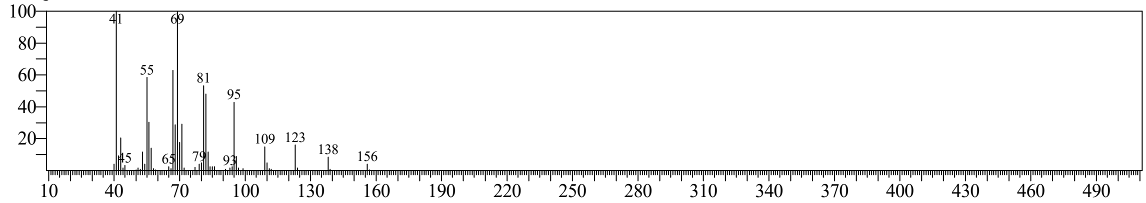

Hit#:4 Entry:577 Library:FFNSC 3.lib
SI:92 Formula:C10 H20 O CAS:141-25-3 MolWeight:156 RetIndex:1228
CompName:Oct-7-enol <3,7-dimethyl->

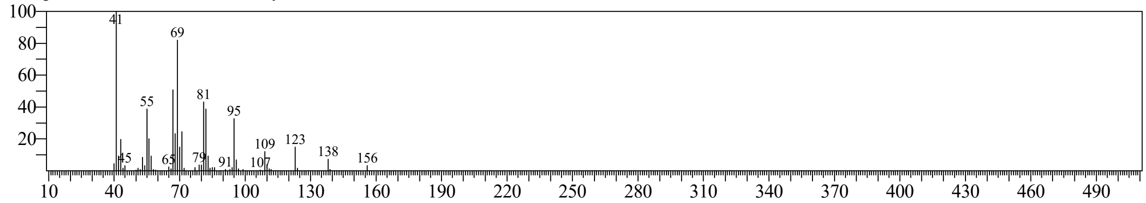

Hit#:5 Entry:10642 Library:NIST17-1.lib
SI:91 Formula:CF5P CAS:1112-04-5 MolWeight:138 RetIndex:0
CompName:Trifluoromethyldifluorophosphine $$ Trifluoromethylphosphonous difluoride # $$

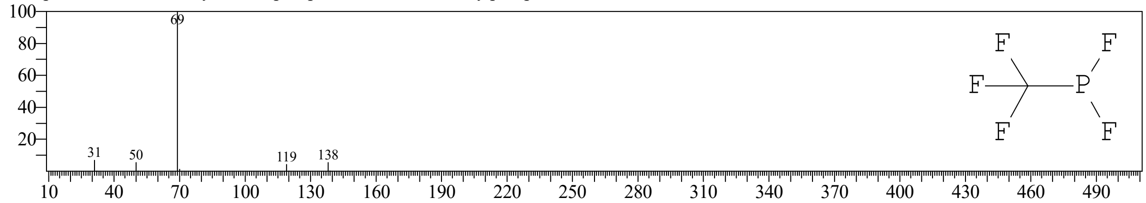

Qualitative Analysis Report

<< Target >>
Line#:35 R.Time:22.137(Scan#:4354) Retention Index:1229 MassPeaks:291
RawMode:Averaged 22.133-22.142(4353-4355) BasePeak:69.10(8623)
BG Mode:Calc. from Peak Group 1 - Event 1 Scan

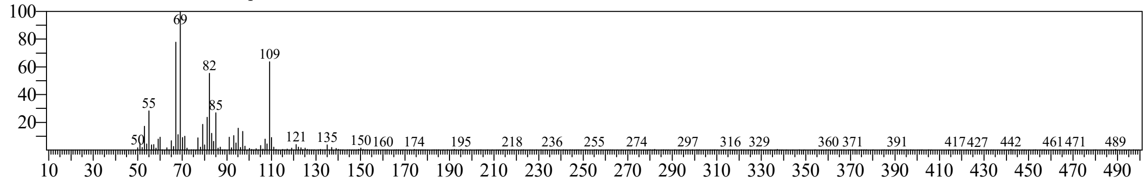

Hit#:1 Entry:228 Library:NIST17-1.lib
SI:99 Formula:C2H3N3 CAS:288-36-8 MolWeight:69 RetIndex:0
CompName:1H-1,2,3-Triazole $$ V-triazole $$ Osotriazole $$ Pyrrodiazole $$ Triazacyclopentadiene $$ 1,2,3-Triazole $$

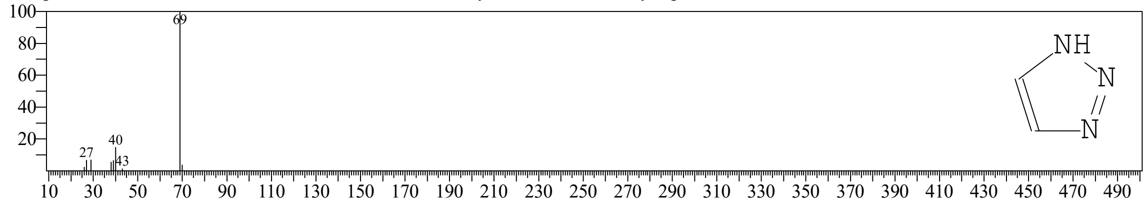

Hit#:2 Entry:981 Library:NIST17-1.lib
SI:95 Formula:CF4 CAS:75-73-0 MolWeight:88 RetIndex:0
CompName:Tetrafluoromethane $$ Carbon tetrafluoride $$ Methane, tetrafluoro- $$ Arcton 0 $$ Carbon fluoride (CF4) $$ Freon 14 $$ FC 14 $$ Perfluoro

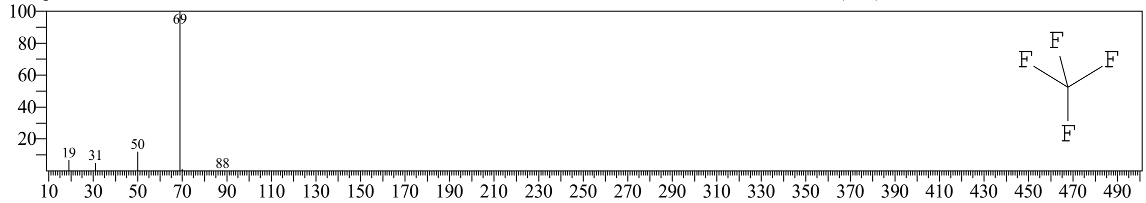

Hit#:3 Entry:10455 Library:NIST17-1.lib
SI:93 Formula:CClF4N CAS:13880-72-3 MolWeight:137 RetIndex:0
CompName:N-Chloro-N-fluoro-trifluoromethylamine $$ N-Chloro-N-tetrafluoromethanamine # $$

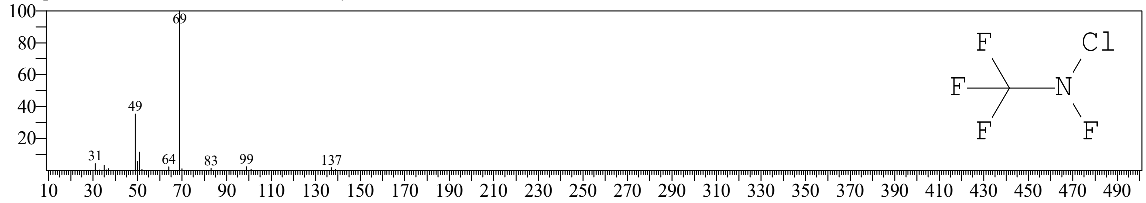

Hit#:4 Entry:10642 Library:NIST17-1.lib
SI:93 Formula:CF5P CAS:1112-04-5 MolWeight:138 RetIndex:0
CompName:Trifluoromethyldifluorophosphine $$ Trifluoromethylphosphonous difluoride # $$

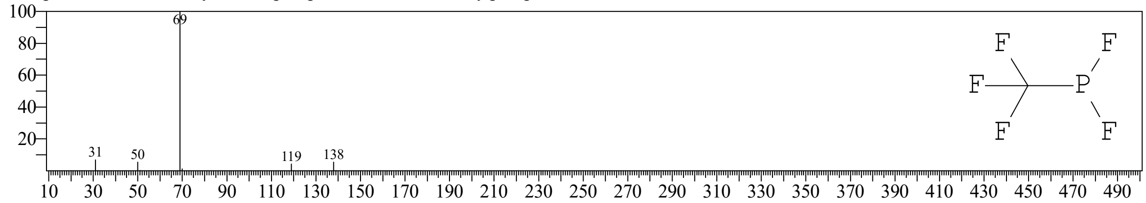

Hit#:5 Entry:2524 Library:NIST17-1.lib
SI:92 Formula:CClF3 CAS:75-72-9 MolWeight:104 RetIndex:0
CompName:Chlorotrifluoromethane $$ Monochlorotrifluoromethane $$ Methane, chlorotrifluoro- $$ Arcton 3 $$ F 13 $$ Freon 13 $$ FC 13 $$ Genetron

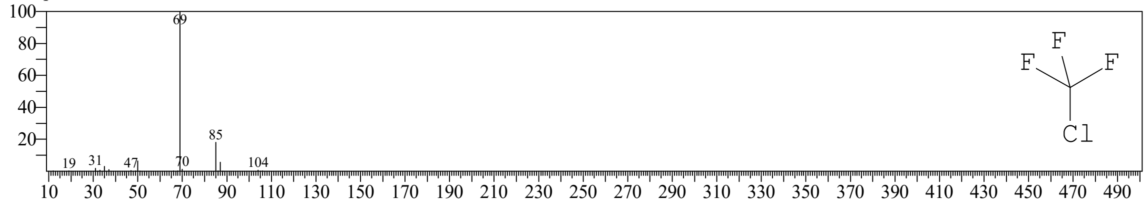

Qualitative Analysis Report

<< Target >>
Line#:36 R.Time:22.496(Scan#:4440) Retention Index:1237 MassPeaks:305
RawMode:Averaged 22.492-22.500(4439-4441) BasePeak:69.10(349351)
BG Mode:Calc. from Peak Group 1 - Event 1 Scan

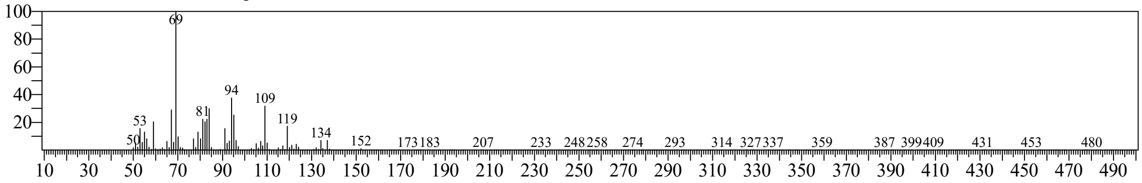

Hit#:1 Entry:228 Library:NIST17-1.lib
SI:99 Formula:C2H3N3 CAS:288-36-8 MolWeight:69 RetIndex:0
CompName:1H-1,2,3-Triazole $$ V-triazole $$ Osotriazole $$ Pyrrodiazole $$ Triazacyclopentadiene $$ 1,2,3-Triazole $$

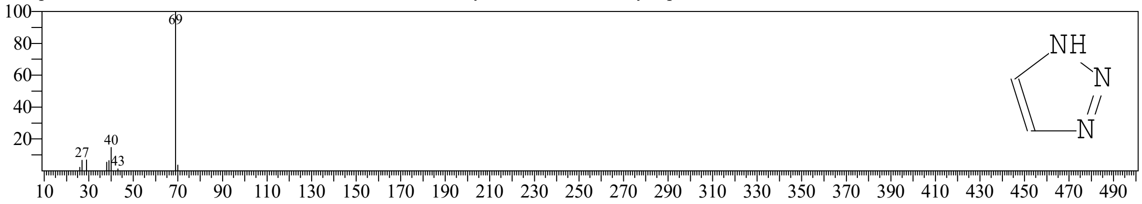

Hit#:2 Entry:1002 Library:FFNSC 3.lib
SI:97 Formula:C10 H16 O CAS:106-26-3 MolWeight:152 RetIndex:1238
CompName:Neral

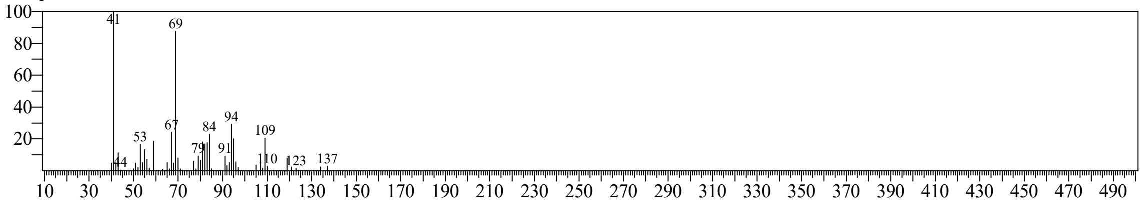

Hit#:3 Entry:981 Library:NIST17-1.lib
SI:95 Formula:CF4 CAS:75-73-0 MolWeight:88 RetIndex:0
CompName:Tetrafluoromethane $$ Carbon tetrafluoride $$ Methane, tetrafluoro- $$ Arcton 0 $$ Carbon fluoride (CF4) $$ Freon 14 $$ FC 14 $$ Perfluoro

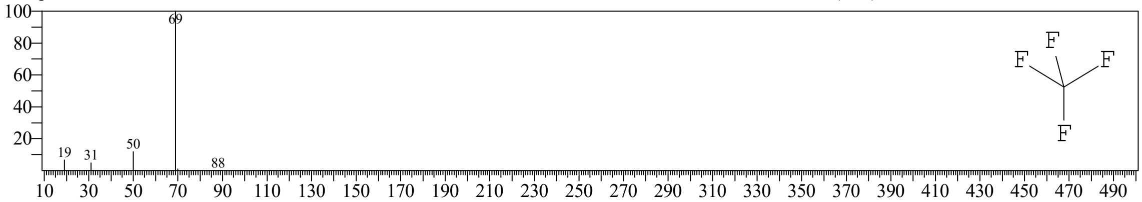

Hit#:4 Entry:10642 Library:NIST17-1.lib
SI:93 Formula:CF5P CAS:1112-04-5 MolWeight:138 RetIndex:0
CompName:Trifluoromethyldifluorophosphine $$ Trifluoromethylphosphonous difluoride # $$

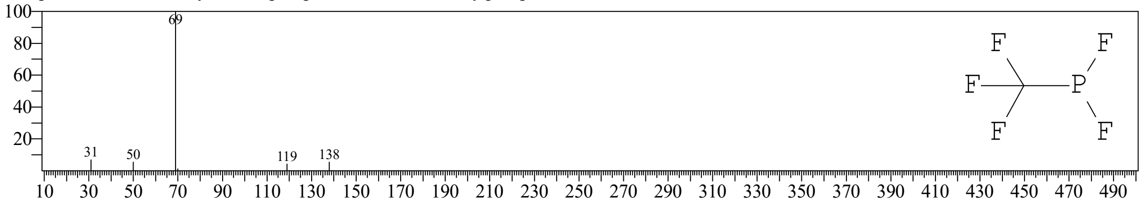

Hit#:5 Entry:1337 Library:NIST17-1.lib
SI:92 Formula:CF2NP CAS:14118-40-2 MolWeight:95 RetIndex:0
CompName:Phosphorocyanidous difluoride $$ Difluorocyanophosphine $$

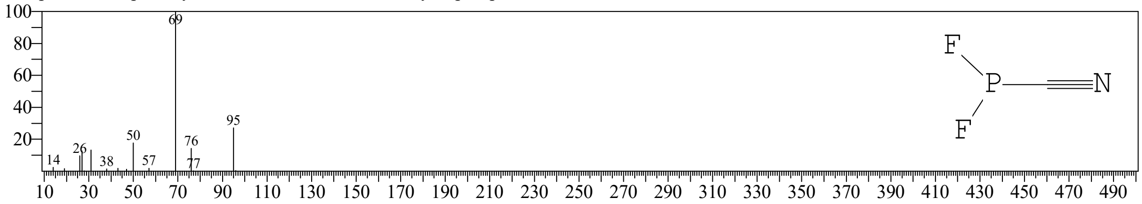

Qualitative Analysis Report

<< Target >>
Line#:37 R.Time:23.038(Scan#:4570) Retention Index:1249 MassPeaks:254
RawMode:Averaged 23.033-23.042(4569-4571) BasePeak:69.10(69630)
BG Mode:Calc. from Peak Group 1 - Event 1 Scan

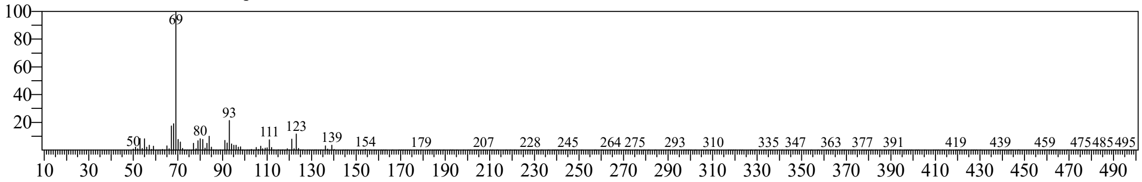

Hit#:1 Entry:228 Library:NIST17-1.lib
SI:99 Formula:C2H3N3 CAS:288-36-8 MolWeight:69 RetIndex:0
CompName:1H-1,2,3-Triazole $$ V-triazole $$ Osotriazole $$ Pyrrodiazole $$ Triazacyclopentadiene $$ 1,2,3-Triazole $$

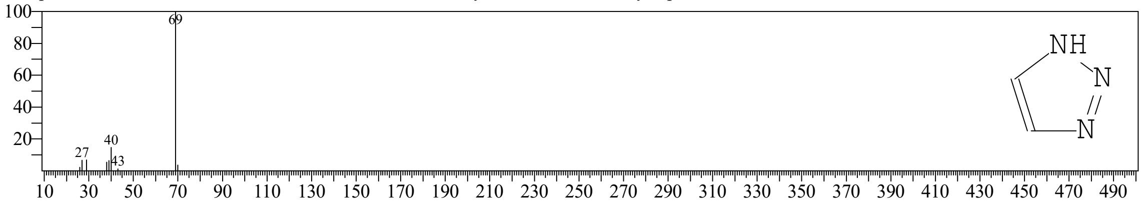

Hit#:2 Entry:981 Library:NIST17-1.lib
SI:96 Formula:CF4 CAS:75-73-0 MolWeight:88 RetIndex:0
CompName:Tetrafluoromethane $$ Carbon tetrafluoride $$ Methane, tetrafluoro- $$ Arcton 0 $$ Carbon fluoride (CF4) $$ Freon 14 $$ FC 14 $$ Perfluoro

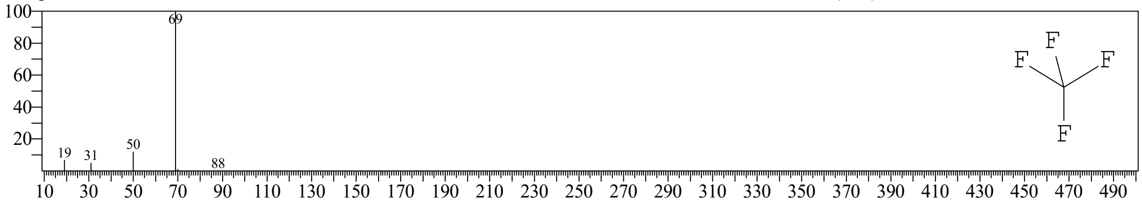

Hit#:3 Entry:169 Library:FFNSC 3.lib
SI:95 Formula:C10 H18 O CAS:106-24-1 MolWeight:154 RetIndex:1255
CompName:Geraniol

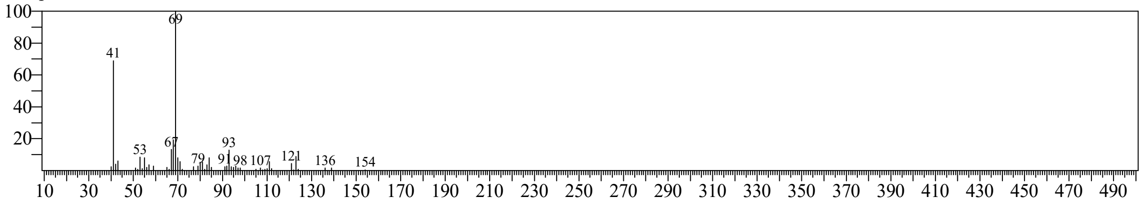

Hit#:4 Entry:10642 Library:NIST17-1.lib
SI:94 Formula:CF5P CAS:1112-04-5 MolWeight:138 RetIndex:0
CompName:Trifluoromethyldifluorophosphine $$ Trifluoromethylphosphonous difluoride # $$

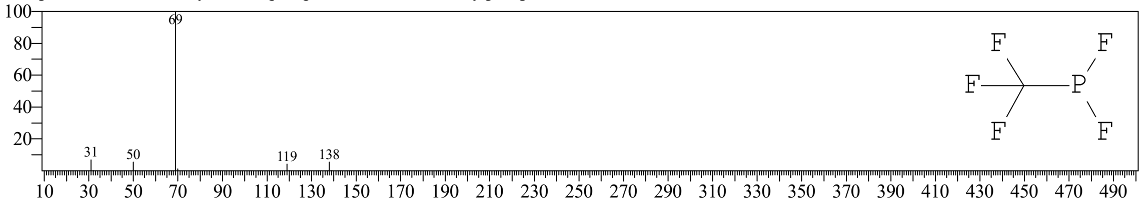

Hit#:5 Entry:13581 Library:NIST17-1.lib
SI:93 Formula:C6H12N2O2 CAS:108201-44-1 MolWeight:144 RetIndex:0
CompName:Diazene, 1-cyclopentyl-2-methoxy-, 1-oxide $$ 1-Cyclopentyl-2-methoxydiazene 1-oxide # $$

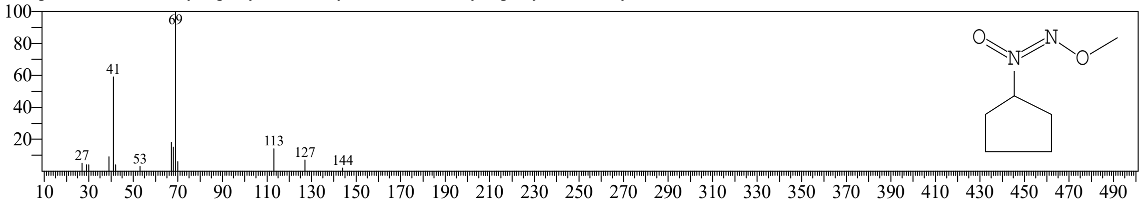

Qualitative Analysis Report

<< Target >>
Line#:38 R.Time:23.863(Scan#:4768) Retention Index:1267 MassPeaks:274
RawMode:Averaged 23.858-23.867(4767-4769) BasePeak:69.10(829874)
BG Mode:Calc. from Peak Group 1 - Event 1 Scan

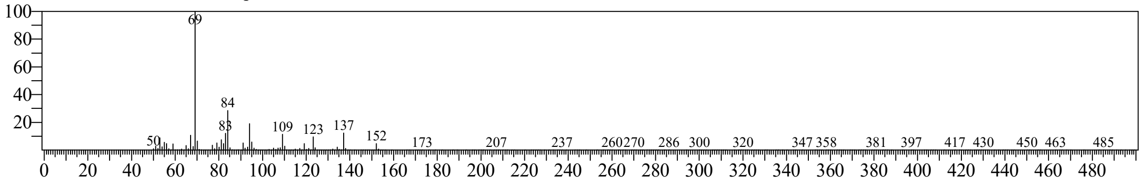

Hit#:1 Entry:228 Library:NIST17-1.lib
SI:99 Formula:C2H3N3 CAS:288-36-8 MolWeight:69 RetIndex:0
CompName:1H-1,2,3-Triazole $$ V-triazole $$ Osotriazole $$ Pyrrodiazole $$ Triazacyclopentadiene $$ 1,2,3-Triazole $$

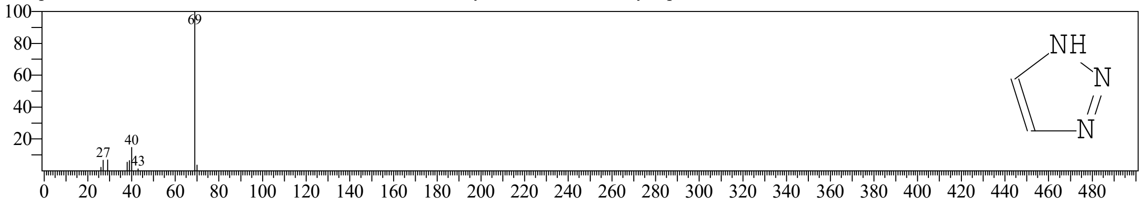

Hit#:2 Entry:10642 Library:NIST17-1.lib
SI:96 Formula:CF5P CAS:1112-04-5 MolWeight:138 RetIndex:0
CompName:Trifluoromethyldifluorophosphine $$ Trifluoromethylphosphonous difluoride # $$

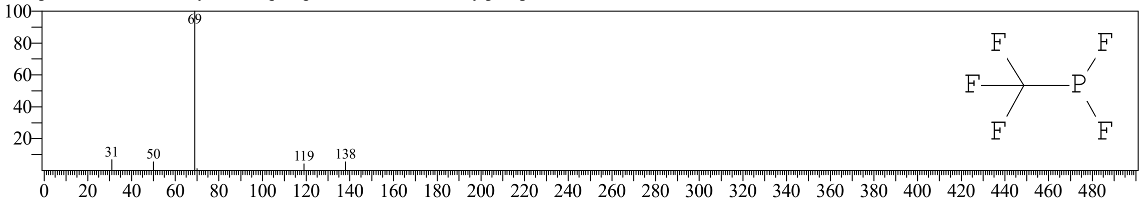

Hit#:3 Entry:981 Library:NIST17-1.lib
SI:96 Formula:CF4 CAS:75-73-0 MolWeight:88 RetIndex:0
CompName:Tetrafluoromethane $$ Carbon tetrafluoride $$ Methane, tetrafluoro- $$ Arcton 0 $$ Carbon fluoride (CF4) $$ Freon 14 $$ FC 14 $$ Perfluoro

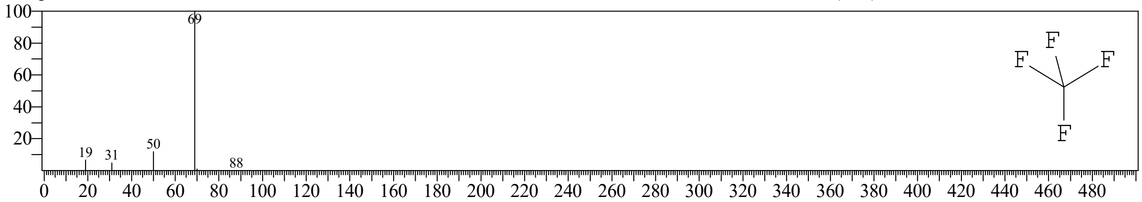

Hit#:4 Entry:1003 Library:FFNSC 3.lib
SI:95 Formula:C10 H16 O CAS:141-27-5 MolWeight:152 RetIndex:1268
CompName:Geranial

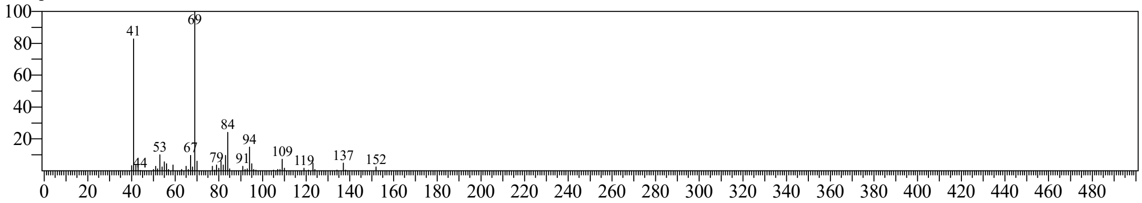

Hit#:5 Entry:1768 Library:NIST17-1.lib
SI:91 Formula:C6H15B CAS:97-94-9 MolWeight:98 RetIndex:0
CompName:Borane, triethyl- $$ Triethylborane $$ Triethylboron $$ (C2H5)3B $$ Triethylborine $$ Borethyl $$

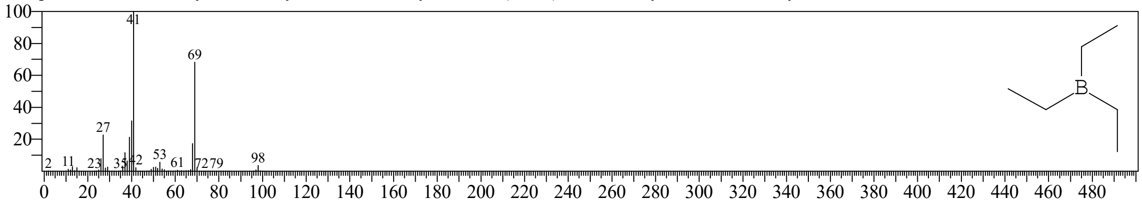

Qualitative Analysis Report

<< Target >>
Line#:39 R.Time:24.275(Scan#:4867) Retention Index:1276 MassPeaks:290
RawMode:Averaged 24.271-24.279(4866-4868) BasePeak:59.05(19964)
BG Mode:Calc. from Peak Group 1 - Event 1 Scan

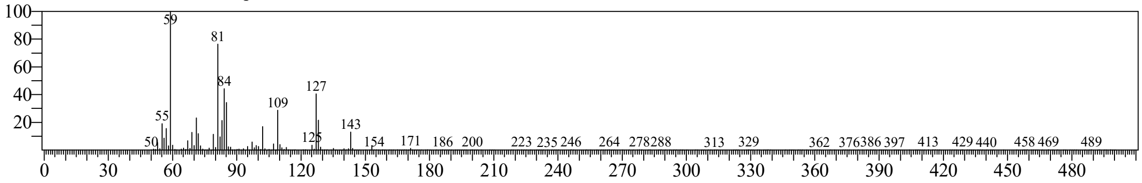

Hit#:1 Entry:27586 Library:NIST17-1.lib
SI:93 Formula:C4H12FN2PS CAS:36267-53-5 MolWeight:170 RetIndex:0
CompName:Phosphonofluoridothioic hydrazide, P-ethyl-2,2-dimethyl- $$ p-Ethyl-N',N'-dimethylphosphonohydrazidothioic fluoride # $$

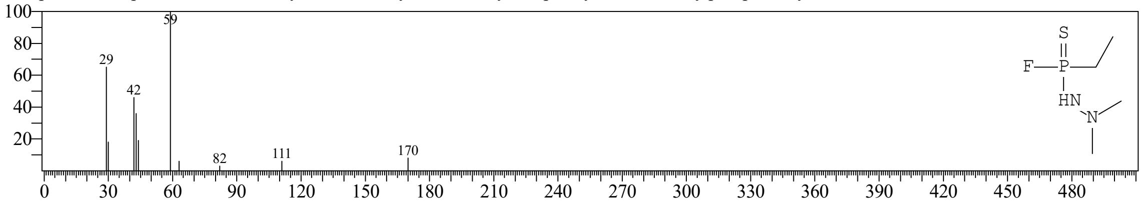

Hit#:2 Entry:120 Library:NIST17-1.lib
SI:91 Formula:CH5N3 CAS:113-00-8 MolWeight:59 RetIndex:0
CompName:Guanidine $$ Aminoformamidine $$ Aminomethanamidine $$ Carbamamidine $$ Carbamidine $$ Guanidin $$ Imidourea $$ Iminourea $$ (NH

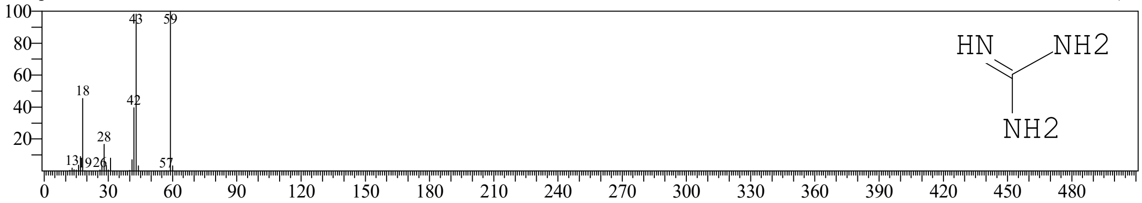

Hit#:3 Entry:144 Library:NIST17-1.lib
SI:85 Formula:C2H8Si CAS:1111-74-6 MolWeight:60 RetIndex:0
CompName:Silane, dimethyl- $$ Dimethylsilane $$ 2-Silapropane $$ (CH3)2SiH2 $$

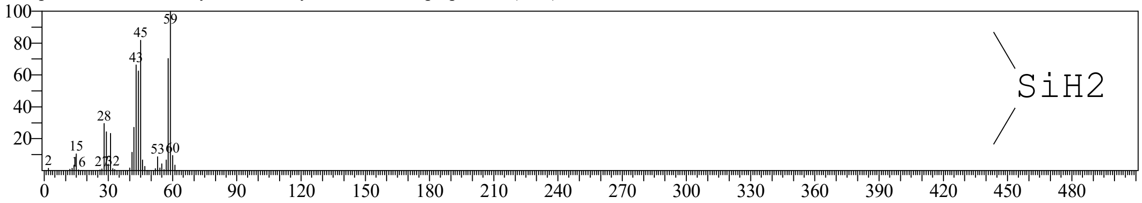

Hit#:4 Entry:29067 Library:NIST17-1.lib
SI:84 Formula:C6H8N2O4 CAS:55514-36-8 MolWeight:172 RetIndex:0
CompName:Butanedioic acid, diazo-, dimethyl ester

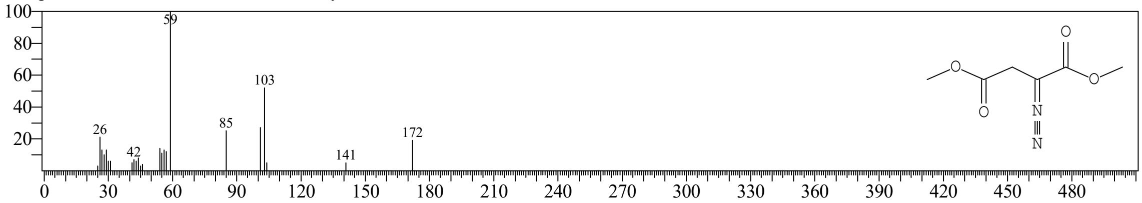

Hit#:5 Entry:13920 Library:NIST17-1.lib
SI:83 Formula:C8H20Si CAS:871-92-1 MolWeight:144 RetIndex:0
CompName:Silane, octyl- $$ Octylsilane $$ n-Octylsilane $$

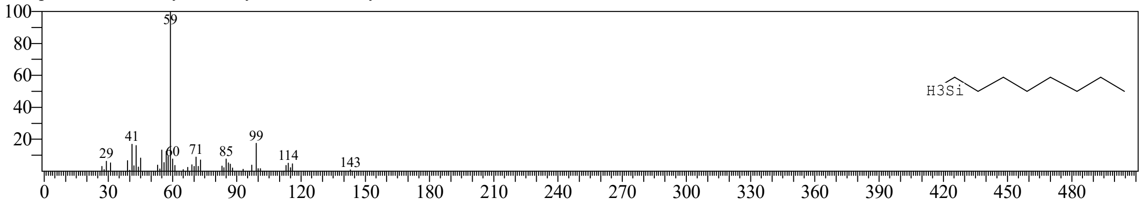

Qualitative Analysis Report

<< Target >>
Line#:40 R.Time:24.408(Scan#:4899) Retention Index:1279 MassPeaks:245
RawMode:Averaged 24.404-24.413(4898-4900) BasePeak:69.10(6778)
BG Mode:Calc. from Peak Group 1 - Event 1 Scan

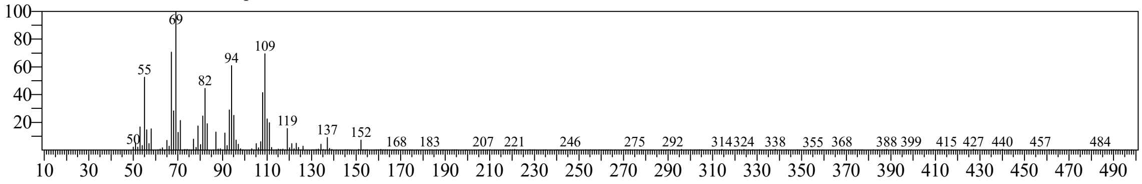

Hit#:1 Entry:228 Library:NIST17-1.lib
SI:98 Formula:C2H3N3 CAS:288-36-8 MolWeight:69 RetIndex:0
CompName:1H-1,2,3-Triazole $$ V-triazole $$ Osotriazole $$ Pyrrodiazole $$ Triazacyclopentadiene $$ 1,2,3-Triazole $$

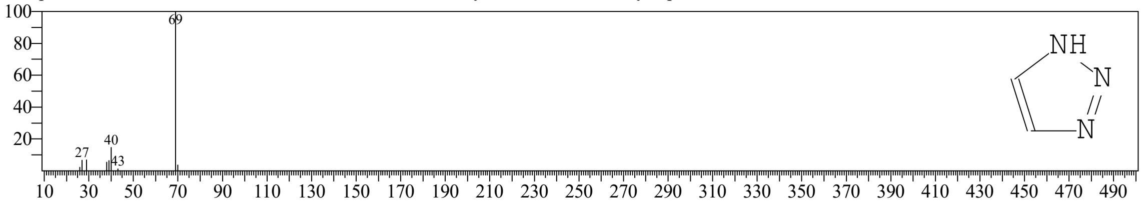

Hit#:2 Entry:981 Library:NIST17-1.lib
SI:94 Formula:CF4 CAS:75-73-0 MolWeight:88 RetIndex:0
CompName:Tetrafluoromethane $$ Carbon tetrafluoride $$ Methane, tetrafluoro- $$ Arcton 0 $$ Carbon fluoride (CF4) $$ Freon 14 $$ FC 14 $$ Perfluoro

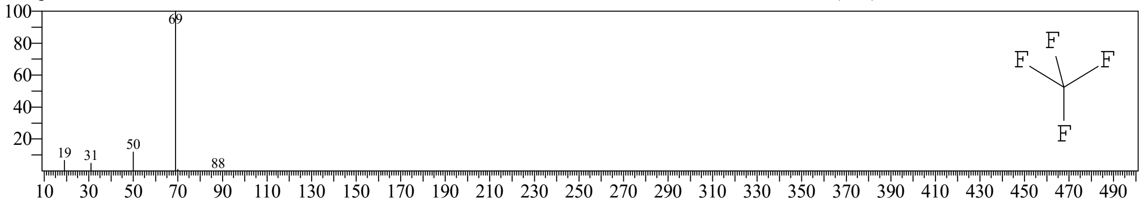

Hit#:3 Entry:73172 Library:NIST17-1.lib
SI:94 Formula:C2H6F4N2P2S CAS:27352-02-9 MolWeight:228 RetIndex:0
CompName:Phosphoramidothioic difluoride, [(dimethylamino)difluorophosphoranylidene]- $$ (Dimethylamino)(difluoro)phosphoranylidenephosphoramido

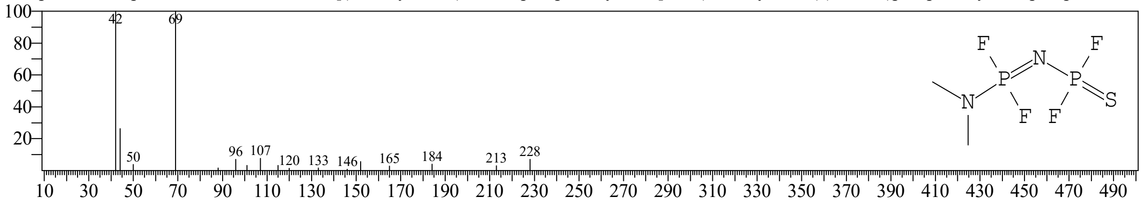

Hit#:4 Entry:10642 Library:NIST17-1.lib
SI:93 Formula:CF5P CAS:1112-04-5 MolWeight:138 RetIndex:0
CompName:Trifluoromethyldifluorophosphine $$ Trifluoromethylphosphonous difluoride # $$

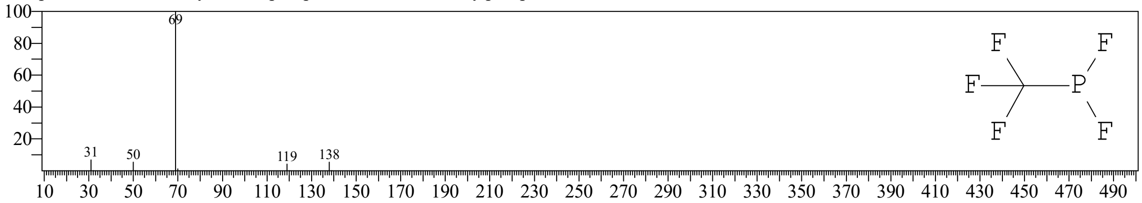

Hit#:5 Entry:1079 Library:NIST17-1.lib
SI:90 Formula:F3P CAS:7783-55-3 MolWeight:88 RetIndex:0
CompName:Phosphorus trifluoride $$ PF3 $$ Phosphorus(III) fluoride $$ Phosphorous-trifluoride- $$ Phosphorus fluoride $$ Phosphorous fluoride $$ TL

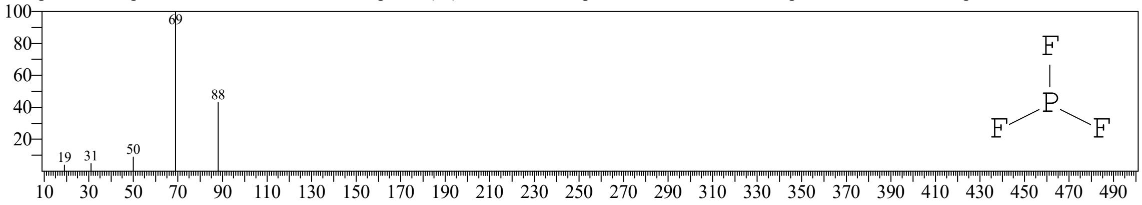

Qualitative Analysis Report

<< Target >>
Line#:41 R.Time:24.533(Scan#:4929) Retention Index:1281 MassPeaks:262
RawMode:Averaged 24.529-24.538(4928-4930) BasePeak:59.05(23755)
BG Mode:Calc. from Peak Group 1 - Event 1 Scan

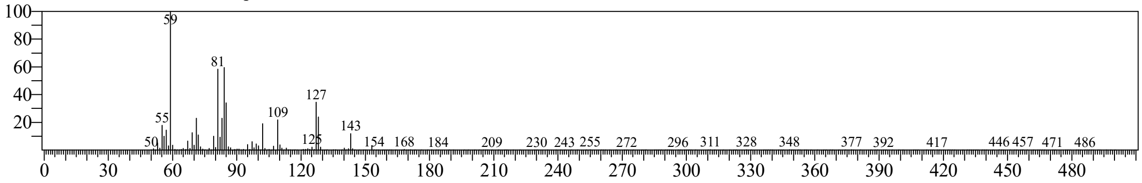

Hit#:1 Entry:27586 Library:NIST17-1.lib
SI:93 Formula:C4H12FN2PS CAS:36267-53-5 MolWeight:170 RetIndex:0
CompName:Phosphonofluoridothioic hydrazide, P-ethyl-2,2-dimethyl- $$ p-Ethyl-N',N'-dimethylphosphonohydrazidothioic fluoride # $$

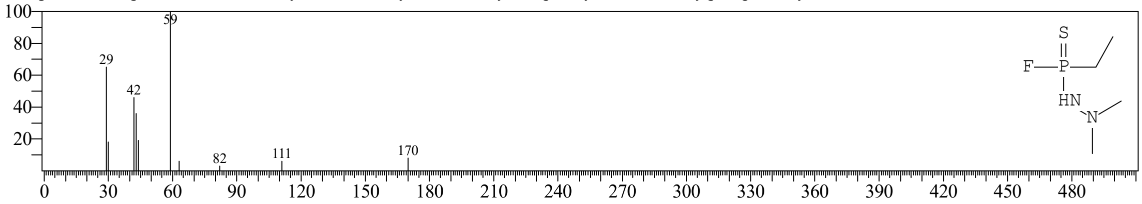

Hit#:2 Entry:120 Library:NIST17-1.lib
SI:91 Formula:CH5N3 CAS:113-00-8 MolWeight:59 RetIndex:0
CompName:Guanidine $$ Aminoformamidine $$ Aminomethanamidine $$ Carbamamidine $$ Carbamidine $$ Guanidin $$ Imidourea $$ Iminourea $$ (NI

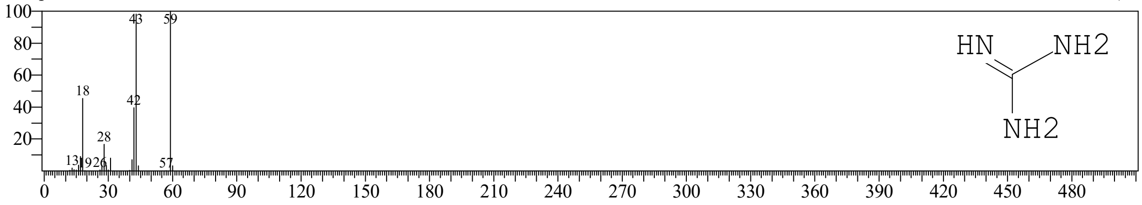

Hit#:3 Entry:21501 Library:NIST17-1.lib
SI:87 Formula:C2H7F2N2PS CAS:36267-50-2 MolWeight:160 RetIndex:0
CompName:Phosphorodifluoridothioic hydrazide, 2,2-dimethyl- $$ Difluorophosphinothioic hydrazide, 2,2-dimethyl- $$ N',N'-Dimethylphosphorohydrazid

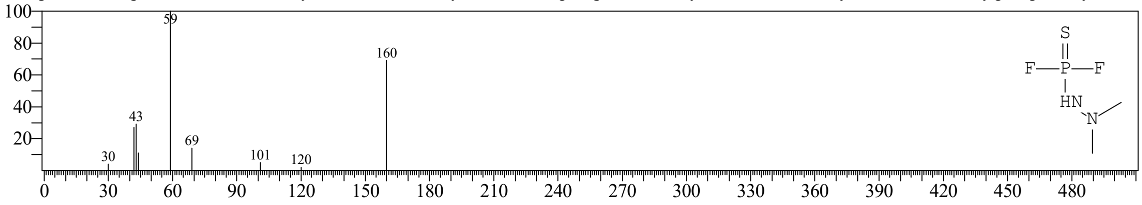

Hit#:4 Entry:144 Library:NIST17-1.lib
SI:85 Formula:C2H8Si CAS:1111-74-6 MolWeight:60 RetIndex:0
CompName:Silane, dimethyl- $$ Dimethylsilane $$ 2-Silapropane $$ (CH3)2SiH2 $$

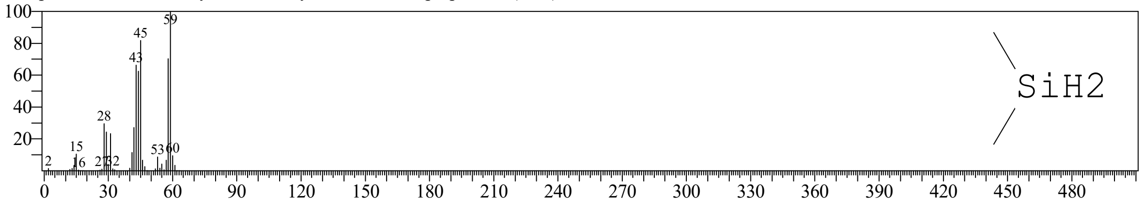

Hit#:5 Entry:29067 Library:NIST17-1.lib
SI:83 Formula:C6H8N2O4 CAS:55514-36-8 MolWeight:172 RetIndex:0
CompName:Butanedioic acid, diazo-, dimethyl ester

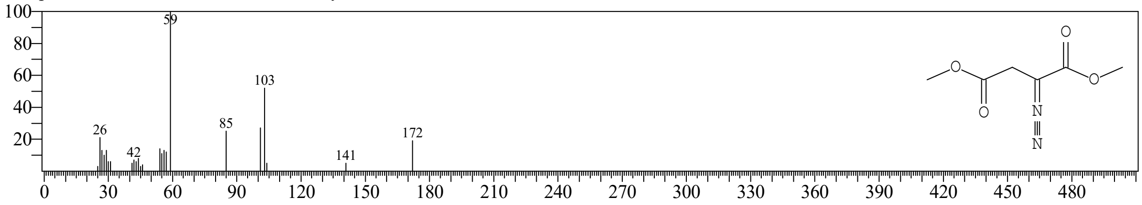

Qualitative Analysis Report

<< Target >>
Line#:42 R.Time:24.950(Scan#:5029) Retention Index:1290 MassPeaks:277
RawMode:Averaged 24.946-24.954(5028-5030) BasePeak:58.05(54821)
BG Mode:Calc. from Peak Group 1 - Event 1 Scan

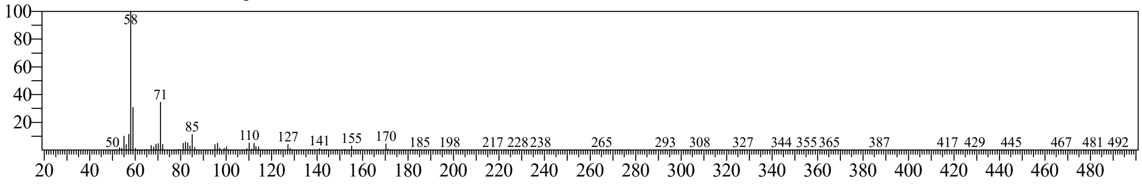

Hit#:1 Entry:843 Library:FFNSC 3.lib
SI:97 Formula:C11 H22 O CAS:112-12-9 MolWeight:170 RetIndex:1294
CompName:Undecan-2-one

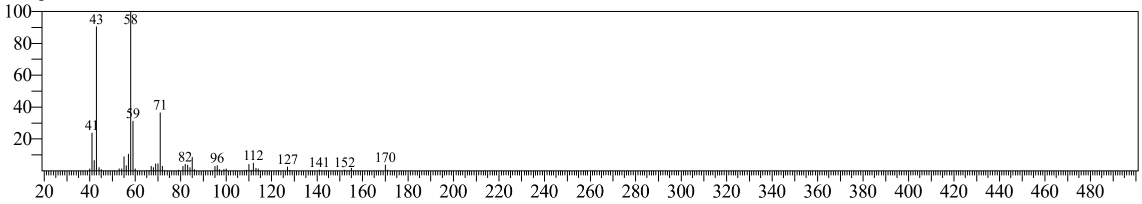

Hit#:2 Entry:143 Library:NIST17-1.lib
SI:92 Formula:C2H8Si CAS:2814-79-1 MolWeight:60 RetIndex:0
CompName:Silane, ethyl- $$ Ethylsilane $$ 1-Silapropane $$ C2H5SiH3 $$

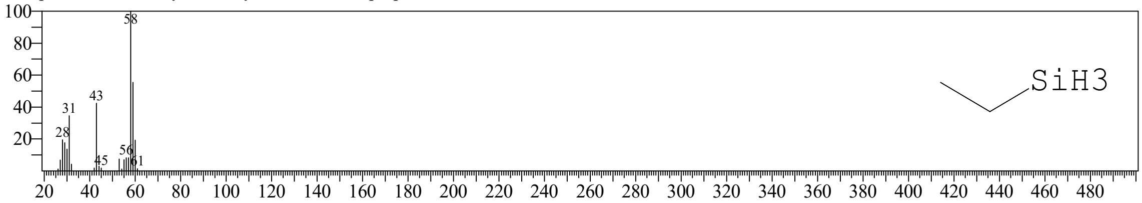

Hit#:3 Entry:805 Library:NIST17-1.lib
SI:92 Formula:C2H2N2S CAS:288-48-2 MolWeight:86 RetIndex:0
CompName:1,2,3-Thiadiazole

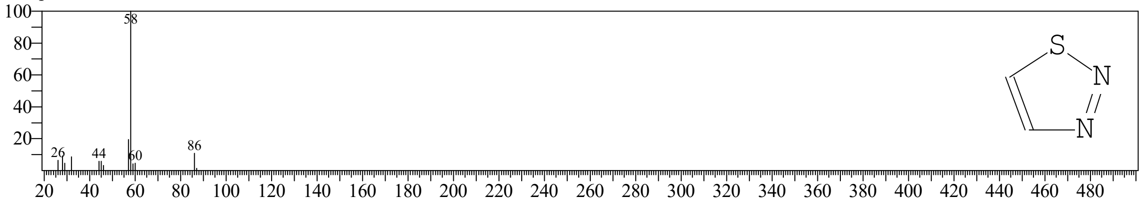

Hit#:4 Entry:154742 Library:NIST17-1.lib
SI:91 Formula:C19H21NOS CAS:1447-71-8 MolWeight:311 RetIndex:0
CompName:1-Propanamine, 3-dibenzo[b,e]thiepin-11(6H)-ylidene-N,N-dimethyl-, S-oxide $$ Dothiepin sulfoxide $$ (3E)-N,N-Dimethyl-3-(5-oxidodibenz

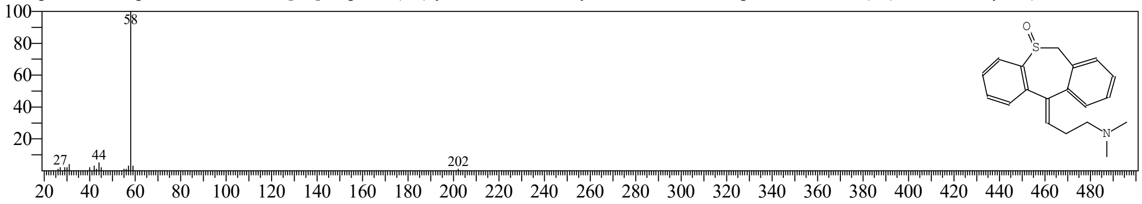

Hit#:5 Entry:70732 Library:NIST17-1.lib
SI:90 Formula:C8H20NO2PS CAS:98543-25-0 MolWeight:225 RetIndex:0
CompName:O-Ethyl S-2-dimethylaminoethyl ethylphosphonothiolate $$ o-Ethyl S-2-(dimethylamino)ethyl ethylphosphonothiolate $$ S-[2-(Dimethylamino)

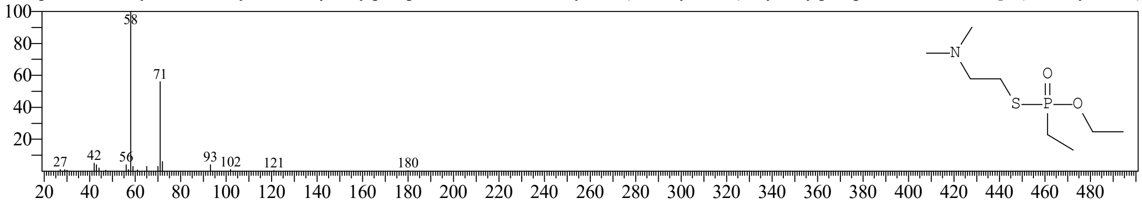

Qualitative Analysis Report

<< Target >>
Line#:43 R.Time:25.196(Scan#:5088) Retention Index:1296 MassPeaks:226
RawMode:Averaged 25.192-25.200(5087-5089) BasePeak:69.10(4767)
BG Mode:Calc. from Peak Group 1 - Event 1 Scan

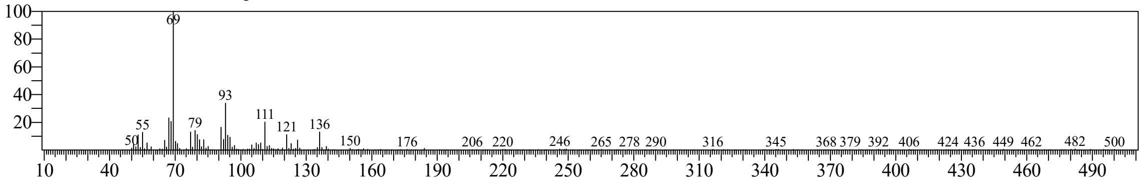

Hit#:1 Entry:228 Library:NIST17-1.lib
SI:99 Formula:C2H3N3 CAS:288-36-8 MolWeight:69 RetIndex:0
CompName:1H-1,2,3-Triazole $$ V-triazole $$ Osotriazole $$ Pyrrodiazole $$ Triazacyclopentadiene $$ 1,2,3-Triazole $$

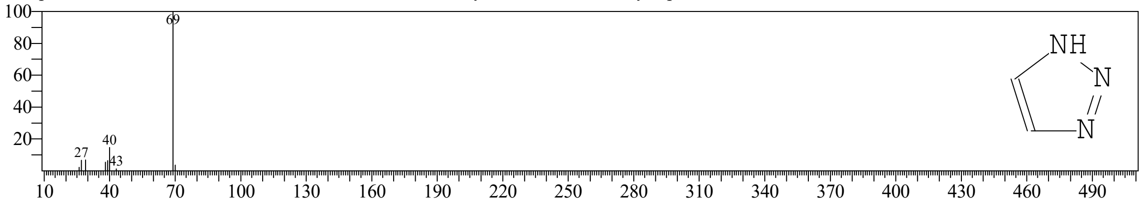

Hit#:2 Entry:981 Library:NIST17-1.lib
SI:96 Formula:CF4 CAS:75-73-0 MolWeight:88 RetIndex:0
CompName:Tetrafluoromethane $$ Carbon tetrafluoride $$ Methane, tetrafluoro- $$ Arcton 0 $$ Carbon fluoride (CF4) $$ Freon 14 $$ FC 14 $$ Perfluoro

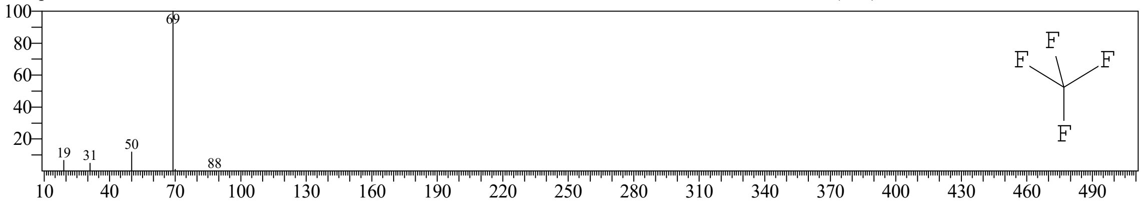

Hit#:3 Entry:10642 Library:NIST17-1.lib
SI:95 Formula:CF5P CAS:1112-04-5 MolWeight:138 RetIndex:0
CompName:Trifluoromethyldifluorophosphine $$ Trifluoromethylphosphonous difluoride # $$

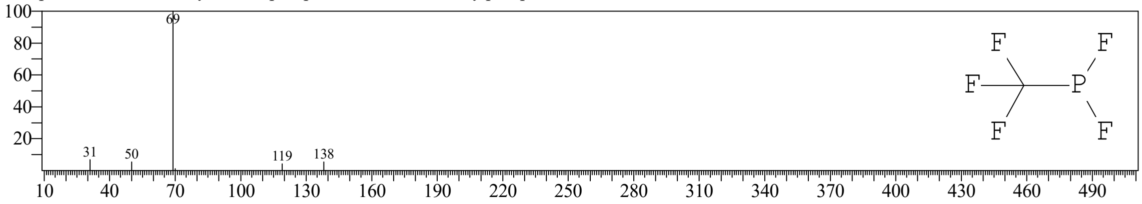

Hit#:4 Entry:10455 Library:NIST17-1.lib
SI:94 Formula:CClF4N CAS:13880-72-3 MolWeight:137 RetIndex:0
CompName:N-Chloro-N-fluoro-trifluoromethylamine $$ N-Chloro-N-tetrafluoromethanamine # $$

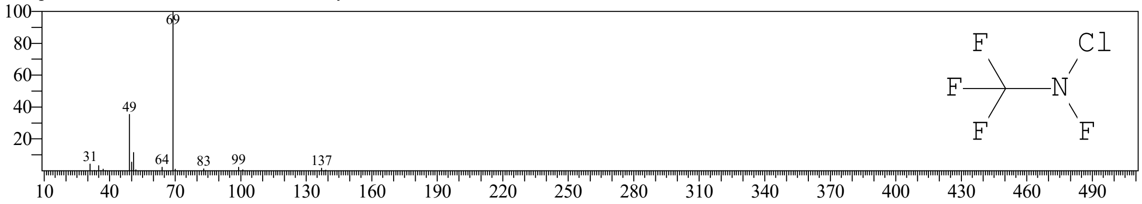

Hit#:5 Entry:13581 Library:NIST17-1.lib
SI:94 Formula:C6H12N2O2 CAS:108201-44-1 MolWeight:144 RetIndex:0
CompName:Diazene, 1-cyclopentyl-2-methoxy-, 1-oxide $$ 1-Cyclopentyl-2-methoxydiazene 1-oxide # $$

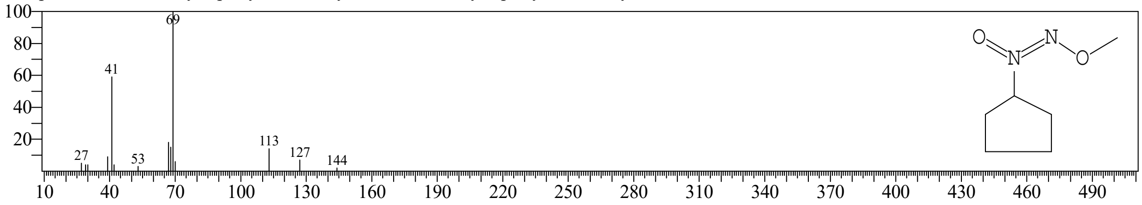

Qualitative Analysis Report

<< Target >>
Line#:44 R.Time:25.958(Scan#:5271) Retention Index:1313 MassPeaks:289
RawMode:Averaged 25.954-25.962(5270-5272) BasePeak:69.10(4022)
BG Mode:Calc. from Peak Group 1 - Event 1 Scan

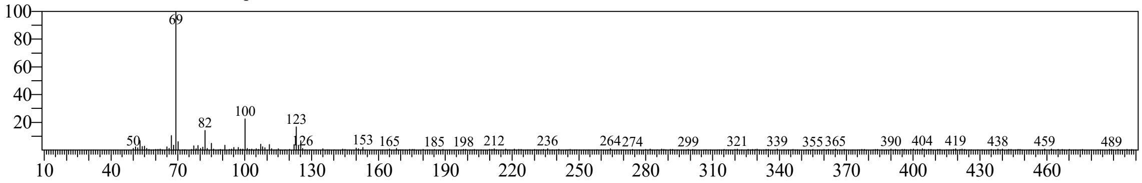

Hit#:1 Entry:228 Library:NIST17-1.lib
SI:99 Formula:C2H3N3 CAS:288-36-8 MolWeight:69 RetIndex:0
CompName:1H-1,2,3-Triazole $$ V-triazole $$ Osotriazole $$ Pyrrodiazole $$ Triazacyclopentadiene $$ 1,2,3-Triazole $$

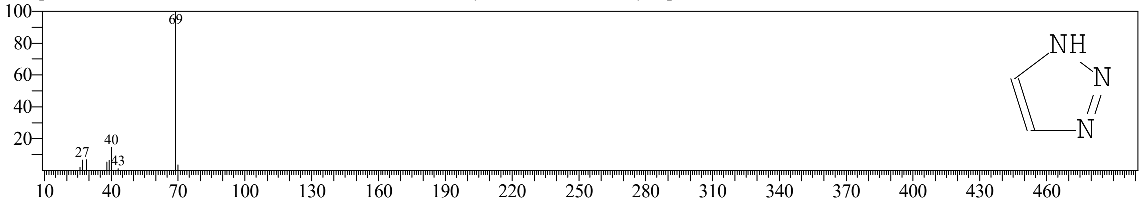

Hit#:2 Entry:981 Library:NIST17-1.lib
SI:96 Formula:CF4 CAS:75-73-0 MolWeight:88 RetIndex:0
CompName:Tetrafluoromethane $$ Carbon tetrafluoride $$ Methane, tetrafluoro- $$ Arcton 0 $$ Carbon fluoride (CF4) $$ Freon 14 $$ FC 14 $$ Perfluoro

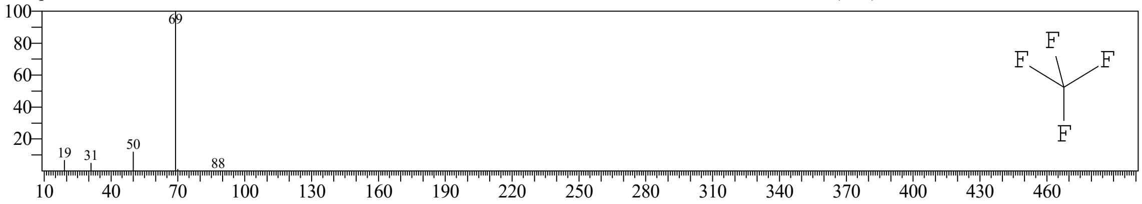

Hit#:3 Entry:2085 Library:FFNSC 3.lib
SI:95 Formula:C10 H16 O2 CAS:4613-38-1 MolWeight:168 RetIndex:1320
CompName:Neranic acid

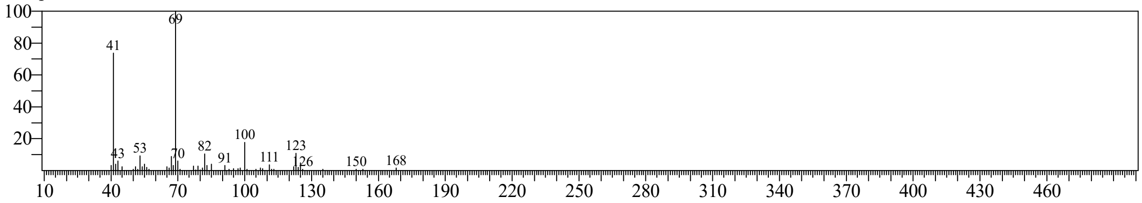

Hit#:4 Entry:10642 Library:NIST17-1.lib
SI:94 Formula:CF5P CAS:1112-04-5 MolWeight:138 RetIndex:0
CompName:Trifluoromethyldifluorophosphine $$ Trifluoromethylphosphonous difluoride # $$

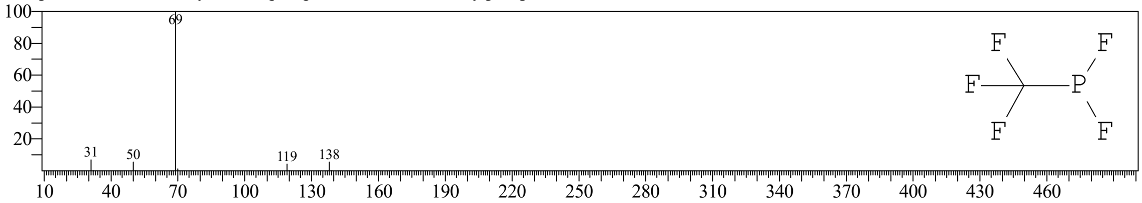

Hit#:5 Entry:10455 Library:NIST17-1.lib
SI:94 Formula:CClF4N CAS:13880-72-3 MolWeight:137 RetIndex:0
CompName:N-Chloro-N-fluoro-trifluoromethylamine $$ N-Chloro-N-tetrafluoromethanamine # $$

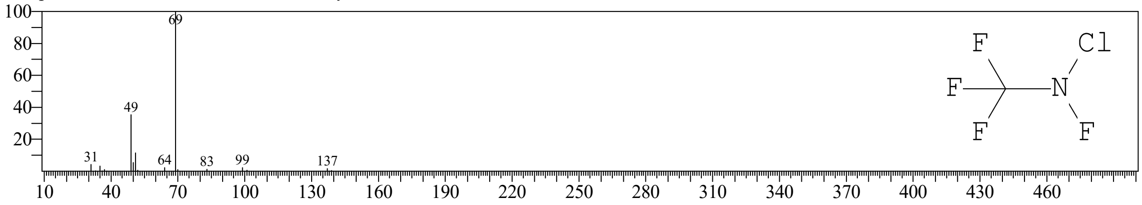

Qualitative Analysis Report

<< Target >>
Line#:45 R.Time:26.767(Scan#:5465) Retention Index:1331 MassPeaks:284
RawMode:Averaged 26.762-26.771(5464-5466) BasePeak:82.05(12366)
BG Mode:Calc. from Peak Group 1 - Event 1 Scan

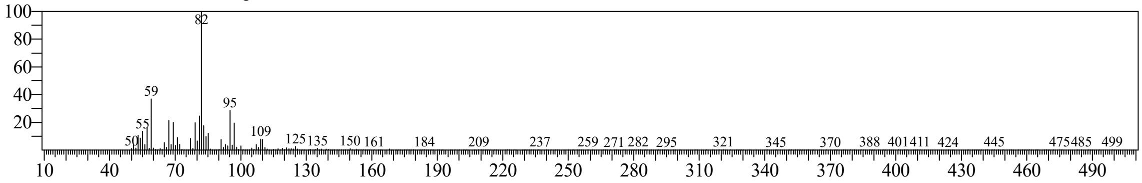

Hit#:1 Entry:3497 Library:NIST17-1.lib
SI:93 Formula:C2H6FO2P CAS:353-88-8 MolWeight:112 RetIndex:0
CompName:Methyl methylphosphonofluoridate $$ Phosphonofluoridic acid, methyl-, methyl ester $$ Methylphoshonic acid, fluoroanhydride, methyl ester $

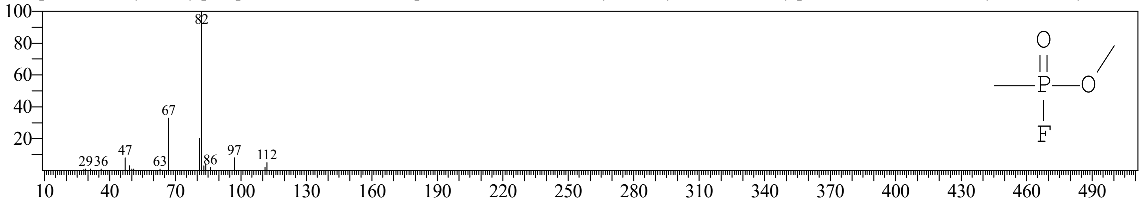

Hit#:2 Entry:18025 Library:NIST17-1.lib
SI:86 Formula:C9H20BN CAS:30430-68-3 MolWeight:153 RetIndex:0
CompName:2-Butyl-1-ethyl-1,2-azaborolidine

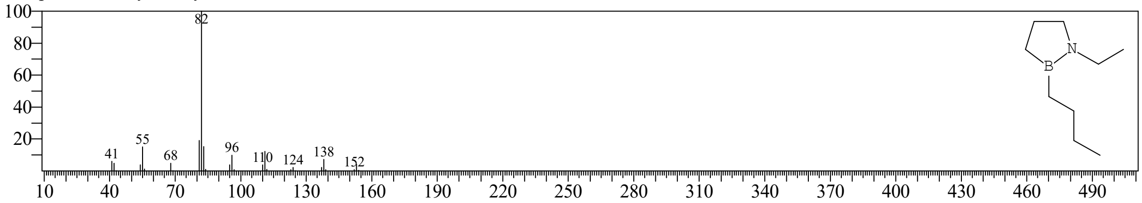

Hit#:3 Entry:555 Library:NIST17-1.lib
SI:85 Formula:CClFO CAS:353-49-1 MolWeight:82 RetIndex:0
CompName:Carbonic chloride fluoride $$ Carbonyl chloride fluoride $$ COClF $$ Carbonyl chlorofluoride $$ Estra-1,3,5(10), 7-tetraen-17-one, 3-hydroxy

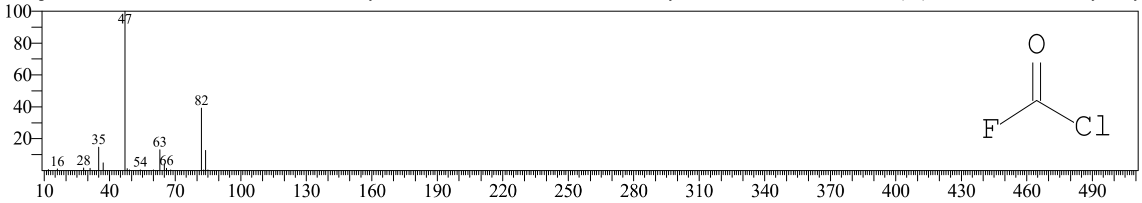

Hit#:4 Entry:556 Library:NIST17-1.lib
SI:85 Formula:CH3FOS CAS:56755-41-0 MolWeight:82 RetIndex:0
CompName:Methanesulfinyl fluoride

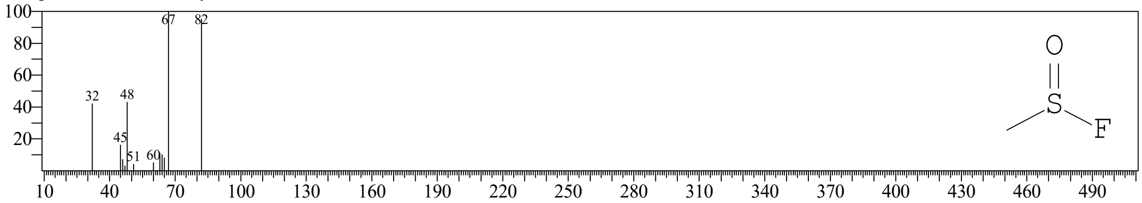

Hit#:5 Entry:173912 Library:NIST17-1.lib
SI:84 Formula:C18H33B3O3 CAS:28289-86-3 MolWeight:330 RetIndex:0
CompName:Boroxin, tricyclohexyl- $$ 2,4,6-Tricyclohexylboroxin # $$

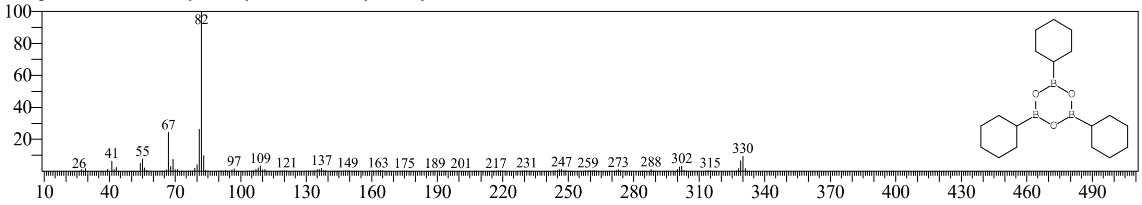

Qualitative Analysis Report

<< Target >>
Line#:46 R.Time:27.458(Scan#:5631) Retention Index:1347 MassPeaks:292
RawMode:Averaged 27.454-27.462(5630-5632) BasePeak:69.10(18214)
BG Mode:Calc. from Peak Group 1 - Event 1 Scan

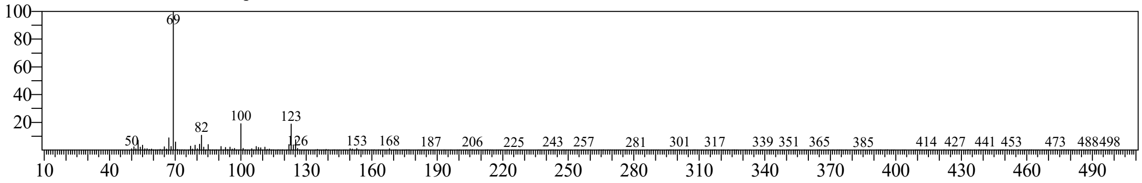

Hit#:1 Entry:228 Library:NIST17-1.lib
SI:100 Formula:C2H3N3 CAS:288-36-8 MolWeight:69 RetIndex:0
CompName:1H-1,2,3-Triazole $$ V-triazole $$ Osotriazole $$ Pyrrodiazole $$ Triazacyclopentadiene $$ 1,2,3-Triazole $$

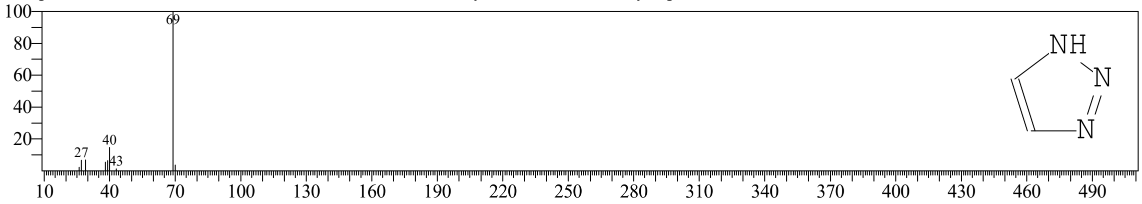

Hit#:2 Entry:26680 Library:NIST17-1.lib
SI:98 Formula:C10H16O2 CAS:459-80-3 MolWeight:168 RetIndex:1342
CompName:Geranic acid $$ 2,6-Octadienoic acid, 3,7-dimethyl- $$ 3,7-Dimethyl-2,6-octadienoic acid $$ (2E)-3,7-Dimethyl-2,6-octadienoic acid # $$

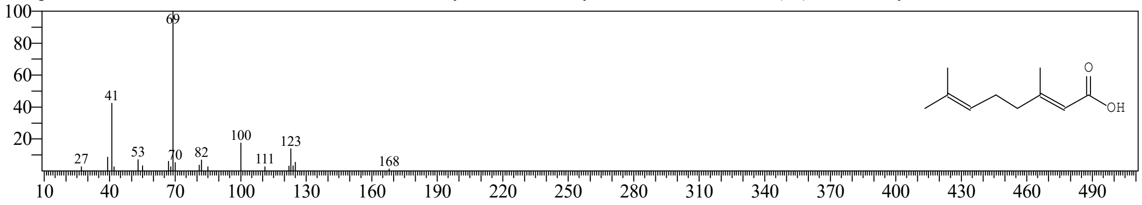

Hit#:3 Entry:981 Library:NIST17-1.lib
SI:96 Formula:CF4 CAS:75-73-0 MolWeight:88 RetIndex:0
CompName:Tetrafluoromethane $$ Carbon tetrafluoride $$ Methane, tetrafluoro- $$ Arcton 0 $$ Carbon fluoride (CF4) $$ Freon 14 $$ FC 14 $$ Perfluoro

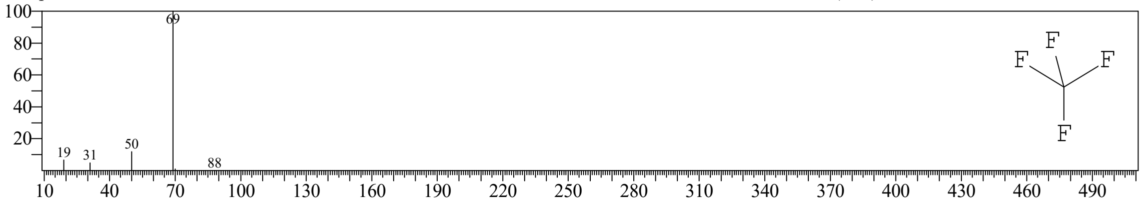

Hit#:4 Entry:10642 Library:NIST17-1.lib
SI:95 Formula:CF5P CAS:1112-04-5 MolWeight:138 RetIndex:0
CompName:Trifluoromethyldifluorophosphine $$ Trifluoromethylphosphonous difluoride # $$

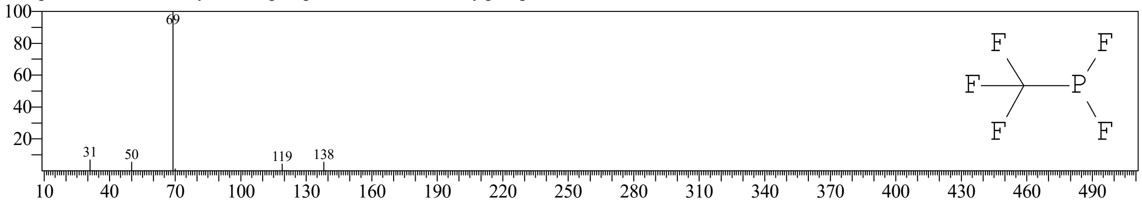

Hit#:5 Entry:26681 Library:NIST17-1.lib
SI:95 Formula:C10H16O2 CAS:4698-08-2 MolWeight:168 RetIndex:1342
CompName:2,6-Octadienoic acid, 3,7-dimethyl-, (E)-

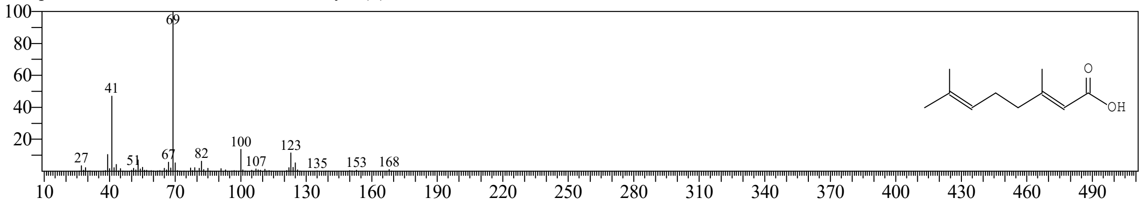

Qualitative Analysis Report

<< Target >>
Line#:47 R.Time:28.346(Scan#:5844) Retention Index:1367 MassPeaks:244
RawMode:Averaged 28.342-28.350(5843-5845) BasePeak:81.10(12622)
BG Mode:Calc. from Peak Group 1 - Event 1 Scan

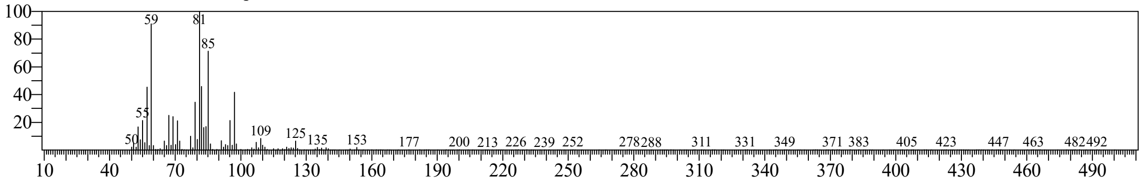

Hit#:1 Entry:134876 Library:NIST17-1.lib
SI:86 Formula:C13H16N4O2S CAS:120675-04-9 MolWeight:292 RetIndex:0
CompName:1-(Phenylsulfonyamino)-5-methyl-4,5,6,7(1H)-tetrahydrobenzo(d)triazole $$ N-(5-Methyl-4,5,6,7-tetrahydro-1H-1,2,3-benzotriazol-1-yl)benze

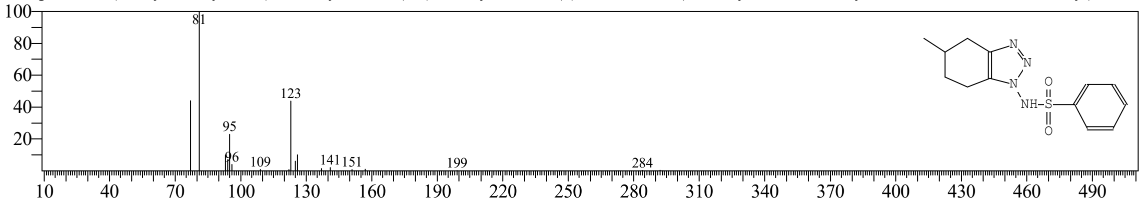

Hit#:2 Entry:3768 Library:NIST17-1.lib
SI:85 Formula:C7H12O CAS:4058-52-0 MolWeight:112 RetIndex:0
CompName:3,4-Pentadien-1-ol, 2,2-dimethyl- $$ 2,2-Dimethyl-3,4-pentadien-1-ol # $$

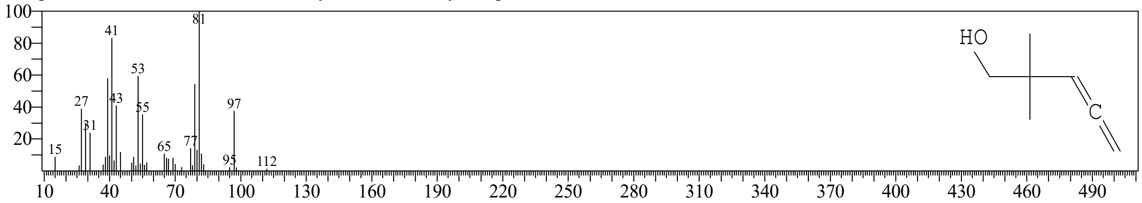

Hit#:3 Entry:158522 Library:NIST17-1.lib
SI:85 Formula:C11H5N7O5 CAS:0-00-0 MolWeight:315 RetIndex:0
CompName:12-(Furan-2-ylmethyl)-4,9,15-trioxa-3,5,8,10,12,14,16-heptaazatetracyclo[11.3.0.0(2,6).0(7,11)]hexadeca-1(16),2,5,7,10,13-hexaen-3-one

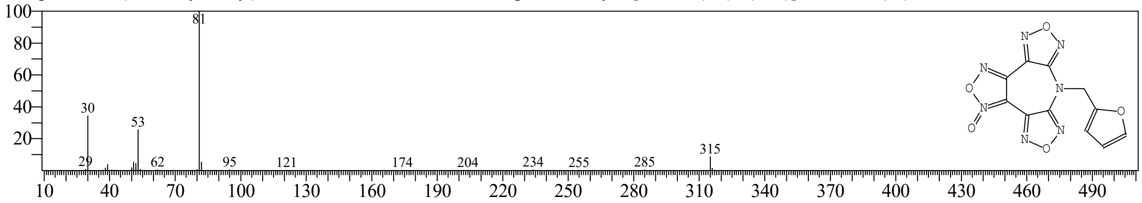

Hit#:4 Entry:105498 Library:NIST17-1.lib
SI:83 Formula:C15H22N2O2 CAS:17901-48-3 MolWeight:262 RetIndex:0
CompName:RMDI, Cis,trans-

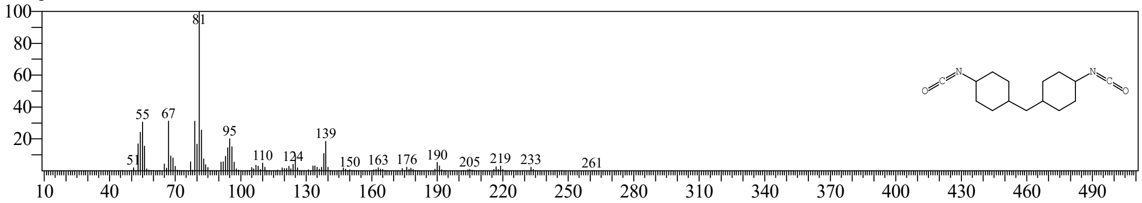

Hit#:5 Entry:105497 Library:NIST17-1.lib
SI:83 Formula:C15H22N2O2 CAS:18937-00-3 MolWeight:262 RetIndex:0
CompName:RMDI, cis,cis-

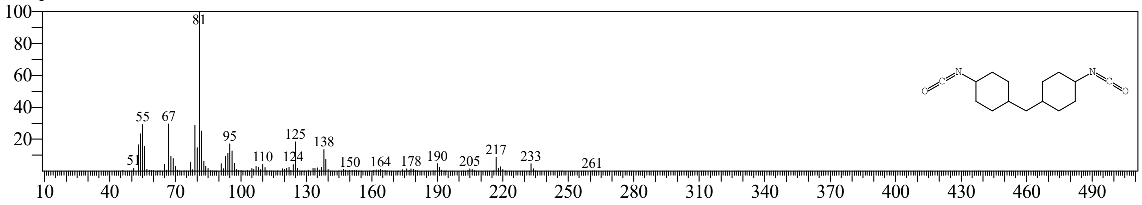

Qualitative Analysis Report

<< Target >>
Line#:48 R.Time:30.992(Scan#:6479) Retention Index:1429 MassPeaks:253
RawMode:Averaged 30.988-30.996(6478-6480) BasePeak:83.05(4390)
BG Mode:Calc. from Peak Group 1 - Event 1 Scan

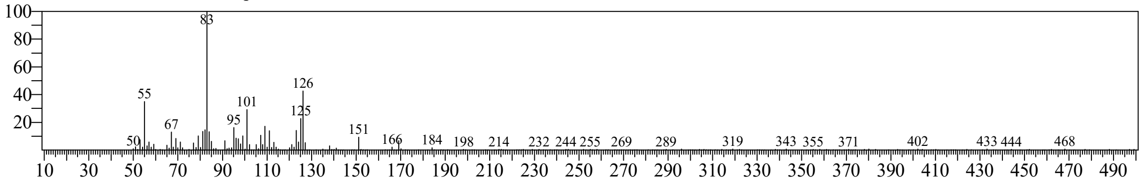

Hit#:1 Entry:3389 Library:NIST17-1.lib
SI:91 Formula:C3HN3S CAS:57352-02-0 MolWeight:111 RetIndex:0
CompName:5-Cyano-1,2,3-thiadiazole $$ 1,2,3-Thiadiazole-5-carbonitrile # $$

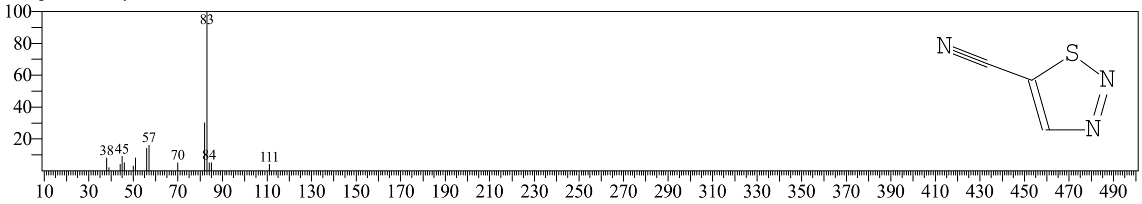

Hit#:2 Entry:181315 Library:NIST17-1.lib
SI:89 Formula:C18H30N2O4 CAS:66737-12-0 MolWeight:338 RetIndex:0
CompName:N,N'-Bis(2,6-dimethyl-6-nitrosohept-2-en-4-one)

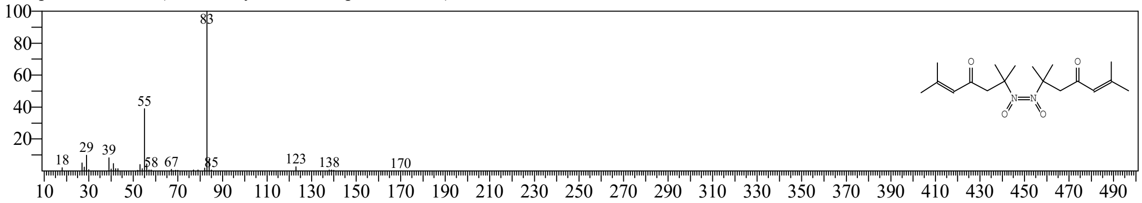

Hit#:3 Entry:29298 Library:NIST17-1.lib
SI:89 Formula:C8H16N2O2 CAS:108201-46-3 MolWeight:172 RetIndex:0
CompName:Diazene, 1-cyclohexyl-2-ethoxy-, 1-oxide $$ Cyclohexyl(ethoxy)diazene oxide # $$

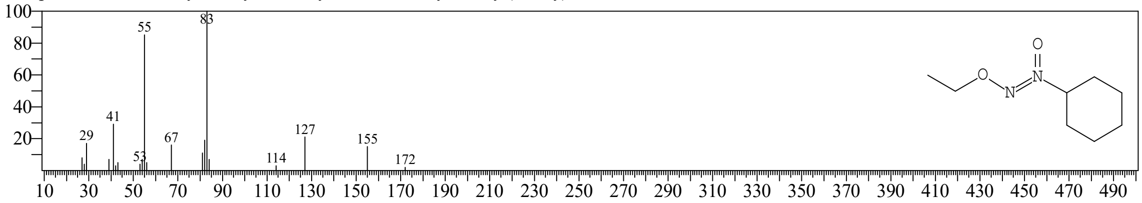

Hit#:4 Entry:47746 Library:NIST17-1.lib
SI:87 Formula:C10H14O4 CAS:69511-49-5 MolWeight:198 RetIndex:1428
CompName:2-Butynoic acid, 4-[(tetrahydro-2H-pyran-2-yl)oxy]-, methyl ester $$ Methyl 4-(tetrahydro-2H-pyran-2-yloxy)-2-butynoate # $$

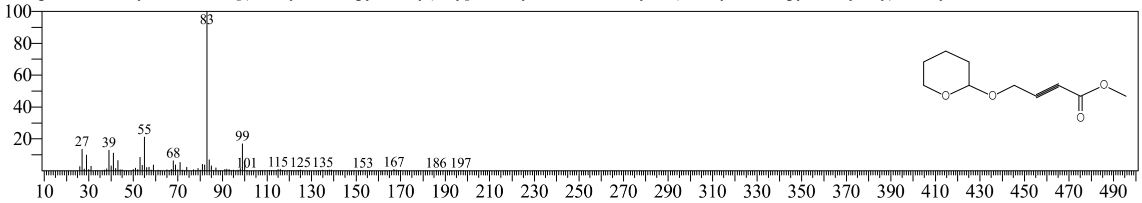

Hit#:5 Entry:36510 Library:NIST17-1.lib
SI:86 Formula:Cl2O7 CAS:12015-53-1 MolWeight:182 RetIndex:0
CompName:Dichlorine heptoxide $$ Chlorine oxide (Cl2O7) $$ Chlorine heptoxide $$ Perchloric anhydride $$ (Perchloryloxy)chlorane trioxide # $$

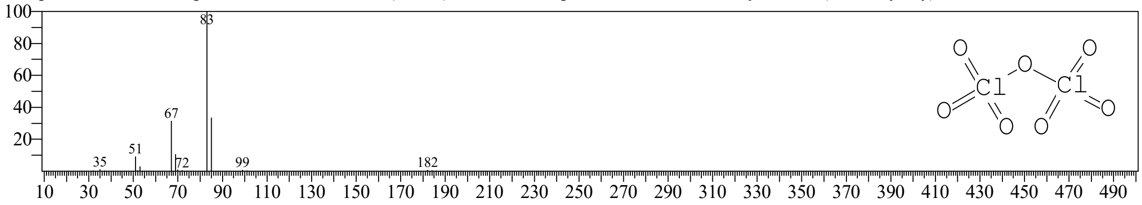

Qualitative Analysis Report

<< Target >>
Line#:49 R.Time:33.446(Scan#:7068) Retention Index:1487 MassPeaks:235
RawMode:Averaged 33.442-33.450(7067-7069) BasePeak:71.10(2186)
BG Mode:Calc. from Peak Group 1 - Event 1 Scan

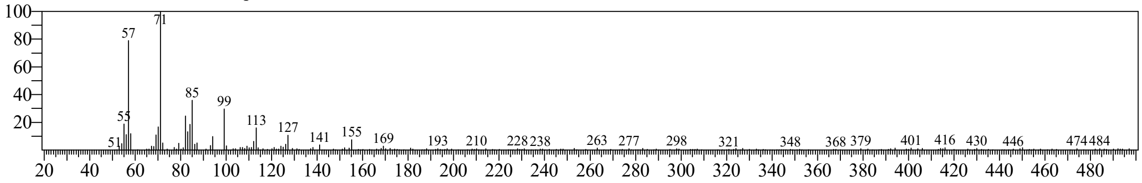

Hit#:1 Entry:19553 Library:NIST17-1.lib
SI:87 Formula:C7H12N2O2 CAS:26537-51-9 MolWeight:156 RetIndex:0
CompName:Sydnone, 3-isopentyl- $$ 3-Isopentyl-1,2,3-oxadiazol-3-ium-5-olate # $$

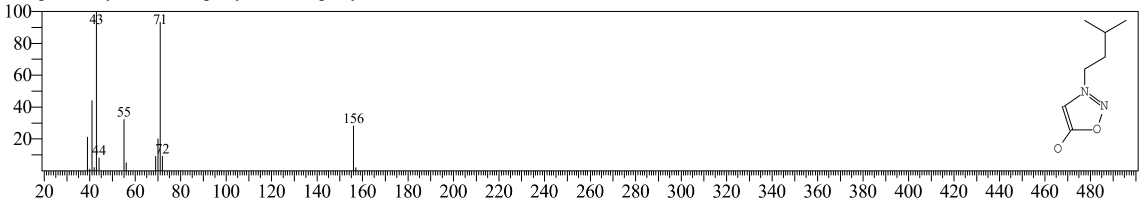

Hit#:2 Entry:72531 Library:NIST17-1.lib
SI:87 Formula:C16H34 CAS:55045-12-0 MolWeight:226 RetIndex:1483
CompName:Tetradecane, 4,11-dimethyl- $$ 4,11-Dimethyltetradecane # $$

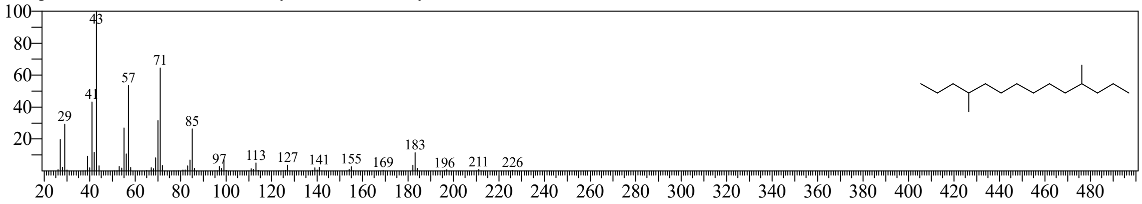

Hit#:3 Entry:75401 Library:NIST17-1.lib
SI:85 Formula:C12H22O4 CAS:0-00-0 MolWeight:230 RetIndex:1485
CompName:Oxalic acid, 2-ethylhexyl ethyl ester

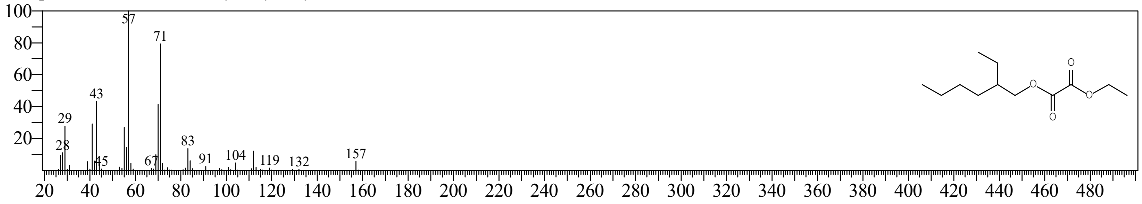

Hit#:4 Entry:72549 Library:NIST17-1.lib
SI:85 Formula:C16H34 CAS:119209-20-0 MolWeight:226 RetIndex:1483
CompName:Decane, 5,6-dipropyl- $$ 5,6-Dipropyldecane # $$

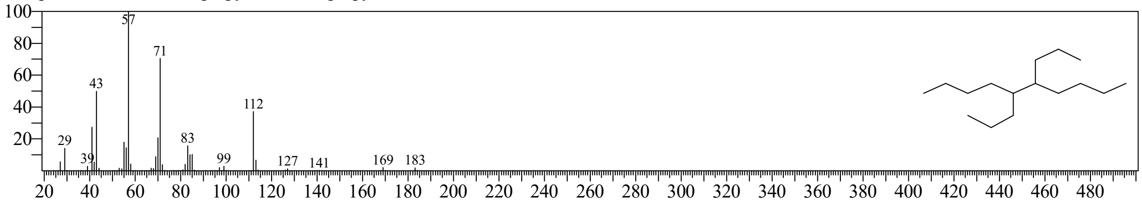

Hit#:5 Entry:72540 Library:NIST17-1.lib
SI:85 Formula:C16H34 CAS:55045-07-3 MolWeight:226 RetIndex:1483
CompName:Dodecane, 2-methyl-8-propyl- $$ 2-Methyl-8-propyldodecane # $$

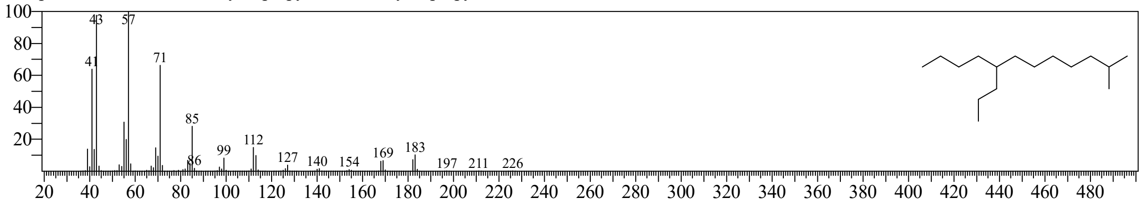

Qualitative Analysis Report

<< Target >>
Line#:50 R.Time:33.650(Scan#:7117) Retention Index:1492 MassPeaks:283
RawMode:Averaged 33.646-33.654(7116-7118) BasePeak:58.05(40024)
BG Mode:Calc. from Peak Group 1 - Event 1 Scan

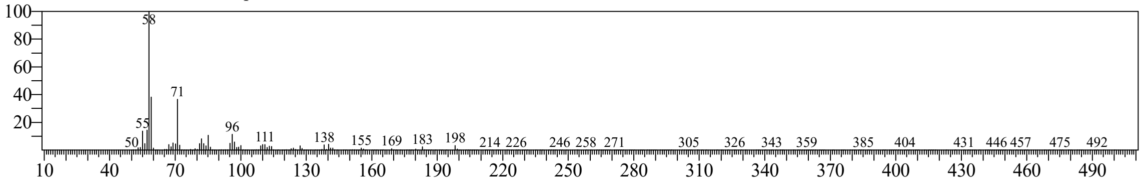

Hit#:1 Entry:852 Library:FFNSC 3.lib
SI:97 Formula:C13 H26 O CAS:593-08-8 MolWeight:198 RetIndex:1495
CompName:Tridecan-2-one

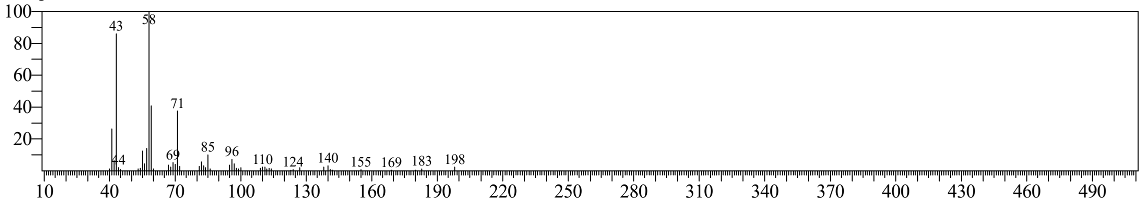

Hit#:2 Entry:143 Library:NIST17-1.lib
SI:92 Formula:C2H8Si CAS:2814-79-1 MolWeight:60 RetIndex:0
CompName:Silane, ethyl- $$ Ethylsilane $$ 1-Silapropane $$ C2H5SiH3 $$

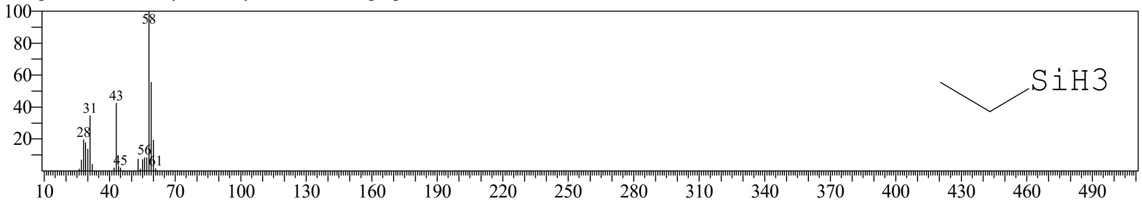

Hit#:3 Entry:805 Library:NIST17-1.lib
SI:91 Formula:C2H2N2S CAS:288-48-2 MolWeight:86 RetIndex:0
CompName:1,2,3-Thiadiazole

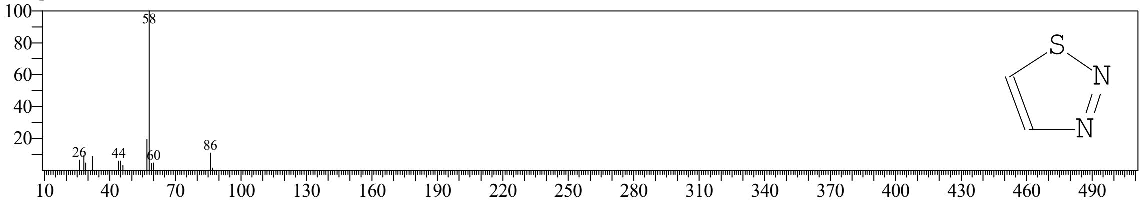

Hit#:4 Entry:9197 Library:NIST17-1.lib
SI:90 Formula:C6H15NO2 CAS:10581-12-1 MolWeight:133 RetIndex:0
CompName:Tetramethylammonium acetate $$ Methanaminium, N,N,N-trimethyl-, acetate $$ Ammonium, tetramethyl-, acetate $$

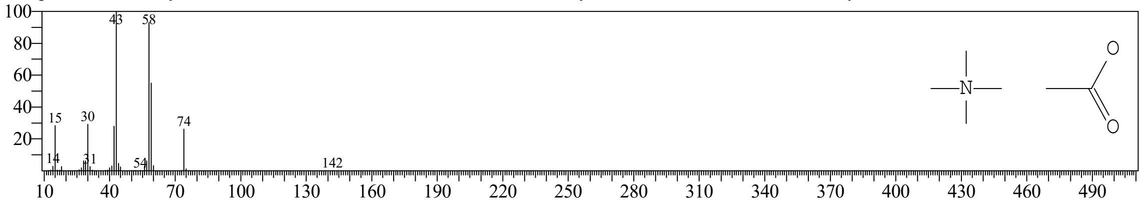

Hit#:5 Entry:70732 Library:NIST17-1.lib
SI:90 Formula:C8H20NO2PS CAS:98543-25-0 MolWeight:225 RetIndex:0
CompName:O-Ethyl S-2-dimethylaminoethyl ethylphosphonothiolate $$ o-Ethyl S-2-(dimethylamino)ethyl ethylphosphonothiolate $$ S-[2-(Dimethylamino)

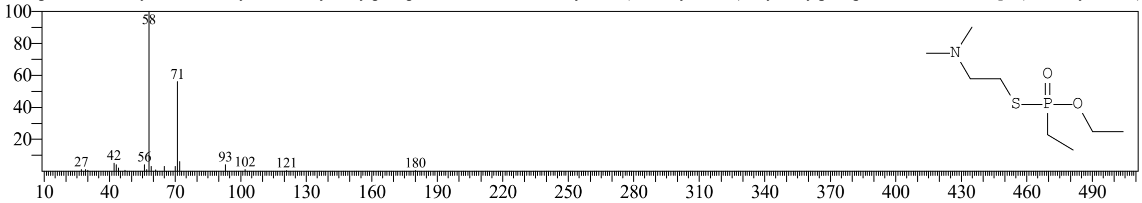

Qualitative Analysis Report

<< Target >>
Line#:51 R.Time:34.733(Scan#:7377) Retention Index:1519 MassPeaks:233
RawMode:Averaged 34.729-34.737(7376-7378) BasePeak:98.05(2331)
BG Mode:Calc. from Peak Group 1 - Event 1 Scan

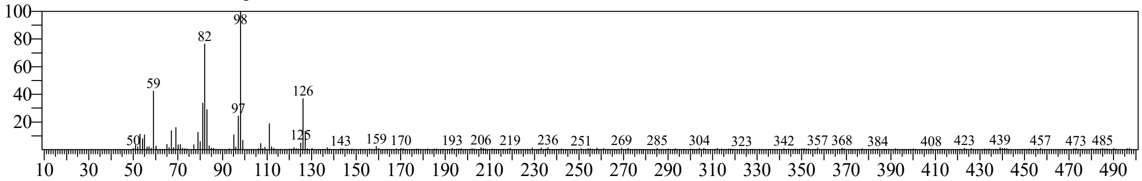

Hit#:1 Entry:1590 Library:NIST17-1.lib
SI:92 Formula:C2H7BN4 CAS:6982-51-0 MolWeight:98 RetIndex:0
CompName:1H-Tetrazaborole, 4,5-dihydro-1,4-dimethyl- $$ .delta.2-Tetrazaboroline, 1,4-dimethyl- $$ Dimethylcyclotetrazenoborane $$ 1,4-Dimethyl-4,5-

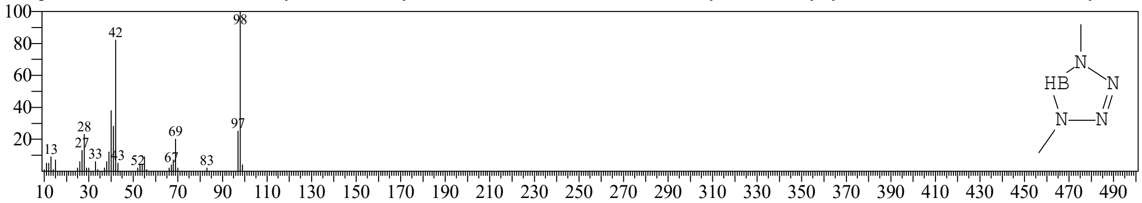

Hit#:2 Entry:3915 Library:NIST17-1.lib
SI:86 Formula:C3H7N5 CAS:0-00-0 MolWeight:113 RetIndex:0
CompName:5-.alpha.-Aminoethyltetrazole $$ 1-(1H-Tetraazol-5-yl)ethanamine # $$

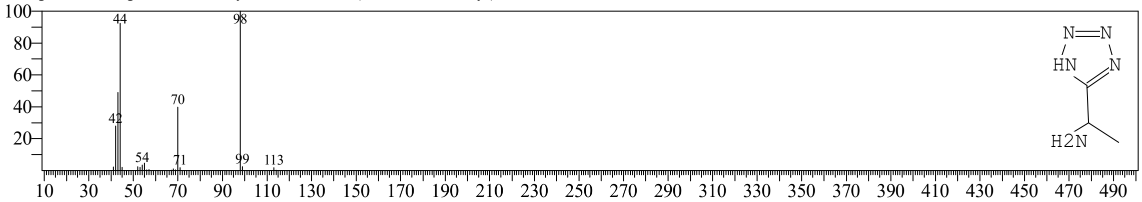

Hit#:3 Entry:26199 Library:NIST17-1.lib
SI:84 Formula:C6H8N4O2 CAS:152028-15-4 MolWeight:168 RetIndex:0
CompName:Acetic acid, 1-(2-methyltetrazol-5-yl)ethenyl ester $$ 1-(2-Methyl-2H-tetraazol-5-yl)vinyl acetate # $$

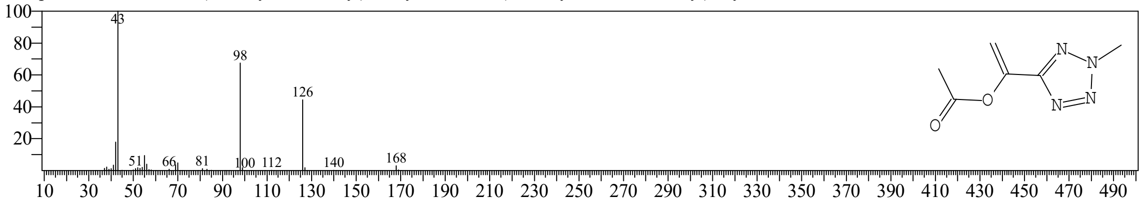

Hit#:4 Entry:59243 Library:NIST17-1.lib
SI:84 Formula:C10H16N2O3 CAS:66398-55-8 MolWeight:212 RetIndex:0
CompName:1-Oxa-3,4-Diazaspiro[4.5]dec-3-ene, 2-(acetyloxy)-2-methyl- $$ 3-Methyl-4-oxa-1,2-diazaspiro[4.5]dec-1-en-3-yl acetate # $$

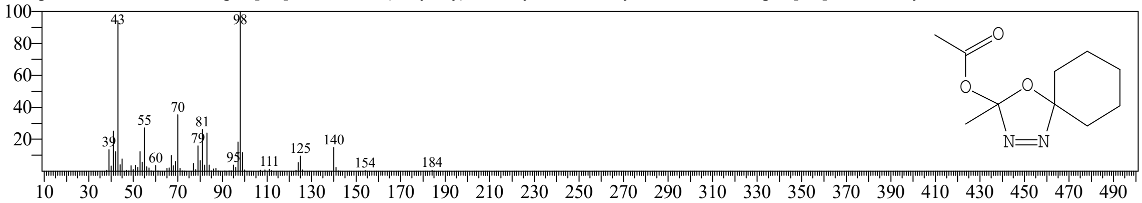

Hit#:5 Entry:26503 Library:NIST17-1.lib
SI:83 Formula:C9H12O3 CAS:22884-78-2 MolWeight:168 RetIndex:1510
CompName:1-Oxaspiro[4.5]decane-2,4-dione

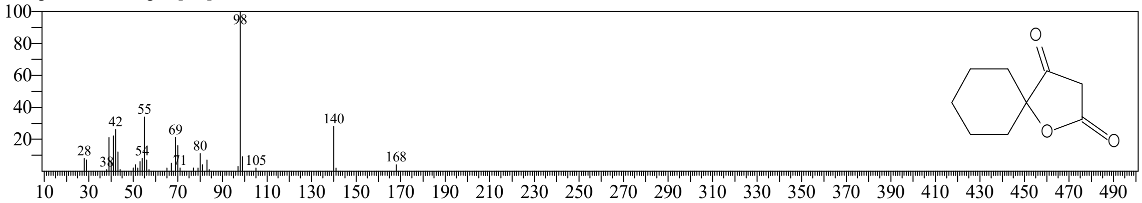

Qualitative Analysis Report

<< Target >>
Line#:52 R.Time:37.175(Scan#:7963) Retention Index:1581 MassPeaks:330
RawMode:Averaged 37.171-37.179(7962-7964) BasePeak:79.05(1895)
BG Mode:Calc. from Peak Group 1 - Event 1 Scan

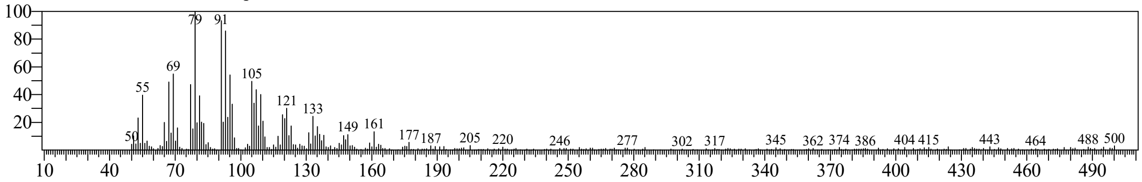

Hit#:1 Entry:145 Library:FFNSC 3.lib
SI:95 Formula:C15 H24 O CAS:1139-30-6 MolWeight:220 RetIndex:1587
CompName:Caryophyllene oxide

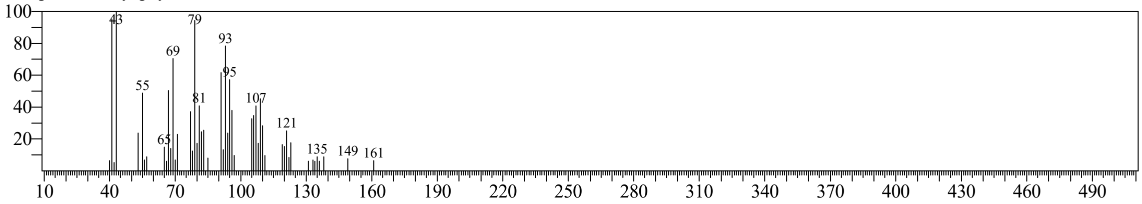

Hit#:2 Entry:5613 Library:NIST17-1.lib
SI:90 Formula:BrN3 CAS:13973-87-0 MolWeight:121 RetIndex:0
CompName:Bromine azide $$ Bromine azide (Br(N3)) $$ Bromine nitride $$ Nitrogen bromide (N3Br) $$

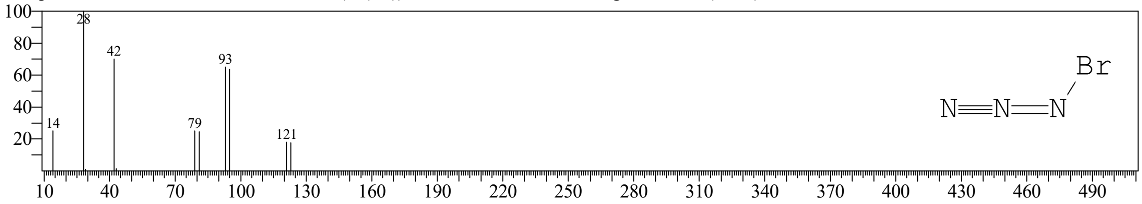

Hit#:3 Entry:2869 Library:FFNSC 3.lib
SI:89 Formula:C15 H26 O CAS:39703-23-6 MolWeight:222 RetIndex:1572
CompName:Longipinanol

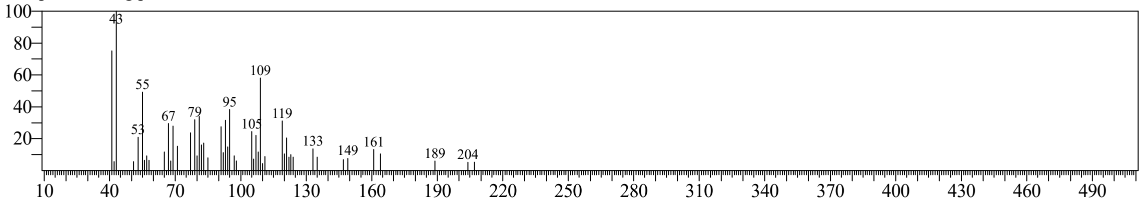

Hit#:4 Entry:903 Library:FFNSC 3.lib
SI:89 Formula:C15 H24 O CAS:72203-24-8 MolWeight:220 RetIndex:1576
CompName:Spathulenol

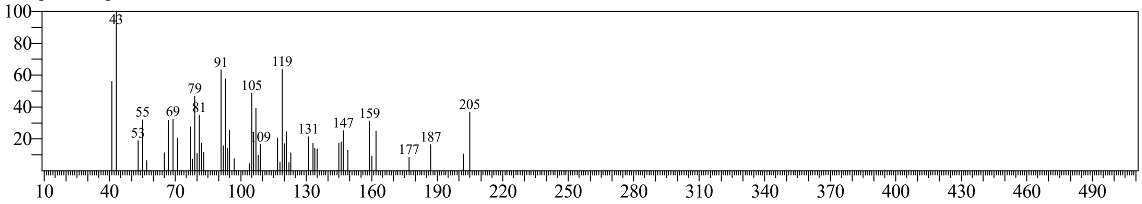

Hit#:5 Entry:66609 Library:NIST17-1.lib
SI:88 Formula:C15H24O CAS:0-00-0 MolWeight:220 RetIndex:1572
CompName:7-Hydroxyfarnesen

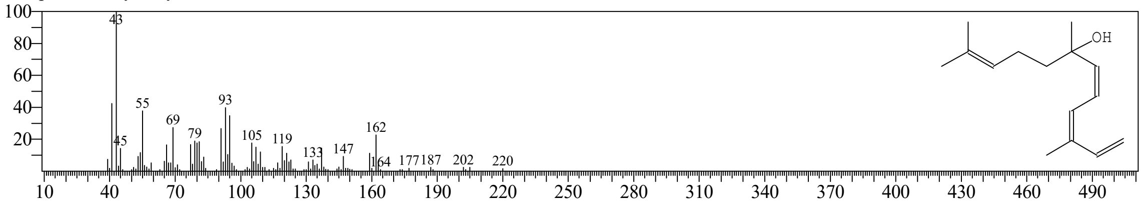

Qualitative Analysis Report

<< Target >>
Line#:53 R.Time:38.754(Scan#:8342) Retention Index:1622 MassPeaks:278
RawMode:Averaged 38.750-38.758(8341-8343) BasePeak:81.10(4493)
BG Mode:Calc. from Peak Group 1 - Event 1 Scan

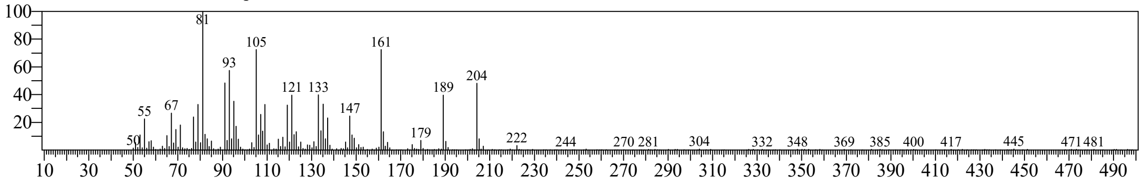

Hit#:1 Entry:1674 Library:FFNSC 3.lib
SI:90 Formula:C15 H26 O CAS:73365-77-2 MolWeight:222 RetIndex:1614
CompName:Cubenol <1,10-di-epi->

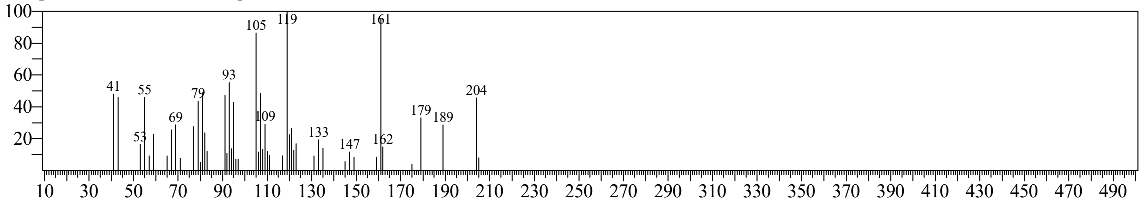

Hit#:2 Entry:533 Library:FFNSC 3.lib
SI:89 Formula:C15 H26 O CAS:98683-12-6 MolWeight:222 RetIndex:1624
CompName:Eudesmol <epi-gamma->

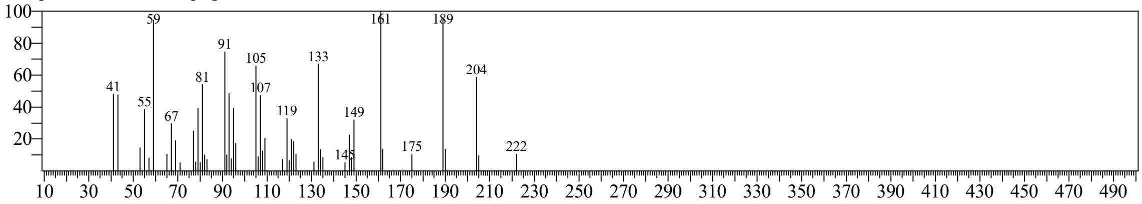

Hit#:3 Entry:68779 Library:NIST17-1.lib
SI:89 Formula:C15H26O CAS:0-00-0 MolWeight:222 RetIndex:1626
CompName:2-(4a,8-Dimethyl-2,3,4,5,6,7-hexahydro-1H-naphthalen-2-yl)propan-2-ol

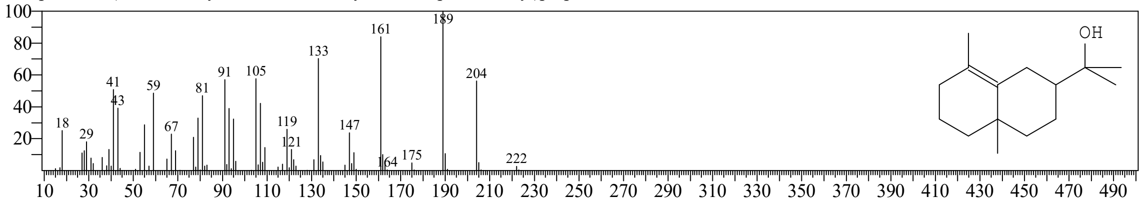

Hit#:4 Entry:1099 Library:FFNSC 3.lib
SI:88 Formula:C15 H26 O CAS:1209-71-8 MolWeight:222 RetIndex:1632
CompName:Eudesmol <gamma->

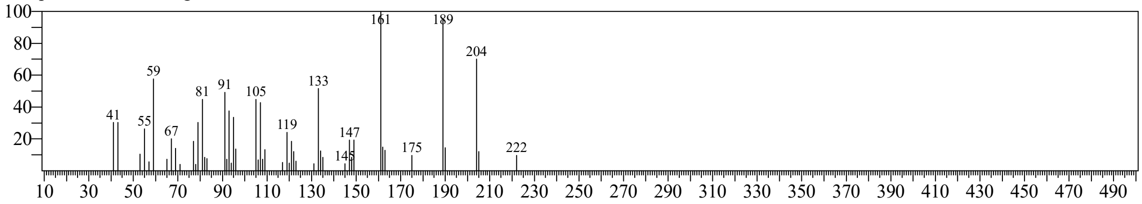

Hit#:5 Entry:2095 Library:FFNSC 3.lib
SI:88 Formula:C15 H26 O CAS:10219-71-3 MolWeight:222 RetIndex:1627
CompName:Eremoligenol

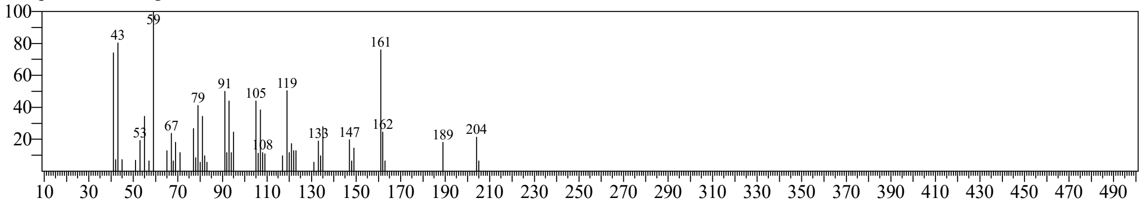

Qualitative Analysis Report

<< Target >>
Line#:54 R.Time:40.321(Scan#:8718) Retention Index:1663 MassPeaks:332
RawMode:Averaged 40.317-40.325(8717-8719) BasePeak:81.10(681)
BG Mode:Calc. from Peak Group 1 - Event 1 Scan

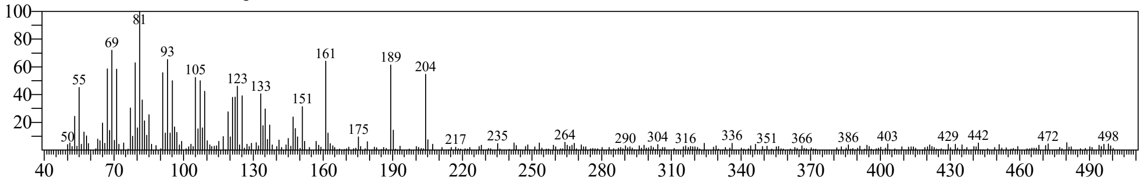

Hit#:1 Entry:1644 Library:FFNSC 3.lib
SI:95 Formula:C15 H26 O CAS:6168-59-8 MolWeight:222 RetIndex:1668
CompName:Intermedeol

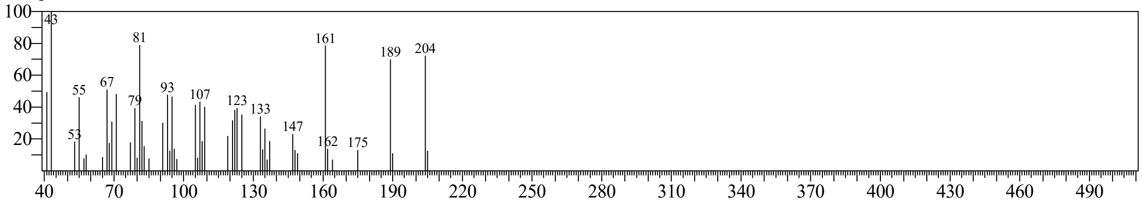

Hit#:2 Entry:2917 Library:FFNSC 3.lib
SI:92 Formula:C15 H26 O CAS:5945-72-2 MolWeight:222 RetIndex:1661
CompName:Intermedeol<neo->

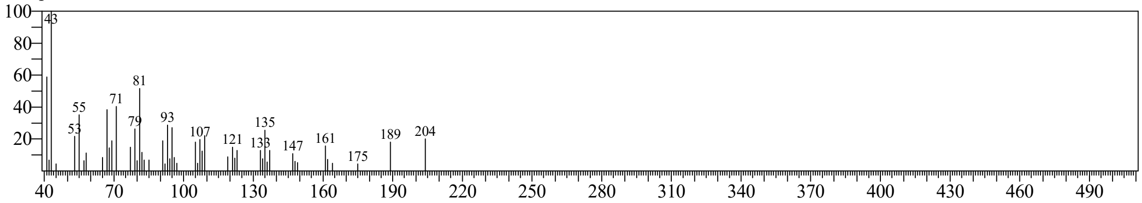

Hit#:3 Entry:2285 Library:FFNSC 3.lib
SI:91 Formula:C15 H26 O CAS:16641-47-7 MolWeight:222 RetIndex:1660
CompName:Selin-11-en-4-alpha-ol

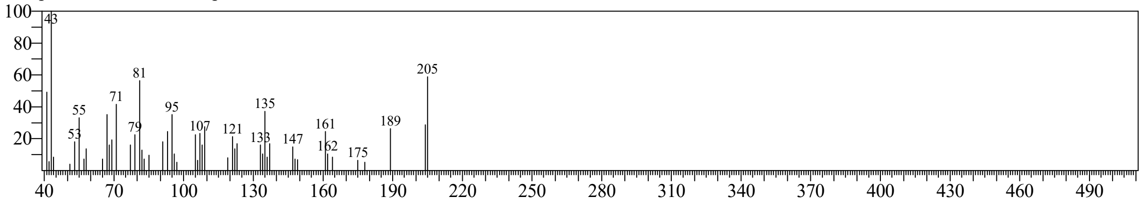

Hit#:4 Entry:2567 Library:FFNSC 3.lib
SI:89 Formula:C15 H24 O CAS:78683-81-5 MolWeight:220 RetIndex:1664
CompName:Caryophyllene <14-hydroxy-(Z)->

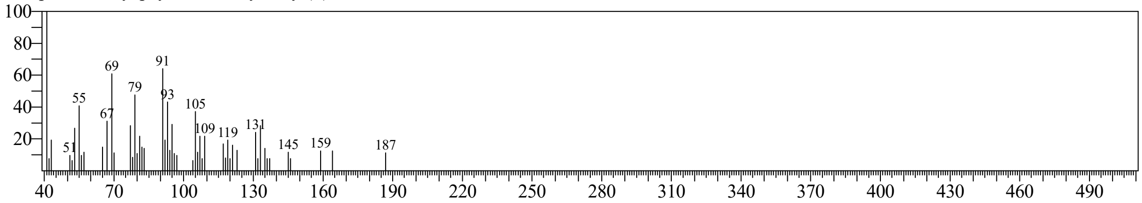

Hit#:5 Entry:2546 Library:FFNSC 3.lib
SI:88 Formula:C15 H24 O CAS:79768-25-5 MolWeight:220 RetIndex:1671
CompName:Caryophyllene <14-hydroxy-9-epi-(E)->

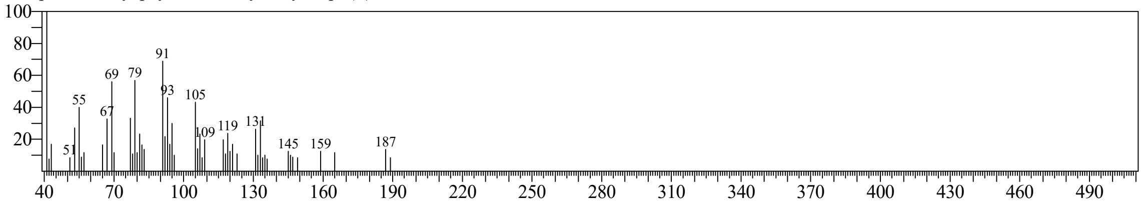

Qualitative Analysis Report

<< Target >>
Line#:55 R.Time:41.521(Scan#:9006) Retention Index:1695 MassPeaks:281
RawMode:Averaged 41.517-41.525(9005-9007) BasePeak:58.05(2230)
BG Mode:Calc. from Peak Group 1 - Event 1 Scan

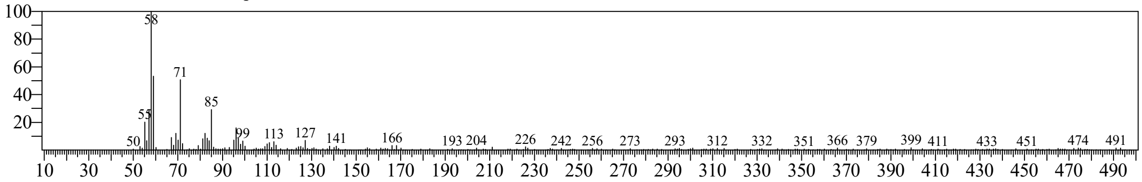

Hit#:1 Entry:683 Library:FFNSC 3.lib
SI:93 Formula:C15 H30 O CAS:2345-28-0 MolWeight:226 RetIndex:1697
CompName:Tridecyl methyl ketone

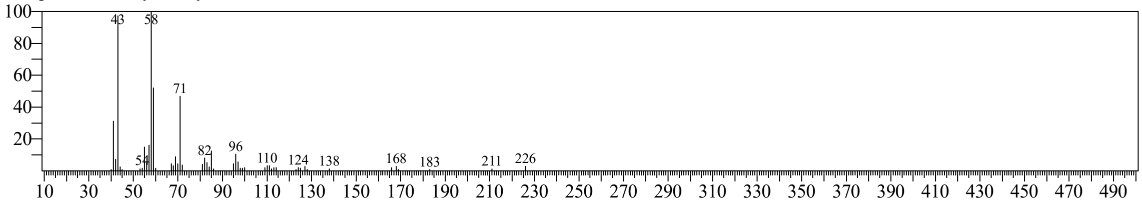

Hit#:2 Entry:143 Library:NIST17-1.lib
SI:93 Formula:C2H8Si CAS:2814-79-1 MolWeight:60 RetIndex:0
CompName:Silane, ethyl- $$ Ethylsilane $$ 1-Silapropane $$ C2H5SiH3 $$

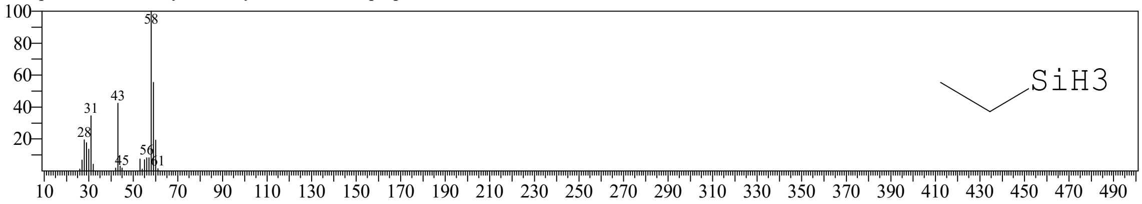

Hit#:3 Entry:9197 Library:NIST17-1.lib
SI:90 Formula:C6H15NO2 CAS:10581-12-1 MolWeight:133 RetIndex:0
CompName:Tetramethylammonium acetate $$ Methanaminium, N,N,N-trimethyl-, acetate $$ Ammonium, tetramethyl-, acetate $$

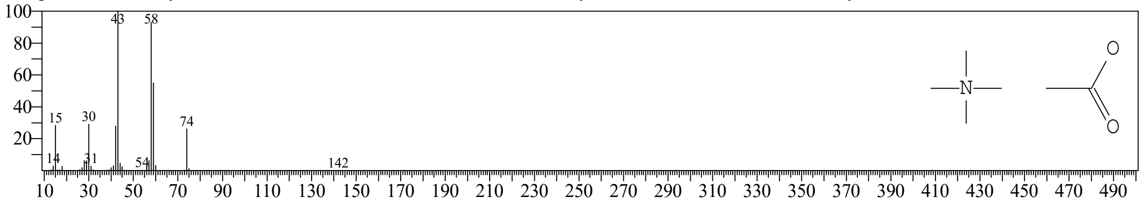

Hit#:4 Entry:805 Library:NIST17-1.lib
SI:89 Formula:C2H2N2S CAS:288-48-2 MolWeight:86 RetIndex:0
CompName:1,2,3-Thiadiazole

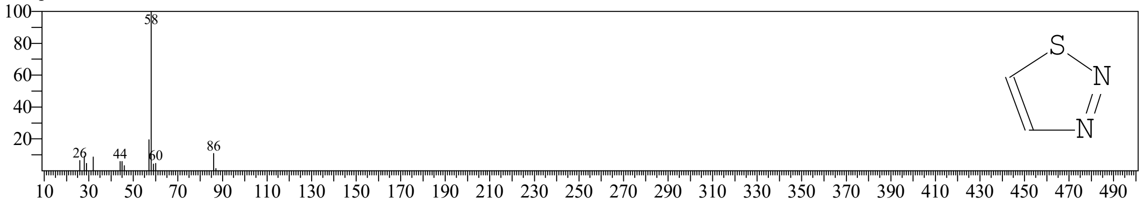

Hit#:5 Entry:58274 Library:NIST17-1.lib
SI:89 Formula:C7H18NO2PS CAS:0-00-0 MolWeight:211 RetIndex:0
CompName:O-Methyl S-2-dimethylaminoethyl ethylphosphonothioate $$ S-[2-(Dimethylamino)ethyl] o-methyl ethylphosphonothioate # $$

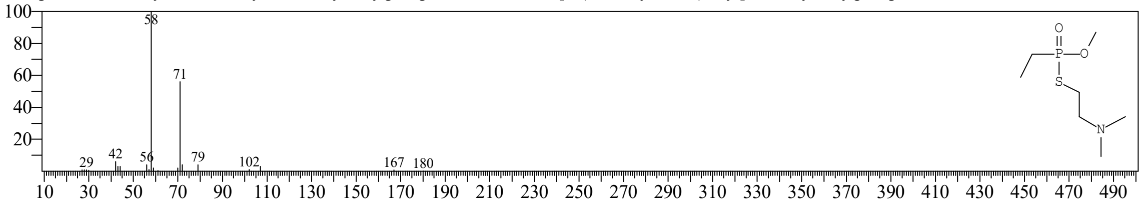

Qualitative Analysis Report

<< Target >>
Line#:56 R.Time:62.392(Scan#:14015) Retention Index:2351 MassPeaks:315
RawMode:Averaged 62.388-62.396(14014-14016) BasePeak:59.05(23041)
BG Mode:Calc. from Peak Group 1 - Event 1 Scan

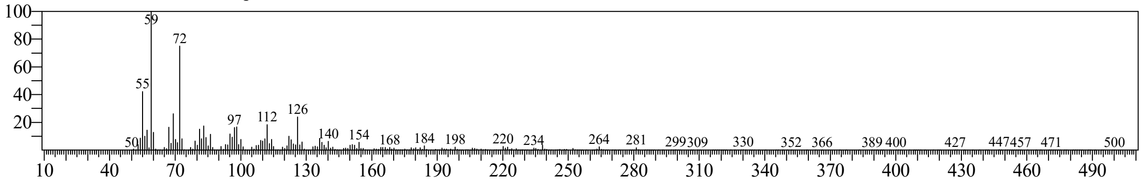

Hit#:1 Entry:27586 Library:NIST17-1.lib
SI:96 Formula:C4H12FN2PS CAS:36267-53-5 MolWeight:170 RetIndex:0
CompName:Phosphonofluoridothioic hydrazide, P-ethyl-2,2-dimethyl- $$ p-Ethyl-N',N'-dimethylphosphonohydrazidothioic fluoride # $$

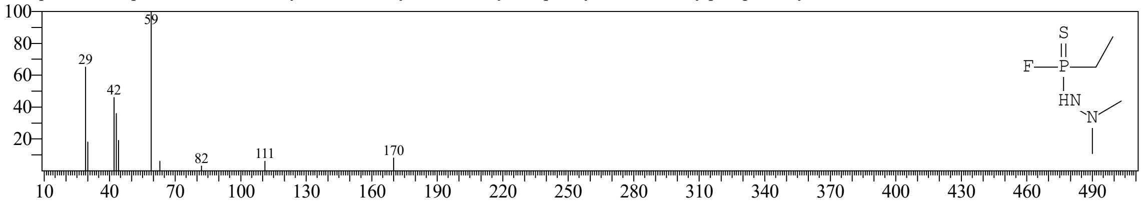

Hit#:2 Entry:19373 Library:NIST17-1.lib
SI:88 Formula:C3H10FN2PS CAS:36267-52-4 MolWeight:156 RetIndex:0
CompName:Phosphonofluoridothioic hydrazide, P,2,2-trimethyl- $$ N',N',p-Trimethylphosphonohydrazidothioic fluoride # $$

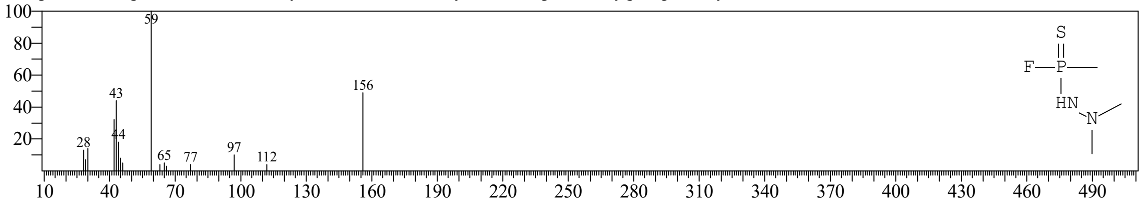

Hit#:3 Entry:120 Library:NIST17-1.lib
SI:87 Formula:CH5N3 CAS:113-00-8 MolWeight:59 RetIndex:0
CompName:Guanidine $$ Aminoformamidine $$ Aminomethanamidine $$ Carbamamidine $$ Carbamidine $$ Guanidin $$ Imidourea $$ Iminourea $$ (NI

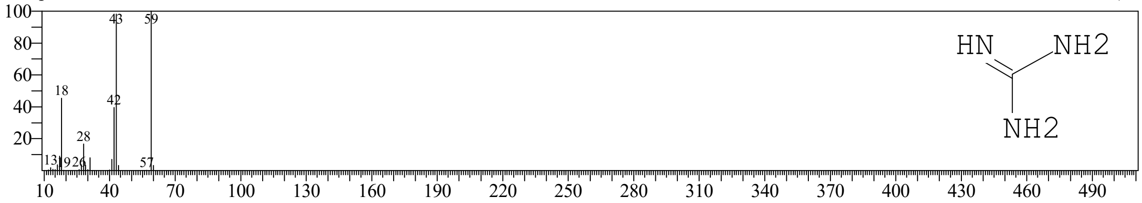

Hit#:4 Entry:157921 Library:NIST17-1.lib
SI:86 Formula:C18H34O4 CAS:137036-69-2 MolWeight:314 RetIndex:2341
CompName:1,2-Ethanediol, 1-[2,2-dimethyl-5-(1-undecenyl)-1,3-dioxolan-4-yl]-, [4R-[4.alpha.(R*),5.alpha.]]- $$ 1-(2,2-Dimethyl-5-[(1Z)-1-undecenyl]-1,

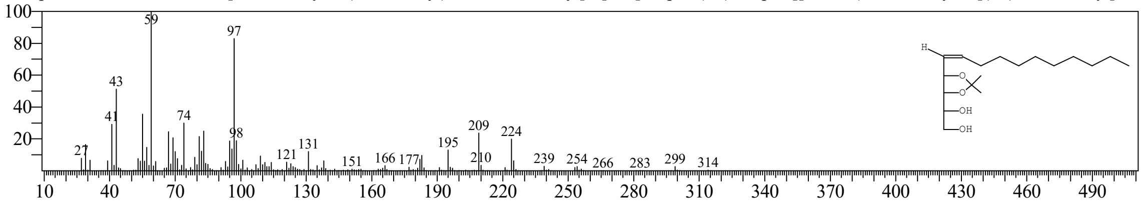

Hit#:5 Entry:2218 Library:NIST17-1.lib
SI:85 Formula:C5H11NO CAS:44603-43-2 MolWeight:101 RetIndex:0
CompName:Methanamine, N-butylidene-, N-oxide $$ Butylidene(methyl)azane oxide # $$

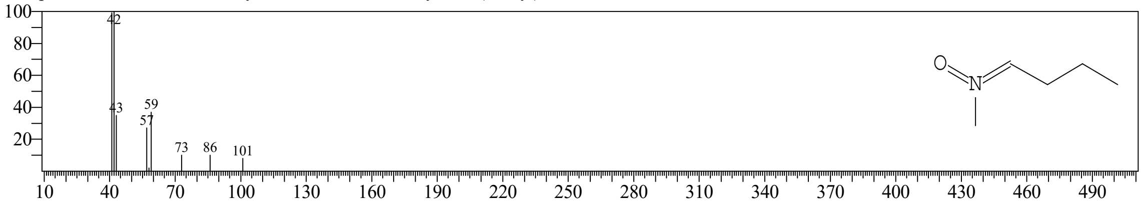

Supplement: Supplementary file 1 [file molecules-31-03346-s001.zip › Supplementary Figure S1_TIC_C citratus.pdf]
