# Supplementary material for: Larvicidal Activity, GC–MS Profiling and Multitarget Molecular Docking of Essential Oils from Cymbopogon citratus, Tanacetum cinerariifolium and Salvia rosmarinus Against Anopheles arabiensis
Source: Molecules. 2026 Sep 21;31(18):3346. doi: 10.3390/molecules31183346 (PMC13610486; doi:10.3390/molecules31183346)

Qualitative Analysis Report

Sample Information

Analyzed	: 21/07/2025
Injection Volume	: 1.00
Data File	: C:\GCMSsolution\Data\Project1\21-07-25_PT303.qgd

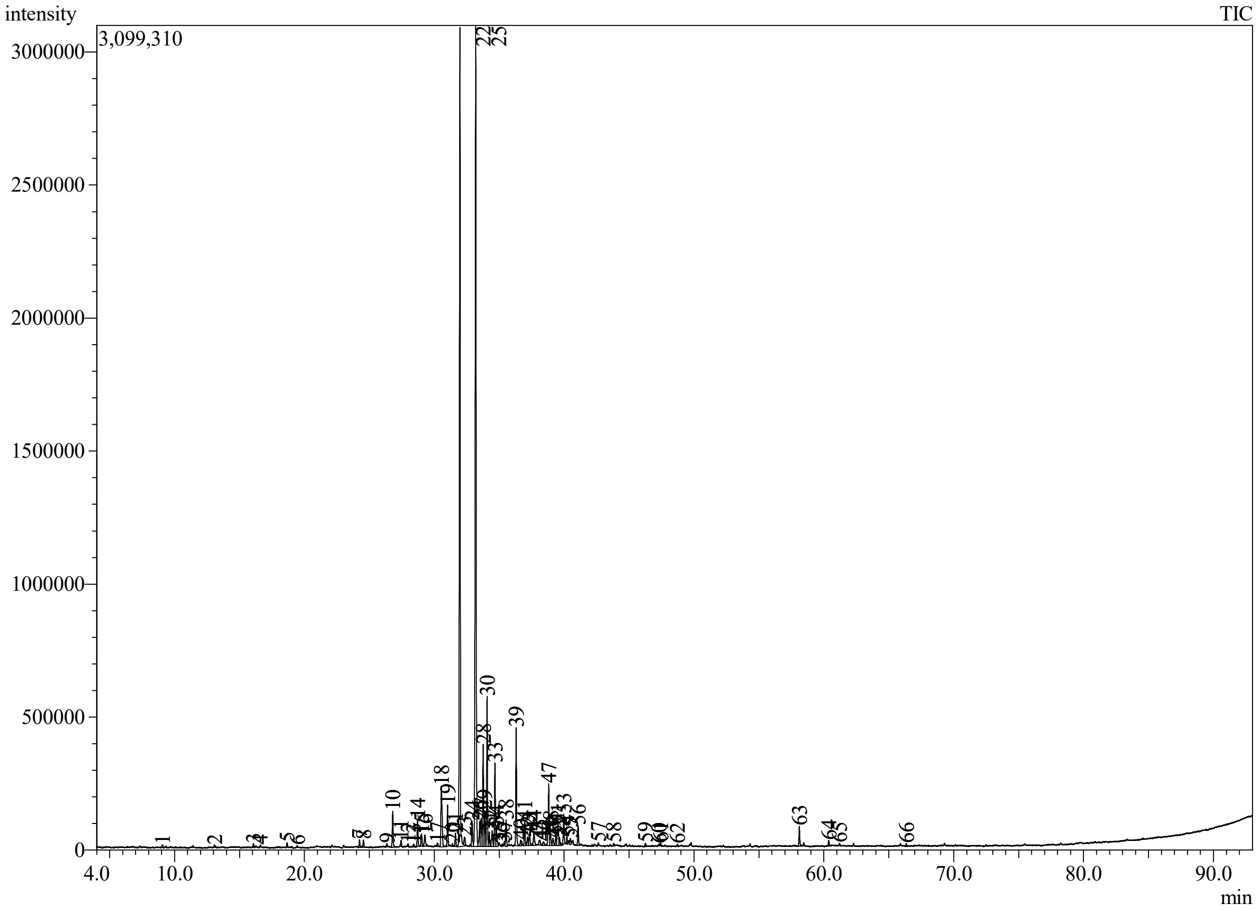

Qualitative Analysis Report

Library
<< Target >>
Line#:1 R.Time:9.067(Scan#:1217) Retention Index:933 MassPeaks:283
RawMode:Averaged 9.063-9.071(1216-1218) BasePeak:93.10(1952)
BG Mode:Calc. from Peak Group 1 - Event 1 Scan

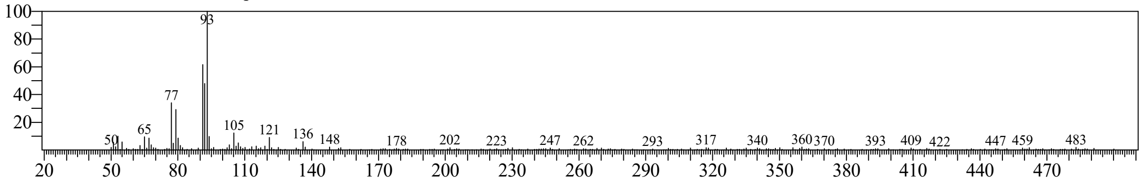

Hit#:1 Entry:817 Library:FFNSC 3.lib
SI:95 Formula:C10 H16 CAS:80-56-8 MolWeight:136 RetIndex:933
CompName:Pinene <alpha->

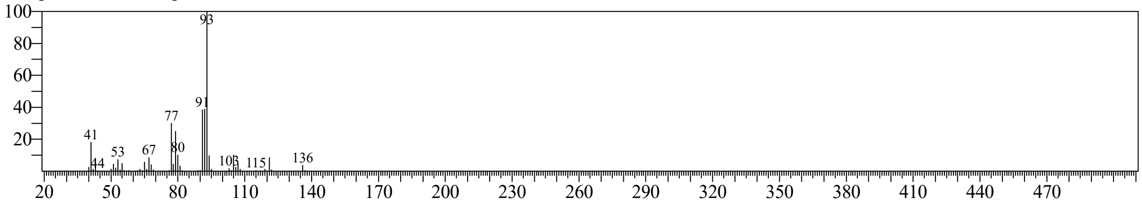

Hit#:2 Entry:957 Library:FFNSC 3.lib
SI:93 Formula:C10 H16 CAS:2867-05-2 MolWeight:136 RetIndex:927
CompName:Thujene <alpha->

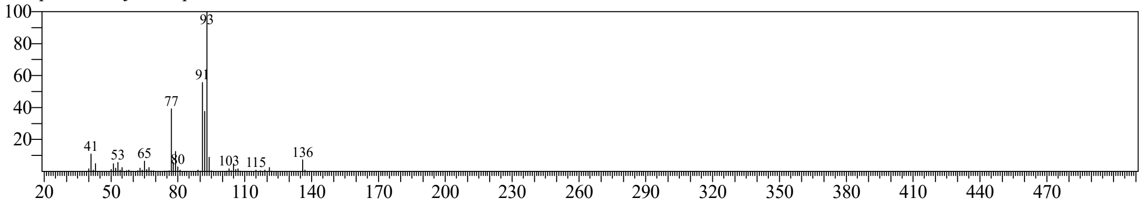

Hit#:3 Entry:24214 Library:NIST17-1.lib
SI:91 Formula:C10H16N2 CAS:87143-58-6 MolWeight:164 RetIndex:0
CompName:3,5-Methanocyclopentapyrazole, 3,3a,4,5,6,6a-hexahydro-3a,4,4-trimethyl-

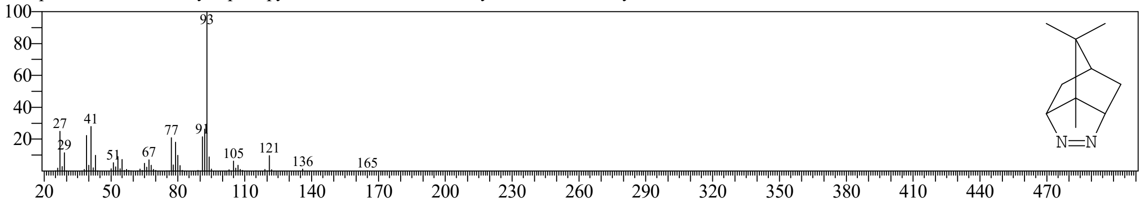

Hit#:4 Entry:10374 Library:NIST17-1.lib
SI:90 Formula:C10H16 CAS:68998-21-0 MolWeight:136 RetIndex:928
CompName:Cyclopropane, 1,1-dimethyl-2-(3-methyl-1,3-butadienyl)- $$ 1,1-Dimethyl-2-[(1E)-3-methyl-1,3-butadienyl]cyclopropane # $$

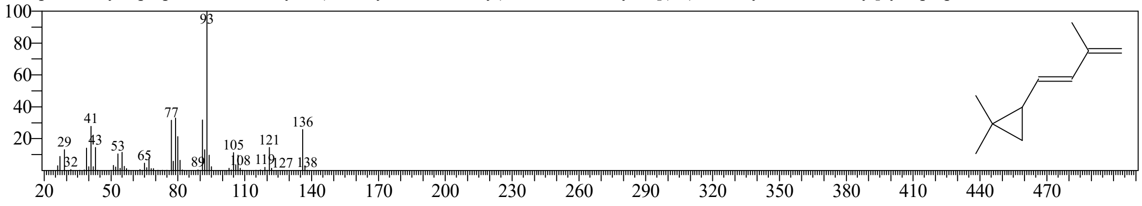

Hit#:5 Entry:10382 Library:NIST17-1.lib
SI:90 Formula:C10H16 CAS:0-00-0 MolWeight:136 RetIndex:943
CompName:Cyclopentene, 3-isopropenyl-5,5-dimethyl- $$ 5-Isopropenyl-3,3-dimethyl-1-cyclopentene # $$

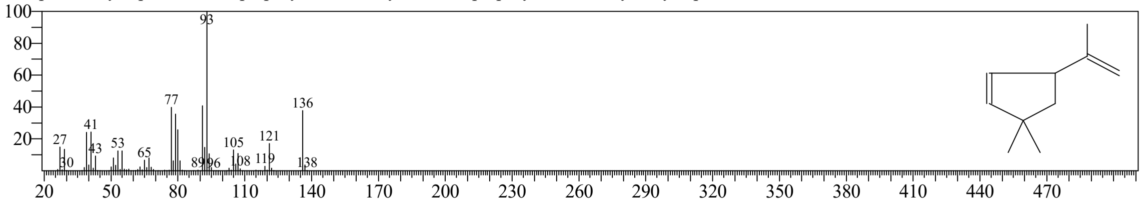

Qualitative Analysis Report

<< Target >>
Line#:2 R.Time:13.079(Scan#:2180) Retention Index:1032 MassPeaks:281
RawMode:Averaged 13.075-13.083(2179-2181) BasePeak:81.10(631)
BG Mode:Calc. from Peak Group 1 - Event 1 Scan

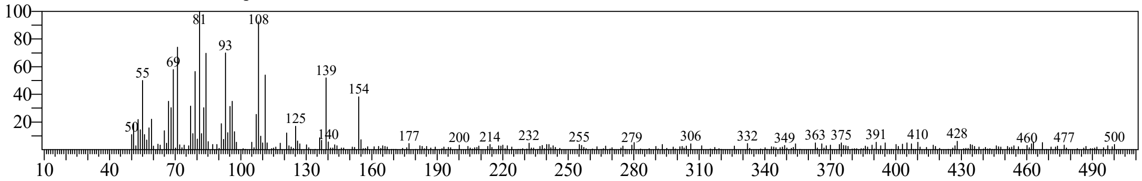

Hit#:1 Entry:621 Library:FFNSC 3.lib
SI:93 Formula:C10 H18 O CAS:470-82-6 MolWeight:154 RetIndex:1032
CompName:Eucalyptol

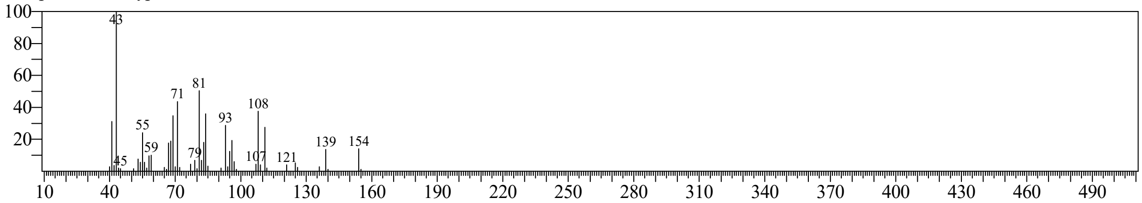

Hit#:2 Entry:6389 Library:NIST17-1.lib
SI:88 Formula:C9H16 CAS:3618-18-6 MolWeight:124 RetIndex:1026
CompName:Methylenecyclooctane $$ Cyclooctane, methylene- $$

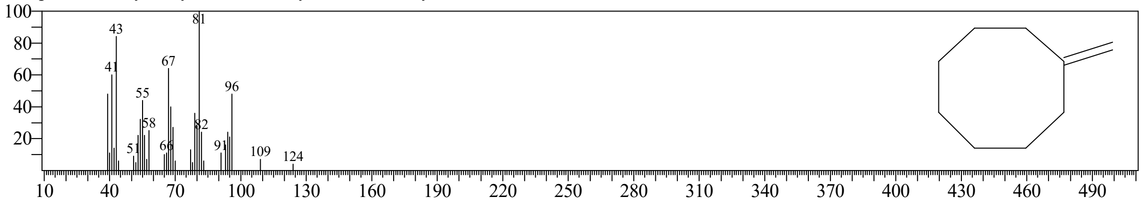

Hit#:3 Entry:6320 Library:NIST17-1.lib
SI:85 Formula:C8H12O CAS:7560-64-7 MolWeight:124 RetIndex:1041
CompName:3-Cyclohexene-1-carboxaldehyde, 4-methyl- $$ 1-Cyclohexene-4-carboxaldehyde, 1-methyl- $$ 4-Methyl-3-cyclohexene-1-carbaldehyde # $$

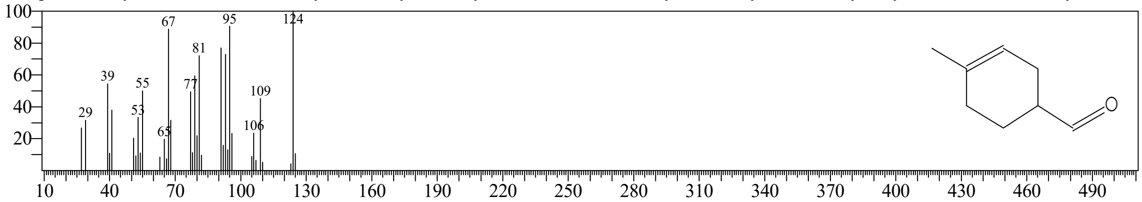

Hit#:4 Entry:3768 Library:NIST17-1.lib
SI:85 Formula:C7H12O CAS:4058-52-0 MolWeight:112 RetIndex:0
CompName:3,4-Pentadien-1-ol, 2,2-dimethyl- $$ 2,2-Dimethyl-3,4-pentadien-1-ol # $$

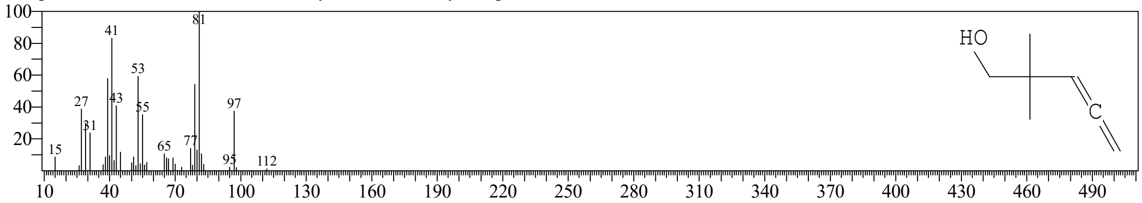

Hit#:5 Entry:2916 Library:NIST17-1.lib
SI:85 Formula:C6H8N2 CAS:50995-98-7 MolWeight:108 RetIndex:1026
CompName:1H-Imidazole, 4-(2-propenyl)- $$ 4-Allylimidazole $$ 4-Allyl-1H-imidazole # $$

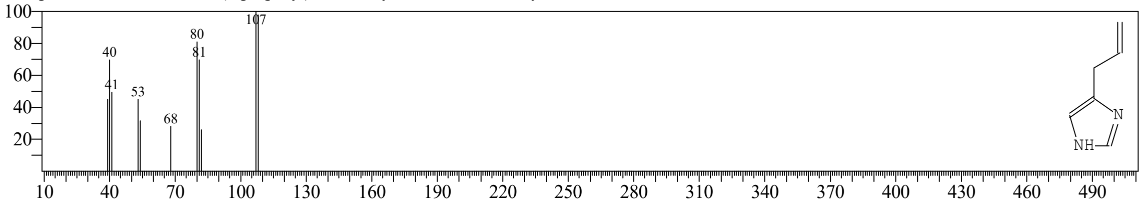

Qualitative Analysis Report

<< Target >>
Line#:3 R.Time:16.071(Scan#:2898) Retention Index:1098 MassPeaks:238
RawMode:Averaged 16.067-16.075(2897-2899) BasePeak:71.10(1644)
BG Mode:Calc. from Peak Group 1 - Event 1 Scan

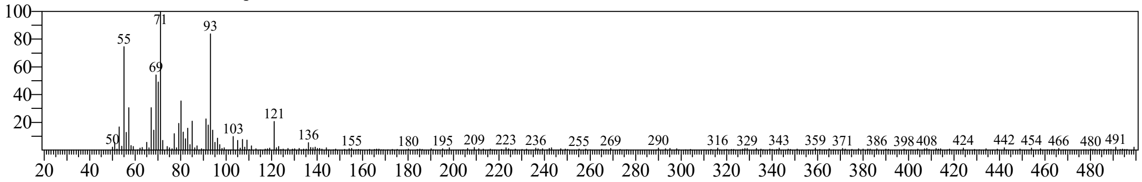

Hit#:1 Entry:3376 Library:FFNSC 3.lib
SI:94 Formula:C17 H23 N O2 CAS:7149-26-0 MolWeight:273 RetIndex:1104
CompName:Linalyl anthranilate

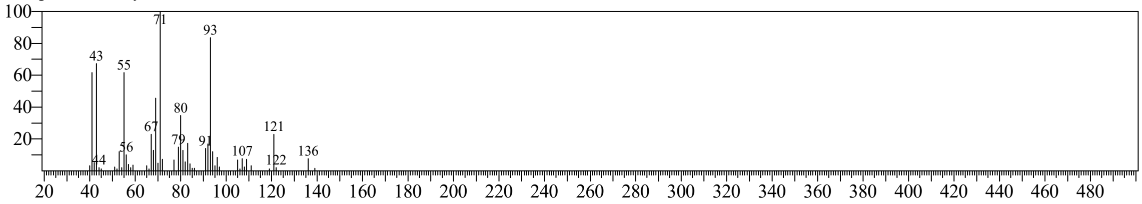

Hit#:2 Entry:365 Library:FFNSC 3.lib
SI:92 Formula:C10 H18 O CAS:78-70-6 MolWeight:154 RetIndex:1101
CompName:Linalool

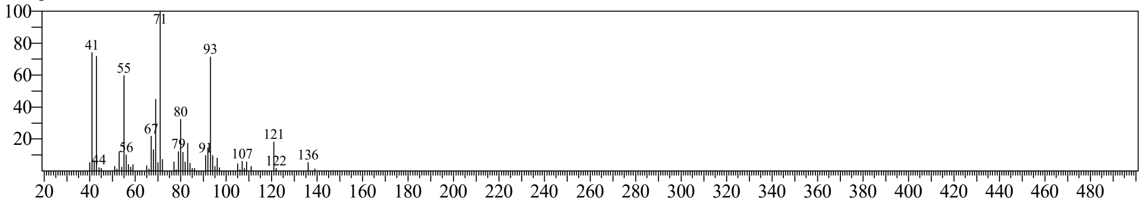

Hit#:3 Entry:12787 Library:NIST17-1.lib
SI:88 Formula:C8H14O2 CAS:5455-24-3 MolWeight:142 RetIndex:1088
CompName:4,5-Octanedione $$ n-Octane-4,5-dione $$ Bibutyryl $$ 4,5-Octadione $$

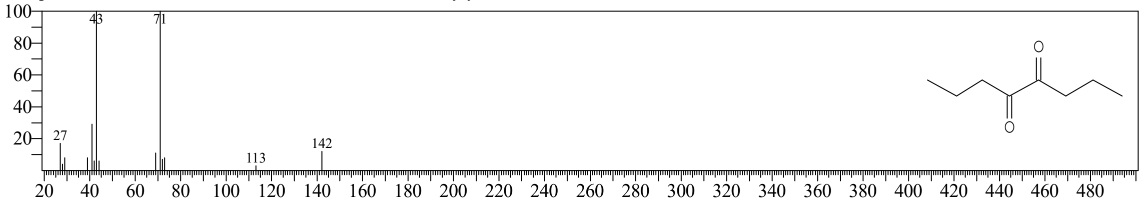

Hit#:4 Entry:29676 Library:NIST17-1.lib
SI:88 Formula:C10H20O2 CAS:117421-32-6 MolWeight:172 RetIndex:1098
CompName:Pentanoic acid, 1,1-dimethylpropyl ester $$ tert-Pentyl pentanoate # $$

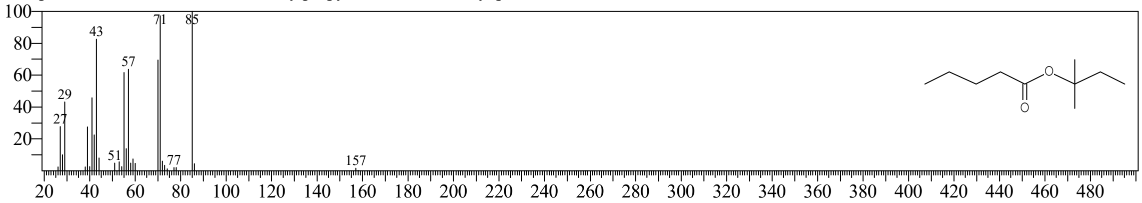

Hit#:5 Entry:954 Library:FFNSC 3.lib
SI:85 Formula:C10 H18 O CAS:17699-16-0 MolWeight:154 RetIndex:1099
CompName:Sabinene hydrate <trans->

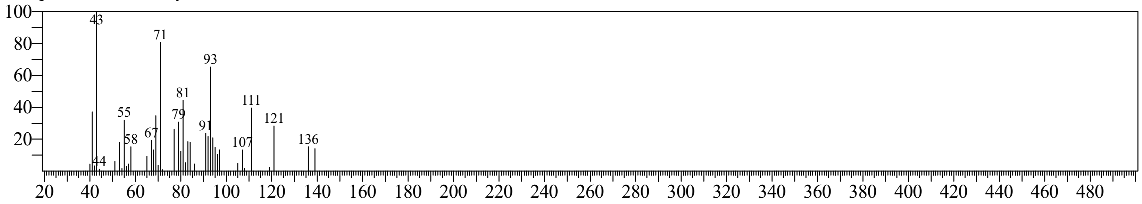

Qualitative Analysis Report

<< Target >>
Line#:4   R.Time:16.596(Scan#:3024) Retention Index:1110 MassPeaks:248
RawMode:Averaged 16.592-16.600(3023-3025) BasePeak:68.10(2004)
BG Mode:Calc. from Peak Group 1 - Event 1 Scan

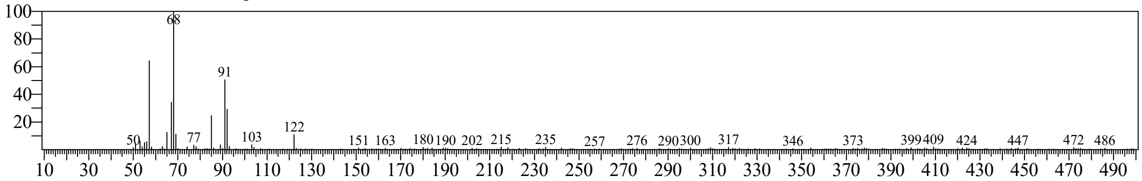

Hit#:1 Entry:980 Library:NIST17-1.lib
SI:99 Formula:F3NO CAS:13847-65-9 MolWeight:87 RetIndex:0
CompName:Trifluoroamine oxide $$ Nitrogen fluoride oxide (NF3O) $$ AMOX $$ Nitrogen Fluoride oxide (F3NO) $$ Trifluoroamine oxide $$ NOF3 $$ N

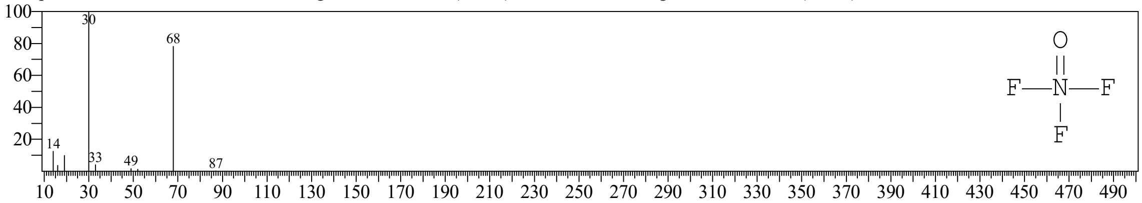

Hit#:2 Entry:19176 Library:NIST17-1.lib
SI:93 Formula:C8H13NO2 CAS:3213-49-8 MolWeight:155 RetIndex:1101
CompName:Butanoic acid, 2-cyano-3-methyl-, ethyl ester $$ Butyric acid, 2-cyano-3-methyl-, ethyl ester $$ Ethyl 2-cyano-3-methylbutanoate $$

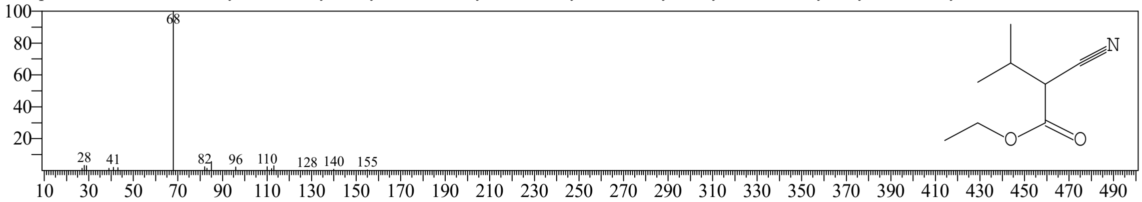

Hit#:3 Entry:1383 Library:NIST17-1.lib
SI:90 Formula:C3H4N4 CAS:18755-47-0 MolWeight:96 RetIndex:0
CompName:1H-Tetrazole, 5-vinyl- $$ 5-Vinyl-1H-tetraazole # $$

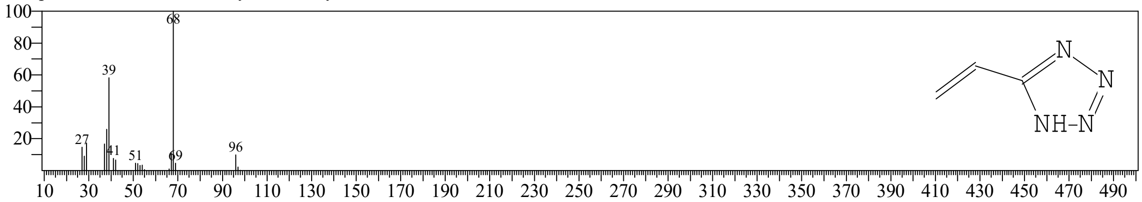

Hit#:4 Entry:642 Library:NIST17-1.lib
SI:89 Formula:C5H9N CAS:7188-38-7 MolWeight:83 RetIndex:0
CompName:Propane, 2-isocyano-2-methyl- $$ tert-Butyl Isocyanide $$ tert-Butylisonitrile $$ (CH3)3CNC $$ t-Butyl isocyanide $$ TBIN $$ 2-Methyl-2-is

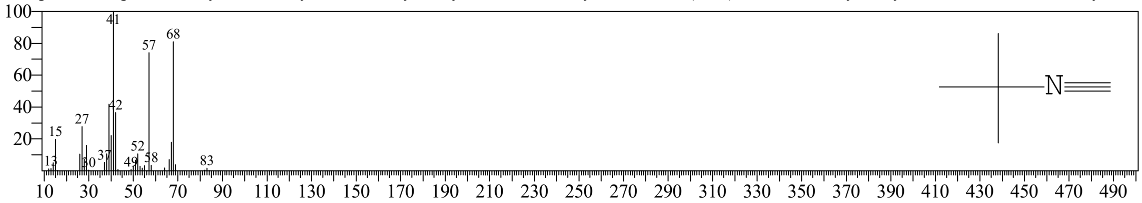

Hit#:5 Entry:8816 Library:NIST17-1.lib
SI:88 Formula:C5H8O2S CAS:1193-10-8 MolWeight:132 RetIndex:0
CompName:2,5-Dihydro-3-methyl-thiophene 1,1-dioxide $$ 3-Methylsulfolene $$ Thiophene, 2,5-dihydro-3-methyl-, 1,1-dioxide $$ 1,3-Butadiene, 2-methy

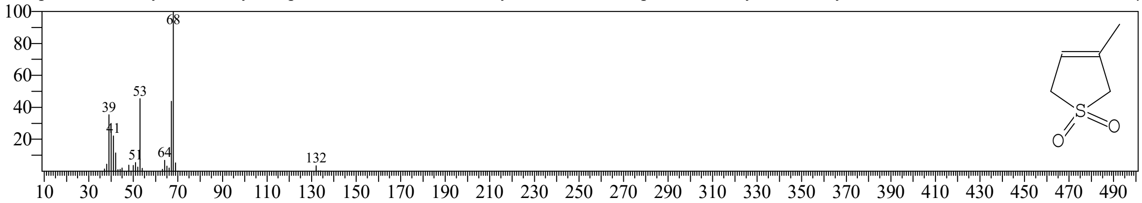

Qualitative Analysis Report

<< Target >>
Line#:5 R.Time:18.658(Scan#:3519) Retention Index:1154 MassPeaks:267
RawMode:Averaged 18.654-18.663(3518-3520) BasePeak:81.10(2582)
BG Mode:Calc. from Peak Group 1 - Event 1 Scan

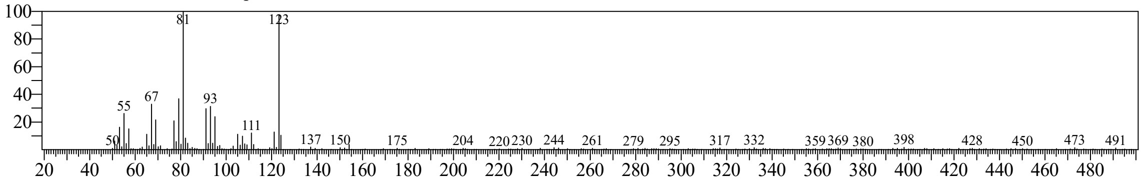

Hit#:1 Entry:1462 Library:FFNSC 3.lib
SI:95 Formula:C10 H18 O CAS:18383-58-9 MolWeight:154 RetIndex:1155
CompName:Chrysanthemol <trans->

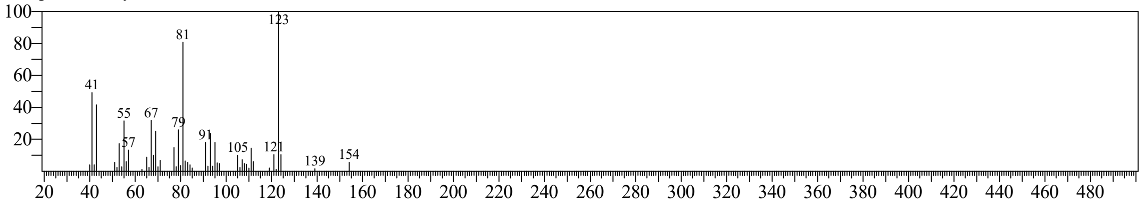

Hit#:2 Entry:158522 Library:NIST17-1.lib
SI:89 Formula:C11H5N7O5 CAS:0-00-0 MolWeight:315 RetIndex:0
CompName:12-(Furan-2-ylmethyl)-4,9,15-trioxa-3,5,8,10,12,14,16-heptaazatetracyclo[11.3.0.0(2,6).0(7,11)]hexadeca-1(16),2,5,7,10,13-hexaen-3-one

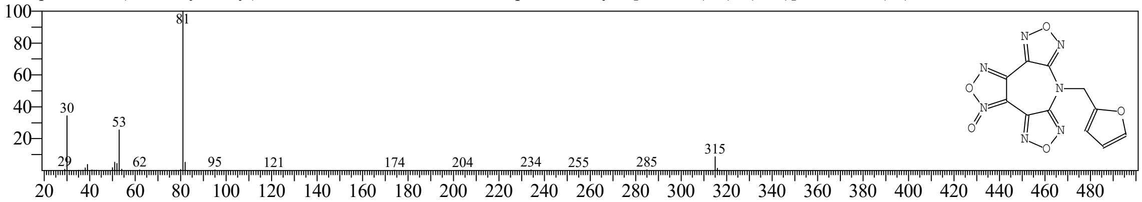

Hit#:3 Entry:17640 Library:NIST17-1.lib
SI:89 Formula:C10H16O CAS:590-71-6 MolWeight:152 RetIndex:0
CompName:2,2-Dimethylocta-3,4-dienal $$ 2,2-Dimethyl-3,4-octadienal # $$ 3,4-Octadienal, 2,2-dimethyl $$

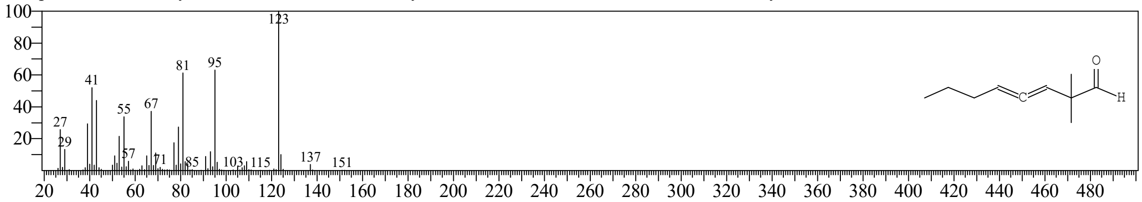

Hit#:4 Entry:153823 Library:NIST17-1.lib
SI:88 Formula:C18H30Zn CAS:74842-24-3 MolWeight:310 RetIndex:0
CompName:Zinc, bis[2,2-dimethyl-3-(2-methyl-2-propenyl)cyclopropyl]- $$ Bis[2,2-dimethyl-3-(2-methyl-2-propenyl)cyclopropyl]zinc # $$

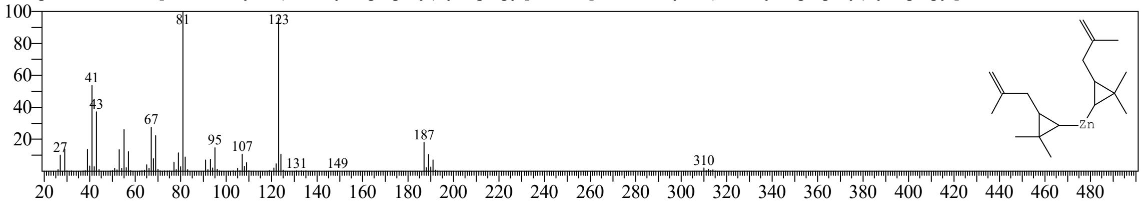

Hit#:5 Entry:153822 Library:NIST17-1.lib
SI:88 Formula:C18H30Zn CAS:81069-88-7 MolWeight:310 RetIndex:0
CompName:Zinc, bis(2,2-dimethyl-3(cis)-(1-methylprop-2-enyl)-cyclopropyl)- $$ Bis[2,2-dimethyl-3-(1-methyl-2-propenyl)cyclopropyl]zinc # $$

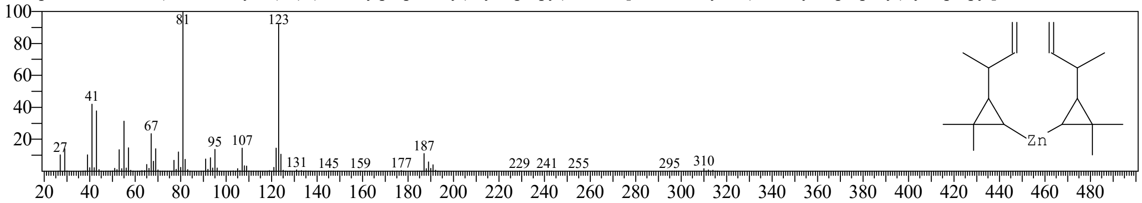

Qualitative Analysis Report

<< Target >>
Line#:6 R.Time:19.438(Scan#:3706) Retention Index:1171 MassPeaks:225
RawMode:Averaged 19.433-19.442(3705-3707) BasePeak:95.10(2496)
BG Mode:Calc. from Peak Group 1 - Event 1 Scan

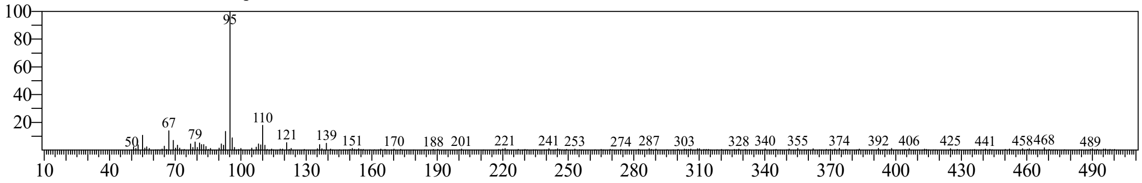

Hit#:1 Entry:1262 Library:FFNSC 3.lib
SI:95 Formula:C10 H18 O CAS:507-70-0 MolWeight:154 RetIndex:1173
CompName:Borneol

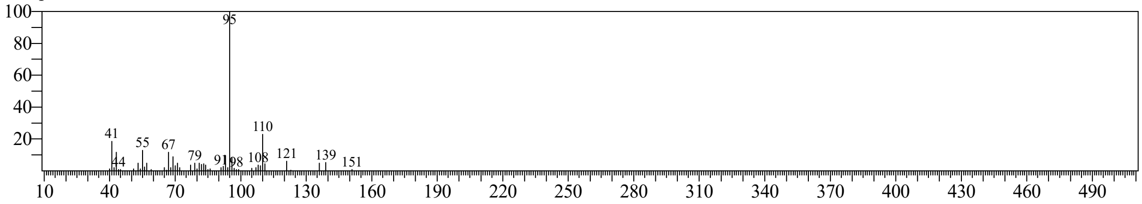

Hit#:2 Entry:687 Library:FFNSC 3.lib
SI:93 Formula:C10 H18 O CAS:10385-78-1 MolWeight:154 RetIndex:1165
CompName:Isoborneol

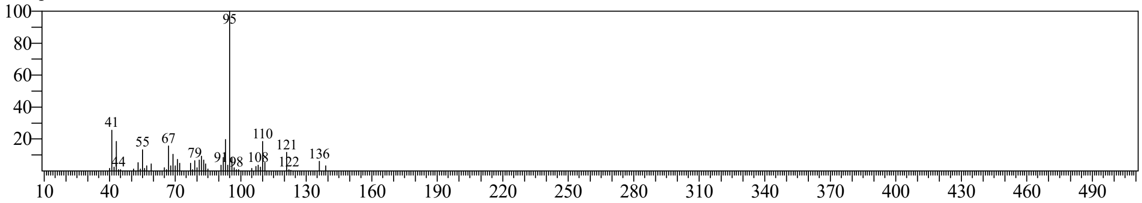

Hit#:3 Entry:26760 Library:NIST17-1.lib
SI:88 Formula:C10H16O2 CAS:5809-03-0 MolWeight:168 RetIndex:1172
CompName:2-Cyclopentene-1-carboxylic acid, 1,2-dimethyl-, ethyl ester $$ Ethyl 1,2-dimethyl-2-cyclopentene-1-carboxylate # $$

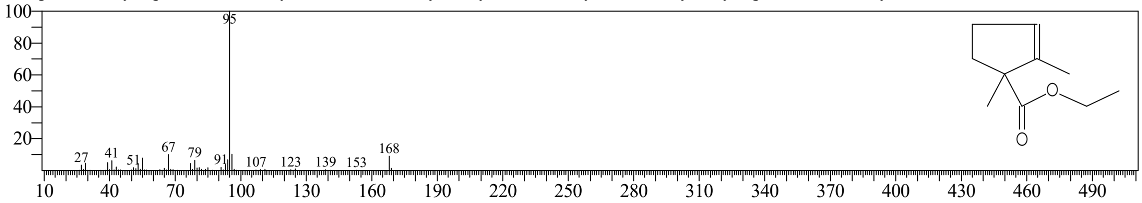

Hit#:4 Entry:36077 Library:NIST17-1.lib
SI:88 Formula:C10H14O3 CAS:0-00-0 MolWeight:182 RetIndex:1179
CompName:2-Furoic acid, 3-methylbut-2-yl ester

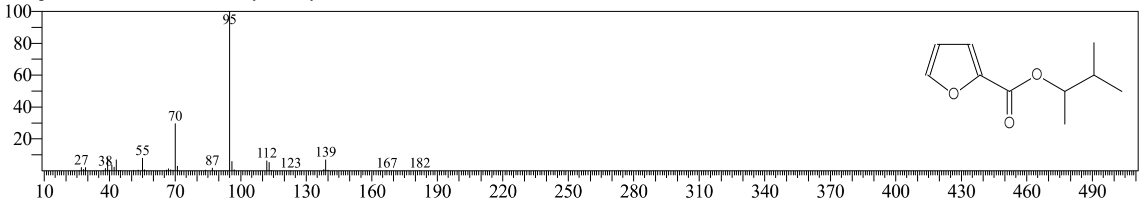

Hit#:5 Entry:26963 Library:NIST17-1.lib
SI:87 Formula:C11H20O CAS:2371-42-8 MolWeight:168 RetIndex:1161
CompName:2-Methylisoborneol

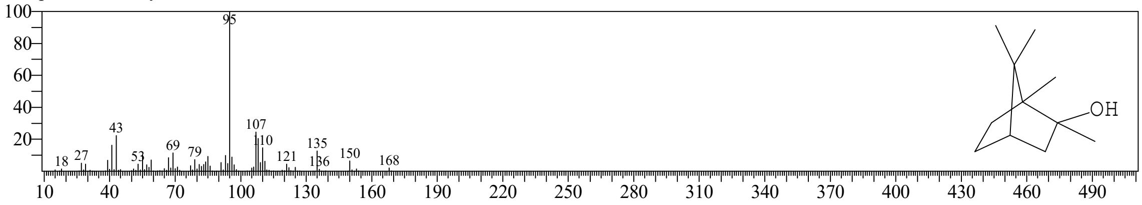

Qualitative Analysis Report

<< Target >>
Line#:7 R.Time:24.238(Scan#:4858) Retention Index:1275 MassPeaks:263
RawMode:Averaged 24.233-24.242(4857-4859) BasePeak:121.15(3006)
BG Mode:Calc. from Peak Group 1 - Event 1 Scan

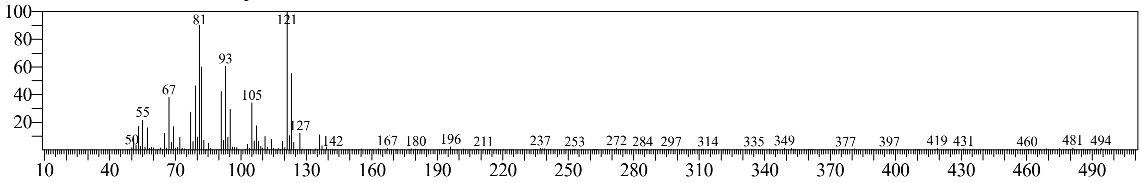

Hit#:1 Entry:46524 Library:NIST17-1.lib
SI:94 Formula:C12H20O2 CAS:0-00-0 MolWeight:196 RetIndex:1266
CompName:Chrysanthemyl alcohol, O-acetyl- (stereoisomer 1)

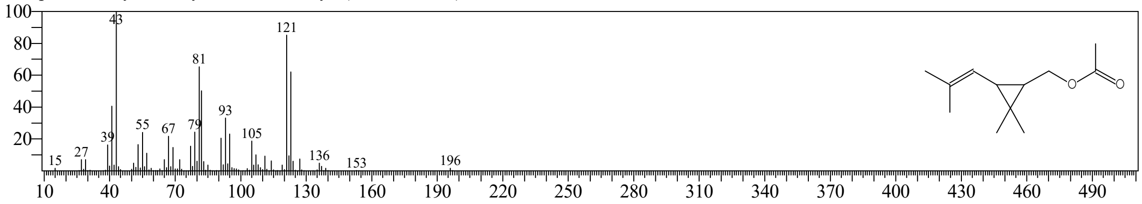

Hit#:2 Entry:46523 Library:NIST17-1.lib
SI:93 Formula:C12H20O2 CAS:0-00-0 MolWeight:196 RetIndex:1266
CompName:Chrysanthemyl alcohol, O-acetyl- (stereoisomer 2)

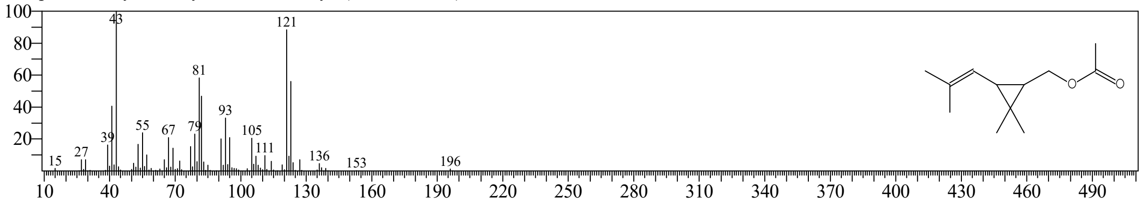

Hit#:3 Entry:46613 Library:NIST17-1.lib
SI:92 Formula:C12H20O2 CAS:54999-63-2 MolWeight:196 RetIndex:1266
CompName:((1R,3R)-2,2-dimethyl-3-(2-methylprop-1-en-1-yl)cyclopropyl)methyl acetate $$ Cyclopropanemethanol, 2,2-dimethyl-3-(2-methyl-1-propen-1-

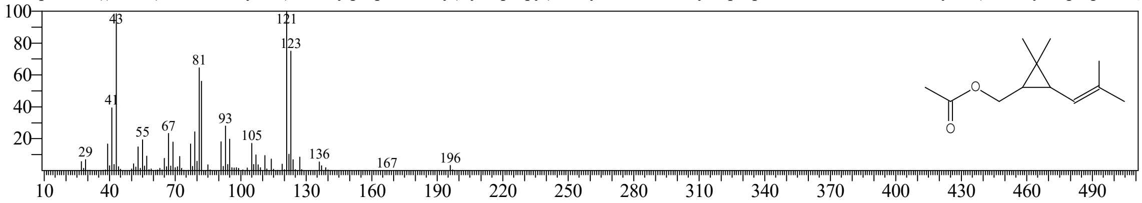

Hit#:4 Entry:34592 Library:NIST17-1.lib
SI:91 Formula:C10H12O3 CAS:51835-45-1 MolWeight:180 RetIndex:1271
CompName:Benzenemethanol, .alpha.-methoxy-, acetate $$ Methoxy(phenyl)methyl acetate # $$

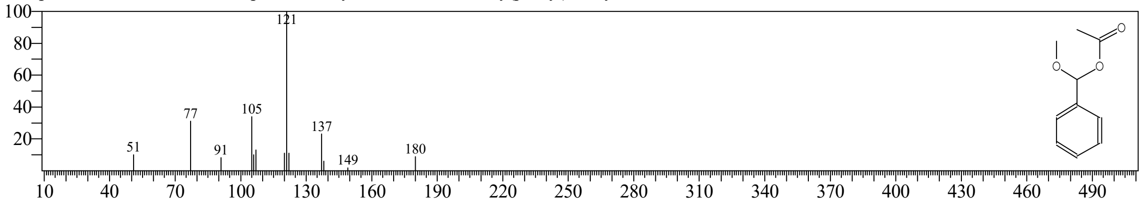

Hit#:5 Entry:24215 Library:NIST17-1.lib
SI:86 Formula:C10H16N2 CAS:0-00-0 MolWeight:164 RetIndex:0
CompName:2,3-Diazabicyclo[2.2.1]hept-2-ene, 5-ethenyl-4,7,7-trimethyl-, (1.alpha.,4.alpha.,5.beta.)- $$ 1,7,7-Trimethyl-6-vinyl-2,3-diazabicyclo[2.2.1]hep

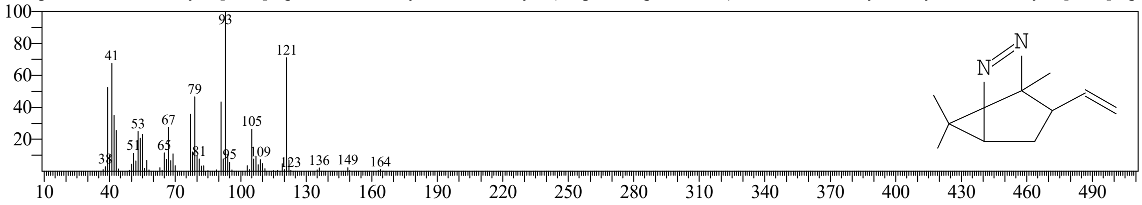

Qualitative Analysis Report

<< Target >>
Line#:8   R.Time:24.546(Scan#:4932) Retention Index:1282 MassPeaks:317
RawMode:Averaged 24.542-24.550(4931-4933) BasePeak:69.10(5277)
BG Mode:Calc. from Peak Group 1 - Event 1 Scan

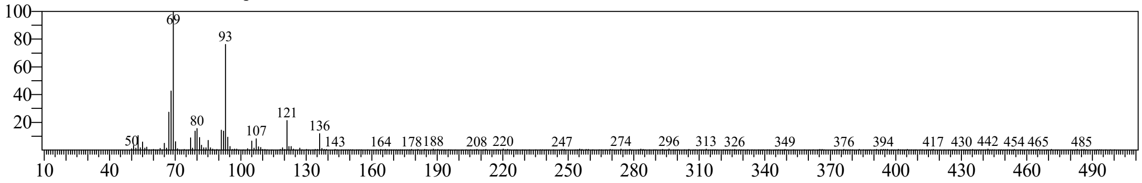

Hit#:1 Entry:228 Library:NIST17-1.lib
SI:99 Formula:C2H3N3 CAS:288-36-8 MolWeight:69 RetIndex:0
CompName:1H-1,2,3-Triazole $$ V-triazole $$ Osotriazole $$ Pyrrodiazole $$ Triazacyclopentadiene $$ 1,2,3-Triazole $$

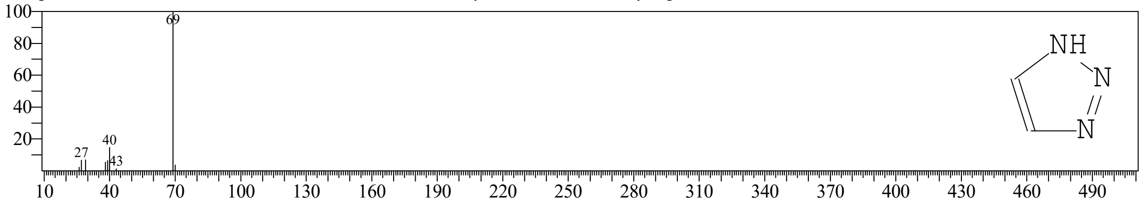

Hit#:2 Entry:1106 Library:FFNSC 3.lib
SI:97 Formula:C12 H20 O2 CAS:25905-14-0 MolWeight:196 RetIndex:1284
CompName:Lavandulyl acetate

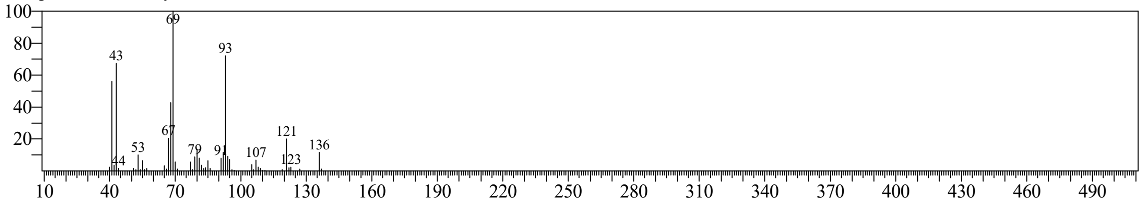

Hit#:3 Entry:981 Library:NIST17-1.lib
SI:96 Formula:CF4 CAS:75-73-0 MolWeight:88 RetIndex:0
CompName:Tetrafluoromethane $$ Carbon tetrafluoride $$ Methane, tetrafluoro- $$ Arcton 0 $$ Carbon fluoride (CF4) $$ Freon 14 $$ FC 14 $$ Perfluoro

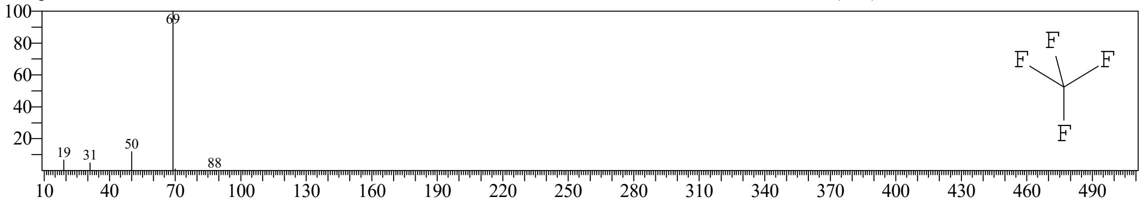

Hit#:4 Entry:10642 Library:NIST17-1.lib
SI:95 Formula:CF5P CAS:1112-04-5 MolWeight:138 RetIndex:0
CompName:Trifluoromethyldifluorophosphine $$ Trifluoromethylphosphonous difluoride # $$

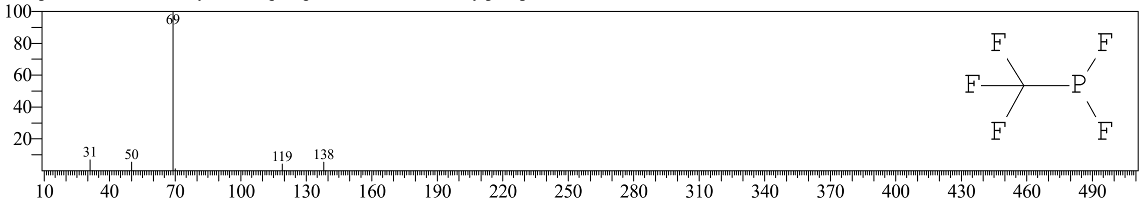

Hit#:5 Entry:10455 Library:NIST17-1.lib
SI:95 Formula:CClF4N CAS:13880-72-3 MolWeight:137 RetIndex:0
CompName:N-Chloro-N-fluoro-trifluoromethylamine $$ N-Chloro-N-tetrafluoromethanamine # $$

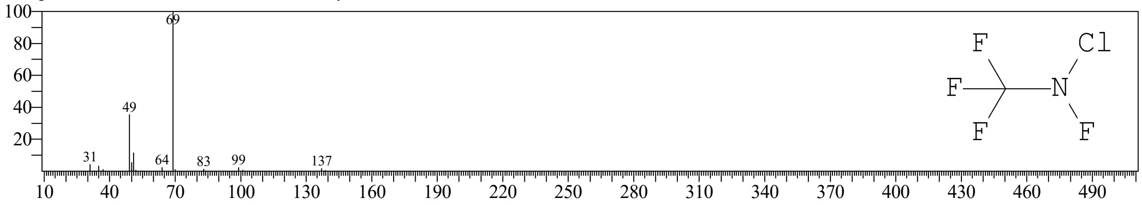

Qualitative Analysis Report

<< Target >>
Line#:9   R.Time:26.350(Scan#:5365) Retention Index:1322 MassPeaks:300
RawMode:Averaged 26.346-26.354(5364-5366) BasePeak:121.15(1222)
BG Mode:Calc. from Peak Group 1 - Event 1 Scan

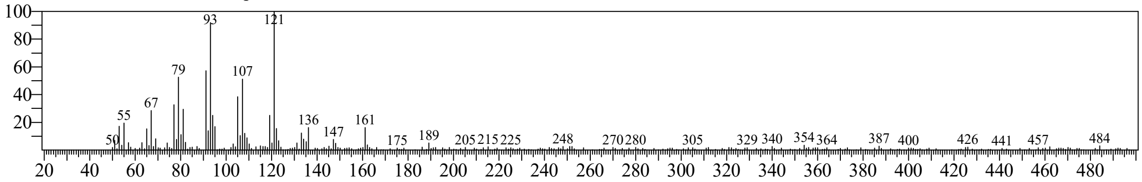

Hit#:1 Entry:71888 Library:NIST17-1.lib
SI:91 Formula:C12H18O2S CAS:0-00-0 MolWeight:226 RetIndex:0
CompName:3,4-Dimethyl-2,5-diprop-2-enyl-2,5-dihydrothiophene 1,1-dioxide

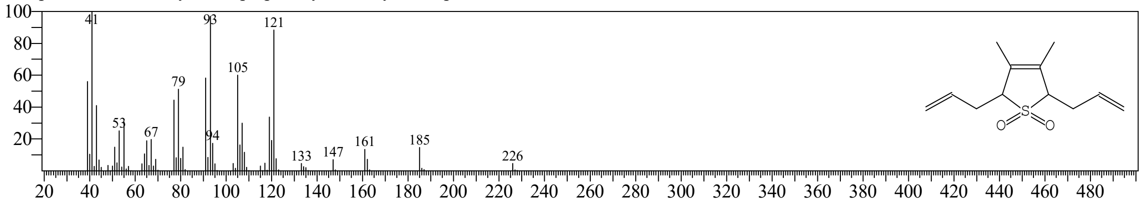

Hit#:2 Entry:24215 Library:NIST17-1.lib
SI:89 Formula:C10H16N2 CAS:0-00-0 MolWeight:164 RetIndex:0
CompName:2,3-Diazabicyclo[2.2.1]hept-2-ene, 5-ethenyl-4,7,7-trimethyl-, (1.alpha.,4.alpha.,5.beta.)- $$ 1,7,7-Trimethyl-6-vinyl-2,3-diazabicyclo[2.2.1]hep

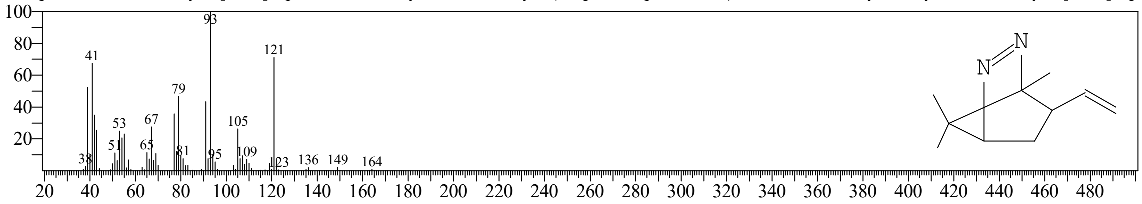

Hit#:3 Entry:52646 Library:NIST17-1.lib
SI:89 Formula:C13H20N2 CAS:0-00-0 MolWeight:204 RetIndex:0
CompName:1,4-Methanocycloocta[d]pyridazine, 1,4,4a,5,6,9,10,10a-octahydro-11,11-dimethyl-, (1.alpha.,4.alpha.,4a.alpha.,10a.alpha.)-

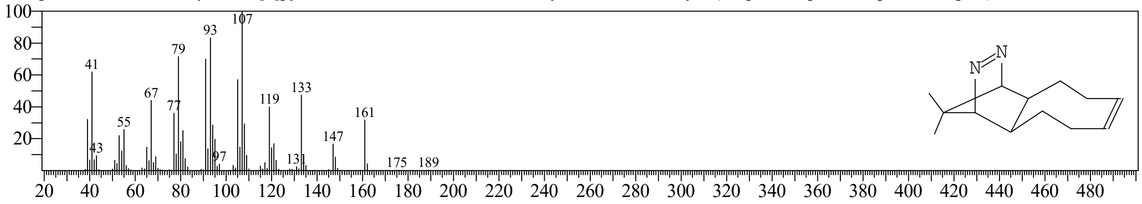

Hit#:4 Entry:33477 Library:NIST17-1.lib
SI:88 Formula:C12H18O CAS:0-00-0 MolWeight:178 RetIndex:1331
CompName:4-Isopropyl-trans-bicyclo[4.3.0]-2-nonen-8-one, (4R,S)- $$ 5-Isopropyl-1,3,3a,4,5,7a-hexahydro-2H-inden-2-one # $$

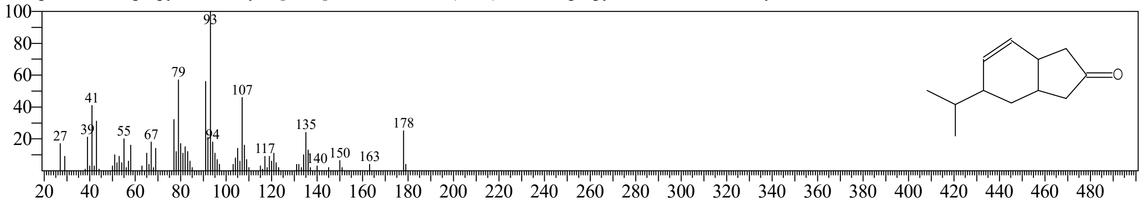

Hit#:5 Entry:33476 Library:NIST17-1.lib
SI:87 Formula:C12H18O CAS:0-00-0 MolWeight:178 RetIndex:1331
CompName:trans-2-Isopropylbicylclo[4.3.0]non-3-ene-8-one $$ 4-Isopropyl-1,3,3a,4,7,7a-hexahydro-2H-inden-2-one # $$

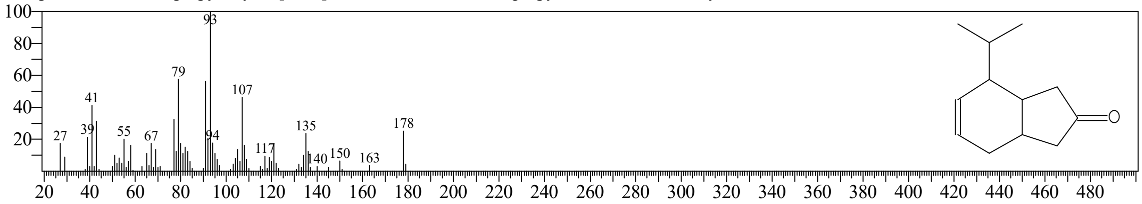

Qualitative Analysis Report

<< Target >>
Line#:10 R.Time:26.796(Scan#:5472) Retention Index:1332 MassPeaks:298
RawMode:Averaged 26.792-26.800(5471-5473) BasePeak:121.15(18594)
BG Mode:Calc. from Peak Group 1 - Event 1 Scan

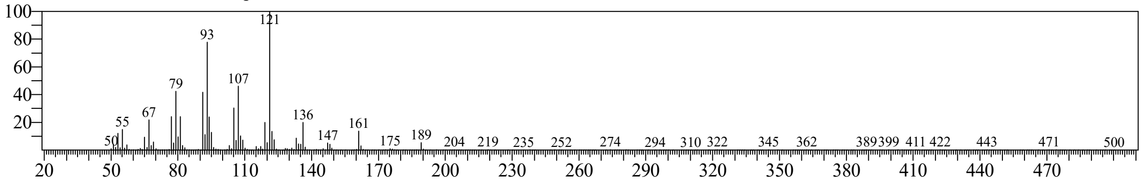

Hit#:1 Entry:1067 Library:FFNSC 3.lib
SI:89 Formula:C15 H24 CAS:370572-92-2 MolWeight:204 RetIndex:1335
CompName:Elemene <delta->

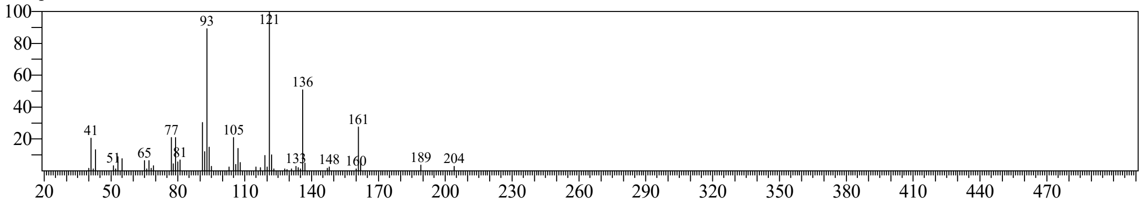

Hit#:2 Entry:52875 Library:NIST17-1.lib
SI:89 Formula:C15H24 CAS:871660-95-6 MolWeight:204 RetIndex:1340
CompName:Panaxene $$ (1R,2S,5S)-2,6,6-Trimethyl-1-(prop-1-en-2-yl)-2-vinylbicyclo[3.2.0]heptane $$ Bicyclo[3.2.0]heptane, 2-ethenyl-2,6,6-trimethyl-1

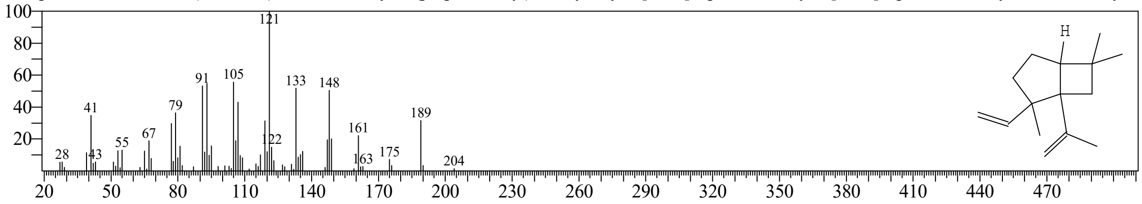

Hit#:3 Entry:71888 Library:NIST17-1.lib
SI:88 Formula:C12H18O2S CAS:0-00-0 MolWeight:226 RetIndex:0
CompName:3,4-Dimethyl-2,5-diprop-2-enyl-2,5-dihydrothiophene 1,1-dioxide

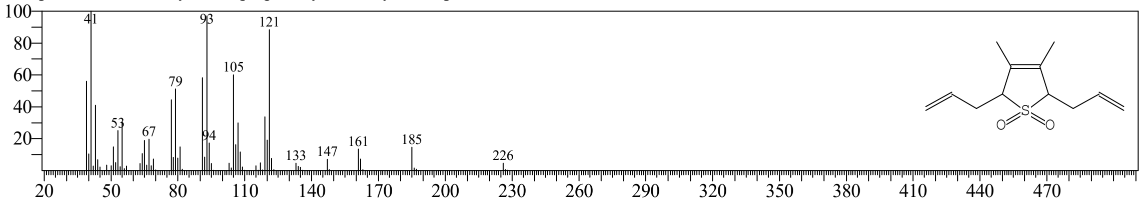

Hit#:4 Entry:24215 Library:NIST17-1.lib
SI:88 Formula:C10H16N2 CAS:0-00-0 MolWeight:164 RetIndex:0
CompName:2,3-Diazabicyclo[2.2.1]hept-2-ene, 5-ethenyl-4,7,7-trimethyl-, (1.alpha.,4.alpha.,5.beta.)- $$ 1,7,7-Trimethyl-6-vinyl-2,3-diazabicyclo[2.2.1]hep

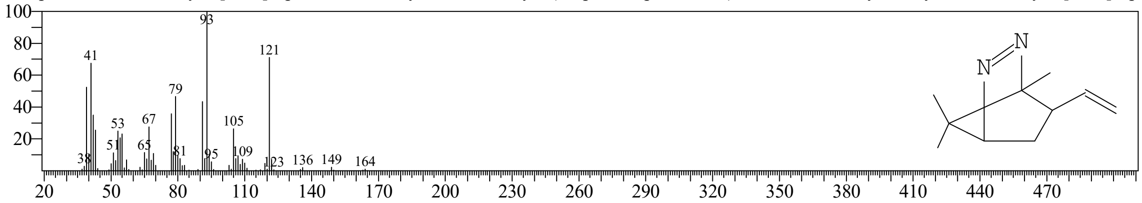

Hit#:5 Entry:10047 Library:NIST17-1.lib
SI:87 Formula:C7H8N2O CAS:1468-29-7 MolWeight:136 RetIndex:0
CompName:Pyridinium, 1-(acetylamino)-, hydroxide, inner salt $$ Pyridinium, 1-acetamido-, hydroxide, inner salt $$ N-Acetyliminopyridinium betaine $$

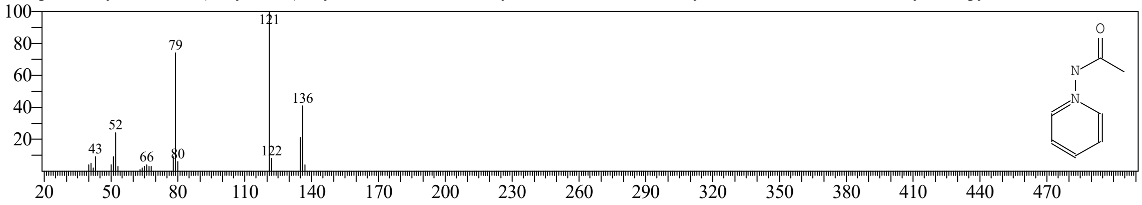

Qualitative Analysis Report

<< Target >>
Line#:11 R.Time:27.442(Scan#:5627) Retention Index:1347 MassPeaks:277
RawMode:Averaged 27.438-27.446(5626-5628) BasePeak:104.10(2081)
BG Mode:Calc. from Peak Group 1 - Event 1 Scan

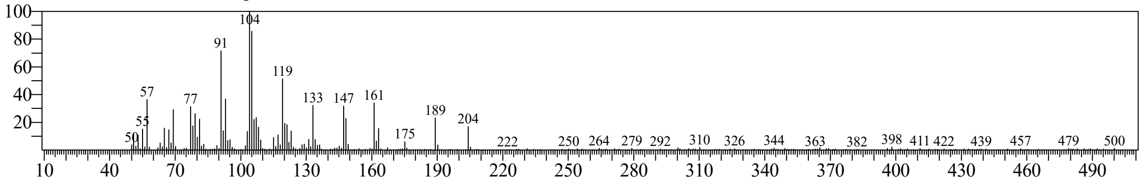

Hit#:1 Entry:45803 Library:NIST17-1.lib
SI:90 Formula:CN4O8 CAS:509-14-8 MolWeight:196 RetIndex:0
CompName:Methane, tetranitro- $$ Tetranitromethane $$ TNM $$ NCI-C55947 $$ Rcra waste number P112 $$ UN 1510 $$

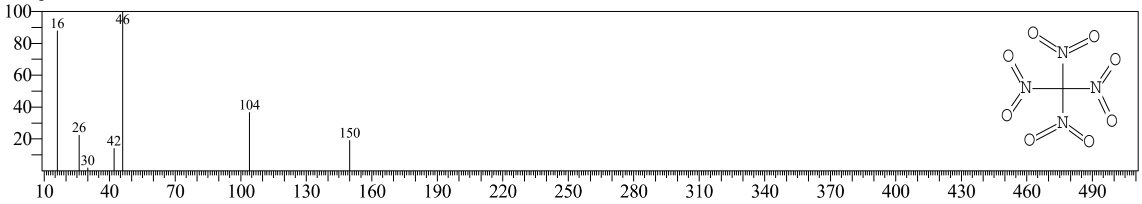

Hit#:2 Entry:23147 Library:NIST17-1.lib
SI:86 Formula:C11H14O CAS:22610-80-6 MolWeight:162 RetIndex:1346
CompName:2-Cyclopenten-1-one, 3-methyl-2-(1,3-pentadienyl)-, (E,Z)- $$ Isopyrethrone $$ 3-Methyl-2-[(1E,3E)-1,3-pentadienyl]-2-cyclopenten-1-one # $

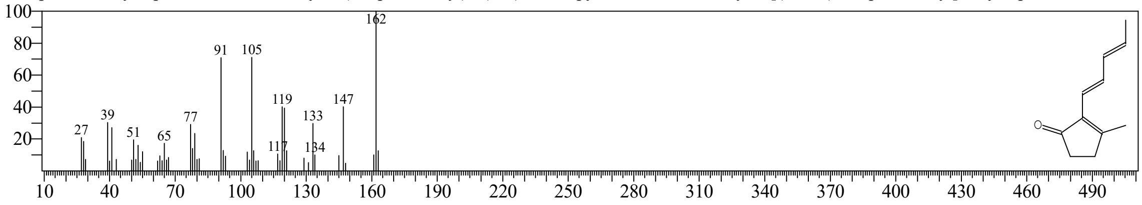

Hit#:3 Entry:2666 Library:NIST17-1.lib
SI:86 Formula:F3OP CAS:13478-20-1 MolWeight:104 RetIndex:0
CompName:Phosphoryl fluoride $$ Phosphorus oxyfluoride $$ Phosphoryl trifluoride $$ Trifluorophosphine oxide $$ Trifluorophosphorus oxide $$ POF3 :

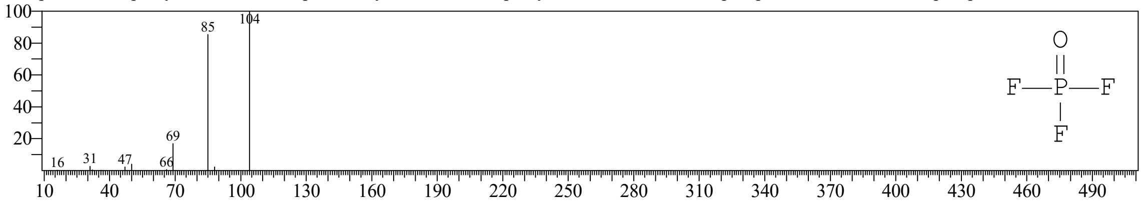

Hit#:4 Entry:6071 Library:NIST17-1.lib
SI:85 Formula:F4H2NP CAS:38047-37-9 MolWeight:123 RetIndex:0
CompName:Phosphoranamine, 1,1,1,1-tetrafluoro-, (TB-5-11)- $$ Amino(tetrafluoro)phosphorane # $$

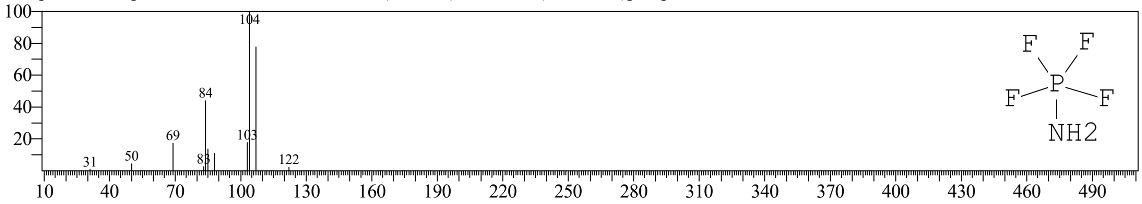

Hit#:5 Entry:117612 Library:NIST17-1.lib
SI:85 Formula:C16H18Zn CAS:74685-32-8 MolWeight:274 RetIndex:0
CompName:Zinc, bis(2-phenylethyl)- $$ Bis(2-phenylethyl)zinc # $$

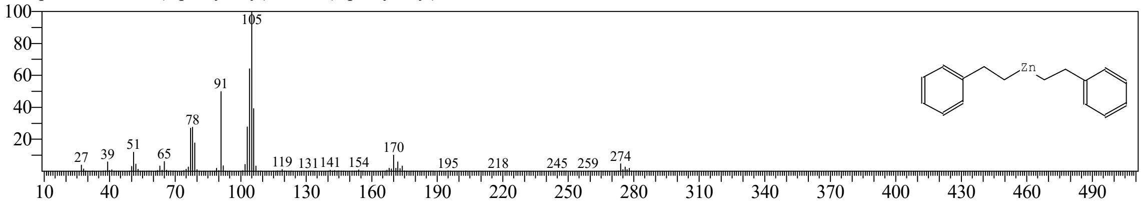

Qualitative Analysis Report

<< Target >>
Line#:12 R.Time:27.983(Scan#:5757) Retention Index:1359 MassPeaks:270
RawMode:Averaged 27.979-27.988(5756-5758) BasePeak:175.15(749)
BG Mode:Calc. from Peak Group 1 - Event 1 Scan

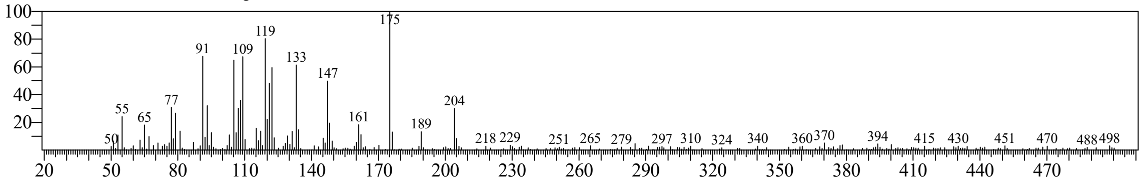

Hit#:1 Entry:2209 Library:FFNSC 3.lib
SI:87 Formula:C15 H24 CAS:138752-23-5 MolWeight:204 RetIndex:1349
CompName:Silphiperfol-5-ene <7-epi->

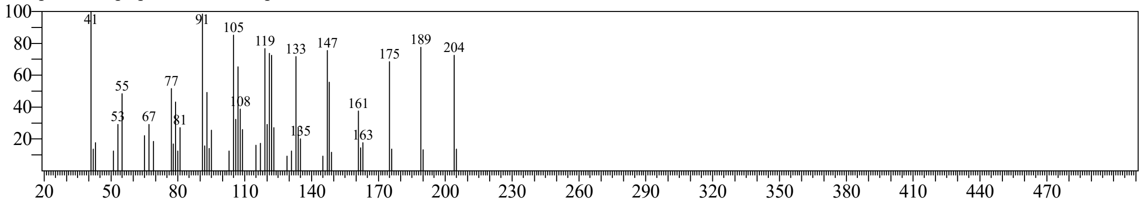

Hit#:2 Entry:41955 Library:NIST17-1.lib
SI:85 Formula:C14H22 CAS:61227-89-2 MolWeight:190 RetIndex:0
CompName:5,6-Decadien-3-yne, 5,7-diethyl- $$ 5,7-Diethyl-5,6-decadien-3-yne # $$

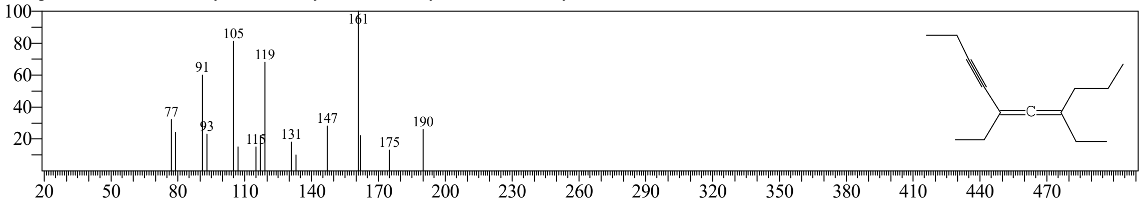

Hit#:3 Entry:41962 Library:NIST17-1.lib
SI:85 Formula:C14H22 CAS:0-00-0 MolWeight:190 RetIndex:1354
CompName:Cycloocta-1,3,6-triene, 2,3,5,5,8,8-hexamethyl- $$ 2,3,5,5,8,8-Hexamethyl-1,3,6-cyclooctatriene # $$

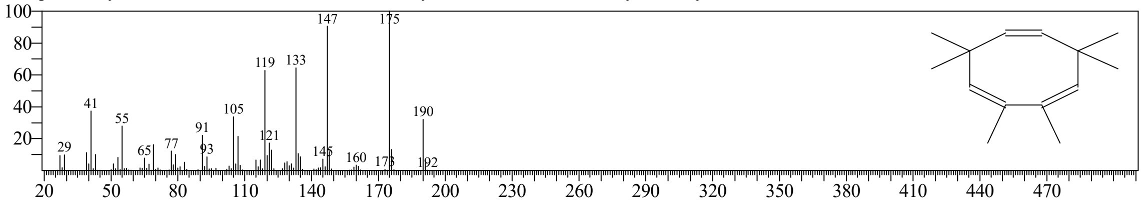

Hit#:4 Entry:223965 Library:NIST17-1.lib
SI:82 Formula:C18H36O5P2 CAS:0-00-0 MolWeight:394 RetIndex:0
CompName:Methylphosphonic acid, anhydride, bis(2,2-dimethylcyclohexyl) ester

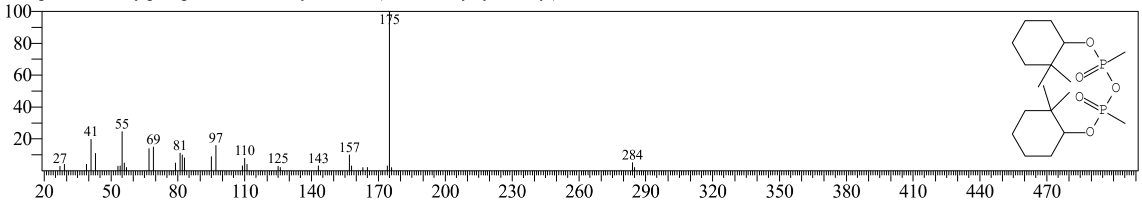

Qualitative Analysis Report

<< Target >>
Line#:13   R.Time:28.408(Scan#:5859) Retention Index:1369 MassPeaks:259
RawMode:Averaged 28.404-28.413(5858-5860) BasePeak:105.10(1082)
BG Mode:Calc. from Peak Group 1 - Event 1 Scan

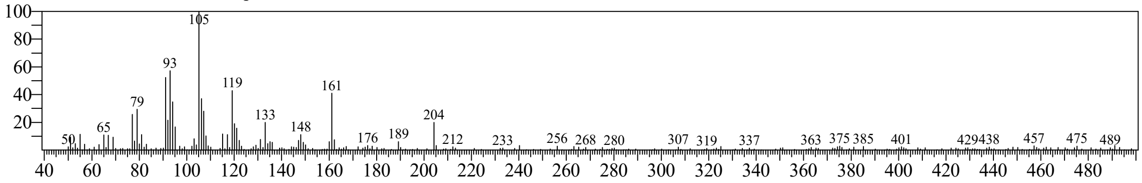

Hit#:1 Entry:1029 Library:FFNSC 3.lib
SI:92 Formula:C15 H24 CAS:30541-92-5 MolWeight:204 RetIndex:1367
CompName:Cyclosativene

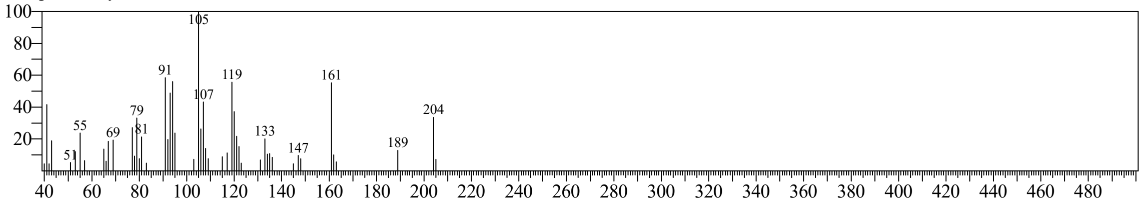

Hit#:2 Entry:245422 Library:NIST17-1.lib
SI:91 Formula:C28H20N4O2 CAS:19226-33-6 MolWeight:444 RetIndex:0
CompName:Benzoic acid, anhydride with N-(4,5-diphenyl-1H-1,2,3-triazol-1-yl)benzenecarboximidic acid $$ Benzoic acid, anhydride with N-(4,5-diphenyl

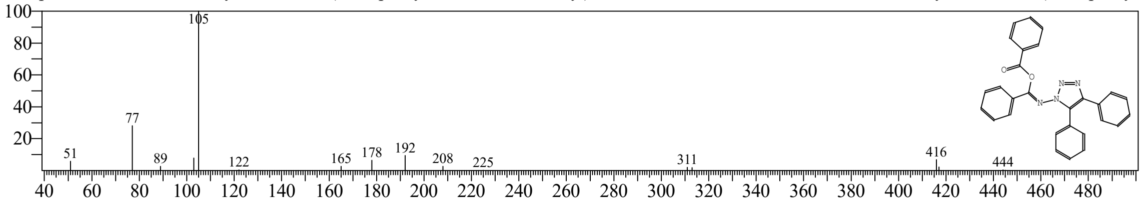

Hit#:3 Entry:1495 Library:FFNSC 3.lib
SI:90 Formula:C15 H24 CAS:14912-44-8 MolWeight:204 RetIndex:1371
CompName:Ylangene <alpha->

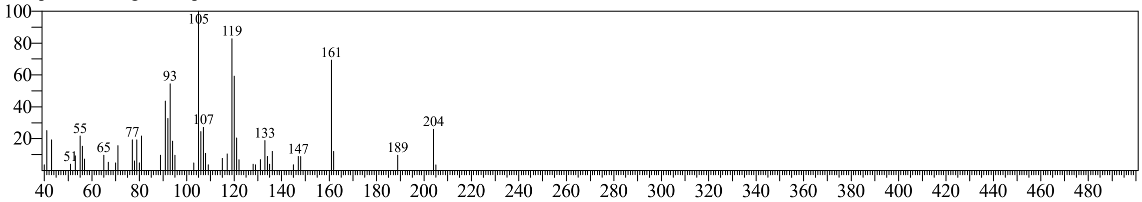

Hit#:4 Entry:1009 Library:FFNSC 3.lib
SI:89 Formula:C15 H24 CAS:138874-68-7 MolWeight:204 RetIndex:1375
CompName:Copaene <alpha->

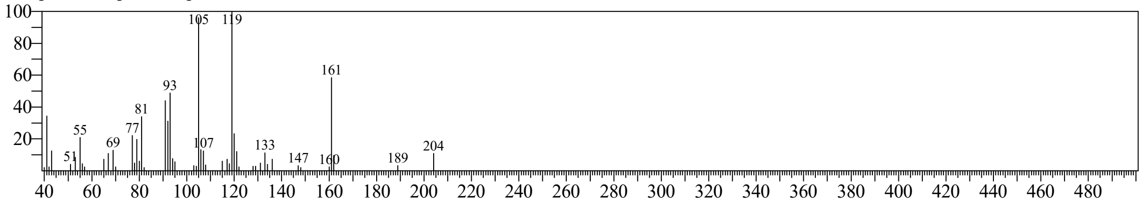

Hit#:5 Entry:163892 Library:NIST17-1.lib
SI:88 Formula:C18H16N4O2 CAS:19226-35-8 MolWeight:320 RetIndex:0
CompName:Benzamide, N-benzoyl-N-(4,5-dimethyl-1H-1,2,3-triazol-1-yl)- $$ Dibenzamide, N-(4,5-dimethyl-1H-1,2,3-triazol-1-yl)- $$ N-Benzoyl-N-(4,5-

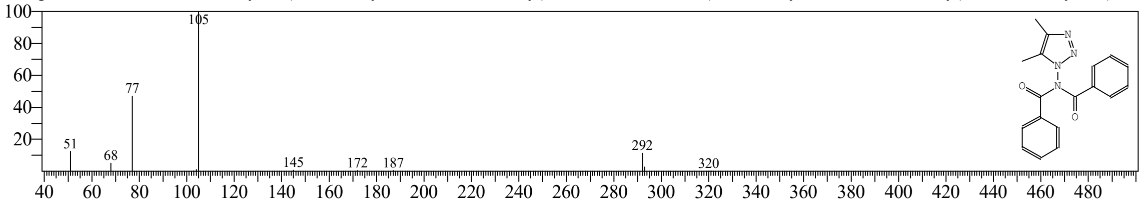

Qualitative Analysis Report

<< Target >>
Line#:14 R.Time:28.717(Scan#:5933) Retention Index:1376 MassPeaks:273
RawMode:Averaged 28.712-28.721(5932-5934) BasePeak:105.10(10788)
BG Mode:Calc. from Peak Group 1 - Event 1 Scan

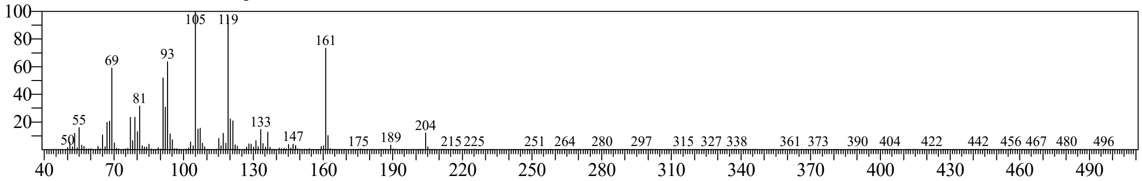

Hit#:1 Entry:1009 Library:FFNSC 3.lib
SI:94 Formula:C15 H24 CAS:138874-68-7 MolWeight:204 RetIndex:1375
CompName:Copaene <alpha->

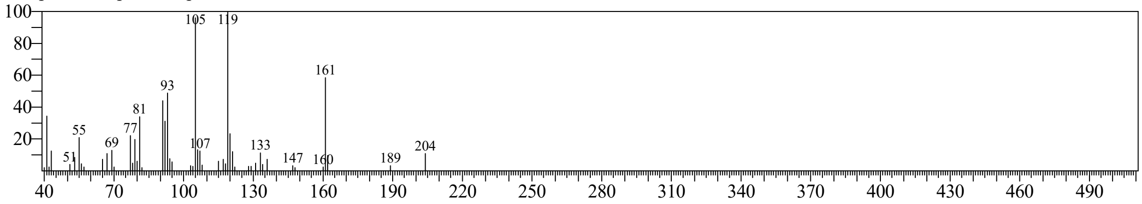

Hit#:2 Entry:1495 Library:FFNSC 3.lib
SI:93 Formula:C15 H24 CAS:14912-44-8 MolWeight:204 RetIndex:1371
CompName:Ylangene <alpha->

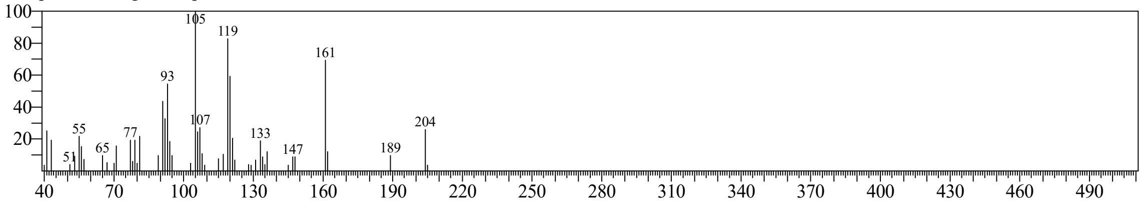

Hit#:3 Entry:22336 Library:NIST17-1.lib
SI:89 Formula:C8H7N3O CAS:1455-84-1 MolWeight:161 RetIndex:1375
CompName:3-Methyl-5-pyrid-2-yl-1,2,4-oxadiazole $$ 2-(3-Methyl-1,2,4-oxadiazol-5-yl)pyridine # $$

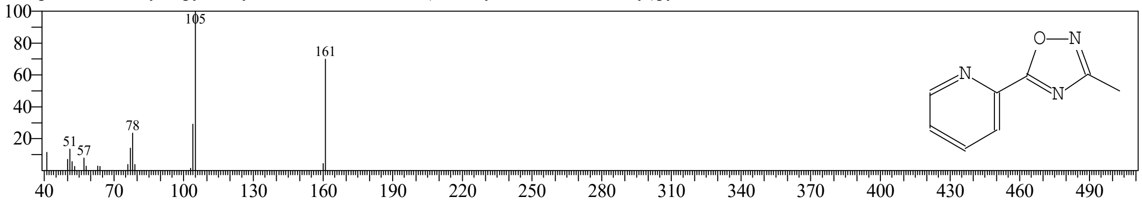

Hit#:4 Entry:41955 Library:NIST17-1.lib
SI:89 Formula:C14H22 CAS:61227-89-2 MolWeight:190 RetIndex:0
CompName:5,6-Decadien-3-yne, 5,7-diethyl- $$ 5,7-Diethyl-5,6-decadien-3-yne # $$

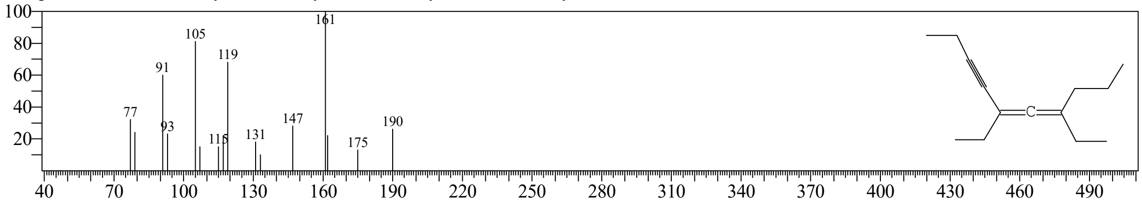

Hit#:5 Entry:1029 Library:FFNSC 3.lib
SI:89 Formula:C15 H24 CAS:30541-92-5 MolWeight:204 RetIndex:1367
CompName:Cyclosativene

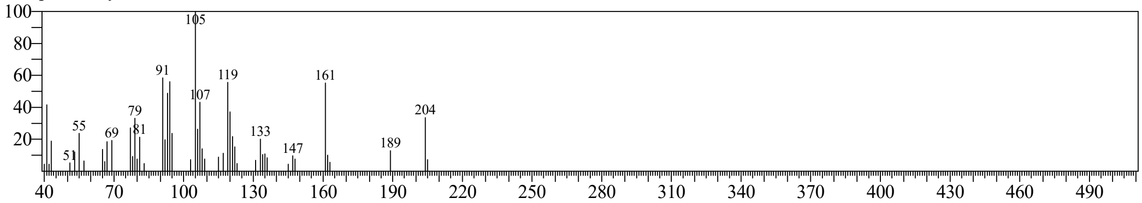

Qualitative Analysis Report

<< Target >>
Line#:15 R.Time:29.021(Scan#:6006) Retention Index:1383 MassPeaks:270
RawMode:Averaged 29.017-29.025(6005-6007) BasePeak:91.10(14674)
BG Mode:Calc. from Peak Group 1 - Event 1 Scan

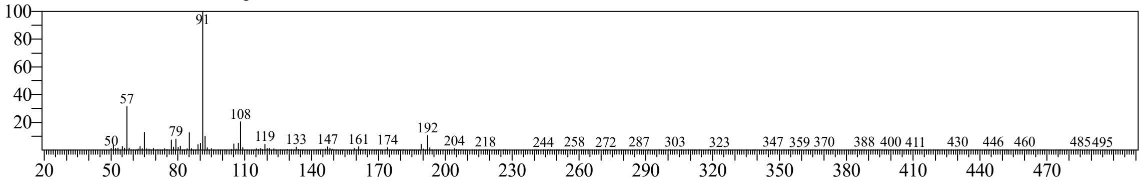

Hit#:1 Entry:168210 Library:NIST17-1.lib
SI:95 Formula:C13H7N7O4 CAS:1394125-25-7 MolWeight:325 RetIndex:0
CompName:12-Benzyl-4,9,15-trioxa-3,5,8,10,12,14,16-heptaazatetracyclo[11.3.0.0(2,6).0(7,11)]hexadeca-1(16),2,5,7,10,13-hexaen-3-one

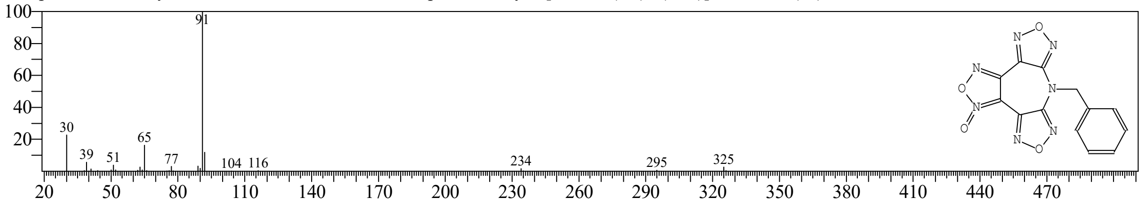

Hit#:2 Entry:15792 Library:NIST17-1.lib
SI:93 Formula:C8H7NS CAS:3012-37-1 MolWeight:149 RetIndex:1389
CompName:Thiocyanic acid, phenylmethyl ester $$ Thiocyanic acid, benzyl ester $$ .alpha.-Thiocyanatotoluene $$ Benzylthiocyanate $$ Solvat 14 $$ Tolu

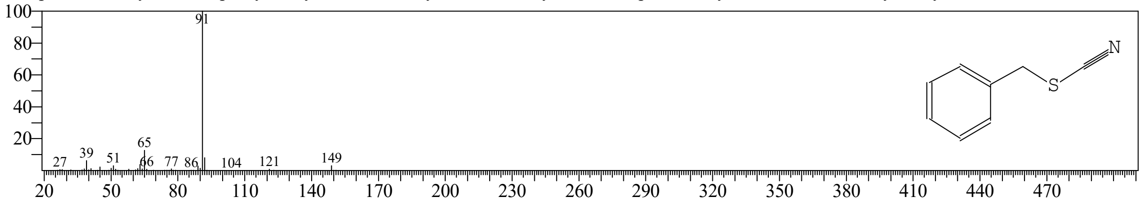

Hit#:3 Entry:217732 Library:NIST17-1.lib
SI:93 Formula:C14H14Hg CAS:780-24-5 MolWeight:384 RetIndex:0
CompName:Dibenzylmercury $$ Mercury dibenzyl $$ Mercury, bis(phenylmethyl)- $$ Mercury, dibenzyl-, $$

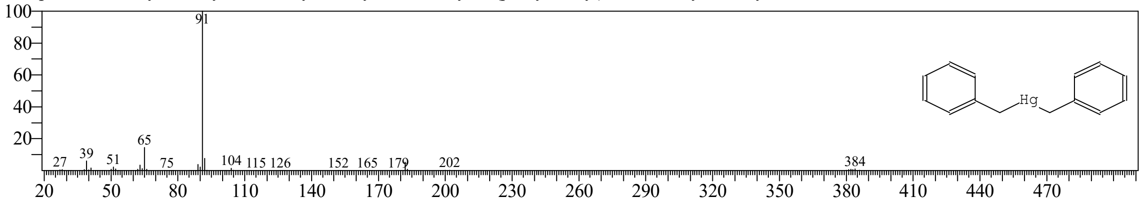

Hit#:4 Entry:59209 Library:NIST17-1.lib
SI:93 Formula:C10H13Br CAS:13633-25-5 MolWeight:212 RetIndex:1388
CompName:Benzene, (4-bromobutyl)- $$ (4-Bromobutyl)benzene $$ 1-Bromo-4-phenylbutane $$ 4-Phenyl-1-bromobutane $$ 4-Phenylbutyl bromide $$

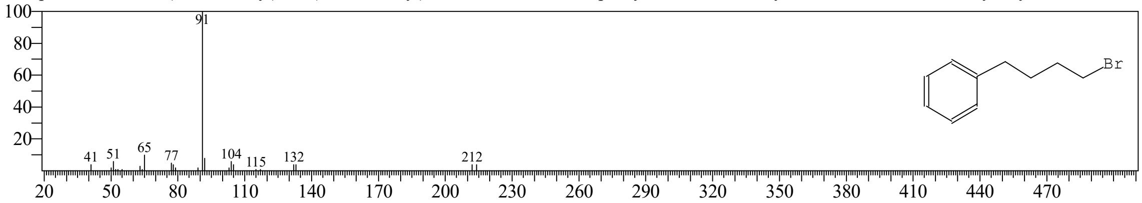

Hit#:5 Entry:22068 Library:NIST17-1.lib
SI:93 Formula:C11H12O CAS:33046-84-3 MolWeight:160 RetIndex:1388
CompName:2-Pentenal, 5-phenyl- $$ 5-Phenyl-2-pentenal $$ (2E)-5-Phenyl-2-pentenal # $$

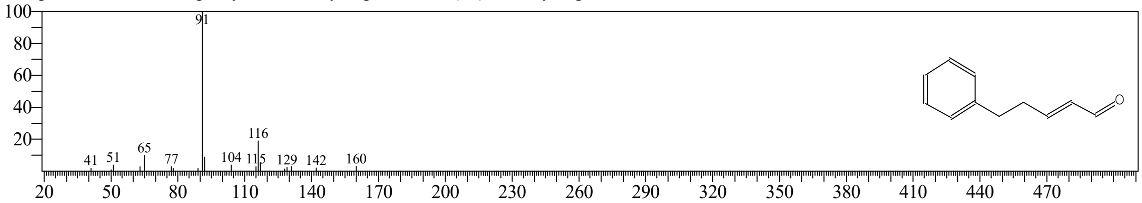

Qualitative Analysis Report

<< Target >>
Line#:16 R.Time:29.279(Scan#:6068) Retention Index:1388 MassPeaks:269
RawMode:Averaged 29.275-29.283(6067-6069) BasePeak:162.15(3789)
BG Mode:Calc. from Peak Group 1 - Event 1 Scan

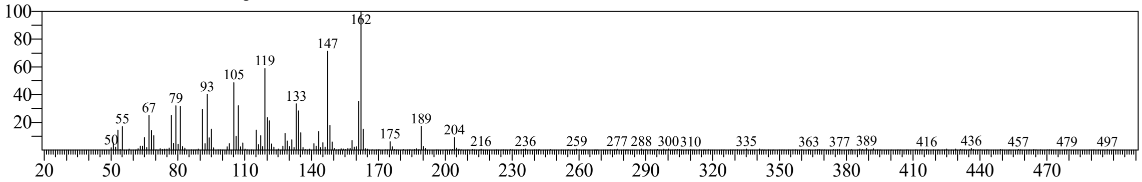

Hit#:1 Entry:2452 Library:FFNSC 3.lib
SI:89 Formula:C15 H24 CAS:65372-78-3 MolWeight:204 RetIndex:1392
CompName:Isocomene <alpha->

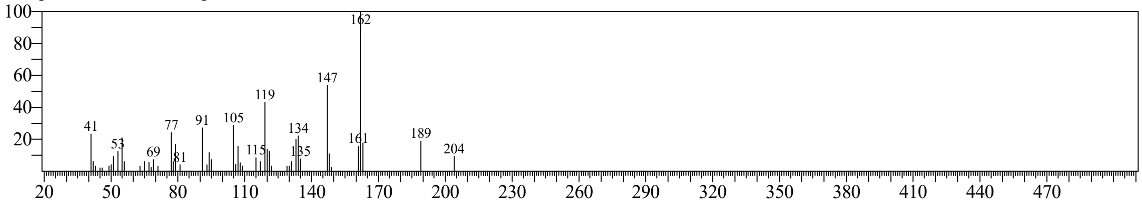

Hit#:2 Entry:41828 Library:NIST17-1.lib
SI:87 Formula:C12H18N2 CAS:0-00-0 MolWeight:190 RetIndex:0
CompName:4,5,6,7-Tetrahydroindazole-3-spirocyclohexane

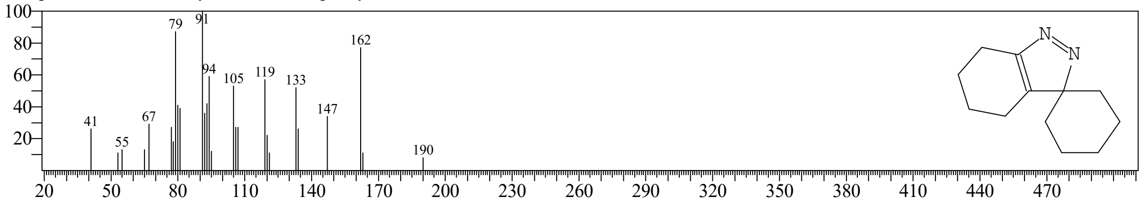

Hit#:3 Entry:52868 Library:NIST17-1.lib
SI:85 Formula:C15H24 CAS:0-00-0 MolWeight:204 RetIndex:1387
CompName:.delta.-Neoclovene

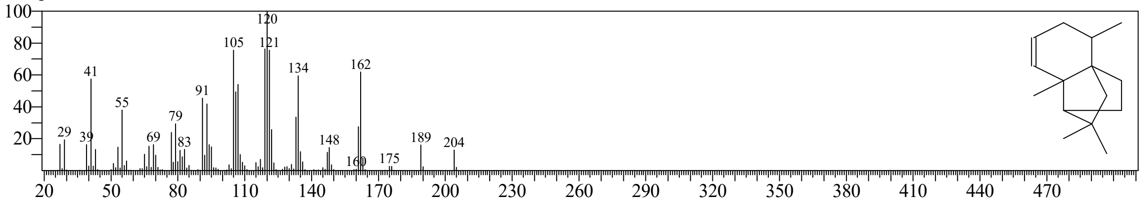

Hit#:4 Entry:35006 Library:NIST17-1.lib
SI:85 Formula:C12H20O CAS:0-00-0 MolWeight:180 RetIndex:1397
CompName:Naphthalene, 2,3,4,4a,5,6,7,8-octahydro-2-hydroxy-[.alpha./.beta.]-2,4a-dimethyl- $$ 2,4a-Dimethyl-2,3,4,4a,5,6,7,8-octahydro-2-naphthalenol

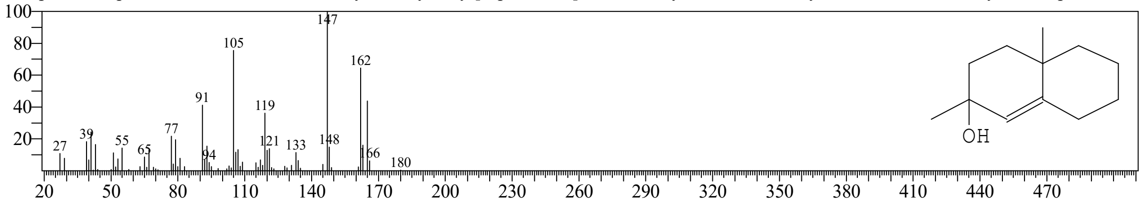

Hit#:5 Entry:1079 Library:FFNSC 3.lib
SI:85 Formula:C15 H24 CAS:514-51-2 MolWeight:204 RetIndex:1383
CompName:Patchoulene <beta->

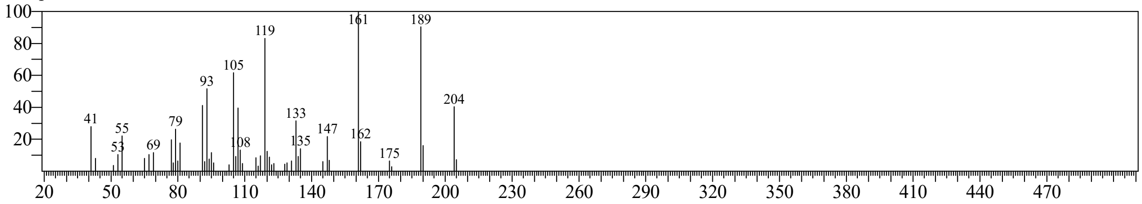

Qualitative Analysis Report

<< Target >>
Line#:17 R.Time:30.238(Scan#:6298) Retention Index:1411 MassPeaks:244
RawMode:Averaged 30.233-30.242(6297-6299) BasePeak:91.10(765)
BG Mode:Calc. from Peak Group 1 - Event 1 Scan

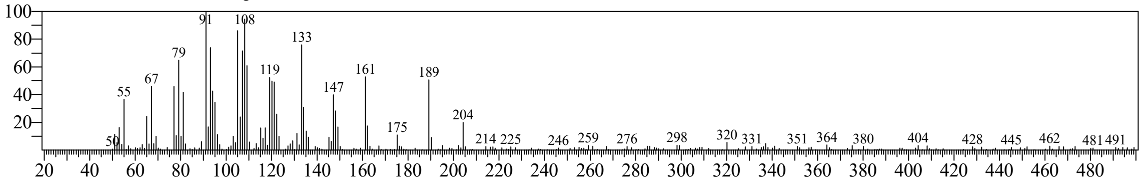

Hit#:1 Entry:52832 Library:NIST17-1.lib
SI:94 Formula:C15H24 CAS:71596-72-0 MolWeight:204 RetIndex:1411
CompName:(1R,3aS,5aS,8aR)-1,3a,5a-Trimethyl-4-methylenedecahydrocyclopenta[c]pentalene $$ Cyclopenta[c]pentalene, decahydro-1,3a,5a-trimethyl-4-r

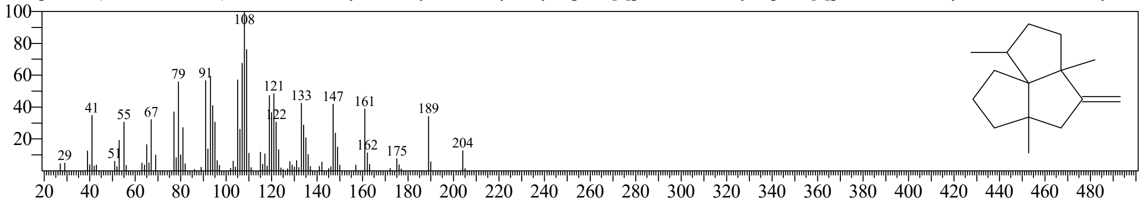

Hit#:2 Entry:31751 Library:NIST17-1.lib
SI:92 Formula:C9H8N2O2 CAS:16844-42-1 MolWeight:176 RetIndex:0
CompName:Sydnone, 3-(phenylmethyl)- $$ Sydnone, 3-benzyl- $$ Benzyl sydnone $$ 3-Benzylsydnone $$ 3-Benzyl-1,2,3-oxadiazol-3-ium-5-olate # $$

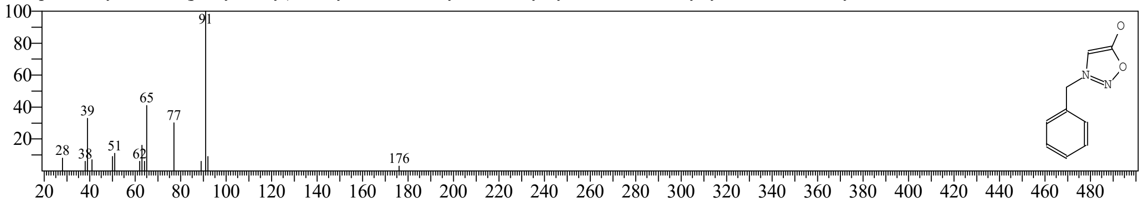

Hit#:3 Entry:1519 Library:FFNSC 3.lib
SI:91 Formula:C15 H24 CAS:475-20-7 MolWeight:204 RetIndex:1412
CompName:Longifolene

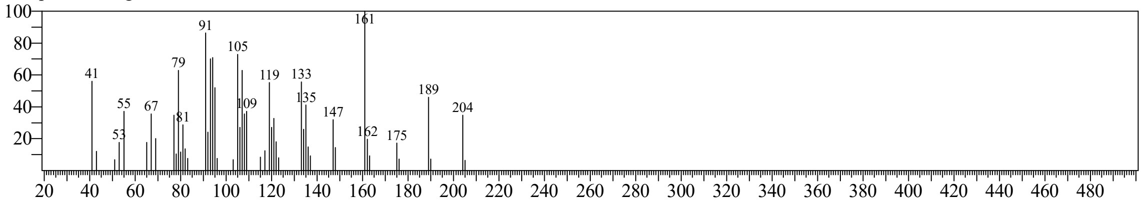

Hit#:4 Entry:52729 Library:NIST17-1.lib
SI:90 Formula:C15H24 CAS:4545-68-0 MolWeight:204 RetIndex:1416
CompName:1,3a-Ethano-3aH-indene, 1,2,3,6,7,7a-hexahydro-2,2,4,7a-tetramethyl-, [1R-(1.alpha.,3a.alpha.,7a.alpha.)]- $$ Neoclovene $$ (-)-.alpha.-Neocl

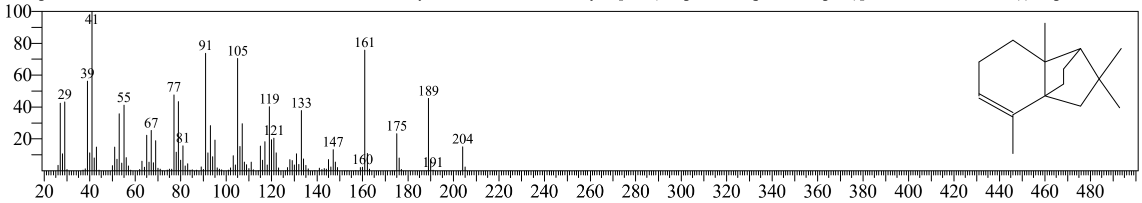

Hit#:5 Entry:52887 Library:NIST17-1.lib
SI:90 Formula:C15H24 CAS:0-00-0 MolWeight:204 RetIndex:1403
CompName:(-)-Tricyclo[6.2.1.0(4,11)]undec-5-ene, 1,5,9,9-tetramethyl- (isocaryophyllene-I1) $$ 2a,4,4,7-Tetramethyl-2,2a,3,4,4a,5,7a,7b-octahydro-1H-cy

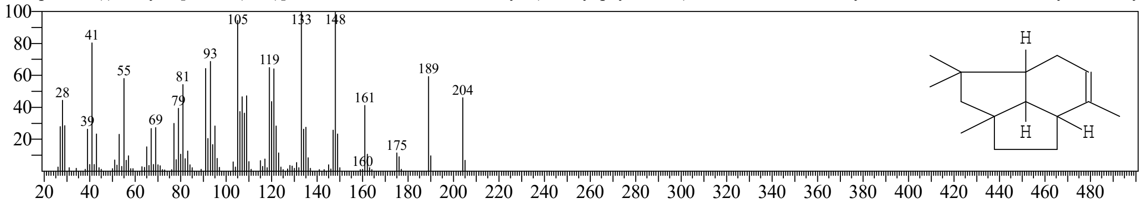

Qualitative Analysis Report

<< Target >>
Line#:18 R.Time:30.542(Scan#:6371) Retention Index:1418 MassPeaks:292
RawMode:Averaged 30.538-30.546(6370-6372) BasePeak:120.10(22648)
BG Mode:Calc. from Peak Group 1 - Event 1 Scan

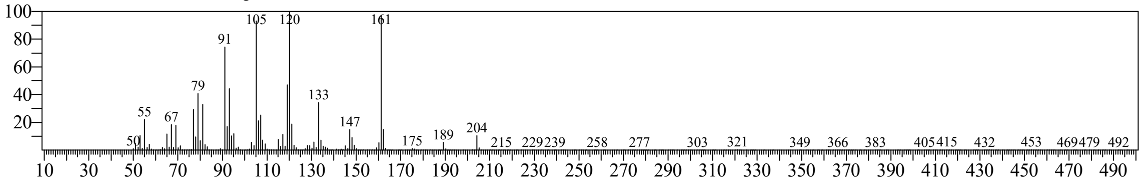

Hit#:1 Entry:2697 Library:FFNSC 3.lib
SI:92 Formula:C15 H24 CAS:20479-06-5 MolWeight:204 RetIndex:1422
CompName:Ylangene<beta->

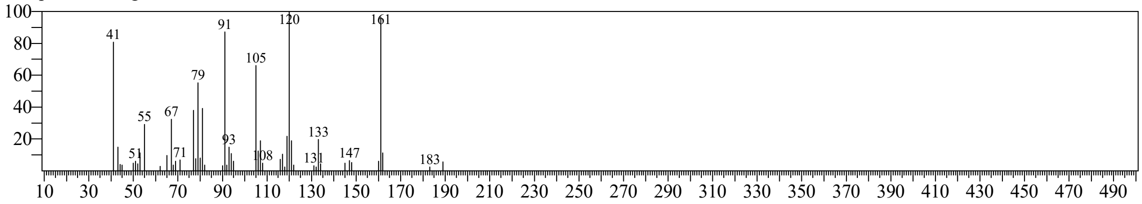

Hit#:2 Entry:52906 Library:NIST17-1.lib
SI:89 Formula:C15H24 CAS:1135-66-6 MolWeight:204 RetIndex:1416
CompName:2H-2,4a-Methanonaphthalene, 1,3,4,5,6,7-hexahydro-1,1,5,5-tetramethyl-, (2S)- $$ 2H-2,4a-Methanonaphthalene, 1,3,4,5,6,7-hexahydro-1,1,5,

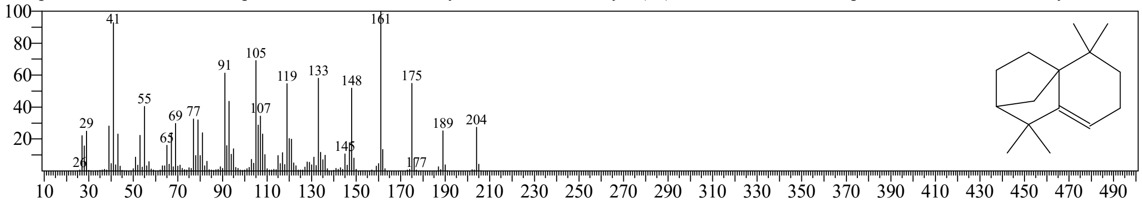

Hit#:3 Entry:52975 Library:NIST17-1.lib
SI:87 Formula:C15H24 CAS:489-40-7 MolWeight:204 RetIndex:1419
CompName:1H-Cycloprop[e]azulene, 1a,2,3,4,4a,5,6,7b-octahydro-1,1,4,7-tetramethyl-, [1aR-(1a.alpha.,4.alpha.,4a.beta.,7b.alpha.)]- $$ 1H-Cycloprop[e]a

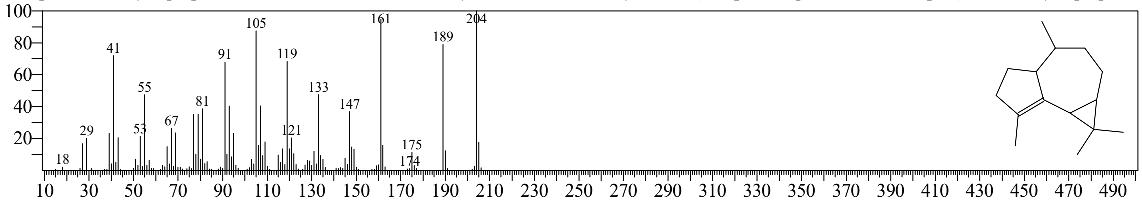

Hit#:4 Entry:52729 Library:NIST17-1.lib
SI:87 Formula:C15H24 CAS:4545-68-0 MolWeight:204 RetIndex:1416
CompName:1,3a-Ethano-3aH-indene, 1,2,3,6,7,7a-hexahydro-2,2,4,7a-tetramethyl-, [1R-(1.alpha.,3a.alpha.,7a.alpha.)]- $$ Neoclovene $$ (-)-.alpha.-Neocl

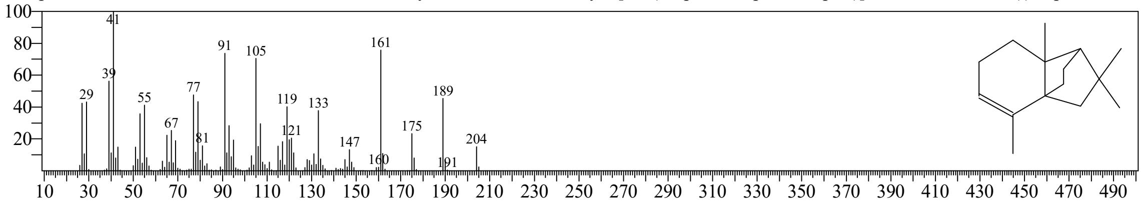

Hit#:5 Entry:723 Library:FFNSC 3.lib
SI:87 Formula:C15 H24 CAS:97499-08-6 MolWeight:204 RetIndex:1423
CompName:Cedrene <beta->

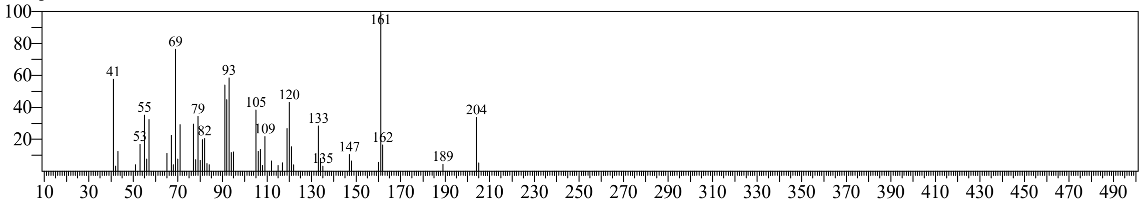

Qualitative Analysis Report

<< Target >>
Line#:19 R.Time:31.029(Scan#:6488) Retention Index:1430 MassPeaks:276
RawMode:Averaged 31.025-31.033(6487-6489) BasePeak:161.15(24401)
BG Mode:Calc. from Peak Group 1 - Event 1 Scan

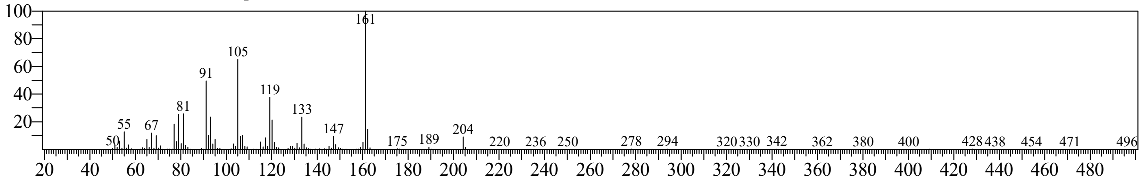

Hit#:1 Entry:2064 Library:FFNSC 3.lib
SI:96 Formula:C15 H24 CAS:147515-11-5 MolWeight:204 RetIndex:1433
CompName:Copaene <beta->

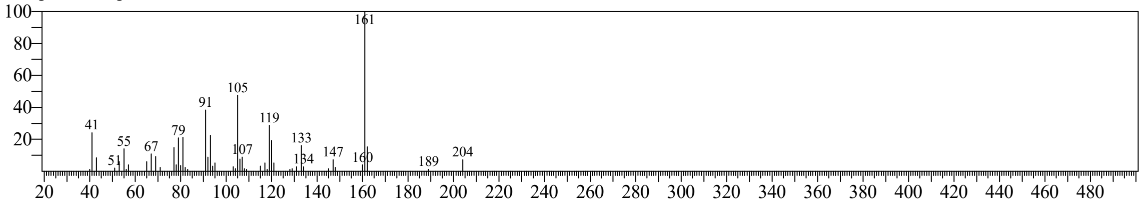

Hit#:2 Entry:52925 Library:NIST17-1.lib
SI:92 Formula:C15H24 CAS:6980-46-7 MolWeight:204 RetIndex:1435
CompName:(1S,4aR,8aS)-1-Isopropyl-7-methyl-4-methylene-1,2,3,4,4a,5,6,8a-octahydronaphthalene $$ .gamma.-Amorphene $$

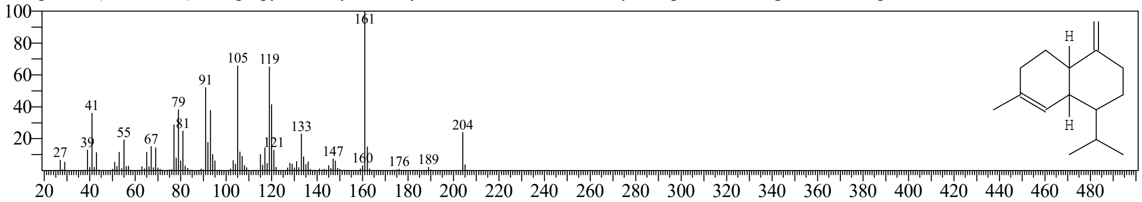

Hit#:3 Entry:52924 Library:NIST17-1.lib
SI:91 Formula:C15H24 CAS:30021-74-0 MolWeight:204 RetIndex:1435
CompName:.gamma.-Muurolene $$ Naphthalene, 1,2,3,4,4a,5,6,8a-octahydro-7-methyl-4-methylene-1-(1-methylethyl)-, (1.alpha.,4a.alpha.,8a.alpha.)- $$ 1-

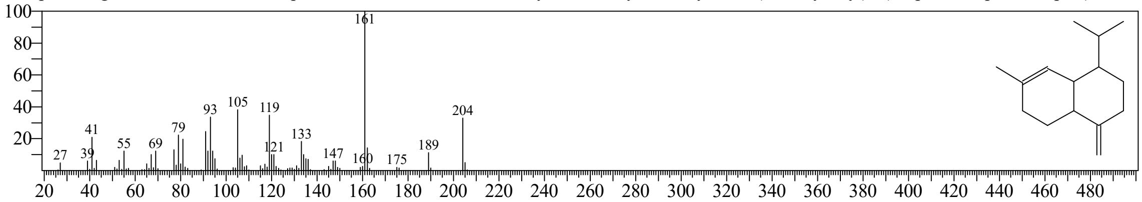

Hit#:4 Entry:1464 Library:FFNSC 3.lib
SI:91 Formula:C15 H24 CAS:79433-67-3 MolWeight:204 RetIndex:1434
CompName:Calarene

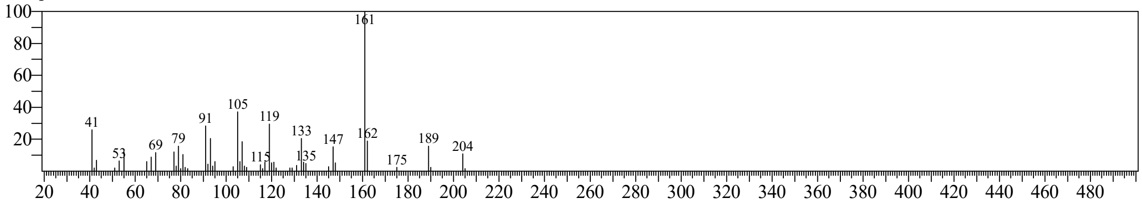

Hit#:5 Entry:52921 Library:NIST17-1.lib
SI:91 Formula:C15H24 CAS:0-00-0 MolWeight:204 RetIndex:1440
CompName:cis-muurola-3,5-diene

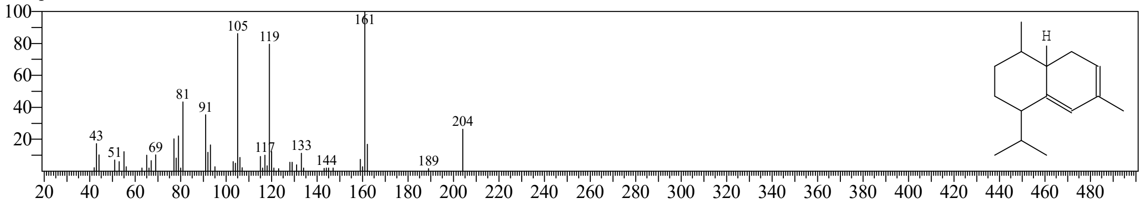

Qualitative Analysis Report

<< Target >>
Line#:20 R.Time:31.400(Scan#:6577) Retention Index:1439 MassPeaks:306
RawMode:Averaged 31.396-31.404(6576-6578) BasePeak:91.10(603)
BG Mode:Calc. from Peak Group 1 - Event 1 Scan

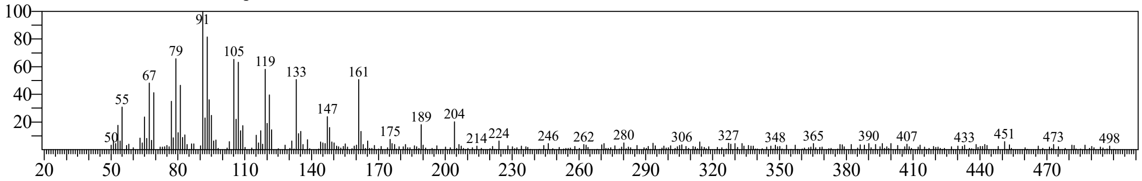

Hit#:1 Entry:1031 Library:FFNSC 3.lib
SI:94 Formula:C15 H24 CAS:109119-91-7 MolWeight:204 RetIndex:1438
CompName:Aromadendrene

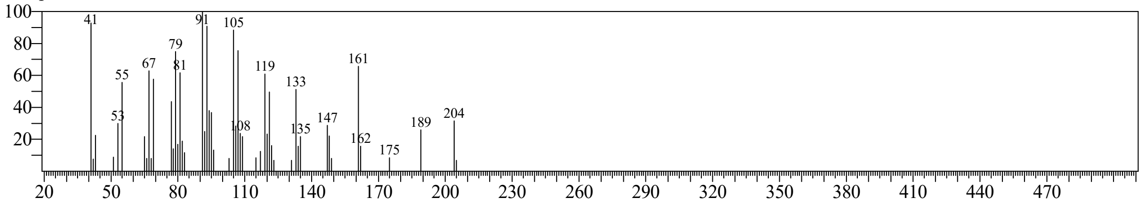

Hit#:2 Entry:1473 Library:FFNSC 3.lib
SI:92 Formula:C15 H24 CAS:3691-12-1 MolWeight:204 RetIndex:1438
CompName:Guaiene <alpha->

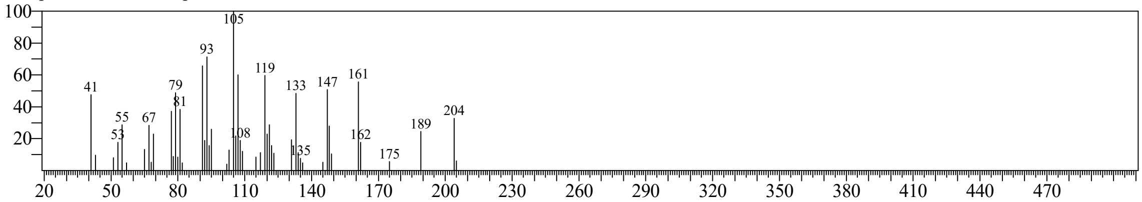

Hit#:3 Entry:570 Library:FFNSC 3.lib
SI:92 Formula:C15 H24 CAS:80923-88-2 MolWeight:204 RetIndex:1449
CompName:Himachalene <alpha->

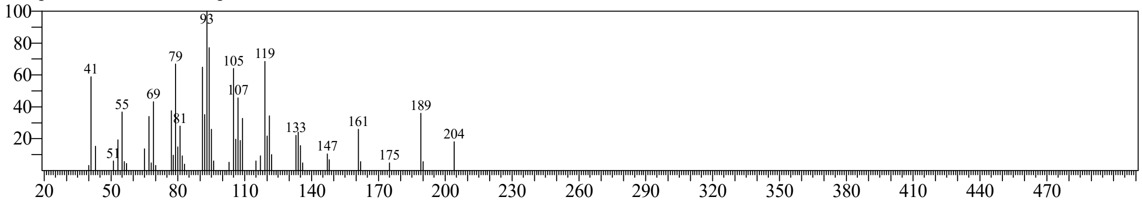

Hit#:4 Entry:52646 Library:NIST17-1.lib
SI:91 Formula:C13H20N2 CAS:0-00-0 MolWeight:204 RetIndex:0
CompName:1,4-Methanocycloocta[d]pyridazine, 1,4,4a,5,6,9,10,10a-octahydro-11,11-dimethyl-, (1.alpha.,4.alpha.,4a.alpha.,10a.alpha.)-

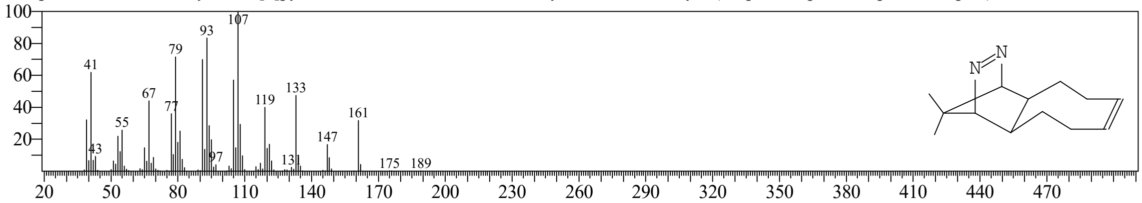

Hit#:5 Entry:31751 Library:NIST17-1.lib
SI:91 Formula:C9H8N2O2 CAS:16844-42-1 MolWeight:176 RetIndex:0
CompName:Sydnone, 3-(phenylmethyl)- $$ Sydnone, 3-benzyl- $$ Benzyl sydnone $$ 3-Benzylsydnone $$ 3-Benzyl-1,2,3-oxadiazol-3-ium-5-olate # $$

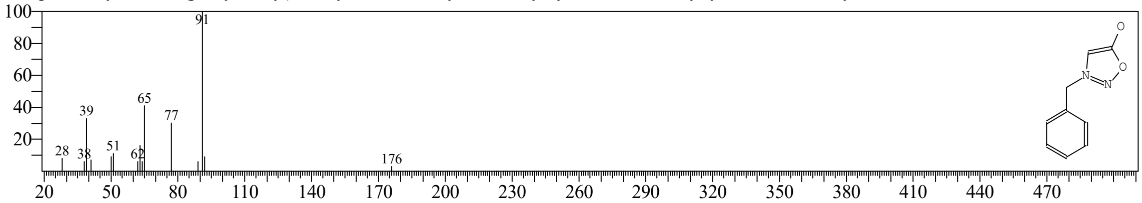

Qualitative Analysis Report

<< Target >>
Line#:21 R.Time:31.625(Scan#:6631) Retention Index:1444 MassPeaks:249
RawMode:Averaged 31.621-31.629(6630-6632) BasePeak:105.10(4466)
BG Mode:Calc. from Peak Group 1 - Event 1 Scan

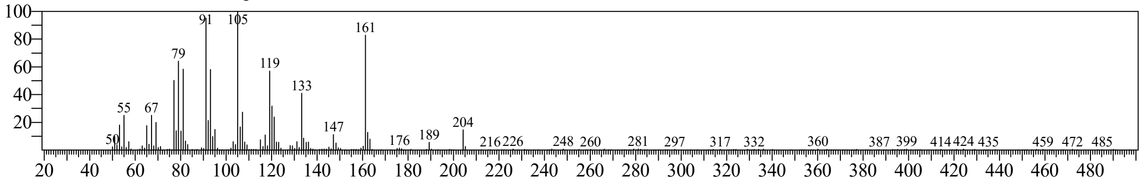

Hit#:1 Entry:1676 Library:FFNSC 3.lib
SI:95 Formula:C15 H24 CAS:317819-80-0 MolWeight:204 RetIndex:1447
CompName:Isogermacrene D

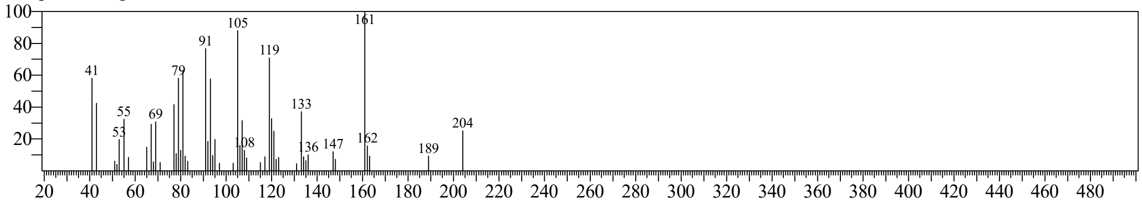

Hit#:2 Entry:163892 Library:NIST17-1.lib
SI:94 Formula:C18H16N4O2 CAS:19226-35-8 MolWeight:320 RetIndex:0
CompName:Benzamide, N-benzoyl-N-(4,5-dimethyl-1H-1,2,3-triazol-1-yl)- $$ Dibenzamide, N-(4,5-dimethyl-1H-1,2,3-triazol-1-yl)- $$ N-Benzoyl-N-(4,5-

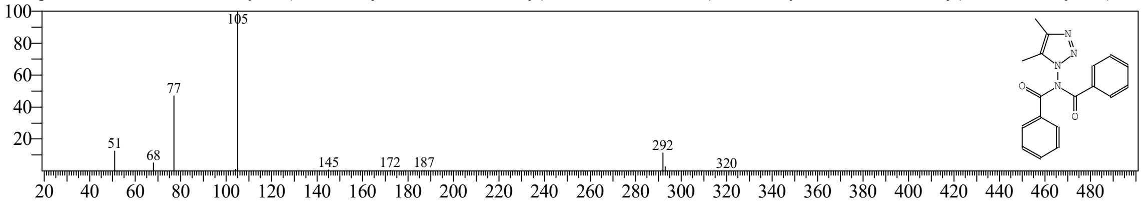

Hit#:3 Entry:1473 Library:FFNSC 3.lib
SI:91 Formula:C15 H24 CAS:3691-12-1 MolWeight:204 RetIndex:1438
CompName:Guaiene <alpha->

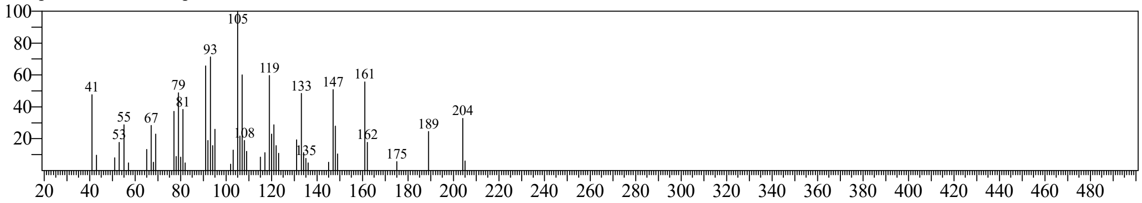

Hit#:4 Entry:52925 Library:NIST17-1.lib
SI:90 Formula:C15H24 CAS:6980-46-7 MolWeight:204 RetIndex:1435
CompName:(1S,4aR,8aS)-1-Isopropyl-7-methyl-4-methylene-1,2,3,4,4a,5,6,8a-octahydronaphthalene $$ .gamma.-Amorphene $$

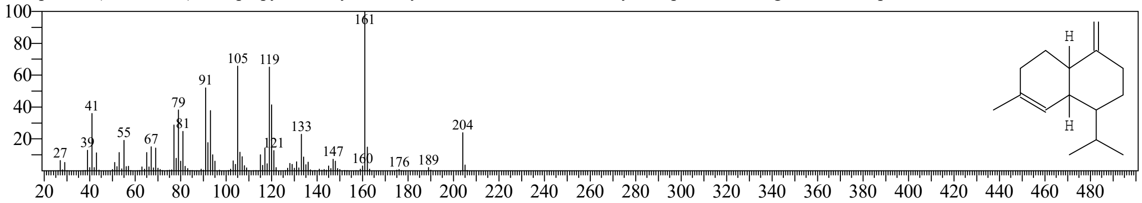

Hit#:5 Entry:2758 Library:FFNSC 3.lib
SI:90 Formula:C15 H24 CAS:246243-00-5 MolWeight:204 RetIndex:1452
CompName:Spirolepechinene

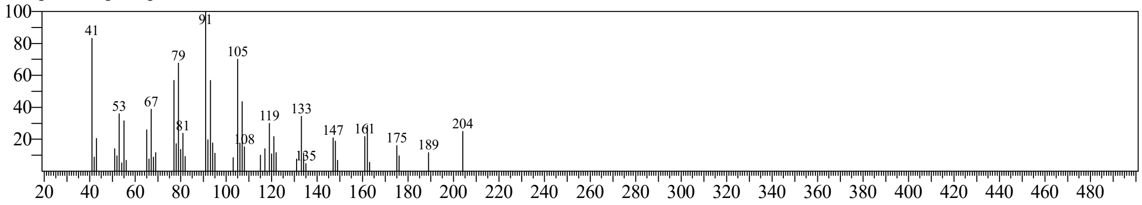

Qualitative Analysis Report

<< Target >>
Line#:22 R.Time:31.975(Scan#:6715) Retention Index:1452 MassPeaks:323
RawMode:Averaged 31.971-31.979(6714-6716) BasePeak:69.10(543034)
BG Mode:Calc. from Peak Group 1 - Event 1 Scan

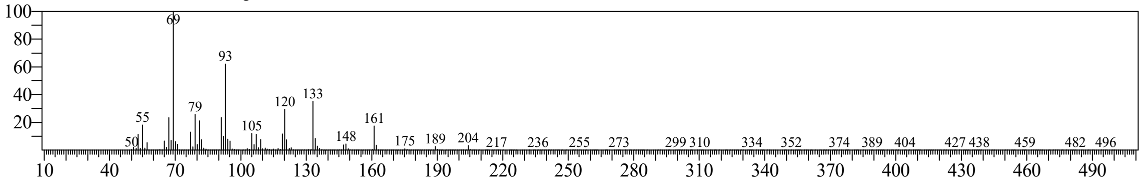

Hit#:1 Entry:228 Library:NIST17-1.lib
SI:99 Formula:C2H3N3 CAS:288-36-8 MolWeight:69 RetIndex:0
CompName:1H-1,2,3-Triazole $$ V-triazole $$ Osotriazole $$ Pyrrodiazole $$ Triazacyclopentadiene $$ 1,2,3-Triazole $$

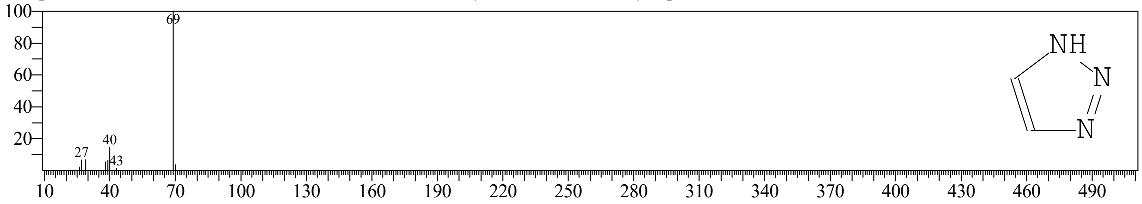

Hit#:2 Entry:1107 Library:FFNSC 3.lib
SI:96 Formula:C15 H24 CAS:18794-84-8 MolWeight:204 RetIndex:1452
CompName:Farnesene <(E)-, beta->

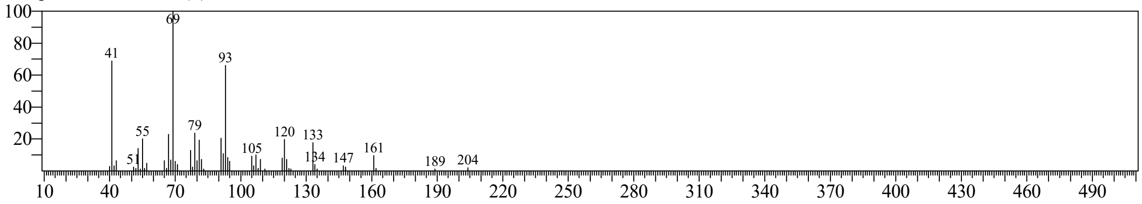

Hit#:3 Entry:981 Library:NIST17-1.lib
SI:96 Formula:CF4 CAS:75-73-0 MolWeight:88 RetIndex:0
CompName:Tetrafluoromethane $$ Carbon tetrafluoride $$ Methane, tetrafluoro- $$ Arcton 0 $$ Carbon fluoride (CF4) $$ Freon 14 $$ FC 14 $$ Perfluoro

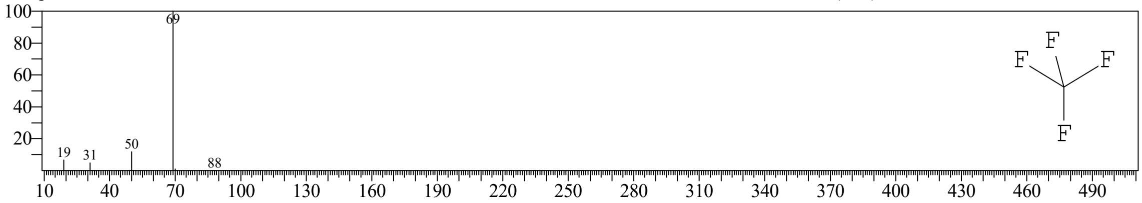

Hit#:4 Entry:10455 Library:NIST17-1.lib
SI:94 Formula:CClF4N CAS:13880-72-3 MolWeight:137 RetIndex:0
CompName:N-Chloro-N-fluoro-trifluoromethylamine $$ N-Chloro-N-tetrafluoromethanamine # $$

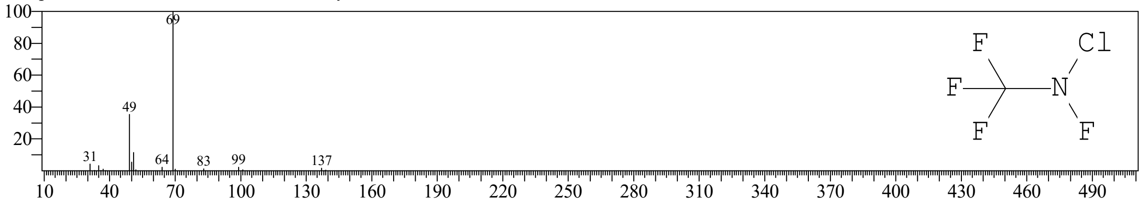

Hit#:5 Entry:13581 Library:NIST17-1.lib
SI:93 Formula:C6H12N2O2 CAS:108201-44-1 MolWeight:144 RetIndex:0
CompName:Diazene, 1-cyclopentyl-2-methoxy-, 1-oxide $$ 1-Cyclopentyl-2-methoxydiazene 1-oxide # $$

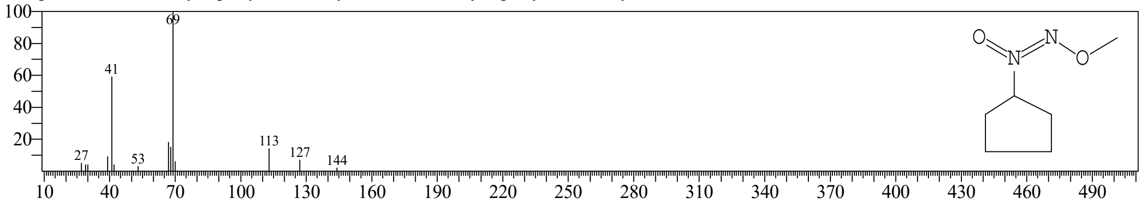

Qualitative Analysis Report

<< Target >>
Line#:23 R.Time:32.329(Scan#:6800) Retention Index:1461 MassPeaks:297
RawMode:Averaged 32.325-32.333(6799-6801) BasePeak:161.15(3143)
BG Mode:Calc. from Peak Group 1 - Event 1 Scan

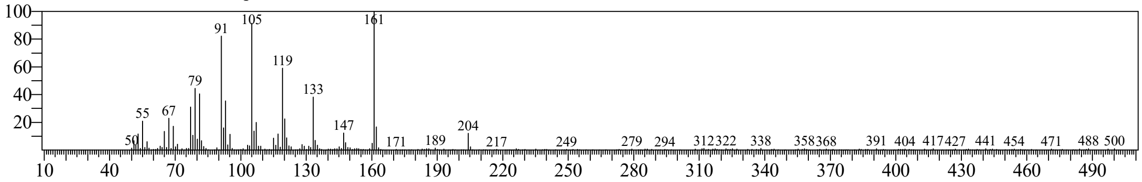

Hit#:1 Entry:41955 Library:NIST17-1.lib
SI:91 Formula:C14H22 CAS:61227-89-2 MolWeight:190 RetIndex:0
CompName:5,6-Decadien-3-yne, 5,7-diethyl- $$ 5,7-Diethyl-5,6-decadien-3-yne # $$

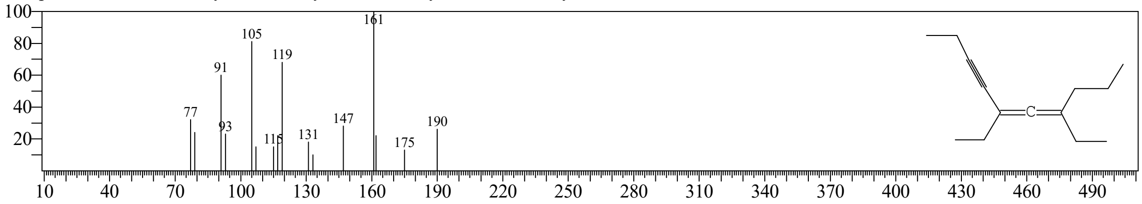

Hit#:2 Entry:2656 Library:FFNSC 3.lib
SI:90 Formula:C15 H24 CAS:189165-77-3 MolWeight:204 RetIndex:1453
CompName:Muurola-3,5-diene<trans->

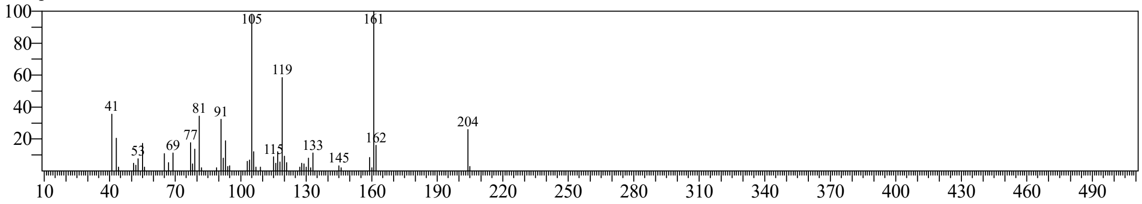

Hit#:3 Entry:52936 Library:NIST17-1.lib
SI:89 Formula:C15H24 CAS:16729-01-4 MolWeight:204 RetIndex:1469
CompName:1-Isopropyl-4,7-dimethyl-1,2,3,5,6,8a-hexahydronaphthalene $$ Cadina-1(10),4-diene $$

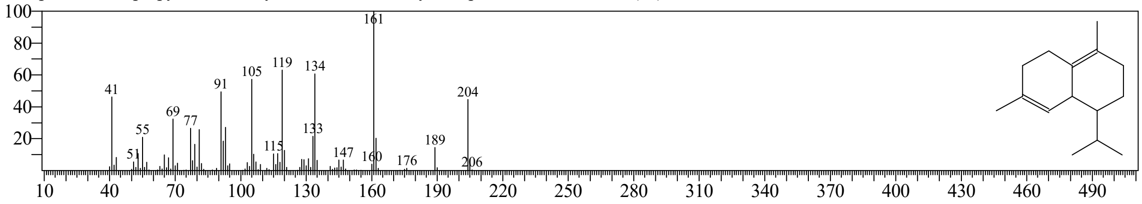

Hit#:4 Entry:52910 Library:NIST17-1.lib
SI:88 Formula:C15H24 CAS:41929-05-9 MolWeight:204 RetIndex:1469
CompName:Zonarene $$ (1R,8aR)-4-Isopropyl-1,6-dimethyl-1,2,3,7,8,8a-hexahydronaphthalene $$ Naphthalene, 1,2,3,7,8,8a-hexahydro-1,6-dimethyl-4-(1

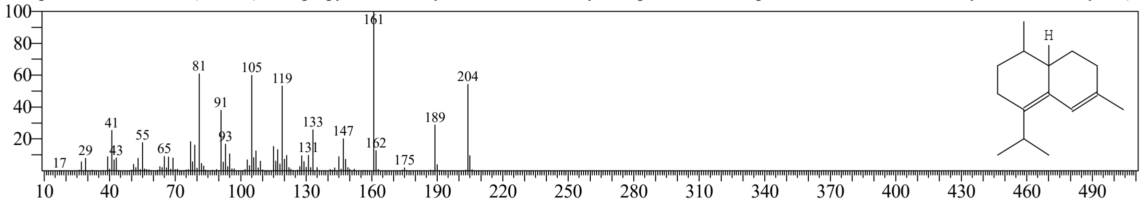

Hit#:5 Entry:2940 Library:FFNSC 3.lib
SI:87 Formula:C15 H24 CAS:4545-68-0 MolWeight:204 RetIndex:1454
CompName:Clovene<alpha-neo->

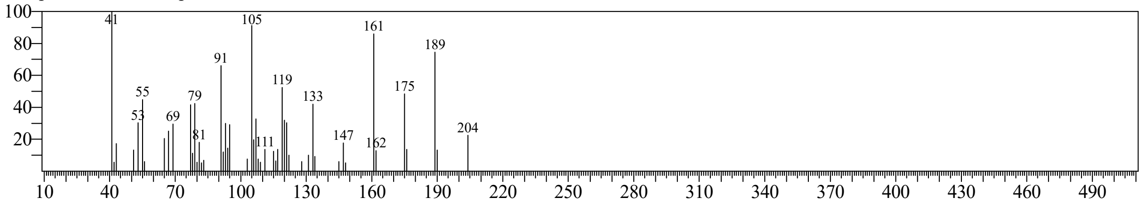

Qualitative Analysis Report

<< Target >>
Line#:24 R.Time:32.917(Scan#:6941) Retention Index:1475 MassPeaks:282
RawMode:Averaged 32.913-32.921(6940-6942) BasePeak:161.15(9615)
BG Mode:Calc. from Peak Group 1 - Event 1 Scan

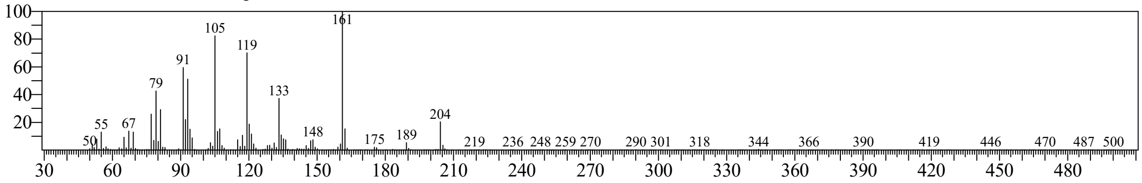

Hit#:1 Entry:1474 Library:FFNSC 3.lib
SI:96 Formula:C15 H24 CAS:30021-74-0 MolWeight:204 RetIndex:1478
CompName:Muurolene <gamma->

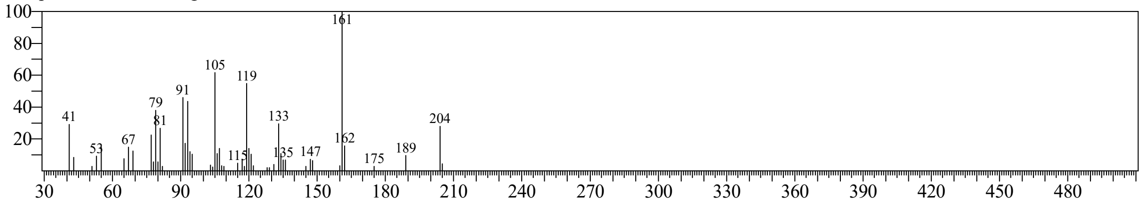

Hit#:2 Entry:41955 Library:NIST17-1.lib
SI:92 Formula:C14H22 CAS:61227-89-2 MolWeight:190 RetIndex:0
CompName:5,6-Decadien-3-yne, 5,7-diethyl- $$ 5,7-Diethyl-5,6-decadien-3-yne # $$

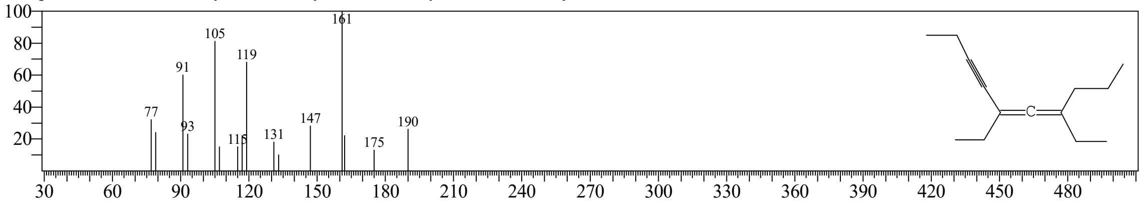

Hit#:3 Entry:969 Library:FFNSC 3.lib
SI:91 Formula:C15 H24 CAS:105453-16-5 MolWeight:204 RetIndex:1480
CompName:Germacrene D

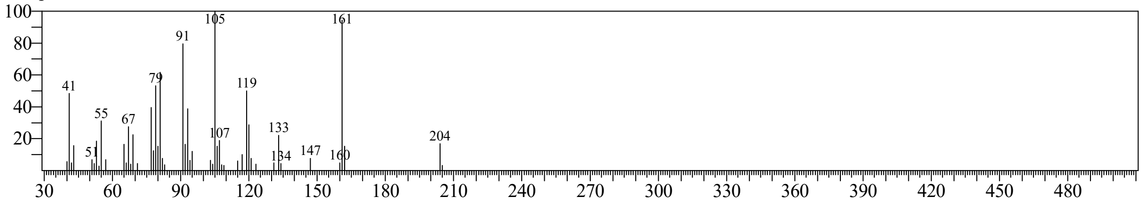

Hit#:4 Entry:52936 Library:NIST17-1.lib
SI:90 Formula:C15H24 CAS:16729-01-4 MolWeight:204 RetIndex:1469
CompName:1-Isopropyl-4,7-dimethyl-1,2,3,5,6,8a-hexahydronaphthalene $$ Cadina-1(10),4-diene $$

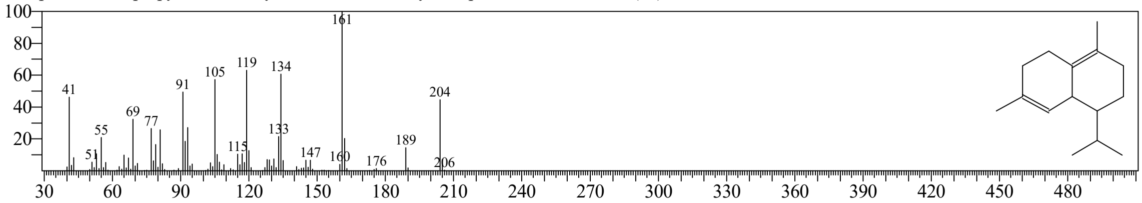

Hit#:5 Entry:52950 Library:NIST17-1.lib
SI:89 Formula:C15H24 CAS:4630-07-3 MolWeight:204 RetIndex:1474
CompName:Naphthalene, 1,2,3,5,6,7,8,8a-octahydro-1,8a-dimethyl-7-(1-methylethenyl)-, [1R-(1.alpha.,7.beta.,8a.alpha.)]- $$ 4.beta.H,5.alpha.-Eremophila-

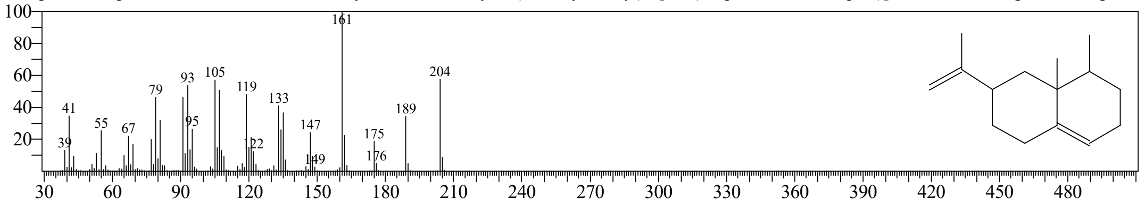

Qualitative Analysis Report

<< Target >>
Line#:25 R.Time:33.192(Scan#:7007) Retention Index:1481 MassPeaks:317
RawMode:Averaged 33.188-33.196(7006-7008) BasePeak:161.15(427570)
BG Mode:Calc. from Peak Group 1 - Event 1 Scan

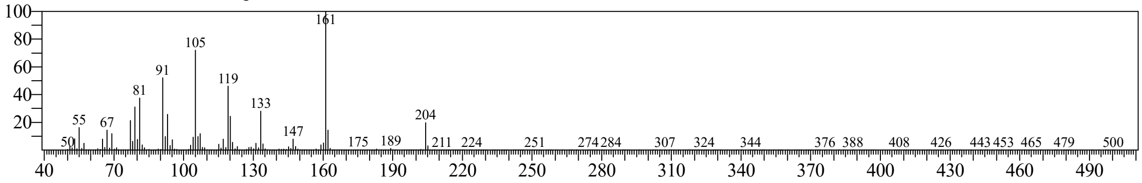

Hit#:1 Entry:1474 Library:FFNSC 3.lib
SI:94 Formula:C15 H24 CAS:30021-74-0 MolWeight:204 RetIndex:1478
CompName:Muurolene <gamma->

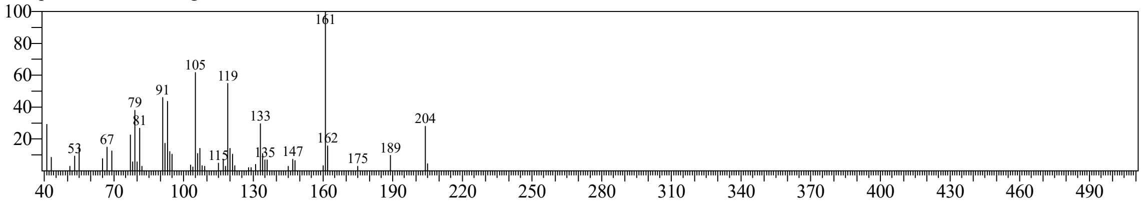

Hit#:2 Entry:2249 Library:FFNSC 3.lib
SI:93 Formula:C15 H24 CAS:142928-08-3 MolWeight:204 RetIndex:1473
CompName:Dauca-5,8-diene

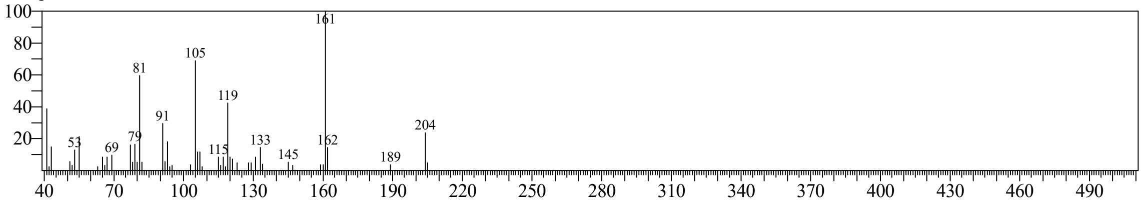

Hit#:3 Entry:1673 Library:FFNSC 3.lib
SI:93 Formula:C15 H24 CAS:6980-46-7 MolWeight:204 RetIndex:1490
CompName:Amorphene <gamma->

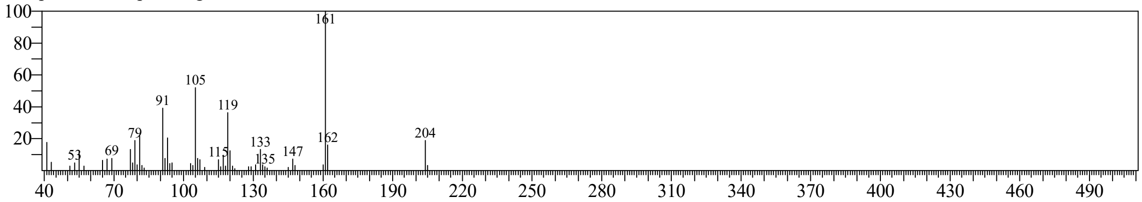

Hit#:4 Entry:969 Library:FFNSC 3.lib
SI:91 Formula:C15 H24 CAS:105453-16-5 MolWeight:204 RetIndex:1480
CompName:Germacrene D

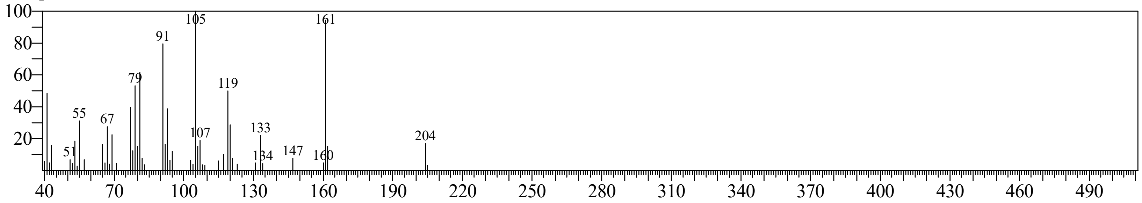

Hit#:5 Entry:41955 Library:NIST17-1.lib
SI:91 Formula:C14H22 CAS:61227-89-2 MolWeight:190 RetIndex:0
CompName:5,6-Decadien-3-yne, 5,7-diethyl- $$ 5,7-Diethyl-5,6-decadien-3-yne # $$

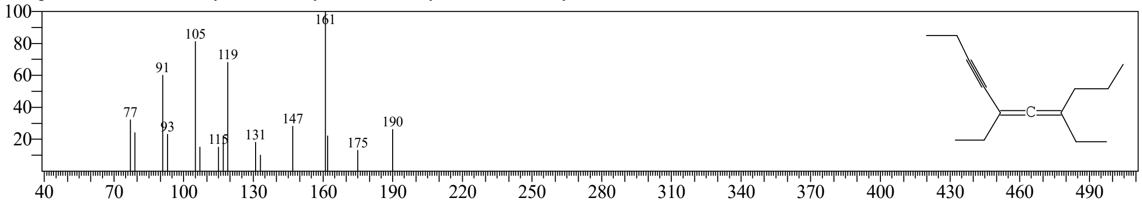

Qualitative Analysis Report

<< Target >>
Line#:26 R.Time:33.496(Scan#:7080) Retention Index:1489 MassPeaks:265
RawMode:Averaged 33.492-33.500(7079-7081) BasePeak:93.10(7345)
BG Mode:Calc. from Peak Group 1 - Event 1 Scan

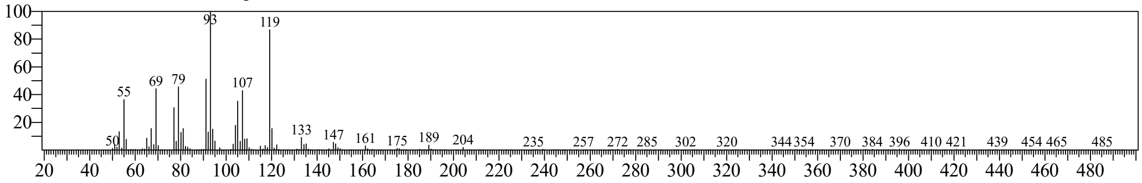

Hit#:1 Entry:992 Library:FFNSC 3.lib
SI:90 Formula:C15 H24 CAS:38738-45-3 MolWeight:204 RetIndex:1496
CompName:Zingiberene <alpha->

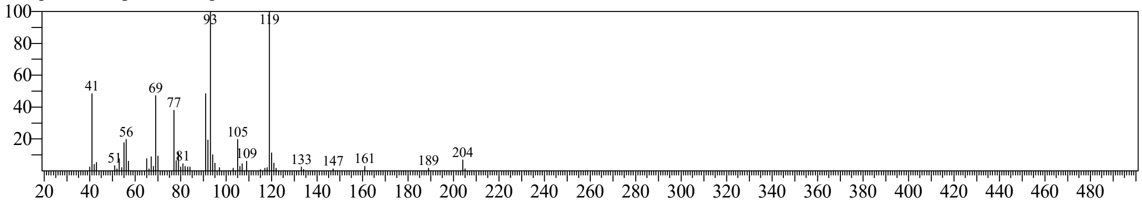

Hit#:2 Entry:1110 Library:FFNSC 3.lib
SI:87 Formula:C15 H24 CAS:67650-90-2 MolWeight:204 RetIndex:1497
CompName:Bicyclogermacrene

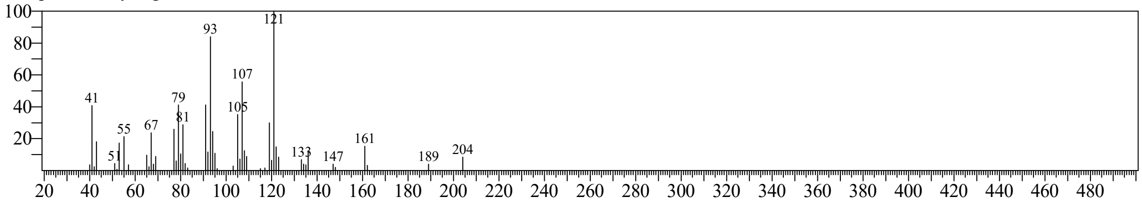

Hit#:3 Entry:71888 Library:NIST17-1.lib
SI:86 Formula:C12H18O2S CAS:0-00-0 MolWeight:226 RetIndex:0
CompName:3,4-Dimethyl-2,5-diprop-2-enyl-2,5-dihydrothiophene 1,1-dioxide

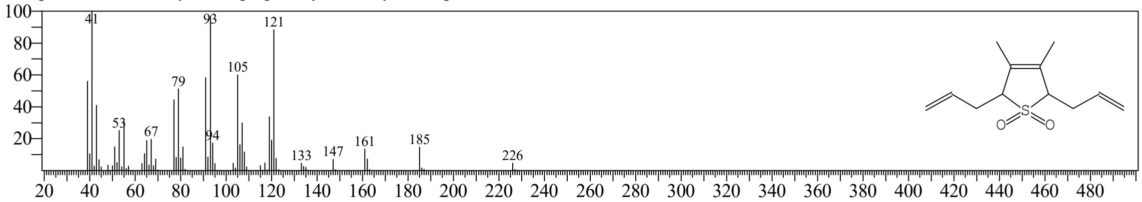

Hit#:4 Entry:2564 Library:FFNSC 3.lib
SI:86 Formula:C15 H24 CAS:90025-12-0 MolWeight:204 RetIndex:1480
CompName:Neocallitropsene <alpha->

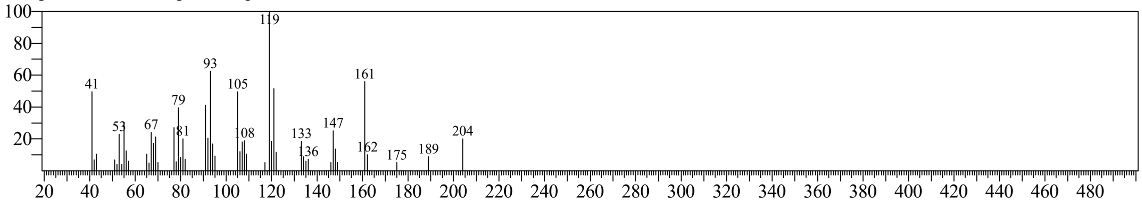

Hit#:5 Entry:52845 Library:NIST17-1.lib
SI:86 Formula:C15H24 CAS:28976-67-2 MolWeight:204 RetIndex:1480
CompName:(R)-1-Methyl-4-(6-methylhept-5-en-2-yl)cyclohexa-1,4-diene $$ .beta.-Curcumene $$ 1,4-Cyclohexadiene, 1-(1,5-dimethyl-4-hexenyl)-4-methy

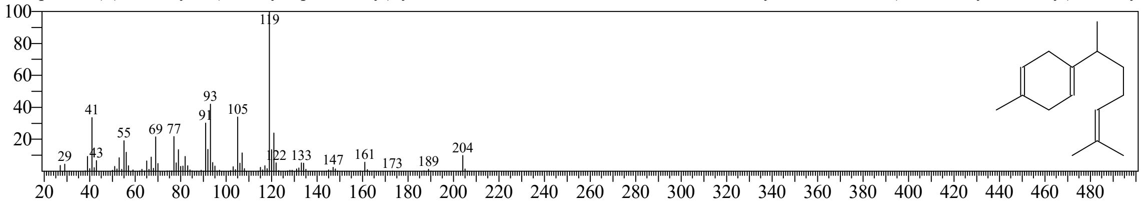

Qualitative Analysis Report

<< Target >>
Line#:27 R.Time:33.600(Scan#:7105) Retention Index:1491 MassPeaks:293
RawMode:Averaged 33.596-33.604(7104-7106) BasePeak:161.15(6049)
BG Mode:Calc. from Peak Group 1 - Event 1 Scan

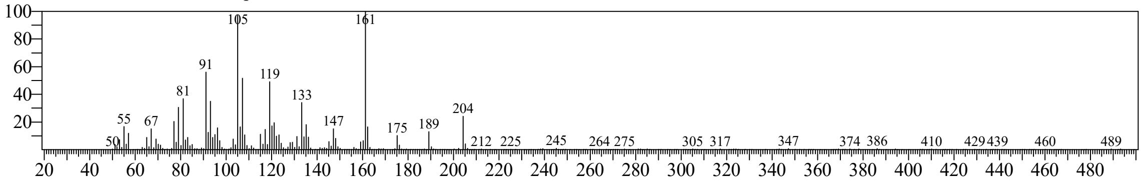

Hit#:1 Entry:41955 Library:NIST17-1.lib
SI:91 Formula:C14H22 CAS:61227-89-2 MolWeight:190 RetIndex:0
CompName:5,6-Decadien-3-yne, 5,7-diethyl- $$ 5,7-Diethyl-5,6-decadien-3-yne # $$

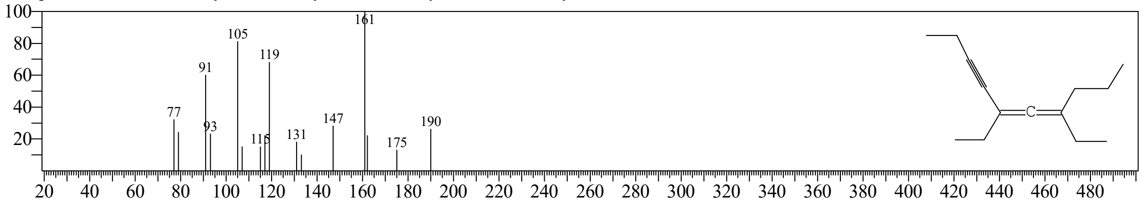

Hit#:2 Entry:1038 Library:FFNSC 3.lib
SI:90 Formula:C15 H24 CAS:74409-93-1 MolWeight:204 RetIndex:1491
CompName:Viridiflorene

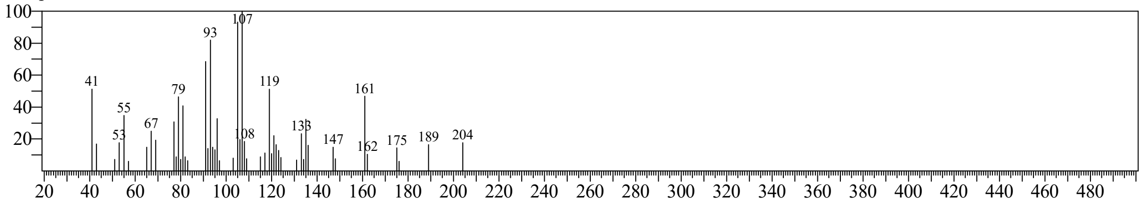

Hit#:3 Entry:52890 Library:NIST17-1.lib
SI:90 Formula:C15H24 CAS:489-83-8 MolWeight:204 RetIndex:1497
CompName:Guaia-3,9-diene $$ 5-Isopropyl-3,8-dimethyl-1,2,4,5,6,7-hexahydroazulene # $$

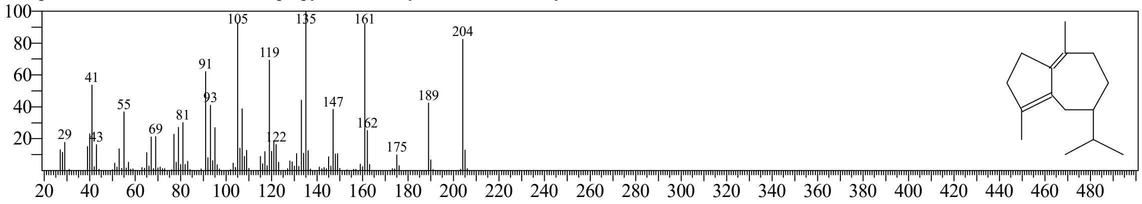

Hit#:4 Entry:2561 Library:FFNSC 3.lib
SI:90 Formula:C15 H24 CAS:88-84-6 MolWeight:204 RetIndex:1498
CompName:Guaiene <cis-beta->

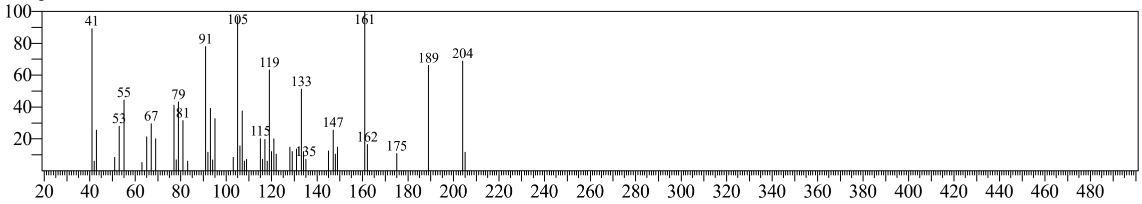

Hit#:5 Entry:1069 Library:FFNSC 3.lib
SI:89 Formula:C15 H24 CAS:483-75-0 MolWeight:204 RetIndex:1482
CompName:Amorphene <alpha->

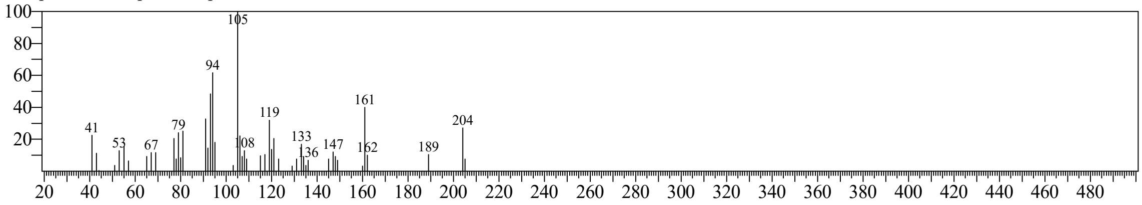

Qualitative Analysis Report

<< Target >>
Line#:28 R.Time:33.767(Scan#:7145) Retention Index:1495 MassPeaks:251
RawMode:Averaged 33.763-33.771(7144-7146) BasePeak:121.10(43824)
BG Mode:Calc. from Peak Group 1 - Event 1 Scan

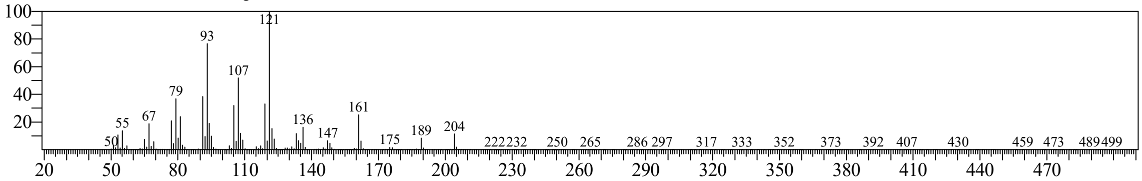

Hit#:1 Entry:52873 Library:NIST17-1.lib
SI:97 Formula:C15H24 CAS:24703-35-3 MolWeight:204 RetIndex:1499
CompName:(1S,2E,6E,10R)-3,7,11,11-Tetramethylbicyclo[8.1.0]undeca-2,6-diene $$ Bicyclo[8.1.0]undeca-2,6-diene, 3,7,11,11-tetramethyl-, (1S,2E,6E,1C

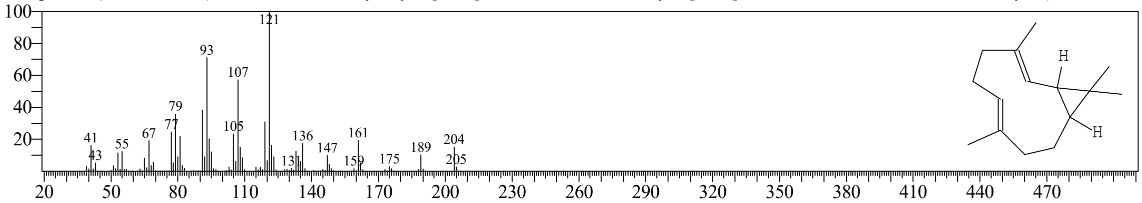

Hit#:2 Entry:1110 Library:FFNSC 3.lib
SI:96 Formula:C15 H24 CAS:67650-90-2 MolWeight:204 RetIndex:1497
CompName:Bicyclogermacrene

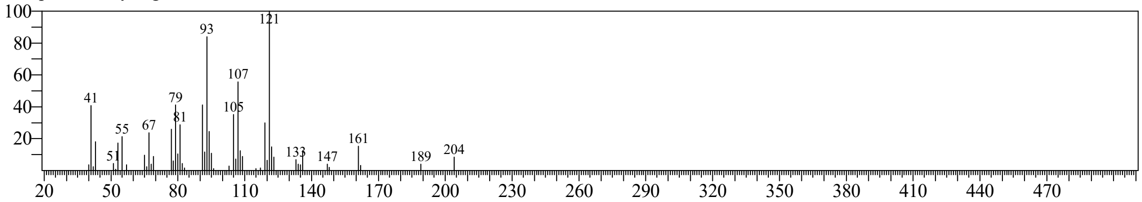

Hit#:3 Entry:71888 Library:NIST17-1.lib
SI:88 Formula:C12H18O2S CAS:0-00-0 MolWeight:226 RetIndex:0
CompName:3,4-Dimethyl-2,5-diprop-2-enyl-2,5-dihydrothiophene 1,1-dioxide

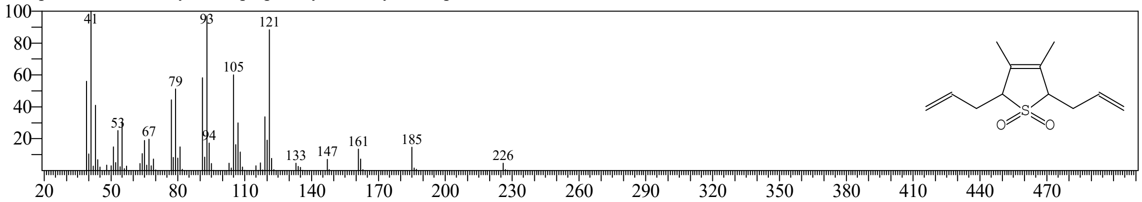

Hit#:4 Entry:24215 Library:NIST17-1.lib
SI:86 Formula:C10H16N2 CAS:0-00-0 MolWeight:164 RetIndex:0
CompName:2,3-Diazabicyclo[2.2.1]hept-2-ene, 5-ethenyl-4,7,7-trimethyl-, (1.alpha.,4.alpha.,5.beta.)- $$ 1,7,7-Trimethyl-6-vinyl-2,3-diazabicyclo[2.2.1]hep

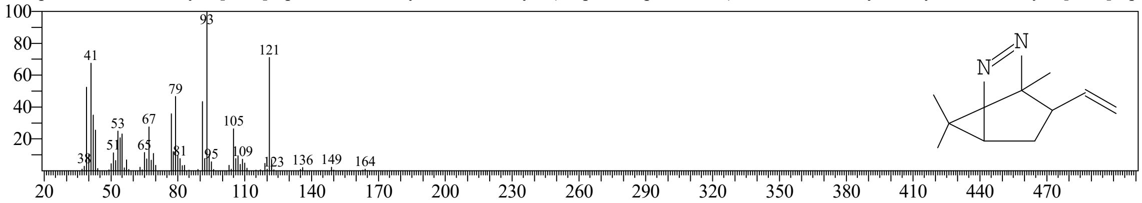

Hit#:5 Entry:41932 Library:NIST17-1.lib
SI:86 Formula:C14H22 CAS:62338-54-9 MolWeight:190 RetIndex:1485
CompName:1,5-Cycloundecadiene, 8,8-dimethyl-9-methylene- $$ 8,8-Dimethyl-9-methylene-1,5-cycloundecadiene # $$

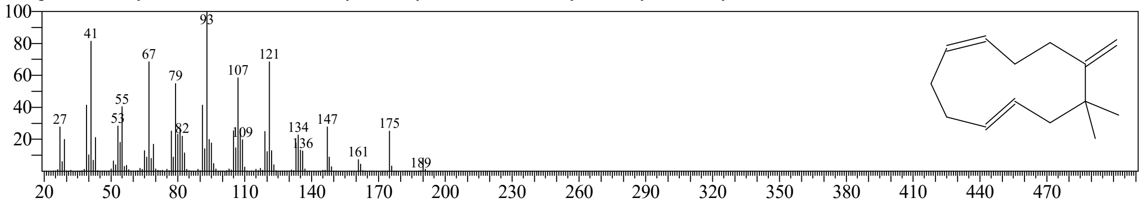

Qualitative Analysis Report

<< Target >>
Line#:29 R.Time:33.879(Scan#:7172) Retention Index:1498 MassPeaks:229
RawMode:Averaged 33.875-33.883(7171-7173) BasePeak:105.10(11977)
BG Mode:Calc. from Peak Group 1 - Event 1 Scan

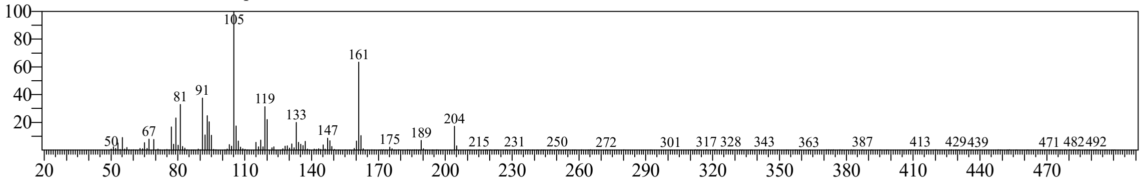

Hit#:1 Entry:1052 Library:FFNSC 3.lib
SI:93 Formula:C15 H24 CAS:10208-80-7 MolWeight:204 RetIndex:1497
CompName:Muurolene <alpha->

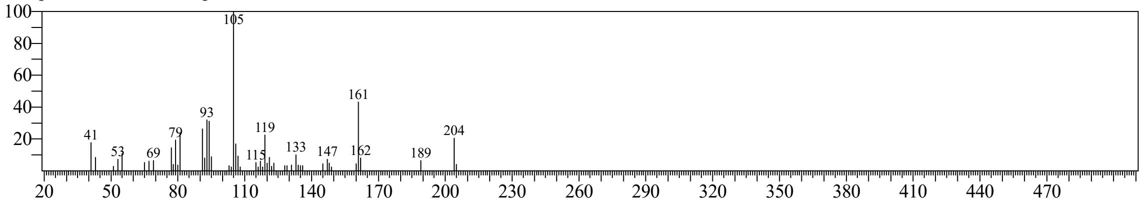

Hit#:2 Entry:163893 Library:NIST17-1.lib
SI:91 Formula:C18H16N4O2 CAS:19226-31-4 MolWeight:320 RetIndex:0
CompName:Benzoic acid, anhydride with N-(4,5-dimethyl-1H-1,2,3-triazol-1-yl)benzenecarboximidic acid $$ Benzoic acid, anhydride with N-(4,5-dimethyl

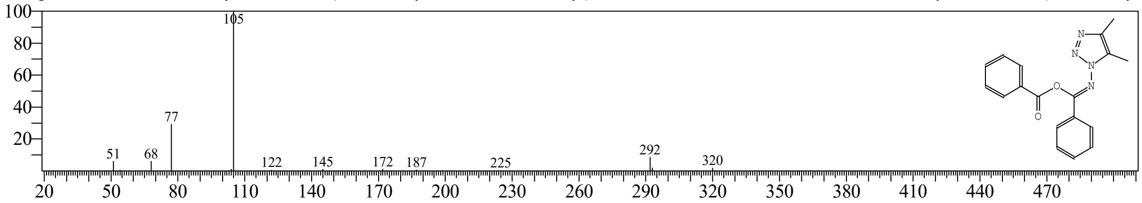

Hit#:3 Entry:1673 Library:FFNSC 3.lib
SI:91 Formula:C15 H24 CAS:6980-46-7 MolWeight:204 RetIndex:1490
CompName:Amorphene <gamma->

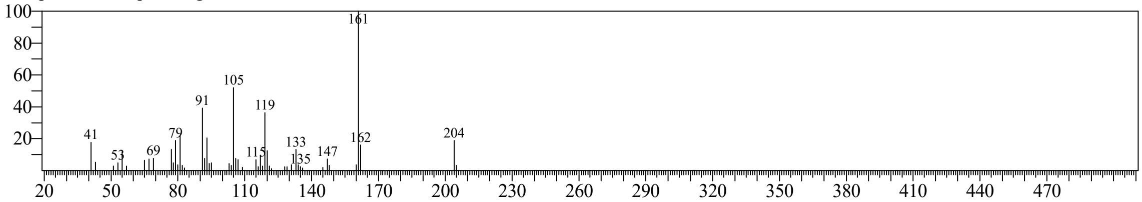

Hit#:4 Entry:2398 Library:FFNSC 3.lib
SI:89 Formula:C15 H24 CAS:54324-03-7 MolWeight:204 RetIndex:1496
CompName:Muurola-4(14),5-diene<trans->

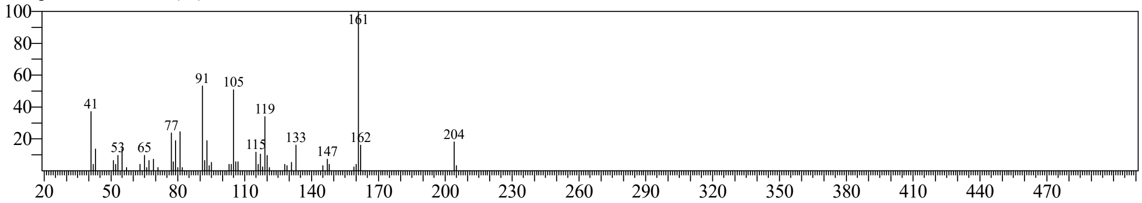

Hit#:5 Entry:42986 Library:NIST17-1.lib
SI:88 Formula:C11H12O3 CAS:0-00-0 MolWeight:192 RetIndex:1495
CompName:Phenylglyoxylic acid, propyl ester

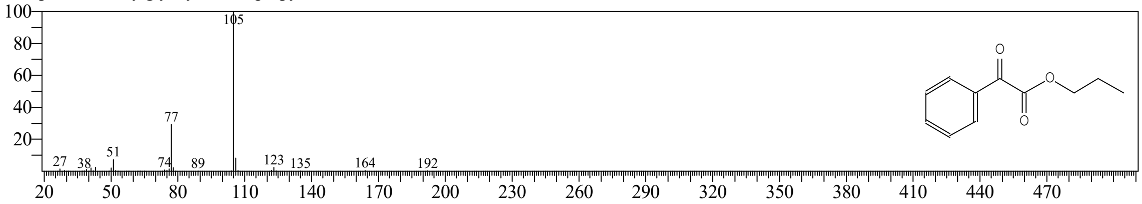

Qualitative Analysis Report

<< Target >>
Line#:30 R.Time:34.067(Scan#:7217) Retention Index:1502 MassPeaks:266
RawMode:Averaged 34.063-34.071(7216-7218) BasePeak:93.10(74210)
BG Mode:Calc. from Peak Group 1 - Event 1 Scan

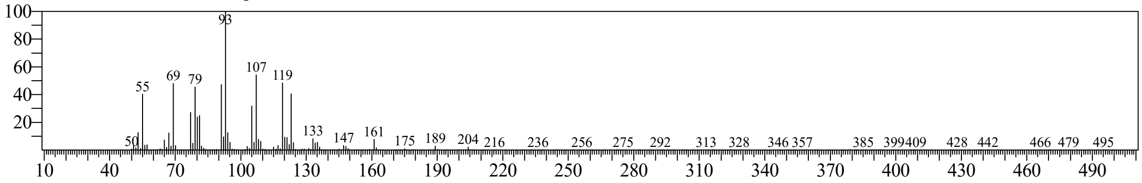

Hit#:1 Entry:1073 Library:FFNSC 3.lib
SI:96 Formula:C15 H24 CAS:502-61-4 MolWeight:204 RetIndex:1504
CompName:Farnesene <(E,E)-, alpha->

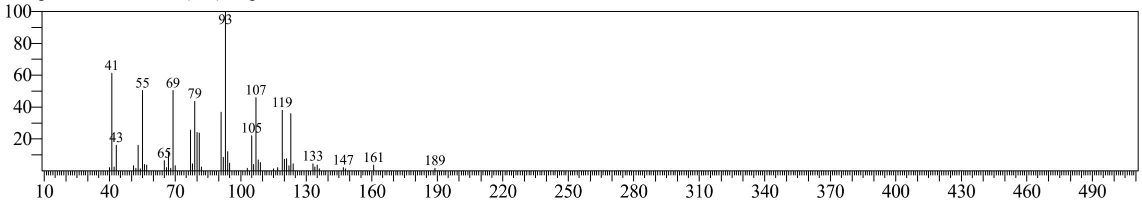

Hit#:2 Entry:1687 Library:FFNSC 3.lib
SI:92 Formula:C15 H24 CAS:13062-00-5 MolWeight:204 RetIndex:1511
CompName:Bisabolene <(Z)-, gamma->

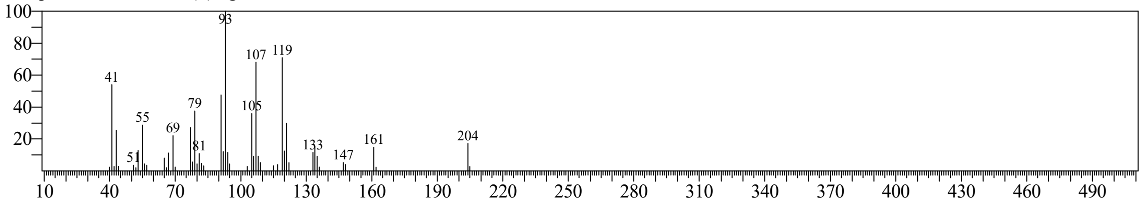

Hit#:3 Entry:1110 Library:FFNSC 3.lib
SI:88 Formula:C15 H24 CAS:67650-90-2 MolWeight:204 RetIndex:1497
CompName:Bicyclogermacrene

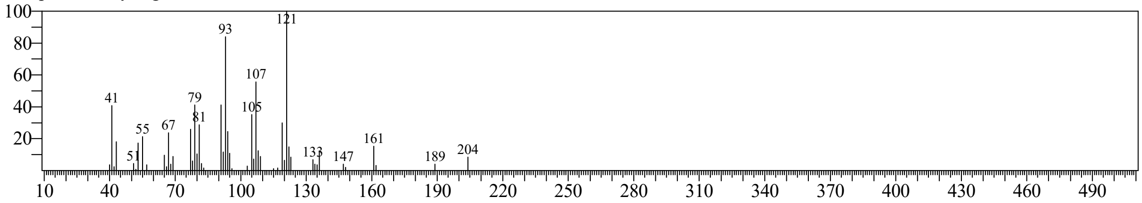

Hit#:4 Entry:5613 Library:NIST17-1.lib
SI:87 Formula:BrN3 CAS:13973-87-0 MolWeight:121 RetIndex:0
CompName:Bromine azide $$ Bromine azide (Br(N3)) $$ Bromine nitride $$ Nitrogen bromide (N3Br) $$

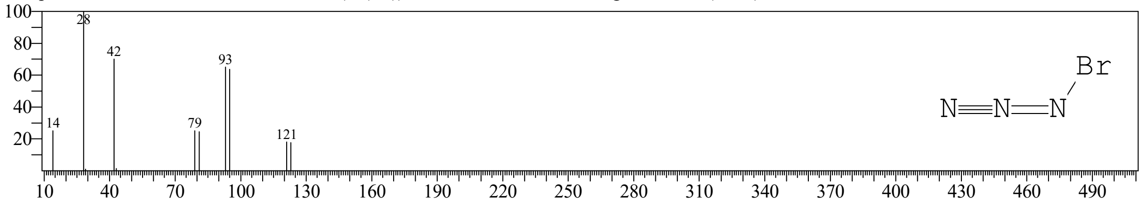

Hit#:5 Entry:992 Library:FFNSC 3.lib
SI:87 Formula:C15 H24 CAS:38738-45-3 MolWeight:204 RetIndex:1496
CompName:Zingiberene <alpha->

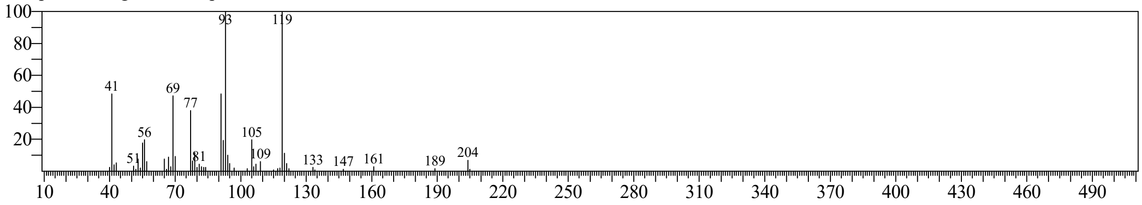

Qualitative Analysis Report

<< Target >>
Line#:31 R.Time:34.233(Scan#:7257) Retention Index:1507 MassPeaks:288
RawMode:Averaged 34.229-34.237(7256-7258) BasePeak:93.10(4956)
BG Mode:Calc. from Peak Group 1 - Event 1 Scan

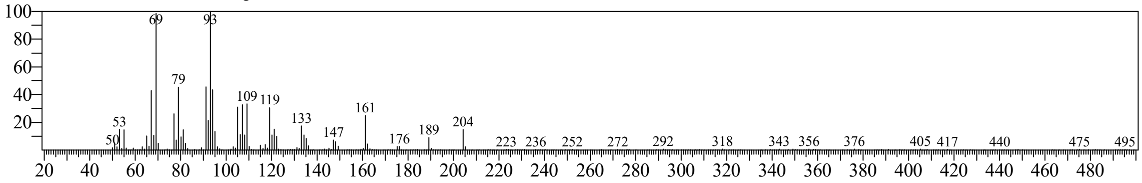

Hit#:1 Entry:961 Library:FFNSC 3.lib
SI:95 Formula:C15 H24 CAS:4891-79-6 MolWeight:204 RetIndex:1508
CompName:Bisabolene <beta->

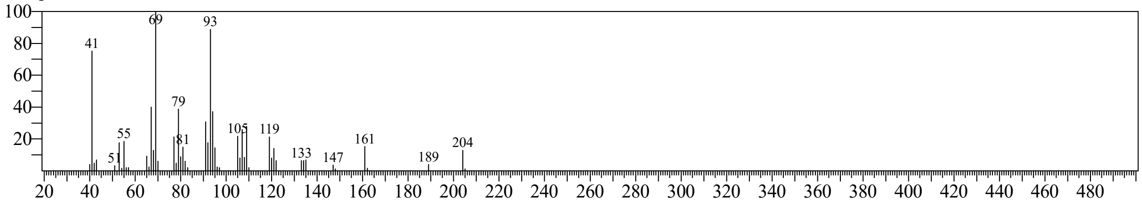

Hit#:2 Entry:52743 Library:NIST17-1.lib
SI:90 Formula:C15H24 CAS:495-61-4 MolWeight:204 RetIndex:1500
CompName:.beta.-Bisabolene $$ Cyclohexene, 1-methyl-4-(5-methyl-1-methylene-4-hexenyl)-, (S)- $$ 1,5-Heptadiene, 6-methyl-2-(4-methyl-3-cyclohexen-

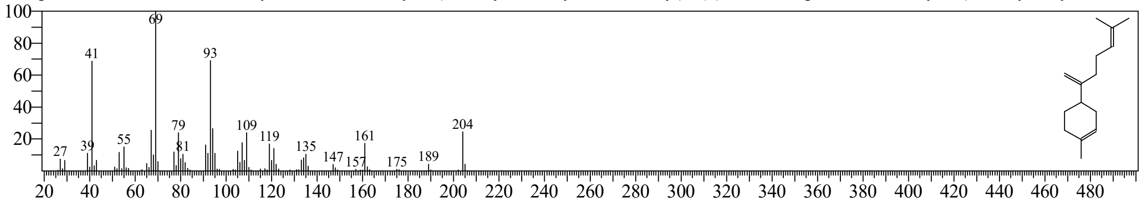

Hit#:3 Entry:2939 Library:NIST17-1.lib
SI:89 Formula:C6H8Si CAS:63878-65-9 MolWeight:108 RetIndex:0
CompName:Silabenzene,1-methyl- $$ 1-Methyl-1-silabenzene $$ 1-Methylsiline # $$

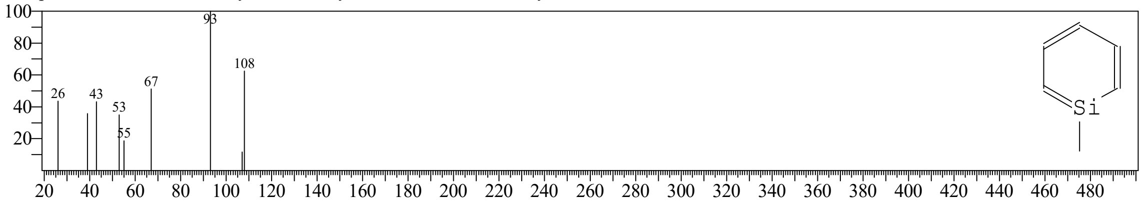

Hit#:4 Entry:1687 Library:FFNSC 3.lib
SI:88 Formula:C15 H24 CAS:13062-00-5 MolWeight:204 RetIndex:1511
CompName:Bisabolene <(Z)-, gamma->

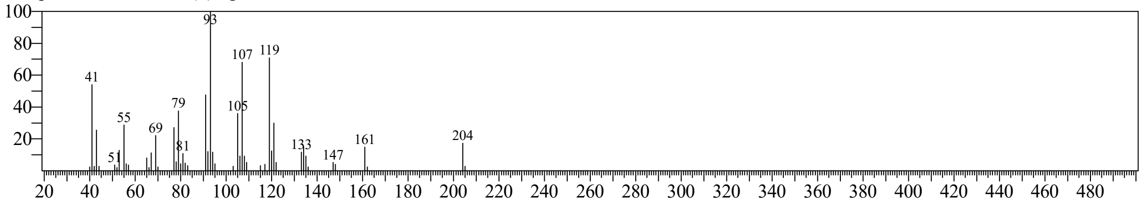

Hit#:5 Entry:1686 Library:FFNSC 3.lib
SI:87 Formula:C15 H24 CAS:29837-07-8 MolWeight:204 RetIndex:1503
CompName:Bisabolene <(Z)-, alpha->

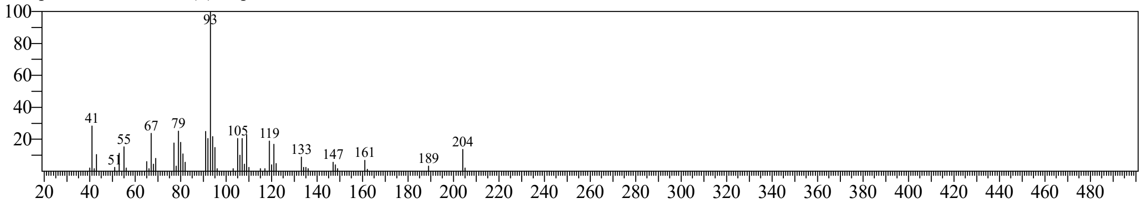

Qualitative Analysis Report

<< Target >>
Line#:32 R.Time:34.454(Scan#:7310) Retention Index:1512 MassPeaks:259
RawMode:Averaged 34.450-34.458(7309-7311) BasePeak:161.15(6605)
BG Mode:Calc. from Peak Group 1 - Event 1 Scan

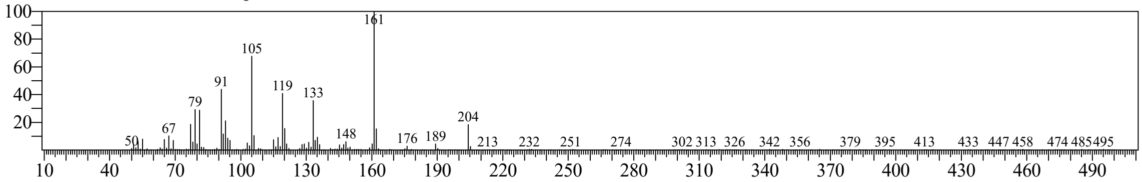

Hit#:1 Entry:1093 Library:FFNSC 3.lib
SI:95 Formula:C15 H24 CAS:38357-83-4 MolWeight:204 RetIndex:1512
CompName:Cadinene <gamma->

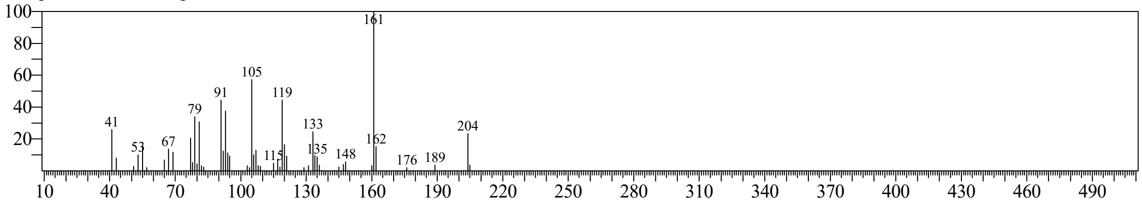

Hit#:2 Entry:52917 Library:NIST17-1.lib
SI:90 Formula:C15H24 CAS:23986-74-5 MolWeight:204 RetIndex:1515
CompName:Germacrene D $$ (S,1Z,6Z)-8-Isopropyl-1-methyl-5-methylenecyclodeca-1,6-diene $$ D-Germacrene $$ 1(10),4(14),5-Germacratriene $$ (-)-[illegible]

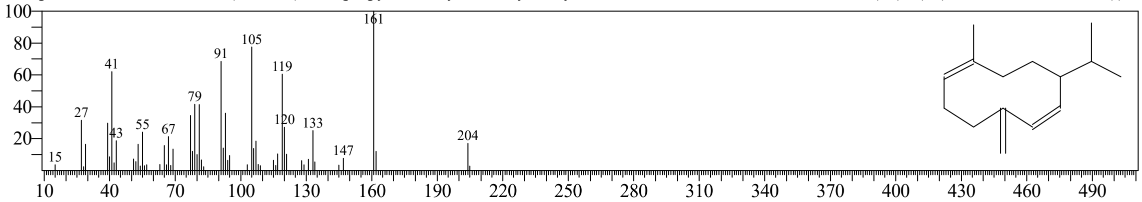

Hit#:3 Entry:2894 Library:FFNSC 3.lib
SI:89 Formula:C15 H24 CAS:41702-63-0 MolWeight:204 RetIndex:1506
CompName:Epizonarene

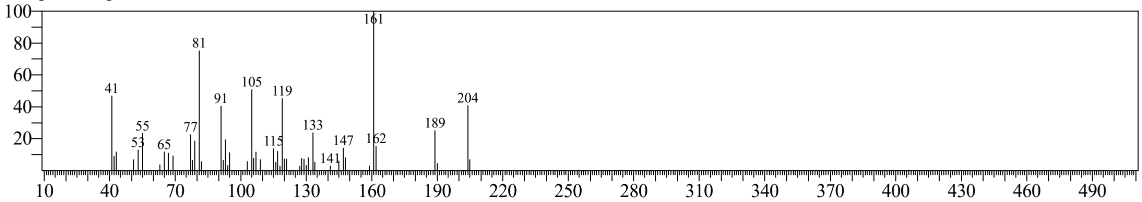

Hit#:4 Entry:838 Library:FFNSC 3.lib
SI:89 Formula:C15 H24 CAS:16729-01-4 MolWeight:204 RetIndex:1518
CompName:Cadinene <delta->

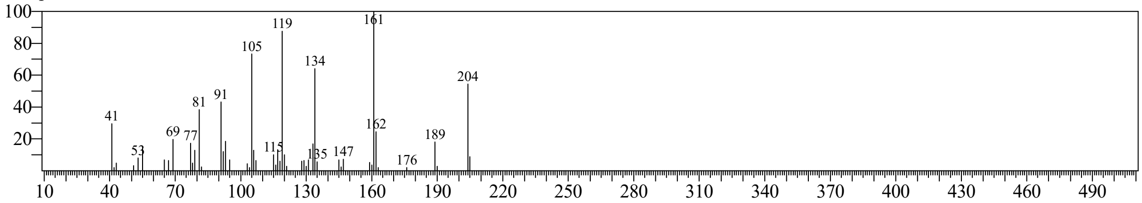

Hit#:5 Entry:1773 Library:FFNSC 3.lib
SI:89 Formula:C15 H24 CAS:317819-82-2 MolWeight:204 RetIndex:1506
CompName:Amorphene <delta->

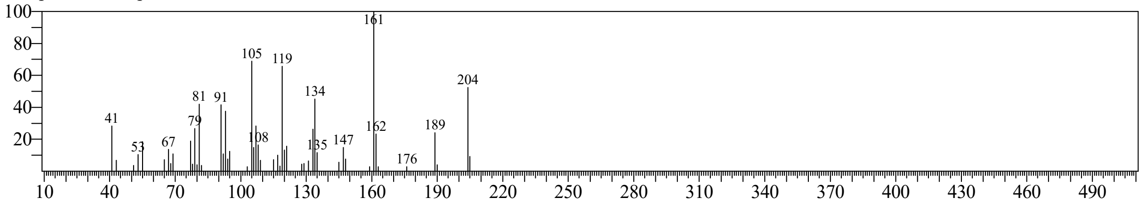

Qualitative Analysis Report

<< Target >>
Line#:33 R.Time:34.671(Scan#:7362) Retention Index:1518 MassPeaks:259
RawMode:Averaged 34.667-34.675(7361-7363) BasePeak:161.15(34145)
BG Mode:Calc. from Peak Group 1 - Event 1 Scan

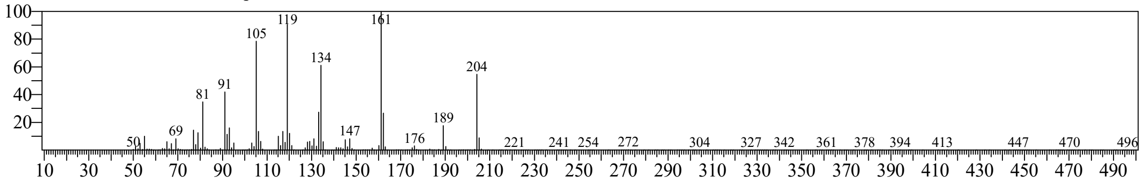

Hit#:1 Entry:838 Library:FFNSC 3.lib
SI:97 Formula:C15 H24 CAS:16729-01-4 MolWeight:204 RetIndex:1518
CompName:Cadinene <delta->

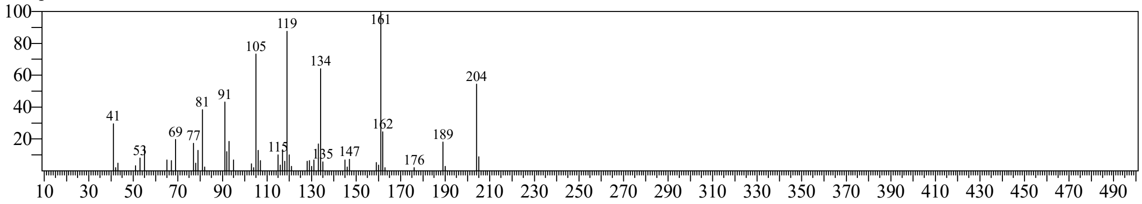

Hit#:2 Entry:41955 Library:NIST17-1.lib
SI:91 Formula:C14H22 CAS:61227-89-2 MolWeight:190 RetIndex:0
CompName:5,6-Decadien-3-yne, 5,7-diethyl- $$ 5,7-Diethyl-5,6-decadien-3-yne # $$

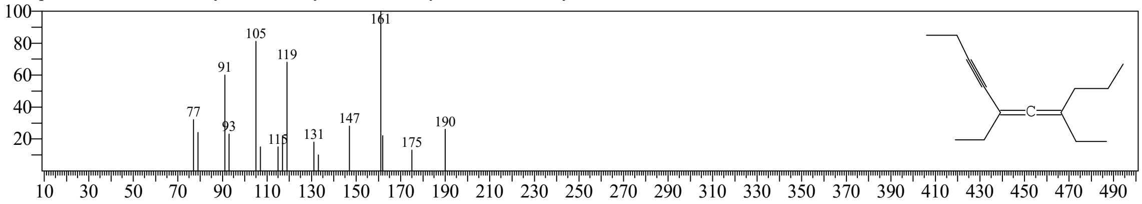

Hit#:3 Entry:1093 Library:FFNSC 3.lib
SI:87 Formula:C15 H24 CAS:38357-83-4 MolWeight:204 RetIndex:1512
CompName:Cadinene <gamma->

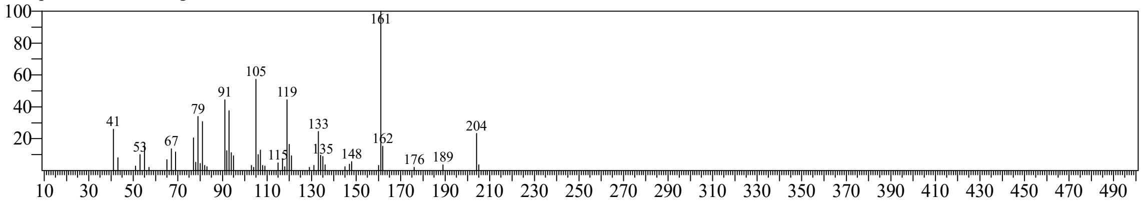

Hit#:4 Entry:52917 Library:NIST17-1.lib
SI:87 Formula:C15H24 CAS:23986-74-5 MolWeight:204 RetIndex:1515
CompName:Germacrene D $$ (S,1Z,6Z)-8-Isopropyl-1-methyl-5-methylenecyclodeca-1,6-diene $$ D-Germacrene $$ 1(10),4(14),5-Germacratriene $$ (-)-C

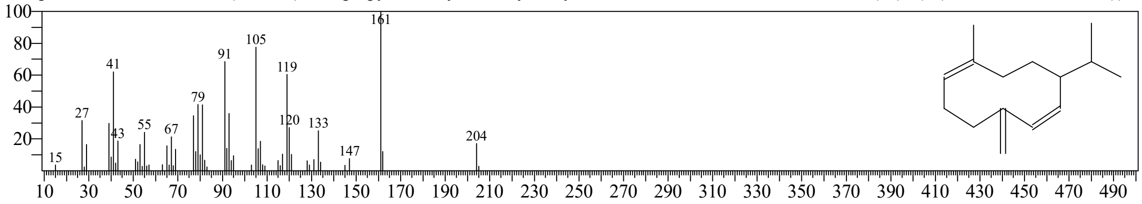

Hit#:5 Entry:52865 Library:NIST17-1.lib
SI:86 Formula:C15H24 CAS:29621-78-1 MolWeight:204 RetIndex:1512
CompName:.alpha.-Cuprenene $$ (S)-1-Methyl-4-(1,2,2-trimethylcyclopentyl)cyclohexa-1,3-diene $$ 1,3-Cyclohexadiene, 1-methyl-4-[(1S)-1,2,2-trimethyl

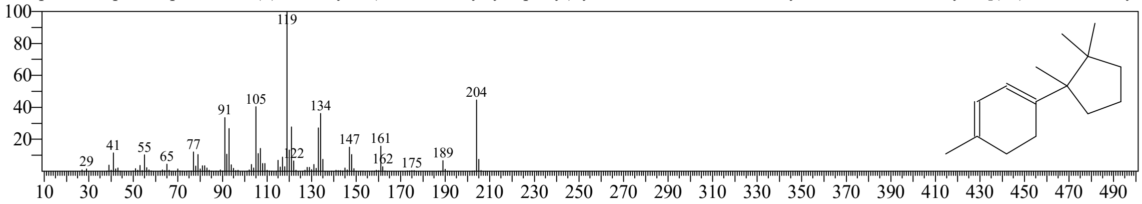

Qualitative Analysis Report

<< Target >>
Line#:34 R.Time:34.775(Scan#:7387) Retention Index:1520 MassPeaks:295
RawMode:Averaged 34.771-34.779(7386-7388) BasePeak:151.15(2981)
BG Mode:Calc. from Peak Group 1 - Event 1 Scan

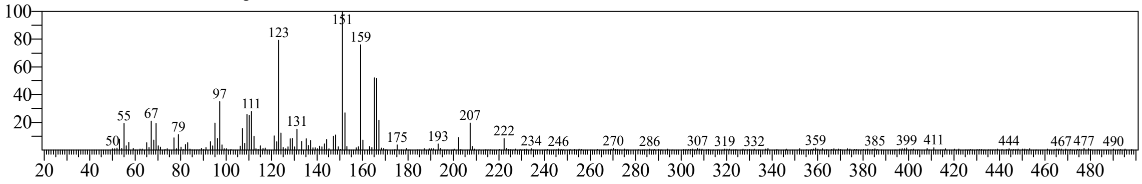

Hit#:1 Entry:67771 Library:NIST17-1.lib
SI:89 Formula:C10H10N2O2S CAS:19703-85-6 MolWeight:222 RetIndex:0
CompName:1,3,4-Thiadiazolium, 5-hydroxy-2-(4-methoxyphenyl)-3-methyl-, hydroxide, inner salt $$ 1,3,4-Thiadiazolium, 5-hydroxy-2-(p-methoxyphenyl)-

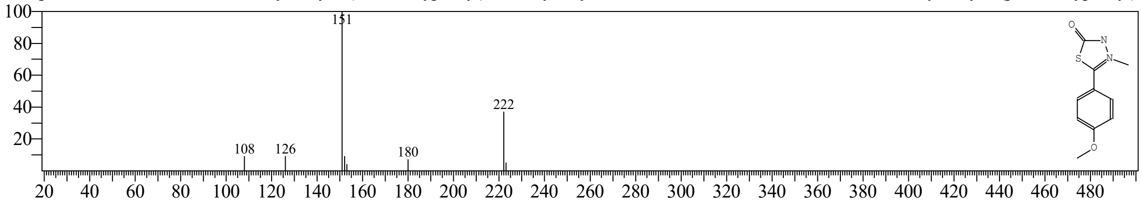

Hit#:2 Entry:93530 Library:NIST17-1.lib
SI:87 Formula:C12H14N2O2S CAS:53492-47-0 MolWeight:250 RetIndex:0
CompName:4-Methyl-3-[2-(4-methylphenylthio)ethyl]sydnone

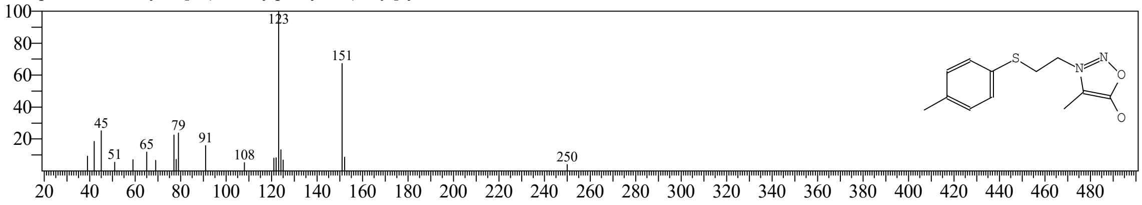

Hit#:3 Entry:38369 Library:NIST17-1.lib
SI:85 Formula:C4H8ClO4P CAS:17027-41-7 MolWeight:186 RetIndex:0
CompName:Phosphoric acid, 2-chloroethenyl dimethyl ester $$ Phosphoric acid, 2-chlorovinyl dimethyl ester $$ O,O-Dimethyl 2-monochlorovinyl phospha

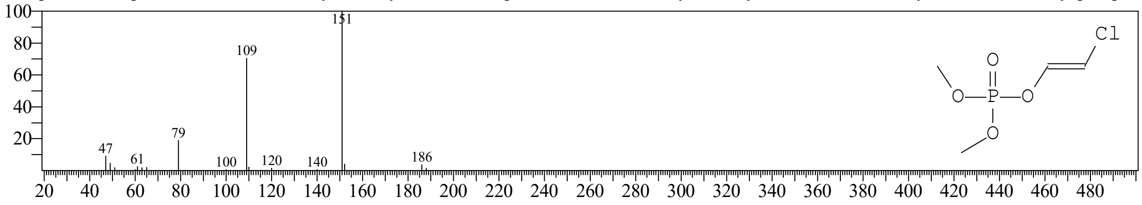

Hit#:4 Entry:80458 Library:NIST17-1.lib
SI:84 Formula:C11H12N2O2S CAS:96991-48-9 MolWeight:236 RetIndex:0
CompName:3-[2-(4-Methylphenylthio)ethyl]-4-H-sydnone

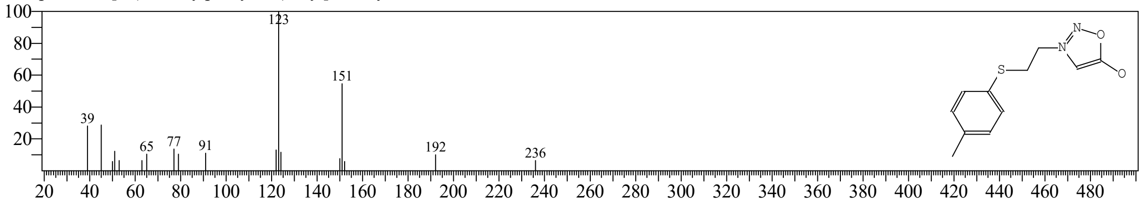

Hit#:5 Entry:56192 Library:NIST17-1.lib
SI:84 Formula:C12H20OSi CAS:0-00-0 MolWeight:208 RetIndex:0
CompName:Silane, (3,3-dimethylbut-2-yloxy)-(phenyl)- $$ Phenyl(1,2,2-trimethylpropoxy)silane # $$

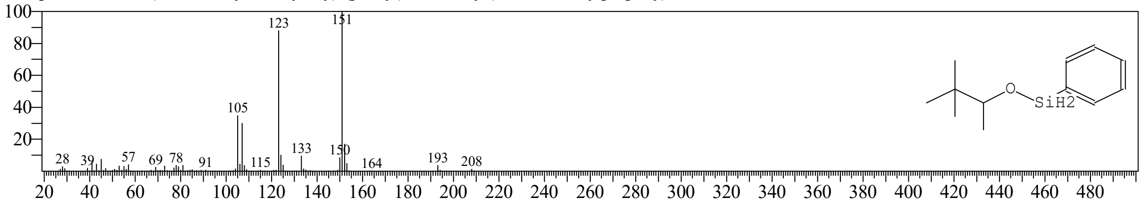

Qualitative Analysis Report

<< Target >>
Line#:35 R.Time:34.996(Scan#:7440) Retention Index:1526 MassPeaks:271
RawMode:Averaged 34.992-35.000(7439-7441) BasePeak:93.10(1254)
BG Mode:Calc. from Peak Group 1 - Event 1 Scan

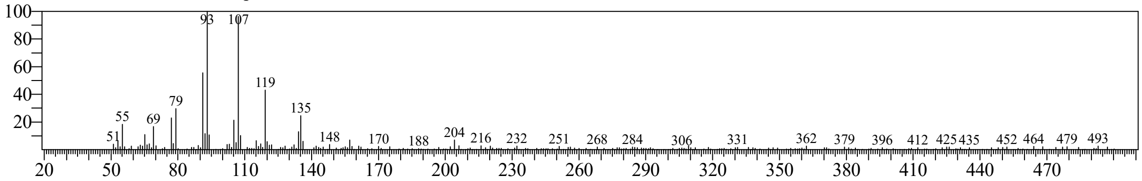

Hit#:1 Entry:52778 Library:NIST17-1.lib
SI:94 Formula:C15H24 CAS:53585-13-0 MolWeight:204 RetIndex:1534
CompName:(E)-1-Methyl-4-(6-methylhept-5-en-2-ylidene)cyclohex-1-ene $$ Cyclohexene, 4-(1,5-dimethyl-4-hexen-1-ylidene)-1-methyl-, (E)- $$ Cyclohex

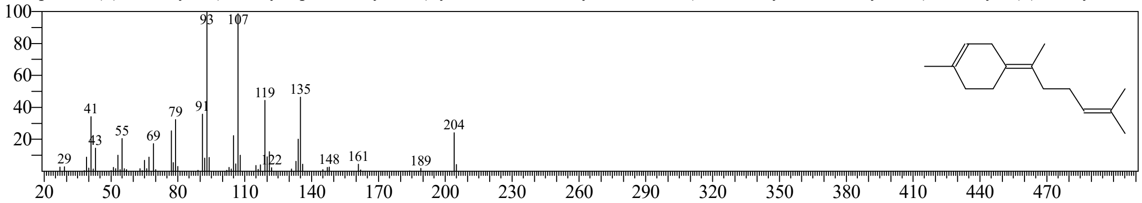

Hit#:2 Entry:52781 Library:NIST17-1.lib
SI:88 Formula:C15H24 CAS:13062-00-5 MolWeight:204 RetIndex:1534
CompName:(Z)-1-Methyl-4-(6-methylhept-5-en-2-ylidene)cyclohex-1-ene $$ Cyclohexene, 4-(1,5-dimethyl-4-hexen-1-ylidene)-1-methyl-, (4Z)- $$ 2-Hepte

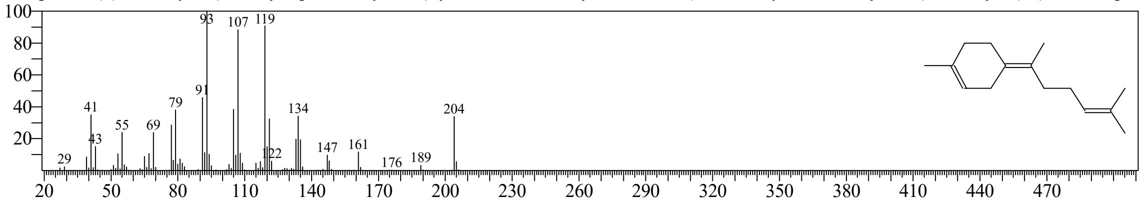

Hit#:3 Entry:10127 Library:NIST17-1.lib
SI:85 Formula:C8H12N2 CAS:109746-10-3 MolWeight:136 RetIndex:0
CompName:1,4-Methano-1H-Cyclopropa[d]pyridazine, 4,4a,5,5a-tetrahydro-6,6-dimethyl-, (1.alpha.,4.alpha.,4a.alpha.,5a.alpha.)-

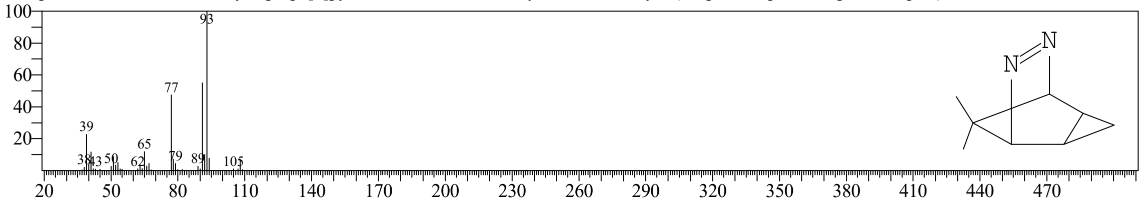

Hit#:4 Entry:148375 Library:NIST17-1.lib
SI:85 Formula:C14H20N5OP CAS:62729-72-0 MolWeight:305 RetIndex:0
CompName:Phosphinic amide, N,N-dimethyl-, bis(2-phenylhydrazino)- $$ N,N-Dimethyl-N''',N''''-diphenylphosphorodihydrazidic amide # $$

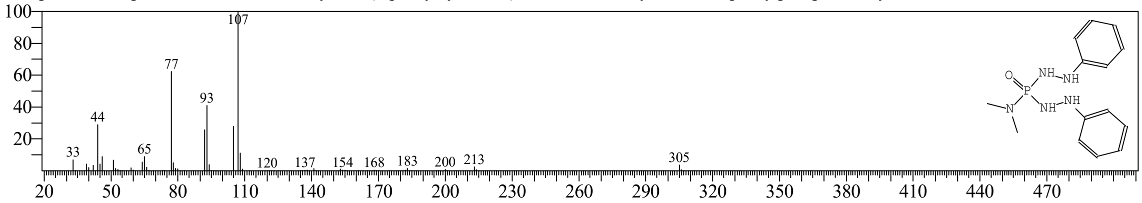

Hit#:5 Entry:31366 Library:NIST17-1.lib
SI:83 Formula:C11H13NO CAS:80188-12-1 MolWeight:175 RetIndex:1517
CompName:3-Butenamide, 2-methyl-N-phenyl- $$ 2-Methyl-N-phenyl-3-butenamide # $$

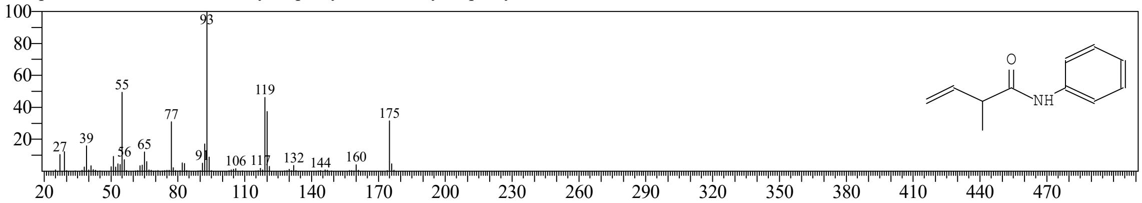

Qualitative Analysis Report

<< Target >>
Line#:36 R.Time:35.225(Scan#:7495) Retention Index:1532 MassPeaks:271
RawMode:Averaged 35.221-35.229(7494-7496) BasePeak:119.10(1065)
BG Mode:Calc. from Peak Group 1 - Event 1 Scan

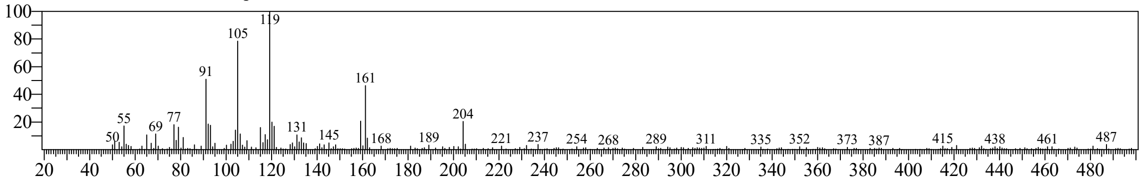

Hit#:1 Entry:1475 Library:FFNSC 3.lib
SI:93 Formula:C15 H24 CAS:38758-02-0 MolWeight:204 RetIndex:1536
CompName:Cadina-1,4-diene <trans->

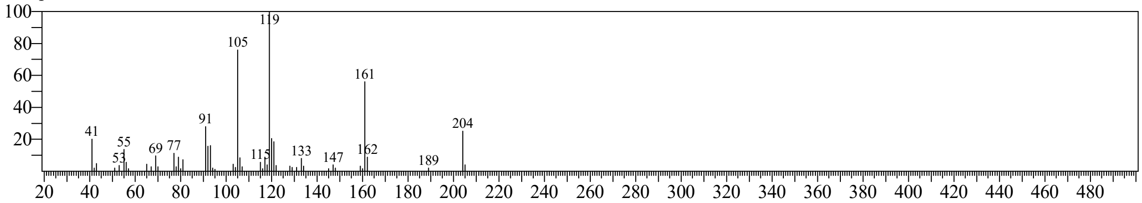

Hit#:2 Entry:41955 Library:NIST17-1.lib
SI:86 Formula:C14H22 CAS:61227-89-2 MolWeight:190 RetIndex:0
CompName:5,6-Decadien-3-yne, 5,7-diethyl- $$ 5,7-Diethyl-5,6-decadien-3-yne # $$

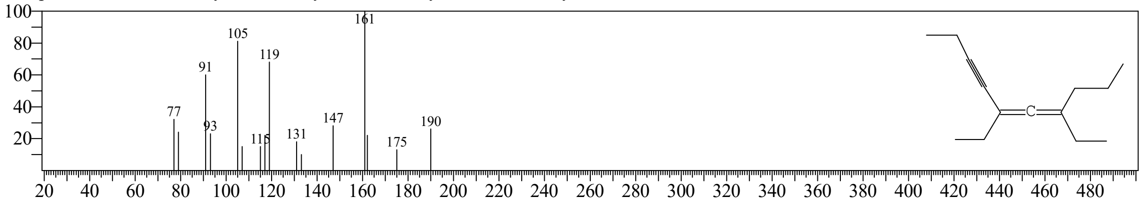

Hit#:3 Entry:52836 Library:NIST17-1.lib
SI:86 Formula:C15H24 CAS:1461-03-6 MolWeight:204 RetIndex:1528
CompName:1H-Benzocycloheptene, 2,4a,5,6,7,8-hexahydro-3,5,5,9-tetramethyl-, (R)- $$ .beta.-Himachalene $$ 1H-Benzocycloheptene, 2,4a.beta.,5,6,7,8-l

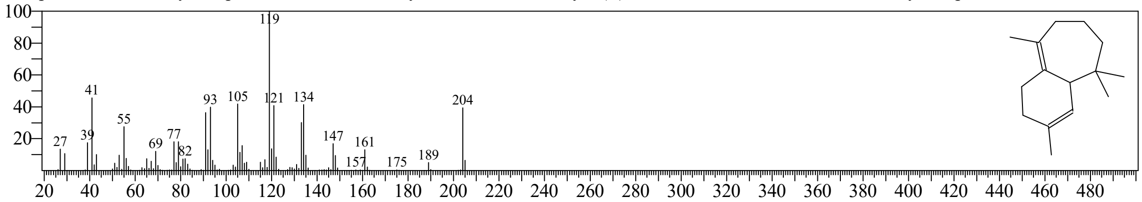

Hit#:4 Entry:174236 Library:NIST17-1.lib
SI:85 Formula:C21H18N2O2 CAS:0-00-0 MolWeight:330 RetIndex:0
CompName:1H-Pyrrolo[2,3-b]pyridin-2(3H)-one, 3,3-di(4-methylphenyl)-, 7-oxide $$ 3,3-Bis(4-methylphenyl)-1,3-dihydro-2H-pyrrolo[2,3-b]pyridin-2-one

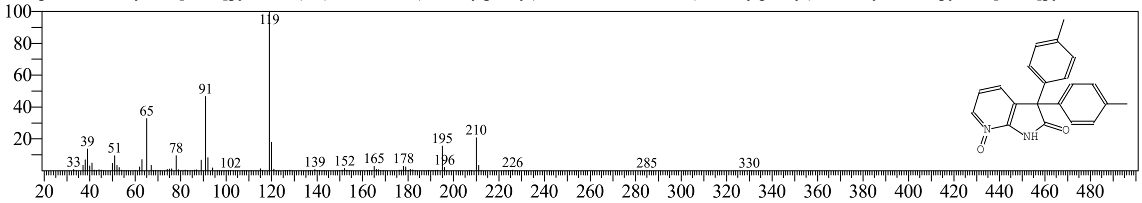

Hit#:5 Entry:1763 Library:FFNSC 3.lib
SI:85 Formula:C15 H24 CAS:82468-90-4 MolWeight:204 RetIndex:1538
CompName:Cadinene <alpha->

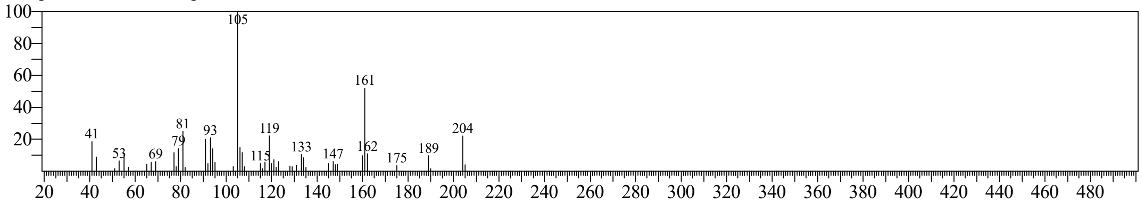

Qualitative Analysis Report

<< Target >>
Line#:37 R.Time:35.392(Scan#:7535) Retention Index:1536 MassPeaks:273
RawMode:Averaged 35.388-35.396(7534-7536) BasePeak:105.10(2296)
BG Mode:Calc. from Peak Group 1 - Event 1 Scan

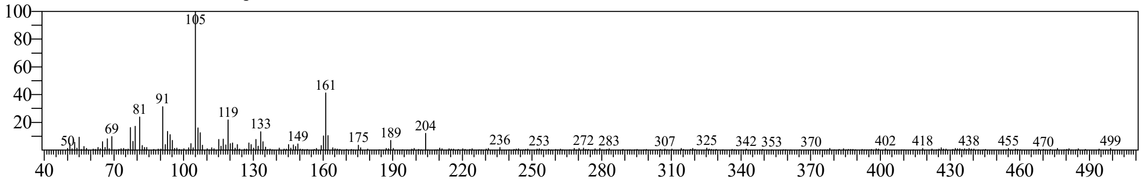

Hit#:1 Entry:1763 Library:FFNSC 3.lib
SI:95 Formula:C15 H24 CAS:82468-90-4 MolWeight:204 RetIndex:1538
CompName:Cadinene <alpha->

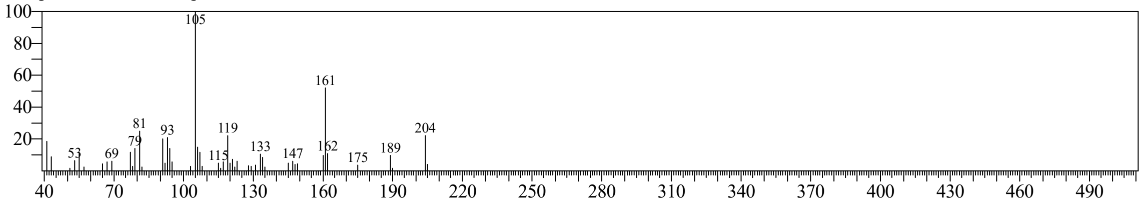

Hit#:2 Entry:163893 Library:NIST17-1.lib
SI:92 Formula:C18H16N4O2 CAS:19226-31-4 MolWeight:320 RetIndex:0
CompName:Benzoic acid, anhydride with N-(4,5-dimethyl-1H-1,2,3-triazol-1-yl)benzenecarboximidic acid $$ Benzoic acid, anhydride with N-(4,5-dimethyl

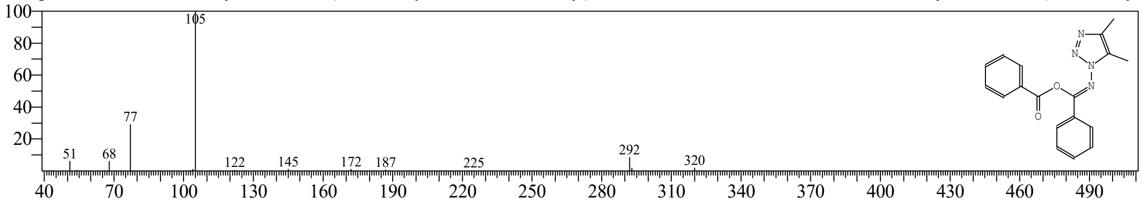

Hit#:3 Entry:208097 Library:NIST17-1.lib
SI:91 Formula:C16H18Se2 CAS:65915-30-2 MolWeight:370 RetIndex:0
CompName:Diselenide, 4,4'-bis(4-methylbenzyl)- $$ 1,2-Bis(4-methylbenzyl)diselane # $$

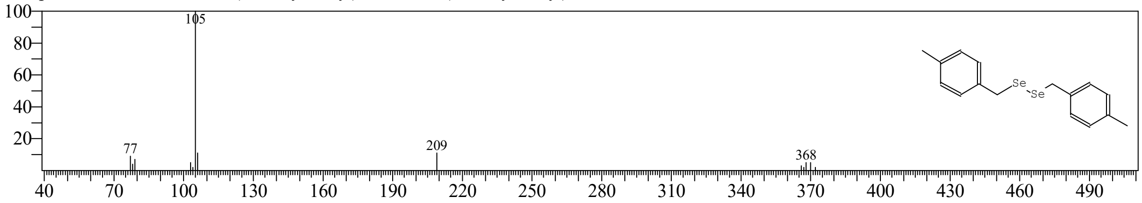

Hit#:4 Entry:97809 Library:NIST17-1.lib
SI:90 Formula:C16H18N2O CAS:64857-67-6 MolWeight:254 RetIndex:0
CompName:1,2-Bis(3,5-dimethylphenyl)-diazene 1-oxide

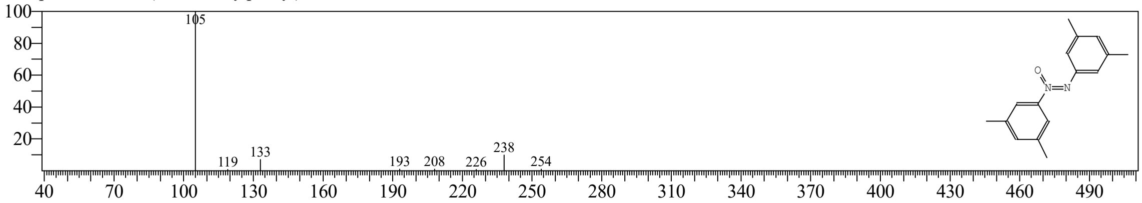

Hit#:5 Entry:163892 Library:NIST17-1.lib
SI:89 Formula:C18H16N4O2 CAS:19226-35-8 MolWeight:320 RetIndex:0
CompName:Benzamide, N-benzoyl-N-(4,5-dimethyl-1H-1,2,3-triazol-1-yl)- $$ Dibenzamide, N-(4,5-dimethyl-1H-1,2,3-triazol-1-yl)- $$ N-Benzoyl-N-(4,5-

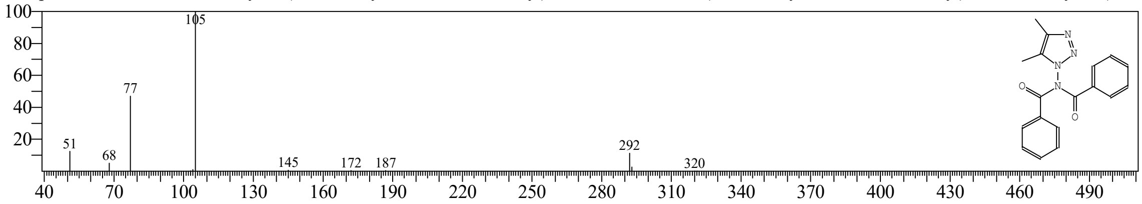

Qualitative Analysis Report

<< Target >>
Line#:38 R.Time:35.529(Scan#:7568) Retention Index:1539 MassPeaks:222
RawMode:Averaged 35.525-35.533(7567-7569) BasePeak:93.10(15137)
BG Mode:Calc. from Peak Group 1 - Event 1 Scan

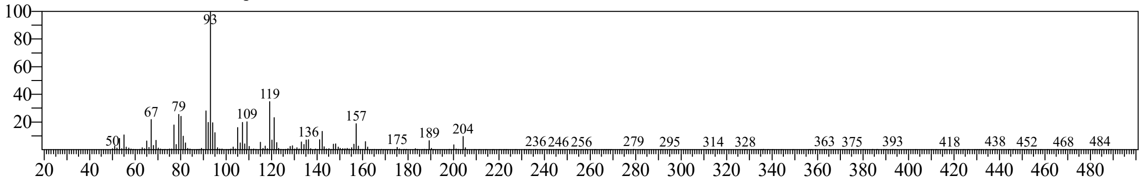

Hit#:1 Entry:1688 Library:FFNSC 3.lib
SI:96 Formula:C15 H24 CAS:25532-79-0 MolWeight:204 RetIndex:1540
CompName:Bisabolene <(E)-, alpha->

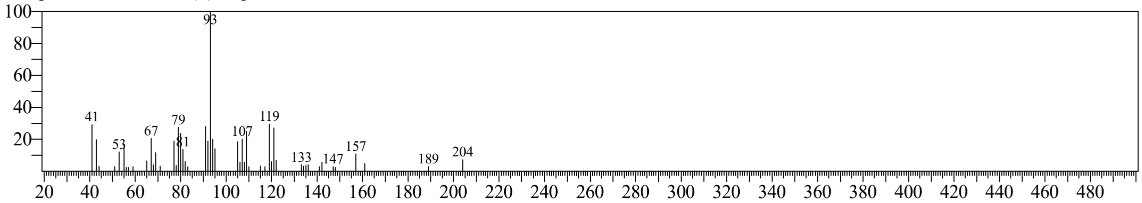

Hit#:2 Entry:104734 Library:NIST17-1.lib
SI:86 Formula:C7H7F5N2OS CAS:90598-04-2 MolWeight:262 RetIndex:0
CompName:(3-Phenylureido)sulfur pentafluoride $$ N-(Pentafluorosulfanyl)-N'-phenylurea $$ Sulfur, pentafluoro(phenylureato-N')- $$ Urea, N-phenyl-N'-

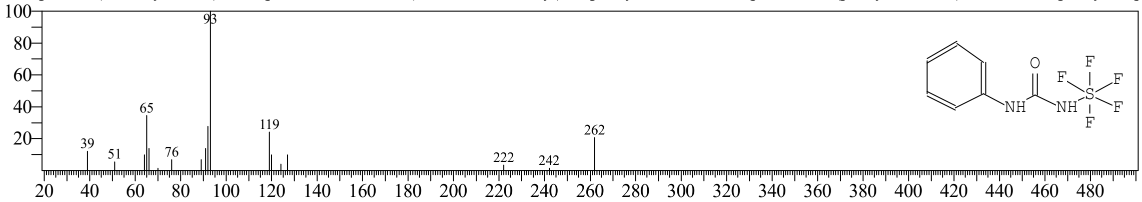

Hit#:3 Entry:52778 Library:NIST17-1.lib
SI:86 Formula:C15H24 CAS:53585-13-0 MolWeight:204 RetIndex:1534
CompName:(E)-1-Methyl-4-(6-methylhept-5-en-2-ylidene)cyclohex-1-ene $$ Cyclohexene, 4-(1,5-dimethyl-4-hexen-1-ylidene)-1-methyl-, (E)- $$ Cyclohex

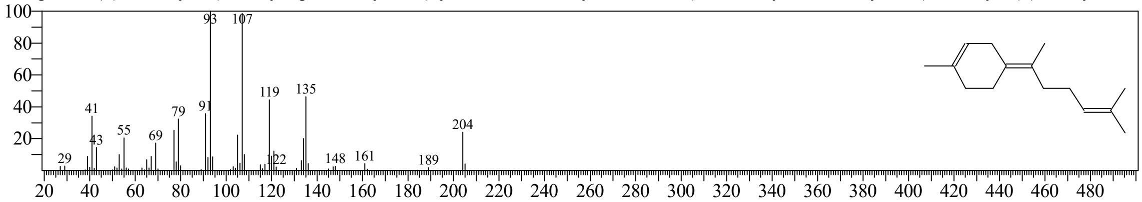

Hit#:4 Entry:34549 Library:NIST17-1.lib
SI:86 Formula:C10H12O3 CAS:66407-26-9 MolWeight:180 RetIndex:1539
CompName:Cyclopenta[c]pyran-1,3-dione, 4,4a,5,6-tetrahydro-4,7-dimethyl- $$ 4,7-Dimethyl-4,4a,5,6-tetrahydrocyclopenta[c]pyran-1,3-dione # $$

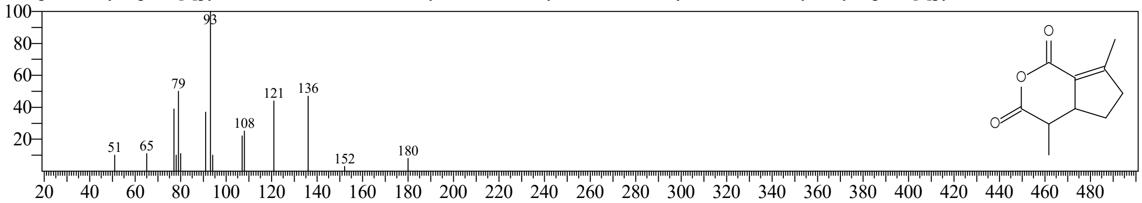

Hit#:5 Entry:24214 Library:NIST17-1.lib
SI:86 Formula:C10H16N2 CAS:87143-58-6 MolWeight:164 RetIndex:0
CompName:3,5-Methanocyclopentapyrazole, 3,3a,4,5,6,6a-hexahydro-3a,4,4-trimethyl-

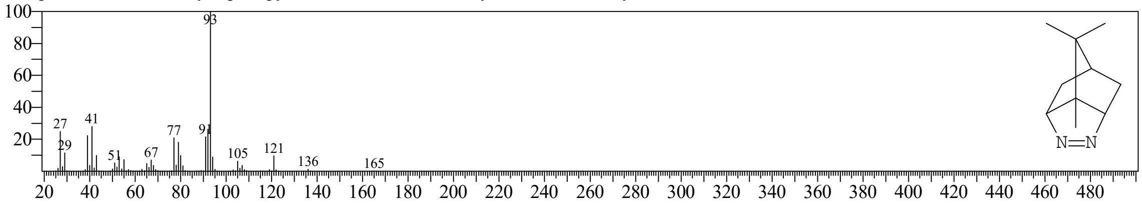

Qualitative Analysis Report

<< Target >>
Line#:39 R.Time:36.304(Scan#:7754) Retention Index:1559 MassPeaks:310
RawMode:Averaged 36.300-36.308(7753-7755) BasePeak:69.10(54866)
BG Mode:Calc. from Peak Group 1 - Event 1 Scan

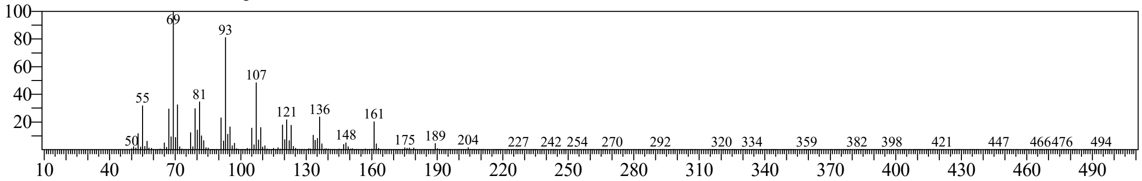

Hit#:1 Entry:228 Library:NIST17-1.lib
SI:99 Formula:C2H3N3 CAS:288-36-8 MolWeight:69 RetIndex:0
CompName:1H-1,2,3-Triazole $$ V-triazole $$ Osotriazole $$ Pyrrodiazole $$ Triazacyclopentadiene $$ 1,2,3-Triazole $$

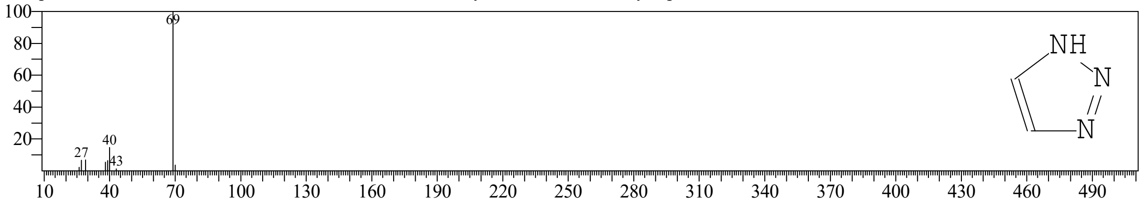

Hit#:2 Entry:1501 Library:FFNSC 3.lib
SI:97 Formula:C15 H26 O CAS:40716-66-3 MolWeight:222 RetIndex:1561
CompName:Nerolidol <(E)->

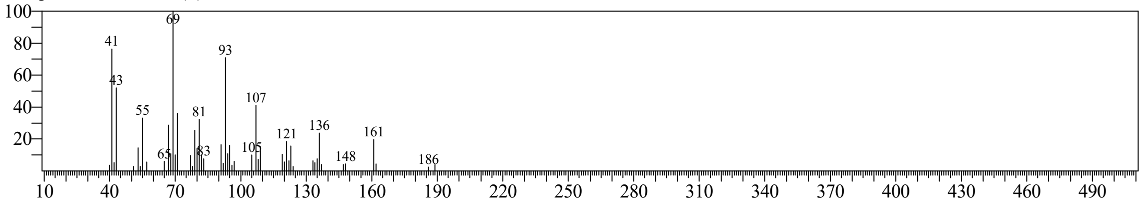

Hit#:3 Entry:981 Library:NIST17-1.lib
SI:95 Formula:CF4 CAS:75-73-0 MolWeight:88 RetIndex:0
CompName:Tetrafluoromethane $$ Carbon tetrafluoride $$ Methane, tetrafluoro- $$ Arcton 0 $$ Carbon fluoride (CF4) $$ Freon 14 $$ FC 14 $$ Perfluoro

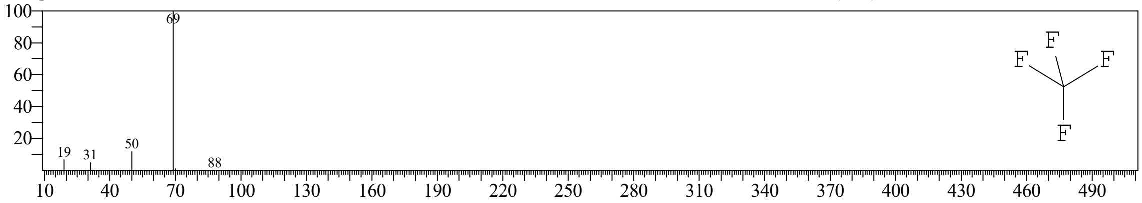

Hit#:4 Entry:10455 Library:NIST17-1.lib
SI:93 Formula:CClF4N CAS:13880-72-3 MolWeight:137 RetIndex:0
CompName:N-Chloro-N-fluoro-trifluoromethylamine $$ N-Chloro-N-tetrafluoromethanamine # $$

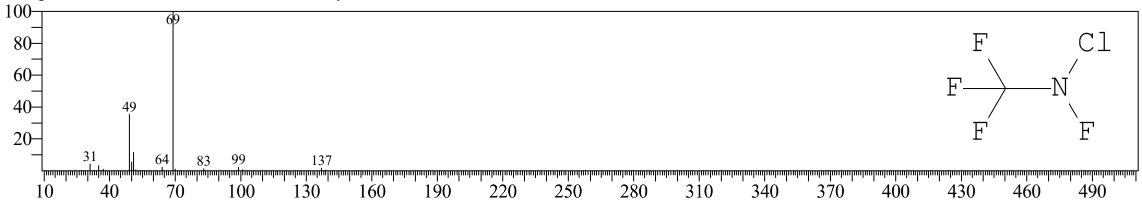

Hit#:5 Entry:10642 Library:NIST17-1.lib
SI:92 Formula:CF5P CAS:1112-04-5 MolWeight:138 RetIndex:0
CompName:Trifluoromethyldifluorophosphine $$ Trifluoromethylphosphonous difluoride # $$

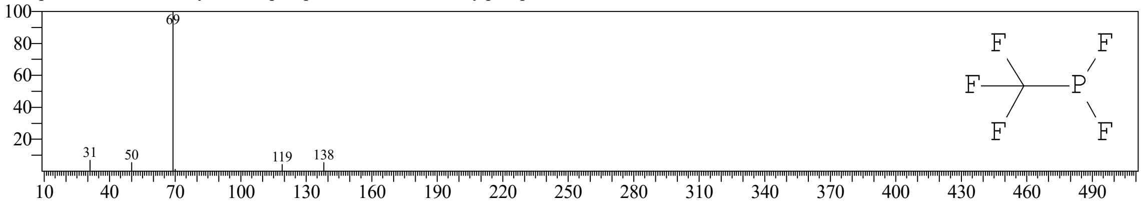

Qualitative Analysis Report

<< Target >>
Line#:40 R.Time:36.658(Scan#:7839) Retention Index:1568 MassPeaks:294
RawMode:Averaged 36.654-36.663(7838-7840) BasePeak:69.10(1566)
BG Mode:Calc. from Peak Group 1 - Event 1 Scan

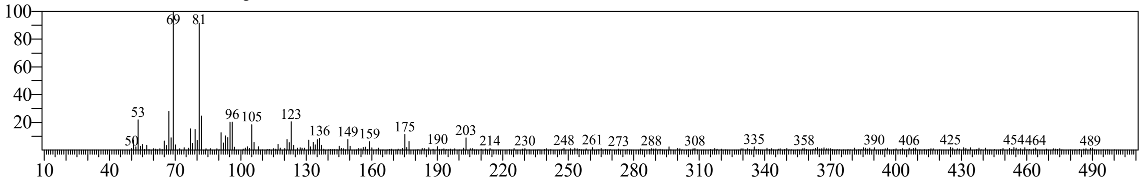

Hit#:1 Entry:228 Library:NIST17-1.lib
SI:100 Formula:C2H3N3 CAS:288-36-8 MolWeight:69 RetIndex:0
CompName:1H-1,2,3-Triazole $$ V-triazole $$ Osotriazole $$ Pyrrodiazole $$ Triazacyclopentadiene $$ 1,2,3-Triazole $$

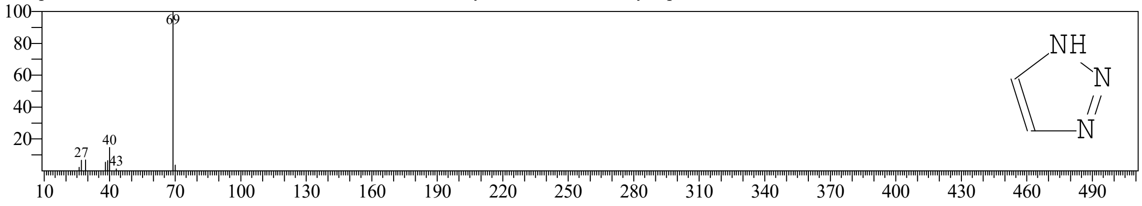

Hit#:2 Entry:981 Library:NIST17-1.lib
SI:96 Formula:CF4 CAS:75-73-0 MolWeight:88 RetIndex:0
CompName:Tetrafluoromethane $$ Carbon tetrafluoride $$ Methane, tetrafluoro- $$ Arcton 0 $$ Carbon fluoride (CF4) $$ Freon 14 $$ FC 14 $$ Perfluoro

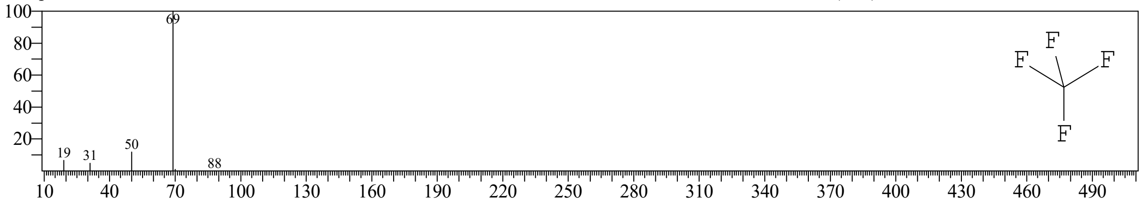

Hit#:3 Entry:10455 Library:NIST17-1.lib
SI:95 Formula:CClF4N CAS:13880-72-3 MolWeight:137 RetIndex:0
CompName:N-Chloro-N-fluoro-trifluoromethylamine $$ N-Chloro-N-tetrafluoromethanamine # $$

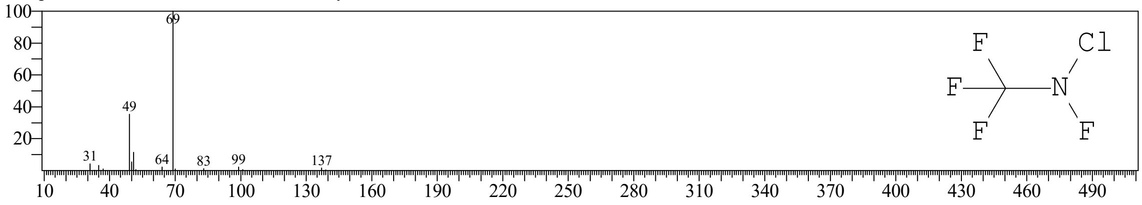

Hit#:4 Entry:10642 Library:NIST17-1.lib
SI:94 Formula:CF5P CAS:1112-04-5 MolWeight:138 RetIndex:0
CompName:Trifluoromethyldifluorophosphine $$ Trifluoromethylphosphonous difluoride # $$

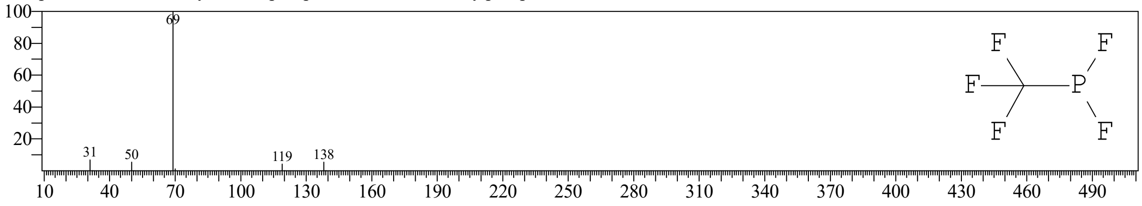

Hit#:5 Entry:13581 Library:NIST17-1.lib
SI:91 Formula:C6H12N2O2 CAS:108201-44-1 MolWeight:144 RetIndex:0
CompName:Diazene, 1-cyclopentyl-2-methoxy-, 1-oxide $$ 1-Cyclopentyl-2-methoxydiazene 1-oxide # $$

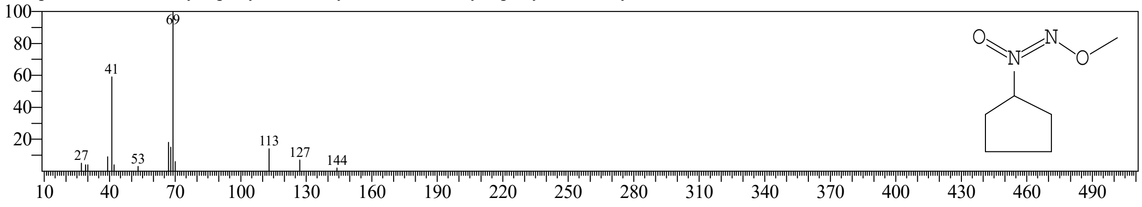

Qualitative Analysis Report

<< Target >>
Line#:41 R.Time:36.971(Scan#:7914) Retention Index:1576 MassPeaks:363
RawMode:Averaged 36.967-36.975(7913-7915) BasePeak:91.05(5019)
BG Mode:Calc. from Peak Group 1 - Event 1 Scan

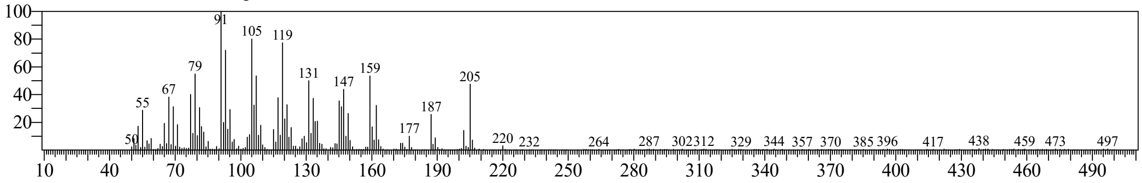

Hit#:1 Entry:903 Library:FFNSC 3.lib
SI:94 Formula:C15 H24 O CAS:72203-24-8 MolWeight:220 RetIndex:1576
CompName:Spathulenol

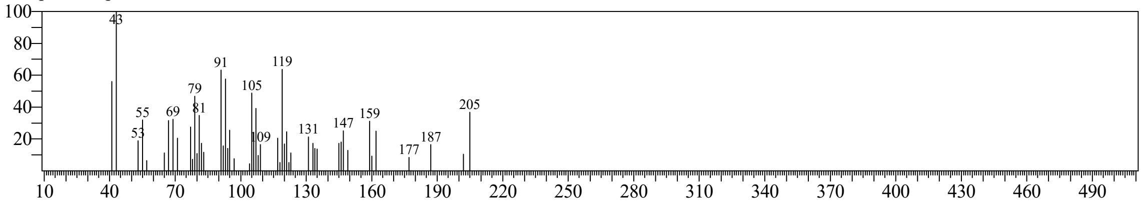

Hit#:2 Entry:31751 Library:NIST17-1.lib
SI:91 Formula:C9H8N2O2 CAS:16844-42-1 MolWeight:176 RetIndex:0
CompName:Sydnone, 3-(phenylmethyl)- $$ Sydnone, 3-benzyl- $$ Benzyl sydnone $$ 3-Benzylsydnone $$ 3-Benzyl-1,2,3-oxadiazol-3-ium-5-olate # $$

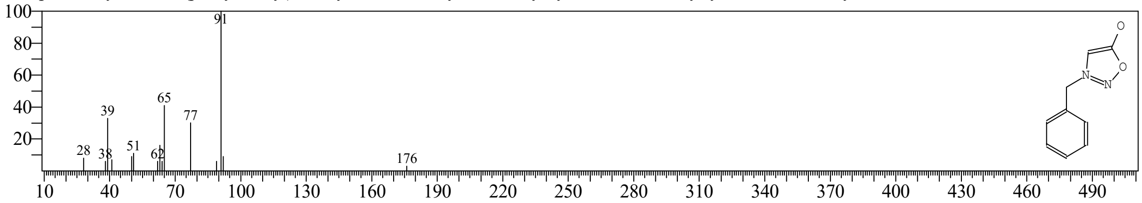

Hit#:3 Entry:23155 Library:NIST17-1.lib
SI:90 Formula:C12H18 CAS:61227-88-1 MolWeight:162 RetIndex:0
CompName:3,4-Nonadien-6-yne, 5-ethyl-3-methyl- $$ 5-Ethyl-3-methyl-3,4-nonadien-6-yne # $$

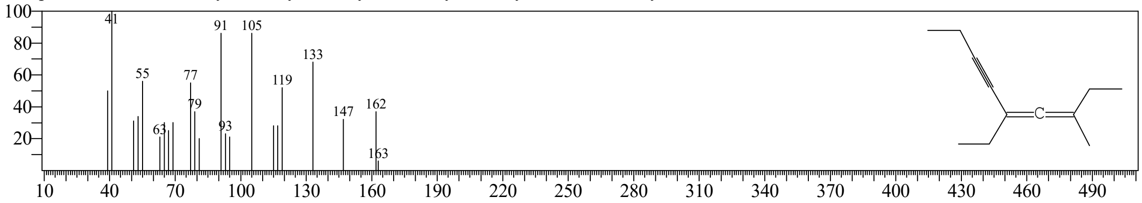

Hit#:4 Entry:79374 Library:NIST17-1.lib
SI:90 Formula:C16H26O CAS:300349-20-6 MolWeight:234 RetIndex:1573
CompName:Khusimyl methyl ether $$ (3S,3aR,6R,8aS)-3-(Methoxymethyl)-7,7-dimethyl-8-methyleneoctahydro-1H-3a,6-methanoazulene $$ 1H-3a,6-Metl

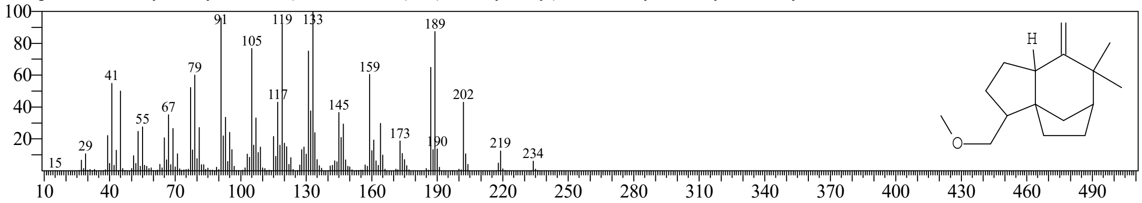

Hit#:5 Entry:41828 Library:NIST17-1.lib
SI:89 Formula:C12H18N2 CAS:0-00-0 MolWeight:190 RetIndex:0
CompName:4,5,6,7-Tetrahydroindazole-3-spirocyclohexane

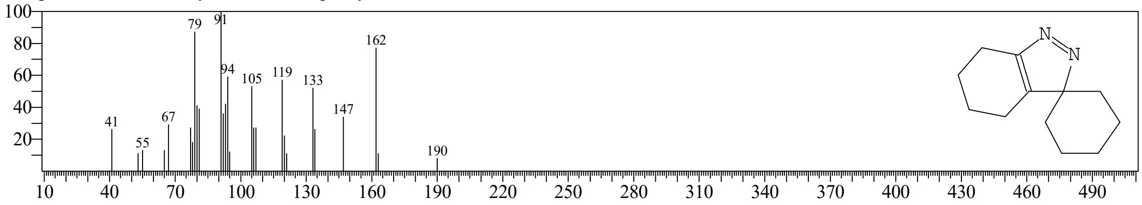

Qualitative Analysis Report

<< Target >>
Line#:42   R.Time:37.188(Scan#:7966) Retention Index:1581 MassPeaks:263
RawMode:Averaged 37.183-37.192(7965-7967) BasePeak:109.15(1448)
BG Mode:Calc. from Peak Group 1 - Event 1 Scan

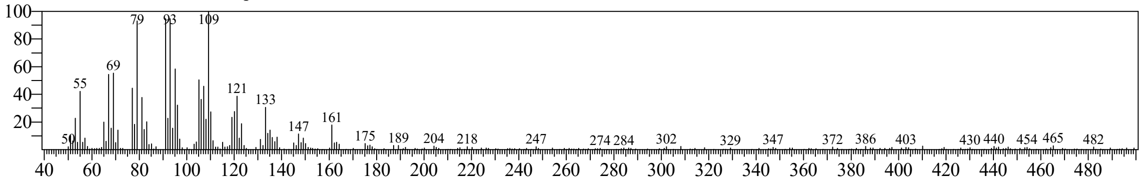

Hit#:1 Entry:145 Library:FFNSC 3.lib
SI:93 Formula:C15 H24 O CAS:1139-30-6 MolWeight:220 RetIndex:1587
CompName:Caryophyllene oxide

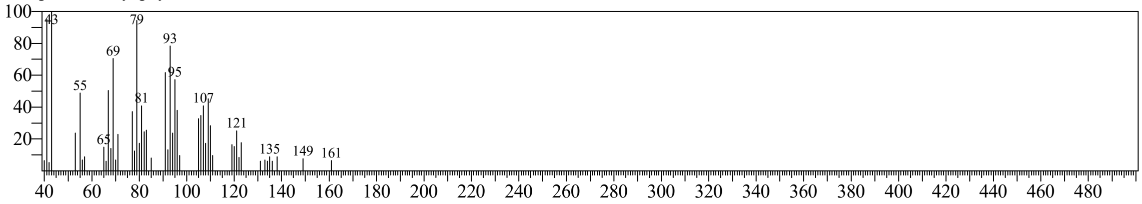

Hit#:2 Entry:2869 Library:FFNSC 3.lib
SI:89 Formula:C15 H26 O CAS:39703-23-6 MolWeight:222 RetIndex:1572
CompName:Longipinanol

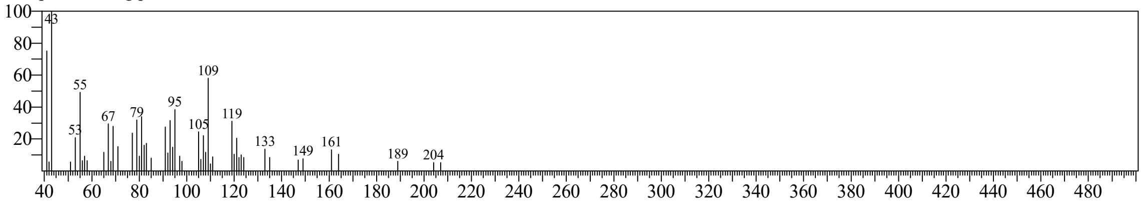

Hit#:3 Entry:2557 Library:FFNSC 3.lib
SI:86 Formula:C15 H26 O CAS:78392-83-3 MolWeight:222 RetIndex:1581
CompName:Thujopsan-2-alpha-ol

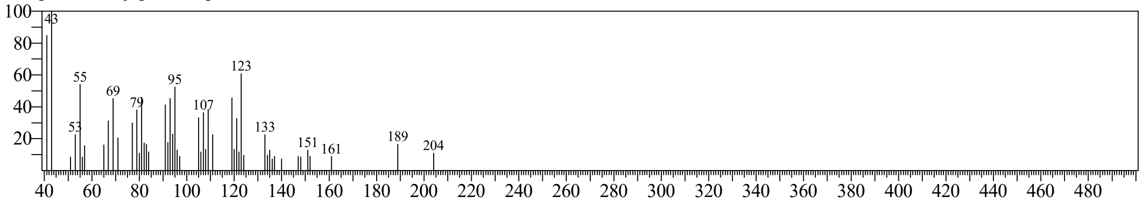

Hit#:4 Entry:43322 Library:NIST17-1.lib
SI:86 Formula:C13H20O CAS:63922-50-9 MolWeight:192 RetIndex:0
CompName:3,4-Heptadien-2-one, 3-cyclopentyl-6-methyl- $$ 3-Cyclopentyl-6-methyl-3,4-heptadien-2-one # $$

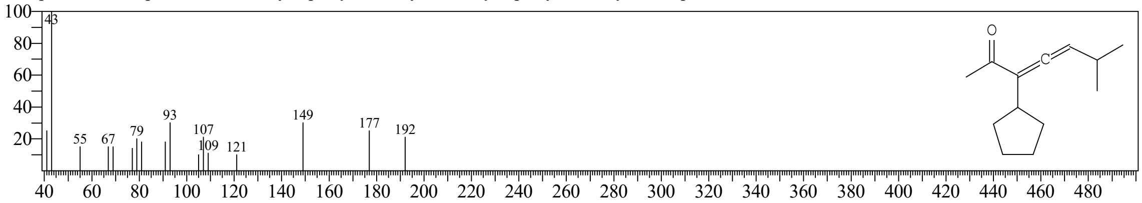

Hit#:5 Entry:903 Library:FFNSC 3.lib
SI:86 Formula:C15 H24 O CAS:72203-24-8 MolWeight:220 RetIndex:1576
CompName:Spathulenol

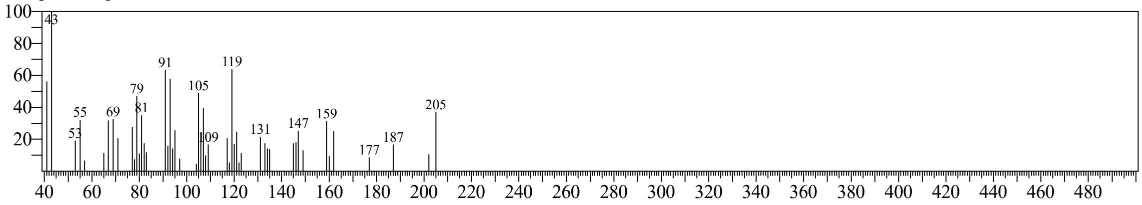

Qualitative Analysis Report

<< Target >>
Line#:43 R.Time:37.329(Scan#:8000) Retention Index:1585 MassPeaks:274
RawMode:Averaged 37.325-37.333(7999-8001) BasePeak:107.10(1722)
BG Mode:Calc. from Peak Group 1 - Event 1 Scan

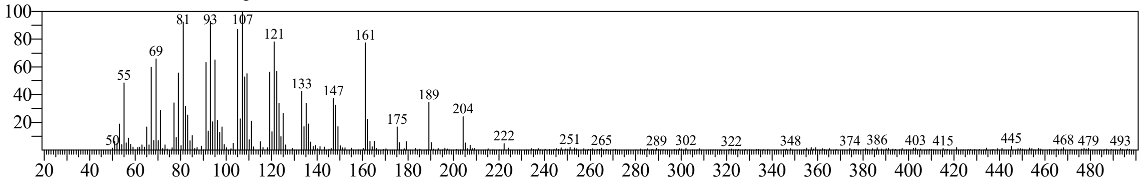

Hit#:1 Entry:2932 Library:FFNSC 3.lib
SI:91 Formula:C15 H26 O CAS:51371-47-2 MolWeight:222 RetIndex:1592
CompName:Globulol

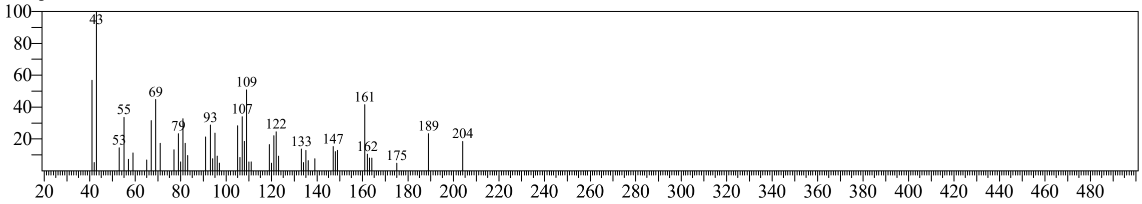

Hit#:2 Entry:1074 Library:FFNSC 3.lib
SI:91 Formula:C15 H26 O CAS:19078-39-8 MolWeight:222 RetIndex:1594
CompName:Viridiflorol

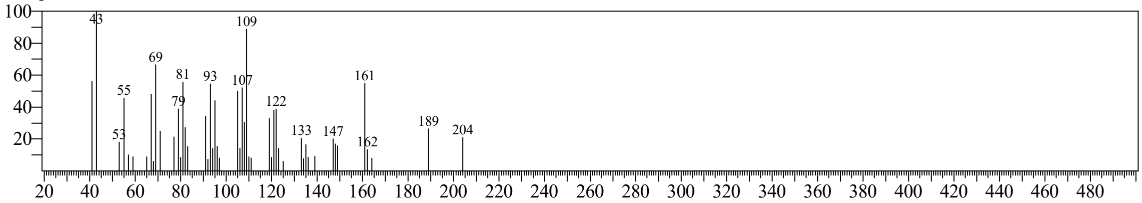

Hit#:3 Entry:2557 Library:FFNSC 3.lib
SI:89 Formula:C15 H26 O CAS:78392-83-3 MolWeight:222 RetIndex:1581
CompName:Thujopsan-2-alpha-ol

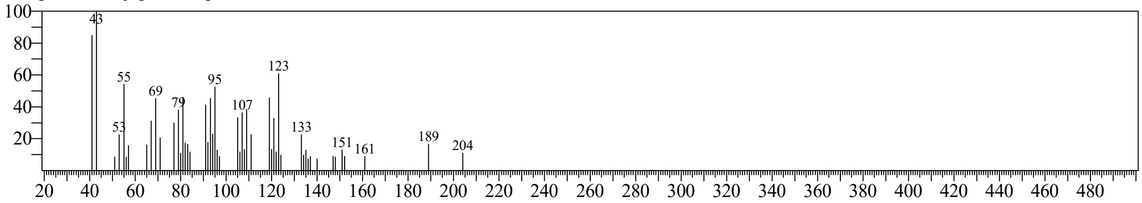

Hit#:4 Entry:68643 Library:NIST17-1.lib
SI:88 Formula:C15H26O CAS:26804-55-7 MolWeight:222 RetIndex:1593
CompName:1,4-Methanoazulen-7-ol, decahydro-1,5,5,8a-tetramethyl-, [1s-(1.alpha.,3a.beta.,4.alpha.,7.beta.,8a.beta.)]-

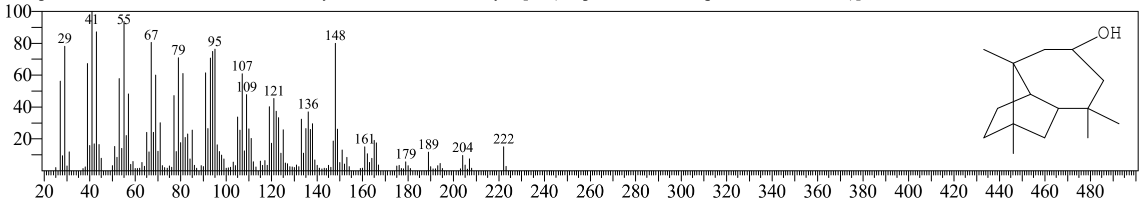

Hit#:5 Entry:2939 Library:NIST17-1.lib
SI:88 Formula:C6H8Si CAS:63878-65-9 MolWeight:108 RetIndex:0
CompName:Silabenzene,1-methyl- $$ 1-Methyl-1-silabenzene $$ 1-Methylsiline # $$

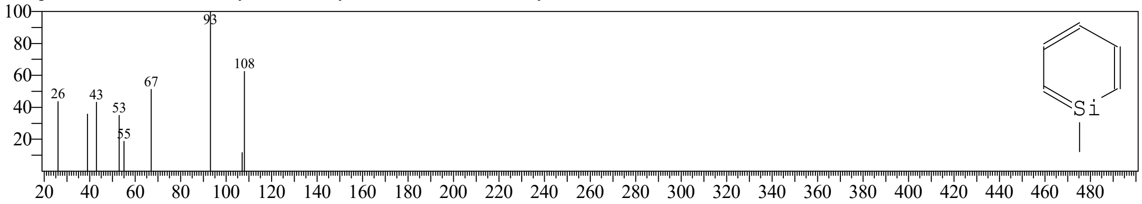

Qualitative Analysis Report

<< Target >>
Line#:44 R.Time:37.679(Scan#:8084) Retention Index:1593 MassPeaks:331
RawMode:Averaged 37.675-37.683(8083-8085) BasePeak:107.10(4318)
BG Mode:Calc. from Peak Group 1 - Event 1 Scan

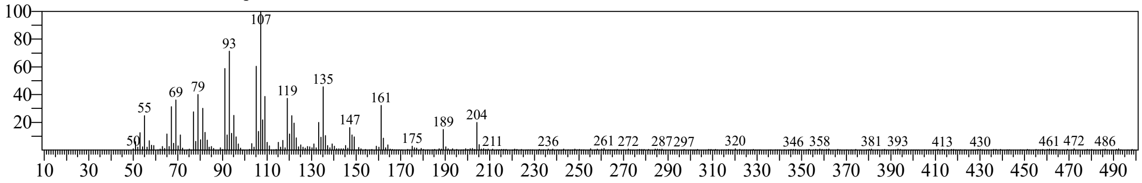

Hit#:1 Entry:52646 Library:NIST17-1.lib
SI:91 Formula:C13H20N2 CAS:0-00-0 MolWeight:204 RetIndex:0
CompName:1,4-Methanocycloocta[d]pyridazine, 1,4,4a,5,6,9,10,10a-octahydro-11,11-dimethyl-, (1.alpha.,4.alpha.,4a.alpha.,10a.alpha.)-

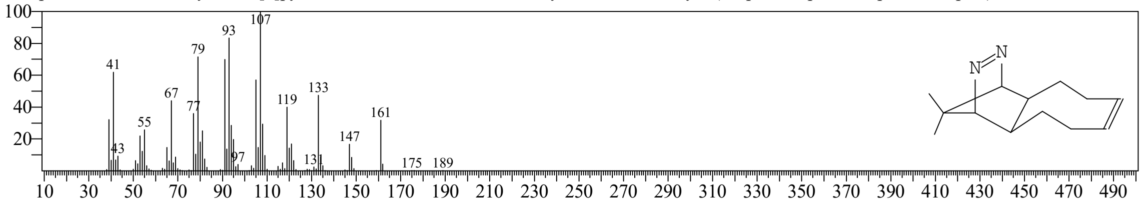

Hit#:2 Entry:7085 Library:NIST17-1.lib
SI:91 Formula:F5P CAS:7647-19-0 MolWeight:126 RetIndex:0
CompName:Phosphorus pentafluoride $$ Phosphorus(V) fluoride $$ PF5 $$ Phosphorane, pentafluoro- $$ UN 2198 $$ Phosphorus fluoride $$ Pentafluoro

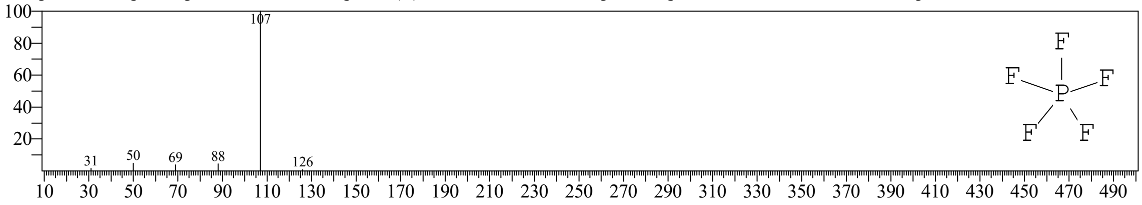

Hit#:3 Entry:52871 Library:NIST17-1.lib
SI:89 Formula:C15H24 CAS:15423-57-1 MolWeight:204 RetIndex:1603
CompName:1,5-Cyclodecadiene, 1,5-dimethyl-8-(1-methylethylidene)-, (E,E)- $$ Germacrene B $$ Germacra-1(10),4,7(11)-triene $$ Germacra-1(10),4,7(1

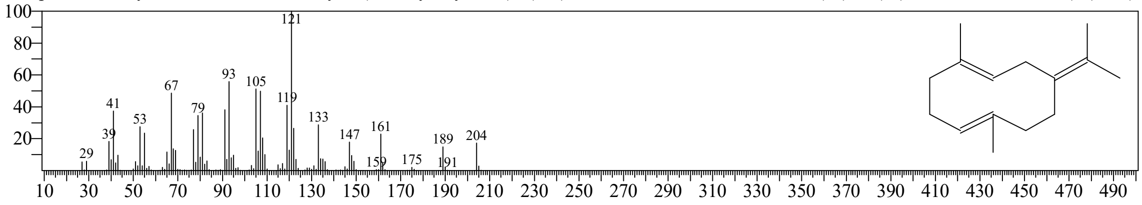

Hit#:4 Entry:68744 Library:NIST17-1.lib
SI:89 Formula:C15H26O CAS:28400-11-5 MolWeight:222 RetIndex:1598
CompName:.beta.-Acorenol $$ 2-((1R,4R,5R)-4,8-Dimethylspiro[4.5]dec-7-en-1-yl)propan-2-ol $$ Spiro[4.5]dec-7-ene-1-methanol, .alpha.,.alpha.,4,8-tetra

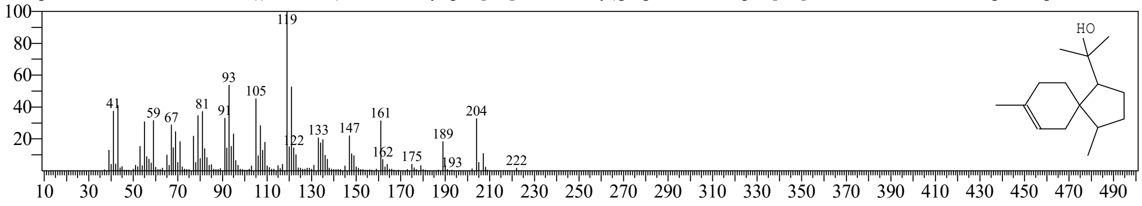

Hit#:5 Entry:9872 Library:NIST17-1.lib
SI:88 Formula:C2H5F4P CAS:0-00-0 MolWeight:136 RetIndex:0
CompName:Ethyltetrafluorophosphorane

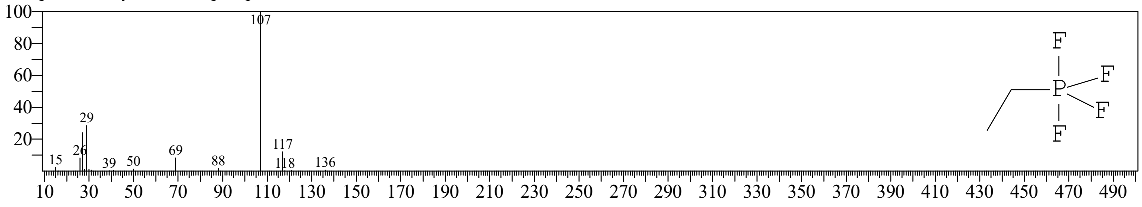

Qualitative Analysis Report

<< Target >>
Line#:45 R.Time:38.121(Scan#:8190) Retention Index:1605 MassPeaks:308
RawMode:Averaged 38.117-38.125(8189-8191) BasePeak:149.15(1061)
BG Mode:Calc. from Peak Group 1 - Event 1 Scan

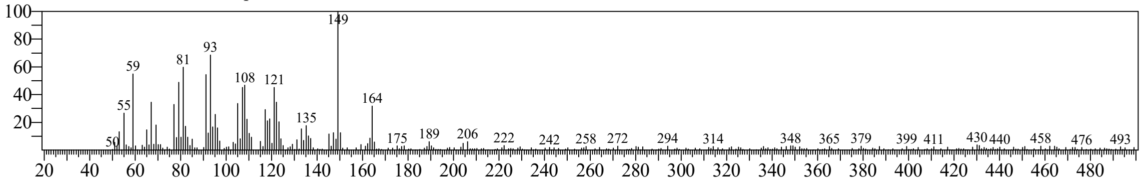

Hit#:1 Entry:15775 Library:NIST17-1.lib
SI:92 Formula:C8H7NO2 CAS:69022-83-9 MolWeight:149 RetIndex:0
CompName:Furo[3,2-b]pyridine, 2-methyl-, 4-oxide $$ 2-Methylfuro(3,2-b)pyridin-N-oxide $$ 2-Methylfuro[3,2-b]pyridine 4-oxide # $$

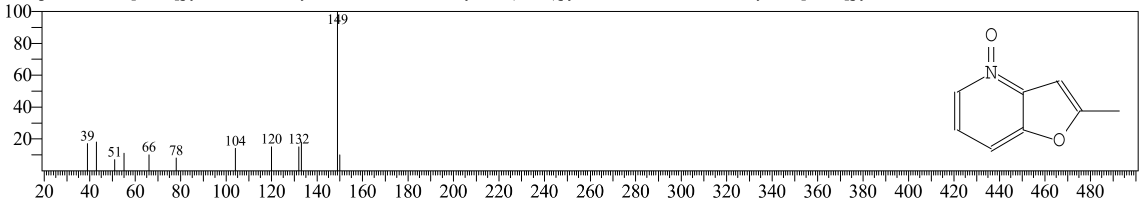

Hit#:2 Entry:68762 Library:NIST17-1.lib
SI:91 Formula:C15H26O CAS:194607-96-0 MolWeight:222 RetIndex:1598
CompName:2-((4aS,8R,8aR)-4a,8-Dimethyl-3,4,4a,5,6,7,8,8a-octahydronaphthalen-2-yl)propan-2-ol $$ cis-Eudesm-6-en-11-ol $$

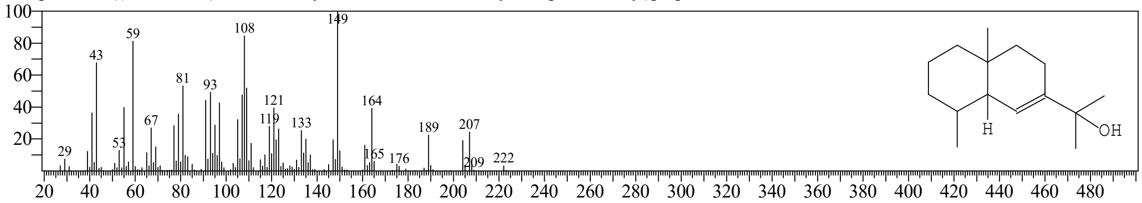

Hit#:3 Entry:43322 Library:NIST17-1.lib
SI:89 Formula:C13H20O CAS:63922-50-9 MolWeight:192 RetIndex:0
CompName:3,4-Heptadien-2-one, 3-cyclopentyl-6-methyl- $$ 3-Cyclopentyl-6-methyl-3,4-heptadien-2-one # $$

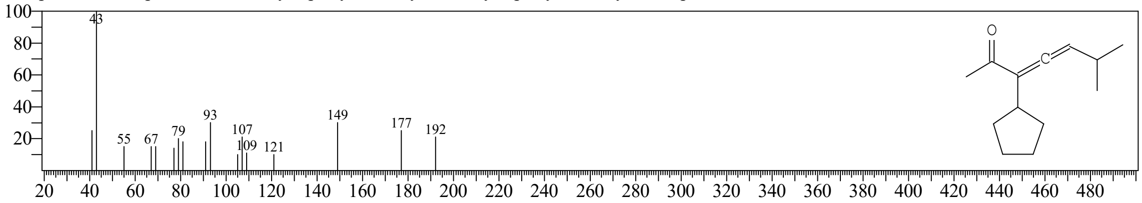

Hit#:4 Entry:43294 Library:NIST17-1.lib
SI:89 Formula:C12H20N2 CAS:0-00-0 MolWeight:192 RetIndex:0
CompName:1,4-Methano-1H-cyclohepta[d]pyridazine, 4,4a,5,6,7,8,9,9a-octahydro-10,10-dimethyl-

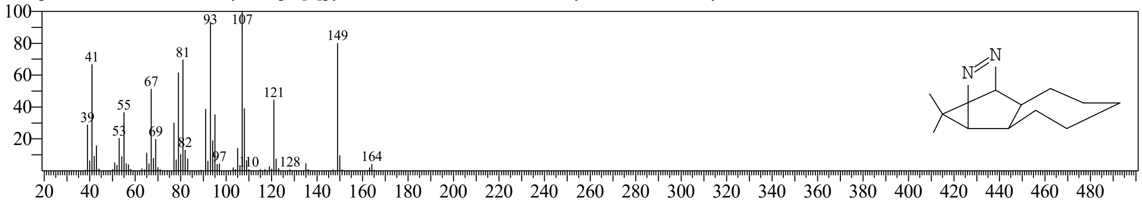

Hit#:5 Entry:3204 Library:FFNSC 3.lib
SI:86 Formula:C15 H26 O CAS:198404-98-7 MolWeight:222 RetIndex:1612
CompName:Javanol isomer II

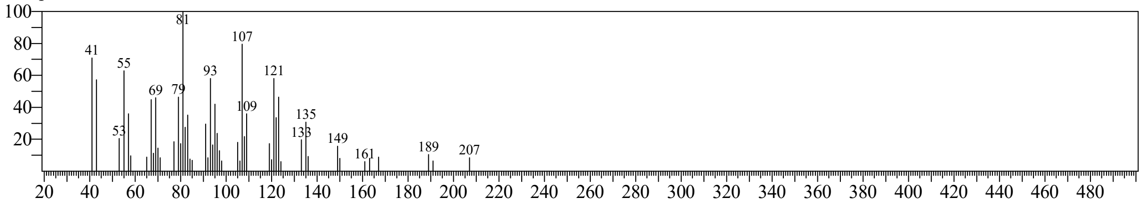

Qualitative Analysis Report

<< Target >>
Line#:46 R.Time:38.388(Scan#:8254) Retention Index:1612 MassPeaks:278
RawMode:Averaged 38.383-38.392(8253-8255) BasePeak:93.10(800)
BG Mode:Calc. from Peak Group 1 - Event 1 Scan

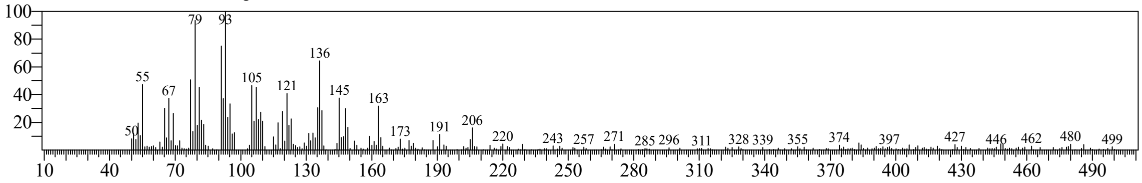

Hit#:1 Entry:54495 Library:NIST17-1.lib
SI:89 Formula:C14H22O CAS:2221-71-8 MolWeight:206 RetIndex:1622
CompName:2-Naphthalenemethanol, decahydro-5-methylene-8-vinyl- $$ Khusilol, dihydro- $$ (5-Methylene-8-vinyldecahydro-2-naphthalenyl)methanol # $

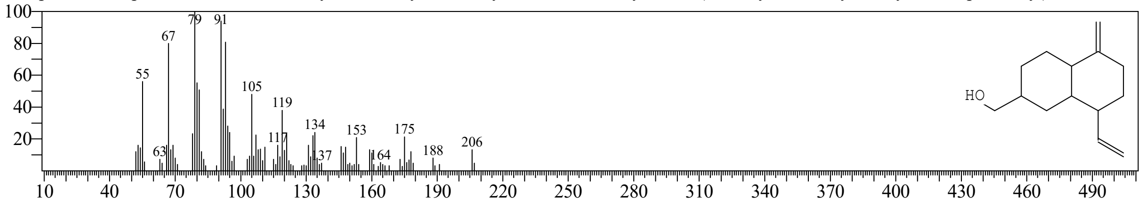

Hit#:2 Entry:5613 Library:NIST17-1.lib
SI:89 Formula:BrN3 CAS:13973-87-0 MolWeight:121 RetIndex:0
CompName:Bromine azide $$ Bromine azide (Br(N3)) $$ Bromine nitride $$ Nitrogen bromide (N3Br) $$

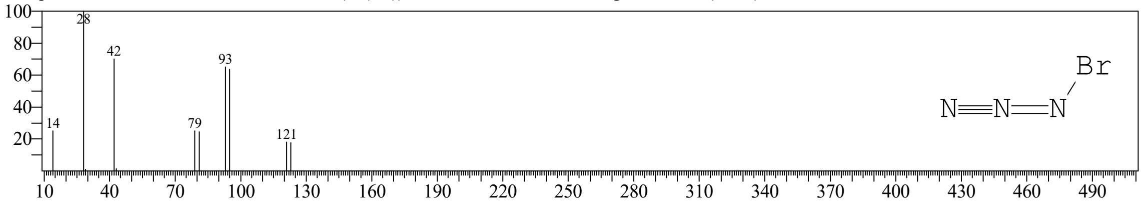

Hit#:3 Entry:2939 Library:NIST17-1.lib
SI:88 Formula:C6H8Si CAS:63878-65-9 MolWeight:108 RetIndex:0
CompName:Silabenzene,1-methyl- $$ 1-Methyl-1-silabenzene $$ 1-Methylsiline # $$

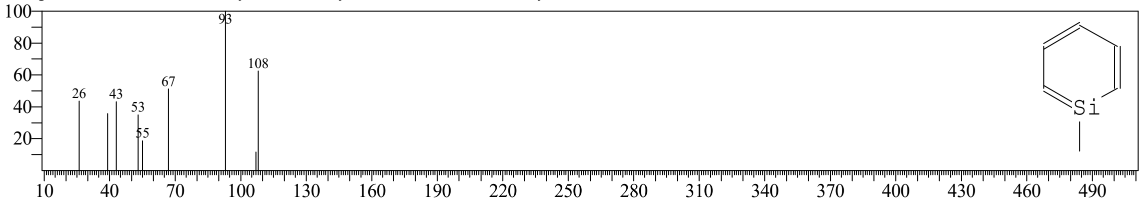

Hit#:4 Entry:38638 Library:NIST17-1.lib
SI:87 Formula:C9H14O2S CAS:0-00-0 MolWeight:186 RetIndex:0
CompName:3,4-Dimethyl-2-prop-2-enyl-2,5-dihydrothiophene 1,1-dioxide

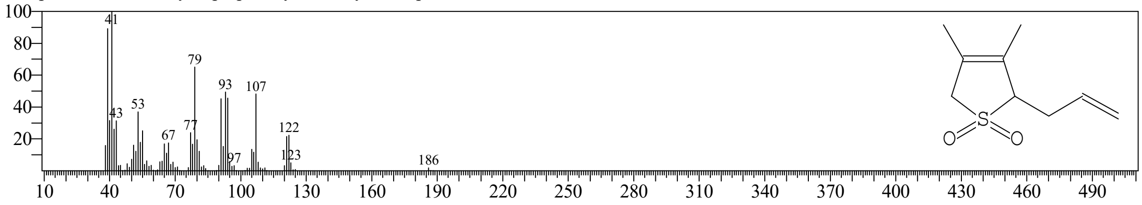

Hit#:5 Entry:52646 Library:NIST17-1.lib
SI:87 Formula:C13H20N2 CAS:0-00-0 MolWeight:204 RetIndex:0
CompName:1,4-Methanocycloocta[d]pyridazine, 1,4,4a,5,6,9,10,10a-octahydro-11,11-dimethyl-, (1.alpha.,4.alpha.,4a.alpha.,10a.alpha.)-

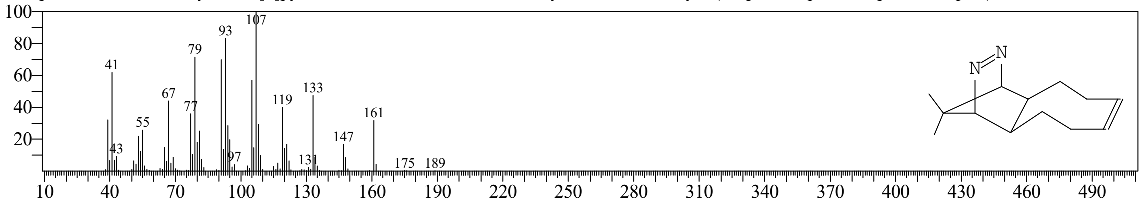

Qualitative Analysis Report

<< Target >>
Line#:47 R.Time:38.808(Scan#:8355) Retention Index:1623 MassPeaks:282
RawMode:Averaged 38.804-38.813(8354-8356) BasePeak:69.10(40662)
BG Mode:Calc. from Peak Group 1 - Event 1 Scan

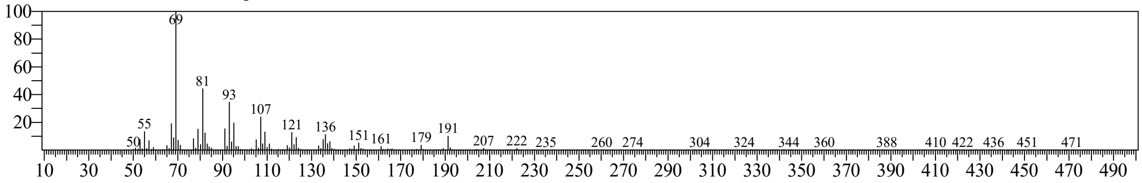

Hit#:1 Entry:228 Library:NIST17-1.lib
SI:99 Formula:C2H3N3 CAS:288-36-8 MolWeight:69 RetIndex:0
CompName:1H-1,2,3-Triazole $$ V-triazole $$ Osotriazole $$ Pyrrodiazole $$ Triazacyclopentadiene $$ 1,2,3-Triazole $$

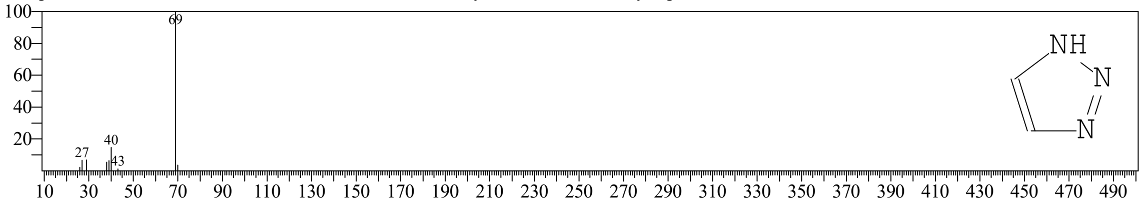

Hit#:2 Entry:10642 Library:NIST17-1.lib
SI:97 Formula:CF5P CAS:1112-04-5 MolWeight:138 RetIndex:0
CompName:Trifluoromethyldifluorophosphine $$ Trifluoromethylphosphonous difluoride # $$

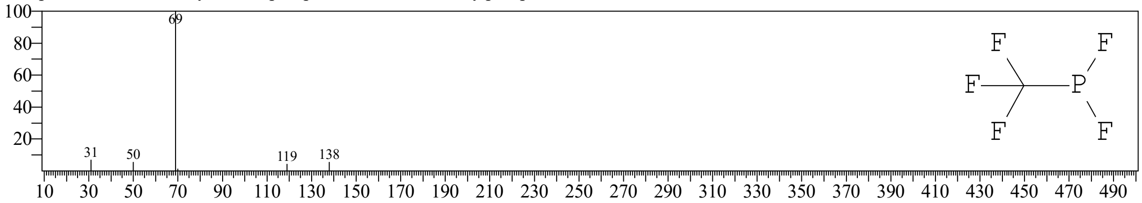

Hit#:3 Entry:981 Library:NIST17-1.lib
SI:96 Formula:CF4 CAS:75-73-0 MolWeight:88 RetIndex:0
CompName:Tetrafluoromethane $$ Carbon tetrafluoride $$ Methane, tetrafluoro- $$ Arcton 0 $$ Carbon fluoride (CF4) $$ Freon 14 $$ FC 14 $$ Perfluoro

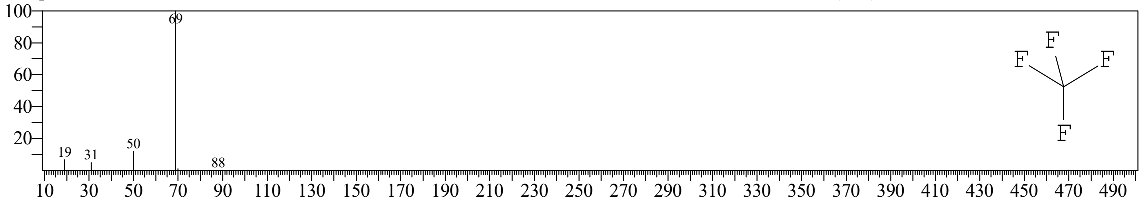

Hit#:4 Entry:2164 Library:FFNSC 3.lib
SI:94 Formula:C15 H26 O CAS:120707-27-9 MolWeight:222 RetIndex:1633
CompName:Sesquilavandulol <(E)->

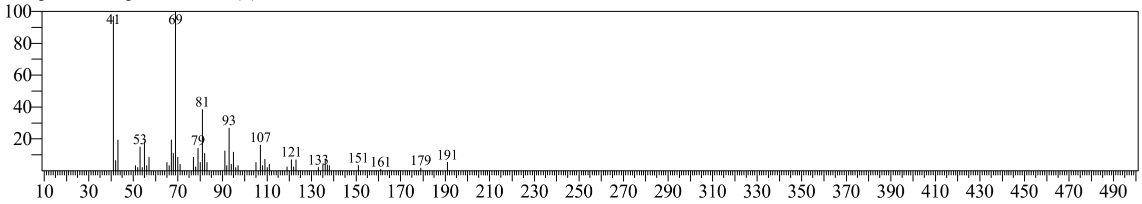

Hit#:5 Entry:13581 Library:NIST17-1.lib
SI:94 Formula:C6H12N2O2 CAS:108201-44-1 MolWeight:144 RetIndex:0
CompName:Diazene, 1-cyclopentyl-2-methoxy-, 1-oxide $$ 1-Cyclopentyl-2-methoxydiazene 1-oxide # $$

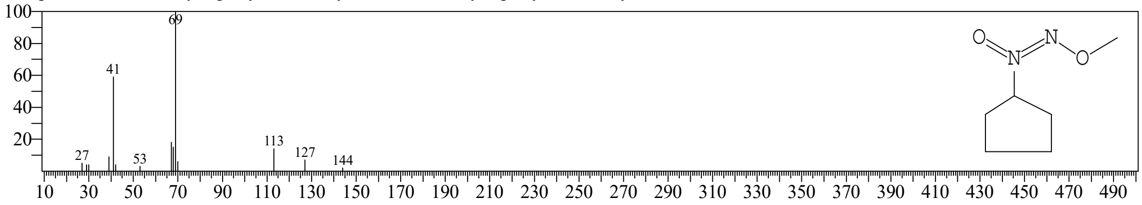

Qualitative Analysis Report

<< Target >>
Line#:48 R.Time:38.950(Scan#:8389) Retention Index:1627 MassPeaks:266
RawMode:Averaged 38.946-38.954(8388-8390) BasePeak:119.10(4760)
BG Mode:Calc. from Peak Group 1 - Event 1 Scan

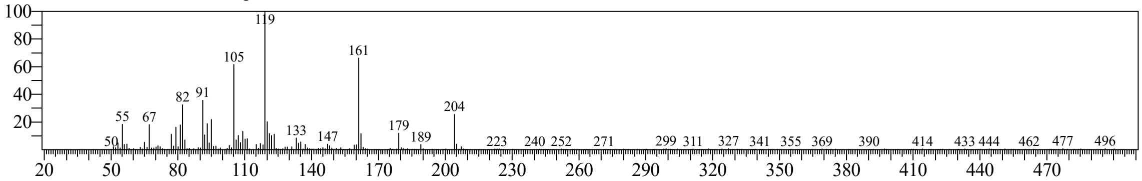

Hit#:1 Entry:1476 Library:FFNSC 3.lib
SI:95 Formula:C15 H26 O CAS:19912-67-5 MolWeight:222 RetIndex:1631
CompName:Epicubenol

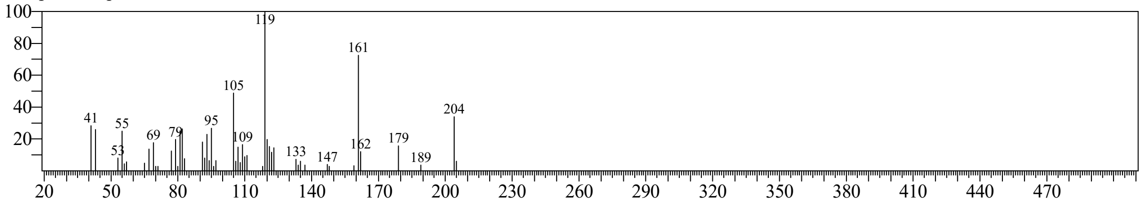

Hit#:2 Entry:906 Library:FFNSC 3.lib
SI:86 Formula:C15 H26 O CAS:121350-29-6 MolWeight:222 RetIndex:1632
CompName:Acorenol <alpha->

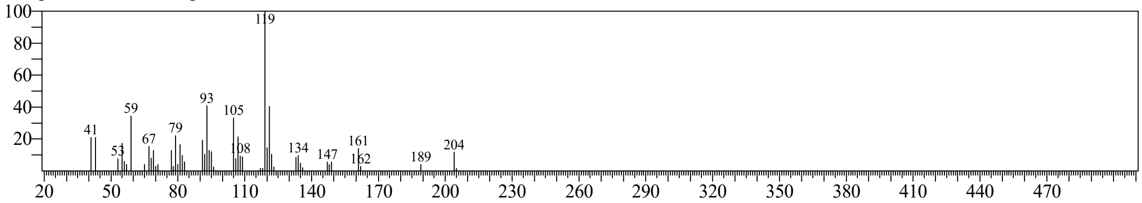

Hit#:3 Entry:5389 Library:NIST17-1.lib
SI:86 Formula:C7H5NO CAS:103-71-9 MolWeight:119 RetIndex:0
CompName:Benzene, isocyanato- $$ Isocyanic acid, phenyl ester $$ Carbanil $$ Isocyanatobenzene $$ Mondur P $$ Phenyl carbonimide $$ Phenyl isocyan

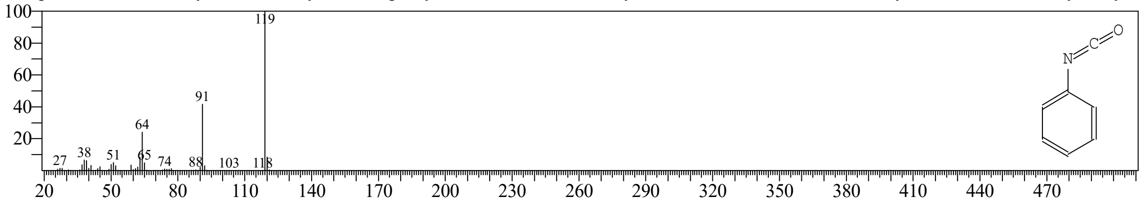

Hit#:4 Entry:293 Library:FFNSC 3.lib
SI:86 Formula:C15 H26 O CAS:28400-11-5 MolWeight:222 RetIndex:1636
CompName:Acorenol <beta->

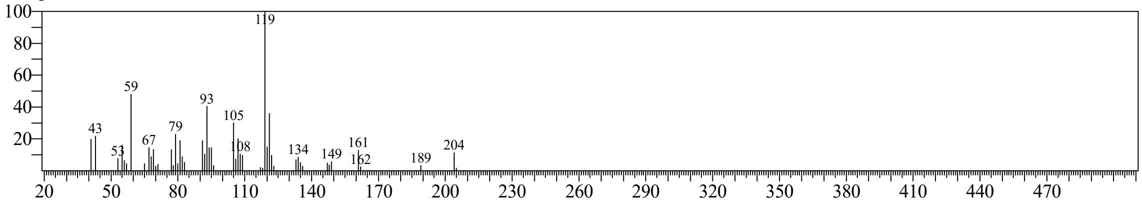

Hit#:5 Entry:41955 Library:NIST17-1.lib
SI:85 Formula:C14H22 CAS:61227-89-2 MolWeight:190 RetIndex:0
CompName:5,6-Decadien-3-yne, 5,7-diethyl- $$ 5,7-Diethyl-5,6-decadien-3-yne # $$

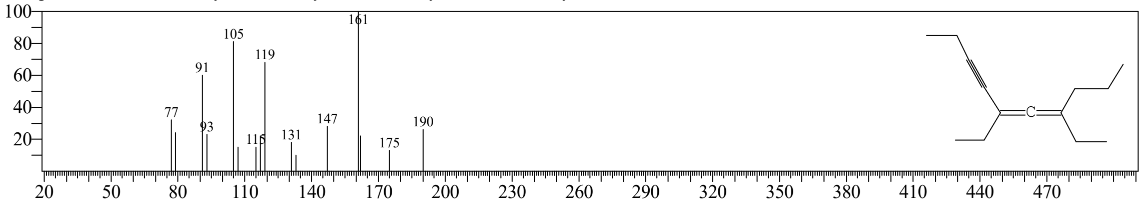

Qualitative Analysis Report

<< Target >>
Line#:49 R.Time:39.133(Scan#:8433) Retention Index:1632 MassPeaks:302
RawMode:Averaged 39.129-39.138(8432-8434) BasePeak:91.10(1204)
BG Mode:Calc. from Peak Group 1 - Event 1 Scan

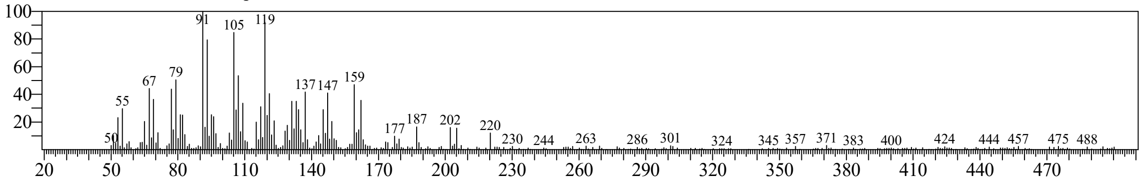

Hit#:1 Entry:31751 Library:NIST17-1.lib
SI:92 Formula:$C_9H_8N_2O_2$ CAS:16844-42-1 MolWeight:176 RetIndex:0
CompName:Sydnone, 3-(phenylmethyl)- $$ Sydnone, 3-benzyl- $$ Benzyl sydnone $$ 3-Benzylsydnone $$ 3-Benzyl-1,2,3-oxadiazol-3-ium-5-olate # $$

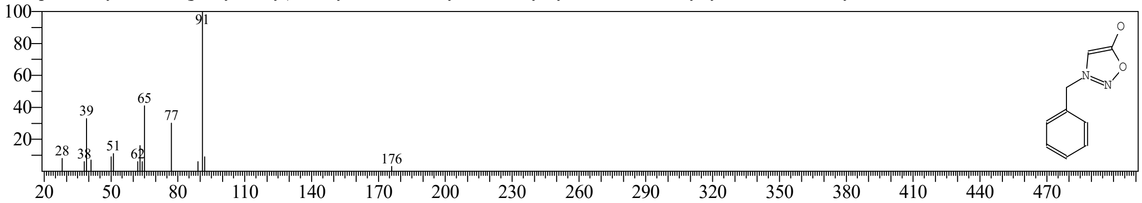

Hit#:2 Entry:23155 Library:NIST17-1.lib
SI:91 Formula:$C_{12}H_{18}$ CAS:61227-88-1 MolWeight:162 RetIndex:0
CompName:3,4-Nonadien-6-yne, 5-ethyl-3-methyl- $$ 5-Ethyl-3-methyl-3,4-nonadien-6-yne # $$

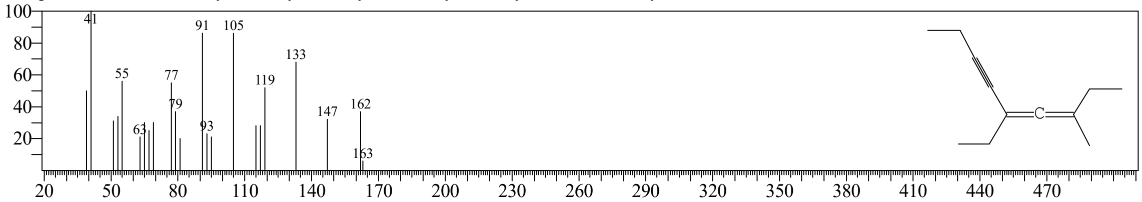

Hit#:3 Entry:66741 Library:NIST17-1.lib
SI:88 Formula:$C_{15}H_{24}O$ CAS:119120-23-9 MolWeight:220 RetIndex:1636
CompName:Eudesma-4(15),7-dien-1.beta. -ol $$ (1R,4aS,8aR)-6-Isopropyl-8a-methyl-4-methylene-1,2,3,4,4a,5,8,8a-octahydronaphthalen-1-ol $$

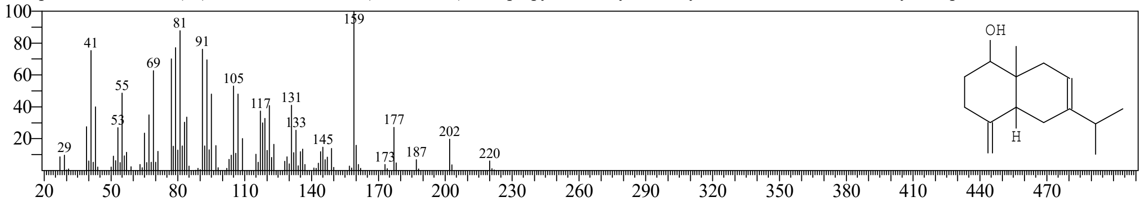

Hit#:4 Entry:56419 Library:NIST17-1.lib
SI:88 Formula:$C_{14}H_{24}O$ CAS:62924-17-8 MolWeight:208 RetIndex:1641
CompName:2-Methyl-4-(2,6,6-trimethylcyclohex-1-enyl)but-2-en-1-ol $$ (2E)-2-Methyl-4-(2,6,6-trimethyl-1-cyclohexen-1-yl)-2-buten-1-ol # $$

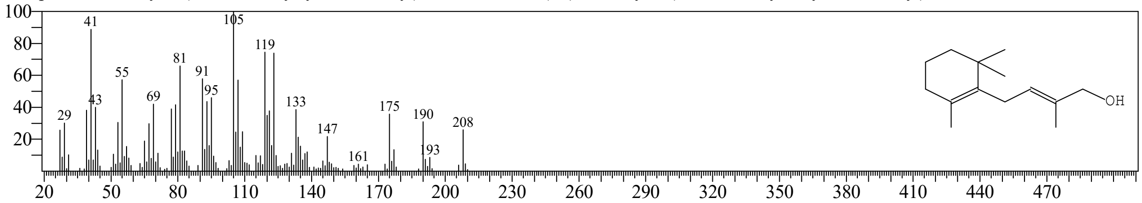

Hit#:5 Entry:231901 Library:NIST17-1.lib
SI:87 Formula:$C_{13}H_{14}Fe_2O_4S_2$ CAS:12215-56-4 MolWeight:410 RetIndex:0
CompName:Iron, [(2,3,5,6-.eta.)-bicyclo[2.2.1]hepta-2,5-diene]tetracarbonylbis[.mu.-(methanethiolato)]di-, (Fe-Fe)

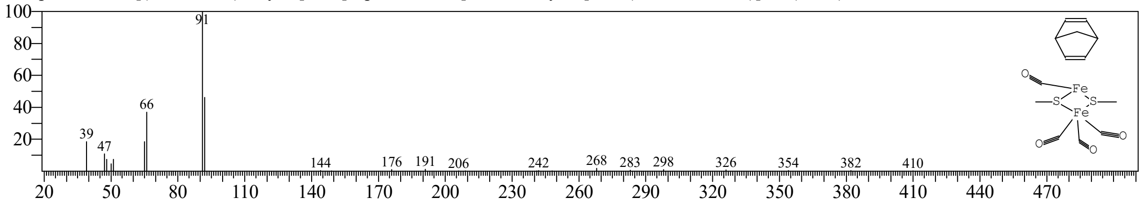

Qualitative Analysis Report

<< Target >>
Line#:50 R.Time:39.358(Scan#:8487) Retention Index:1638 MassPeaks:289
RawMode:Averaged 39.354-39.362(8486-8488) BasePeak:91.05(1755)
BG Mode:Calc. from Peak Group 1 - Event 1 Scan

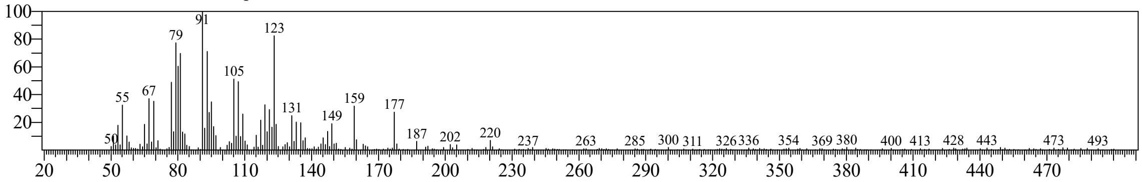

Hit#:1 Entry:31751 Library:NIST17-1.lib
SI:91 Formula:C9H8N2O2 CAS:16844-42-1 MolWeight:176 RetIndex:0
CompName:Sydnone, 3-(phenylmethyl)- $$ Sydnone, 3-benzyl- $$ Benzyl sydnone $$ 3-Benzylsydnone $$ 3-Benzyl-1,2,3-oxadiazol-3-ium-5-olate # $$

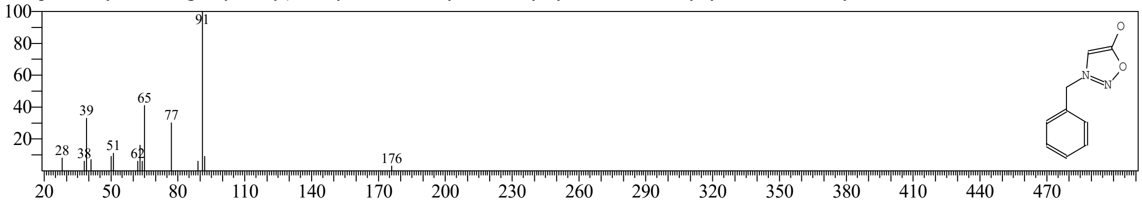

Hit#:2 Entry:2554 Library:FFNSC 3.lib
SI:89 Formula:C15 H24 O CAS:85760-81-2 MolWeight:220 RetIndex:1644
CompName:Aromandendrene epoxide <allo->

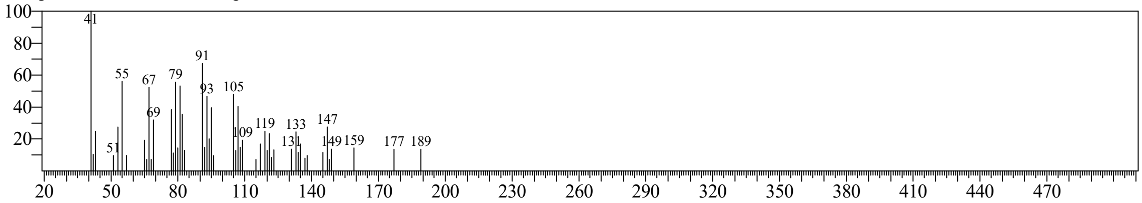

Hit#:3 Entry:66741 Library:NIST17-1.lib
SI:89 Formula:C15H24O CAS:119120-23-9 MolWeight:220 RetIndex:1636
CompName:Eudesma-4(15),7-dien-1.beta. -ol $$ (1R,4aS,8aR)-6-Isopropyl-8a-methyl-4-methylene-1,2,3,4,4a,5,8,8a-octahydronaphthalen-1-ol $$

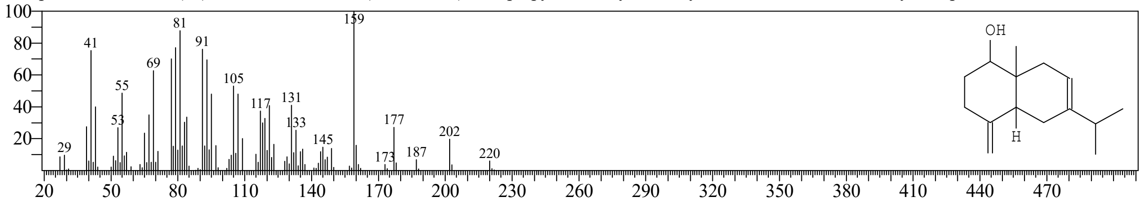

Hit#:4 Entry:56895 Library:NIST17-1.lib
SI:89 Formula:C12H16ClN CAS:0-00-0 MolWeight:209 RetIndex:1647
CompName:Benzylamine, N-(3-chloro-2,2-dimethyl-1-propylidene)- $$ N-[(E)-3-Chloro-2,2-dimethylpropylidene](phenyl)methanamine # $$

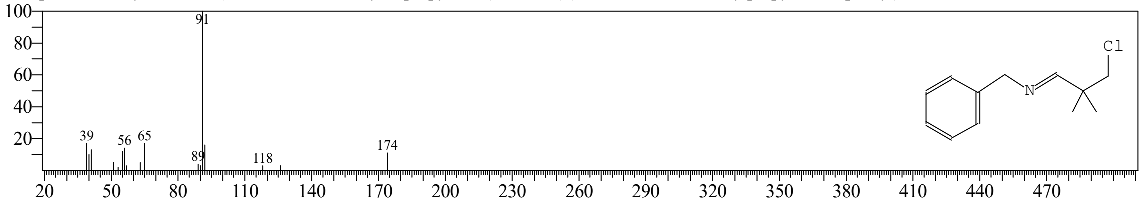

Hit#:5 Entry:43322 Library:NIST17-1.lib
SI:88 Formula:C13H20O CAS:63922-50-9 MolWeight:192 RetIndex:0
CompName:3,4-Heptadien-2-one, 3-cyclopentyl-6-methyl- $$ 3-Cyclopentyl-6-methyl-3,4-heptadien-2-one # $$

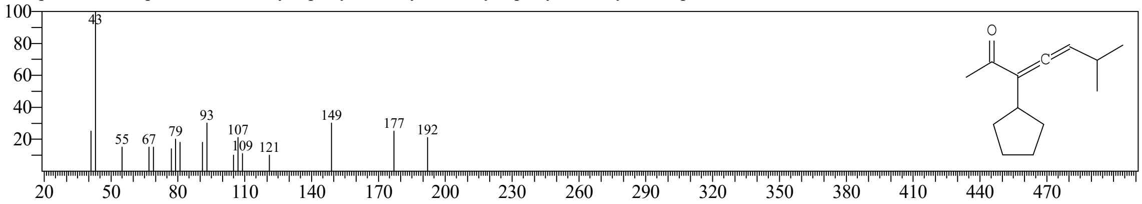

Qualitative Analysis Report

<< Target >>
Line#:51 R.Time:39.542(Scan#:8531) Retention Index:1642 MassPeaks:249
RawMode:Averaged 39.538-39.546(8530-8532) BasePeak:95.10(5389)
BG Mode:Calc. from Peak Group 1 - Event 1 Scan

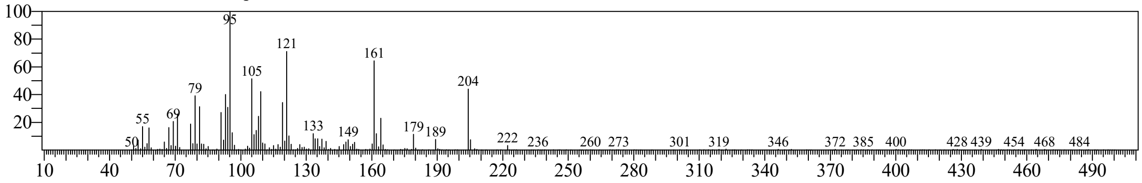

Hit#:1 Entry:2054 Library:FFNSC 3.lib
SI:97 Formula:C15 H26 O CAS:19912-62-0 MolWeight:222 RetIndex:1645
CompName:T-Muurolol

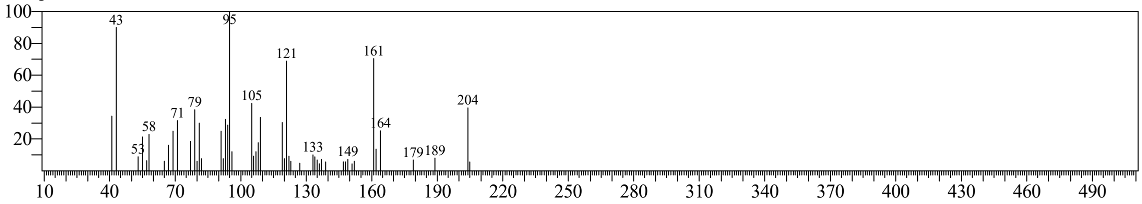

Hit#:2 Entry:5613 Library:NIST17-1.lib
SI:89 Formula:BrN3 CAS:13973-87-0 MolWeight:121 RetIndex:0
CompName:Bromine azide $$ Bromine azide (Br(N3)) $$ Bromine nitride $$ Nitrogen bromide (N3Br) $$

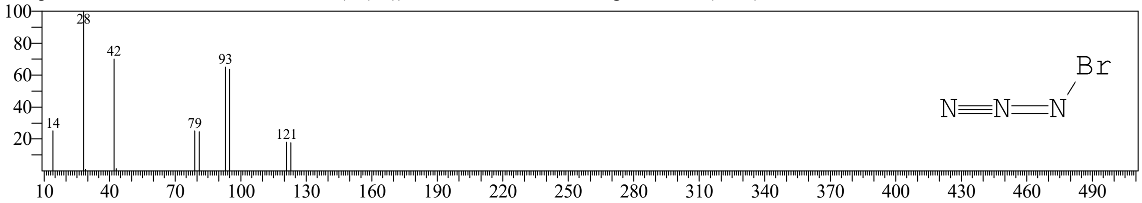

Hit#:3 Entry:1102 Library:FFNSC 3.lib
SI:87 Formula:C15 H26 O CAS:81967-90-0 MolWeight:222 RetIndex:1641
CompName:Naphth-1-ol <1,2,3,4,4a,7,8,8a-octahydro-, 4-isopropyl-, 1,6-dimethyl->

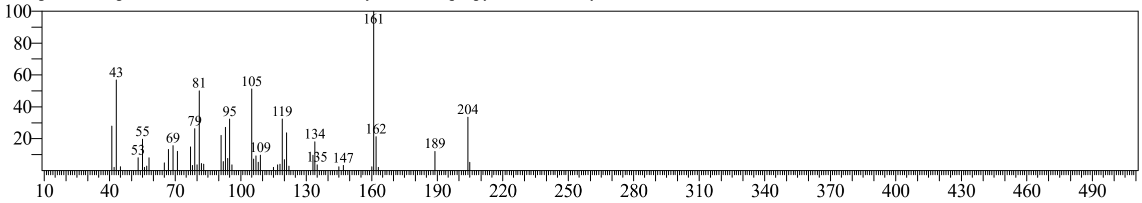

Hit#:4 Entry:2655 Library:FFNSC 3.lib
SI:87 Formula:C15 H26 O CAS:1891-45-8 MolWeight:222 RetIndex:1650
CompName:Himachalol

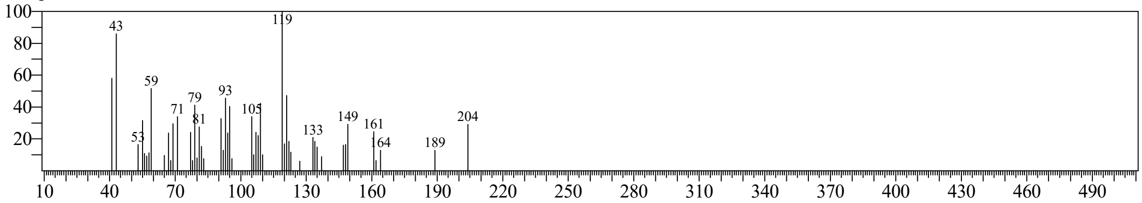

Hit#:5 Entry:1187 Library:FFNSC 3.lib
SI:86 Formula:C15 H26 O CAS:19435-97-3 MolWeight:222 RetIndex:1651
CompName:Muurolol <alpha->

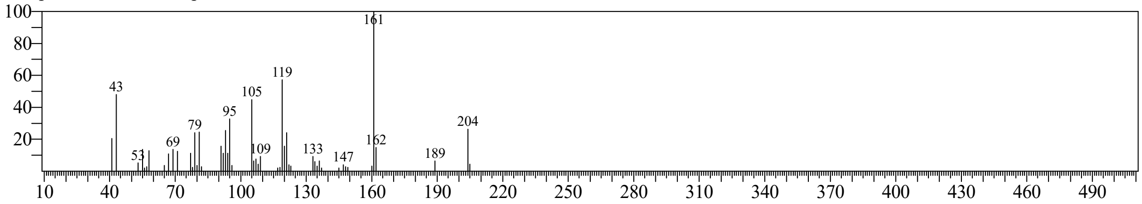

Qualitative Analysis Report

<< Target >>
Line#:52   R.Time:39.638(Scan#:8554) Retention Index:1645 MassPeaks:245
RawMode:Averaged 39.633-39.642(8553-8555) BasePeak:161.15(1217)
BG Mode:Calc. from Peak Group 1 - Event 1 Scan

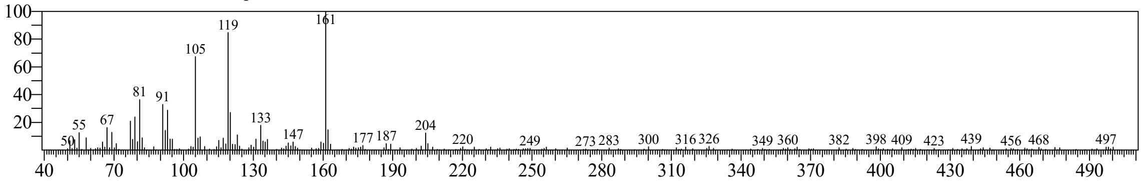

Hit#:1 Entry:1187 Library:FFNSC 3.lib
SI:89 Formula:C15 H26 O CAS:19435-97-3 MolWeight:222 RetIndex:1651
CompName:Muurolol <alpha->

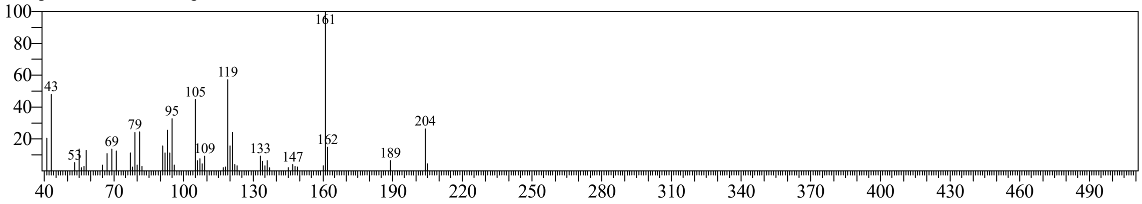

Hit#:2 Entry:41955 Library:NIST17-1.lib
SI:89 Formula:C14H22 CAS:61227-89-2 MolWeight:190 RetIndex:0
CompName:5,6-Decadien-3-yne, 5,7-diethyl- $$ 5,7-Diethyl-5,6-decadien-3-yne # $$

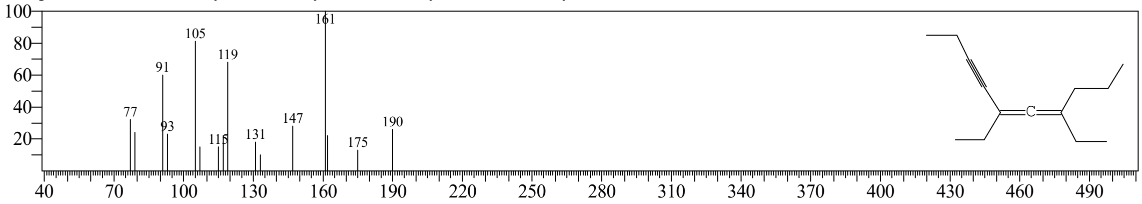

Hit#:3 Entry:1102 Library:FFNSC 3.lib
SI:88 Formula:C15 H26 O CAS:81967-90-0 MolWeight:222 RetIndex:1641
CompName:Naphth-1-ol <1,2,3,4,4a,7,8,8a-octahydro-, 4-isopropyl-, 1,6-dimethyl->

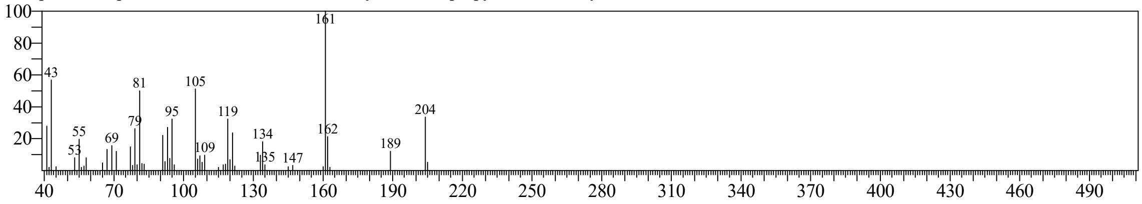

Hit#:4 Entry:2423 Library:FFNSC 3.lib
SI:85 Formula:C15 H26 O CAS:5937-11-1 MolWeight:222 RetIndex:1640
CompName:Cadinol <epi-alpha->

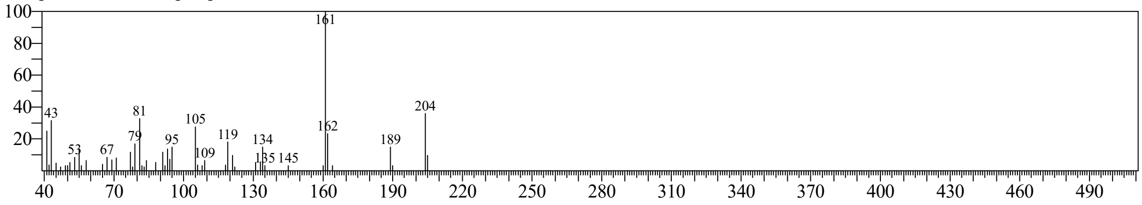

Hit#:5 Entry:2578 Library:FFNSC 3.lib
SI:84 Formula:C15 H26 O CAS:217650-27-6 MolWeight:222 RetIndex:1638
CompName:Cadin-4en-7-ol <cis->

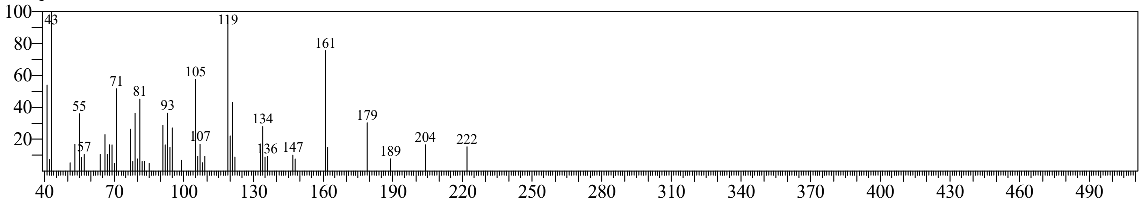

Qualitative Analysis Report

<< Target >>
Line#:53 R.Time:39.962(Scan#:8632) Retention Index:1654 MassPeaks:316
RawMode:Averaged 39.958-39.967(8631-8633) BasePeak:95.10(10353)
BG Mode:Calc. from Peak Group 1 - Event 1 Scan

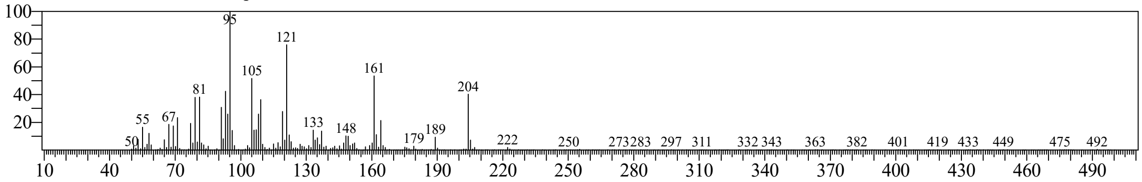

Hit#:1 Entry:1496 Library:FFNSC 3.lib
SI:97 Formula:C15 H26 O CAS:481-34-5 MolWeight:222 RetIndex:1659
CompName:Cadin-4-en-10-ol

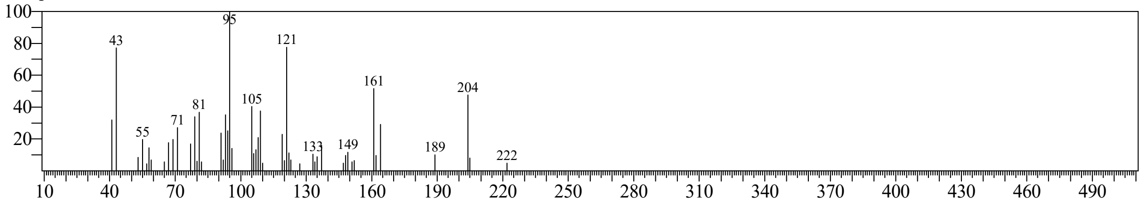

Hit#:2 Entry:2054 Library:FFNSC 3.lib
SI:96 Formula:C15 H26 O CAS:19912-62-0 MolWeight:222 RetIndex:1645
CompName:T-Muurolol

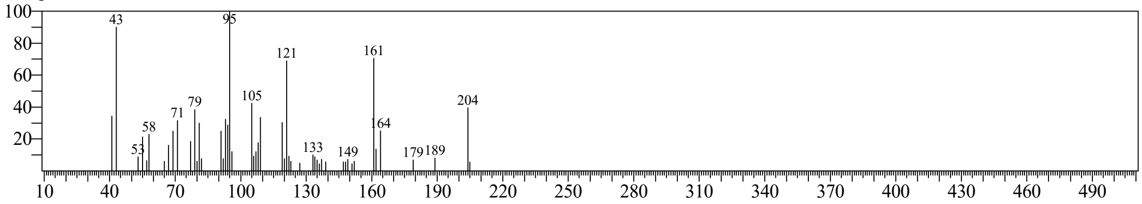

Hit#:3 Entry:5613 Library:NIST17-1.lib
SI:90 Formula:BrN3 CAS:13973-87-0 MolWeight:121 RetIndex:0
CompName:Bromine azide $$ Bromine azide (Br(N3)) $$ Bromine nitride $$ Nitrogen bromide (N3Br) $$

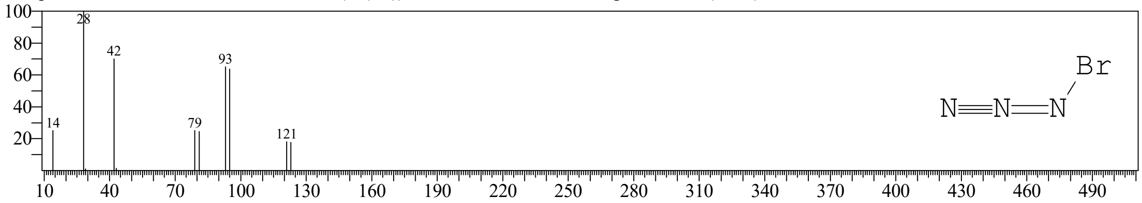

Hit#:4 Entry:2655 Library:FFNSC 3.lib
SI:87 Formula:C15 H26 O CAS:1891-45-8 MolWeight:222 RetIndex:1650
CompName:Himachalol

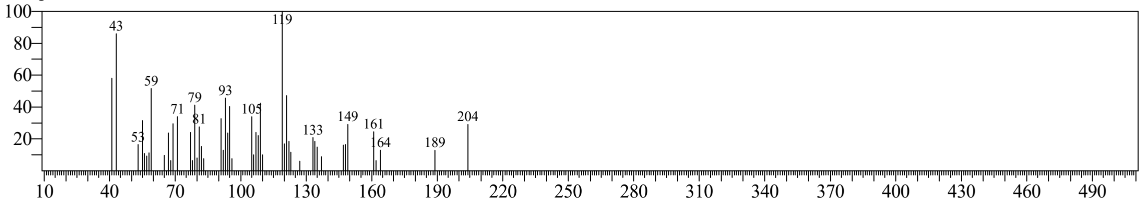

Hit#:5 Entry:67897 Library:NIST17-1.lib
SI:86 Formula:C11H7ClO3 CAS:0-00-0 MolWeight:222 RetIndex:1663
CompName:2-Furoic acid, 4-chlorophenyl ester

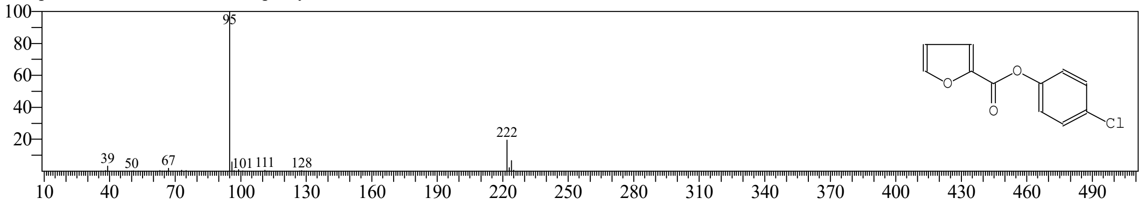

Qualitative Analysis Report

<< Target >>
Line#:54 R.Time:40.217(Scan#:8693) Retention Index:1660 MassPeaks:269
RawMode:Averaged 40.212-40.221(8692-8694) BasePeak:67.05(2690)
BG Mode:Calc. from Peak Group 1 - Event 1 Scan

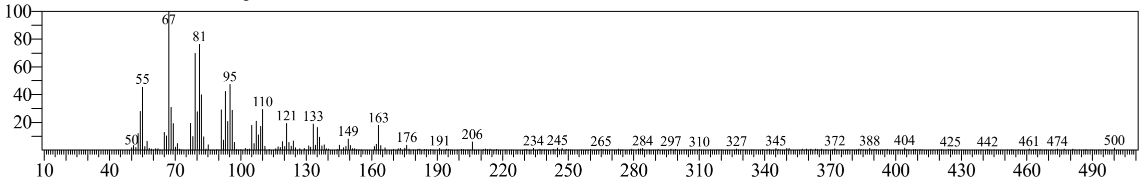

Hit#:1 Entry:192 Library:NIST17-1.lib
SI:93 Formula:CH3F2N CAS:753-58-2 MolWeight:67 RetIndex:0
CompName:Methanamine, N,N-difluoro- $$ Methylamine, N,N-difluoro- $$ (Difluoroamino)methane $$ N,N-Difluoromethylamine $$ Methyldifluoroamin

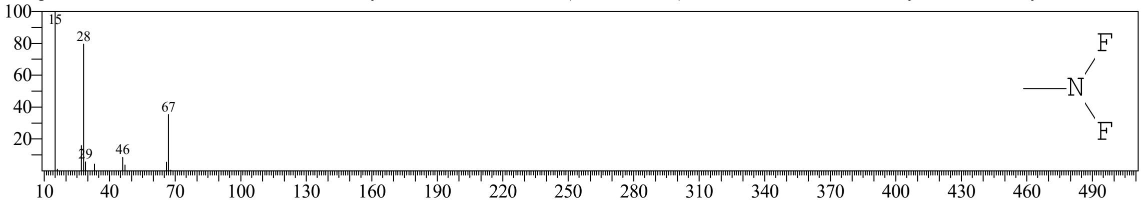

Hit#:2 Entry:24390 Library:NIST17-1.lib
SI:93 Formula:C12H20 CAS:1129-90-4 MolWeight:164 RetIndex:0
CompName:Cyclododecyne $$ 1-Cyclododecyne # $$

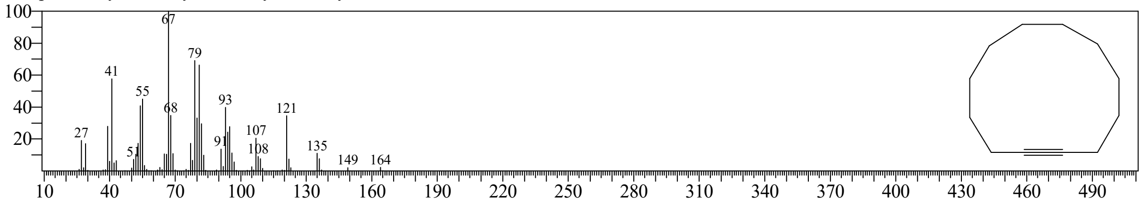

Hit#:3 Entry:198 Library:NIST17-1.lib
SI:92 Formula:ClO2 CAS:10049-04-4 MolWeight:67 RetIndex:0
CompName:Chlorine dioxide $$ Chlorine oxide (ClO2) $$ Chlorine(IV) oxide $$ Chloryl radical $$ Doxcide 50 $$ Chlorine oxide $$ ClO2 $$ Alcide $$ A

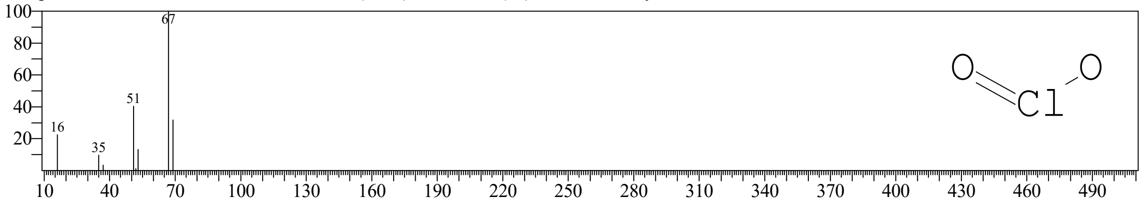

Hit#:4 Entry:137648 Library:NIST17-1.lib
SI:89 Formula:C19H34O2 CAS:0-00-0 MolWeight:294 RetIndex:0
CompName:Methyl 7,8-octadecadienoate

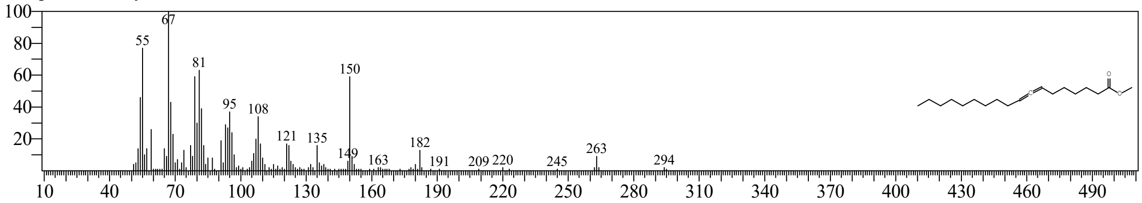

Hit#:5 Entry:137649 Library:NIST17-1.lib
SI:88 Formula:C19H34O2 CAS:0-00-0 MolWeight:294 RetIndex:0
CompName:Methyl 8,9-octadecadienoate

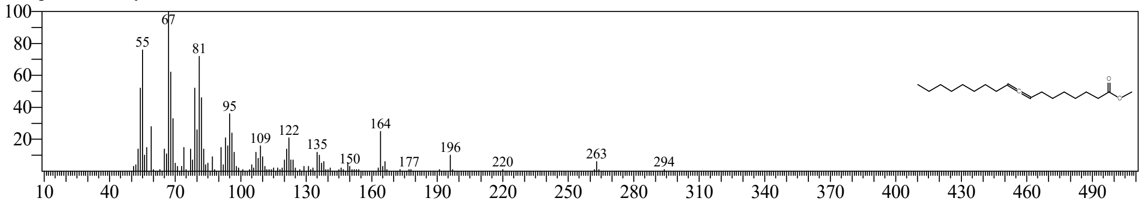

Qualitative Analysis Report

<< Target >>
Line#:55 R.Time:40.483(Scan#:8757) Retention Index:1667 MassPeaks:273
RawMode:Averaged 40.479-40.487(8756-8758) BasePeak:81.10(866)
BG Mode:Calc. from Peak Group 1 - Event 1 Scan

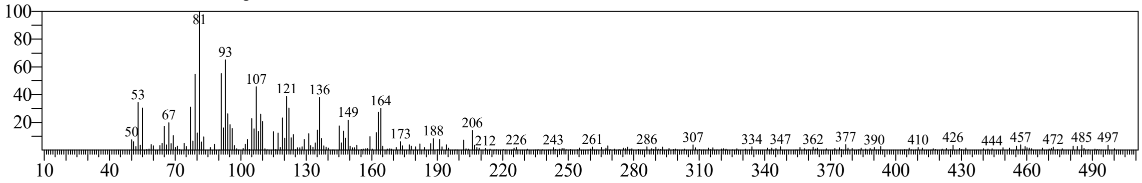

Hit#:1 Entry:3768 Library:NIST17-1.lib
SI:90 Formula:C7H12O CAS:4058-52-0 MolWeight:112 RetIndex:0
CompName:3,4-Pentadien-1-ol, 2,2-dimethyl- $$ 2,2-Dimethyl-3,4-pentadien-1-ol # $$

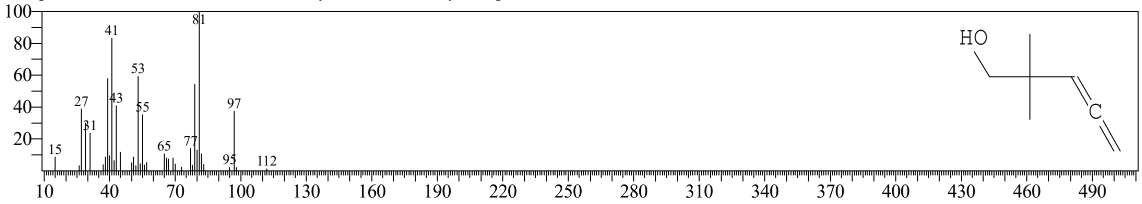

Hit#:2 Entry:158522 Library:NIST17-1.lib
SI:87 Formula:C11H5N7O5 CAS:0-00-0 MolWeight:315 RetIndex:0
CompName:12-(Furan-2-ylmethyl)-4,9,15-trioxa-3,5,8,10,12,14,16-heptaazatetracyclo[11.3.0.0(2,6).0(7,11)]hexadeca-1(16),2,5,7,10,13-hexaen-3-one

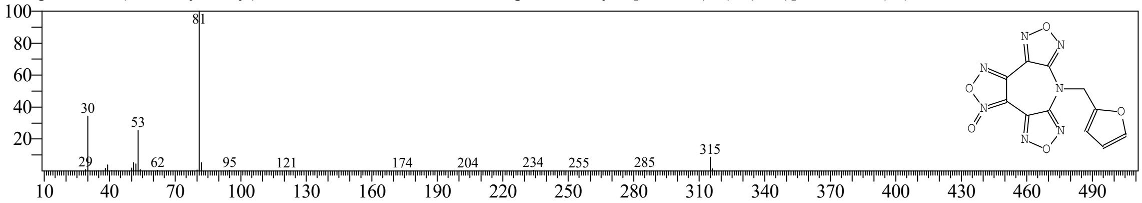

Hit#:3 Entry:134877 Library:NIST17-1.lib
SI:86 Formula:C13H16N4O2S CAS:3400-05-3 MolWeight:292 RetIndex:0
CompName:1-((4-Methylphenyl)sulfonylamino)-4,5,6,7(1H)-tetrahydrobenzo(d)triazole $$ 4-Methyl-N-(4,5,6,7-tetrahydro-1H-1,2,3-benzotriazol-1-yl)benz

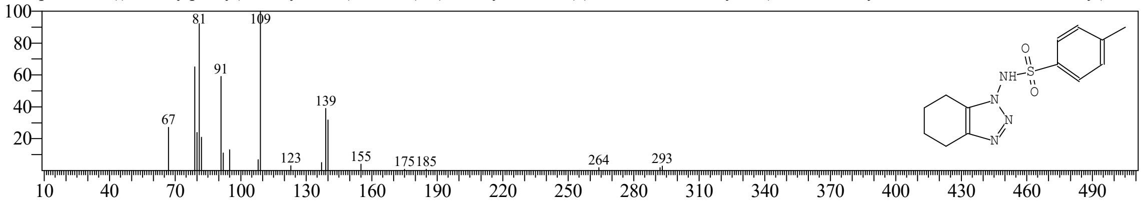

Hit#:4 Entry:38638 Library:NIST17-1.lib
SI:86 Formula:C9H14O2S CAS:0-00-0 MolWeight:186 RetIndex:0
CompName:3,4-Dimethyl-2-prop-2-enyl-2,5-dihydrothiophene 1,1-dioxide

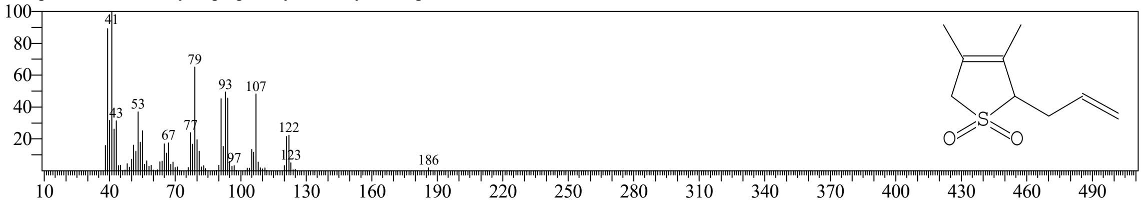

Hit#:5 Entry:3239 Library:NIST17-1.lib
SI:85 Formula:C7H10O CAS:4058-51-9 MolWeight:110 RetIndex:0
CompName:3,4-Pentadienal, 2,2-dimethyl- $$ 2,2-Dimethyl-3,4-pentadienal # $$

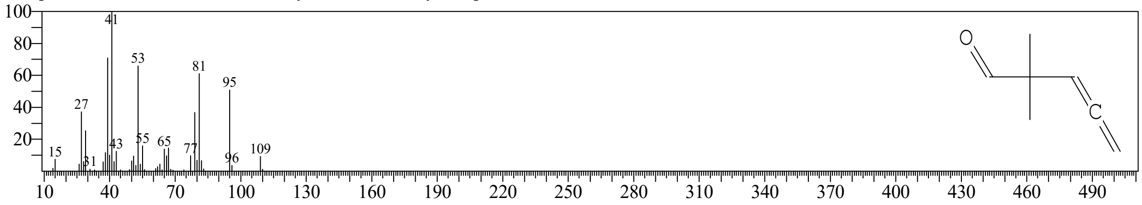

Qualitative Analysis Report

<< Target >>
Line#:56 R.Time:41.075(Scan#:8899) Retention Index:1683 MassPeaks:286
RawMode:Averaged 41.071-41.079(8898-8900) BasePeak:119.10(7795)
BG Mode:Calc. from Peak Group 1 - Event 1 Scan

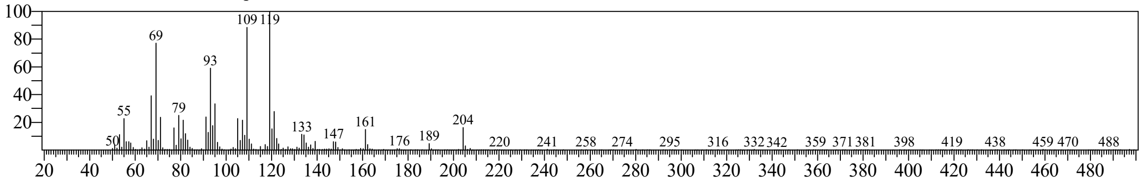

Hit#:1 Entry:2734 Library:FFNSC 3.lib
SI:95 Formula:C15 H26 O CAS:23178-88-3 MolWeight:222 RetIndex:1679
CompName:Bisabolol<epi-alpha->

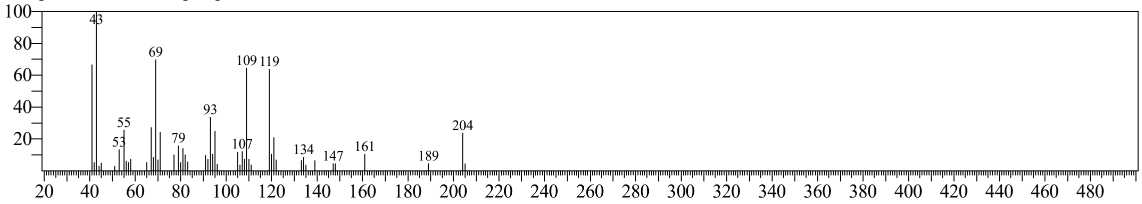

Hit#:2 Entry:65886 Library:NIST17-1.lib
SI:92 Formula:C10H20OS2 CAS:105705-07-5 MolWeight:220 RetIndex:1674
CompName:2-(1S,2S-Dimethyl-4-hydroxybutyl)-1,3-dithiane $$ 4-(1,3-Dithian-2-yl)-3-methyl-1-pentanol # $$

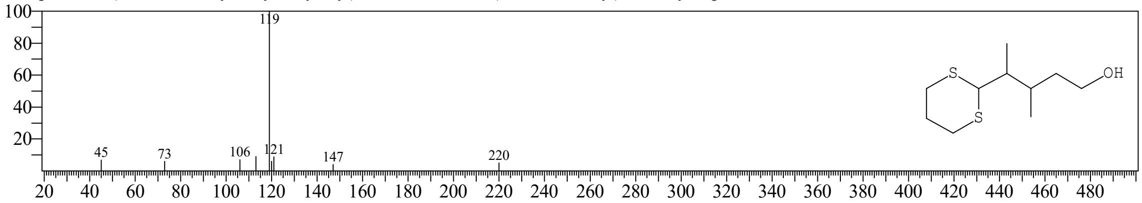

Hit#:3 Entry:1008 Library:FFNSC 3.lib
SI:90 Formula:C15 H26 O CAS:515-69-5 MolWeight:222 RetIndex:1688
CompName:Bisabolol <alpha->

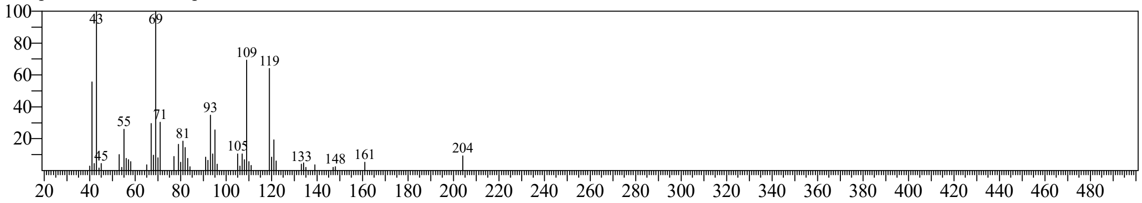

Hit#:4 Entry:111966 Library:NIST17-1.lib
SI:86 Formula:C13H19NO3S CAS:70291-66-6 MolWeight:269 RetIndex:0
CompName:4,8-Dimethyl-6-(p-tolyl)-5,6,7,8-tetrahydro-4H-1,3,2,6-dioxathiazocine 2-oxide $$ 4,8-Dimethyl-6-(4-methylphenyl)-1,3,2,6-dioxathiazocane 2-

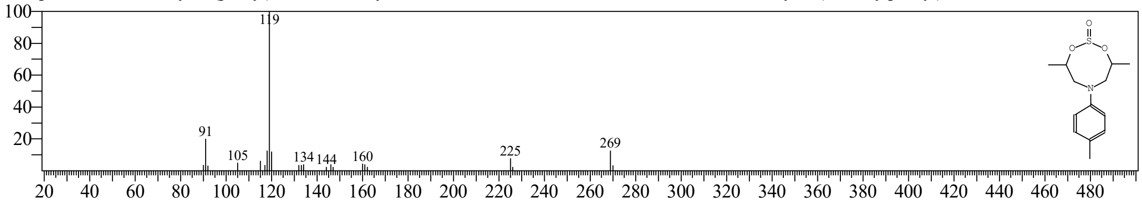

Hit#:5 Entry:68722 Library:NIST17-1.lib
SI:84 Formula:C15H26O CAS:19435-77-9 MolWeight:222 RetIndex:1689
CompName:(4aR,5R,9aR)-1,1,4a,8-Tetramethyl-2,3,4,4a,5,6,7,9a-octahydro-1H-benzo[7]annulen-5-ol $$ 1H-Benzocyclohepten-5-ol, 2,3,4,4a,5,6,7,9a-octa

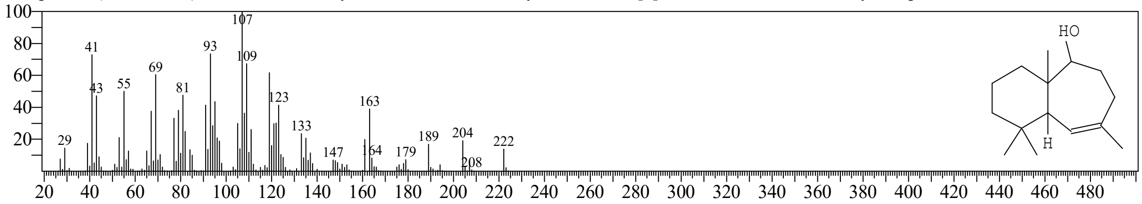

Qualitative Analysis Report

<< Target >>
Line#:57 R.Time:42.629(Scan#:9272) Retention Index:1725 MassPeaks:240
RawMode:Averaged 42.625-42.633(9271-9273) BasePeak:69.10(2179)
BG Mode:Calc. from Peak Group 1 - Event 1 Scan

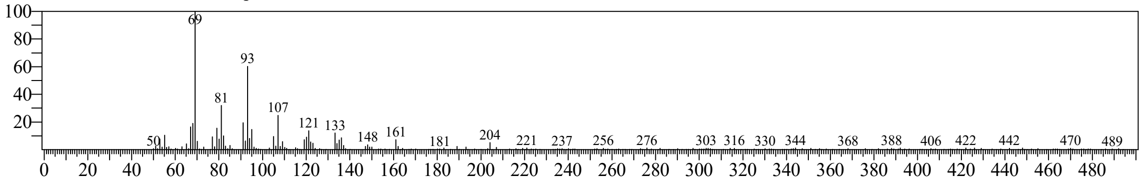

Hit#:1 Entry:228 Library:NIST17-1.lib
SI:99 Formula:C2H3N3 CAS:288-36-8 MolWeight:69 RetIndex:0
CompName:1H-1,2,3-Triazole $$ V-triazole $$ Osotriazole $$ Pyrrodiazole $$ Triazacyclopentadiene $$ 1,2,3-Triazole $$

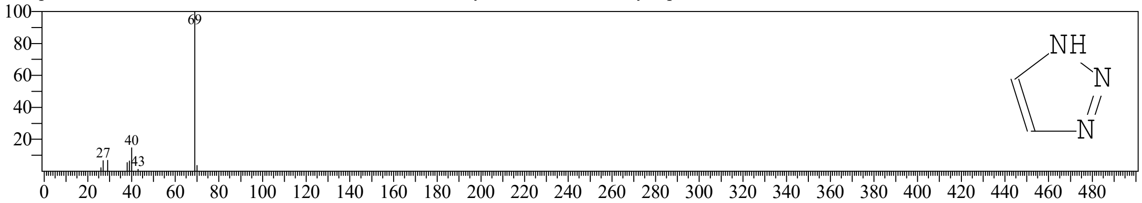

Hit#:2 Entry:981 Library:NIST17-1.lib
SI:96 Formula:CF4 CAS:75-73-0 MolWeight:88 RetIndex:0
CompName:Tetrafluoromethane $$ Carbon tetrafluoride $$ Methane, tetrafluoro- $$ Arcton 0 $$ Carbon fluoride (CF4) $$ Freon 14 $$ FC 14 $$ Perfluoro

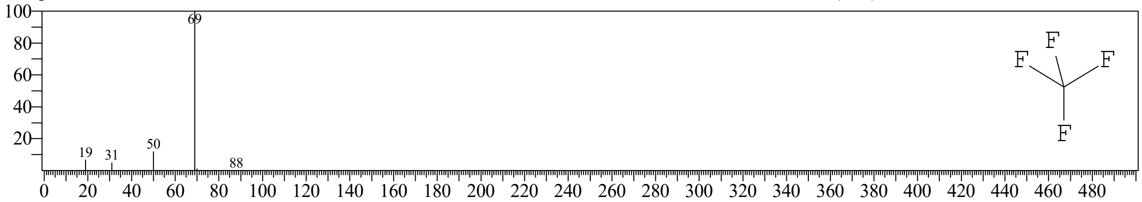

Hit#:3 Entry:10642 Library:NIST17-1.lib
SI:94 Formula:CF5P CAS:1112-04-5 MolWeight:138 RetIndex:0
CompName:Trifluoromethyldifluorophosphine $$ Trifluoromethylphosphonous difluoride # $$

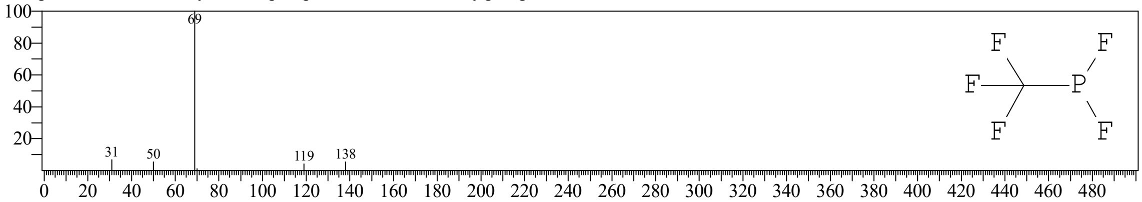

Hit#:4 Entry:10455 Library:NIST17-1.lib
SI:94 Formula:CClF4N CAS:13880-72-3 MolWeight:137 RetIndex:0
CompName:N-Chloro-N-fluoro-trifluoromethylamine $$ N-Chloro-N-tetrafluoromethanamine # $$

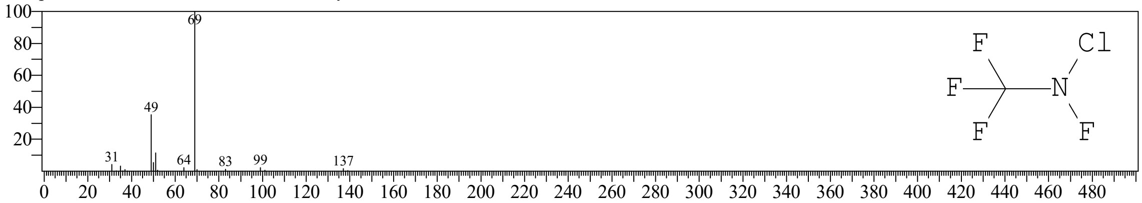

Hit#:5 Entry:7138 Library:NIST17-1.lib
SI:93 Formula:C5H5NOS CAS:60034-28-8 MolWeight:127 RetIndex:0
CompName:Crotonyl isothiocyanate $$ (2E)-2-Butenoyl isothiocyanate # $$

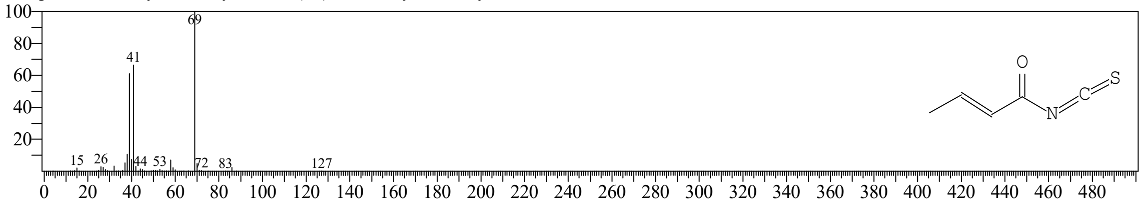

Qualitative Analysis Report

<< Target >>
Line#:58 R.Time:43.821(Scan#:9558) Retention Index:1758 MassPeaks:312
RawMode:Averaged 43.817-43.825(9557-9559) BasePeak:99.00(334)
BG Mode:Calc. from Peak Group 1 - Event 1 Scan

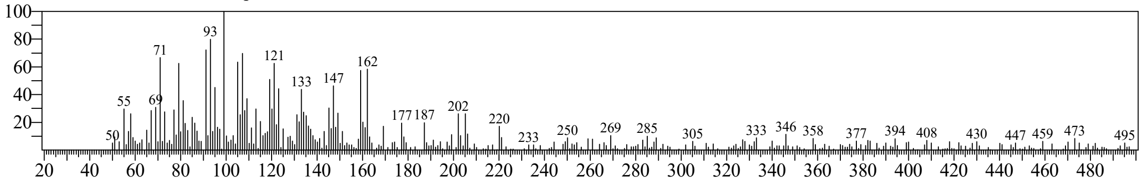

Hit#:1 Entry:47538 Library:NIST17-1.lib
SI:90 Formula:$C_8H_{24}B_2N_4$ CAS:1630-79-1 MolWeight:198 RetIndex:0
CompName:Diborane(4)tetramine, octamethyl- $$ Diborane(4), tetrakis(dimethylamino)- $$ Dimethylamine, N,N',N'',N'''-diborane(4)diylidenetetrakis- $$ T

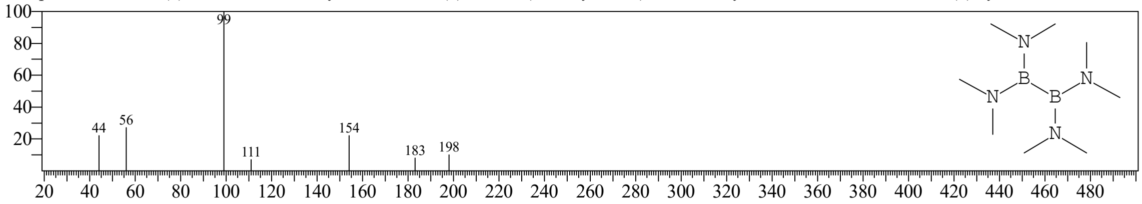

Hit#:2 Entry:6589 Library:NIST17-1.lib
SI:89 Formula:$C_2H_7O_4P$ CAS:1623-14-9 MolWeight:126 RetIndex:0
CompName:Phosphoric acid, monoethyl ester $$ Monoethyl phosphate $$ Ethyl phosphate ((eto)(ho)2po) $$ Ethyl dihydrogen phosphate $$ Monoethyl aci

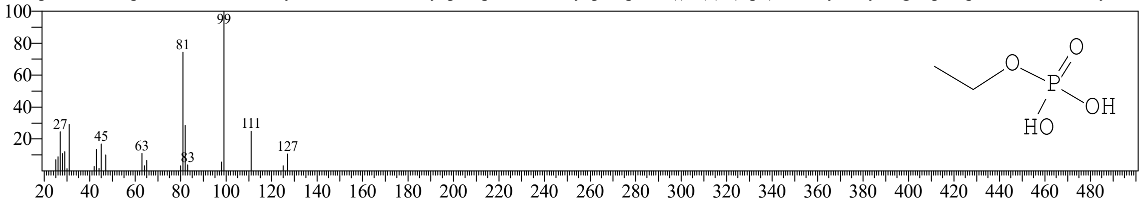

Hit#:3 Entry:1831 Library:NIST17-1.lib
SI:89 Formula:$C_2H_5N_5$ CAS:6154-04-7 MolWeight:99 RetIndex:0
CompName:5-Amino-2-methyl-2H-tetrazole $$ 2-Methyl-5-aminotetrazole $$ 2H-Tetrazol-5-amine, 2-methyl- $$ 2H-Tetrazole, 5-amino-2-methyl- $$ 5-An

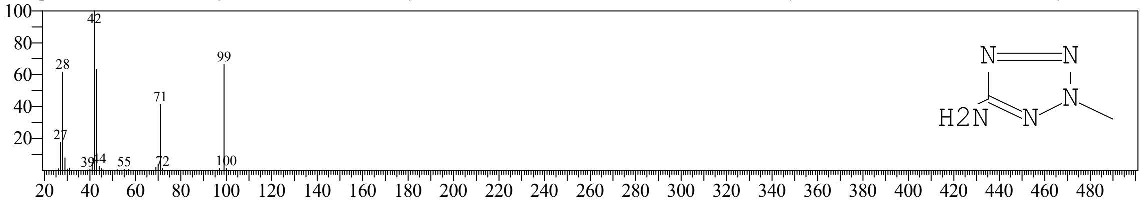

Hit#:4 Entry:153933 Library:NIST17-1.lib
SI:87 Formula:$C_{19}H_{39}BO_2$ CAS:66237-68-1 MolWeight:310 RetIndex:0
CompName:Octadecane-1,3-diol methaneboronate $$ 2-Methyl-4-pentadecyl-1,3,2-dioxaborinane # $$

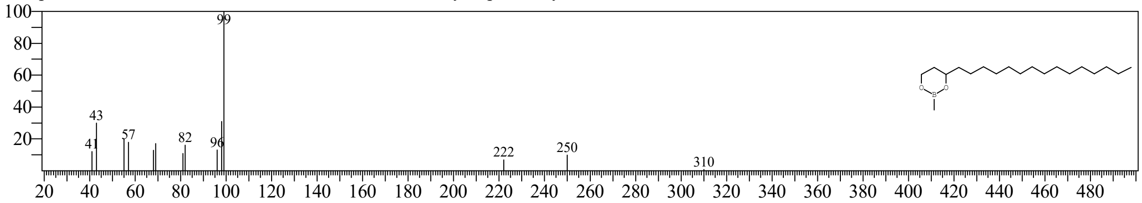

Hit#:5 Entry:18140 Library:NIST17-1.lib
SI:86 Formula:$C_5H_{12}FO_2P$ CAS:13273-12-6 MolWeight:154 RetIndex:0
CompName:2-Methyl-2-propyl methylphosphonofluoridate $$ tert-Butyl methylphosphonofluoridoate # $$

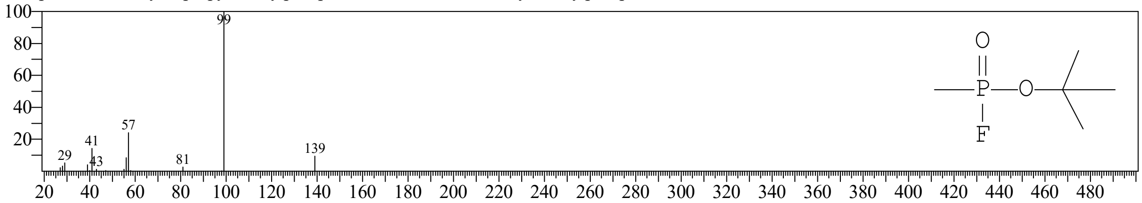

Qualitative Analysis Report

<< Target >>
Line#:59 R.Time:46.258(Scan#:10143) Retention Index:1828 MassPeaks:311
RawMode:Averaged 46.254-46.263(10142-10144) BasePeak:91.10(523)
BG Mode:Calc. from Peak Group 1 - Event 1 Scan

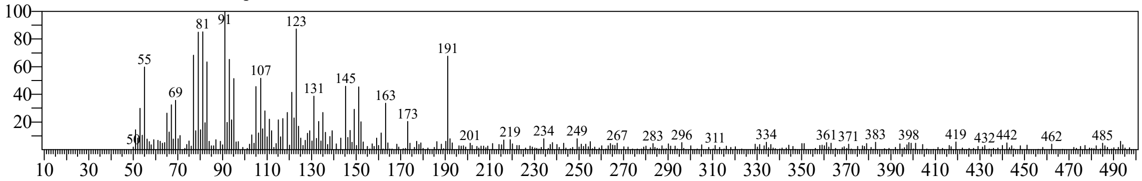

Hit#:1 Entry:31751 Library:NIST17-1.lib
SI:91 Formula:C9H8N2O2 CAS:16844-42-1 MolWeight:176 RetIndex:0
CompName:Sydnone, 3-(phenylmethyl)- $$ Sydnone, 3-benzyl- $$ Benzyl sydnone $$ 3-Benzylsydnone $$ 3-Benzyl-1,2,3-oxadiazol-3-ium-5-olate # $$

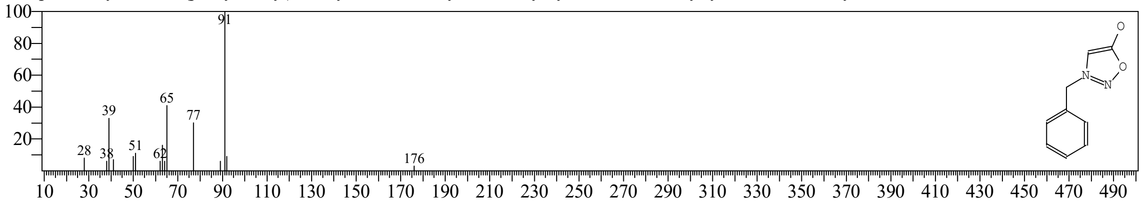

Hit#:2 Entry:23034 Library:NIST17-1.lib
SI:88 Formula:C10H14N2 CAS:24175-02-8 MolWeight:162 RetIndex:0
CompName:diazoadamantane $$ Aziadamantane $$

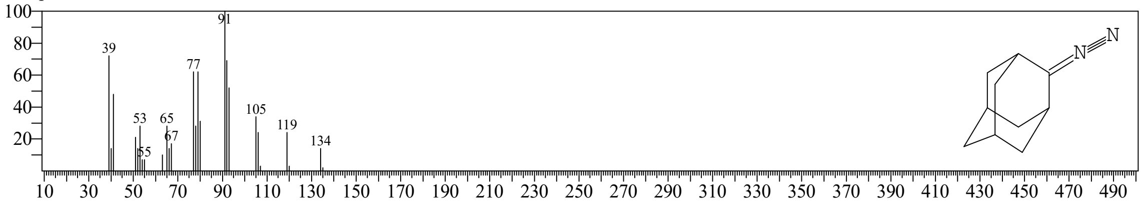

Hit#:3 Entry:79223 Library:NIST17-1.lib
SI:88 Formula:C15H22O2 CAS:5090-90-4 MolWeight:234 RetIndex:1833
CompName:(2R,3R,4aR,5S)-2-Hydroxy-4a,5-dimethyl-3-(prop-1-en-2-yl)-3,4,4a,5,6,7-hexahydronaphthalen-1(2H)-one $$ 1(2H)-Naphthalenone, 3,4,4a,5,

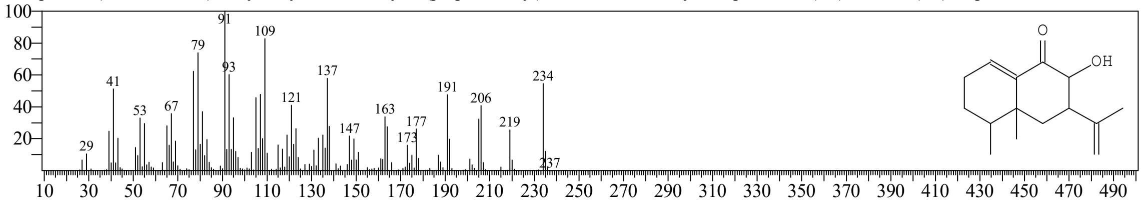

Hit#:4 Entry:59632 Library:NIST17-1.lib
SI:88 Formula:C13H12N2O CAS:0-00-0 MolWeight:212 RetIndex:0
CompName:1-Phenyl-2-(4-methylphenyl)-diazene 1-oxide $$ 2-(4-Methylphenyl)-1-phenyldiazene 1-oxide # $$

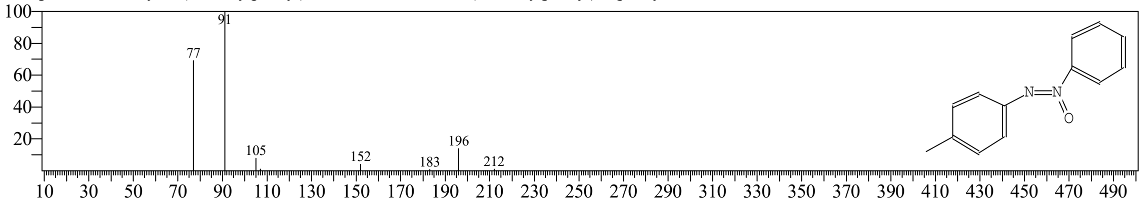

Hit#:5 Entry:51145 Library:NIST17-1.lib
SI:87 Formula:C15H22 CAS:4722-42-3 MolWeight:202 RetIndex:0
CompName:1,8-Cyclopentadecadiyne

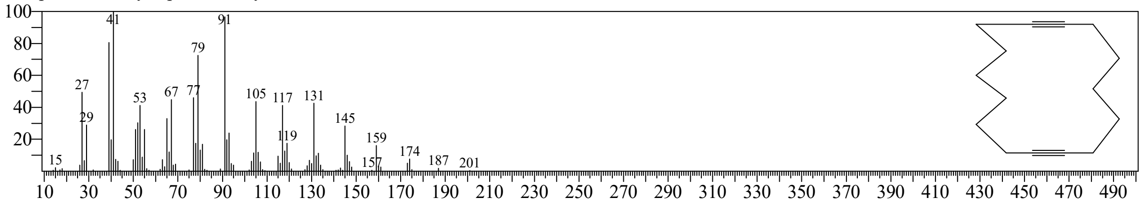

Qualitative Analysis Report

<< Target >>
Line#:60 R.Time:47.300(Scan#:10393) Retention Index:1858 MassPeaks:319
RawMode:Averaged 47.296-47.304(10392-10394) BasePeak:69.10(470)
BG Mode:Calc. from Peak Group 1 - Event 1 Scan

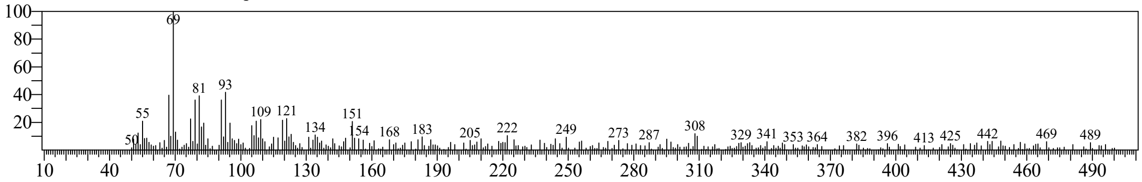

Hit#:1 Entry:228 Library:NIST17-1.lib
SI:98 Formula:C2H3N3 CAS:288-36-8 MolWeight:69 RetIndex:0
CompName:1H-1,2,3-Triazole $$ V-triazole $$ Osotriazole $$ Pyrrodiazole $$ Triazacyclopentadiene $$ 1,2,3-Triazole $$

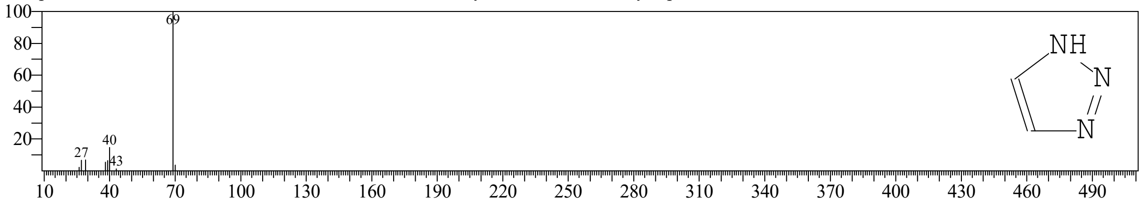

Hit#:2 Entry:981 Library:NIST17-1.lib
SI:94 Formula:CF4 CAS:75-73-0 MolWeight:88 RetIndex:0
CompName:Tetrafluoromethane $$ Carbon tetrafluoride $$ Methane, tetrafluoro- $$ Arcton 0 $$ Carbon fluoride (CF4) $$ Freon 14 $$ FC 14 $$ Perfluoro

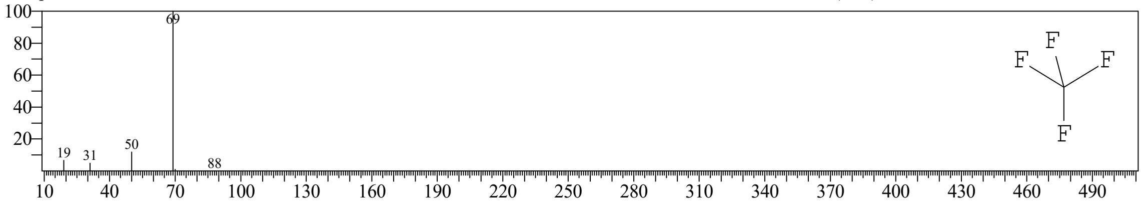

Hit#:3 Entry:9867 Library:NIST17-1.lib
SI:93 Formula:CF4OS CAS:812-12-4 MolWeight:136 RetIndex:0
CompName:Methanesulfinyl fluoride, trifluoro- $$ Trifluoromethanesulfinyl fluoride $$ Trifluoromethylsulfinyl fluoride $$

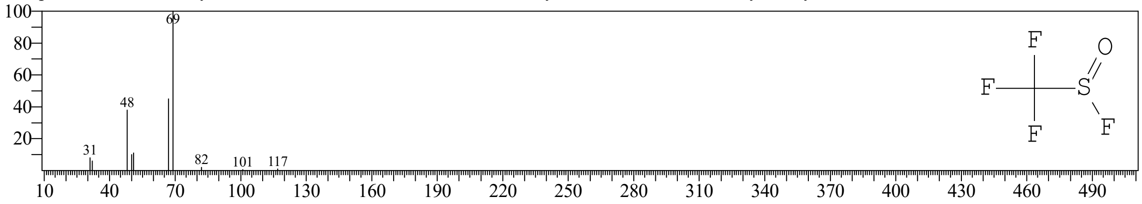

Hit#:4 Entry:13581 Library:NIST17-1.lib
SI:91 Formula:C6H12N2O2 CAS:108201-44-1 MolWeight:144 RetIndex:0
CompName:Diazene, 1-cyclopentyl-2-methoxy-, 1-oxide $$ 1-Cyclopentyl-2-methoxydiazene 1-oxide # $$

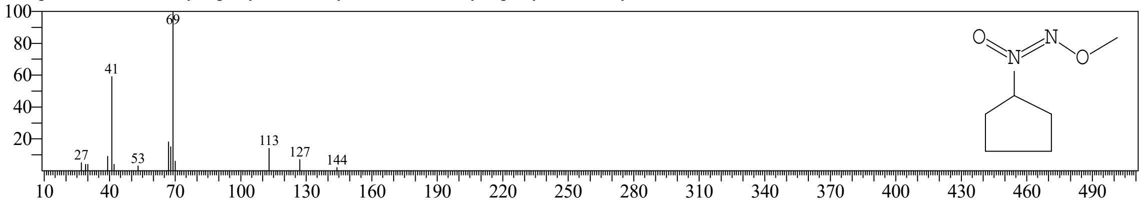

Hit#:5 Entry:73172 Library:NIST17-1.lib
SI:91 Formula:C2H6F4N2P2S CAS:27352-02-9 MolWeight:228 RetIndex:0
CompName:Phosphoramidothioic difluoride, [(dimethylamino)difluorophosphoranylidene]- $$ (Dimethylamino)(difluoro)phosphoranylidenephosphoramidc

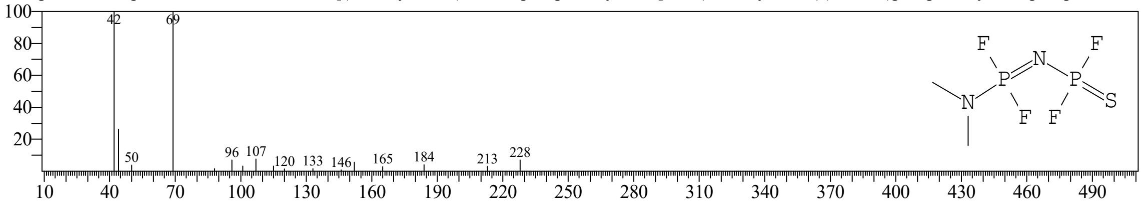

Qualitative Analysis Report

<< Target >>
Line#:61 R.Time:47.429(Scan#:10424) Retention Index:1862 MassPeaks:263
RawMode:Averaged 47.425-47.433(10423-10425) BasePeak:69.10(1149)
BG Mode:Calc. from Peak Group 1 - Event 1 Scan

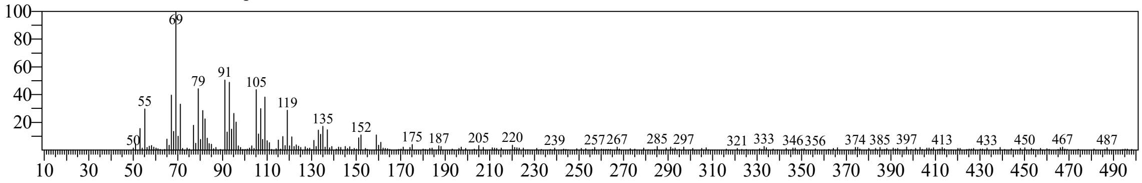

Hit#:1 Entry:228 Library:NIST17-1.lib
SI:99 Formula:C2H3N3 CAS:288-36-8 MolWeight:69 RetIndex:0
CompName:1H-1,2,3-Triazole $$ V-triazole $$ Osotriazole $$ Pyrrodiazole $$ Triazacyclopentadiene $$ 1,2,3-Triazole $$

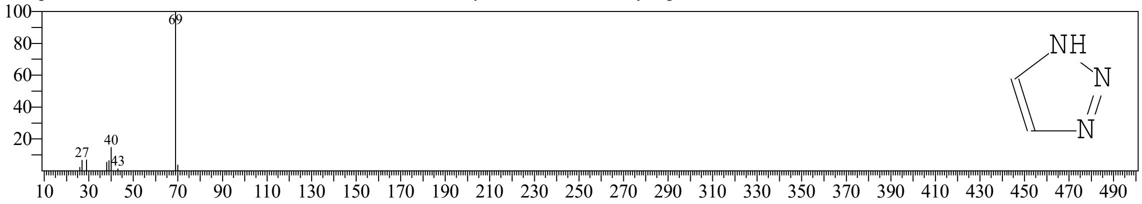

Hit#:2 Entry:981 Library:NIST17-1.lib
SI:95 Formula:CF4 CAS:75-73-0 MolWeight:88 RetIndex:0
CompName:Tetrafluoromethane $$ Carbon tetrafluoride $$ Methane, tetrafluoro- $$ Arcton 0 $$ Carbon fluoride (CF4) $$ Freon 14 $$ FC 14 $$ Perfluoro

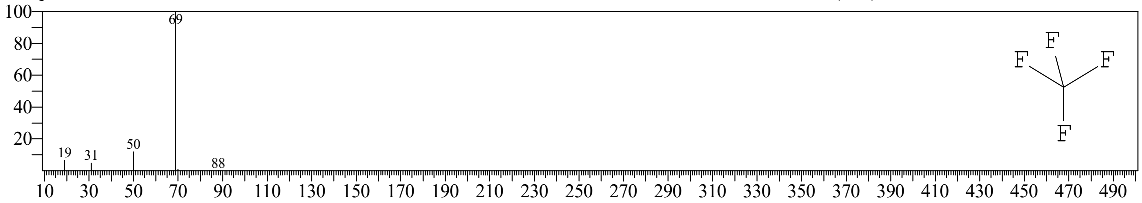

Hit#:3 Entry:9867 Library:NIST17-1.lib
SI:93 Formula:CF4OS CAS:812-12-4 MolWeight:136 RetIndex:0
CompName:Methanesulfinyl fluoride, trifluoro- $$ Trifluoromethanesulfinyl fluoride $$ Trifluoromethylsulfinyl fluoride $$

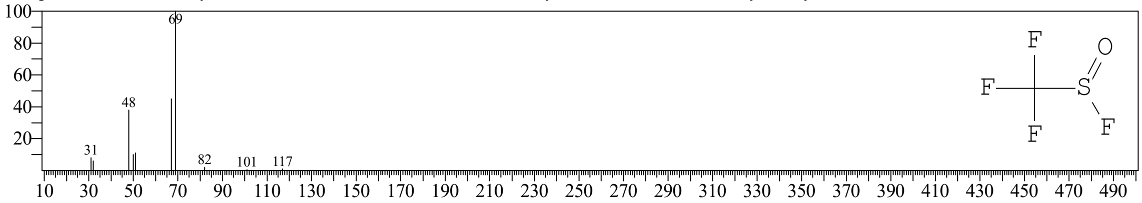

Hit#:4 Entry:10455 Library:NIST17-1.lib
SI:91 Formula:CClF4N CAS:13880-72-3 MolWeight:137 RetIndex:0
CompName:N-Chloro-N-fluoro-trifluoromethylamine $$ N-Chloro-N-tetrafluoromethanamine # $$

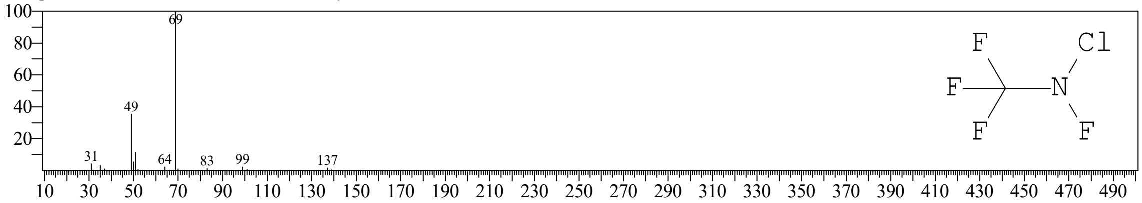

Hit#:5 Entry:10642 Library:NIST17-1.lib
SI:91 Formula:CF5P CAS:1112-04-5 MolWeight:138 RetIndex:0
CompName:Trifluoromethyldifluorophosphine $$ Trifluoromethylphosphonous difluoride # $$

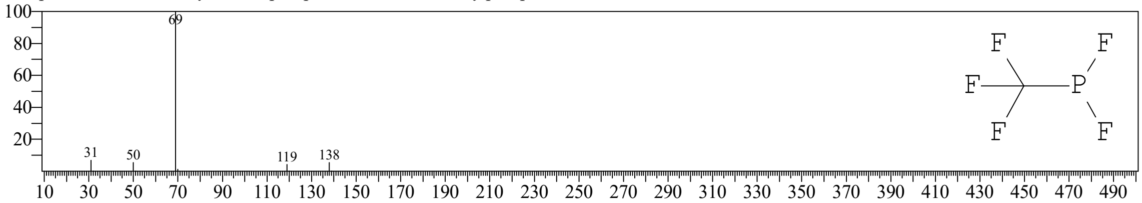

Qualitative Analysis Report

<< Target >>
Line#:62 R.Time:48.708(Scan#:10731) Retention Index:1899 MassPeaks:327
RawMode:Averaged 48.704-48.712(10730-10732) BasePeak:123.10(283)
BG Mode:Calc. from Peak Group 1 - Event 1 Scan

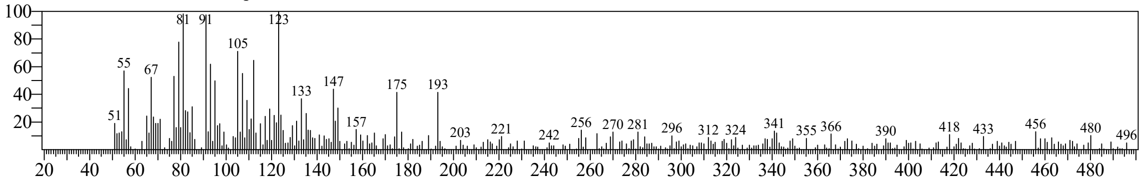

Hit#:1 Entry:37378 Library:NIST17-1.lib
SI:87 Formula:C10H16OS CAS:55849-07-5 MolWeight:184 RetIndex:0
CompName:1H-Thiepine, 2,3,6,7-tetrahydro-4,5-didehydro-3,3,6,6-tetramethyl-, 1-oxide $$ 3,3,6,6-Tetramethyl-4,5-didehydro-2,3,6,7-tetrahydrothiepine 1-

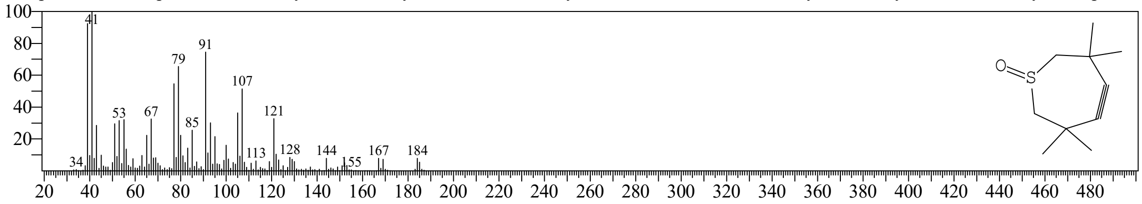

Hit#:2 Entry:90316 Library:NIST17-1.lib
SI:86 Formula:C15H18O3 CAS:130838-06-1 MolWeight:246 RetIndex:1892
CompName:Spirafolide $$ (3aS,5aS,10aS,10bS)-5a,10-dimethyl-3-methylene-3a,4,5,5a,10a,10b-hexahydrooxepino[4,5-g]benzofuran-2(3H)-one $$ Furo[2.

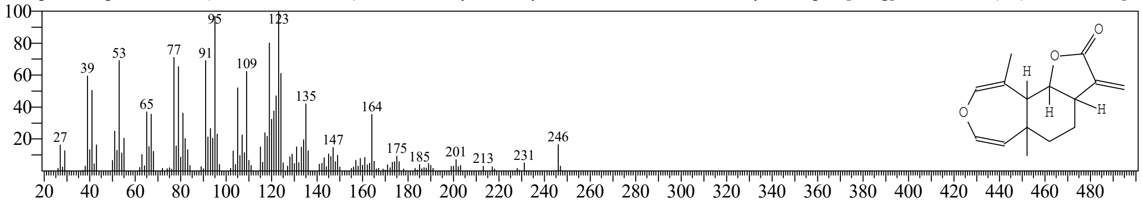

Hit#:3 Entry:38639 Library:NIST17-1.lib
SI:86 Formula:C9H14O2S CAS:0-00-0 MolWeight:186 RetIndex:0
CompName:5-But-2-enyl-4-methyl-2,3-dihydrothiophene 1,1-dioxide

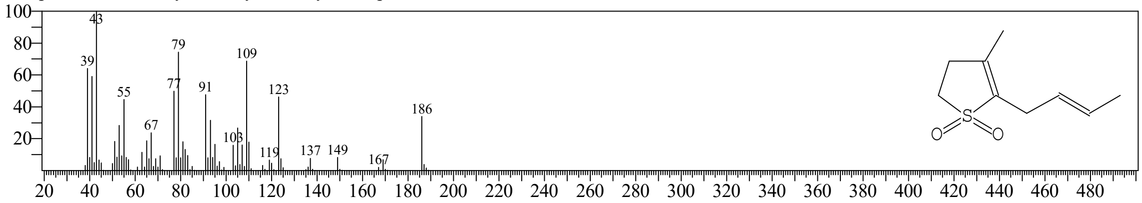

Hit#:4 Entry:81046 Library:NIST17-1.lib
SI:86 Formula:C15H24O2 CAS:0-00-0 MolWeight:236 RetIndex:1904
CompName:Bicyclo[4.4.0]dec-2-ene-4-ol, 2-methyl-9-(prop-1-en-3-ol-2-yl)- $$ 6-[1-(Hydroxymethyl)vinyl]-4,8a-dimethyl-1,2,4a,5,6,7,8,8a-octahydro-2-na

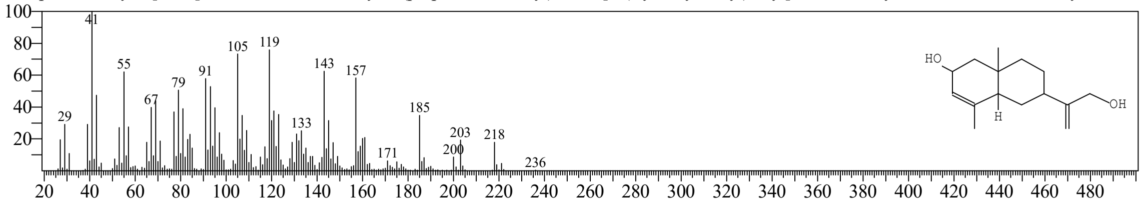

Hit#:5 Entry:221990 Library:NIST17-1.lib
SI:86 Formula:C22H35BO5 CAS:30888-49-4 MolWeight:390 RetIndex:0
CompName:Pregnan-11-one, 3,20-dihydroxy-17,21-[(methylborylene)bis(oxy)]-, (3.alpha.,5.beta.,20S)- $$ 5.beta.-Pregnan-11-one, 3.alpha.,17,20.alpha.,21-

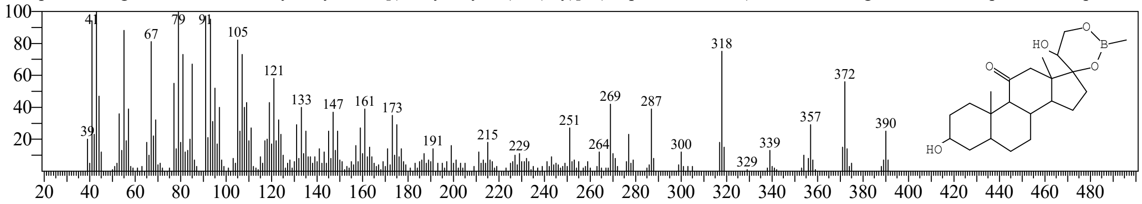

Qualitative Analysis Report

<< Target >>
Line#:63 R.Time:58.108(Scan#:12987) Retention Index:2200 MassPeaks:301
RawMode:Averaged 58.104-58.112(12986-12988) BasePeak:123.15(16951)
BG Mode:Calc. from Peak Group 1 - Event 1 Scan

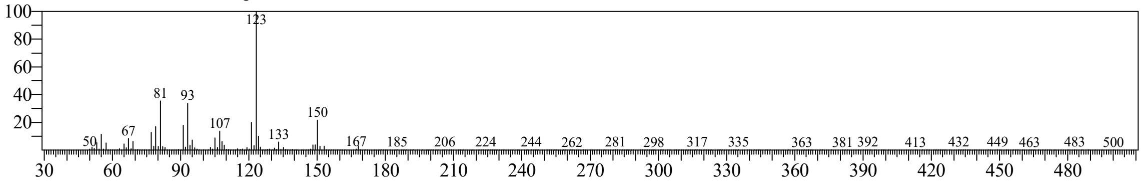

Hit#:1 Entry:26125 Library:NIST17-1.lib
SI:86 Formula:C3H9AsO3 CAS:23809-18-9 MolWeight:168 RetIndex:0
CompName:Arsonic acid, methyl-, dimethyl ester $$ Methanearsonic acid, dimethyl ester $$ Dimethyl methylarsonate $$

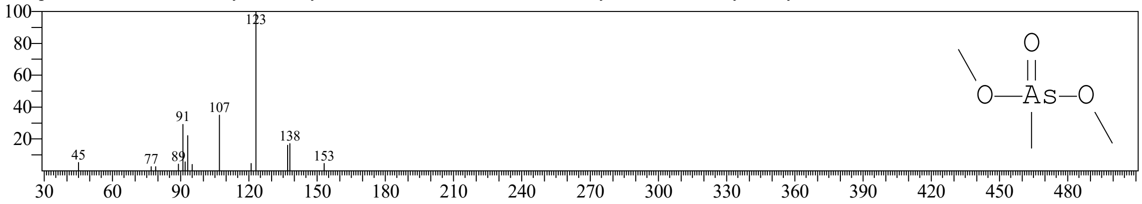

Hit#:2 Entry:93530 Library:NIST17-1.lib
SI:86 Formula:C12H14N2O2S CAS:53492-47-0 MolWeight:250 RetIndex:0
CompName:4-Methyl-3-[2-(4-methylphenylthio)ethyl]sydnone

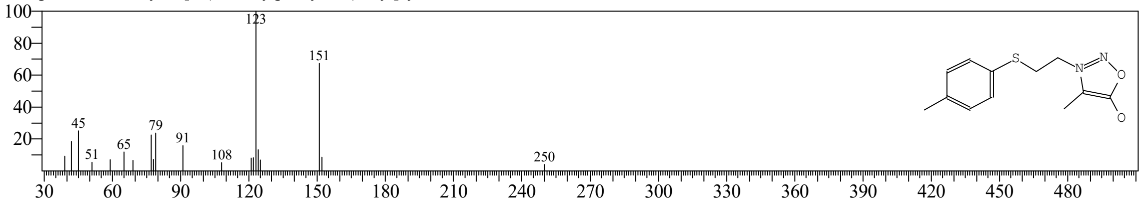

Hit#:3 Entry:79602 Library:NIST17-1.lib
SI:86 Formula:C10H10FN5O CAS:0-00-0 MolWeight:235 RetIndex:0
CompName:Benzamide, N-(2-ethyl-2H-1,2,3,4-tetrazol-5-yl)-4-fluoro-

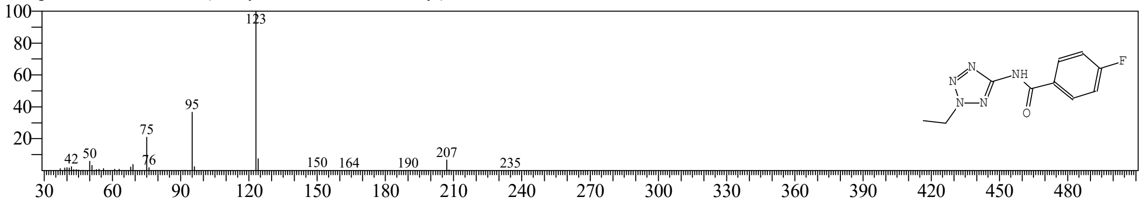

Hit#:4 Entry:36954 Library:NIST17-1.lib
SI:86 Formula:C3H9AsO4 CAS:13006-30-9 MolWeight:184 RetIndex:0
CompName:Trimethyl arsenate $$ Arsenic acid (H3Aso4), trimethyl ester $$ Trimethoxyarsine oxide $$

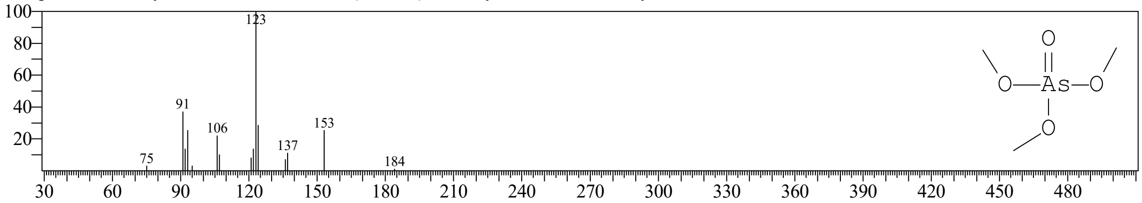

Hit#:5 Entry:80458 Library:NIST17-1.lib
SI:85 Formula:C11H12N2O2S CAS:96991-48-9 MolWeight:236 RetIndex:0
CompName:3-[2-(4-Methylphenylthio)ethyl]-4-H-sydnone

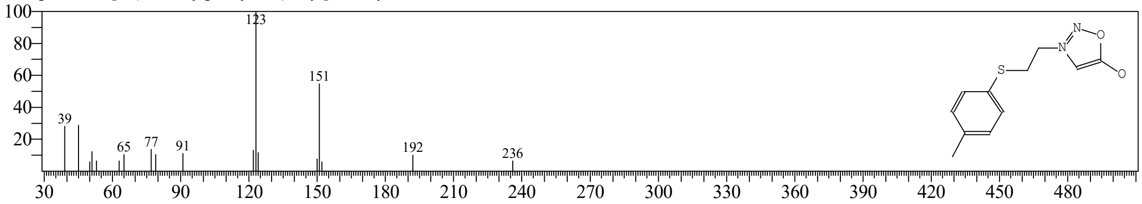

Qualitative Analysis Report

<< Target >>
Line#:64 R.Time:60.375(Scan#:13531) Retention Index:2279 MassPeaks:287
RawMode:Averaged 60.371-60.379(13530-13532) BasePeak:123.15(4277)
BG Mode:Calc. from Peak Group 1 - Event 1 Scan

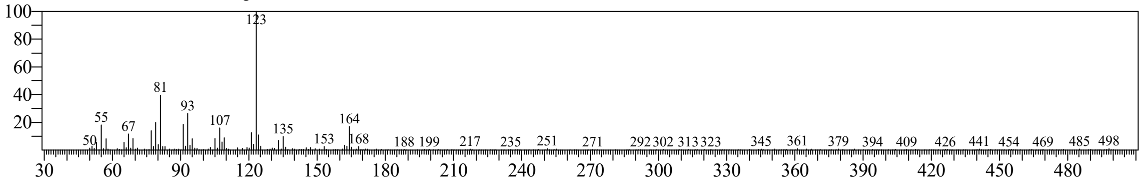

Hit#:1 Entry:155145 Library:NIST17-1.lib
SI:93 Formula:C14H7Cl2FO3 CAS:0-00-0 MolWeight:312 RetIndex:2270
CompName:2-Fluorobenzoic acid, 2-formyl-4,6-dichlorophenyl ester

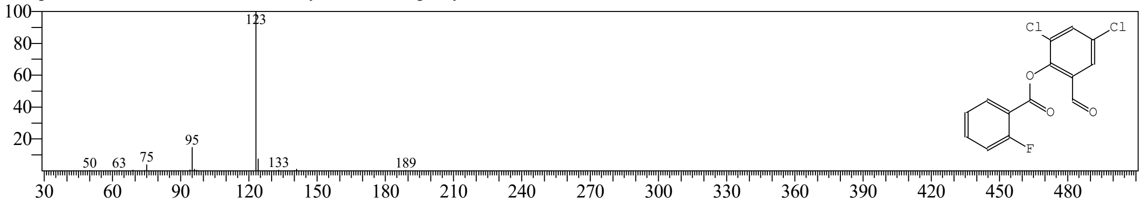

Hit#:2 Entry:155146 Library:NIST17-1.lib
SI:91 Formula:C14H7Cl2FO3 CAS:0-00-0 MolWeight:312 RetIndex:2270
CompName:4-Fluorobenzoic acid, 2-formyl-4,6-dichlorophenyl ester

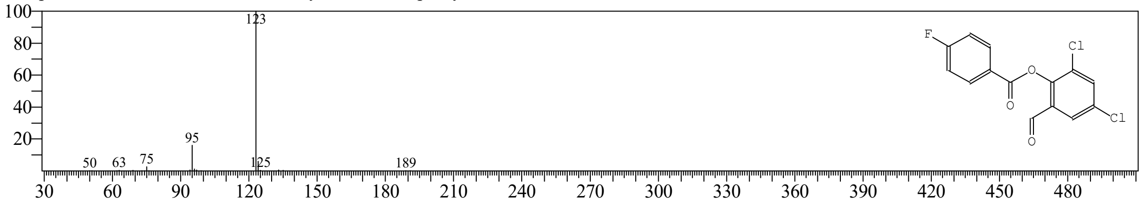

Hit#:3 Entry:155144 Library:NIST17-1.lib
SI:90 Formula:C14H7Cl2FO3 CAS:0-00-0 MolWeight:312 RetIndex:2270
CompName:3-Fluorobenzoic acid, 2-formyl-4,6-dichlorophenyl ester

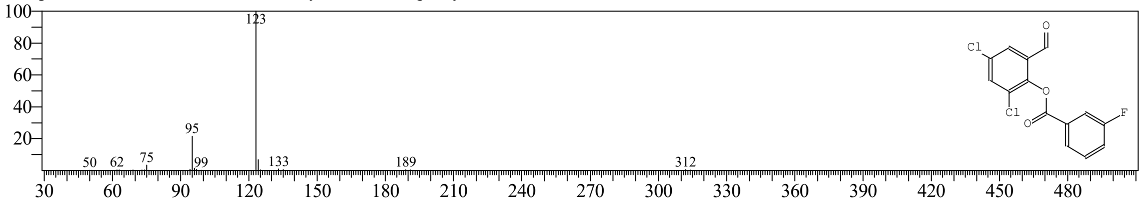

Hit#:4 Entry:93530 Library:NIST17-1.lib
SI:88 Formula:C12H14N2O2S CAS:53492-47-0 MolWeight:250 RetIndex:0
CompName:4-Methyl-3-[2-(4-methylphenylthio)ethyl]sydnone

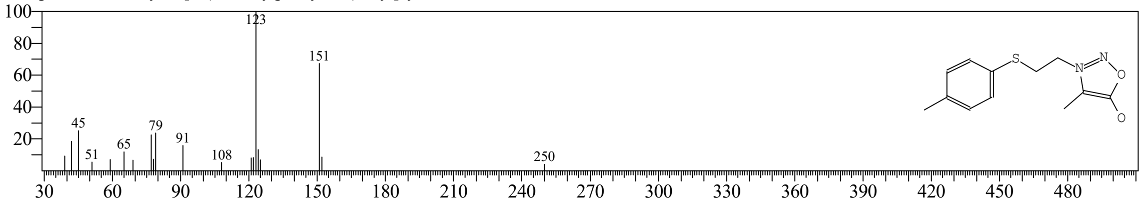

Hit#:5 Entry:36954 Library:NIST17-1.lib
SI:87 Formula:C3H9AsO4 CAS:13006-30-9 MolWeight:184 RetIndex:0
CompName:Trimethyl arsenate $$ Arsenic acid (H3Aso4), trimethyl ester $$ Trimethoxyarsine oxide $$

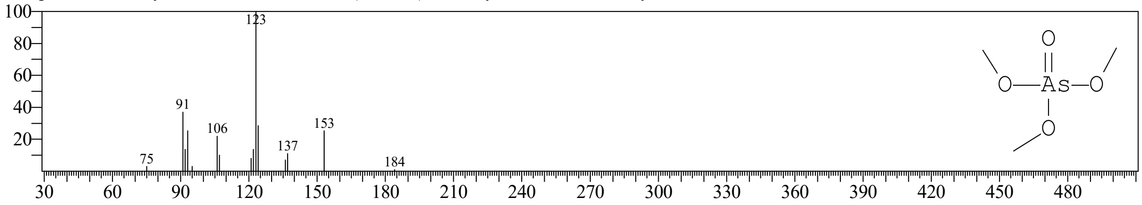

Qualitative Analysis Report

<< Target >>
Line#:65   R.Time:61.208(Scan#:13731) Retention Index:2308 MassPeaks:266
RawMode:Averaged 61.204-61.212(13730-13732) BasePeak:123.15(1274)
BG Mode:Calc. from Peak Group 1 - Event 1 Scan

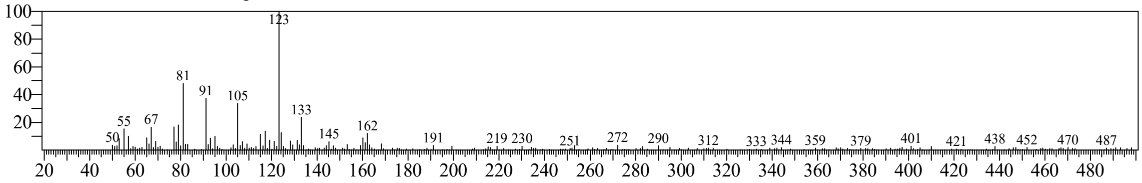

Hit#:1 Entry:93530 Library:NIST17-1.lib
SI:87 Formula:C12H14N2O2S CAS:53492-47-0 MolWeight:250 RetIndex:0
CompName:4-Methyl-3-[2-(4-methylphenylthio)ethyl]sydnone

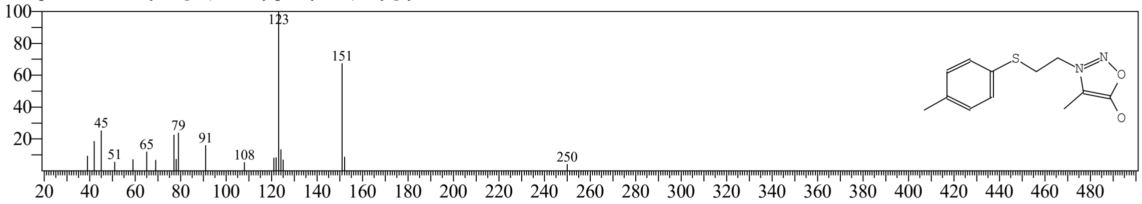

Hit#:2 Entry:36954 Library:NIST17-1.lib
SI:87 Formula:C3H9AsO4 CAS:13006-30-9 MolWeight:184 RetIndex:0
CompName:Trimethyl arsenate $$ Arsenic acid (H3Aso4), trimethyl ester $$ Trimethoxyarsine oxide $$

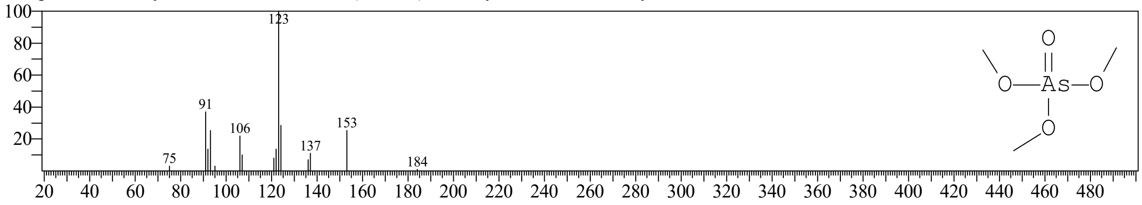

Hit#:3 Entry:65839 Library:NIST17-1.lib
SI:87 Formula:C10H12N4O2 CAS:0-00-0 MolWeight:220 RetIndex:0
CompName:Spiro[4.4]non-3-en-2-one, 4-methyl-3-(1H-tetrazol-5-yl)-1-oxa-

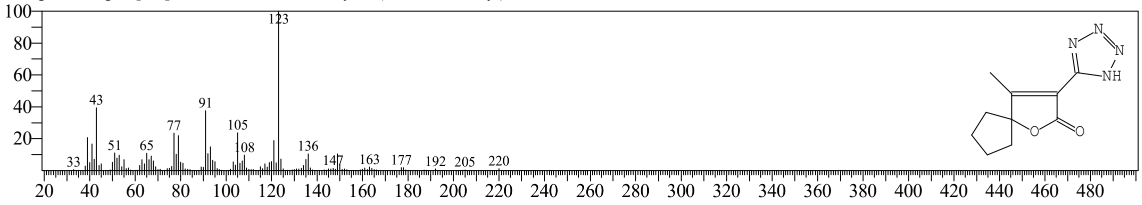

Hit#:4 Entry:17640 Library:NIST17-1.lib
SI:86 Formula:C10H16O CAS:590-71-6 MolWeight:152 RetIndex:0
CompName:2,2-Dimethylocta-3,4-dienal $$ 2,2-Dimethyl-3,4-octadienal # $$ 3,4-Octadienal, 2,2-dimethyl $$

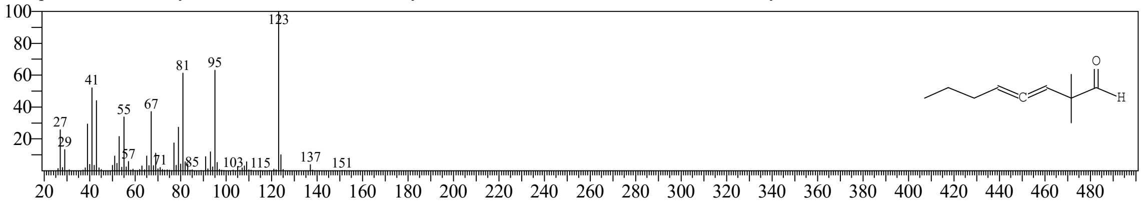

Hit#:5 Entry:26125 Library:NIST17-1.lib
SI:84 Formula:C3H9AsO3 CAS:23809-18-9 MolWeight:168 RetIndex:0
CompName:Arsonic acid, methyl-, dimethyl ester $$ Methanearsonic acid, dimethyl ester $$ Dimethyl methylarsonate $$

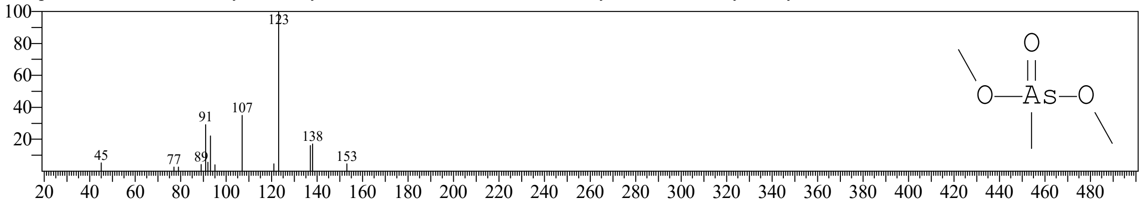

Qualitative Analysis Report

<< Target >>
Line#:66 R.Time:66.342(Scan#:14963) Retention Index:2498 MassPeaks:334
RawMode:Averaged 66.338-66.346(14962-14964) BasePeak:57.10(1115)
BG Mode:Calc. from Peak Group 1 - Event 1 Scan

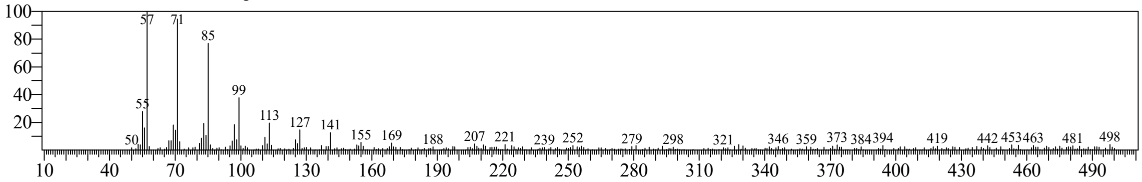

Hit#:1 Entry:760 Library:NIST17-1.lib
SI:94 Formula:CH3N5 CAS:4418-61-5 MolWeight:85 RetIndex:0
CompName:1H-Tetrazol-5-amine $$ 1H-Tetrazole, 5-amino- $$ 5-Amino-1,2,3,4-tetrazole $$ 5-Amino-1H-tetrazole $$ 5-Aminotetrazole $$ Tetrazole, 5-ar

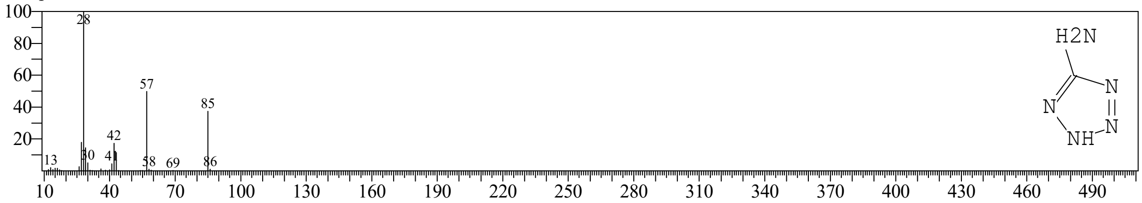

Hit#:2 Entry:102 Library:NIST17-1.lib
SI:93 Formula:C2H3NO CAS:624-83-9 MolWeight:57 RetIndex:0
CompName:Methane, isocyanato- $$ Isocyanic acid, methyl ester $$ Methyl isocyanate $$ CH3NCO $$ Isocyanate de methyle $$ Isocyanatomethane $$ M

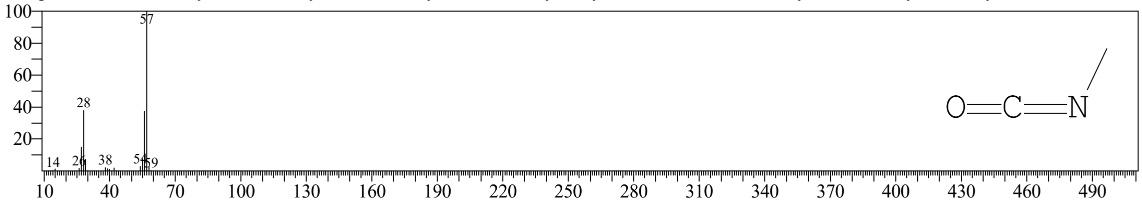

Hit#:3 Entry:951 Library:FFNSC 3.lib
SI:91 Formula:C25 H52 CAS:629-99-2 MolWeight:352 RetIndex:2500
CompName:Pentacosane <n->

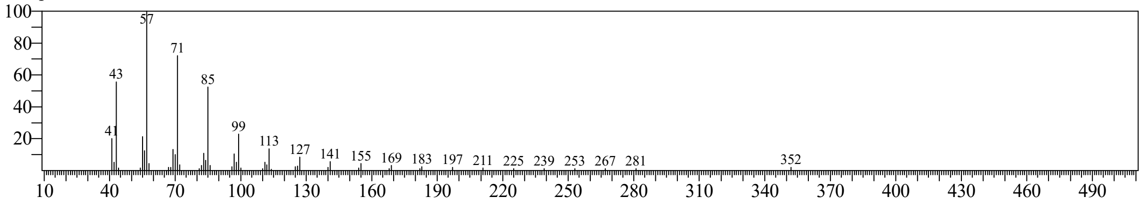

Hit#:4 Entry:58252 Library:NIST17-1.lib
SI:91 Formula:C6H9N7O2 CAS:0-00-0 MolWeight:211 RetIndex:0
CompName:6-Tert-butyl-[1,2,3]triazolo[4,5-e][1,2,3,4]tetrazine-1,3-dione

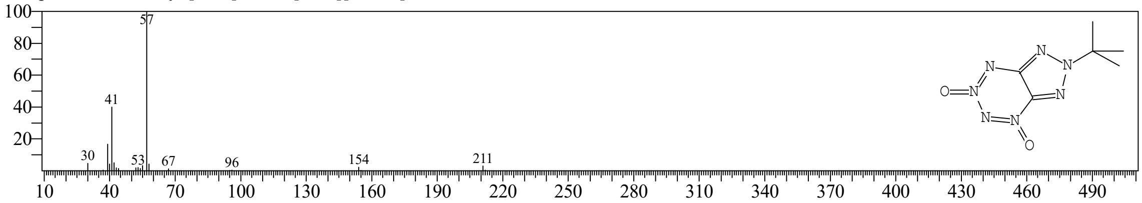

Hit#:5 Entry:196393 Library:NIST17-1.lib
SI:91 Formula:C22H47BO2 CAS:0-00-0 MolWeight:354 RetIndex:0
CompName:Boric acid, ethyl-, didecyl ester $$ Didecyl ethylboronate # $$

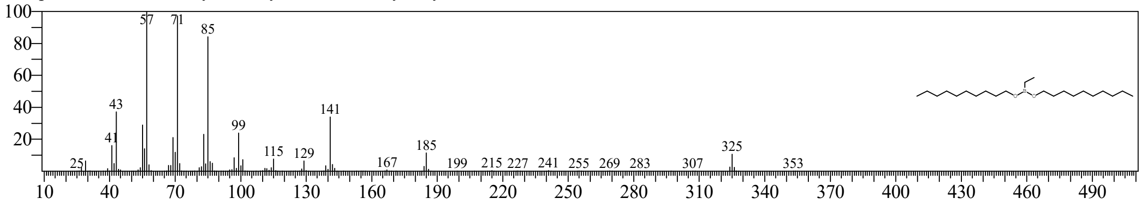

Supplement: Supplementary file 1 [file molecules-31-03346-s001.zip › Supplentary Figure S2_TIC_T cinerariifolium.pdf]
