# Supplementary material for: Larvicidal Activity, GC–MS Profiling and Multitarget Molecular Docking of Essential Oils from Cymbopogon citratus, Tanacetum cinerariifolium and Salvia rosmarinus Against Anopheles arabiensis
Source: Molecules. 2026 Sep 21;31(18):3346. doi: 10.3390/molecules31183346 (PMC13610486; doi:10.3390/molecules31183346)

Qualitative Analysis Report

Sample Information

C:\GCMSsolution\Data\Project1\21-07-25_RS302.qgd
Analyzed : 21/07/2025
1.00

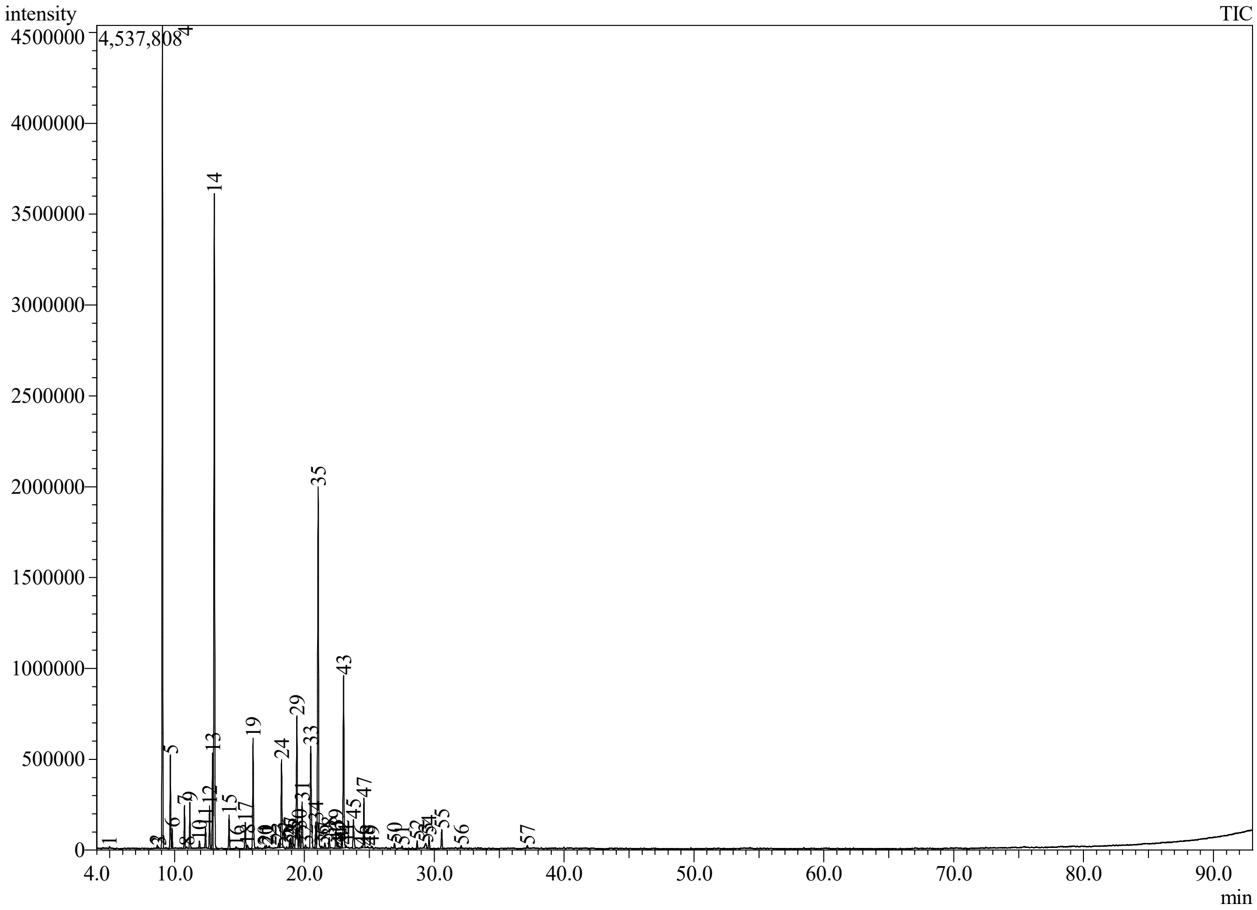

Qualitative Analysis Report

Library

<< Target >>
Line#:1 R.Time:4.983(Scan#:237) Retention Index:798! MassPeaks:232
RawMode:Averaged 4.979-4.987(236-238) BasePeak:83.05(2796)
BG Mode:Calc. from Peak Group 1 - Event 1 Scan

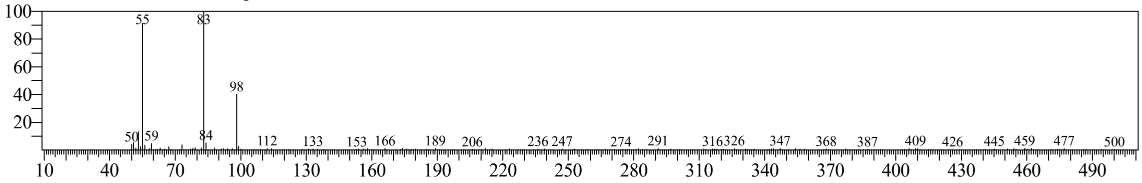

Hit#:1 Entry:1641 Library:FFNSC 3.lib
SI:97 Formula:C6 H10 O CAS:141-79-7 MolWeight:98 RetIndex:797
CompName:Isobutenyl methyl ketone

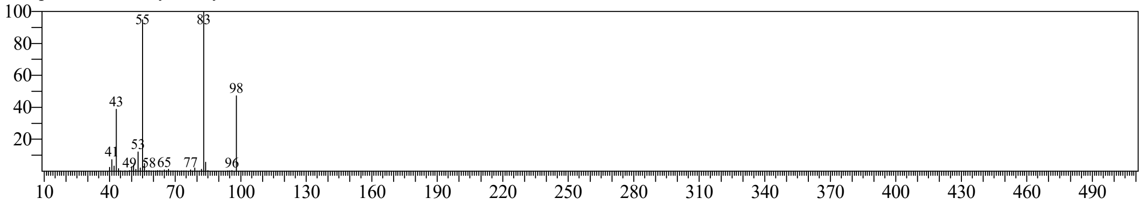

Hit#:2 Entry:7598 Library:NIST17-1.lib
SI:95 Formula:C7H12O2 CAS:10160-87-9 MolWeight:128 RetIndex:803
CompName:3,3-Diethoxy-1-propyne $$ 1-Propyne, 3,3-diethoxy- $$ Propargylaldehyde diethyl acetal $$

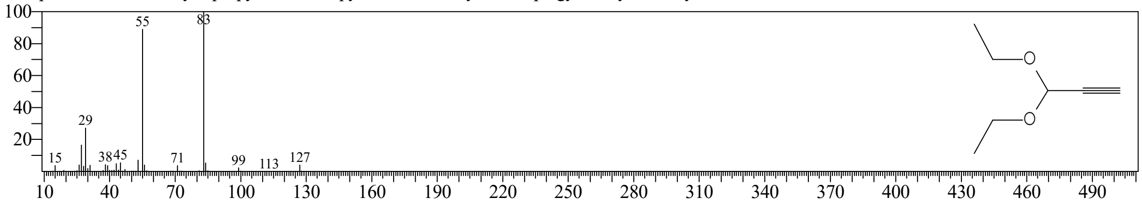

Hit#:3 Entry:6491 Library:NIST17-1.lib
SI:91 Formula:C6H11N3 CAS:19573-22-9 MolWeight:125 RetIndex:0
CompName:Cyclohexane, azido- $$ Cyclohexyl azide $$ Azidocyclohexane $$

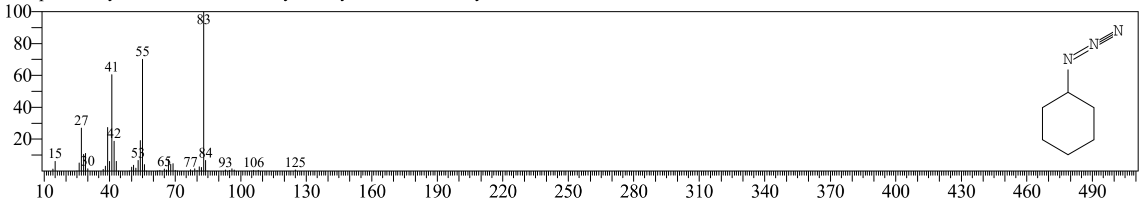

Hit#:4 Entry:181315 Library:NIST17-1.lib
SI:90 Formula:C18H30N2O4 CAS:66737-12-0 MolWeight:338 RetIndex:0
CompName:N,N'-Bis(2,6-dimethyl-6-nitrosohept-2-en-4-one)

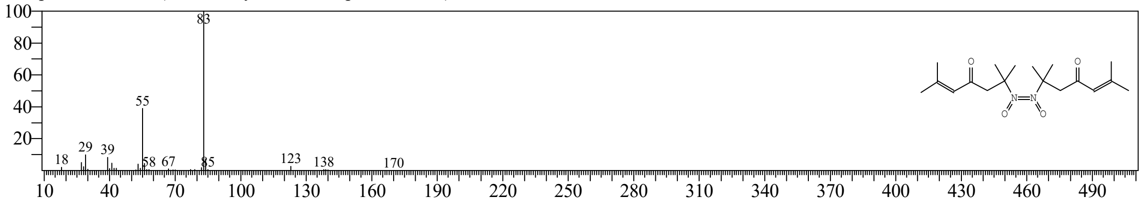

Hit#:5 Entry:3830 Library:NIST17-1.lib
SI:87 Formula:C8H16 CAS:620-00-8 MolWeight:112 RetIndex:802
CompName:3-Ethyl-2-hexene $$ (2E)-3-Ethyl-2-hexene # $$

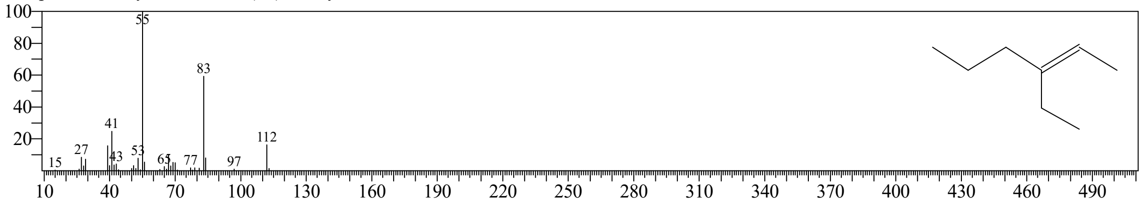

Qualitative Analysis Report

<< Target >>
Line#:2 R.Time:8.658(Scan#:1119) Retention Index:922 MassPeaks:224
RawMode:Averaged 8.654-8.662(1118-1120) BasePeak:93.10(4651)
BG Mode:Calc. from Peak Group 1 - Event 1 Scan

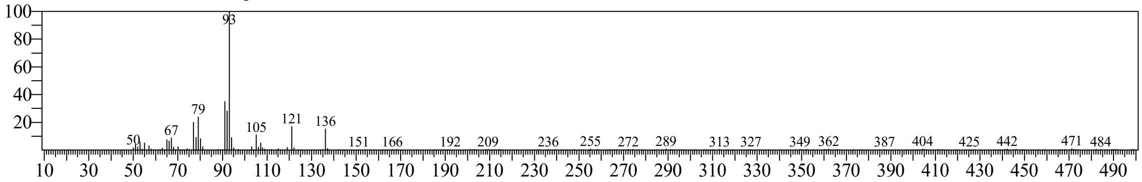

Hit#:1 Entry:986 Library:FFNSC 3.lib
SI:97 Formula:C10 H16 CAS:508-32-7 MolWeight:136 RetIndex:923
CompName:Tricyclene

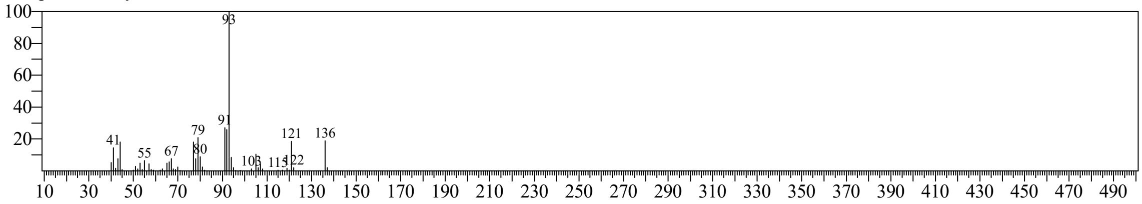

Hit#:2 Entry:3281 Library:FFNSC 3.lib
SI:97 Formula:C10 H16 CAS:4889-83-2 MolWeight:136 RetIndex:927
CompName:3-Methylapopinene

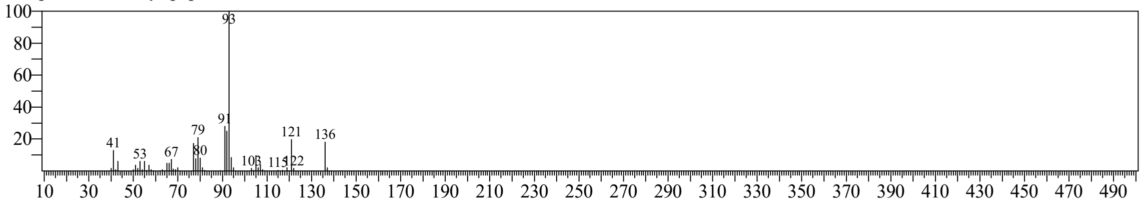

Hit#:3 Entry:10362 Library:NIST17-1.lib
SI:95 Formula:C10H16 CAS:5208-50-4 MolWeight:136 RetIndex:919
CompName:4-Carene, (1S,3S,6R)-(-)- $$ 4,7,7-Trimethylbicyclo[4.1.0]hept-2-ene # $$

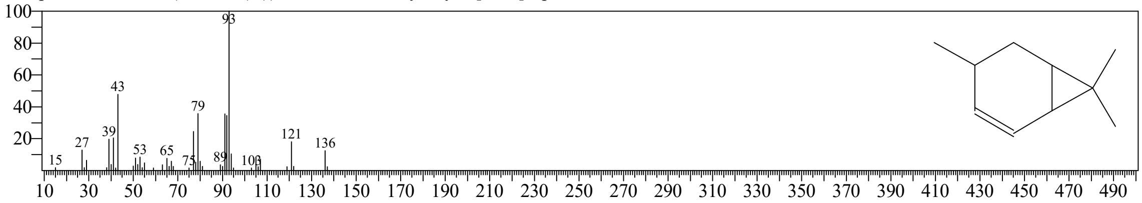

Hit#:4 Entry:10361 Library:NIST17-1.lib
SI:94 Formula:C10H16 CAS:5208-49-1 MolWeight:136 RetIndex:919
CompName:4-Carene, (1S,3R,6R)-(-)- $$ 4,7,7-Trimethylbicyclo[4.1.0]hept-2-ene # $$

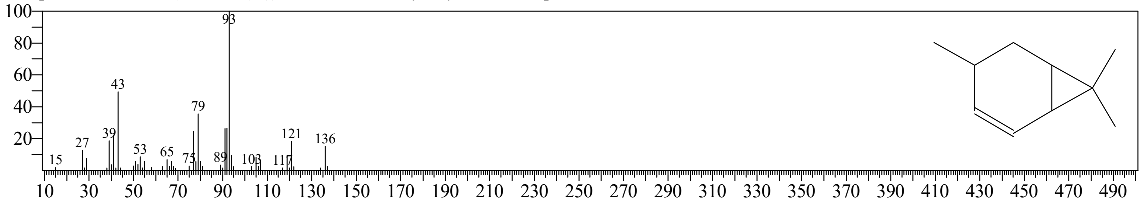

Hit#:5 Entry:24214 Library:NIST17-1.lib
SI:93 Formula:C10H16N2 CAS:87143-58-6 MolWeight:164 RetIndex:0
CompName:3,5-Methanocyclopentapyrazole, 3,3a,4,5,6,6a-hexahydro-3a,4,4-trimethyl-

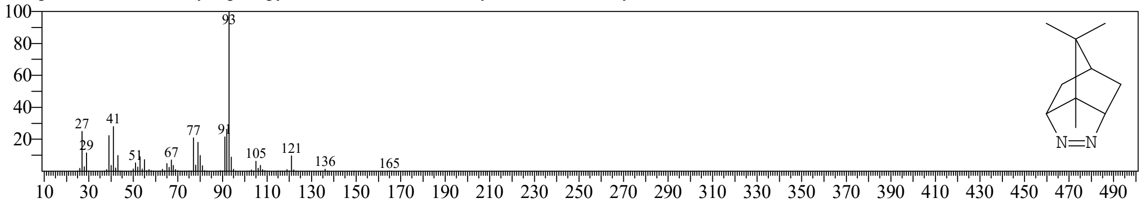

Qualitative Analysis Report

<< Target >>
Line#:3 R.Time:8.762(Scan#:1144) Retention Index:925 MassPeaks:230
RawMode:Averaged 8.758-8.767(1143-1145) BasePeak:93.10(1903)
BG Mode:Calc. from Peak Group 1 - Event 1 Scan

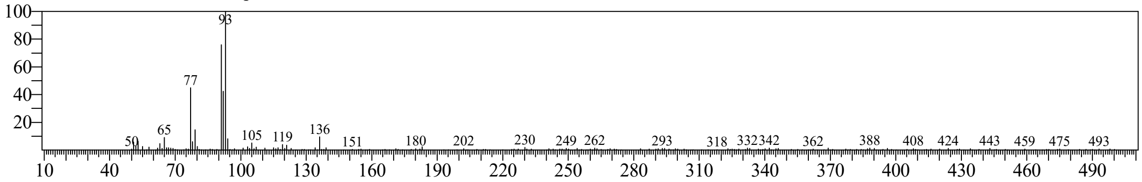

Hit#:1 Entry:957 Library:FFNSC 3.lib
SI:96 Formula:C10 H16 CAS:2867-05-2 MolWeight:136 RetIndex:927
CompName:Thujene <alpha->

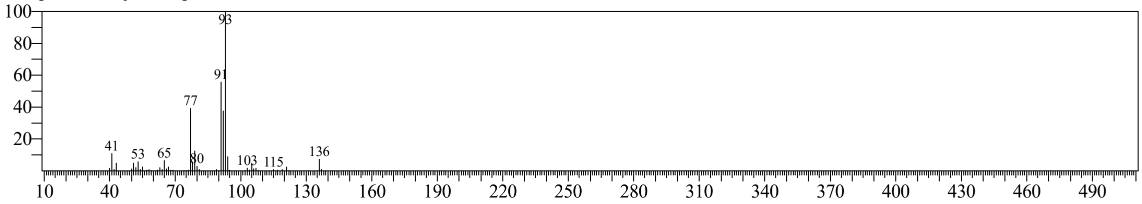

Hit#:2 Entry:10127 Library:NIST17-1.lib
SI:91 Formula:C8H12N2 CAS:109746-10-3 MolWeight:136 RetIndex:0
CompName:1,4-Methano-1H-Cyclopropa[d]pyridazine, 4,4a,5,5a-tetrahydro-6,6-dimethyl-, (1.alpha.,4.alpha.,4a.alpha.,5a.alpha.)-

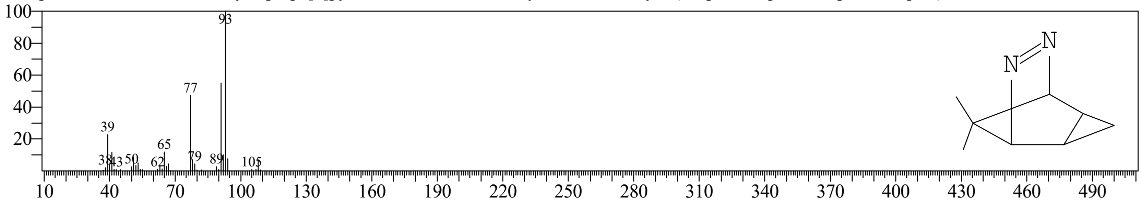

Hit#:3 Entry:817 Library:FFNSC 3.lib
SI:90 Formula:C10 H16 CAS:80-56-8 MolWeight:136 RetIndex:933
CompName:Pinene <alpha->

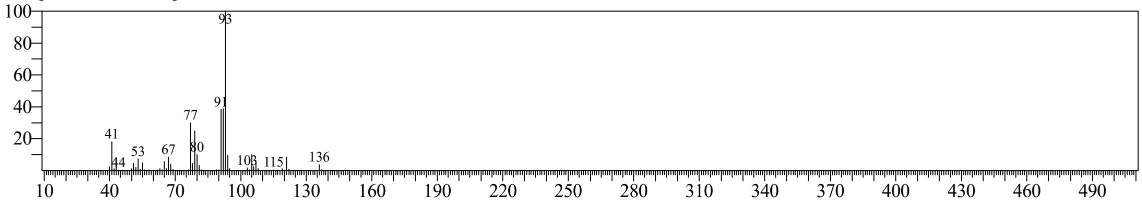

Hit#:4 Entry:10362 Library:NIST17-1.lib
SI:89 Formula:C10H16 CAS:5208-50-4 MolWeight:136 RetIndex:919
CompName:4-Carene, (1S,3S,6R)-(-)- $$ 4,7,7-Trimethylbicyclo[4.1.0]hept-2-ene # $$

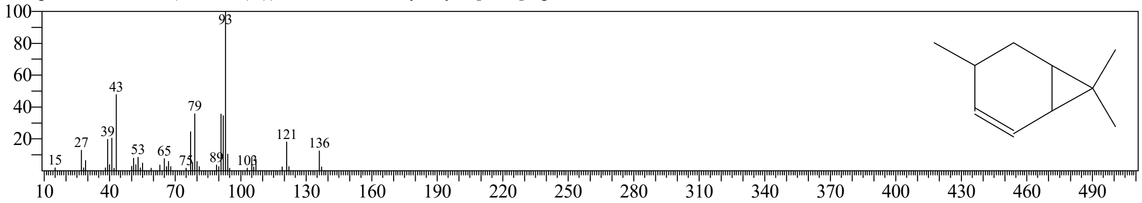

Hit#:5 Entry:10361 Library:NIST17-1.lib
SI:87 Formula:C10H16 CAS:5208-49-1 MolWeight:136 RetIndex:919
CompName:4-Carene, (1S,3R,6R)-(-)- $$ 4,7,7-Trimethylbicyclo[4.1.0]hept-2-ene # $$

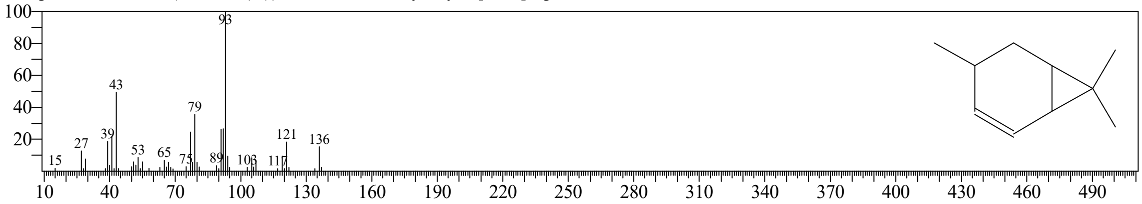

Qualitative Analysis Report

<< Target >>
Line#:4 R.Time:9.067(Scan#:1217) Retention Index:933 MassPeaks:305
RawMode:Averaged 9.063-9.071(1216-1218) BasePeak:93.10(1181581)
BG Mode:Calc. from Peak Group 1 - Event 1 Scan

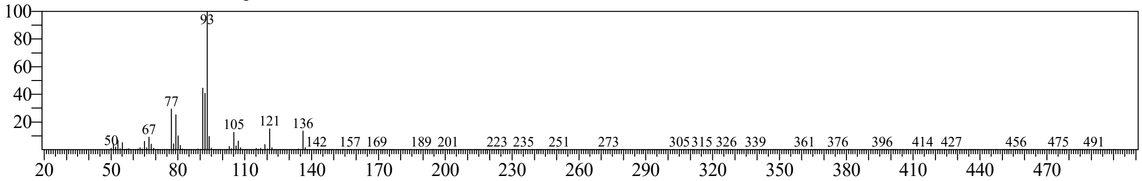

Hit#:1 Entry:817 Library:FFNSC 3.lib
SI:97 Formula:C10 H16 CAS:80-56-8 MolWeight:136 RetIndex:933
CompName:Pinene <alpha->

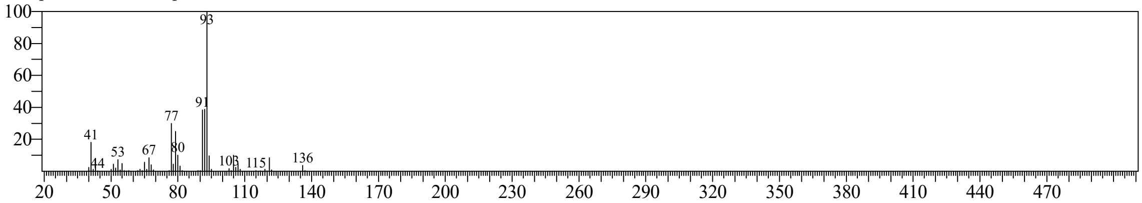

Hit#:2 Entry:986 Library:FFNSC 3.lib
SI:93 Formula:C10 H16 CAS:508-32-7 MolWeight:136 RetIndex:923
CompName:Tricyclene

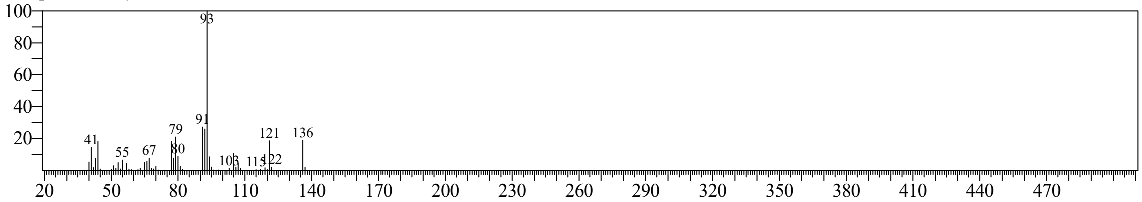

Hit#:3 Entry:3281 Library:FFNSC 3.lib
SI:93 Formula:C10 H16 CAS:4889-83-2 MolWeight:136 RetIndex:927
CompName:3-Methylapopinene

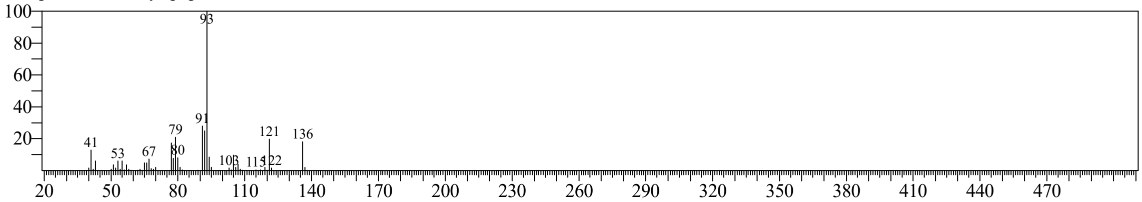

Hit#:4 Entry:10374 Library:NIST17-1.lib
SI:93 Formula:C10H16 CAS:68998-21-0 MolWeight:136 RetIndex:928
CompName:Cyclopropane, 1,1-dimethyl-2-(3-methyl-1,3-butadienyl)- $$ 1,1-Dimethyl-2-[(1E)-3-methyl-1,3-butadienyl]cyclopropane # $$

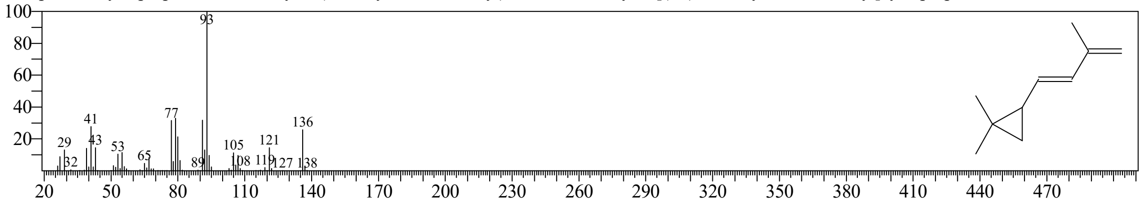

Hit#:5 Entry:24214 Library:NIST17-1.lib
SI:93 Formula:C10H16N2 CAS:87143-58-6 MolWeight:164 RetIndex:0
CompName:3,5-Methanocyclopentapyrazole, 3,3a,4,5,6,6a-hexahydro-3a,4,4-trimethyl-

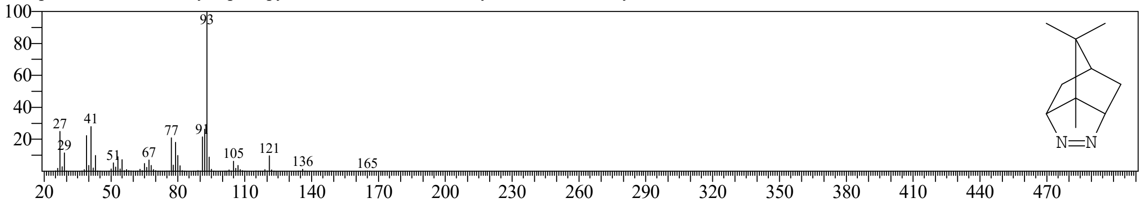

Qualitative Analysis Report

<< Target >>
Line#:5 R.Time:9.675(Scan#:1363) Retention Index:948 MassPeaks:234
RawMode:Averaged 9.671-9.679(1362-1364) BasePeak:93.10(102153)
BG Mode:Calc. from Peak Group 1 - Event 1 Scan

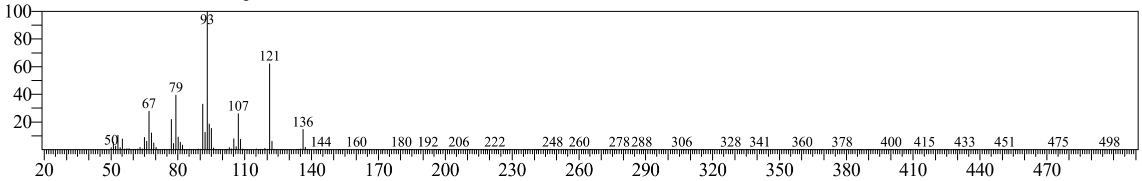

Hit#:1 Entry:3282 Library:FFNSC 3.lib
SI:99 Formula:C10 H16 CAS:497-32-5 MolWeight:136 RetIndex:954
CompName:2,2-Dimethyl-5-methylene norbornane

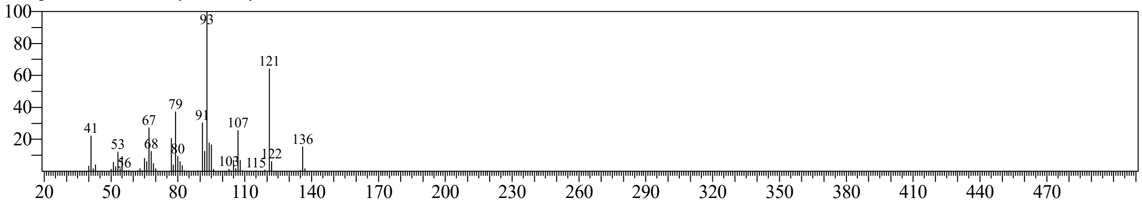

Hit#:2 Entry:952 Library:FFNSC 3.lib
SI:99 Formula:C10 H16 CAS:79-92-5 MolWeight:136 RetIndex:953
CompName:Camphene

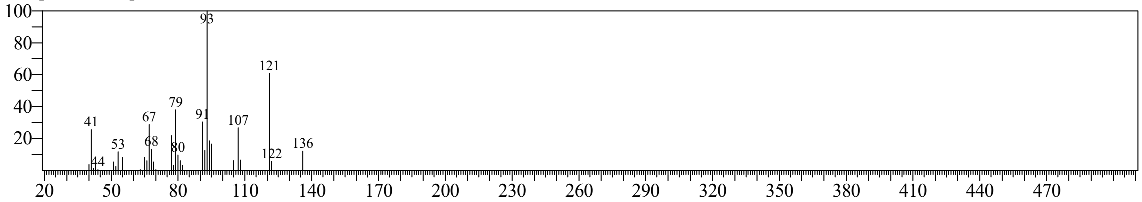

Hit#:3 Entry:10395 Library:NIST17-1.lib
SI:96 Formula:C10H16 CAS:5794-04-7 MolWeight:136 RetIndex:943
CompName:Bicyclo[2.2.1]heptane, 2,2-dimethyl-3-methylene-, (1S)- $$ 2,2-Dimethyl-3-methylenebicyclo[2.2.1]heptane $$

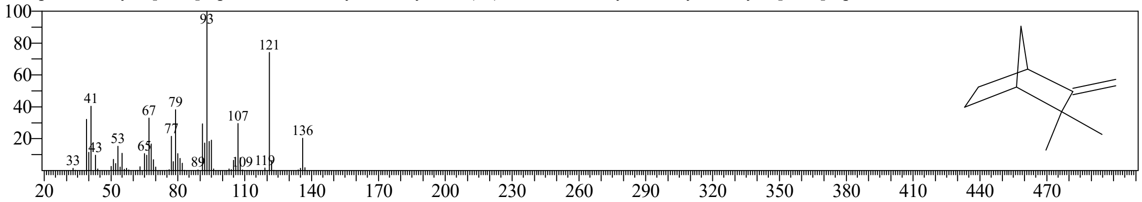

Hit#:4 Entry:10401 Library:NIST17-1.lib
SI:92 Formula:C10H16 CAS:1727-69-1 MolWeight:136 RetIndex:956
CompName:Cyclopentene, 4-ethenyl-1,5,5-trimethyl- $$ 1,5,5-Trimethyl-4-vinyl-1-cyclopentene # $$

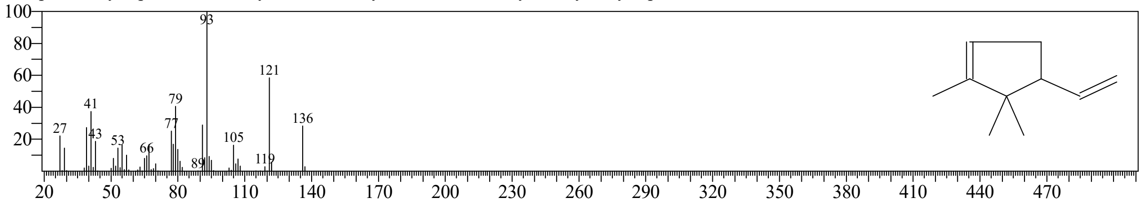

Hit#:5 Entry:10373 Library:NIST17-1.lib
SI:91 Formula:C10H16 CAS:471-84-1 MolWeight:136 RetIndex:943
CompName:Bicyclo[2.2.1]heptane, 7,7-dimethyl-2-methylene- $$ Norbornane, 7,7-dimethyl-2-methylene- $$ .alpha.-Fenchene $$ Fenchene $$ 7,7-Dimethy

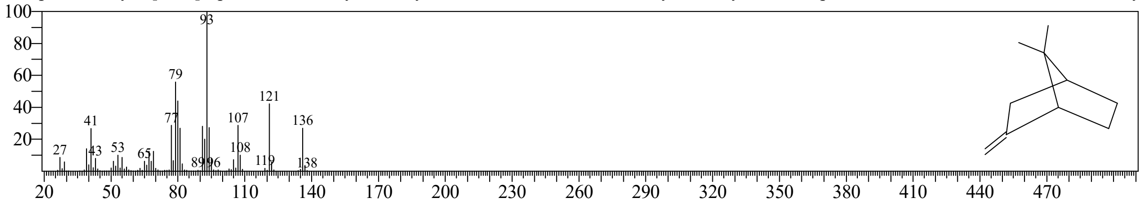

Qualitative Analysis Report

<< Target >>
Line#:6   R.Time:9.813(Scan#:1396) Retention Index:952 MassPeaks:254
RawMode:Averaged 9.808-9.817(1395-1397) BasePeak:91.10(36872)
BG Mode:Calc. from Peak Group 1 - Event 1 Scan

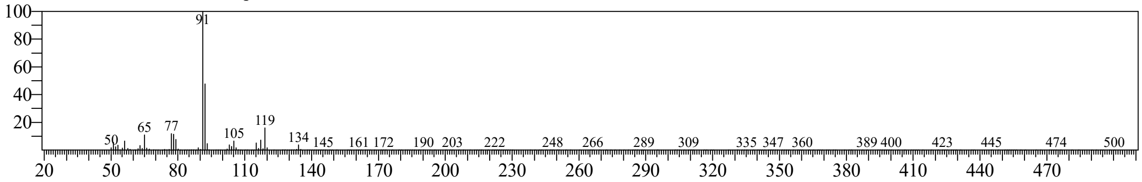

Hit#:1 Entry:556 Library:FFNSC 3.lib
SI:98 Formula:C10 H14 CAS:36262-09-6 MolWeight:134 RetIndex:953
CompName:Thuja-2,4(10)-diene

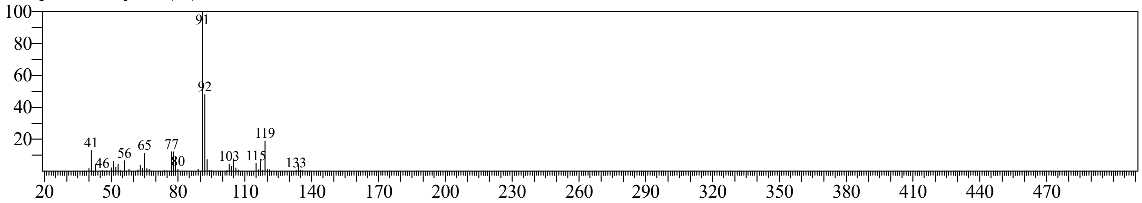

Hit#:2 Entry:231901 Library:NIST17-1.lib
SI:91 Formula:C13H14Fe2O4S2 CAS:12215-56-4 MolWeight:410 RetIndex:0
CompName:Iron, [(2,3,5,6-.eta.)-bicyclo[2.2.1]hepta-2,5-diene]tetracarbonylbis[.mu.-(methanethiolato)]di-, (Fe-Fe)

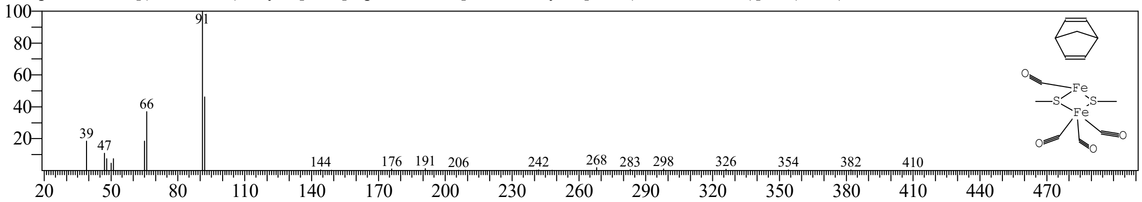

Hit#:3 Entry:5580 Library:NIST17-1.lib
SI:89 Formula:C9H12 CAS:17634-51-4 MolWeight:120 RetIndex:946
CompName:1,3,5-Cycloheptatriene, 7-ethyl- $$ 7-Ethyl-1,3,5-cycloheptatriene $$ 7-Ethylcycloheptatriene $$

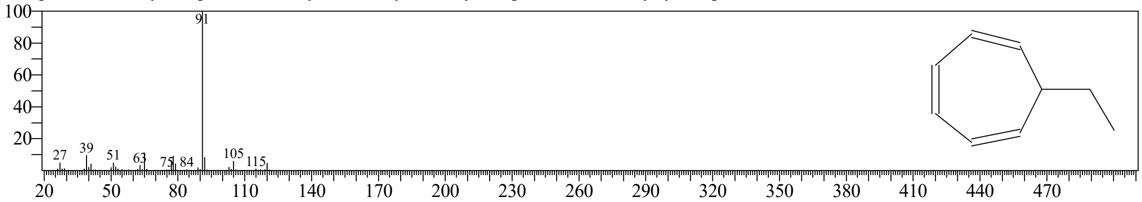

Hit#:4 Entry:93293 Library:NIST17-1.lib
SI:89 Formula:C10H10FeO4 CAS:32716-70-4 MolWeight:250 RetIndex:0
CompName:Iron, tricarbonyl[(2,3,4,5-.eta.)-2,4-cycloheptadien-1-ol]-

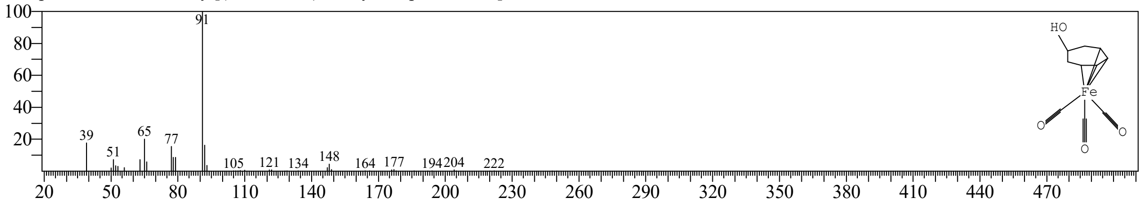

Hit#:5 Entry:168210 Library:NIST17-1.lib
SI:89 Formula:C13H7N7O4 CAS:1394125-25-7 MolWeight:325 RetIndex:0
CompName:12-Benzyl-4,9,15-trioxa-3,5,8,10,12,14,16-heptaazatetracyclo[11.3.0.0(2,6).0(7,11)]hexadeca-1(16),2,5,7,10,13-hexaen-3-one

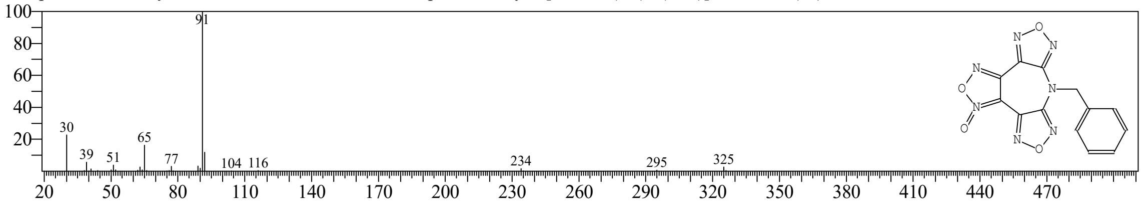

Qualitative Analysis Report

<< Target >>
Line#:7 R.Time:10.762(Scan#:1624) Retention Index:977 MassPeaks:275
RawMode:Averaged 10.758-10.767(1623-1625) BasePeak:93.10(64064)
BG Mode:Calc. from Peak Group 1 - Event 1 Scan

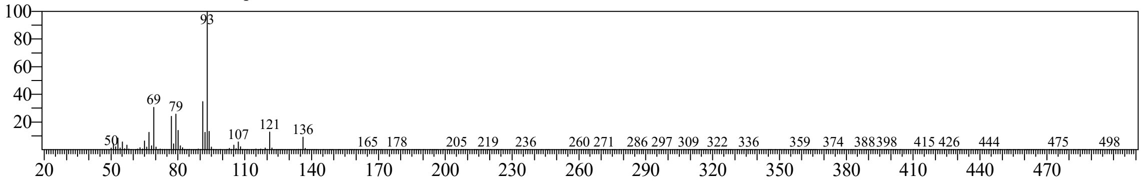

Hit#:1 Entry:837 Library:FFNSC 3.lib
SI:96 Formula:C10 H16 CAS:127-91-3 MolWeight:136 RetIndex:978
CompName:Pinene <beta->

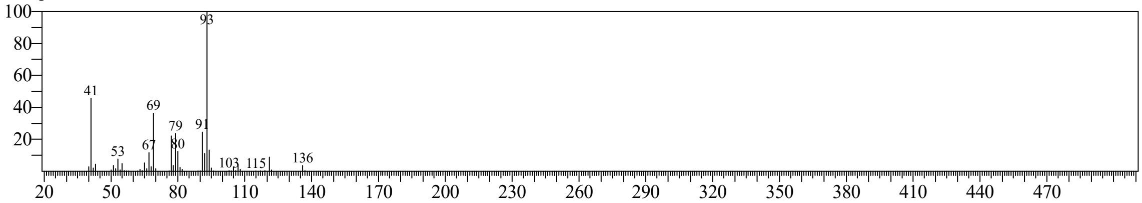

Hit#:2 Entry:958 Library:FFNSC 3.lib
SI:94 Formula:C10 H16 CAS:3387-41-5 MolWeight:136 RetIndex:972
CompName:Sabinene

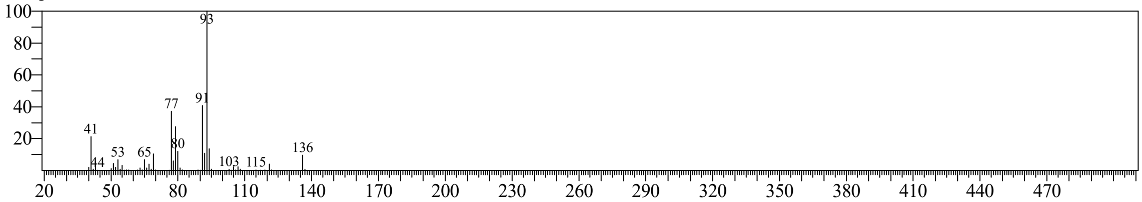

Hit#:3 Entry:10357 Library:NIST17-1.lib
SI:92 Formula:C10H16 CAS:3779-61-1 MolWeight:136 RetIndex:976
CompName:trans-.beta.-Ocimene $$ 1,3,6-Octatriene, 3,7-dimethyl-, (E)- $$ .beta.-trans-Ocimene $$ trans-3,7-Dimethyl-1,3,6-Octatriene $$ Ocimene, trans

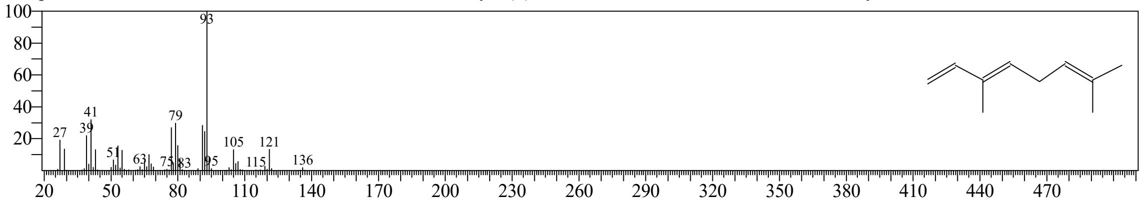

Hit#:4 Entry:24214 Library:NIST17-1.lib
SI:91 Formula:C10H16N2 CAS:87143-58-6 MolWeight:164 RetIndex:0
CompName:3,5-Methanocyclopentapyrazole, 3,3a,4,5,6,6a-hexahydro-3a,4,4-trimethyl-

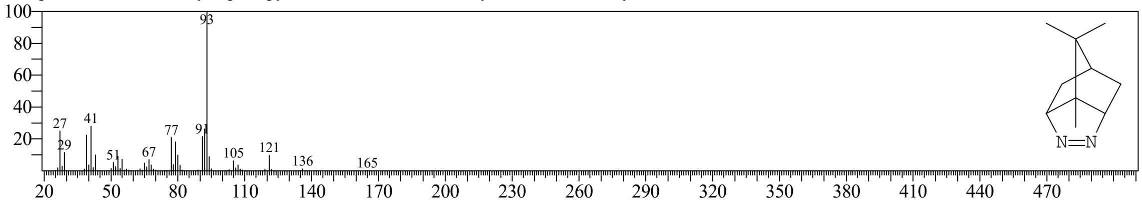

Hit#:5 Entry:10358 Library:NIST17-1.lib
SI:91 Formula:C10H16 CAS:3338-55-4 MolWeight:136 RetIndex:976
CompName:1,3,6-Octatriene, 3,7-dimethyl-, (Z)- $$ .beta.-cis-Ocimene $$ cis-.beta.-Ocimene $$ cis-3,7-Dimethyl-1,3,6-octatriene $$ Ocimene, cis-.beta.- $

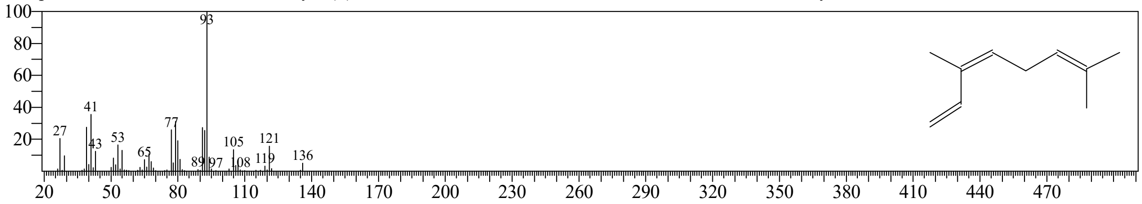

Qualitative Analysis Report

<< Target >>
Line#:8   R.Time:10.929(Scan#:1664) Retention Index:981 MassPeaks:265
RawMode:Averaged 10.925-10.933(1663-1665) BasePeak:108.10(575)
BG Mode:Calc. from Peak Group 1 - Event 1 Scan

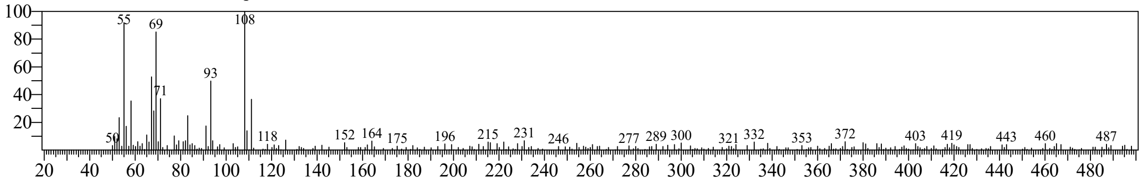

Hit#:1 Entry:675 Library:FFNSC 3.lib
SI:95 Formula:C8 H14 O CAS:110-93-0 MolWeight:126 RetIndex:986
CompName:Hept-5-en-2-one <6-methyl->

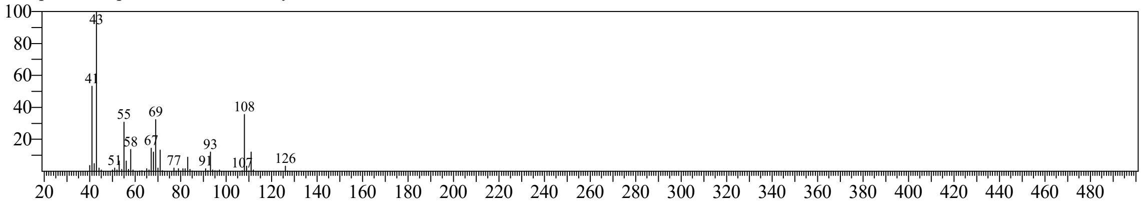

Hit#:2 Entry:110970 Library:NIST17-1.lib
SI:86 Formula:C14H16CrO2 CAS:57820-92-5 MolWeight:268 RetIndex:0
CompName:Chromium, bis(anisole)-

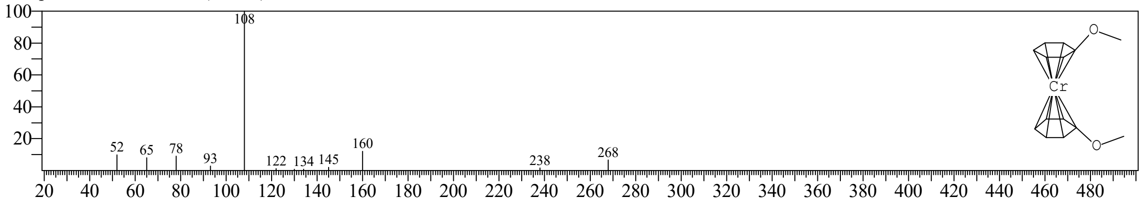

Hit#:3 Entry:2939 Library:NIST17-1.lib
SI:84 Formula:C6H8Si CAS:63878-65-9 MolWeight:108 RetIndex:0
CompName:Silabenzene,1-methyl- $$ 1-Methyl-1-silabenzene $$ 1-Methylsiline # $$

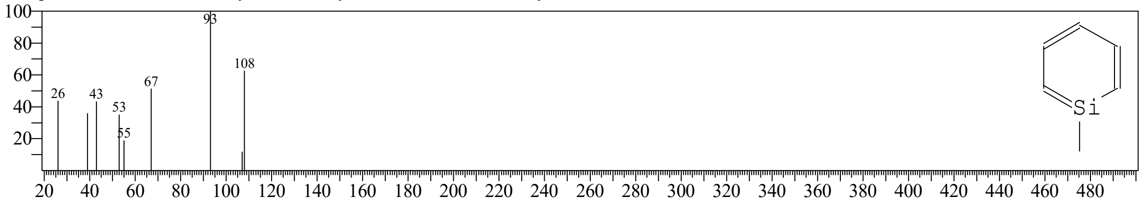

Hit#:4 Entry:3052 Library:NIST17-1.lib
SI:81 Formula:C8H12 CAS:7124-40-5 MolWeight:108 RetIndex:0
CompName:1,2-Cyclooctadiene

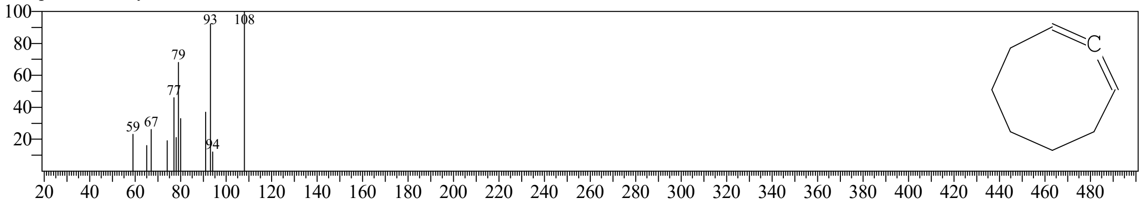

<< Target >>
Line#:9 R.Time:11.179(Scan#:1724) Retention Index:987 MassPeaks:237
RawMode:Averaged 11.175-11.183(1723-1725) BasePeak:93.10(68694)
BG Mode:Calc. from Peak Group 1 - Event 1 Scan

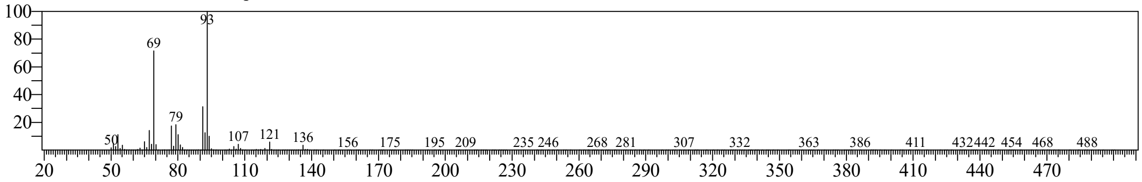

Hit#:1 Entry:628 Library:FFNSC 3.lib
SI:97 Formula:C10 H16 CAS:123-35-3 MolWeight:136 RetIndex:991
CompName:Myrcene

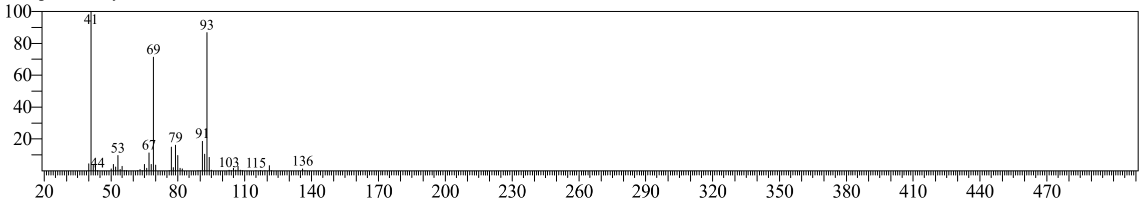

Hit#:2 Entry:837 Library:FFNSC 3.lib
SI:94 Formula:C10 H16 CAS:127-91-3 MolWeight:136 RetIndex:978
CompName:Pinene <beta->

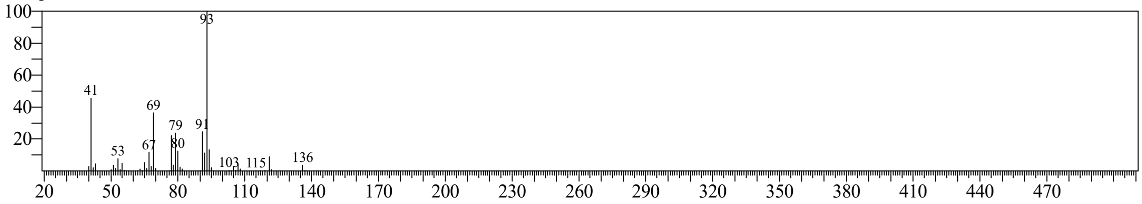

Hit#:3 Entry:24214 Library:NIST17-1.lib
SI:90 Formula:C10H16N2 CAS:87143-58-6 MolWeight:164 RetIndex:0
CompName:3,5-Methanocyclopentapyrazole, 3,3a,4,5,6,6a-hexahydro-3a,4,4-trimethyl-

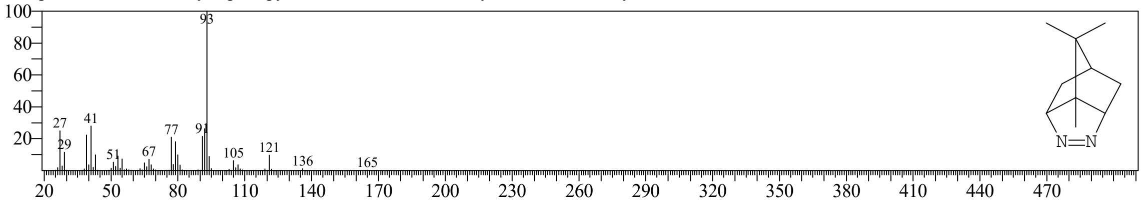

Hit#:4 Entry:10367 Library:NIST17-1.lib
SI:88 Formula:C10H16 CAS:99-84-3 MolWeight:136 RetIndex:993
CompName:Cyclohexene, 4-methylene-1-(1-methylethyl)- $$ p-Mentha-1(7),3-diene $$ .beta.-Terpinen $$ .beta.-Terpinene $$ Beta terpinene $$ 1-Isopropy

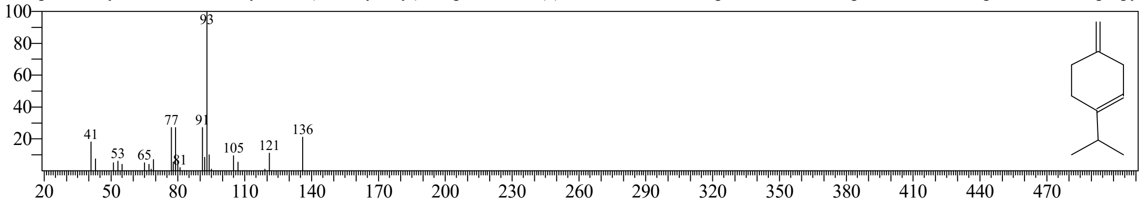

Hit#:5 Entry:10127 Library:NIST17-1.lib
SI:88 Formula:C8H12N2 CAS:109746-10-3 MolWeight:136 RetIndex:0
CompName:1,4-Methano-1H-Cyclopropa[d]pyridazine, 4,4a,5,5a-tetrahydro-6,6-dimethyl-, (1.alpha.,4.alpha.,4a.alpha.,5a.alpha.)-

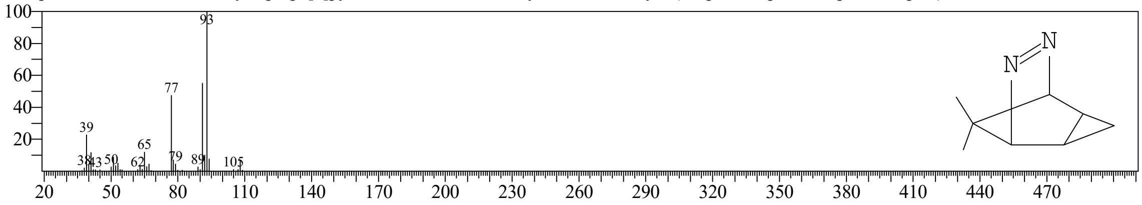

Qualitative Analysis Report

<< Target >>
Line#:10 R.Time:11.913(Scan#:1900) Retention Index:1006 MassPeaks:266
RawMode:Averaged 11.908-11.917(1899-1901) BasePeak:93.10(9764)
BG Mode:Calc. from Peak Group 1 - Event 1 Scan

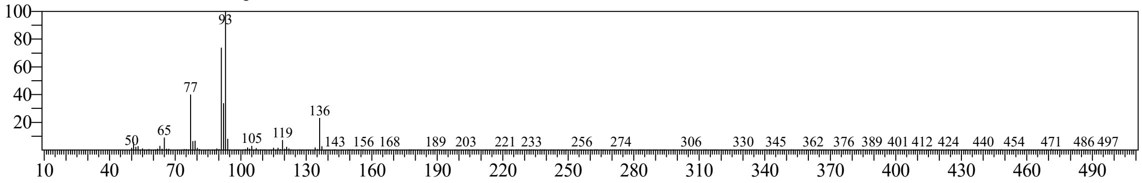

Hit#:1 Entry:648 Library:FFNSC 3.lib
SI:95 Formula:C10 H16 CAS:99-83-2 MolWeight:136 RetIndex:1007
CompName:Phellandrene <alpha->

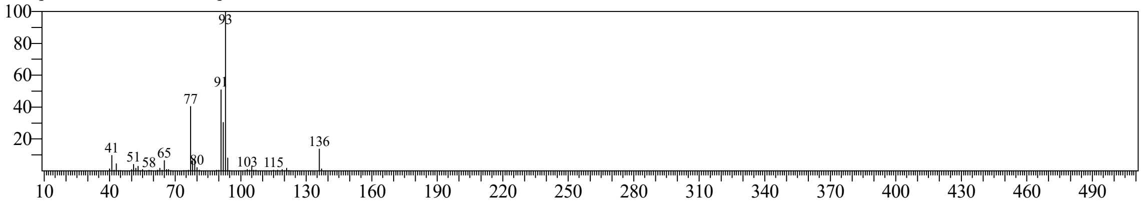

Hit#:2 Entry:10127 Library:NIST17-1.lib
SI:92 Formula:C8H12N2 CAS:109746-10-3 MolWeight:136 RetIndex:0
CompName:1,4-Methano-1H-Cyclopropa[d]pyridazine, 4,4a,5,5a-tetrahydro-6,6-dimethyl-, (1.alpha.,4.alpha.,4a.alpha.,5a.alpha.)-

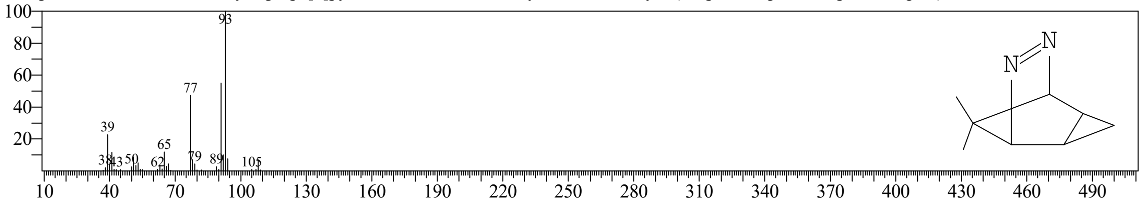

Hit#:3 Entry:10391 Library:NIST17-1.lib
SI:88 Formula:C10H16 CAS:99-85-4 MolWeight:136 RetIndex:998
CompName:.gamma.-Terpinene $$ 1,4-Cyclohexadiene, 1-methyl-4-(1-methylethyl)- $$ .gamma.-Terpinen $$ p-Mentha-1,4-diene $$ Crithmene $$ Moslen

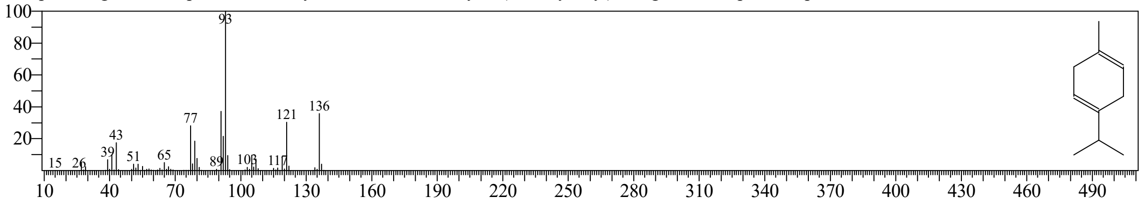

Hit#:4 Entry:1124 Library:FFNSC 3.lib
SI:85 Formula:C10 H16 CAS:13466-78-9 MolWeight:136 RetIndex:1009
CompName:Carene <delta-3->

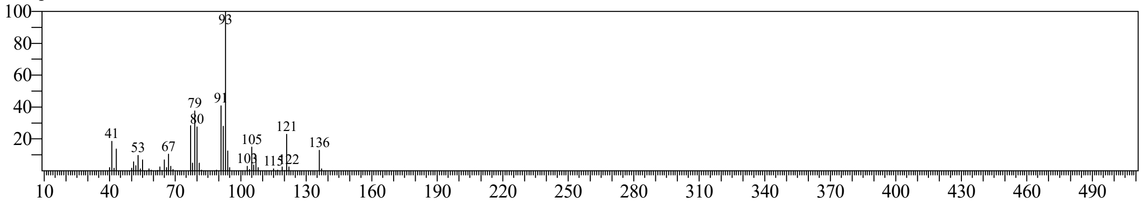

Hit#:5 Entry:24214 Library:NIST17-1.lib
SI:85 Formula:C10H16N2 CAS:87143-58-6 MolWeight:164 RetIndex:0
CompName:3,5-Methanocyclopentapyrazole, 3,3a,4,5,6,6a-hexahydro-3a,4,4-trimethyl-

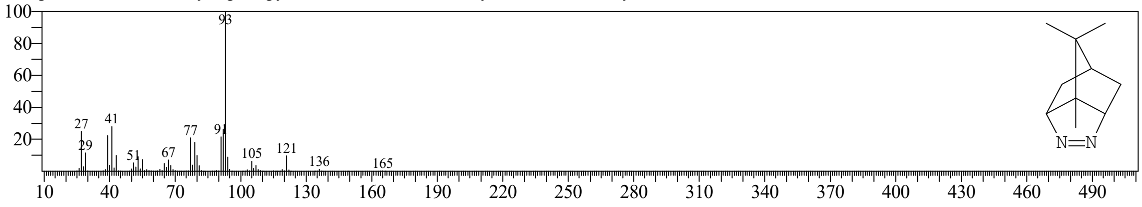

Qualitative Analysis Report

<< Target >>
Line#:11   R.Time:12.379(Scan#:2012) Retention Index:1016 MassPeaks:267
RawMode:Averaged 12.375-12.383(2011-2013) BasePeak:93.10(19139)
BG Mode:Calc. from Peak Group 1 - Event 1 Scan

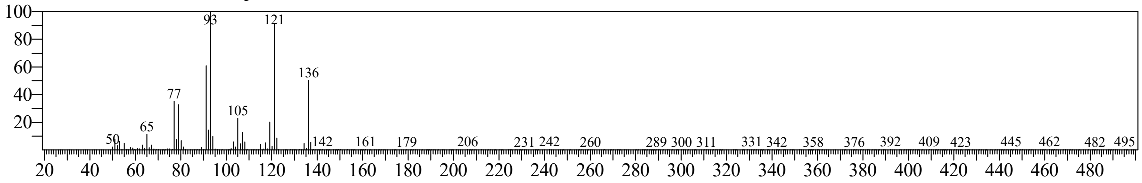

Hit#:1 Entry:277 Library:FFNSC 3.lib
SI:97 Formula:C10 H16 CAS:99-86-5 MolWeight:136 RetIndex:1018
CompName:Terpinene <alpha->

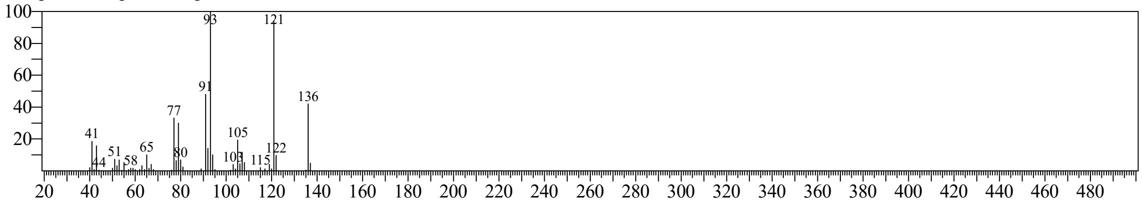

Hit#:2 Entry:10402 Library:NIST17-1.lib
SI:93 Formula:C10H16 CAS:99805-90-0 MolWeight:136 RetIndex:1023
CompName:Cyclohexene, 4-methyl-3-(1-methylethylidene)- $$ 4-Methyl-3-(1-methylethylidene)-1-cyclohexene # $$

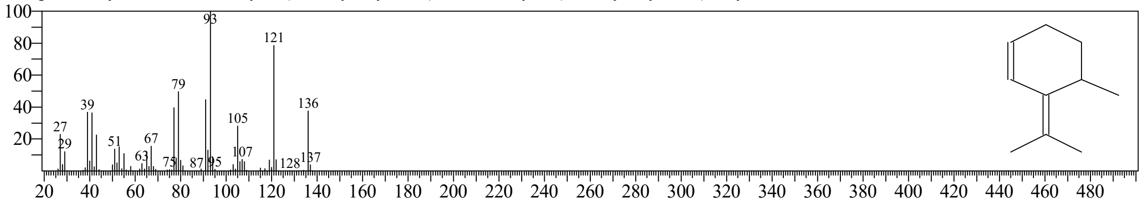

Hit#:3 Entry:10409 Library:NIST17-1.lib
SI:93 Formula:C10H16 CAS:586-63-0 MolWeight:136 RetIndex:1023
CompName:Cyclohexene, 3-methyl-6-(1-methylethylidene)- $$ p-Mentha-2,4(8)-diene $$ Isoterpinolene $$ 3-Methyl-6-(1-methylethylidene)-1-cyclohexene

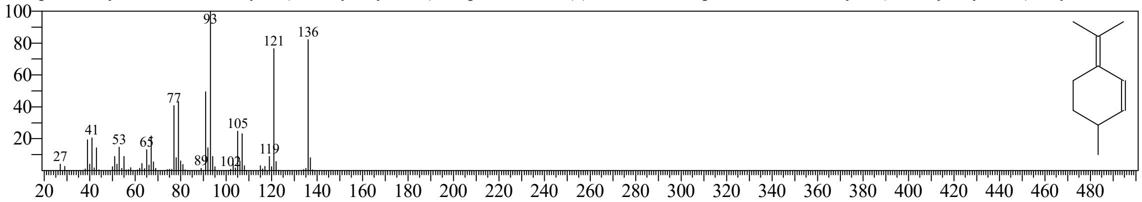

Hit#:4 Entry:10127 Library:NIST17-1.lib
SI:92 Formula:C8H12N2 CAS:109746-10-3 MolWeight:136 RetIndex:0
CompName:1,4-Methano-1H-Cyclopropa[d]pyridazine, 4,4a,5,5a-tetrahydro-6,6-dimethyl-, (1.alpha.,4.alpha.,4a.alpha.,5a.alpha.)-

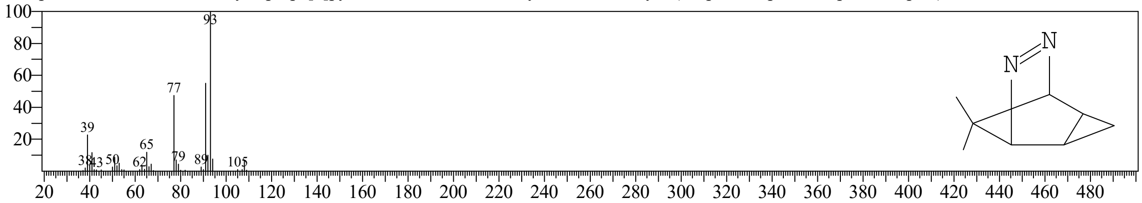

Hit#:5 Entry:2330 Library:FFNSC 3.lib
SI:89 Formula:C10 H16 CAS:499-03-6 MolWeight:136 RetIndex:1011
CompName:Sylvestrene <iso->

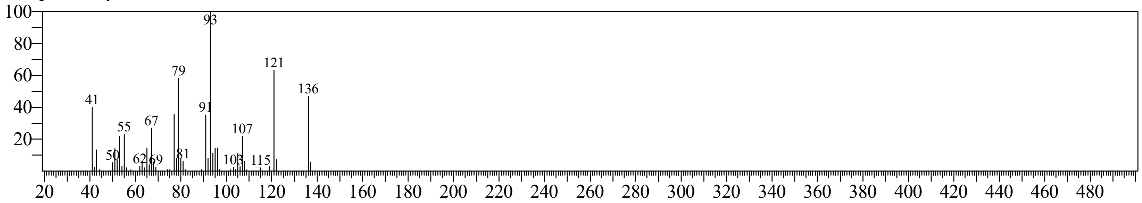

Qualitative Analysis Report

<< Target >>
Line#:12 R.Time:12.696(Scan#:2088) Retention Index:1023 MassPeaks:268
RawMode:Averaged 12.692-12.700(2087-2089) BasePeak:119.10(82810)
BG Mode:Calc. from Peak Group 1 - Event 1 Scan

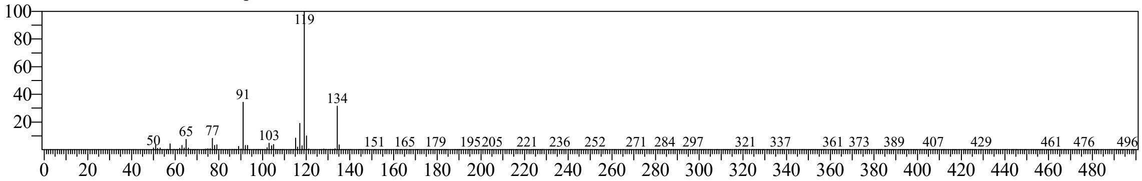

Hit#:1 Entry:317 Library:FFNSC 3.lib
SI:98 Formula:C10 H14 CAS:99-87-6 MolWeight:134 RetIndex:1025
CompName:Cymene <para->

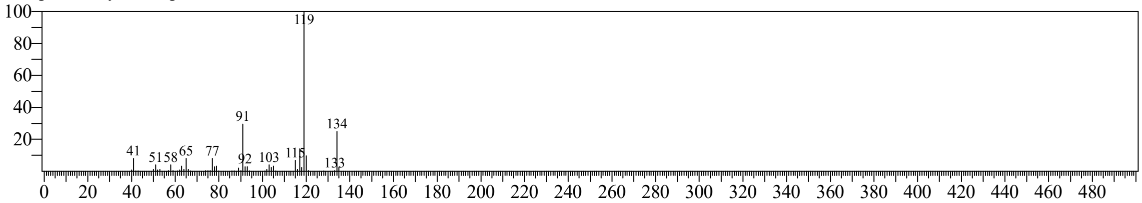

Hit#:2 Entry:5389 Library:NIST17-1.lib
SI:91 Formula:C7H5NO CAS:103-71-9 MolWeight:119 RetIndex:0
CompName:Benzene, isocyanato- $$ Isocyanic acid, phenyl ester $$ Carbanil $$ Isocyanatobenzene $$ Mondur P $$ Phenyl carbonimide $$ Phenyl isocyan

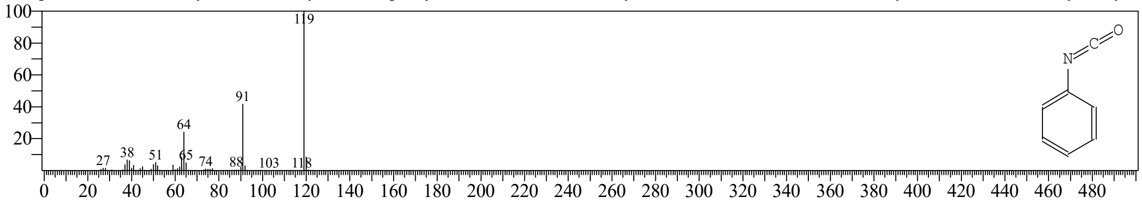

Hit#:3 Entry:1735 Library:FFNSC 3.lib
SI:91 Formula:C10 H14 CAS:527-84-4 MolWeight:134 RetIndex:1024
CompName:Cymene <ortho->

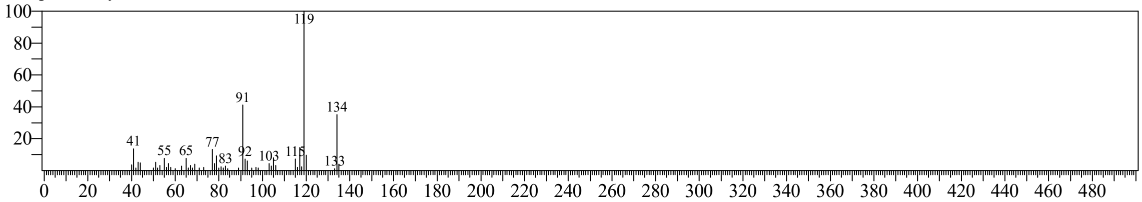

Hit#:4 Entry:32530 Library:NIST17-1.lib
SI:90 Formula:C10H11NS CAS:206559-59-3 MolWeight:177 RetIndex:0
CompName:3,4-Dimethylbenzyl isothiocyanate $$ 4-(Isothiocyanatomethyl)-1,2-dimethylbenzene # $$

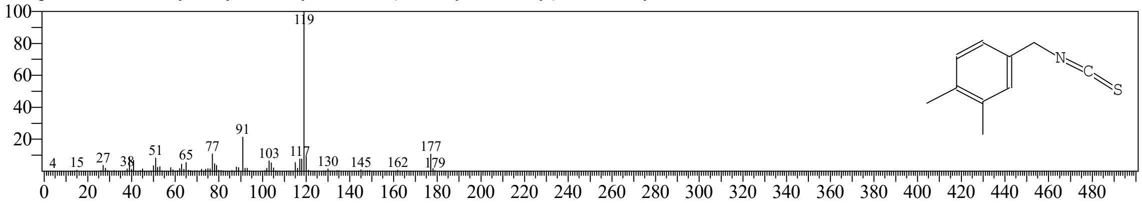

Hit#:5 Entry:242784 Library:NIST17-1.lib
SI:89 Formula:C19H20BrNO4S CAS:0-00-0 MolWeight:437 RetIndex:0
CompName:(S)-(7-Bromo-3-isopropyl-1,1-dioxido-3,4-dihydro-2H-benzo[b][1,4,5]oxathiazepin-2-yl)(m-tolyl)methanone $$ SMR001788266 $$ MLS0027

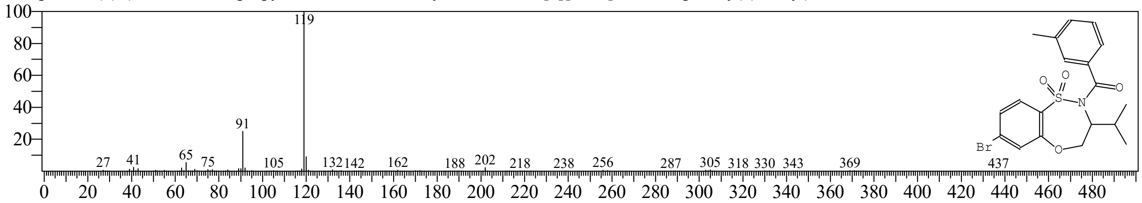

Qualitative Analysis Report

<< Target >>
Line#:13 R.Time:12.929(Scan#:2144) Retention Index:1028 MassPeaks:253
RawMode:Averaged 12.925-12.933(2143-2145) BasePeak:68.10(67223)
BG Mode:Calc. from Peak Group 1 - Event 1 Scan

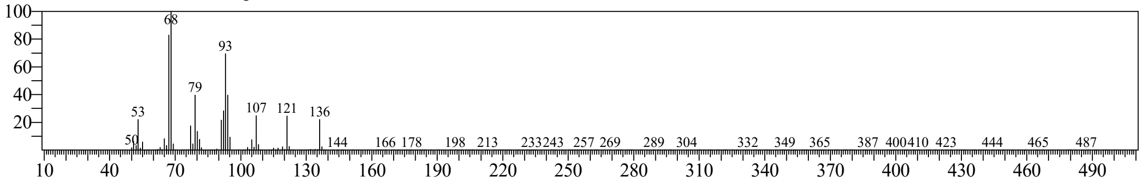

Hit#:1 Entry:980 Library:NIST17-1.lib
SI:100 Formula:F3NO CAS:13847-65-9 MolWeight:87 RetIndex:0
CompName:Trifluoroamine oxide $$ Nitrogen fluoride oxide (NF3O) $$ AMOX $$ Nitrogen Fluoride oxide (F3NO) $$ Trifluoroamine oxide $$ NOF3 $$ N

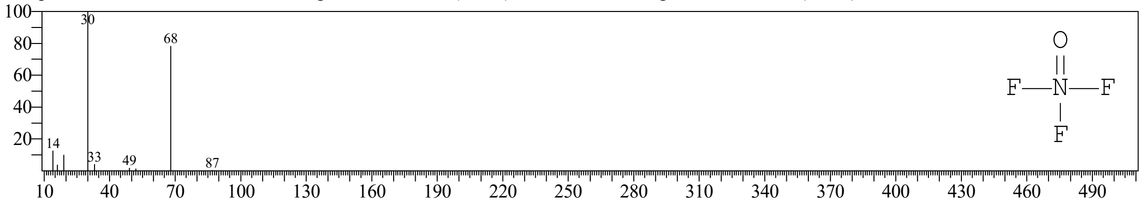

Hit#:2 Entry:443 Library:FFNSC 3.lib
SI:96 Formula:C10 H16 CAS:138-86-3 MolWeight:136 RetIndex:1030
CompName:Limonene

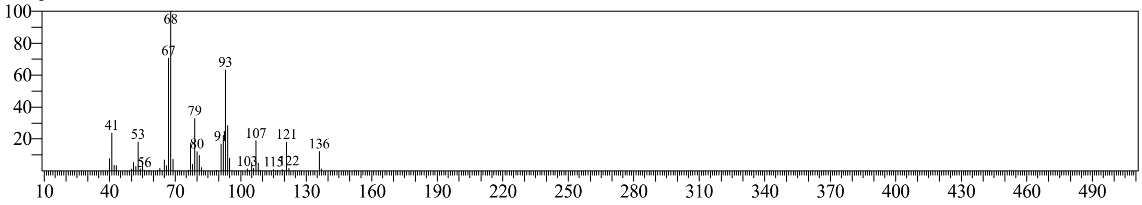

Hit#:3 Entry:206 Library:NIST17-1.lib
SI:94 Formula:C3O2 CAS:504-64-3 MolWeight:68 RetIndex:0
CompName:1,2-Propadiene-1,3-dione $$ Carbon suboxide $$ Carbon oxide (C3O2) $$ C3O2 $$ 1,3-Allenedione # $$

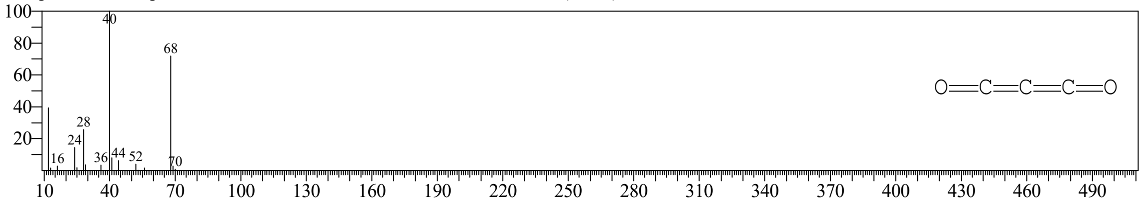

Hit#:4 Entry:10326 Library:NIST17-1.lib
SI:92 Formula:C10H16 CAS:5989-27-5 MolWeight:136 RetIndex:1018
CompName:D-Limonene $$ Cyclohexene, 1-methyl-4-(1-methylethenyl)-, (R)- $$ p-Mentha-1,8-diene, (R)-(+)- $$ (+)-(R)-Limonene $$ (+)-(4R)-Limonene

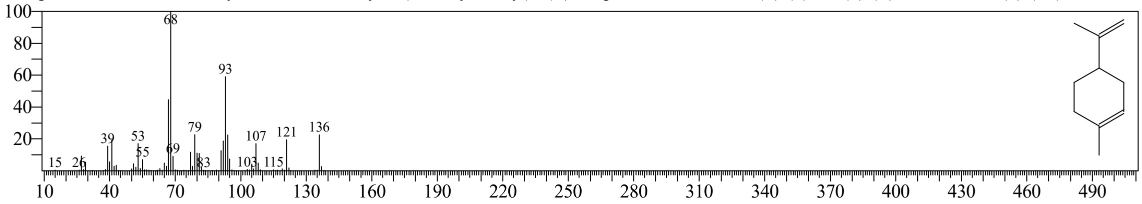

Hit#:5 Entry:8816 Library:NIST17-1.lib
SI:91 Formula:C5H8O2S CAS:1193-10-8 MolWeight:132 RetIndex:0
CompName:2,5-Dihydro-3-methyl-thiophene 1,1-dioxide $$ 3-Methylsulfolene $$ Thiophene, 2,5-dihydro-3-methyl-, 1,1-dioxide $$ 1,3-Butadiene, 2-methy

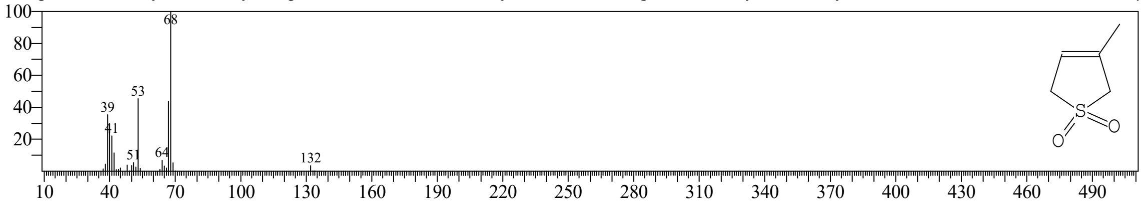

Qualitative Analysis Report

<< Target >>
Line#:14 R.Time:13.063(Scan#:2176) Retention Index:1031 MassPeaks:250
RawMode:Averaged 13.058-13.067(2175-2177) BasePeak:81.10(299679)
BG Mode:Calc. from Peak Group 1 - Event 1 Scan

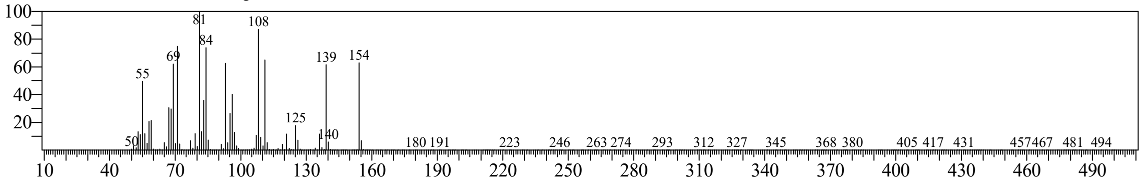

Hit#:1 Entry:621 Library:FFNSC 3.lib
SI:96 Formula:C10 H18 O CAS:470-82-6 MolWeight:154 RetIndex:1032
CompName:Eucalyptol

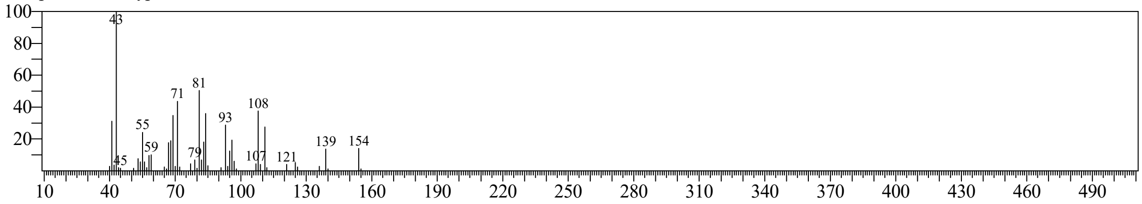

Hit#:2 Entry:6389 Library:NIST17-1.lib
SI:86 Formula:C9H16 CAS:3618-18-6 MolWeight:124 RetIndex:1026
CompName:Methylenecyclooctane $$ Cyclooctane, methylene- $$

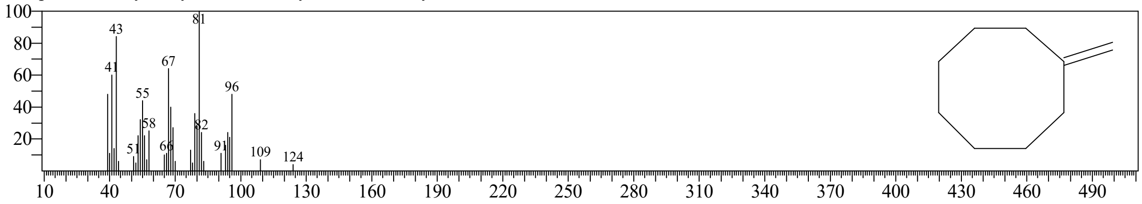

Hit#:3 Entry:158522 Library:NIST17-1.lib
SI:85 Formula:C11H5N7O5 CAS:0-00-0 MolWeight:315 RetIndex:0
CompName:12-(Furan-2-ylmethyl)-4,9,15-trioxa-3,5,8,10,12,14,16-heptaazatetracyclo[11.3.0.0(2,6).0(7,11)]hexadeca-1(16),2,5,7,10,13-hexaen-3-one

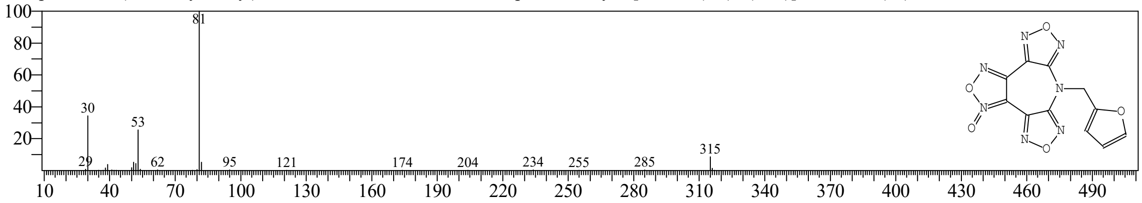

Hit#:4 Entry:1380 Library:NIST17-1.lib
SI:85 Formula:C2H6FOP CAS:753-70-8 MolWeight:96 RetIndex:0
CompName:Dimethylphosphinic fluoride

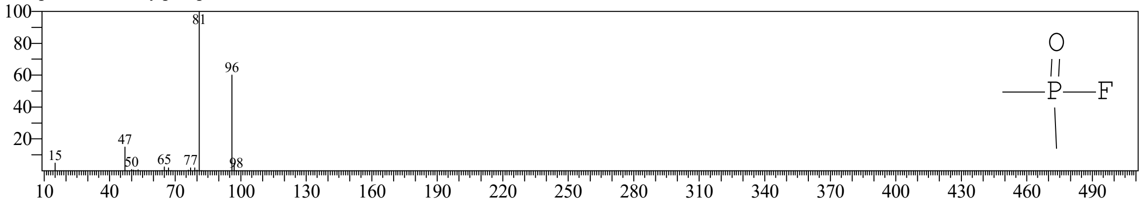

Hit#:5 Entry:11988 Library:NIST17-1.lib
SI:84 Formula:C9H16O CAS:0-00-0 MolWeight:140 RetIndex:1031
CompName:7-Methyl-8-oxabicyclo[4.3.0]nonane $$ 1-Methyloctahydro-2-benzofuran # $$

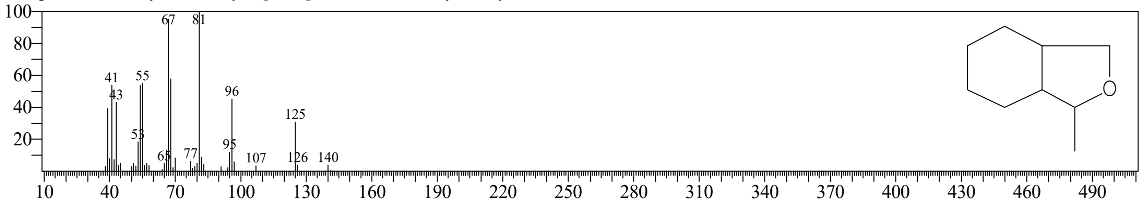

Qualitative Analysis Report

<< Target >>
Line#:15 R.Time:14.196(Scan#:2448) Retention Index:1057 MassPeaks:266
RawMode:Averaged 14.192-14.200(2447-2449) BasePeak:93.10(40359)
BG Mode:Calc. from Peak Group 1 - Event 1 Scan

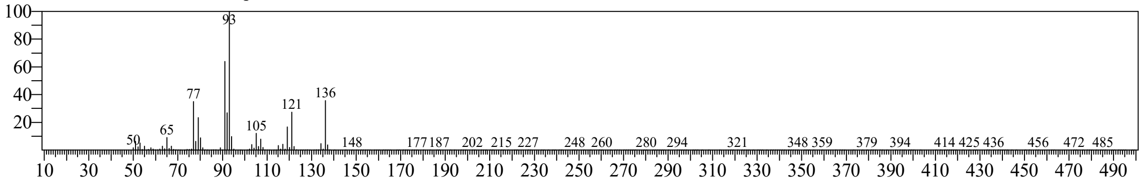

Hit#:1 Entry:818 Library:FFNSC 3.lib
SI:95 Formula:C10 H16 CAS:99-85-4 MolWeight:136 RetIndex:1058
CompName:Terpinene <gamma->

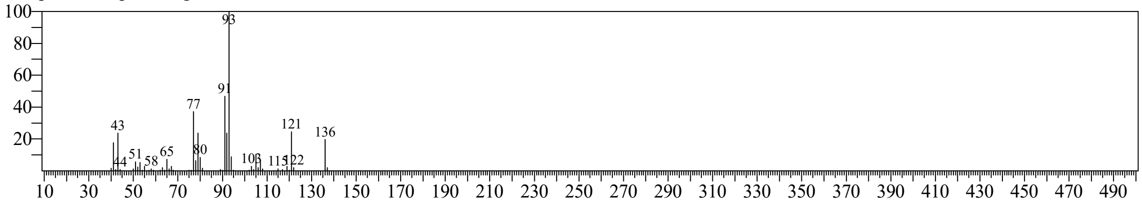

Hit#:2 Entry:10127 Library:NIST17-1.lib
SI:91 Formula:C8H12N2 CAS:109746-10-3 MolWeight:136 RetIndex:0
CompName:1,4-Methano-1H-Cyclopropa[d]pyridazine, 4,4a,5,5a-tetrahydro-6,6-dimethyl-, (1.alpha.,4.alpha.,4a.alpha.,5a.alpha.)-

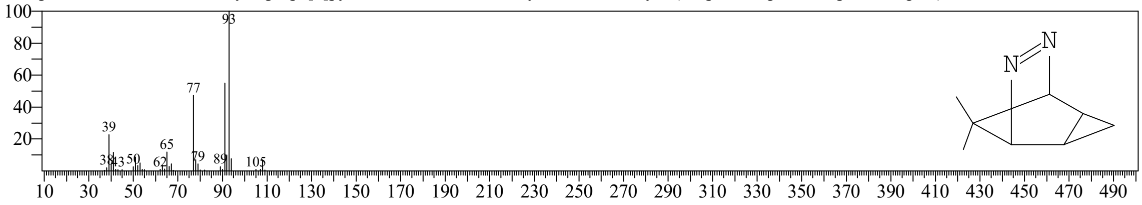

Hit#:3 Entry:10404 Library:NIST17-1.lib
SI:89 Formula:C10H16 CAS:586-62-9 MolWeight:136 RetIndex:1052
CompName:Cyclohexene, 1-methyl-4-(1-methylethylidene)- $$ p-Mentha-1,4(8)-diene $$ Terpinolene $$ Terpinolen $$ UN 2541 $$ .alpha.- Terpinolen $$

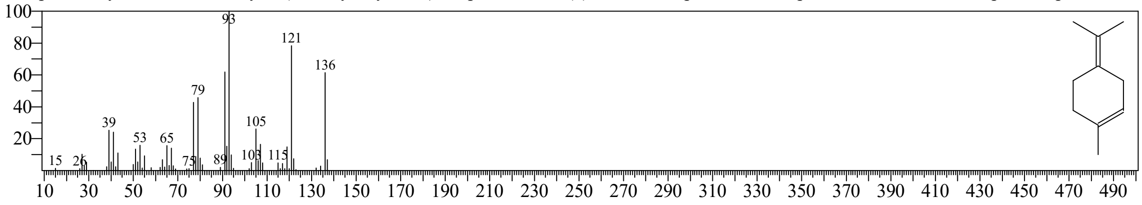

Hit#:4 Entry:10219 Library:NIST17-1.lib
SI:89 Formula:C9H12O CAS:29765-85-3 MolWeight:136 RetIndex:1060
CompName:2-Cyclopenten-1-one, 2,3,5-trimethyl-4-methylene- $$ 2,3,5-Trimethyl-4-methylene-2-cyclopenten-1-one # $$

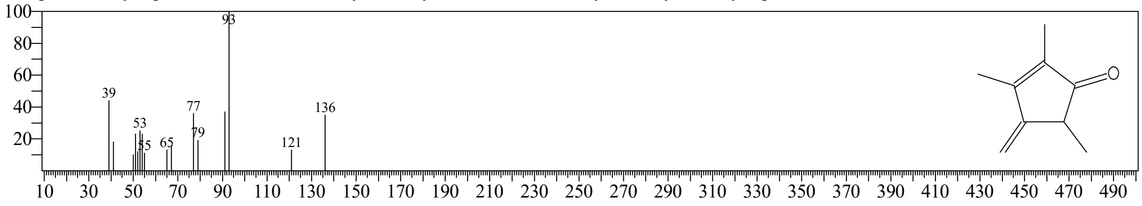

Hit#:5 Entry:24214 Library:NIST17-1.lib
SI:87 Formula:C10H16N2 CAS:87143-58-6 MolWeight:164 RetIndex:0
CompName:3,5-Methanocyclopentapyrazole, 3,3a,4,5,6,6a-hexahydro-3a,4,4-trimethyl-

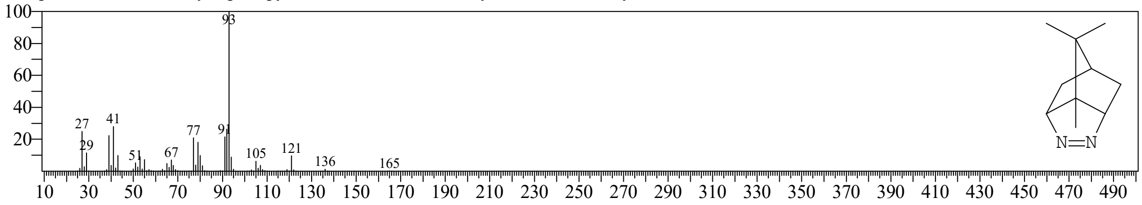

Qualitative Analysis Report

<< Target >>
Line#:16 R.Time:14.738(Scan#:2578) Retention Index:1069 MassPeaks:248
RawMode:Averaged 14.733-14.742(2577-2579) BasePeak:93.10(932)
BG Mode:Calc. from Peak Group 1 - Event 1 Scan

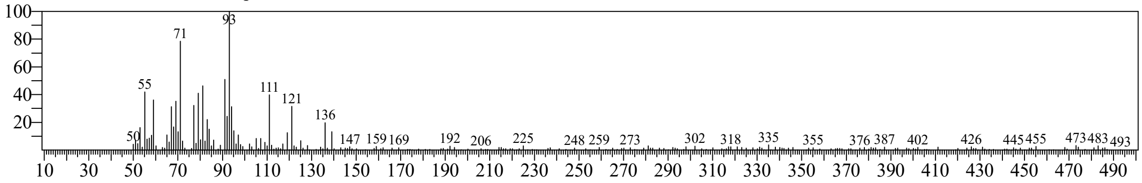

Hit#:1 Entry:953 Library:FFNSC 3.lib
SI:93 Formula:C10 H18 O CAS:15537-55-0 MolWeight:154 RetIndex:1069
CompName:Sabinene hydrate <cis->

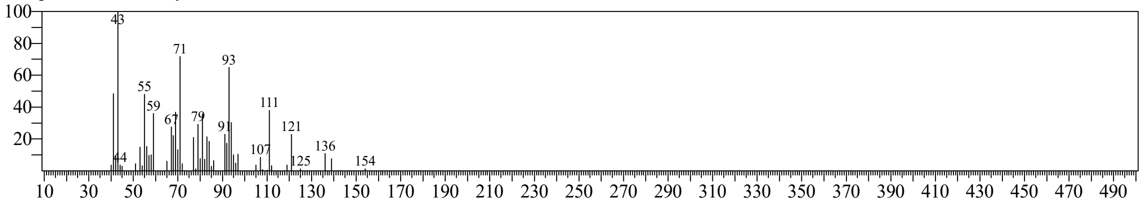

Hit#:2 Entry:5613 Library:NIST17-1.lib
SI:89 Formula:BrN3 CAS:13973-87-0 MolWeight:121 RetIndex:0
CompName:Bromine azide $$ Bromine azide (Br(N3)) $$ Bromine nitride $$ Nitrogen bromide (N3Br) $$

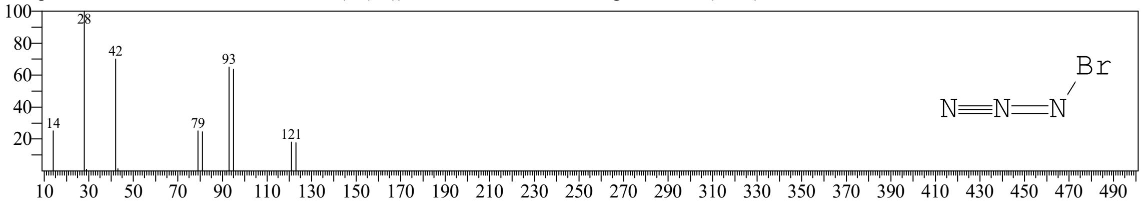

Hit#:3 Entry:10219 Library:NIST17-1.lib
SI:88 Formula:C9H12O CAS:29765-85-3 MolWeight:136 RetIndex:1060
CompName:2-Cyclopenten-1-one, 2,3,5-trimethyl-4-methylene- $$ 2,3,5-Trimethyl-4-methylene-2-cyclopenten-1-one # $$

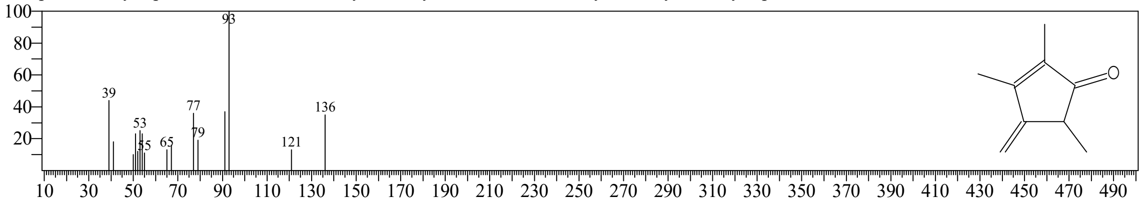

Hit#:4 Entry:2939 Library:NIST17-1.lib
SI:87 Formula:C6H8Si CAS:63878-65-9 MolWeight:108 RetIndex:0
CompName:Silabenzene,1-methyl- $$ 1-Methyl-1-silabenzene $$ 1-Methylsiline # $$

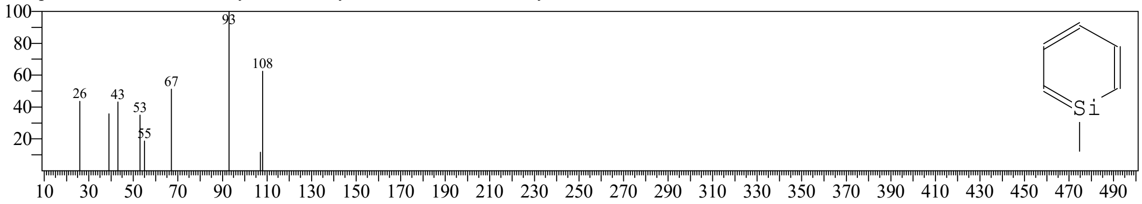

Hit#:5 Entry:18854 Library:NIST17-1.lib
SI:87 Formula:C10H18O CAS:7712-79-0 MolWeight:154 RetIndex:1079
CompName:(1S,3S,4S,5R)-1-Isopropyl-4-methylbicyclo[3.1.0]hexan-3-ol $$ Bicyclo[3.1.0]hexan-3-ol, 4-methyl-1-(1-methylethyl)-, (1S,3S,4S,5R)- $$ 3-T

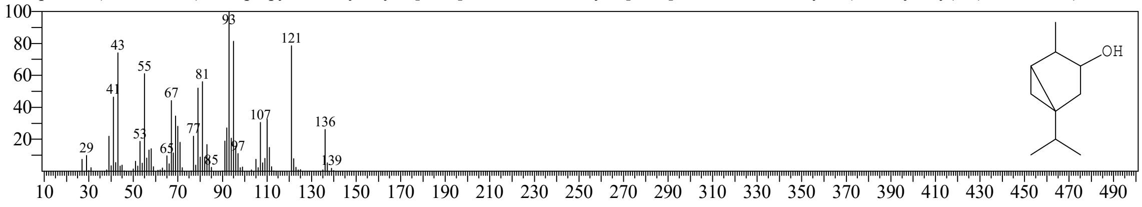

Qualitative Analysis Report

<< Target >>
Line#:17 R.Time:15.442(Scan#:2747) Retention Index:1084 MassPeaks:273
RawMode:Averaged 15.438-15.446(2746-2748) BasePeak:93.10(23223)
BG Mode:Calc. from Peak Group 1 - Event 1 Scan

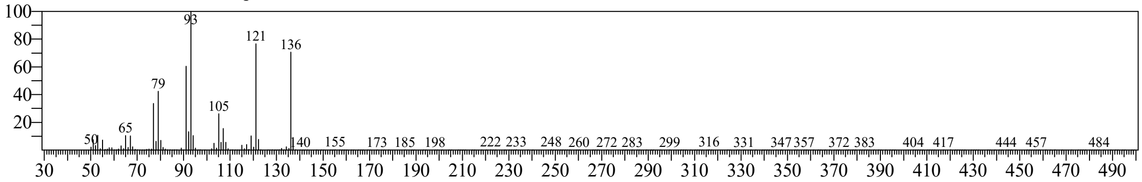

Hit#:1 Entry:603 Library:FFNSC 3.lib
SI:97 Formula:C10 H16 CAS:586-62-9 MolWeight:136 RetIndex:1086
CompName:Terpinolene

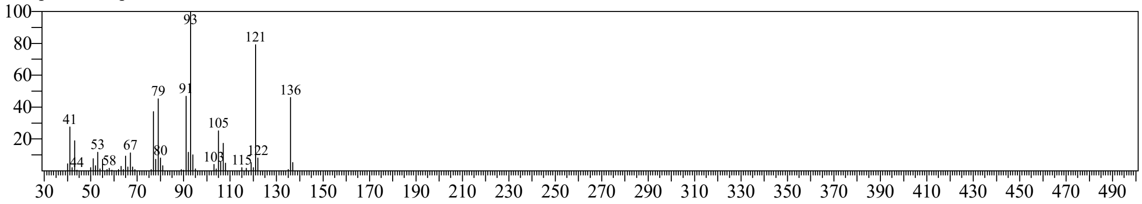

Hit#:2 Entry:10127 Library:NIST17-1.lib
SI:90 Formula:C8H12N2 CAS:109746-10-3 MolWeight:136 RetIndex:0
CompName:1,4-Methano-1H-Cyclopropa[d]pyridazine, 4,4a,5,5a-tetrahydro-6,6-dimethyl-, (1.alpha.,4.alpha.,4a.alpha.,5a.alpha.)-

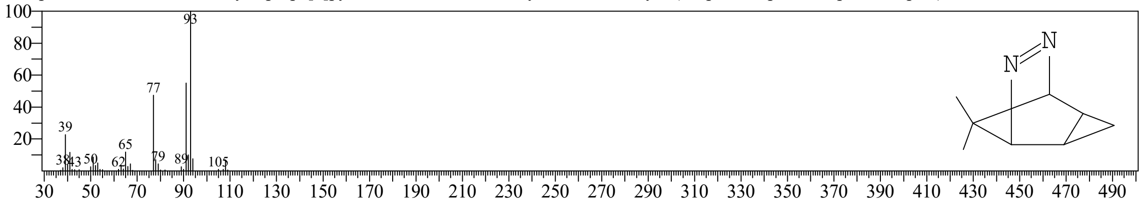

Hit#:3 Entry:24215 Library:NIST17-1.lib
SI:89 Formula:C10H16N2 CAS:0-00-0 MolWeight:164 RetIndex:0
CompName:2,3-Diazabicyclo[2.2.1]hept-2-ene, 5-ethenyl-4,7,7-trimethyl-, (1.alpha.,4.alpha.,5.beta.)- $$ 1,7,7-Trimethyl-6-vinyl-2,3-diazabicyclo[2.2.1]hep

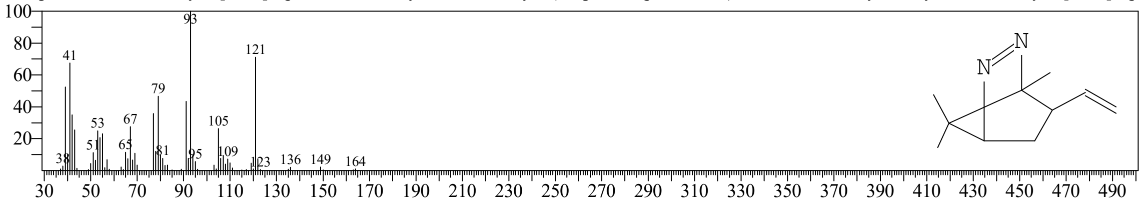

Hit#:4 Entry:10440 Library:NIST17-1.lib
SI:87 Formula:C10H16 CAS:14803-30-6 MolWeight:136 RetIndex:0
CompName:Cyclopropane, trimethyl(2-methyl-1-propenylidene)- $$ Propene, 2-methyl-1-(trimethylcyclopropylidene)- $$ 1,1,2-Trimethyl-3-(2-methyl-1-pro

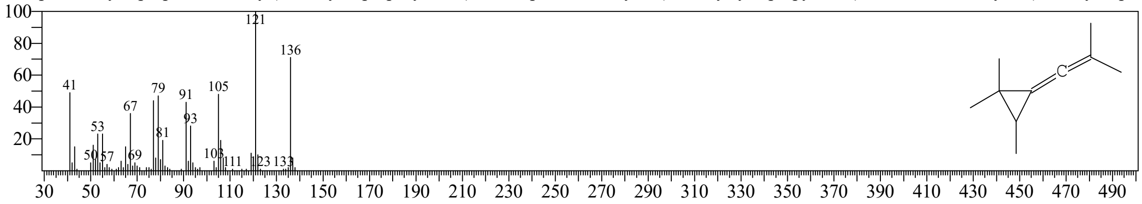

Hit#:5 Entry:71888 Library:NIST17-1.lib
SI:86 Formula:C12H18O2S CAS:0-00-0 MolWeight:226 RetIndex:0
CompName:3,4-Dimethyl-2,5-diprop-2-enyl-2,5-dihydrothiophene 1,1-dioxide

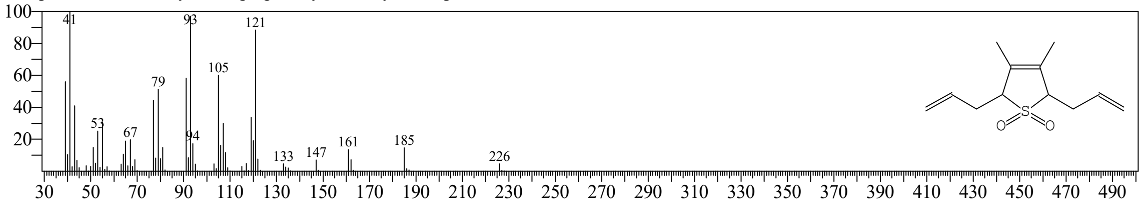

Qualitative Analysis Report

<< Target >>
Line#:18 R.Time:15.613(Scan#:2788) Retention Index:1088 MassPeaks:297
RawMode:Averaged 15.608-15.617(2787-2789) BasePeak:117.10(2589)
BG Mode:Calc. from Peak Group 1 - Event 1 Scan

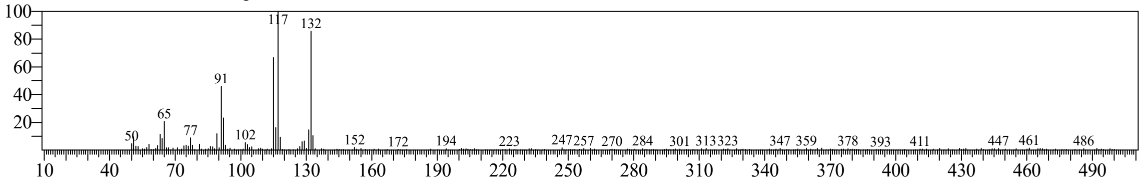

Hit#:1 Entry:77 Library:FFNSC 3.lib
SI:96 Formula:C10 H12 CAS:1195-32-0 MolWeight:132 RetIndex:1093
CompName:Cymenene <para->

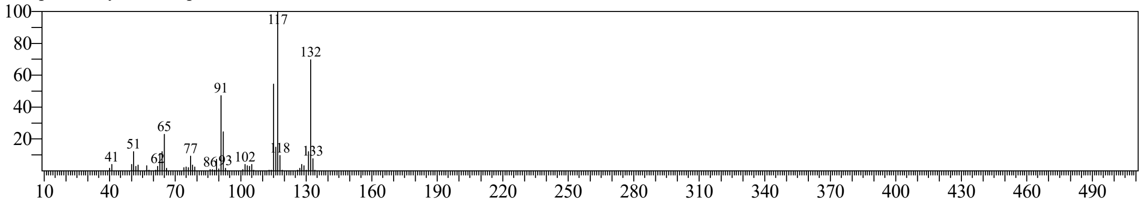

Hit#:2 Entry:2154 Library:FFNSC 3.lib
SI:94 Formula:C10 H12 CAS:1124-20-5 MolWeight:132 RetIndex:1080
CompName:Cymenene <meta->

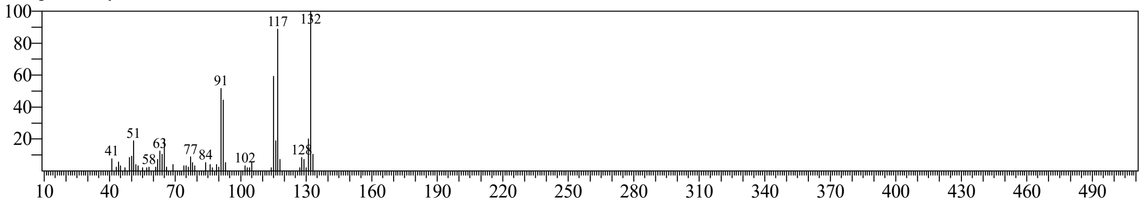

Hit#:3 Entry:117396 Library:NIST17-1.lib
SI:92 Formula:C15H14Se CAS:72065-43-1 MolWeight:274 RetIndex:0
CompName:Bicyclo[4.2.1]nona-2,4,7-triene, 9-(phenylseleno)-, syn- $$ Bicyclo[4.2.1]nona-2,4,7-trien-9-yl phenyl selenide # $$

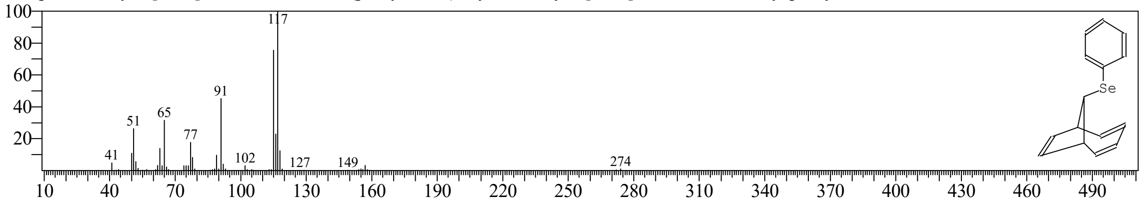

Hit#:4 Entry:9104 Library:NIST17-1.lib
SI:91 Formula:C10H12 CAS:3333-13-9 MolWeight:132 RetIndex:1096
CompName:Benzene, 1-methyl-4-(2-propenyl)- $$ p-Allyltoluene $$ p-Methylallylbenzene $$ Toluene, p-allyl- $$ 1-Allyl-4-methylbenzene $$ 3-p-Tolylpro

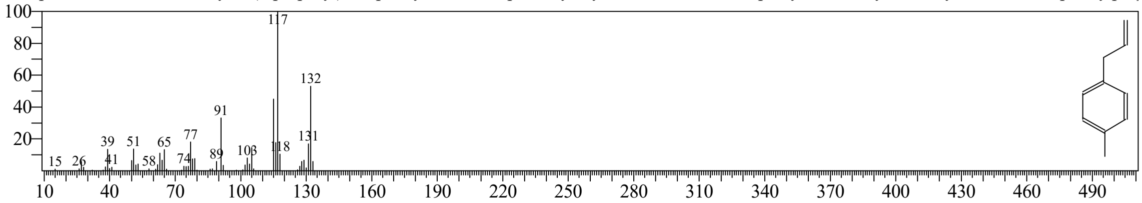

Hit#:5 Entry:117397 Library:NIST17-1.lib
SI:91 Formula:C15H14Se CAS:72065-50-0 MolWeight:274 RetIndex:0
CompName:Bicyclo[4.2.1]nona-2,4,7-triene, 9-(phenylseleno)-, (anti-) $$ Bicyclo[4.2.1]nona-2,4,7-trien-9-yl phenyl selenide # $$

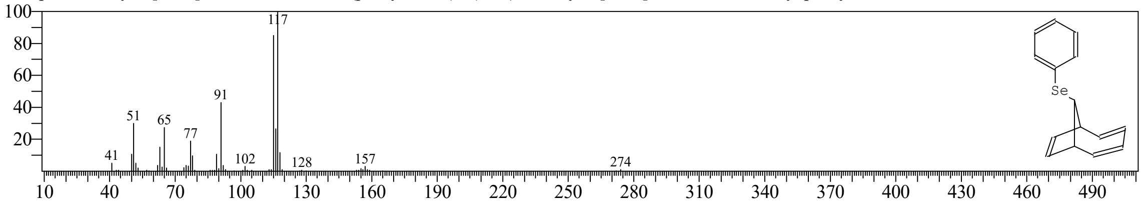

Qualitative Analysis Report

<< Target >>
Line#:19 R.Time:16.042(Scan#:2891) Retention Index:1098 MassPeaks:292
RawMode:Averaged 16.038-16.046(2890-2892) BasePeak:71.10(84948)
BG Mode:Calc. from Peak Group 1 - Event 1 Scan

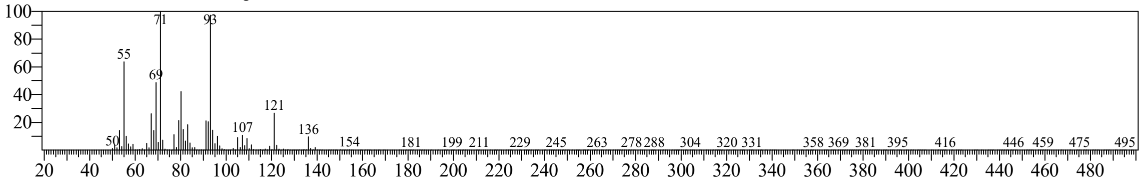

Hit#:1 Entry:3376 Library:FFNSC 3.lib
SI:97 Formula:C17 H23 N O2 CAS:7149-26-0 MolWeight:273 RetIndex:1104
CompName:Linalyl anthranilate

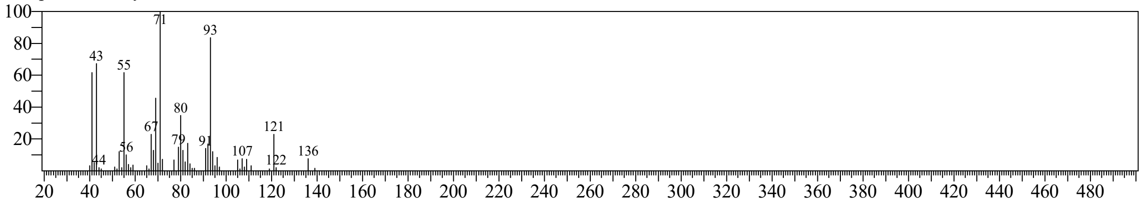

Hit#:2 Entry:365 Library:FFNSC 3.lib
SI:95 Formula:C10 H18 O CAS:78-70-6 MolWeight:154 RetIndex:1101
CompName:Linalool

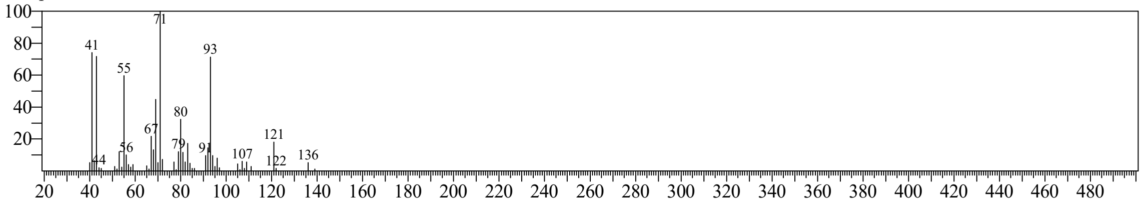

Hit#:3 Entry:954 Library:FFNSC 3.lib
SI:88 Formula:C10 H18 O CAS:17699-16-0 MolWeight:154 RetIndex:1099
CompName:Sabinene hydrate <trans->

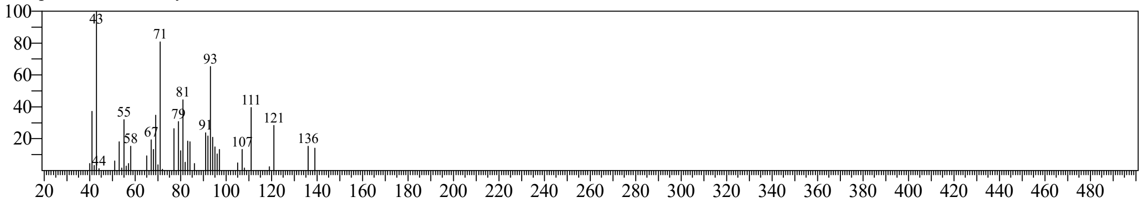

Hit#:4 Entry:12787 Library:NIST17-1.lib
SI:87 Formula:C8H14O2 CAS:5455-24-3 MolWeight:142 RetIndex:1088
CompName:4,5-Octanedione $$ n-Octane-4,5-dione $$ Bibutyryl $$ 4,5-Octadione $$

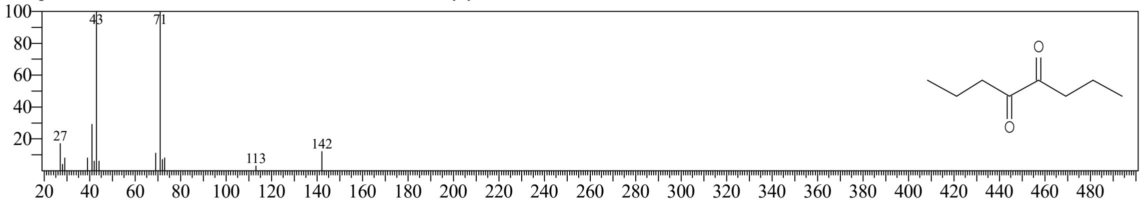

Hit#:5 Entry:18713 Library:NIST17-1.lib
SI:86 Formula:C10H18O CAS:4948-29-2 MolWeight:154 RetIndex:1088
CompName:trans-2-Pinanol $$ Bicyclo(3.1.1)heptan-2-ol, 2,6,6-trimethyl-, (1.alpha.,2.beta.,5.alpha.)- $$ Pinan-2.beta.-ol $$ 2-Pinanol, trans- $$ 2,6,6-Trim

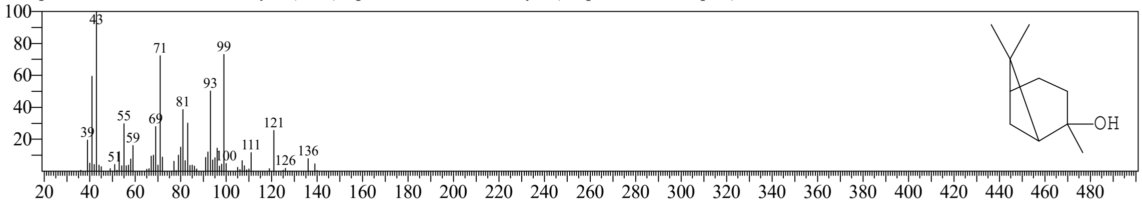

Qualitative Analysis Report

<< Target >>
Line#:20 R.Time:16.954(Scan#:3110) Retention Index:1117 MassPeaks:246
RawMode:Averaged 16.950-16.958(3109-3111) BasePeak:81.10(2047)
BG Mode:Calc. from Peak Group 1 - Event 1 Scan

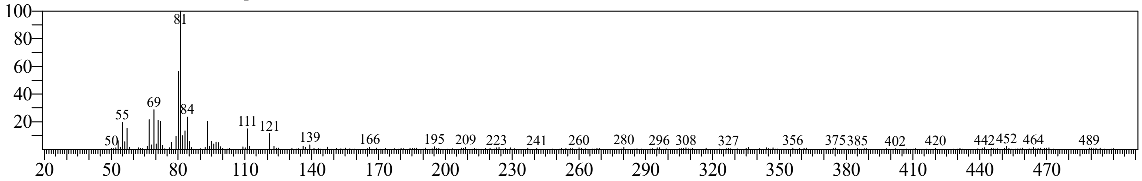

Hit#:1 Entry:909 Library:FFNSC 3.lib
SI:95 Formula:C10 H18 O CAS:1632-73-1 MolWeight:154 RetIndex:1123
CompName:Fenchyl alcohol

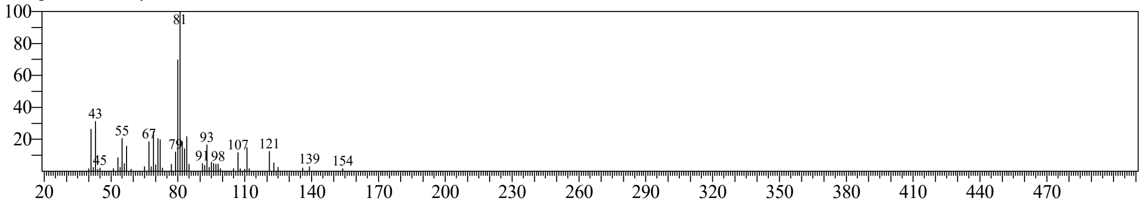

Hit#:2 Entry:19638 Library:NIST17-1.lib
SI:92 Formula:C8H12OS CAS:1883-78-9 MolWeight:156 RetIndex:1127
CompName:Furan, 2-[[(1-methylethyl)thio]methyl]- $$ Furan, 2-[(isopropylthio)methyl]- $$ Isopropyl furfuryl sulfide $$ 2-[[(1-Methylethyl)thio]methyl]fu

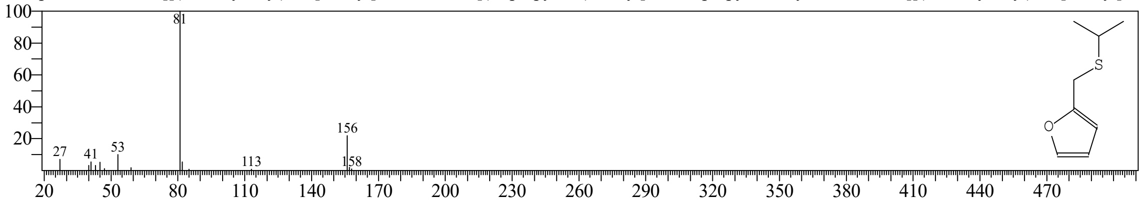

Hit#:3 Entry:2227 Library:FFNSC 3.lib
SI:92 Formula:C10 H18 O CAS:14575-74-7 MolWeight:154 RetIndex:1119
CompName:Fenchol <endo->

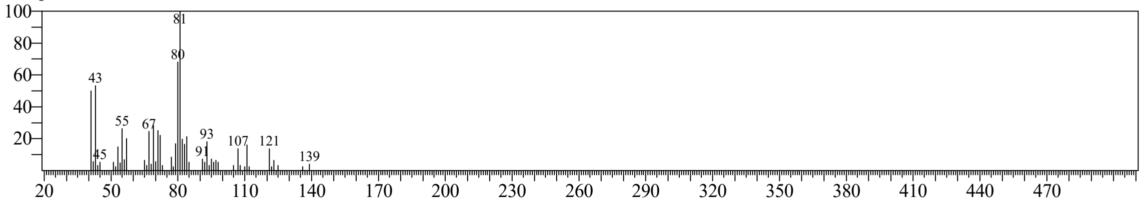

Hit#:4 Entry:2710 Library:FFNSC 3.lib
SI:91 Formula:C10 H18 O CAS:22627-95-8 MolWeight:154 RetIndex:1123
CompName:Fenchol <exo->

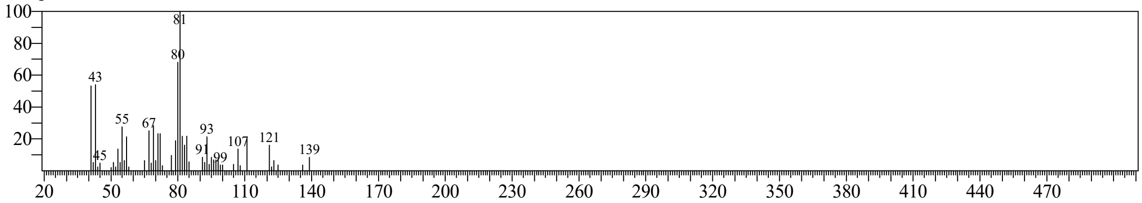

Hit#:5 Entry:186893 Library:NIST17-1.lib
SI:89 Formula:C13H13ClN2O5S CAS:4793-48-0 MolWeight:344 RetIndex:0
CompName:Furosemide, methyl ester $$ 2-Chloro-4-[(2-furylmethyl)amino]-5-(methoxycarbonyl)benzenesulfonimidic acid # $$

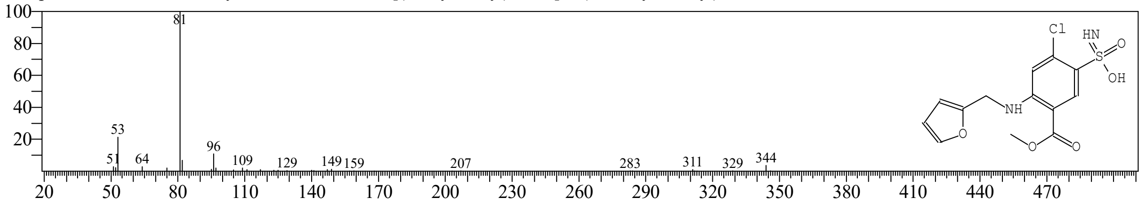

Qualitative Analysis Report

<< Target >>
Line#:21 R.Time:17.063(Scan#:3136) Retention Index:1120 MassPeaks:259
RawMode:Averaged 17.058-17.067(3135-3137) BasePeak:107.10(2511)
BG Mode:Calc. from Peak Group 1 - Event 1 Scan

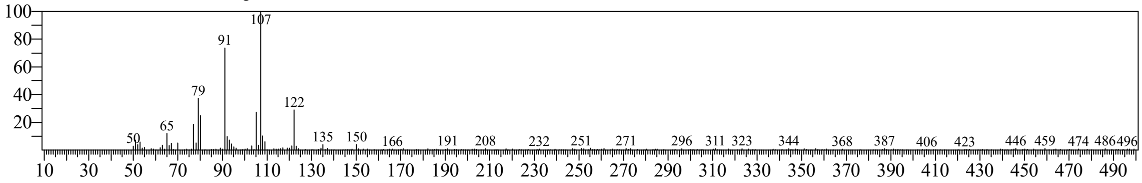

Hit#:1 Entry:7085 Library:NIST17-1.lib
SI:95 Formula:F5P CAS:7647-19-0 MolWeight:126 RetIndex:0
CompName:Phosphorus pentafluoride $$ Phosphorus(V) fluoride $$ PF5 $$ Phosphorane, pentafluoro- $$ UN 2198 $$ Phosphorus fluoride $$ Pentafluoro

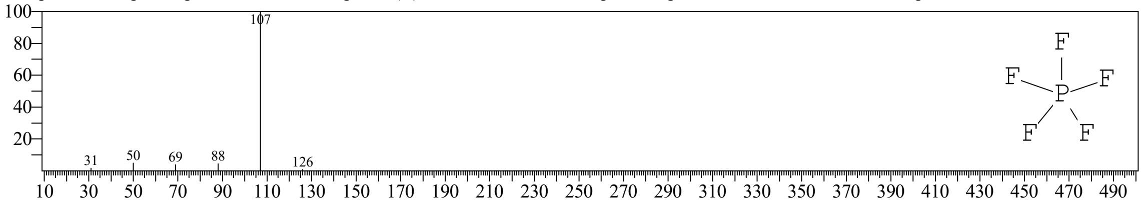

Hit#:2 Entry:1478 Library:FFNSC 3.lib
SI:93 Formula:C10 H14 O CAS:473-06-3 MolWeight:150 RetIndex:1122
CompName:Chrysanthenone

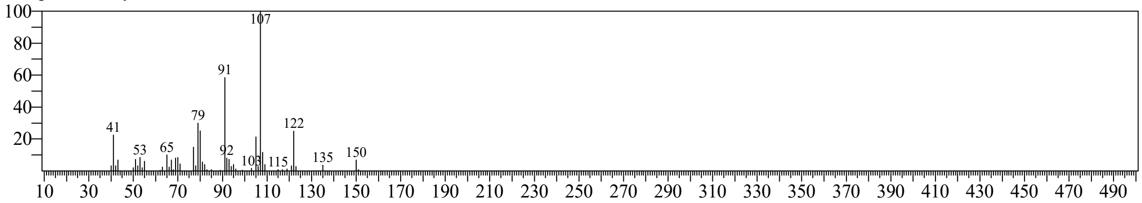

Hit#:3 Entry:9872 Library:NIST17-1.lib
SI:92 Formula:C2H5F4P CAS:0-00-0 MolWeight:136 RetIndex:0
CompName:Ethyltetrafluorophosphorane

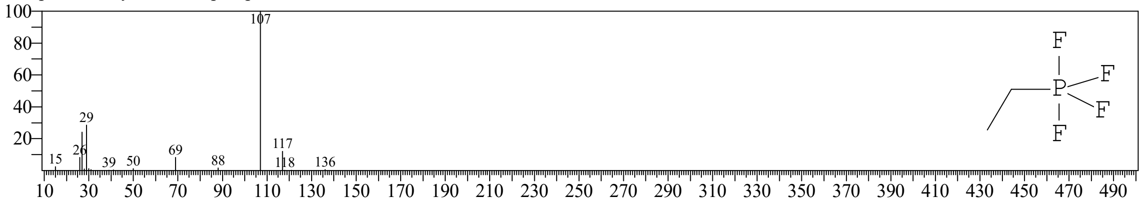

Hit#:4 Entry:9296 Library:NIST17-1.lib
SI:88 Formula:C2H3F4P CAS:3583-99-1 MolWeight:134 RetIndex:0
CompName:Tetrafluorovinylphosphorane

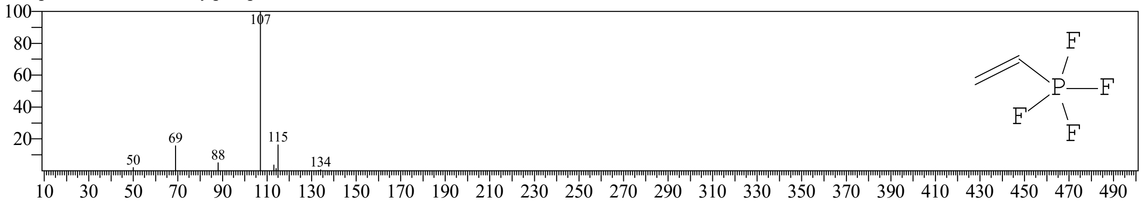

Hit#:5 Entry:120100 Library:NIST17-1.lib
SI:87 Formula:C14H16NO3P CAS:56830-89-8 MolWeight:277 RetIndex:0
CompName:Phosphoramidic acid, bis(4-methylphenyl) ester $$ Bis(4-methylphenyl) amidophosphate # $$

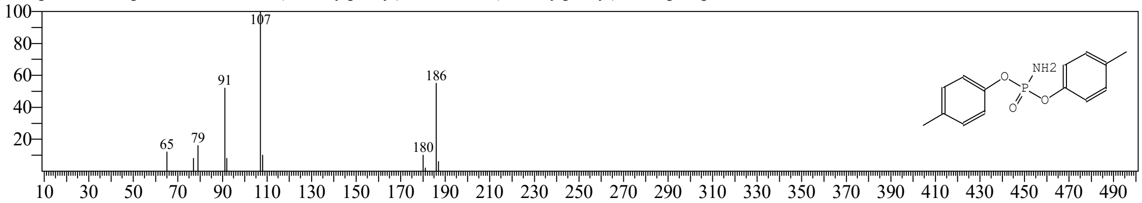

Qualitative Analysis Report

<< Target >>
Line#:22 R.Time:17.283(Scan#:3189) Retention Index:1124 MassPeaks:227
RawMode:Averaged 17.279-17.288(3188-3190) BasePeak:93.10(1499)
BG Mode:Calc. from Peak Group 1 - Event 1 Scan

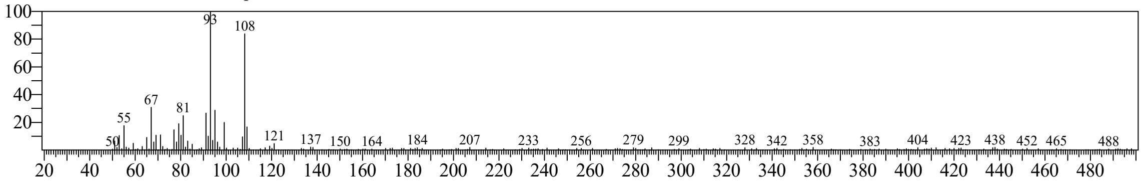

Hit#:1 Entry:2939 Library:NIST17-1.lib
SI:94 Formula:C6H8Si CAS:63878-65-9 MolWeight:108 RetIndex:0
CompName:Silabenzene,1-methyl- $$ 1-Methyl-1-silabenzene $$ 1-Methylsiline # $$

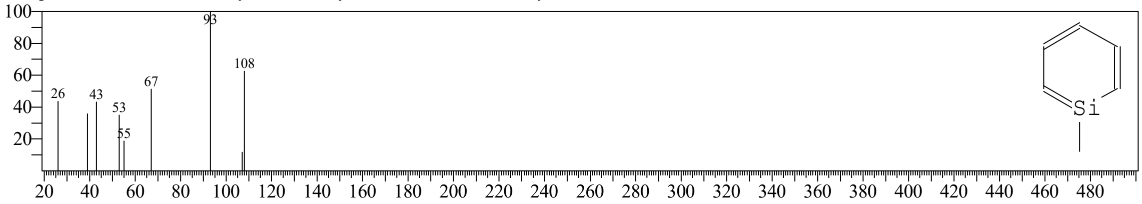

Hit#:2 Entry:2916 Library:FFNSC 3.lib
SI:94 Formula:C10 H16 O CAS:4501-58-0 MolWeight:152 RetIndex:1125
CompName:Campholenal<alpha->

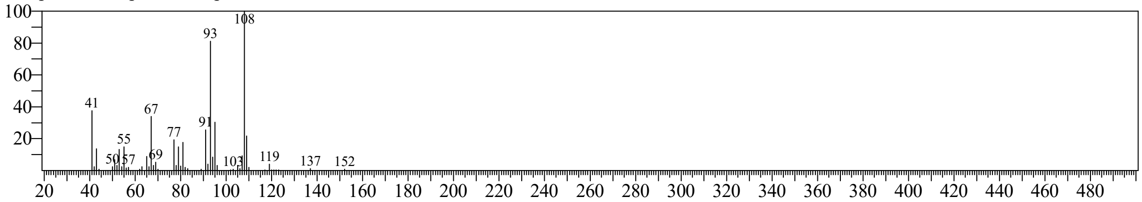

Hit#:3 Entry:1461 Library:FFNSC 3.lib
SI:92 Formula:C10 H16 O CAS:91819-58-8 MolWeight:152 RetIndex:1126
CompName:Campholenic aldehyde <alpha->

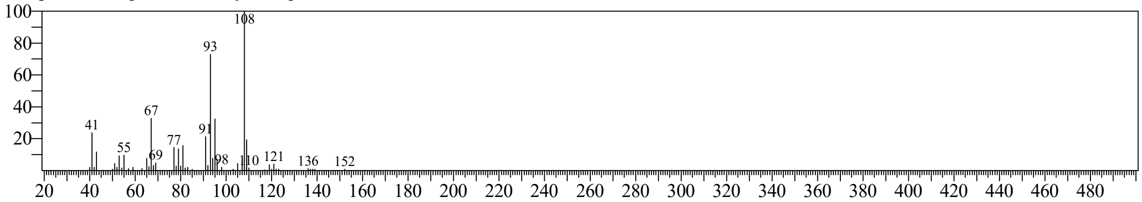

Hit#:4 Entry:17557 Library:NIST17-1.lib
SI:92 Formula:C10H16O CAS:3570-04-5 MolWeight:152 RetIndex:1131
CompName:Camphenol, 6- $$ 5,5-Dimethyl-6-methylenebicyclo[2.2.1]heptan-2-ol # $$ Camphen-6-ol $$ Camphenol $$ 6-Hydroxycamphene $$

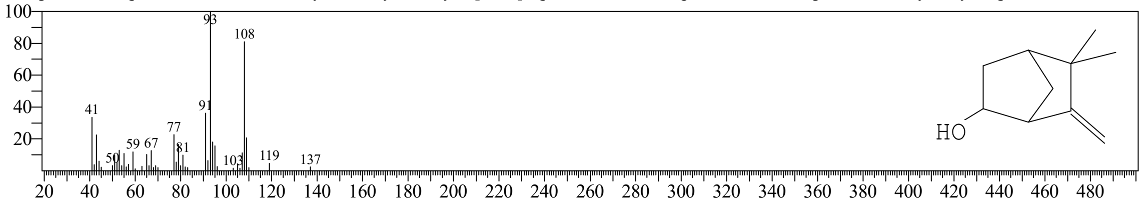

Hit#:5 Entry:17595 Library:NIST17-1.lib
SI:88 Formula:C10H16O CAS:0-00-0 MolWeight:152 RetIndex:1120
CompName:1,7,7-Trimethylbicyclo[2.2.1]hept-5-en-2-ol

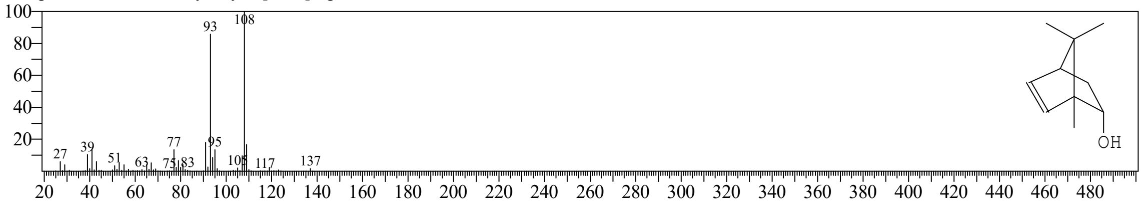

Qualitative Analysis Report

<< Target >>
Line#:23 R.Time:18.017(Scan#:3365) Retention Index:1140 MassPeaks:264
RawMode:Averaged 18.012-18.021(3364-3366) BasePeak:91.10(2340)
BG Mode:Calc. from Peak Group 1 - Event 1 Scan

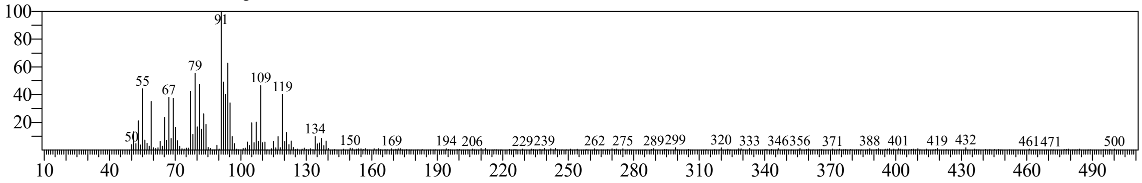

Hit#:1 Entry:231901 Library:NIST17-1.lib
SI:93 Formula:C13H14Fe2O4S2 CAS:12215-56-4 MolWeight:410 RetIndex:0
CompName:Iron, [(2,3,5,6-.eta.)-bicyclo[2.2.1]hepta-2,5-diene]tetracarbonylbis[.mu.-(methanethiolato)]di-, (Fe-Fe)

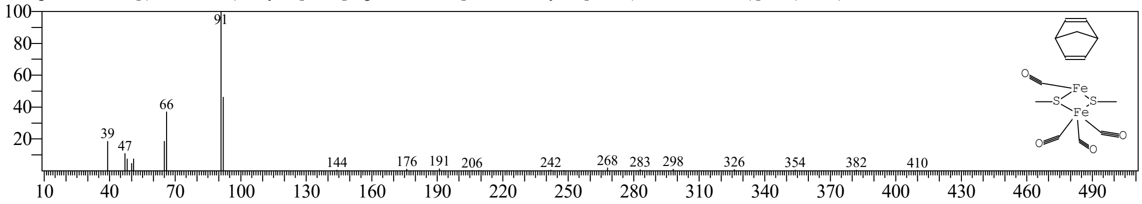

Hit#:2 Entry:17564 Library:NIST17-1.lib
SI:93 Formula:C10H16O CAS:13040-03-4 MolWeight:152 RetIndex:1136
CompName:(1R)-cis-Verbenol $$ Bicyclo[3.1.1]hept-3-en-2-ol, 4,6,6-trimethyl-, [1R-(1.alpha.,2.beta.,5.alpha.)]- $$ 2-Pinen-4-ol, (1R,4R,5R)-(+)- $$ (+)-ci

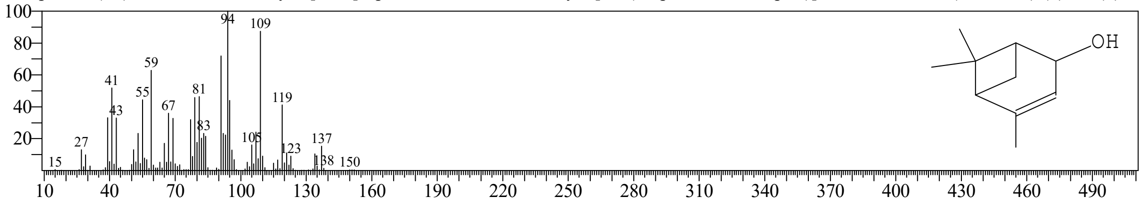

Hit#:3 Entry:17563 Library:NIST17-1.lib
SI:93 Formula:C10H16O CAS:1845-30-3 MolWeight:152 RetIndex:1136
CompName:cis-Verbenol $$ Bicyclo[3.1.1]hept-3-en-2-ol, 4,6,6-trimethyl-, (1.alpha.,2.beta.,5.alpha.)- $$ Bicyclo(3.1.1)hept-3-en-2-ol, 4,6,6-trimethyl-, (1R

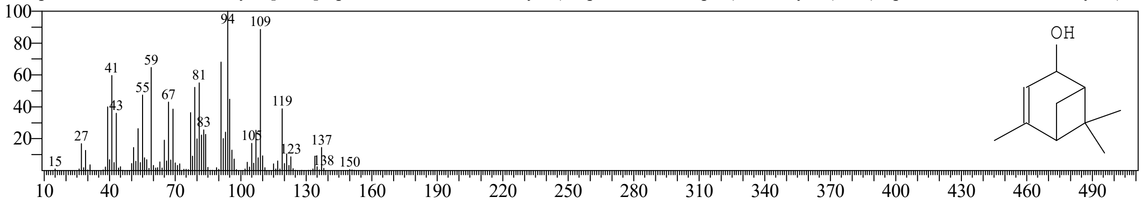

Hit#:4 Entry:17562 Library:NIST17-1.lib
SI:91 Formula:C10H16O CAS:18881-04-4 MolWeight:152 RetIndex:1136
CompName:Bicyclo[3.1.1]hept-3-en-2-ol, 4,6,6-trimethyl-, [1S-(1.alpha.,2.beta.,5.alpha.)]- $$ (S)-cis-Verbenol $$ (-)-(Z)-Verbenol $$ Bicyclo(3.1.1)hept-3-

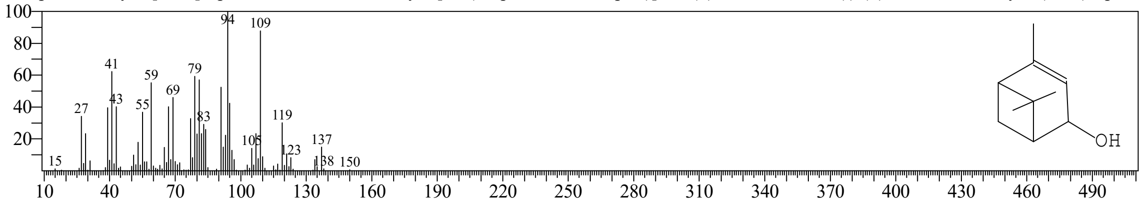

Hit#:5 Entry:1672 Library:FFNSC 3.lib
SI:91 Formula:C10 H16 O CAS:1820-09-3 MolWeight:152 RetIndex:1145
CompName:Verbenol <trans->

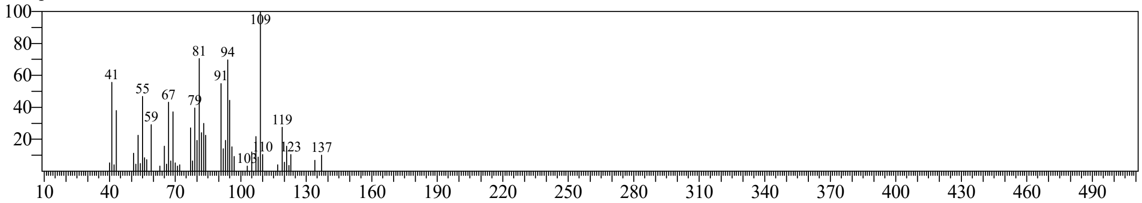

Qualitative Analysis Report

<< Target >>
Line#:24 R.Time:18.233(Scan#:3417) Retention Index:1145 MassPeaks:300
RawMode:Averaged 18.229-18.238(3416-3418) BasePeak:95.10(81888)
BG Mode:Calc. from Peak Group 1 - Event 1 Scan

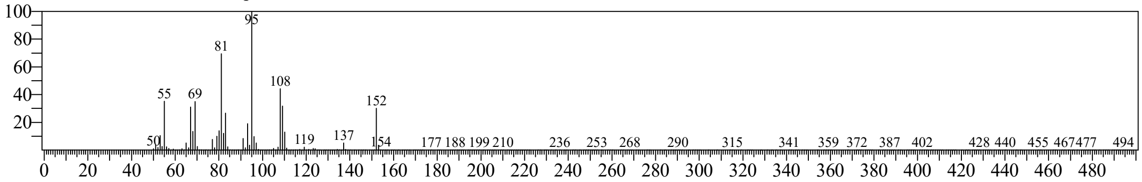

Hit#:1 Entry:298 Library:FFNSC 3.lib
SI:98 Formula:C10 H16 O CAS:76-22-2 MolWeight:152 RetIndex:1149
CompName:Camphor

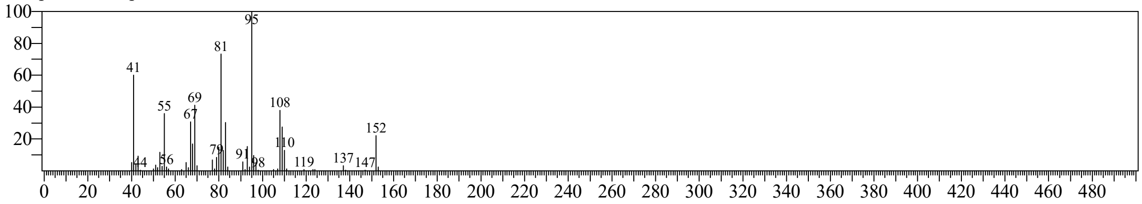

Hit#:2 Entry:30769 Library:NIST17-1.lib
SI:86 Formula:C10H19Cl CAS:80483-50-7 MolWeight:174 RetIndex:1143
CompName:2-Chloro-1-isopropyl-4-methylcyclohexane

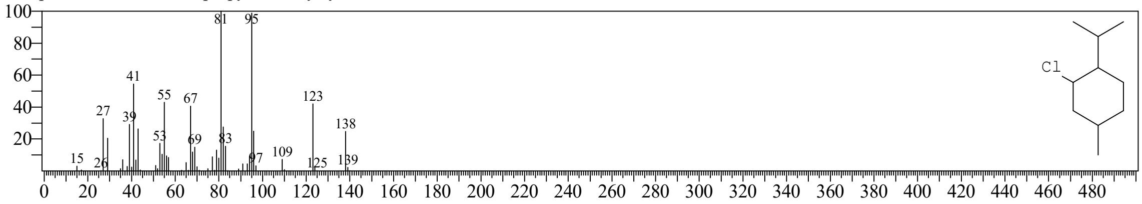

Hit#:3 Entry:34970 Library:NIST17-1.lib
SI:86 Formula:C12H20O CAS:19576-21-7 MolWeight:180 RetIndex:1155
CompName:1-Propanone, 1-[2-(1,1-dimethylethyl)-2-cyclopropen-1-yl]-2,2-dimethyl- $$ 1-Propanone, 1-(2-tert-butyl-2-cyclopropen-1-yl)-2,2-dimethyl- $$

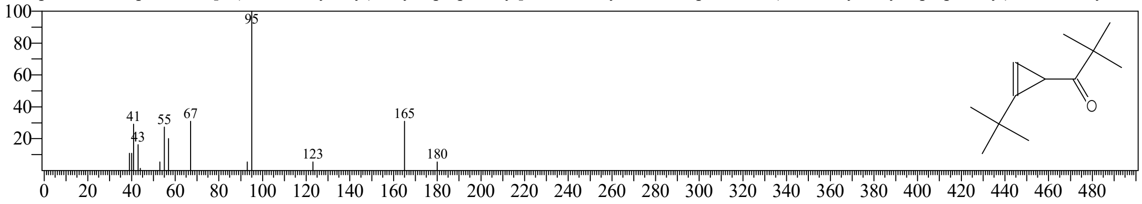

Hit#:4 Entry:17944 Library:NIST17-1.lib
SI:85 Formula:C8H11NS CAS:18530-33-1 MolWeight:153 RetIndex:0
CompName:Bicyclo[2.2.1]heptane, 2-isothiocyanato-, exo- $$ 2-Isothiocyanatobicyclo[2.2.1]heptane # $$

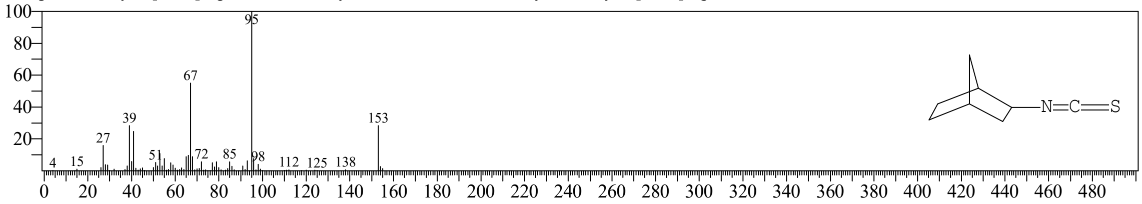

Hit#:5 Entry:11059 Library:NIST17-1.lib
SI:85 Formula:C9H14O CAS:697-37-0 MolWeight:138 RetIndex:1147
CompName:1-(1-Propynyl)cyclohexanol

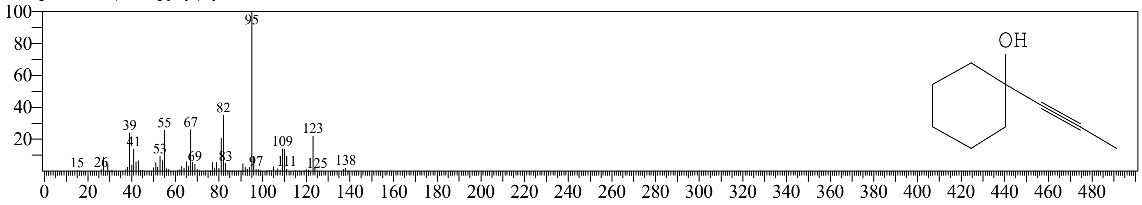

Qualitative Analysis Report

<< Target >>
Line#:25 R.Time:18.621(Scan#:3510) Retention Index:1153 MassPeaks:290
RawMode:Averaged 18.617-18.625(3509-3511) BasePeak:71.10(848)
BG Mode:Calc. from Peak Group 1 - Event 1 Scan

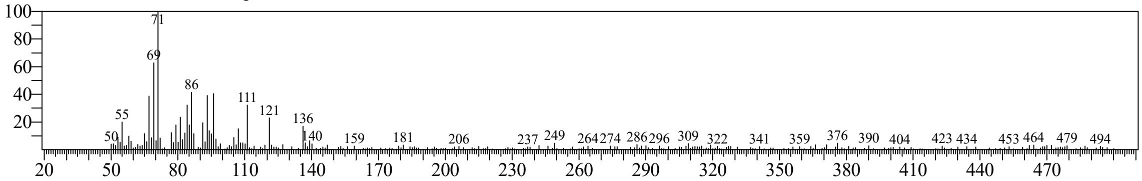

Hit#:1 Entry:689 Library:FFNSC 3.lib
SI:92 Formula:C10 H18 O CAS:465-31-6 MolWeight:154 RetIndex:1156
CompName:Camphene hydrate

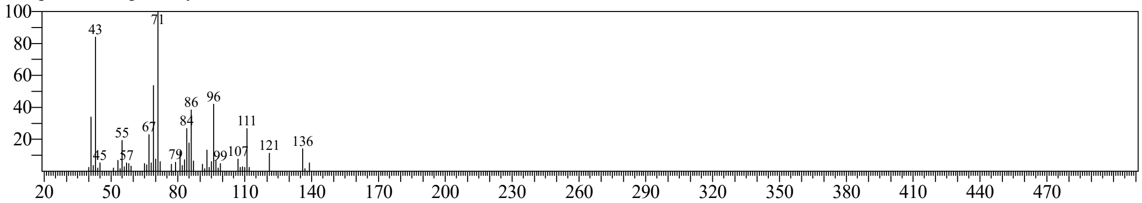

Hit#:2 Entry:18695 Library:NIST17-1.lib
SI:88 Formula:C10H18O CAS:7299-41-4 MolWeight:154 RetIndex:1158
CompName:Cyclohexanol, 1-methyl-4-(1-methylethenyl)-, cis- $$ Terpineol, cis-.beta.- $$ p-Menth-8-en-1-ol, cis $$ cis-.beta.-Terpineol $$ cis-p-Menth-8-e

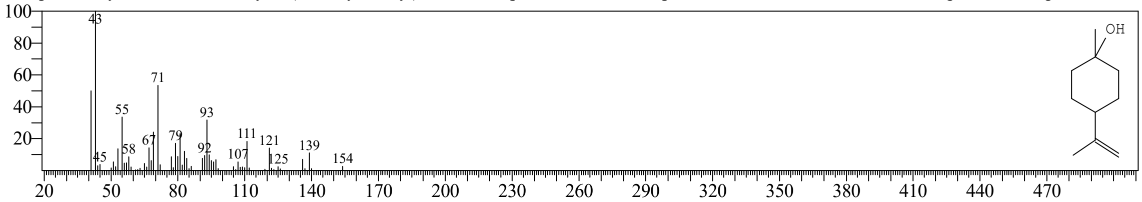

Hit#:3 Entry:18797 Library:NIST17-1.lib
SI:88 Formula:C10H18O CAS:7299-40-3 MolWeight:154 RetIndex:1158
CompName:p-Menth-8-en-1-ol, stereoisomer $$ Cyclohexanol, 1-methyl-4-(1-methylethenyl)-, trans- $$ 4-Isopropenyl-1-methylcyclohexanol, trans- $$ tran

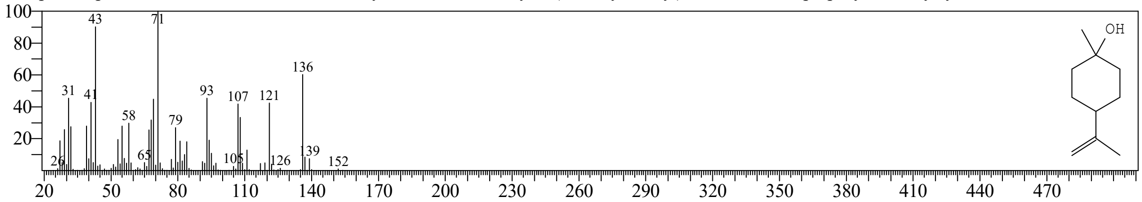

Hit#:4 Entry:223670 Library:NIST17-1.lib
SI:88 Formula:C24H48BNO2 CAS:0-00-0 MolWeight:393 RetIndex:0
CompName:N-Ethyl-2-amino-octade-4-en-1,3-diol buthaneboronate $$ N-(2-Butyl-4-[(1E)-1-pentadecenyl]-1,3,2-dioxaborinan-5-yl)-N-ethylamine # $$

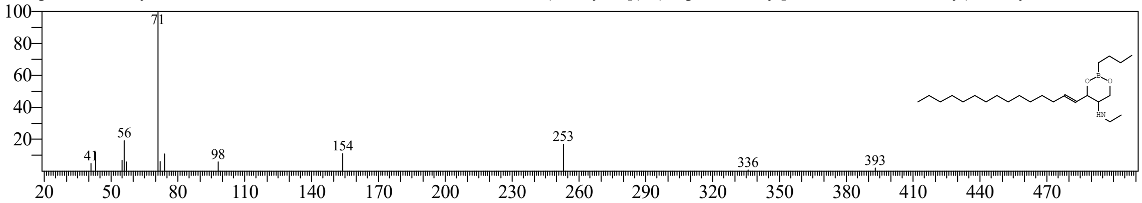

Hit#:5 Entry:18802 Library:NIST17-1.lib
SI:87 Formula:C10H18O CAS:138-87-4 MolWeight:154 RetIndex:1158
CompName:Cyclohexanol, 1-methyl-4-(1-methylethenyl)- $$ p-Menth-8-en-1-ol $$ .beta.-Terpineol $$ t-Menth-1-en-8-ol $$ .beta.-Terpinol $$ 4-Isopropen

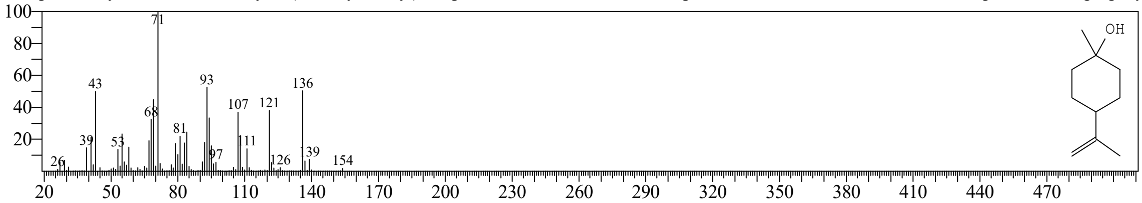

Qualitative Analysis Report

<< Target >>
Line#:26 R.Time:18.875(Scan#:3571) Retention Index:1159 MassPeaks:200
RawMode:Averaged 18.871-18.879(3570-3572) BasePeak:83.10(6694)
BG Mode:Calc. from Peak Group 1 - Event 1 Scan

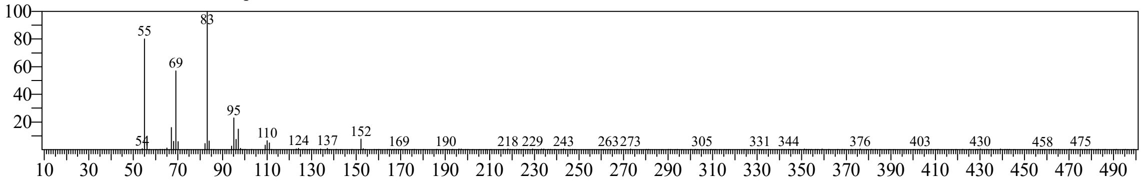

Hit#:1 Entry:26728 Library:NIST17-1.lib
SI:92 Formula:C10H16O2 CAS:90358-41-1 MolWeight:168 RetIndex:1153
CompName:(Z)-(E)-2-Methylbut-2-en-1-yl 2-methylbut-2-enoate $$ 2-Butenoic acid, 2-methyl-, (2E)-2-methyl-2-buten-1-yl ester, (2Z)- $$ 2-Butenoic acid,

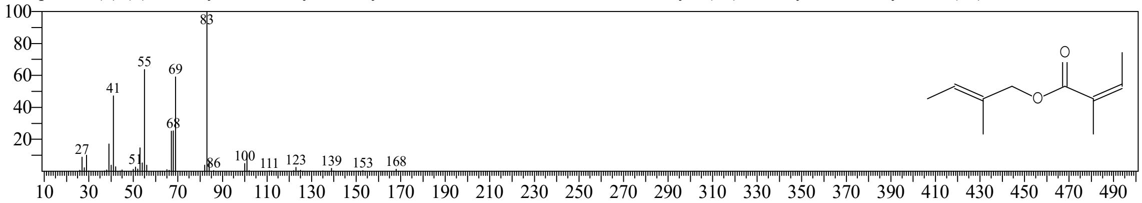

Hit#:2 Entry:2277 Library:NIST17-1.lib
SI:91 Formula:C2H2F4 CAS:359-35-3 MolWeight:102 RetIndex:0
CompName:Ethane, 1,1,2,2-tetrafluoro- $$ Freon 134 $$ R 134 $$ 1,1,2,2-Tetrafluoroethane $$ CHF2CHF2 $$

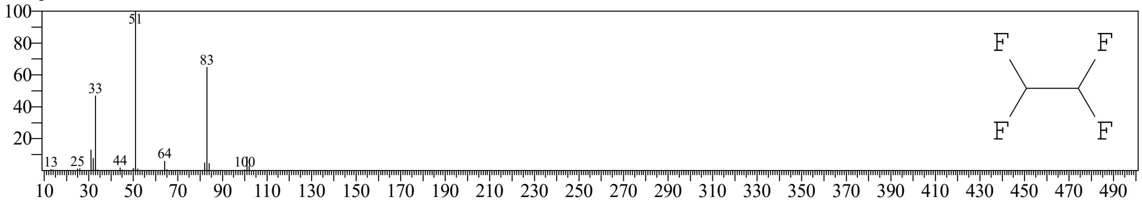

Hit#:3 Entry:3389 Library:NIST17-1.lib
SI:91 Formula:C3HN3S CAS:57352-02-0 MolWeight:111 RetIndex:0
CompName:5-Cyano-1,2,3-thiadiazole $$ 1,2,3-Thiadiazole-5-carbonitrile # $$

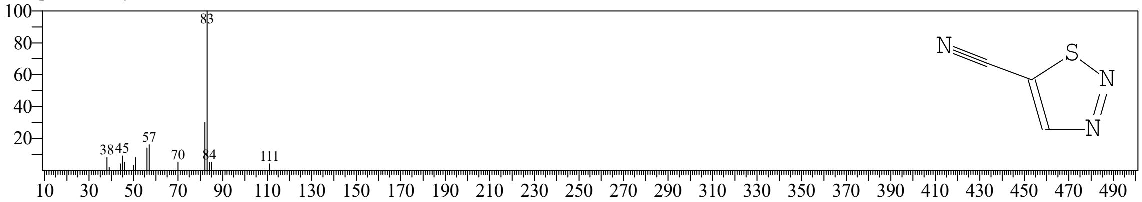

Hit#:4 Entry:2365 Library:FFNSC 3.lib
SI:90 Formula:C10 H14 O CAS:539-70-8 MolWeight:150 RetIndex:1149
CompName:Myrcenone

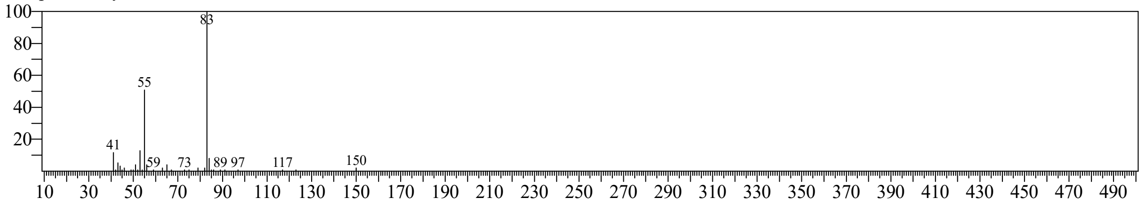

Hit#:5 Entry:20661 Library:NIST17-1.lib
SI:89 Formula:C7H14N2O2 CAS:72553-41-4 MolWeight:158 RetIndex:0
CompName:Diazene, 1-cyclohexyl-2-methoxy-, 1-oxide $$ 1-Cyclohexyl-2-methoxydiazene 1-oxide # $$

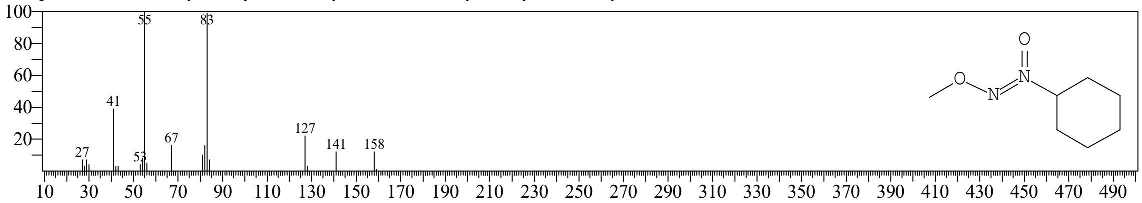

Qualitative Analysis Report

<< Target >>
Line#:27 R.Time:18.950(Scan#:3589) Retention Index:1160 MassPeaks:193
RawMode:Averaged 18.946-18.954(3588-3590) BasePeak:53.05(5489)
BG Mode:Calc. from Peak Group 1 - Event 1 Scan

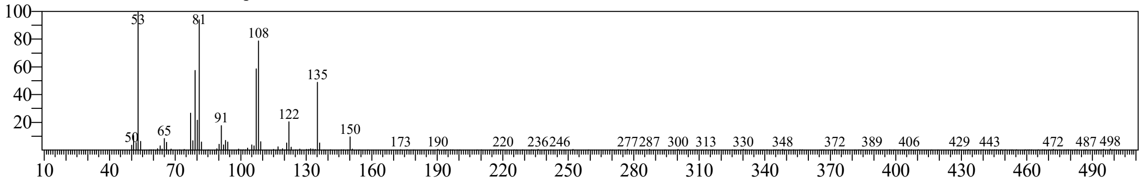

Hit#:1 Entry:73 Library:NIST17-1.lib
SI:98 Formula:F2HN CAS:10405-27-3 MolWeight:53 RetIndex:0
CompName:Difluoramine $$ Difluoroamine $$ HNF2 $$ Fluorimide $$ N,N-Difluoroamine # $$

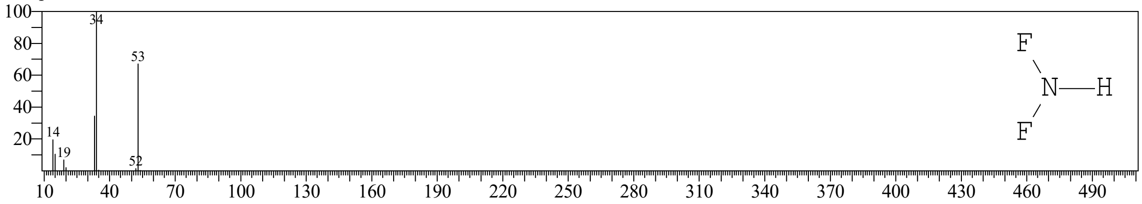

Hit#:2 Entry:1096 Library:FFNSC 3.lib
SI:91 Formula:C10 H14 O CAS:30460-92-5 MolWeight:150 RetIndex:1164
CompName:Pinocarvone

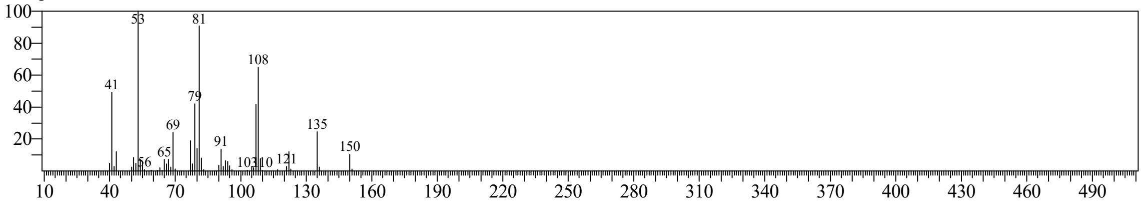

Hit#:3 Entry:158522 Library:NIST17-1.lib
SI:83 Formula:C11H5N7O5 CAS:0-00-0 MolWeight:315 RetIndex:0
CompName:12-(Furan-2-ylmethyl)-4,9,15-trioxa-3,5,8,10,12,14,16-heptaazatetracyclo[11.3.0.0(2,6).0(7,11)]hexadeca-1(16),2,5,7,10,13-hexaen-3-one

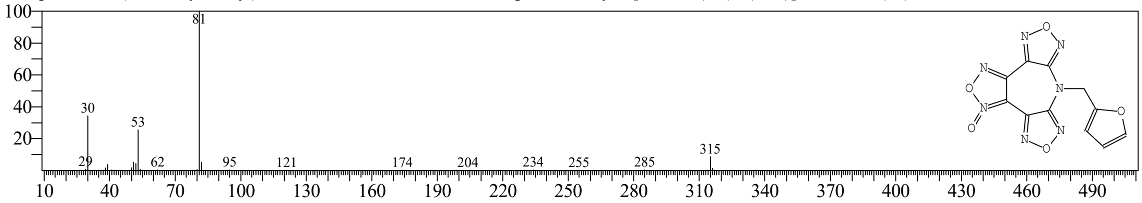

Hit#:4 Entry:1311 Library:NIST17-1.lib
SI:83 Formula:C7H10 CAS:3642-12-4 MolWeight:94 RetIndex:0
CompName:Hepta-1,2,6-heptatriene

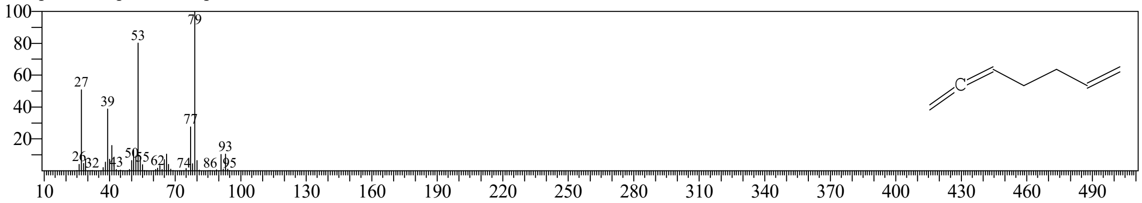

Hit#:5 Entry:574 Library:NIST17-1.lib
SI:83 Formula:C5H6O CAS:4009-55-6 MolWeight:82 RetIndex:0
CompName:3,4-Pentadienal

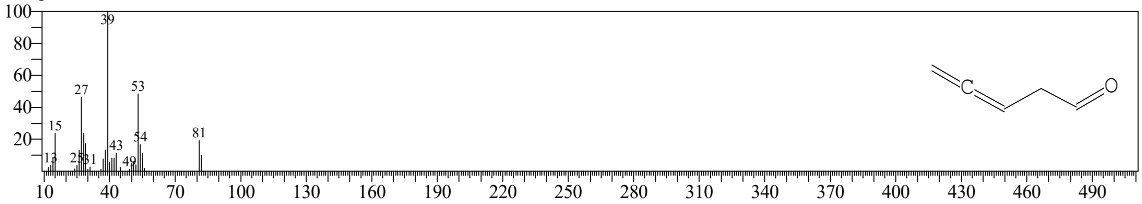

Qualitative Analysis Report

<< Target >>
Line#:28 R.Time:19.154(Scan#:3638) Retention Index:1165 MassPeaks:249
RawMode:Averaged 19.150-19.158(3637-3639) BasePeak:95.10(3814)
BG Mode:Calc. from Peak Group 1 - Event 1 Scan

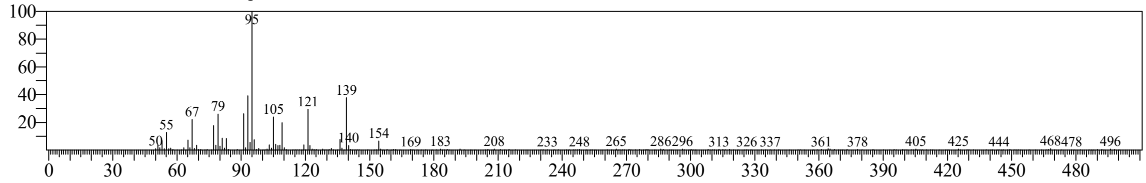

Hit#:1 Entry:11826 Library:NIST17-1.lib
SI:88 Formula:C8H12O2 CAS:0-00-0 MolWeight:140 RetIndex:1162
CompName:1,2-Dimethyl-cyclopent-2-enecarboxylic acid $$ 1,2-Dimethyl-2-cyclopentene-1-carboxylic acid # $$

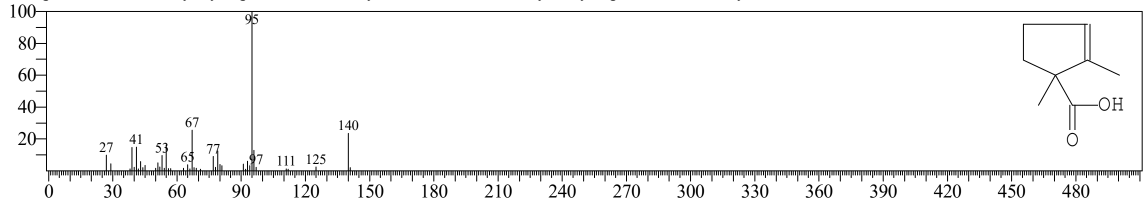

Hit#:2 Entry:5613 Library:NIST17-1.lib
SI:88 Formula:BrN3 CAS:13973-87-0 MolWeight:121 RetIndex:0
CompName:Bromine azide $$ Bromine azide (Br(N3)) $$ Bromine nitride $$ Nitrogen bromide (N3Br) $$

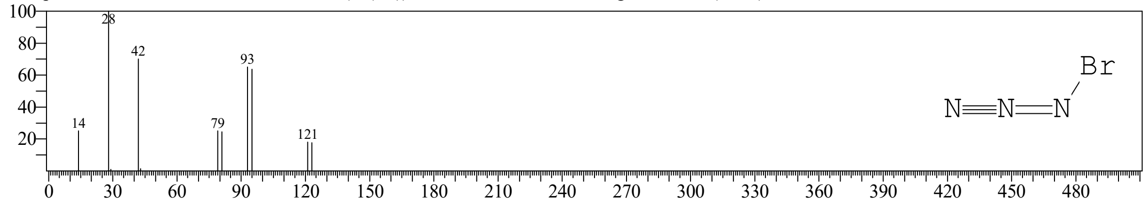

Hit#:3 Entry:11236 Library:NIST17-1.lib
SI:87 Formula:CH2BrNO2 CAS:563-70-2 MolWeight:139 RetIndex:0
CompName:Bromonitromethane $$ Methane, bromonitro- $$

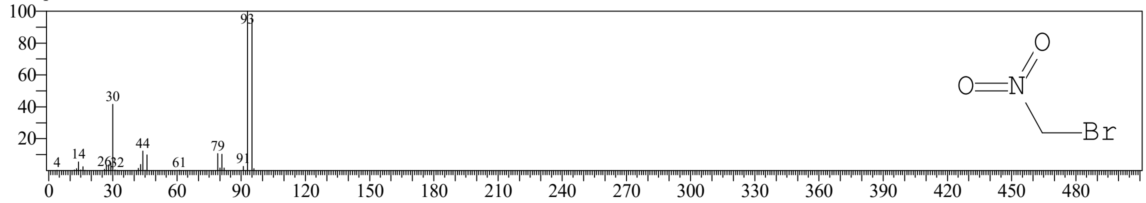

Hit#:4 Entry:3169 Library:NIST17-1.lib
SI:85 Formula:C5H6N2O CAS:25016-16-4 MolWeight:110 RetIndex:1156
CompName:Ethanone, 1-(1H-pyrazol-4-yl)- $$ Ketone, methyl pyrazol-4-yl $$ 4-Acetylpyrazole $$ 1-(1H-Pyrazol-4-yl)ethanone # $$

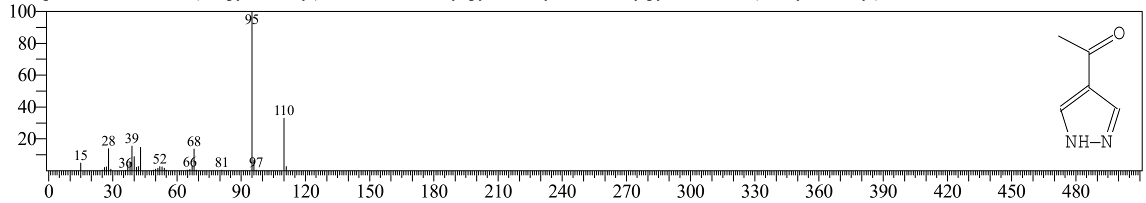

Hit#:5 Entry:6426 Library:NIST17-1.lib
SI:84 Formula:C2H8NO3P CAS:2697-42-9 MolWeight:125 RetIndex:0
CompName:Phosphoramidic acid, dimethyl ester $$ Dimethyl phosphoramidate $$ Dimethyl amidophosphate # $$

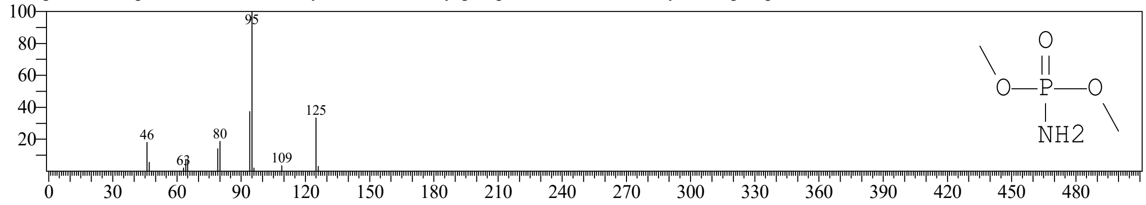

Qualitative Analysis Report

<< Target >>
Line#:29 R.Time:19.413(Scan#:3700) Retention Index:1170 MassPeaks:299
RawMode:Averaged 19.408-19.417(3699-3701) BasePeak:95.10(262509)
BG Mode:Calc. from Peak Group 1 - Event 1 Scan

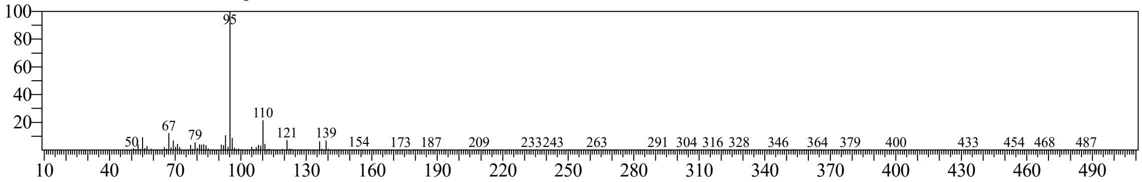

Hit#:1 Entry:1262 Library:FFNSC 3.lib
SI:97 Formula:C10 H18 O CAS:507-70-0 MolWeight:154 RetIndex:1173
CompName:Borneol

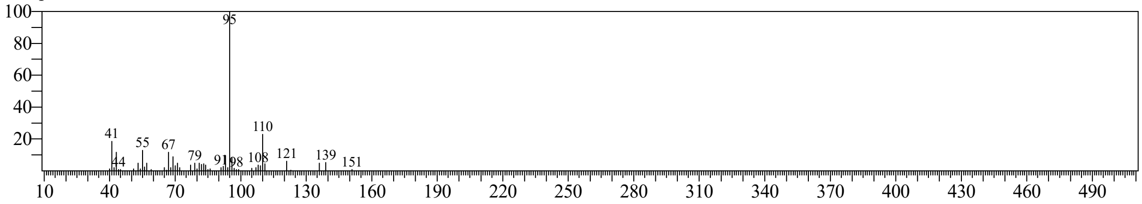

Hit#:2 Entry:687 Library:FFNSC 3.lib
SI:94 Formula:C10 H18 O CAS:10385-78-1 MolWeight:154 RetIndex:1165
CompName:Isoborneol

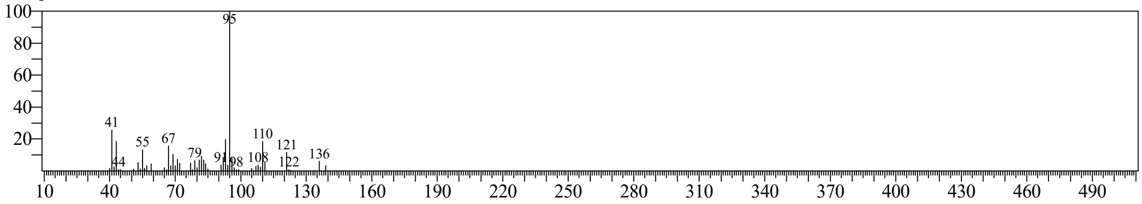

Hit#:3 Entry:26760 Library:NIST17-1.lib
SI:90 Formula:C10H16O2 CAS:5809-03-0 MolWeight:168 RetIndex:1172
CompName:2-Cyclopentene-1-carboxylic acid, 1,2-dimethyl-, ethyl ester $$ Ethyl 1,2-dimethyl-2-cyclopentene-1-carboxylate # $$

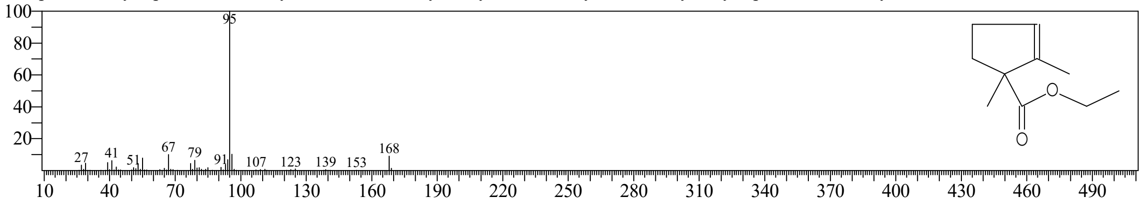

Hit#:4 Entry:36077 Library:NIST17-1.lib
SI:89 Formula:C10H14O3 CAS:0-00-0 MolWeight:182 RetIndex:1179
CompName:2-Furoic acid, 3-methylbut-2-yl ester

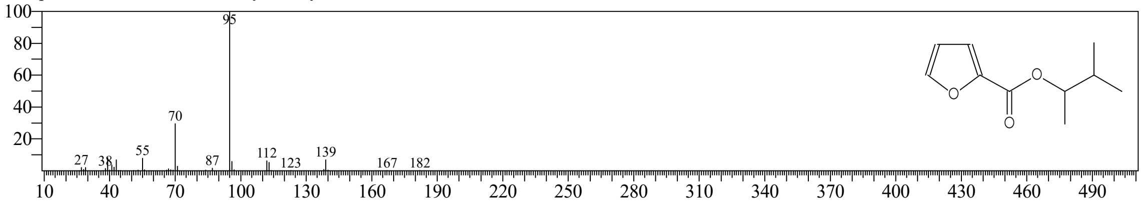

Hit#:5 Entry:26963 Library:NIST17-1.lib
SI:88 Formula:C11H20O CAS:2371-42-8 MolWeight:168 RetIndex:1161
CompName:2-Methylisoborneol

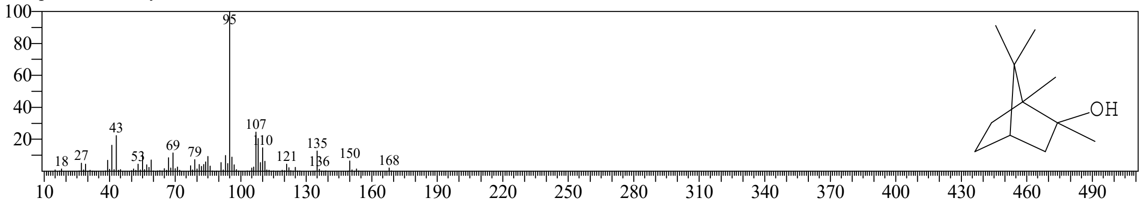

Qualitative Analysis Report

<< Target >>
Line#:30   R.Time:19.592(Scan#:3743) Retention Index:1174 MassPeaks:288
RawMode:Averaged 19.587-19.596(3742-3744) BasePeak:55.05(16745)
BG Mode:Calc. from Peak Group 1 - Event 1 Scan

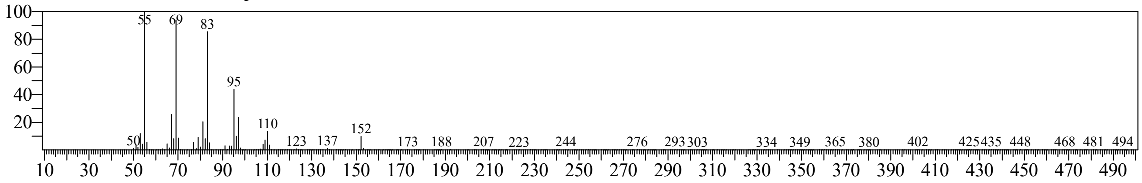

Hit#:1 Entry:1097 Library:FFNSC 3.lib
SI:97 Formula:C10 H16 O CAS:15358-88-0 MolWeight:152 RetIndex:1176
CompName:Pinocamphone <cis->

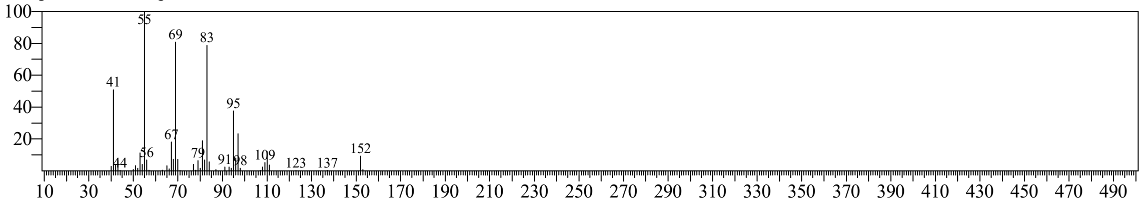

Hit#:2 Entry:29226 Library:NIST17-1.lib
SI:93 Formula:C8H12O4 CAS:0-00-0 MolWeight:172 RetIndex:1174
CompName:Oxalic acid, cyclobutyl ethyl ester

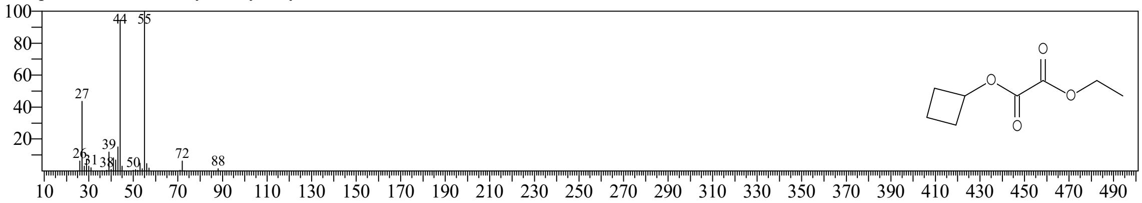

Hit#:3 Entry:659 Library:NIST17-1.lib
SI:92 Formula:C2H4N4 CAS:16681-77-9 MolWeight:84 RetIndex:0
CompName:1H-Tetrazole, 1-methyl- $$ N-Methyltetrazole $$ 1-Methyl-1H-tetrazole $$ 1-Methyltetrazole $$ 1-Methyl-1H-tetraazole # $$

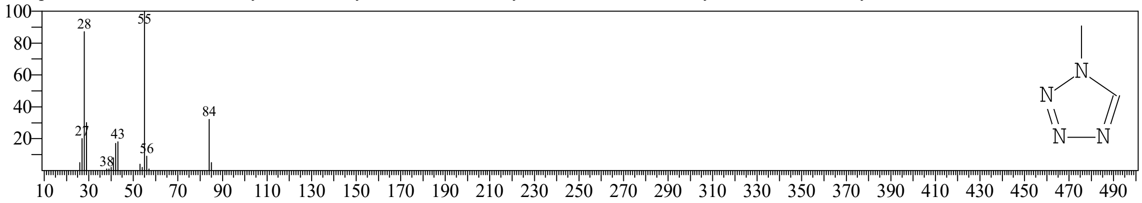

Hit#:4 Entry:640 Library:NIST17-1.lib
SI:89 Formula:C5H9N CAS:2769-64-4 MolWeight:83 RetIndex:0
CompName:Butane, 1-isocyano- $$ Butyl isocyanide $$ n-Butyl isocyanide $$ Butylisonitrile $$ n-C4H9NC $$

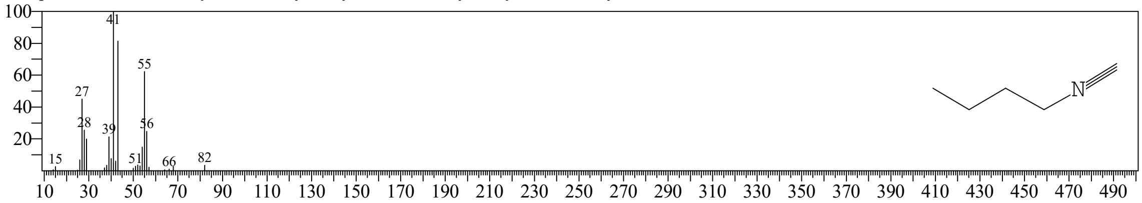

Hit#:5 Entry:87 Library:NIST17-1.lib
SI:88 Formula:C3H5N CAS:624-79-3 MolWeight:55 RetIndex:0
CompName:Ethyl isocyanide $$ Ethyl carbylamine $$ Ethane, isocyano- $$ Isocyanoethane $$ C2H5NC $$ Carbylamine, ethyl- $$ Ethylisokyanid $$ Ethyl

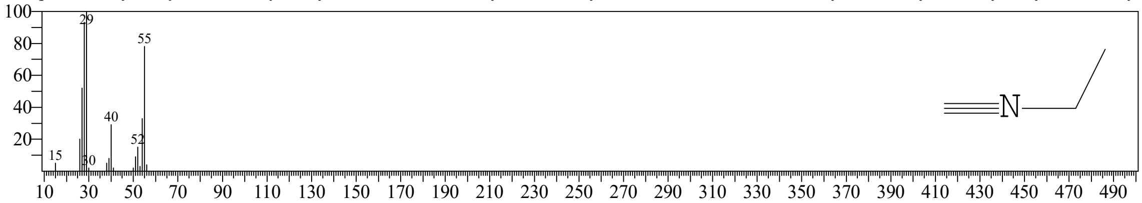

Qualitative Analysis Report

<< Target >>
Line#:31 R.Time:19.817(Scan#:3797) Retention Index:1179 MassPeaks:276
RawMode:Averaged 19.813-19.821(3796-3798) BasePeak:71.05(44589)
BG Mode:Calc. from Peak Group 1 - Event 1 Scan

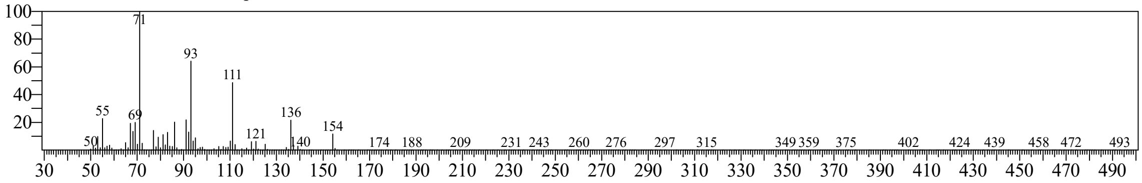

Hit#:1 Entry:1006 Library:FFNSC 3.lib
SI:95 Formula:C10 H18 O CAS:562-74-3 MolWeight:154 RetIndex:1184
CompName:Terpinen-4-ol

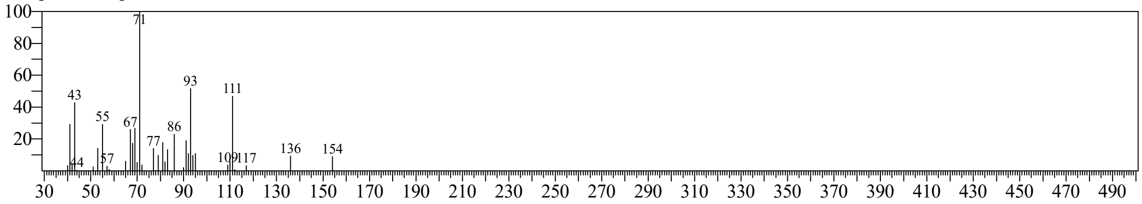

Hit#:2 Entry:19553 Library:NIST17-1.lib
SI:89 Formula:C7H12N2O2 CAS:26537-51-9 MolWeight:156 RetIndex:0
CompName:Sydnone, 3-isopentyl- $$ 3-Isopentyl-1,2,3-oxadiazol-3-ium-5-olate # $$

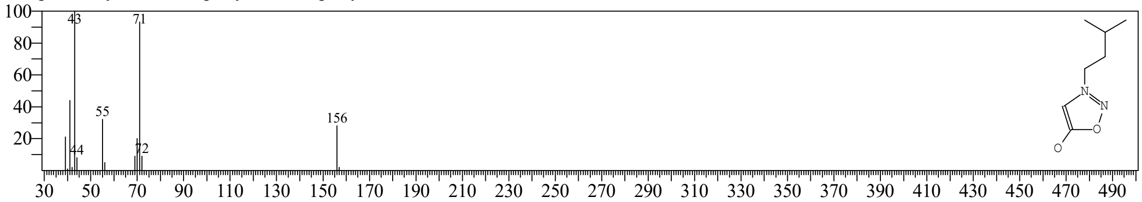

Hit#:3 Entry:13448 Library:NIST17-1.lib
SI:87 Formula:C4H4N2O2S CAS:22097-10-5 MolWeight:144 RetIndex:0
CompName:1,2,3-Thiadiazole-4-carboxylic acid, 5-methyl- $$ 5-Methyl-1,2,3-thiadiazole-4-carboxylic acid # $$

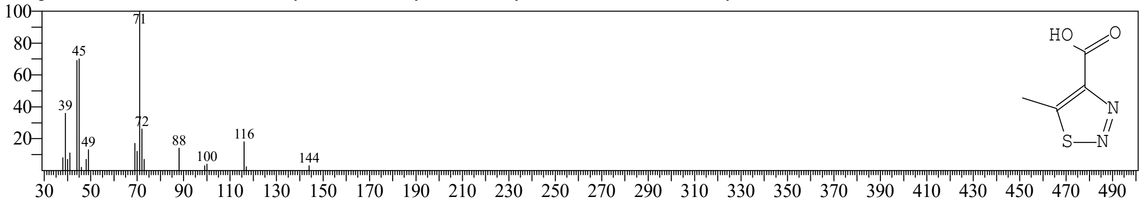

Hit#:4 Entry:1942 Library:NIST17-1.lib
SI:87 Formula:C3H4N2S CAS:50406-54-7 MolWeight:100 RetIndex:0
CompName:1,2,3-Thiadiazole, 5-methyl- $$ 5-Methyl-1,2,3-thiadiazole # $$

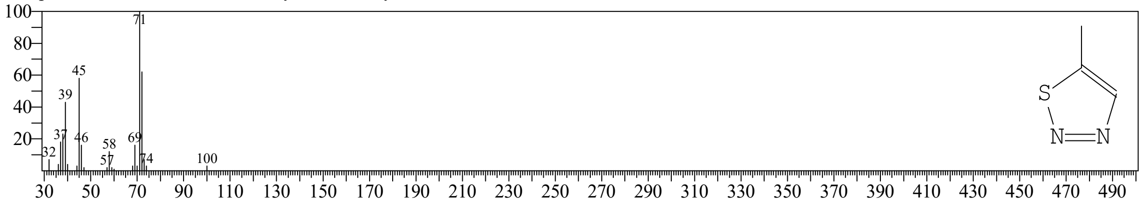

Hit#:5 Entry:223670 Library:NIST17-1.lib
SI:86 Formula:C24H48BNO2 CAS:0-00-0 MolWeight:393 RetIndex:0
CompName:N-Ethyl-2-amino-octade-4-en-1,3-diol buthaneboronate $$ N-(2-Butyl-4-[(1E)-1-pentadecenyl]-1,3,2-dioxaborinan-5-yl)-N-ethylamine # $$

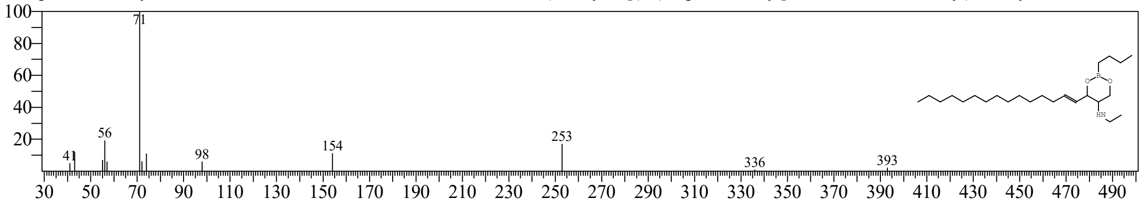

Qualitative Analysis Report

<< Target >>
Line#:32 R.Time:20.079(Scan#:3860) Retention Index:1184 MassPeaks:273
RawMode:Averaged 20.075-20.083(3859-3861) BasePeak:135.15(3916)
BG Mode:Calc. from Peak Group 1 - Event 1 Scan

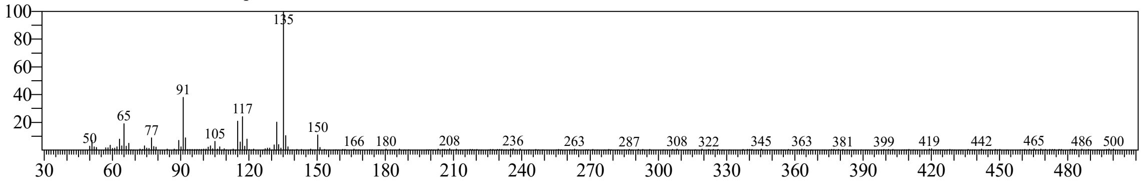

Hit#:1 Entry:875 Library:FFNSC 3.lib
SI:96 Formula:C10 H14 O CAS:1197-01-9 MolWeight:150 RetIndex:1189
CompName:Cymen-8-ol <para->

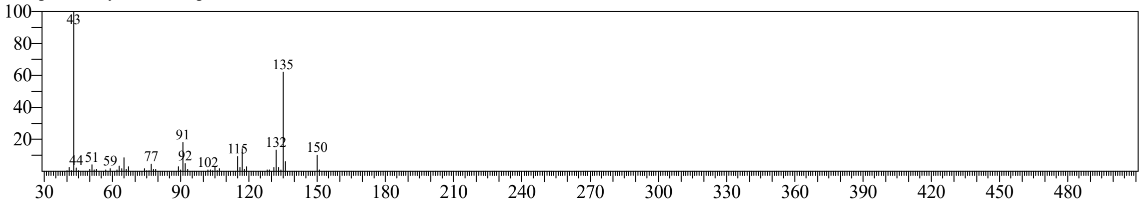

Hit#:2 Entry:215434 Library:NIST17-1.lib
SI:94 Formula:C20H20N4O4 CAS:19226-32-5 MolWeight:380 RetIndex:0
CompName:Benzoic acid, 4-methoxy-, anhydride with N-(4,5-dimethyl-1H-1,2,3-triazol-1-yl)-4-methoxybenzenecarboximidic acid $$ p-Anisic acid, anhydr

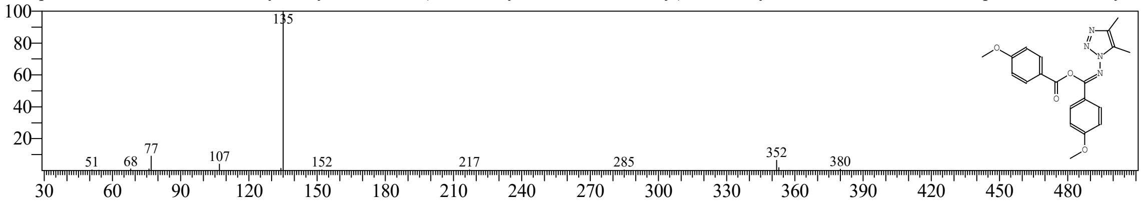

Hit#:3 Entry:25502 Library:NIST17-1.lib
SI:90 Formula:C10H14O2 CAS:3395-83-3 MolWeight:166 RetIndex:1193
CompName:Benzene, 1-(dimethoxymethyl)-4-methyl- $$ .alpha.,.alpha.-Dimethoxy-p-xylene $$ p-Tolualdehyde, dimethyl acetal $$ p-Tolyl aldehyde dimeth

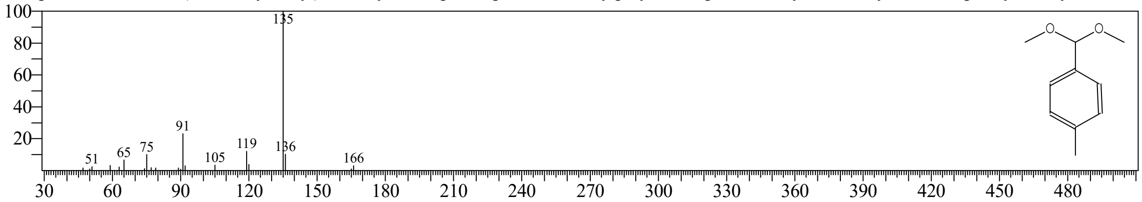

Hit#:4 Entry:9744 Library:NIST17-1.lib
SI:88 Formula:C7H5NO2 CAS:21725-69-9 MolWeight:135 RetIndex:1185
CompName:1,2-Benzisoxazol-3(2H)-one $$ 1,2-Benzisoxazolin-3-one $$ 1,2-Benzisoxazol-3-ol $$ 3-Hydroxybenzisoxazole $$ 3-Hydroxy-1,2-benzisoxaz

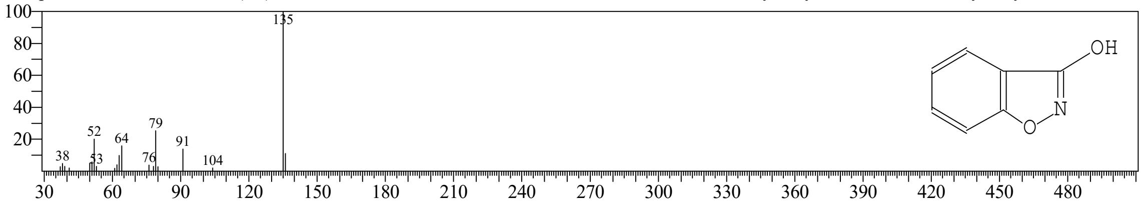

Hit#:5 Entry:195663 Library:NIST17-1.lib
SI:88 Formula:C18H14N2O6 CAS:40499-78-3 MolWeight:354 RetIndex:0
CompName:Furazan, di-p-anisoyl-, 2-oxide $$ Furazan N-oxide, 4,5-bis(4-methoxybenzoyl)- $$

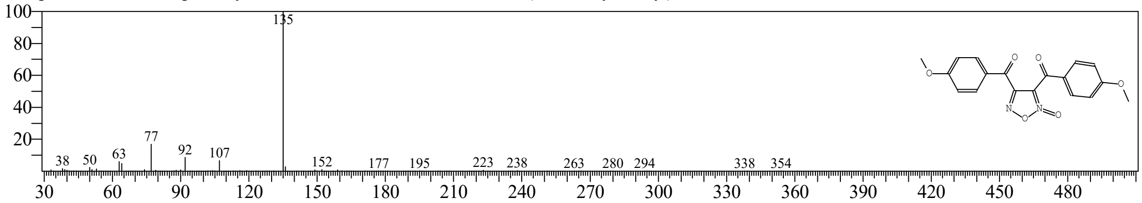

Qualitative Analysis Report

<< Target >>
Line#:33   R.Time:20.488(Scan#:3958) Retention Index:1193 MassPeaks:300
RawMode:Averaged 20.483-20.492(3957-3959) BasePeak:59.05(81749)
BG Mode:Calc. from Peak Group 1 - Event 1 Scan

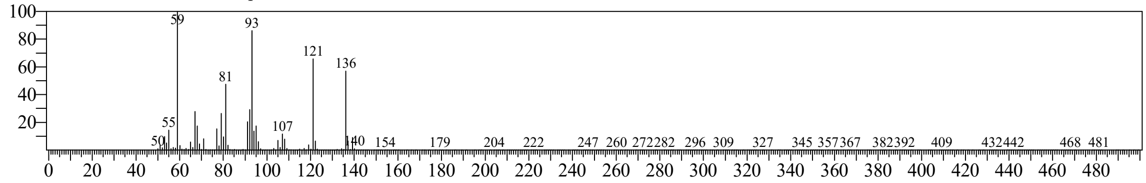

Hit#:1 Entry:120 Library:NIST17-1.lib
SI:96 Formula:CH5N3 CAS:113-00-8 MolWeight:59 RetIndex:0
CompName:Guanidine $$ Aminoformamidine $$ Aminomethanamidine $$ Carbamamidine $$ Carbamidine $$ Guanidin $$ Imidourea $$ Iminourea $$ (NI

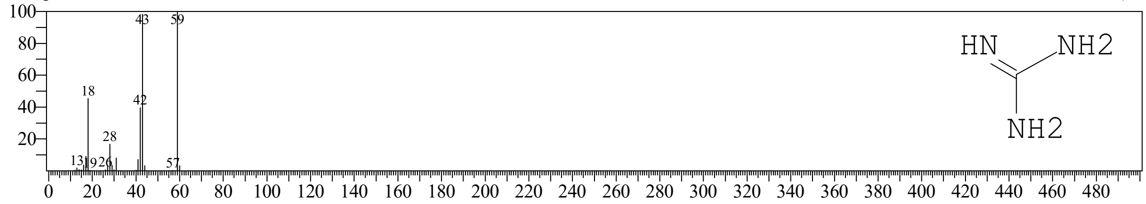

Hit#:2 Entry:27586 Library:NIST17-1.lib
SI:94 Formula:C4H12FN2PS CAS:36267-53-5 MolWeight:170 RetIndex:0
CompName:Phosphonofluoridothioic hydrazide, P-ethyl-2,2-dimethyl- $$ p-Ethyl-N',N'-dimethylphosphonohydrazidothioic fluoride # $$

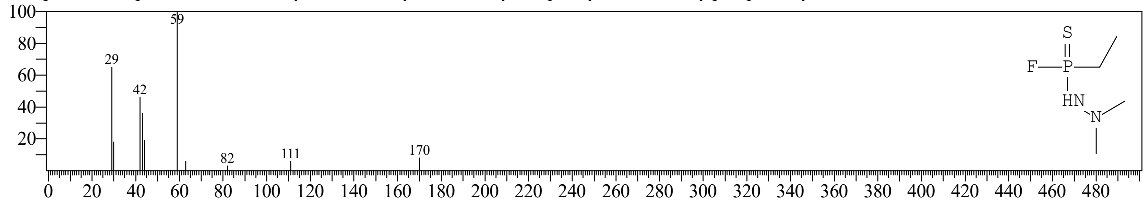

Hit#:3 Entry:497 Library:FFNSC 3.lib
SI:94 Formula:C10 H18 O CAS:98-55-5 MolWeight:154 RetIndex:1195
CompName:Terpineol <alpha->

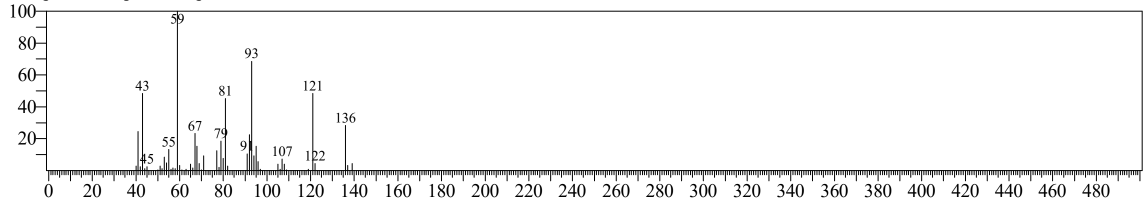

Hit#:4 Entry:144 Library:NIST17-1.lib
SI:86 Formula:C2H8Si CAS:1111-74-6 MolWeight:60 RetIndex:0
CompName:Silane, dimethyl- $$ Dimethylsilane $$ 2-Silapropane $$ (CH3)2SiH2 $$

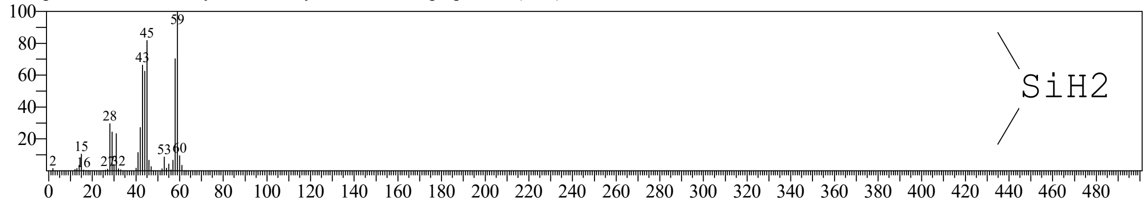

Hit#:5 Entry:20036 Library:NIST17-1.lib
SI:85 Formula:C10H20O CAS:16624-02-5 MolWeight:156 RetIndex:1191
CompName:Cycloheptanemethanol, .alpha.,.alpha.-dimethyl- $$ 2-Cycloheptyl-2-propanol # $$

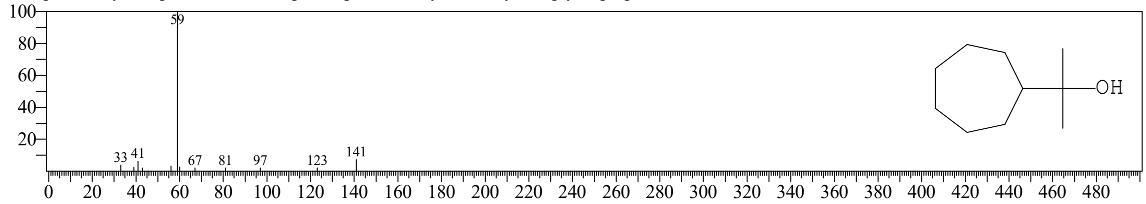

Qualitative Analysis Report

<< Target >>
Line#:34   R.Time:20.879(Scan#:4052) Retention Index:1202 MassPeaks:272
RawMode:Averaged 20.875-20.883(4051-4053) BasePeak:95.10(39211)
BG Mode:Calc. from Peak Group 1 - Event 1 Scan

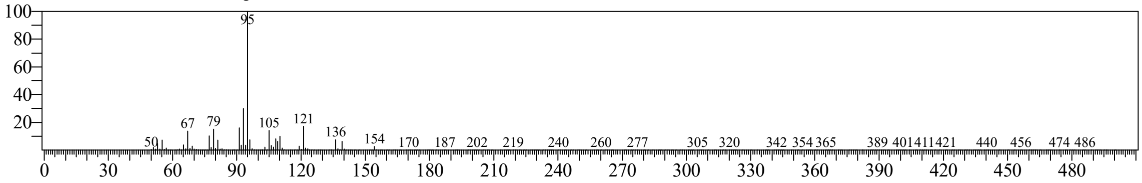

Hit#:1 Entry:11236 Library:NIST17-1.lib
SI:87 Formula:CH2BrNO2 CAS:563-70-2 MolWeight:139 RetIndex:0
CompName:Bromonitromethane $$ Methane, bromonitro- $$

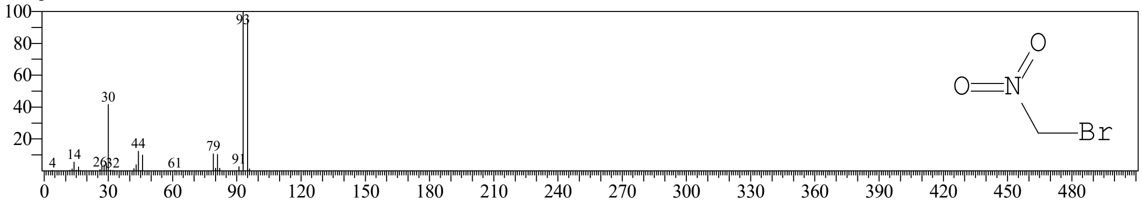

Hit#:2 Entry:1355 Library:NIST17-1.lib
SI:85 Formula:C5H5NO CAS:694-59-7 MolWeight:95 RetIndex:0
CompName:Pyridine, 1-oxide $$ Pyridine oxide $$ Pyridine N-oxide $$ Pyridine-1-oxide $$

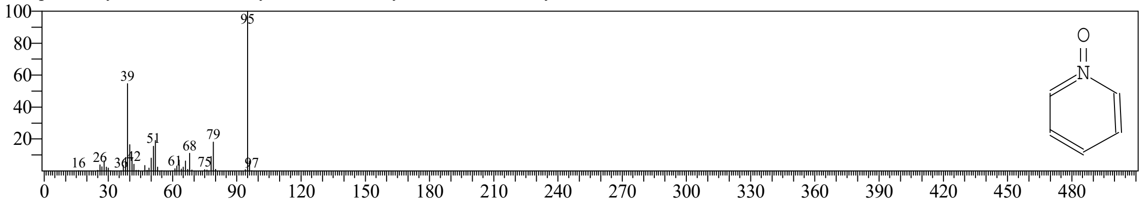

Hit#:3 Entry:6426 Library:NIST17-1.lib
SI:85 Formula:C2H8NO3P CAS:2697-42-9 MolWeight:125 RetIndex:0
CompName:Phosphoramidic acid, dimethyl ester $$ Dimethyl phosphoramidate $$ Dimethyl amidophosphate # $$

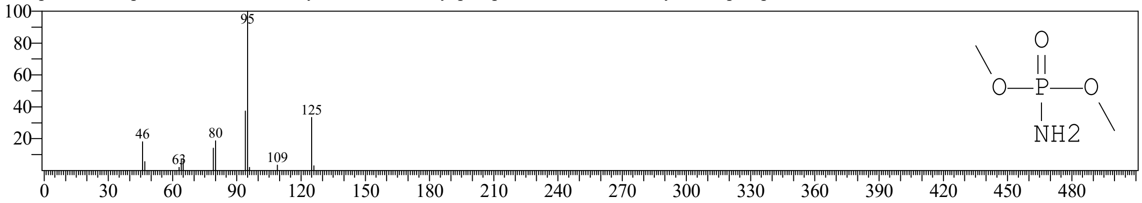

Hit#:4 Entry:10478 Library:NIST17-1.lib
SI:85 Formula:C5H7N5 CAS:0-00-0 MolWeight:137 RetIndex:0
CompName:4-(Azidomethyl)-1-methylpyrazole

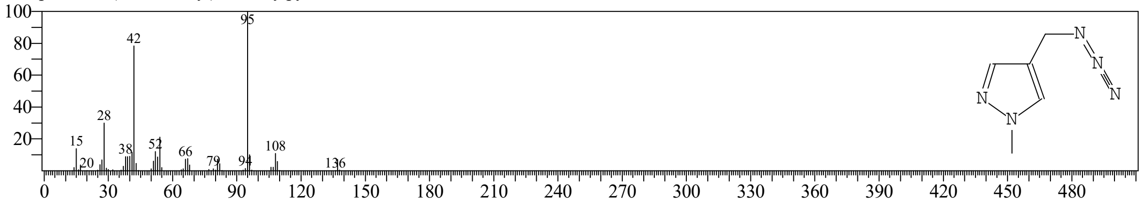

Hit#:5 Entry:25625 Library:NIST17-1.lib
SI:85 Formula:C11H18O CAS:19780-98-4 MolWeight:166 RetIndex:1197
CompName:2,6-Dimethyl-1-nonen-3-yn-5-ol

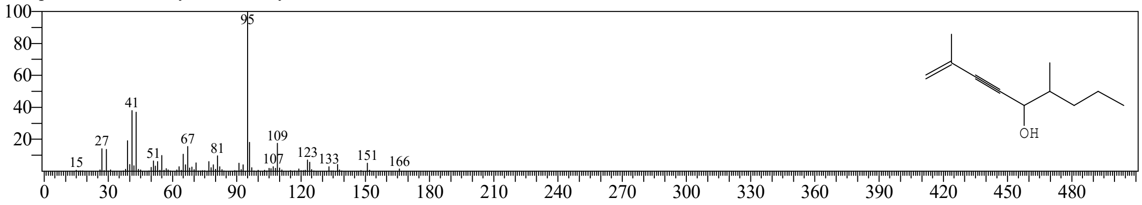

Qualitative Analysis Report

<< Target >>
Line#:35 R.Time:21.058(Scan#:4095) Retention Index:1206 MassPeaks:266
RawMode:Averaged 21.054-21.063(4094-4096) BasePeak:107.10(264697)
BG Mode:Calc. from Peak Group 1 - Event 1 Scan

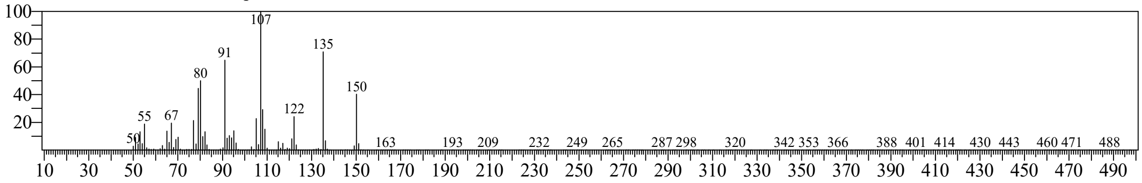

Hit#:1 Entry:820 Library:FFNSC 3.lib
SI:97 Formula:C10 H14 O CAS:80-57-9 MolWeight:150 RetIndex:1208
CompName:Verbenone

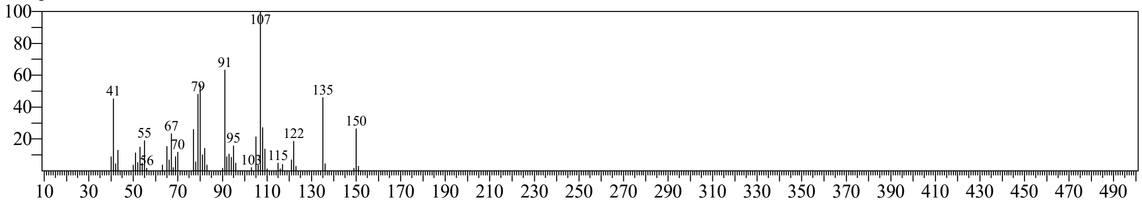

Hit#:2 Entry:7085 Library:NIST17-1.lib
SI:97 Formula:F5P CAS:7647-19-0 MolWeight:126 RetIndex:0
CompName:Phosphorus pentafluoride $$ Phosphorus(V) fluoride $$ PF5 $$ Phosphorane, pentafluoro- $$ UN 2198 $$ Phosphorus fluoride $$ Pentafluoro

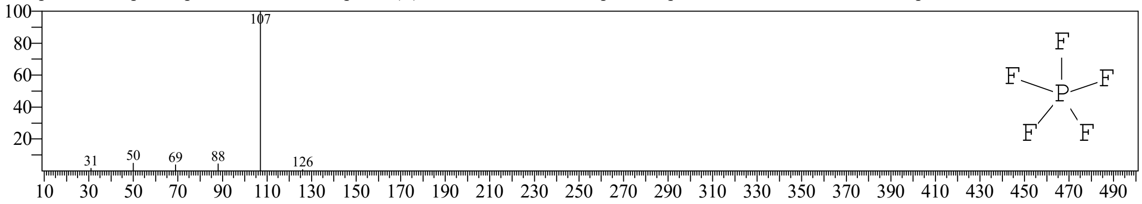

Hit#:3 Entry:9872 Library:NIST17-1.lib
SI:95 Formula:C2H5F4P CAS:0-00-0 MolWeight:136 RetIndex:0
CompName:Ethyltetrafluorophosphorane

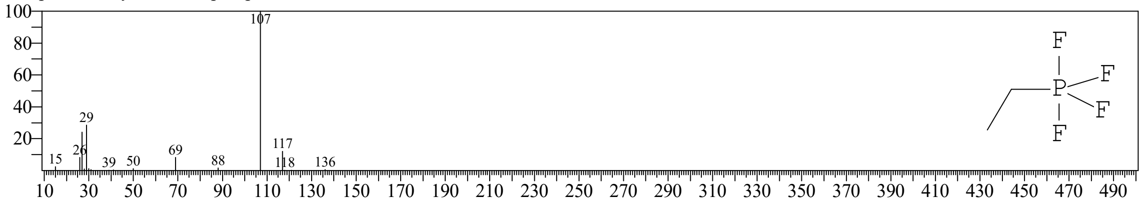

Hit#:4 Entry:33195 Library:NIST17-1.lib
SI:93 Formula:C10H14N2O CAS:14487-70-8 MolWeight:178 RetIndex:0
CompName:3-Diazo-1,7,7-trimethylbicyclo[2.2.1]heptan-2-one

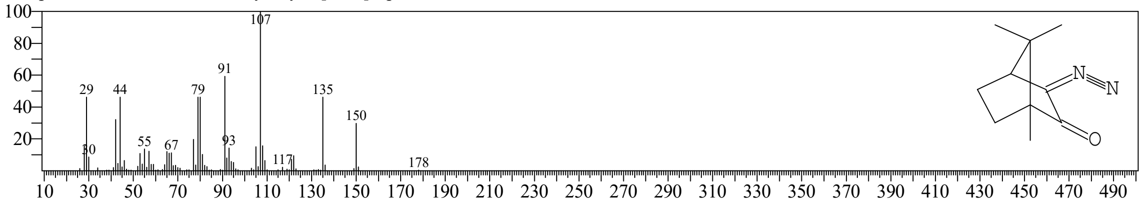

Hit#:5 Entry:9296 Library:NIST17-1.lib
SI:92 Formula:C2H3F4P CAS:3583-99-1 MolWeight:134 RetIndex:0
CompName:Tetrafluorovinylphosphorane

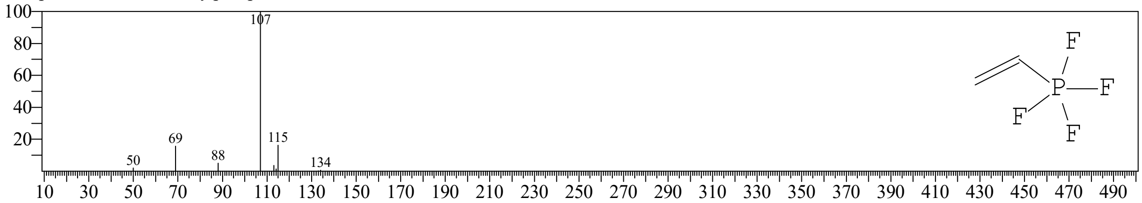

Qualitative Analysis Report

<< Target >>
Line#:36 R.Time:21.325(Scan#:4159) Retention Index:1211 MassPeaks:229
RawMode:Averaged 21.321-21.329(4158-4160) BasePeak:82.10(2531)
BG Mode:Calc. from Peak Group 1 - Event 1 Scan

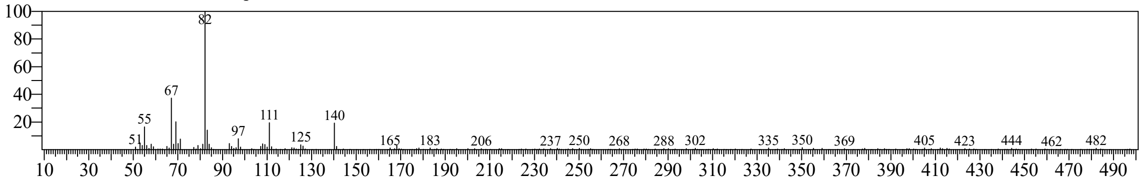

Hit#:1 Entry:3497 Library:NIST17-1.lib
SI:93 Formula:C2H6FO2P CAS:353-88-8 MolWeight:112 RetIndex:0
CompName:Methyl methylphosphonofluoridate $$ Phosphonofluoridic acid, methyl-, methyl ester $$ Methylphoshonic acid, fluoroanhydride, methyl ester $

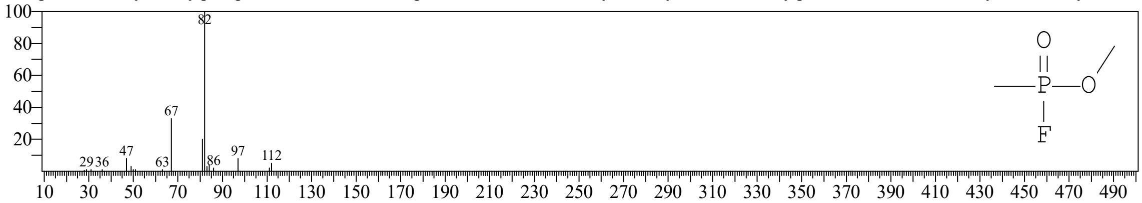

Hit#:2 Entry:6488 Library:NIST17-1.lib
SI:90 Formula:C6H11N3 CAS:6986-90-9 MolWeight:125 RetIndex:1215
CompName:1H-Imidazole-4-ethanamine, .alpha.-methyl- $$ .alpha.-Methylhistamine $$ 1-(1H-Imidazol-4-yl)-2-propanamine # $$

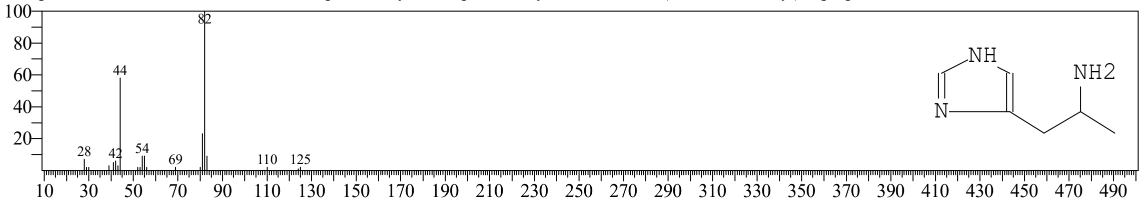

Hit#:3 Entry:18025 Library:NIST17-1.lib
SI:87 Formula:C9H20BN CAS:30430-68-3 MolWeight:153 RetIndex:0
CompName:2-Butyl-1-ethyl-1,2-azaborolidine

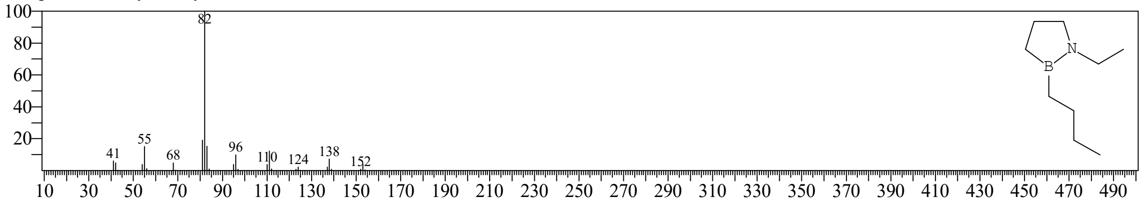

Hit#:4 Entry:555 Library:NIST17-1.lib
SI:85 Formula:CClFO CAS:353-49-1 MolWeight:82 RetIndex:0
CompName:Carbonic chloride fluoride $$ Carbonyl chloride fluoride $$ COClF $$ Carbonyl chlorofluoride $$ Estra-1,3,5(10), 7-tetraen-17-one, 3-hydroxy

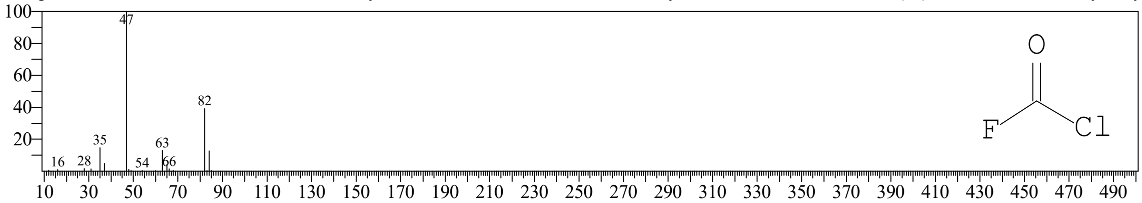

Hit#:5 Entry:556 Library:NIST17-1.lib
SI:85 Formula:CH3FOS CAS:56755-41-0 MolWeight:82 RetIndex:0
CompName:Methanesulfinyl fluoride

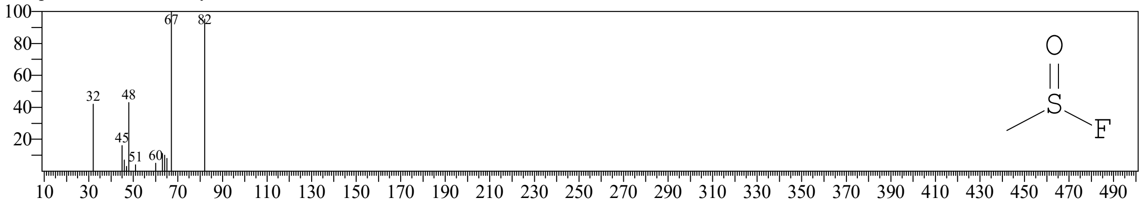

Qualitative Analysis Report

<< Target >>
Line#:37 R.Time:21.571(Scan#:4218) Retention Index:1217 MassPeaks:300
RawMode:Averaged 21.567-21.575(4217-4219) BasePeak:83.05(3797)
BG Mode:Calc. from Peak Group 1 - Event 1 Scan

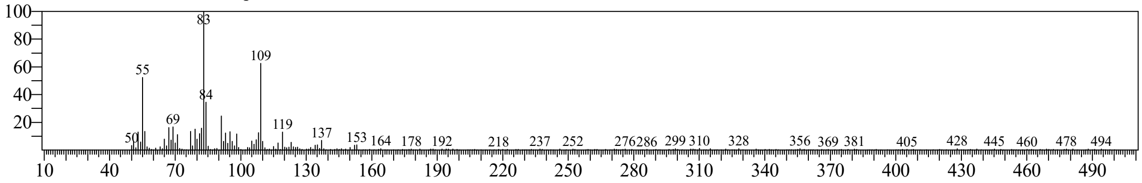

Hit#:1 Entry:3389 Library:NIST17-1.lib
SI:92 Formula:C3HN3S CAS:57352-02-0 MolWeight:111 RetIndex:0
CompName:5-Cyano-1,2,3-thiadiazole $$ 1,2,3-Thiadiazole-5-carbonitrile # $$

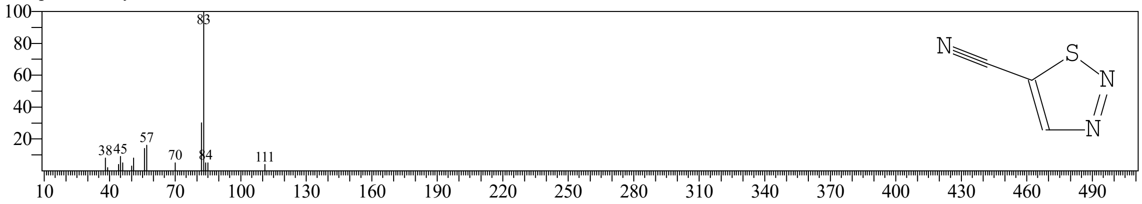

Hit#:2 Entry:939 Library:FFNSC 3.lib
SI:90 Formula:C10 H16 O CAS:1197-07-5 MolWeight:152 RetIndex:1223
CompName:Carveol <trans->

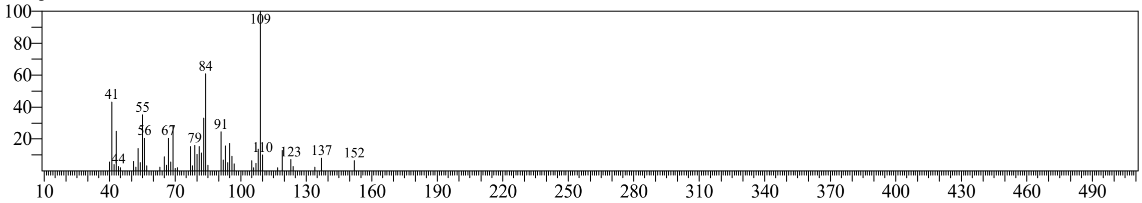

Hit#:3 Entry:36510 Library:NIST17-1.lib
SI:89 Formula:Cl2O7 CAS:12015-53-1 MolWeight:182 RetIndex:0
CompName:Dichlorine heptoxide $$ Chlorine oxide (Cl2O7) $$ Chlorine heptoxide $$ Perchloric anhydride $$ (Perchloryloxy)chlorane trioxide # $$

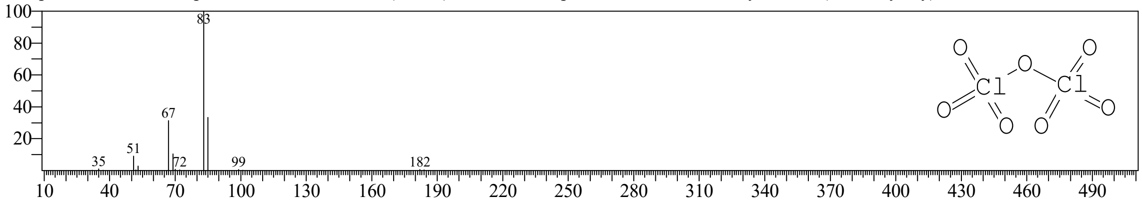

Hit#:4 Entry:29298 Library:NIST17-1.lib
SI:88 Formula:C8H16N2O2 CAS:108201-46-3 MolWeight:172 RetIndex:0
CompName:Diazene, 1-cyclohexyl-2-ethoxy-, 1-oxide $$ Cyclohexyl(ethoxy)diazene oxide # $$

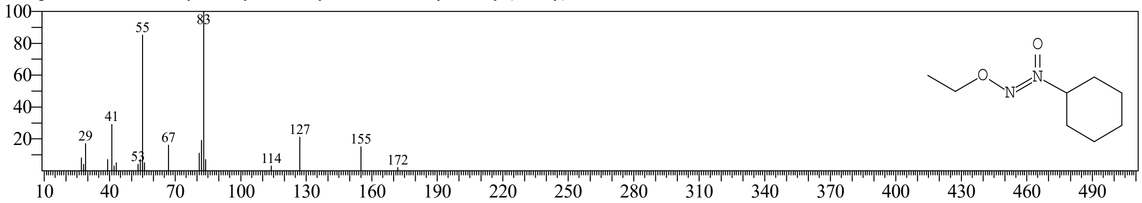

Hit#:5 Entry:12898 Library:NIST17-1.lib
SI:87 Formula:C8H14O2 CAS:140-77-2 MolWeight:142 RetIndex:1216
CompName:Cyclopentanepropanoic acid $$ Cyclopentanepropionic acid $$ Cyclopentylpropionic acid $$ Propionic acid, 3-cyclopentyl- $$ 3-Cyclopentylpı

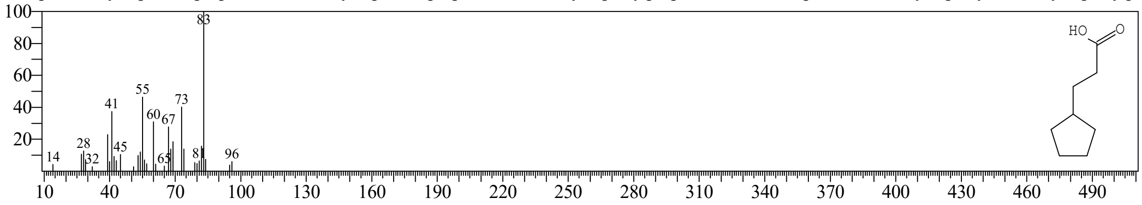

Qualitative Analysis Report

<< Target >>
Line#:38 R.Time:21.908(Scan#:4299) Retention Index:1224 MassPeaks:304
RawMode:Averaged 21.904-21.913(4298-4300) BasePeak:69.10(7519)
BG Mode:Calc. from Peak Group 1 - Event 1 Scan

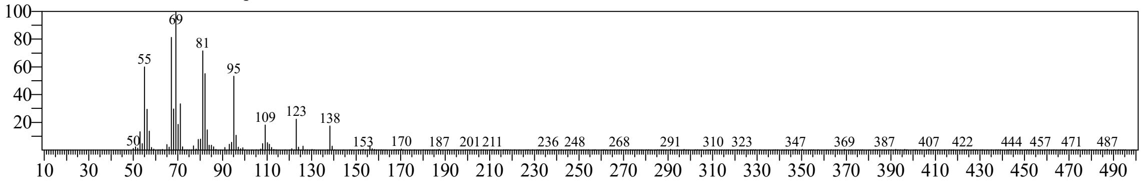

Hit#:1 Entry:228 Library:NIST17-1.lib
SI:97 Formula:C2H3N3 CAS:288-36-8 MolWeight:69 RetIndex:0
CompName:1H-1,2,3-Triazole $$ V-triazole $$ Osotriazole $$ Pyrrodiazole $$ Triazacyclopentadiene $$ 1,2,3-Triazole $$

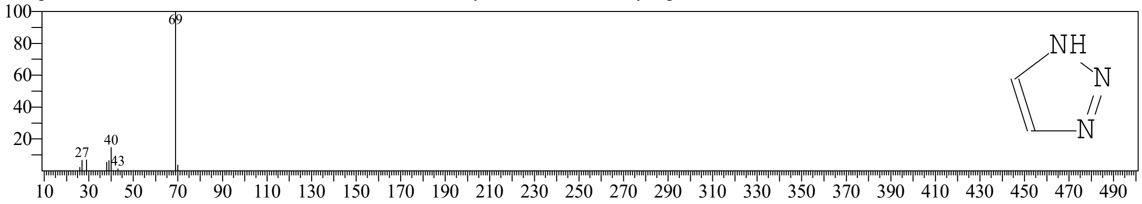

Hit#:2 Entry:922 Library:FFNSC 3.lib
SI:96 Formula:C10 H20 O CAS:106-22-9 MolWeight:156 RetIndex:1232
CompName:Citronellol

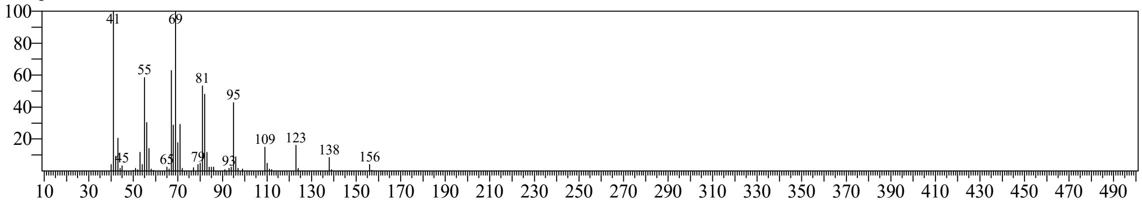

Hit#:3 Entry:577 Library:FFNSC 3.lib
SI:95 Formula:C10 H20 O CAS:141-25-3 MolWeight:156 RetIndex:1228
CompName:Oct-7-enol <3,7-dimethyl->

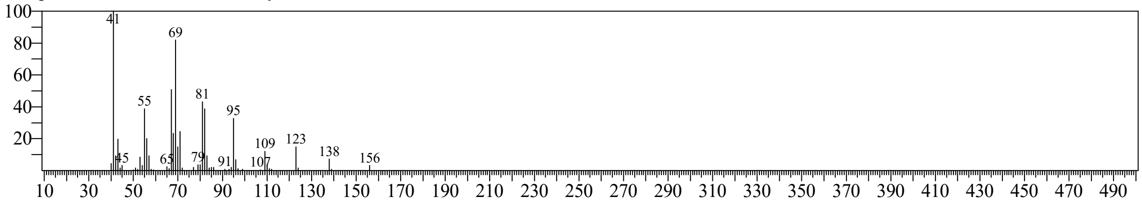

Hit#:4 Entry:981 Library:NIST17-1.lib
SI:94 Formula:CF4 CAS:75-73-0 MolWeight:88 RetIndex:0
CompName:Tetrafluoromethane $$ Carbon tetrafluoride $$ Methane, tetrafluoro- $$ Arcton 0 $$ Carbon fluoride (CF4) $$ Freon 14 $$ FC 14 $$ Perfluoro

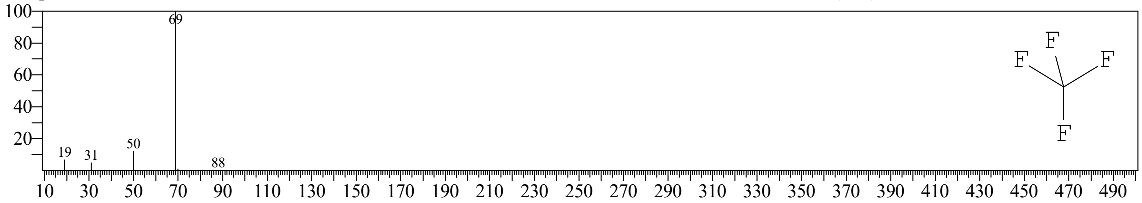

Hit#:5 Entry:10642 Library:NIST17-1.lib
SI:91 Formula:CF5P CAS:1112-04-5 MolWeight:138 RetIndex:0
CompName:Trifluoromethyldifluorophosphine $$ Trifluoromethylphosphonous difluoride # $$

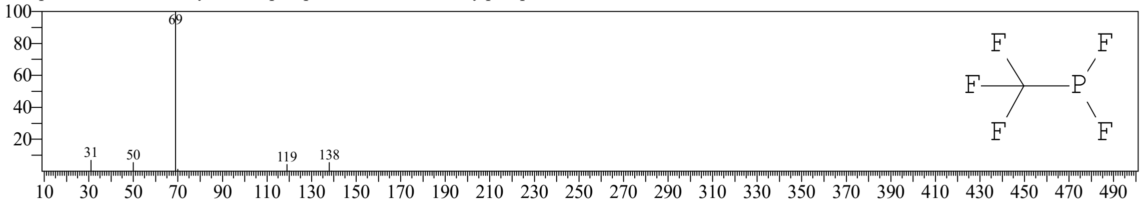

Qualitative Analysis Report

<< Target >>
Line#:39 R.Time:22.425(Scan#:4423) Retention Index:1235 MassPeaks:237
RawMode:Averaged 22.421-22.429(4422-4424) BasePeak:69.10(15824)
BG Mode:Calc. from Peak Group 1 - Event 1 Scan

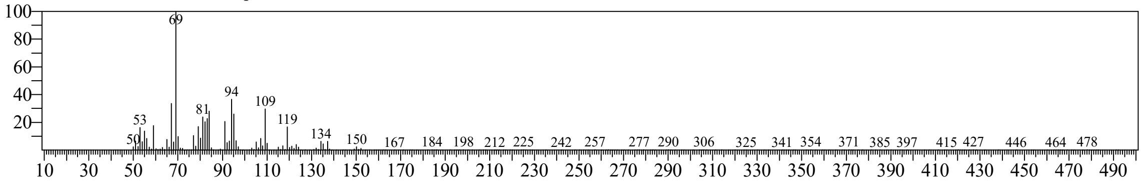

Hit#:1 Entry:228 Library:NIST17-1.lib
SI:99 Formula:C2H3N3 CAS:288-36-8 MolWeight:69 RetIndex:0
CompName:1H-1,2,3-Triazole $$ V-triazole $$ Osotriazole $$ Pyrrodiazole $$ Triazacyclopentadiene $$ 1,2,3-Triazole $$

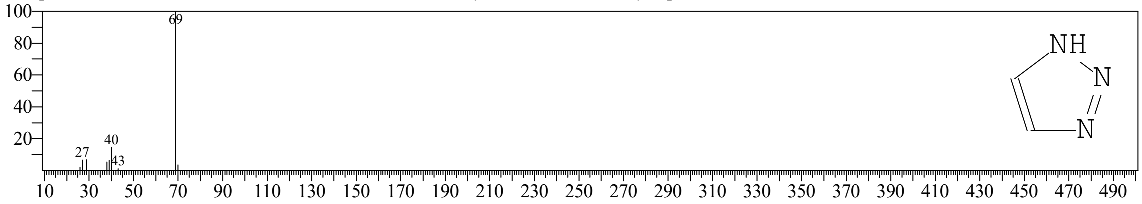

Hit#:2 Entry:1002 Library:FFNSC 3.lib
SI:96 Formula:C10 H16 O CAS:106-26-3 MolWeight:152 RetIndex:1238
CompName:Neral

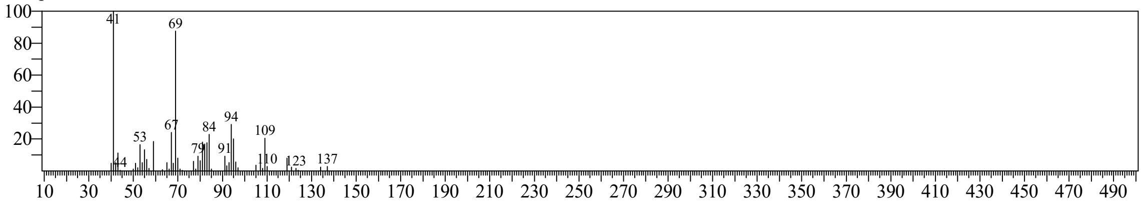

Hit#:3 Entry:981 Library:NIST17-1.lib
SI:95 Formula:CF4 CAS:75-73-0 MolWeight:88 RetIndex:0
CompName:Tetrafluoromethane $$ Carbon tetrafluoride $$ Methane, tetrafluoro- $$ Arcton 0 $$ Carbon fluoride (CF4) $$ Freon 14 $$ FC 14 $$ Perfluoro

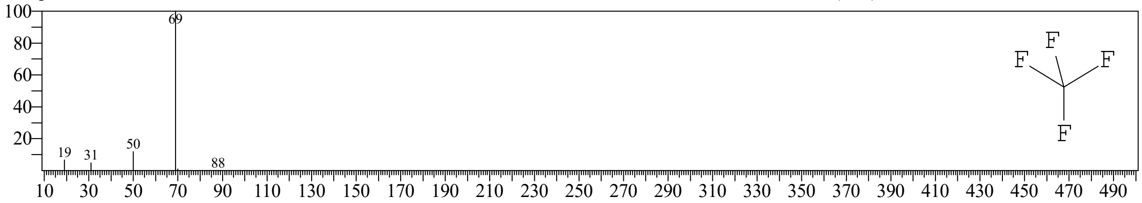

Hit#:4 Entry:9867 Library:NIST17-1.lib
SI:93 Formula:CF4OS CAS:812-12-4 MolWeight:136 RetIndex:0
CompName:Methanesulfinyl fluoride, trifluoro- $$ Trifluoromethanesulfinyl fluoride $$ Trifluoromethylsulfinyl fluoride $$

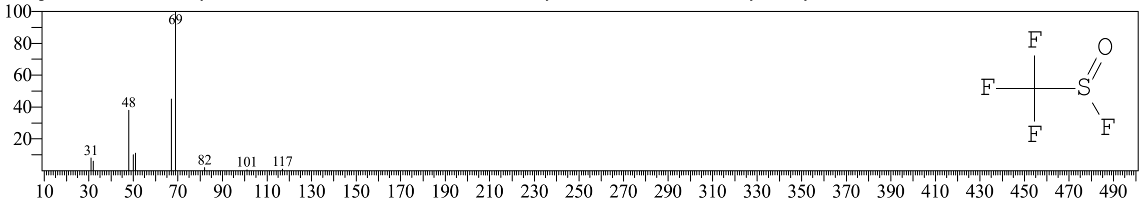

Hit#:5 Entry:10642 Library:NIST17-1.lib
SI:93 Formula:CF5P CAS:1112-04-5 MolWeight:138 RetIndex:0
CompName:Trifluoromethyldifluorophosphine $$ Trifluoromethylphosphonous difluoride # $$

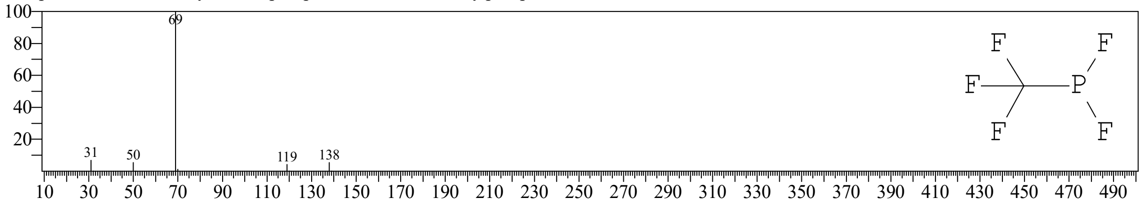

Qualitative Analysis Report

<< Target >>
Line#:40 R.Time:22.567(Scan#:4457) Retention Index:1238 MassPeaks:241
RawMode:Averaged 22.563-22.571(4456-4458) BasePeak:93.10(2837)
BG Mode:Calc. from Peak Group 1 - Event 1 Scan

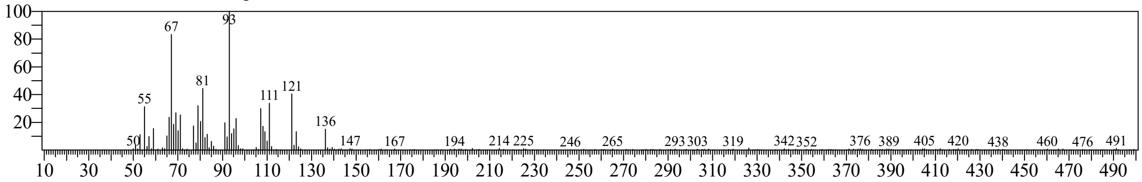

Hit#:1 Entry:5613 Library:NIST17-1.lib
SI:89 Formula:BrN3 CAS:13973-87-0 MolWeight:121 RetIndex:0
CompName:Bromine azide $$ Bromine azide (Br(N3)) $$ Bromine nitride $$ Nitrogen bromide (N3Br) $$

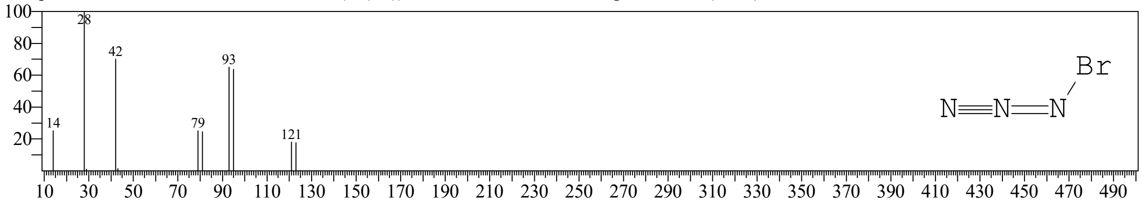

Hit#:2 Entry:2939 Library:NIST17-1.lib
SI:88 Formula:C6H8Si CAS:63878-65-9 MolWeight:108 RetIndex:0
CompName:Silabenzene,1-methyl- $$ 1-Methyl-1-silabenzene $$ 1-Methylsiline # $$

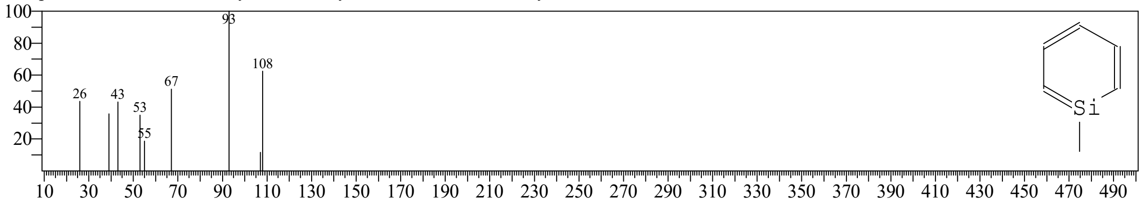

Hit#:3 Entry:38643 Library:NIST17-1.lib
SI:85 Formula:C9H14O2S CAS:83570-55-2 MolWeight:186 RetIndex:0
CompName:7-Thiaprotoadamantane 7,7-dioxide $$ 7-Thiaprotoadamantan 7,7-Dioxide $$ 2,6-Methanocyclopenta[b]thiopyran, hexahydro-, 1,1-dioxide $$

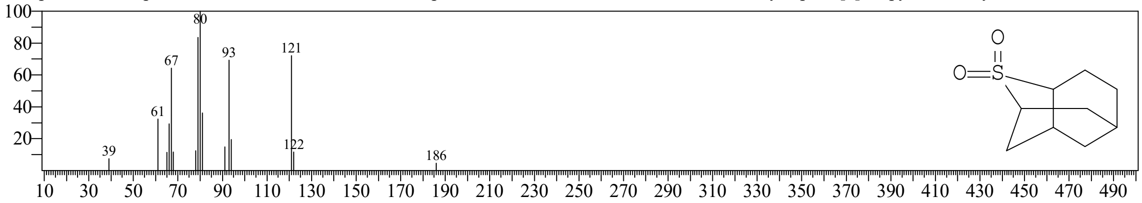

Hit#:4 Entry:24390 Library:NIST17-1.lib
SI:85 Formula:C12H20 CAS:1129-90-4 MolWeight:164 RetIndex:0
CompName:Cyclododecyne $$ 1-Cyclododecyne # $$

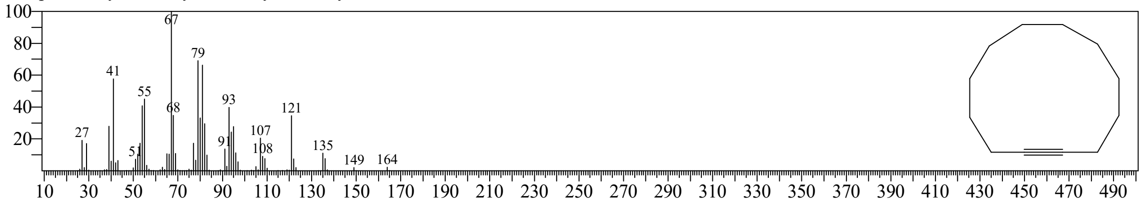

Hit#:5 Entry:18866 Library:NIST17-1.lib
SI:84 Formula:C10H18O CAS:13835-75-1 MolWeight:154 RetIndex:1230
CompName:3-Cyclohexene-1-ethanol, .beta.,4-dimethyl-, [S-(R*,S*)]- $$ p-Menth-1-en-9-ol, (4R,8S)-(+)- $$ (+)-p-Menta-1-en-9-ol $$ 3-Cyclohexene-1-et

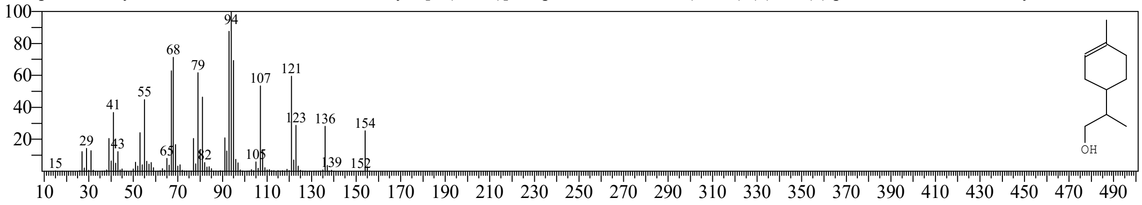

Qualitative Analysis Report

<< Target >>
Line#:41 R.Time:22.692(Scan#:4487) Retention Index:1241 MassPeaks:266
RawMode:Averaged 22.688-22.696(4486-4488) BasePeak:82.05(1795)
BG Mode:Calc. from Peak Group 1 - Event 1 Scan

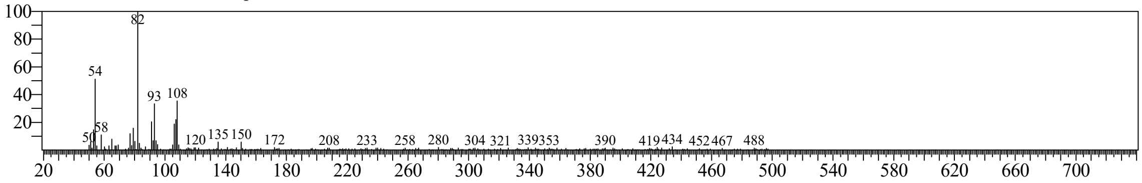

Hit#:1 Entry:294 Library:FFNSC 3.lib
SI:96 Formula:C10 H14 O CAS:99-49-0 MolWeight:150 RetIndex:1246
CompName:Carvone

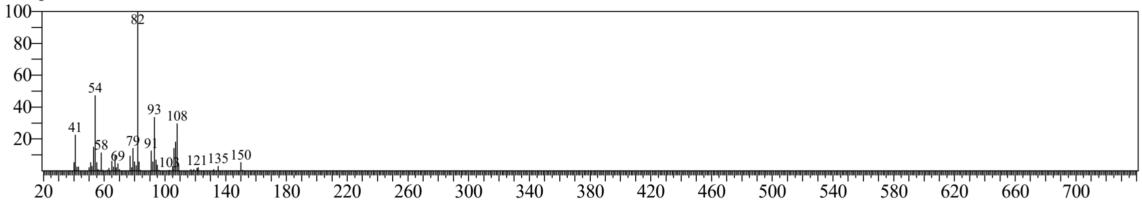

Hit#:2 Entry:3497 Library:NIST17-1.lib
SI:86 Formula:C2H6FO2P CAS:353-88-8 MolWeight:112 RetIndex:0
CompName:Methyl methylphosphonofluoridate $$ Phosphonofluoridic acid, methyl-, methyl ester $$ Methylphoshonic acid, fluoroanhydride, methyl ester $

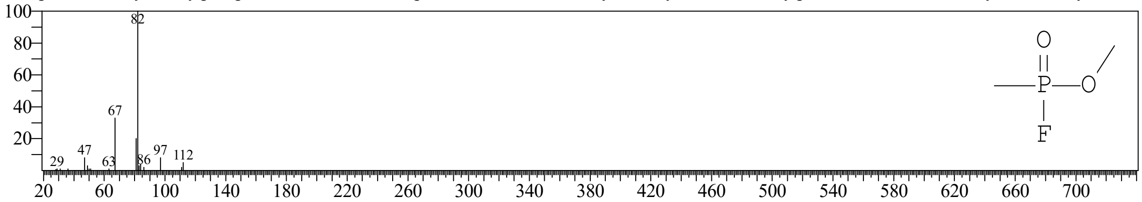

Hit#:3 Entry:13205 Library:NIST17-1.lib
SI:85 Formula:C6H9NOS CAS:0-00-0 MolWeight:143 RetIndex:1250
CompName:3-Cyano-thiopropionic acid, S-ethyl ester $$ S-Ethyl 3-cyanopropanethioate # $$

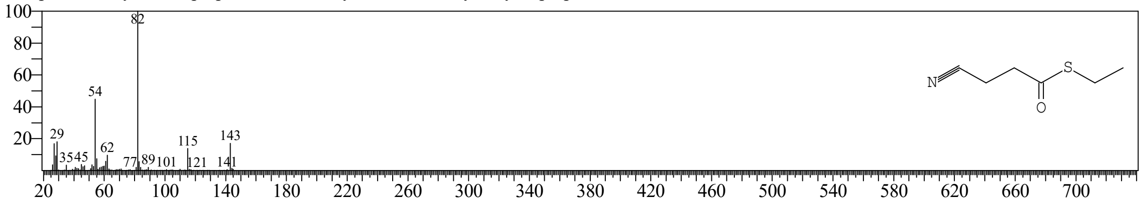

Hit#:4 Entry:55340 Library:NIST17-1.lib
SI:85 Formula:CAgF3S CAS:811-68-7 MolWeight:208 RetIndex:0
CompName:Methanethiol, trifluoro-, silver(1+) salt $$ Silver trifluoromethanethiolate $$

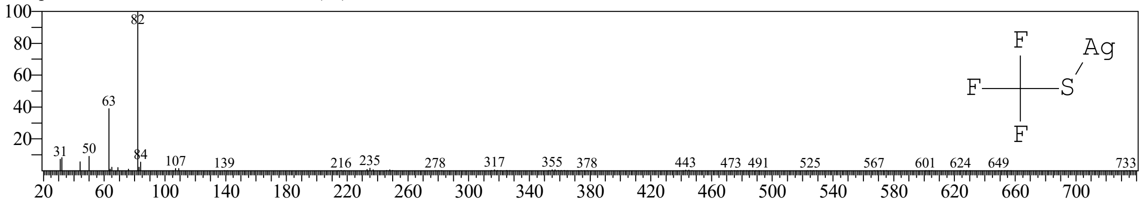

Hit#:5 Entry:6701 Library:NIST17-1.lib
SI:84 Formula:C6H6O3 CAS:89291-42-9 MolWeight:126 RetIndex:1234
CompName:2(5H)-Furanone, 5-(2-hydroxyethylidene)- $$ (5Z)-5-(2-Hydroxyethylidene)-2(5H)-furanone # $$

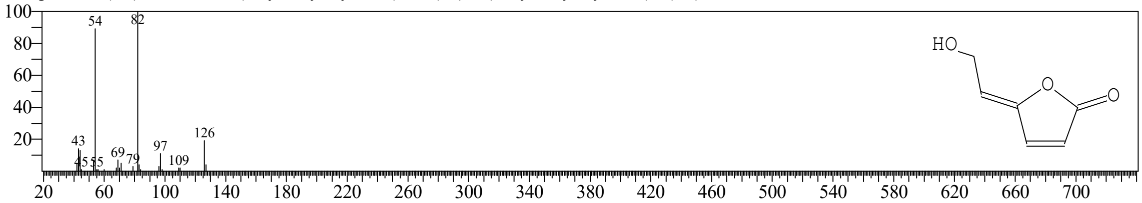

Qualitative Analysis Report

<< Target >>
Line#:42 R.Time:22.879(Scan#:4532) Retention Index:1245 MassPeaks:267
RawMode:Averaged 22.875-22.883(4531-4533) BasePeak:93.10(2815)
BG Mode:Calc. from Peak Group 1 - Event 1 Scan

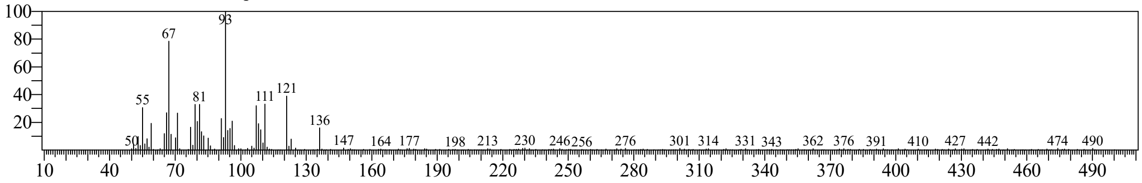

Hit#:1 Entry:5613 Library:NIST17-1.lib
SI:89 Formula:BrN3 CAS:13973-87-0 MolWeight:121 RetIndex:0
CompName:Bromine azide $$ Bromine azide (Br(N3)) $$ Bromine nitride $$ Nitrogen bromide (N3Br) $$

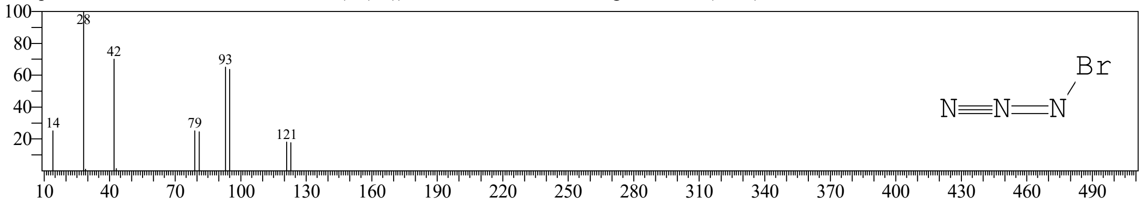

Hit#:2 Entry:2939 Library:NIST17-1.lib
SI:88 Formula:C6H8Si CAS:63878-65-9 MolWeight:108 RetIndex:0
CompName:Silabenzene,1-methyl- $$ 1-Methyl-1-silabenzene $$ 1-Methylsiline # $$

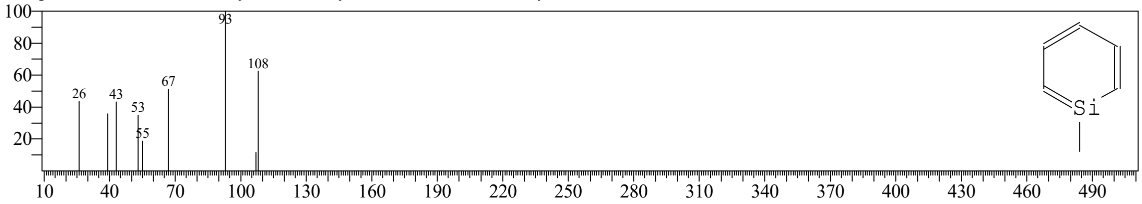

Hit#:3 Entry:18806 Library:NIST17-1.lib
SI:85 Formula:C10H18O CAS:18479-64-6 MolWeight:154 RetIndex:1250
CompName:Shisool $$ Cyclohexanemethanol, 4-(1-methylethenyl)- $$ p-Menth-8-en-7-ol (8CI) $$ (4-Isopropenylcyclohexyl)methanol $$ Dihydroperillyl a

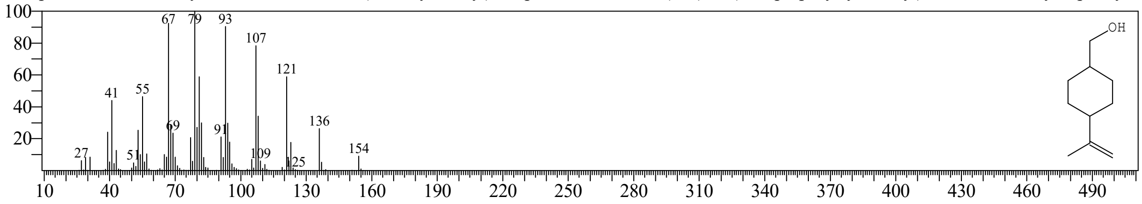

Hit#:4 Entry:18849 Library:NIST17-1.lib
SI:84 Formula:C10H18O CAS:22451-48-5 MolWeight:154 RetIndex:1250
CompName:trans-Shisool $$ (4-Isopropenylcyclohexyl)methanol # $$

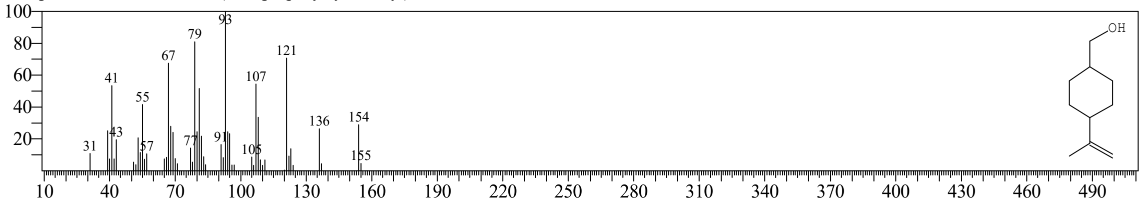

Hit#:5 Entry:24390 Library:NIST17-1.lib
SI:84 Formula:C12H20 CAS:1129-90-4 MolWeight:164 RetIndex:0
CompName:Cyclododecyne $$ 1-Cyclododecyne # $$

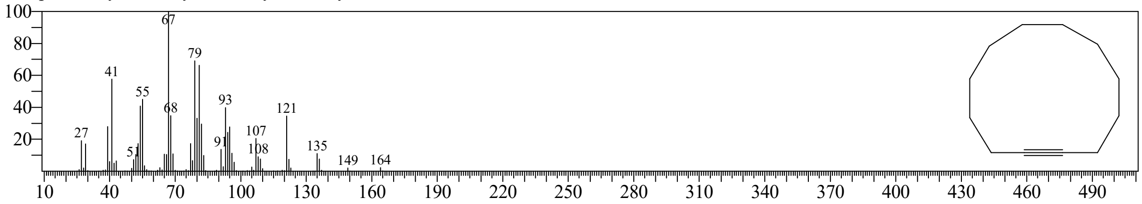

Qualitative Analysis Report

<< Target >>
Line#:43 R.Time:23.012(Scan#:4564) Retention Index:1248 MassPeaks:315
RawMode:Averaged 23.008-23.017(4563-4565) BasePeak:69.10(281276)
BG Mode:Calc. from Peak Group 1 - Event 1 Scan

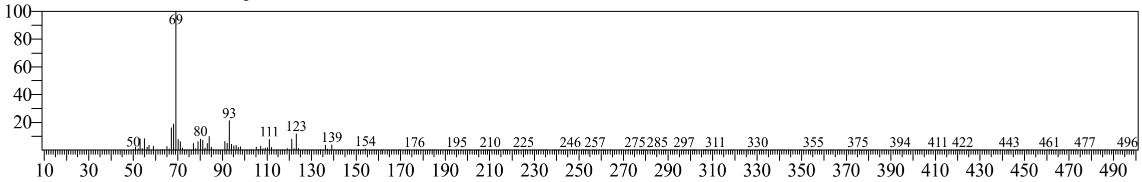

Hit#:1 Entry:228 Library:NIST17-1.lib
SI:99 Formula:C2H3N3 CAS:288-36-8 MolWeight:69 RetIndex:0
CompName:1H-1,2,3-Triazole $$ V-triazole $$ Osotriazole $$ Pyrrodiazole $$ Triazacyclopentadiene $$ 1,2,3-Triazole $$

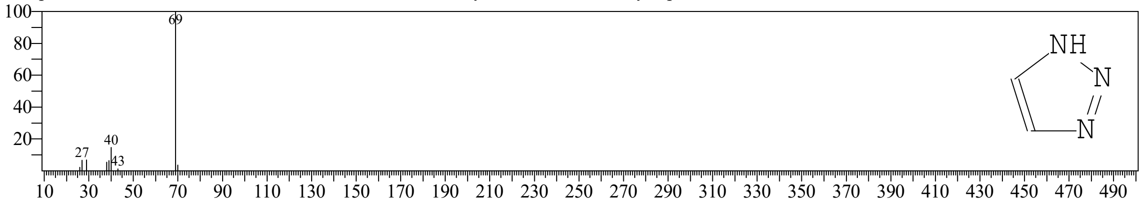

Hit#:2 Entry:981 Library:NIST17-1.lib
SI:96 Formula:CF4 CAS:75-73-0 MolWeight:88 RetIndex:0
CompName:Tetrafluoromethane $$ Carbon tetrafluoride $$ Methane, tetrafluoro- $$ Arcton 0 $$ Carbon fluoride (CF4) $$ Freon 14 $$ FC 14 $$ Perfluoro

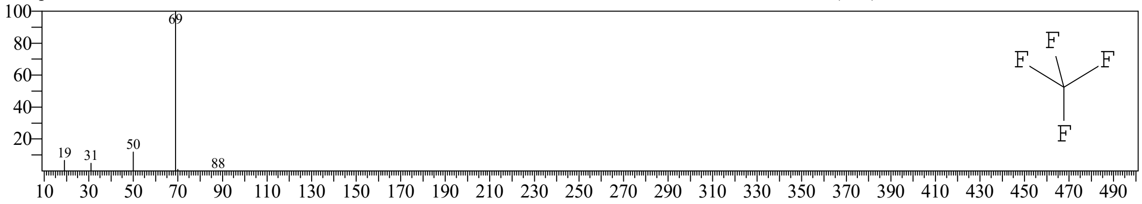

Hit#:3 Entry:169 Library:FFNSC 3.lib
SI:95 Formula:C10 H18 O CAS:106-24-1 MolWeight:154 RetIndex:1255
CompName:Geraniol

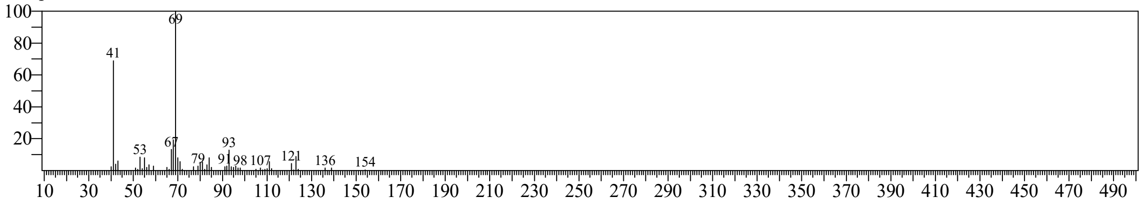

Hit#:4 Entry:10642 Library:NIST17-1.lib
SI:94 Formula:CF5P CAS:1112-04-5 MolWeight:138 RetIndex:0
CompName:Trifluoromethyldifluorophosphine $$ Trifluoromethylphosphonous difluoride # $$

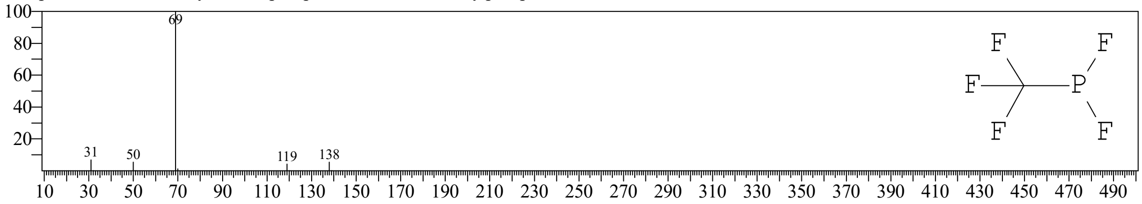

Hit#:5 Entry:10455 Library:NIST17-1.lib
SI:93 Formula:CClF4N CAS:13880-72-3 MolWeight:137 RetIndex:0
CompName:N-Chloro-N-fluoro-trifluoromethylamine $$ N-Chloro-N-tetrafluoromethanamine # $$

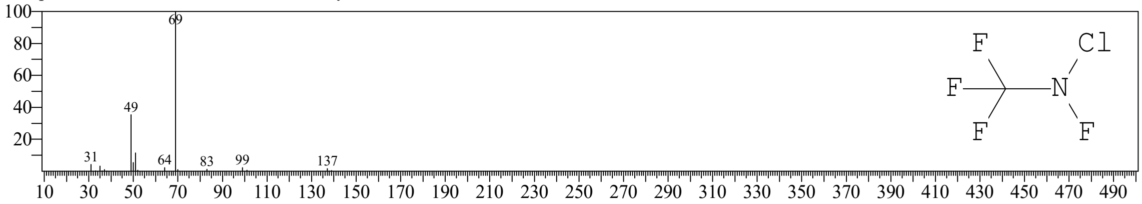

Qualitative Analysis Report

<< Target >>
Line#:44 R.Time:23.179(Scan#:4604) Retention Index:1252 MassPeaks:243
RawMode:Averaged 23.175-23.183(4603-4605) BasePeak:81.10(426)
BG Mode:Calc. from Peak Group 1 - Event 1 Scan

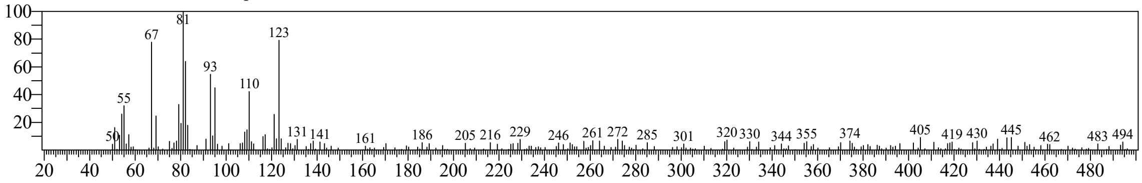

Hit#:1 Entry:36236 Library:NIST17-1.lib
SI:90 Formula:C11H18O2 CAS:0-00-0 MolWeight:182 RetIndex:1259
CompName:Methyl 5-methyl-2-ethenyl-cyclohexane-1-carboxylate $$ Methyl 5-methyl-2-vinylcyclohexanecarboxylate # $$

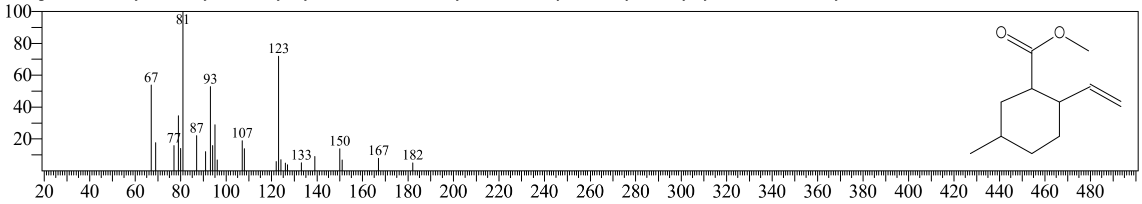

Hit#:2 Entry:2266 Library:FFNSC 3.lib
SI:86 Formula:C10 H18 O CAS:15358-92-6 MolWeight:154 RetIndex:1254
CompName:Myrtanol <cis->

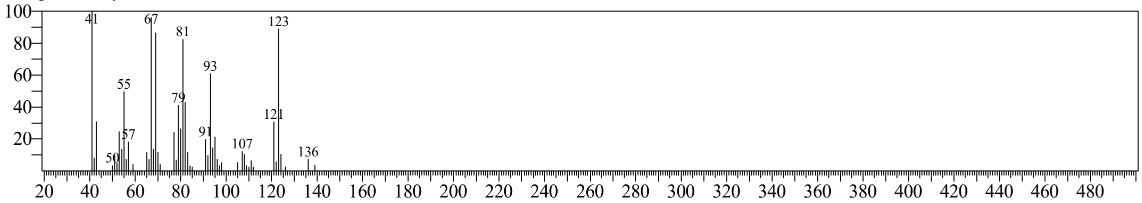

Hit#:3 Entry:735 Library:FFNSC 3.lib
SI:86 Formula:C10 H18 O2 CAS:21722-83-8 MolWeight:170 RetIndex:1251
CompName:Cyclohexaneethyl acetate

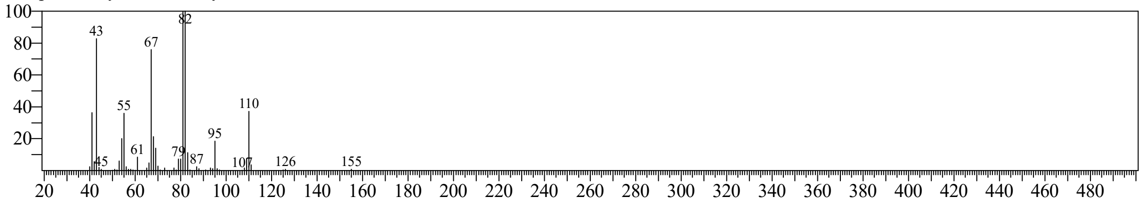

Hit#:4 Entry:45275 Library:NIST17-1.lib
SI:85 Formula:C7H9N5O2 CAS:2278-16-2 MolWeight:195 RetIndex:0
CompName:3H-v-Triazolo(4,5-d)pyrimidine-5,7(4H,6H)-dione, 3,4,6-trimethyl- $$ 1,3,9-Trimethyl-8-azaxanthin $$ 3,4,6-Trimethyl-3H-v-triazolo(4,5-d)py

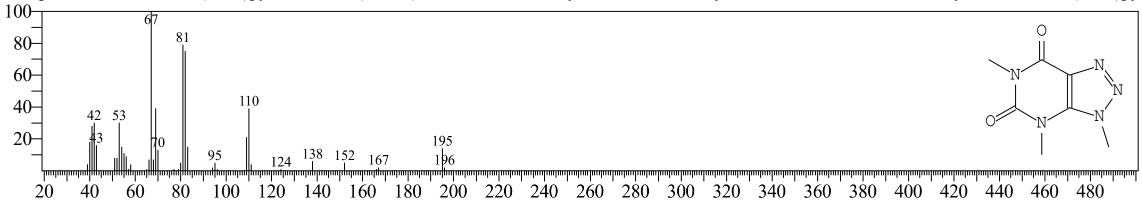

Hit#:5 Entry:3162 Library:NIST17-1.lib
SI:84 Formula:C5H6N2O CAS:17758-50-8 MolWeight:110 RetIndex:0
CompName:Pyrimidine, 5-methyl-, 1-oxide $$ 5-Methylpyrimidine 1-oxide $$

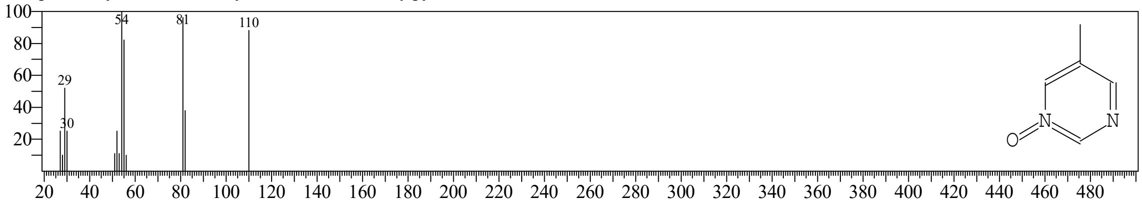

Qualitative Analysis Report

<< Target >>
Line#:45 R.Time:23.771(Scan#:4746) Retention Index:1265 MassPeaks:270
RawMode:Averaged 23.767-23.775(4745-4747) BasePeak:69.10(45708)
BG Mode:Calc. from Peak Group 1 - Event 1 Scan

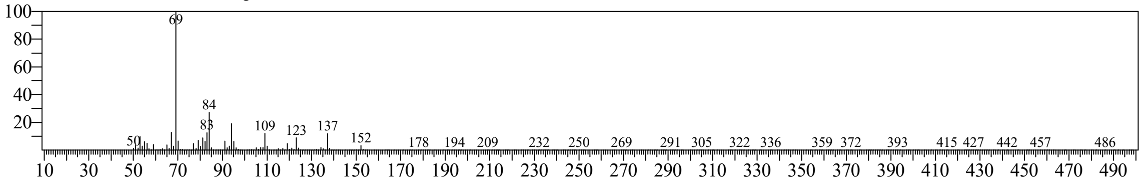

Hit#:1 Entry:228 Library:NIST17-1.lib
SI:99 Formula:C2H3N3 CAS:288-36-8 MolWeight:69 RetIndex:0
CompName:1H-1,2,3-Triazole $$ V-triazole $$ Osotriazole $$ Pyrrodiazole $$ Triazacyclopentadiene $$ 1,2,3-Triazole $$

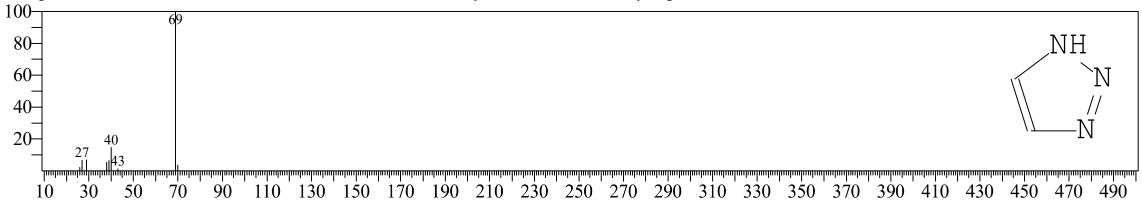

Hit#:2 Entry:10642 Library:NIST17-1.lib
SI:96 Formula:CF5P CAS:1112-04-5 MolWeight:138 RetIndex:0
CompName:Trifluoromethyldifluorophosphine $$ Trifluoromethylphosphonous difluoride # $$

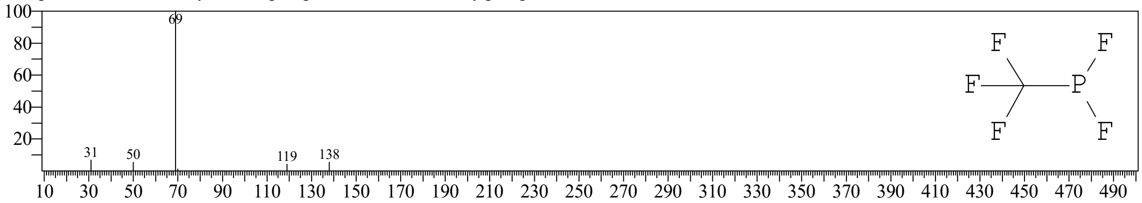

Hit#:3 Entry:981 Library:NIST17-1.lib
SI:96 Formula:CF4 CAS:75-73-0 MolWeight:88 RetIndex:0
CompName:Tetrafluoromethane $$ Carbon tetrafluoride $$ Methane, tetrafluoro- $$ Arcton 0 $$ Carbon fluoride (CF4) $$ Freon 14 $$ FC 14 $$ Perfluoro

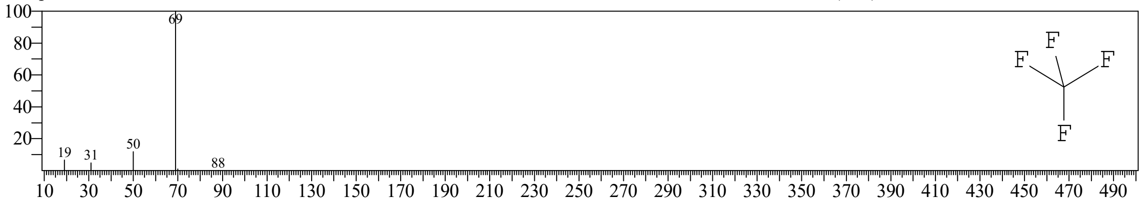

Hit#:4 Entry:1003 Library:FFNSC 3.lib
SI:94 Formula:C10 H16 O CAS:141-27-5 MolWeight:152 RetIndex:1268
CompName:Geranial

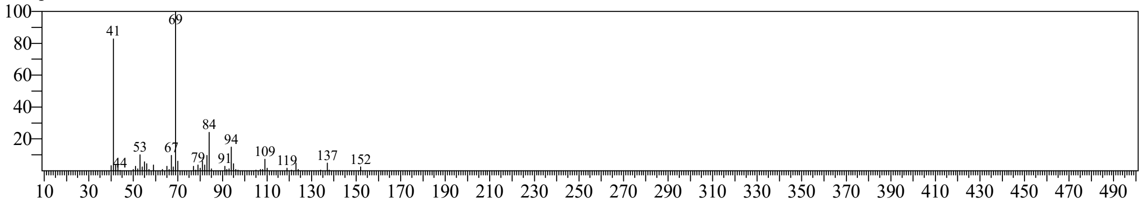

Hit#:5 Entry:2524 Library:NIST17-1.lib
SI:92 Formula:CClF3 CAS:75-72-9 MolWeight:104 RetIndex:0
CompName:Chlorotrifluoromethane $$ Monochlorotrifluoromethane $$ Methane, chlorotrifluoro- $$ Arcton 3 $$ F 13 $$ Freon 13 $$ FC 13 $$ Genetron

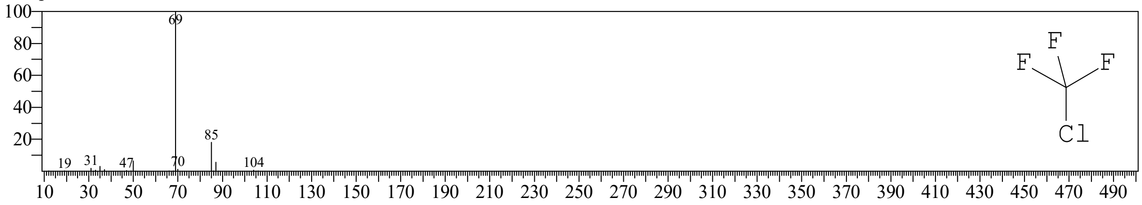

Qualitative Analysis Report

<< Target >>
Line#:46 R.Time:24.400(Scan#:4897) Retention Index:1278 MassPeaks:254
RawMode:Averaged 24.396-24.404(4896-4898) BasePeak:135.15(1785)
BG Mode:Calc. from Peak Group 1 - Event 1 Scan

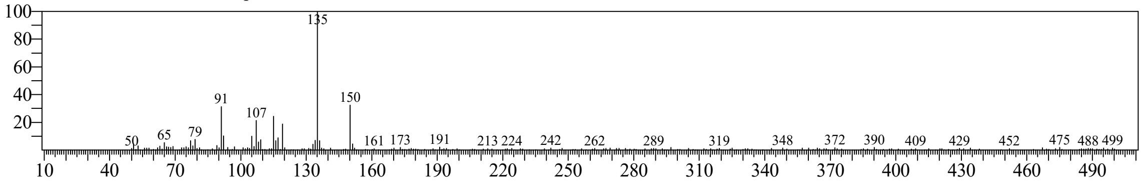

Hit#:1 Entry:215434 Library:NIST17-1.lib
SI:90 Formula:C20H20N4O4 CAS:19226-32-5 MolWeight:380 RetIndex:0
CompName:Benzoic acid, 4-methoxy-, anhydride with N-(4,5-dimethyl-1H-1,2,3-triazol-1-yl)-4-methoxybenzenecarboximidic acid $$ p-Anisic acid, anhydr

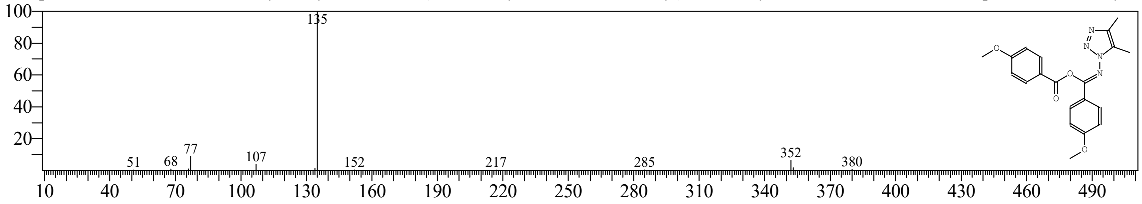

Hit#:2 Entry:63781 Library:NIST17-1.lib
SI:89 Formula:C5H13Cl2N2OP CAS:58993-59-2 MolWeight:218 RetIndex:0
CompName:Tetramethyldiamidodichloromethylphosphonate $$ p-(Dichloromethyl)-N,N,N',N'-tetramethylphosphonic diamide # $$

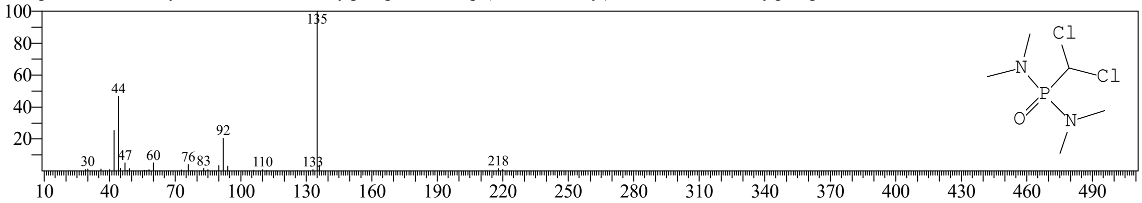

Hit#:3 Entry:54964 Library:NIST17-1.lib
SI:88 Formula:C11H13NOS CAS:0-00-0 MolWeight:207 RetIndex:0
CompName:4-n-Butoxyphenyl isocyanate

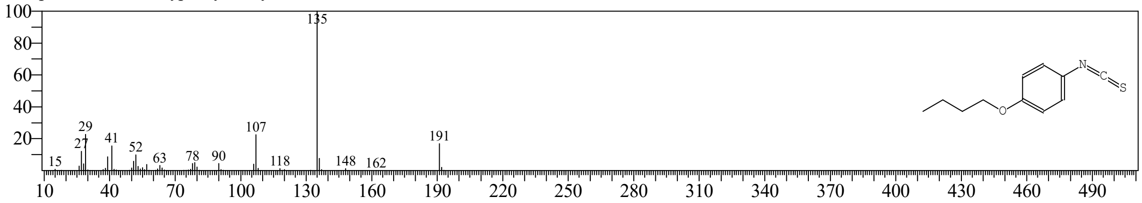

Hit#:4 Entry:247746 Library:NIST17-1.lib
SI:87 Formula:C19H20BrNO5S CAS:0-00-0 MolWeight:453 RetIndex:0
CompName:(7-Bromo-3-isopropyl-1,1-dioxido-3,4-dihydro-2H-benzo[b][1,4,5]oxathiazepin-2-yl)(3-methoxyphenyl)methanone $$ SMR001788269 $$ ML

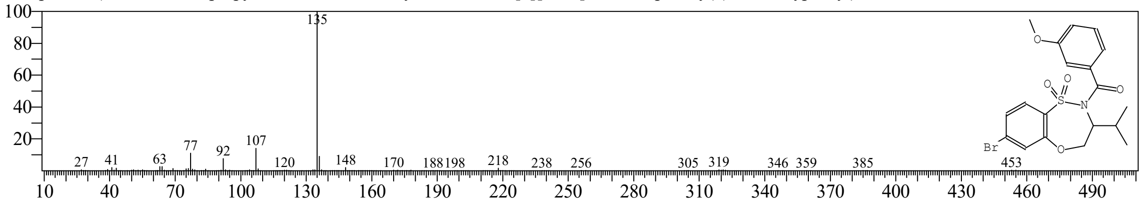

Hit#:5 Entry:16516 Library:NIST17-1.lib
SI:87 Formula:C10H14O CAS:536-60-7 MolWeight:150 RetIndex:1284
CompName:p-Cymen-7-ol $$ Cumic alcohol $$ Benzenemethanol, 4-(1-methylethyl)- $$ p-Isopropylbenzyl alcohol $$ Cuminic alcohol $$ Cuminol $$ Cun

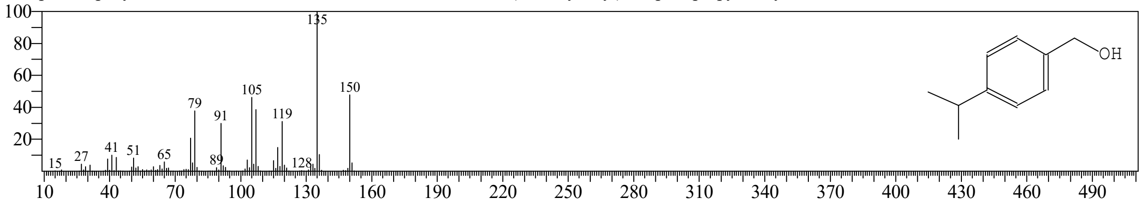

Qualitative Analysis Report

<< Target >>
Line#:47   R.Time:24.579(Scan#:4940) Retention Index:1282 MassPeaks:332
RawMode:Averaged 24.575-24.583(4939-4941) BasePeak:95.10(50205)
BG Mode:Calc. from Peak Group 1 - Event 1 Scan

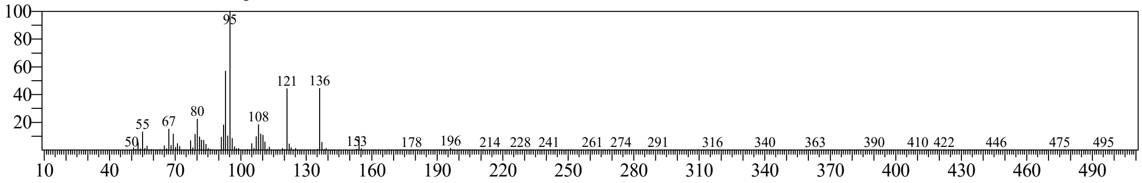

Hit#:1 Entry:46591 Library:NIST17-1.lib
SI:97 Formula:C12H20O2 CAS:76-49-3 MolWeight:196 RetIndex:1277
CompName:Bornyl acetate $$ Bicyclo[2.2.1]heptan-2-ol, 1,7,7-trimethyl-, acetate, endo- $$ Borneol, acetate $$ Bornyl acetic ether $$ 2-Camphanol acetate

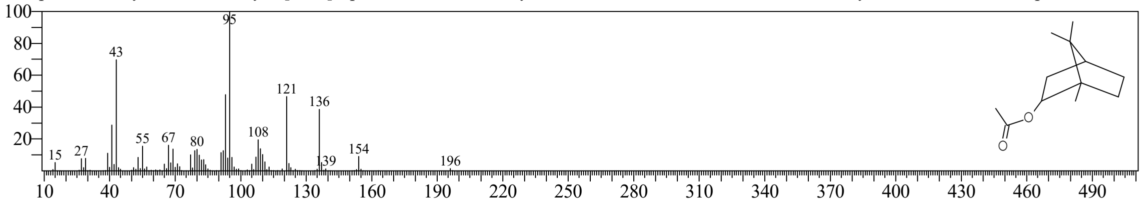

Hit#:2 Entry:991 Library:FFNSC 3.lib
SI:97 Formula:C12 H20 O2 CAS:92618-89-8 MolWeight:196 RetIndex:1285
CompName:Bornyl acetate

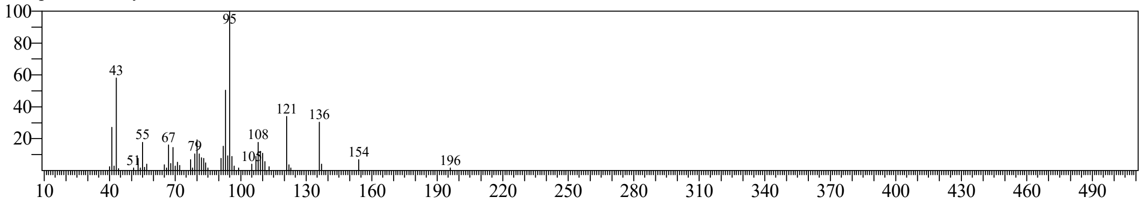

Hit#:3 Entry:1629 Library:FFNSC 3.lib
SI:96 Formula:C12 H20 O2 CAS:125-12-2 MolWeight:196 RetIndex:1287
CompName:Isobornyl acetate

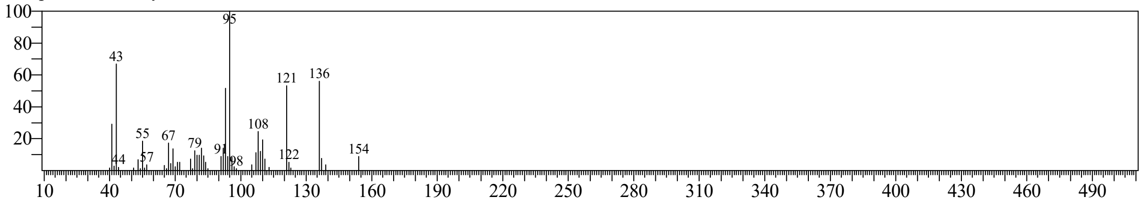

Hit#:4 Entry:46596 Library:NIST17-1.lib
SI:95 Formula:C12H20O2 CAS:5655-61-8 MolWeight:196 RetIndex:1277
CompName:Bicyclo[2.2.1]heptan-2-ol, 1,7,7-trimethyl-, acetate, (1S-endo)- $$ Borneol, acetate, (1S,2R,4S)-(-)- $$ (-)-Bornyl acetate $$ L-.alpha.-bornyl ac

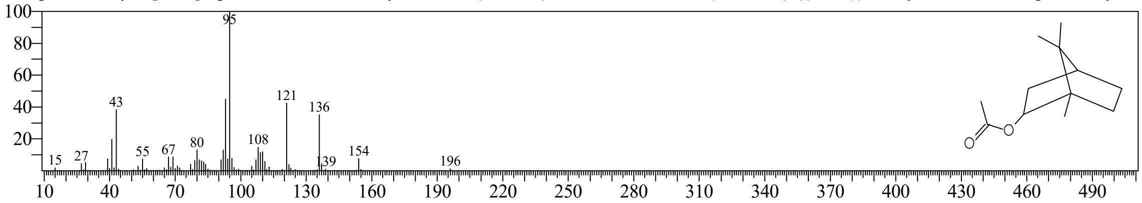

Hit#:5 Entry:36266 Library:NIST17-1.lib
SI:93 Formula:C11H18O2 CAS:1200-67-5 MolWeight:182 RetIndex:1275
CompName:Isobornyl formate $$ Bicyclo[2.2.1]heptan-2-ol, 1,7,7-trimethyl-, formate, exo- $$ 1,7,7-Trimethylbicyclo[2.2.1]hept-2-yl formate, exo- $$ Isob

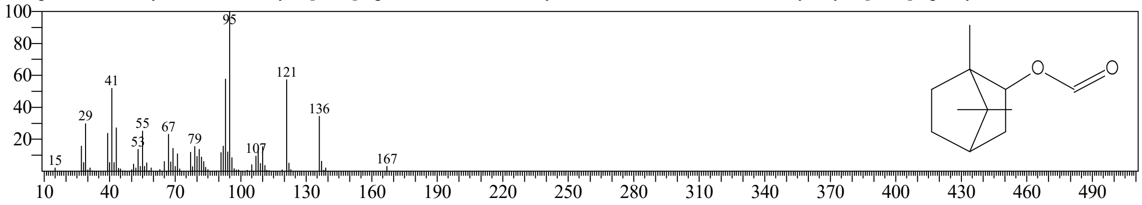

Qualitative Analysis Report

<< Target >>
Line#:48 R.Time:24.850(Scan#:5005) Retention Index:1288 MassPeaks:253
RawMode:Averaged 24.846-24.854(5004-5006) BasePeak:135.15(1381)
BG Mode:Calc. from Peak Group 1 - Event 1 Scan

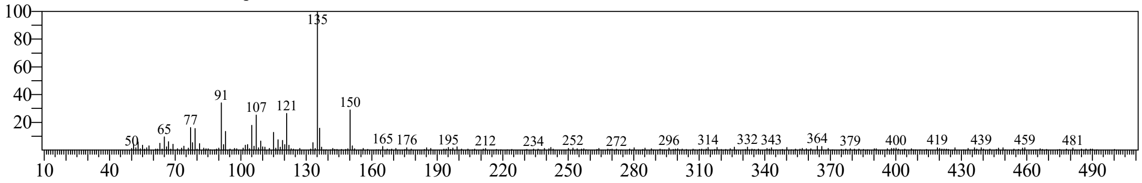

Hit#:1 Entry:215434 Library:NIST17-1.lib
SI:91 Formula:C20H20N4O4 CAS:19226-32-5 MolWeight:380 RetIndex:0
CompName:Benzoic acid, 4-methoxy-, anhydride with N-(4,5-dimethyl-1H-1,2,3-triazol-1-yl)-4-methoxybenzenecarboximidic acid $$ p-Anisic acid, anhydr

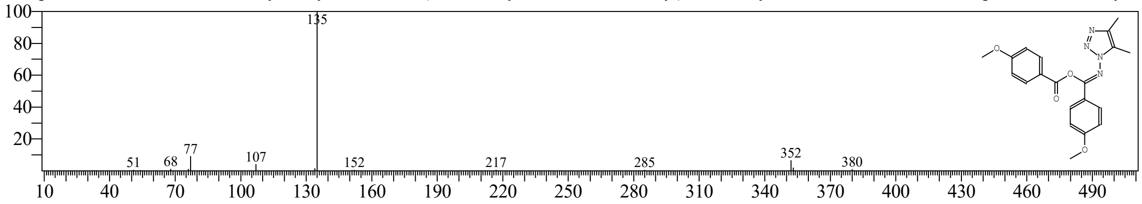

Hit#:2 Entry:634 Library:FFNSC 3.lib
SI:90 Formula:C10 H14 O CAS:89-83-8 MolWeight:150 RetIndex:1293
CompName:Thymol

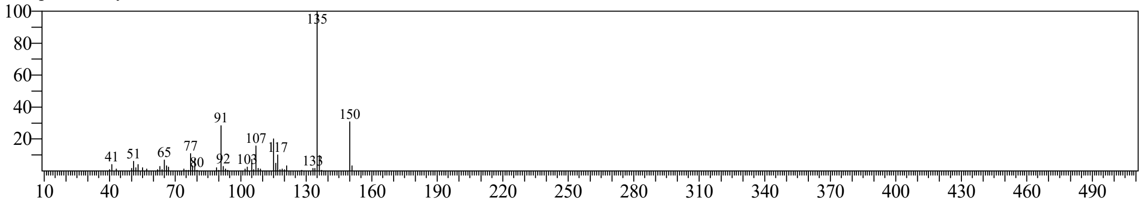

Hit#:3 Entry:157209 Library:NIST17-1.lib
SI:88 Formula:C10H15Cl3Ge CAS:0-00-0 MolWeight:314 RetIndex:0
CompName:Trichlor-(1,2,3,4,5-pentamethylcyclopenta-2,4-dienyl)-german $$ Trichloro(1,2,3,4,5-pentamethyl-2,4-cyclopentadien-1-yl)germane # $$

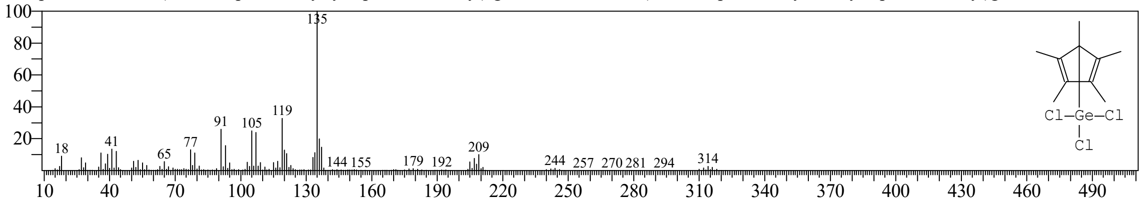

Hit#:4 Entry:16516 Library:NIST17-1.lib
SI:88 Formula:C10H14O CAS:536-60-7 MolWeight:150 RetIndex:1284
CompName:p-Cymen-7-ol $$ Cumic alcohol $$ Benzenemethanol, 4-(1-methylethyl)- $$ p-Isopropylbenzyl alcohol $$ Cuminic alcohol $$ Cuminol $$ Cun

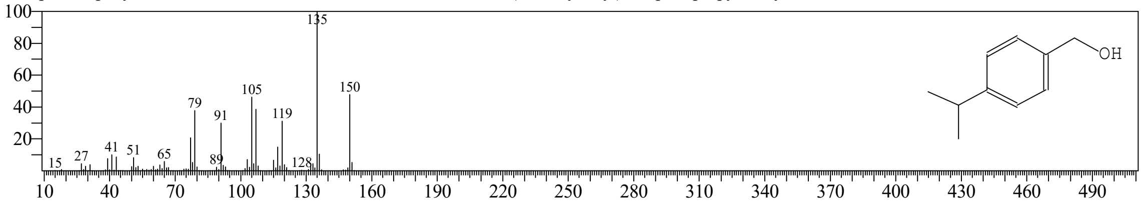

Hit#:5 Entry:95249 Library:NIST17-1.lib
SI:87 Formula:C10H15Cl2OP CAS:0-00-0 MolWeight:252 RetIndex:0
CompName:Phosphonic dichloride, 1-adamantyl- $$ 1-Adamantylphosphonic dichloride # $$

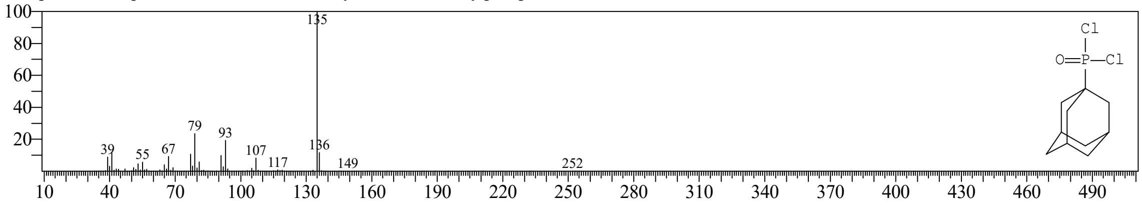

<< Target >>
Line#:49 R.Time:25.163(Scan#:5080) Retention Index:1295 MassPeaks:243
RawMode:Averaged 25.158-25.167(5079-5081) BasePeak:135.15(1409)
BG Mode:Calc. from Peak Group 1 - Event 1 Scan

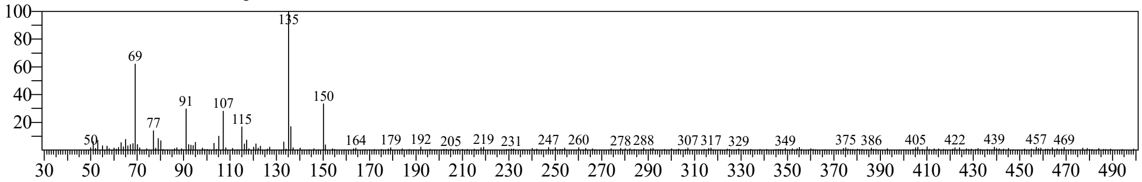

Hit#:1 Entry:634 Library:FFNSC 3.lib
SI:94 Formula:C10 H14 O CAS:89-83-8 MolWeight:150 RetIndex:1293
CompName:Thymol

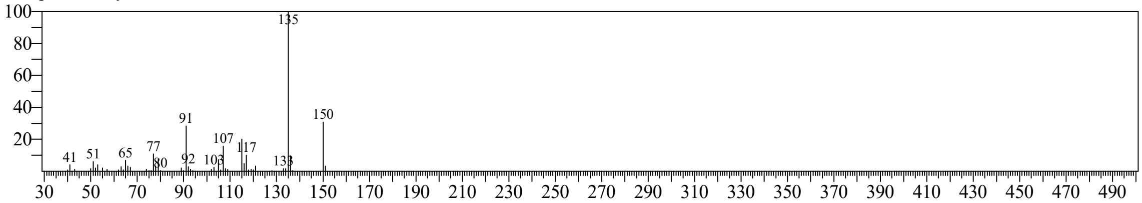

Hit#:2 Entry:215434 Library:NIST17-1.lib
SI:90 Formula:C20H20N4O4 CAS:19226-32-5 MolWeight:380 RetIndex:0
CompName:Benzoic acid, 4-methoxy-, anhydride with N-(4,5-dimethyl-1H-1,2,3-triazol-1-yl)-4-methoxybenzenecarboximidic acid $$ p-Anisic acid, anhydr

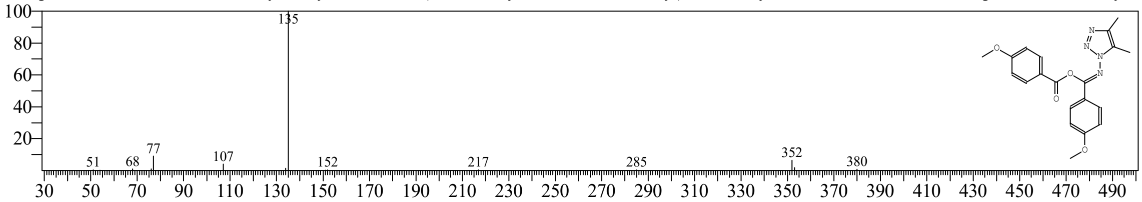

Hit#:3 Entry:981 Library:FFNSC 3.lib
SI:89 Formula:C10 H14 O CAS:499-75-2 MolWeight:150 RetIndex:1300
CompName:Carvacrol

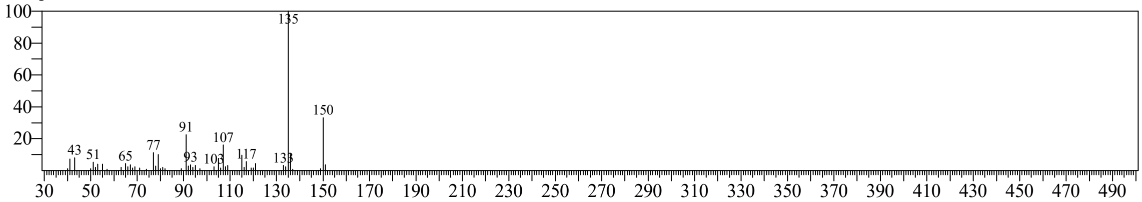

Hit#:4 Entry:63947 Library:NIST17-1.lib
SI:88 Formula:C9H10N6O CAS:0-00-0 MolWeight:218 RetIndex:0
CompName:1H-1,2,3,4-Tetrazole-1-acetamide, N-(4-methyl-2-pyridinyl)-

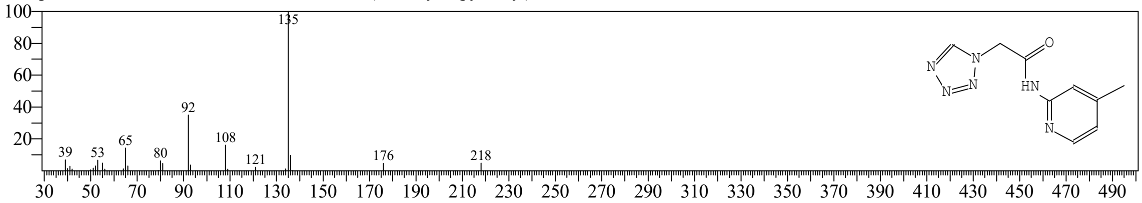

Hit#:5 Entry:35 Library:FFNSC 3.lib
SI:87 Formula:C10 H14 O CAS:98-54-4 MolWeight:150 RetIndex:1298
CompName:Phenol <4-tert-butyl->

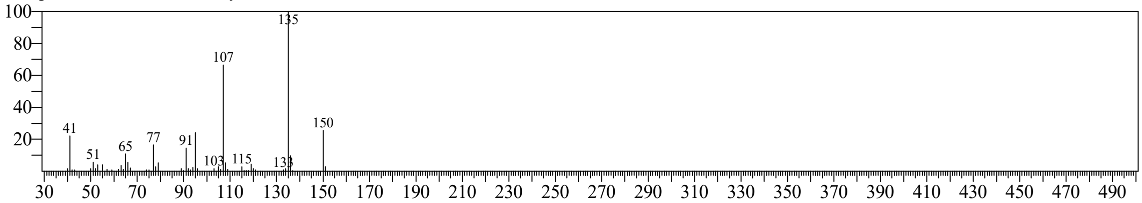

Qualitative Analysis Report

<< Target >>
Line#:50   R.Time:26.933(Scan#:5505) Retention Index:1335 MassPeaks:255
RawMode:Averaged 26.929-26.938(5504-5506) BasePeak:150.15(3439)
BG Mode:Calc. from Peak Group 1 - Event 1 Scan

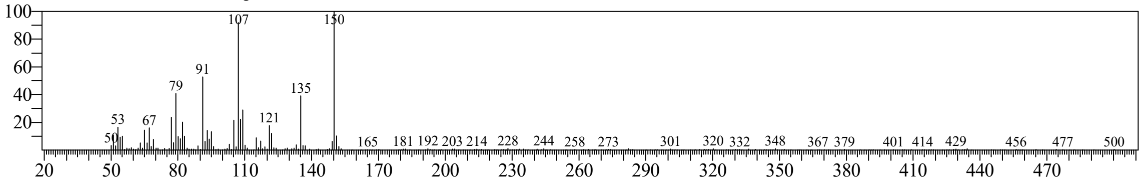

Hit#:1 Entry:87777 Library:NIST17-1.lib
SI:89 Formula:C10H12O2Se CAS:112422-89-6 MolWeight:244 RetIndex:0
CompName:3-Selenetanol, 3-(4-methoxyphenyl)- $$ 3-(4-Methoxyphenyl)-3-selenetanol # $$

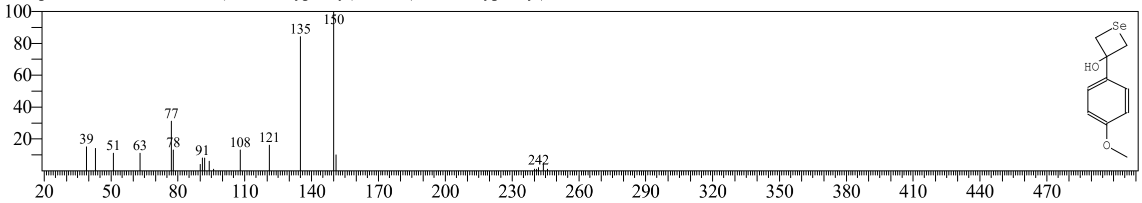

Hit#:2 Entry:33195 Library:NIST17-1.lib
SI:88 Formula:C10H14N2O CAS:14487-70-8 MolWeight:178 RetIndex:0
CompName:3-Diazo-1,7,7-trimethylbicyclo[2.2.1]heptan-2-one

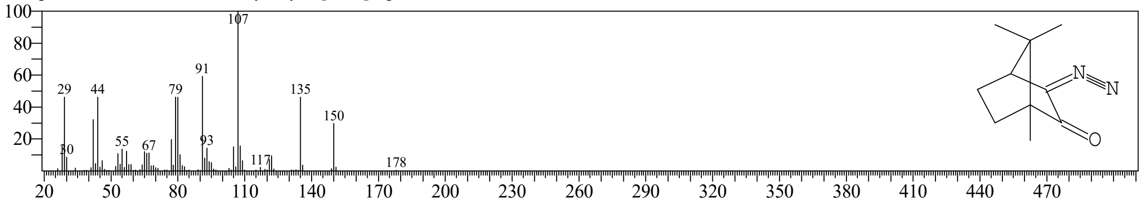

Hit#:3 Entry:16296 Library:NIST17-1.lib
SI:84 Formula:C9H10O2 CAS:21834-60-6 MolWeight:150 RetIndex:1344
CompName:2H-1-Benzopyran-3-ol, 3,4-dihydro- $$ 3-Chromanol # $$

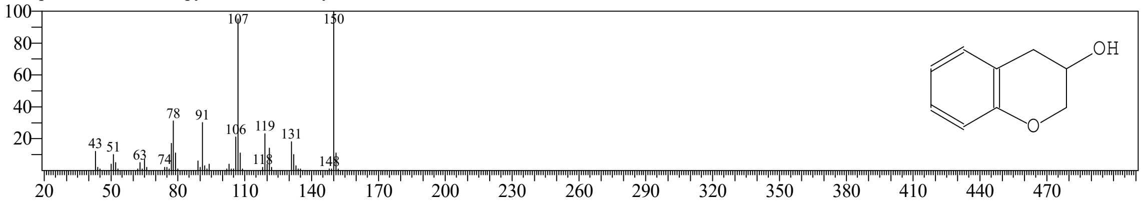

Hit#:4 Entry:25862 Library:NIST17-1.lib
SI:83 Formula:C8H9NOS CAS:17420-02-9 MolWeight:167 RetIndex:0
CompName:Benzenamine, 2,6-dimethyl-N-sulfinyl- $$ 2,6-Xylidine, N-sulfinyl- $$ 2,6-Dimethyl-N-sulfinylaniline $$

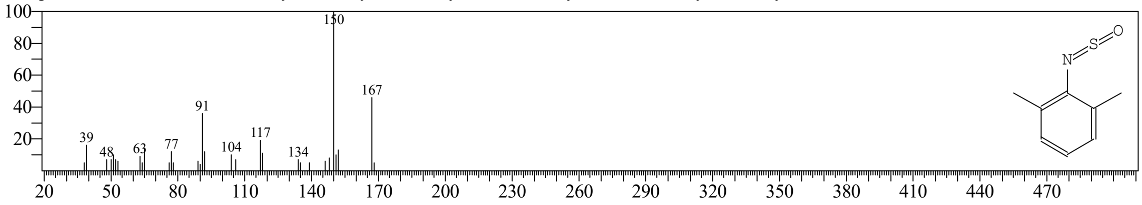

Hit#:5 Entry:26991 Library:NIST17-1.lib
SI:82 Formula:C11H20O CAS:14551-31-6 MolWeight:168 RetIndex:1325
CompName:Spiro[4.5]decan-6-ol, 6-methyl- $$ 6-Methylspiro[4.5]decan-6-ol # $$

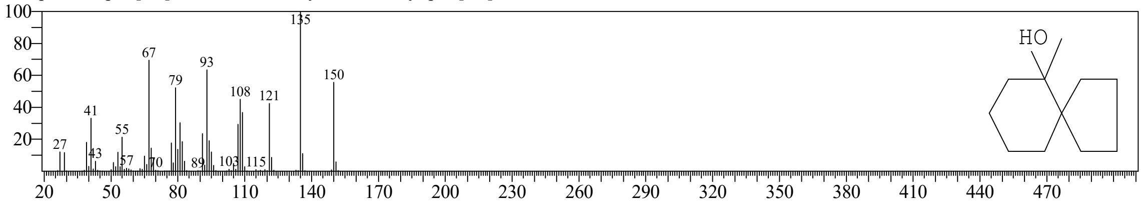

Qualitative Analysis Report

<< Target >>
Line#:51 R.Time:27.525(Scan#:5647) Retention Index:1349 MassPeaks:278
RawMode:Averaged 27.521-27.529(5646-5648) BasePeak:164.10(1326)
BG Mode:Calc. from Peak Group 1 - Event 1 Scan

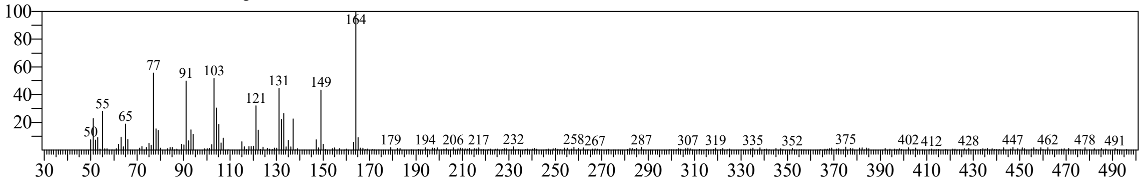

Hit#:1 Entry:160 Library:FFNSC 3.lib
SI:95 Formula:C10 H12 O2 CAS:97-53-0 MolWeight:164 RetIndex:1357
CompName:Eugenol

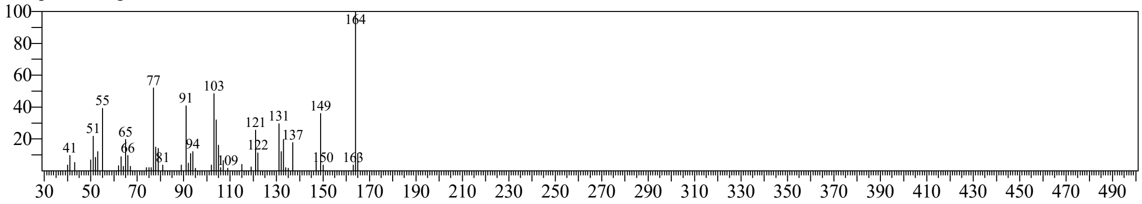

Hit#:2 Entry:24202 Library:NIST17-1.lib
SI:86 Formula:C10H12S CAS:24309-31-7 MolWeight:164 RetIndex:1347
CompName:Allyl (2-methylphenyl) sulfide $$ 1-(Allylsulfanyl)-2-methylbenzene # $$

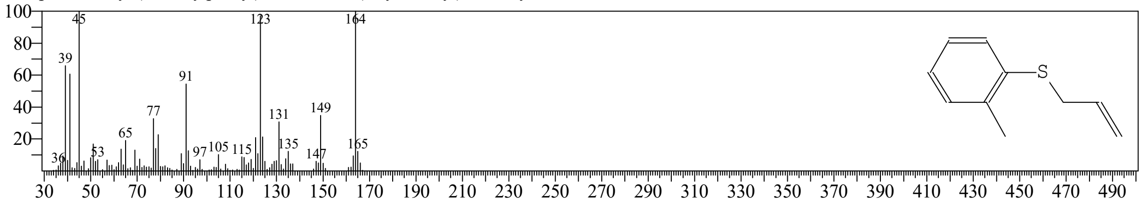

Hit#:3 Entry:24205 Library:NIST17-1.lib
SI:84 Formula:C10H12S CAS:1516-28-5 MolWeight:164 RetIndex:1347
CompName:Allyl(4-methylphenyl) sulfide $$ 1-(Allylsulfanyl)-4-methylbenzene # $$

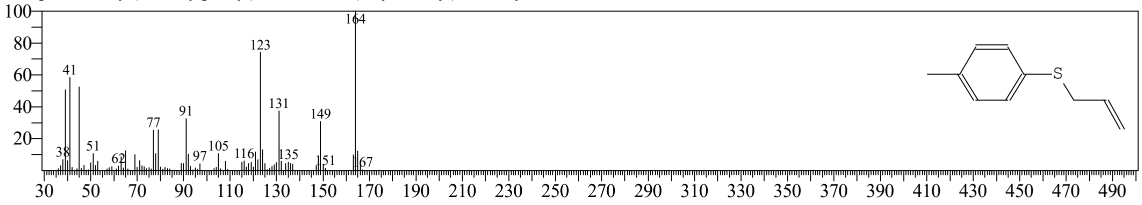

Hit#:4 Entry:24241 Library:NIST17-1.lib
SI:83 Formula:C10H16N2 CAS:24340-88-3 MolWeight:164 RetIndex:1353
CompName:p-Phenylenediamine, N'-ethyl-N,N-dimethyl-

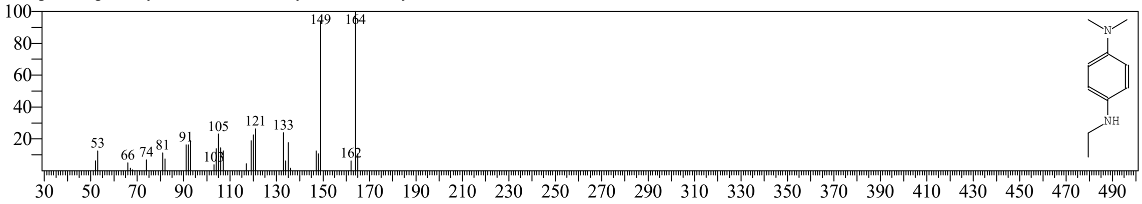

Hit#:5 Entry:23806 Library:NIST17-1.lib
SI:83 Formula:C7H4N2O3 CAS:57948-15-9 MolWeight:164 RetIndex:0
CompName:Benzofuroxan-6-carboxaldehyde $$ 2,1,3-Benzoxadiazole-5-carbaldehyde 3-oxide # $$

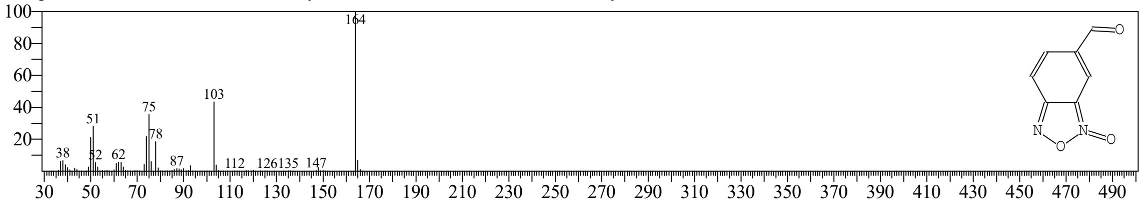

Qualitative Analysis Report

<< Target >>
Line#:52 R.Time:28.679(Scan#:5924) Retention Index:1375 MassPeaks:275
RawMode:Averaged 28.675-28.683(5923-5925) BasePeak:69.10(10771)
BG Mode:Calc. from Peak Group 1 - Event 1 Scan

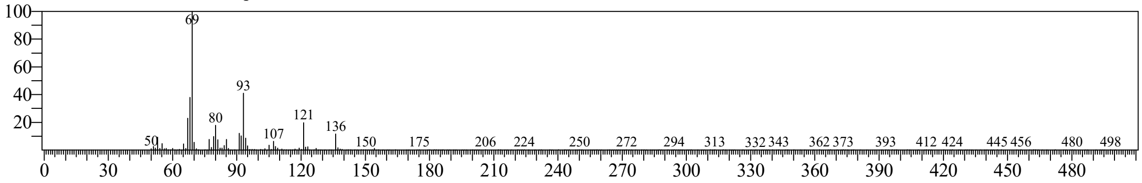

Hit#:1 Entry:228 Library:NIST17-1.lib
SI:100 Formula:C2H3N3 CAS:288-36-8 MolWeight:69 RetIndex:0
CompName:1H-1,2,3-Triazole $$ V-triazole $$ Osotriazole $$ Pyrrodiazole $$ Triazacyclopentadiene $$ 1,2,3-Triazole $$

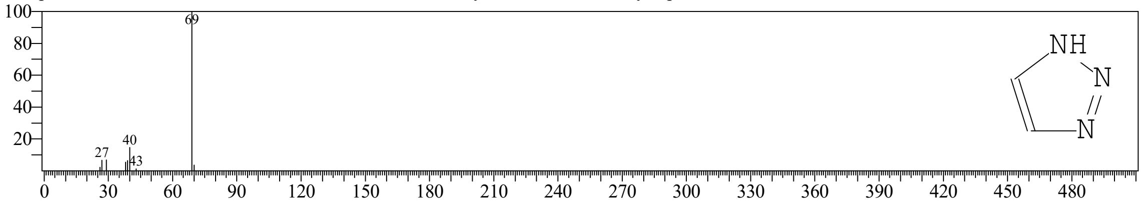

Hit#:2 Entry:981 Library:NIST17-1.lib
SI:96 Formula:CF4 CAS:75-73-0 MolWeight:88 RetIndex:0
CompName:Tetrafluoromethane $$ Carbon tetrafluoride $$ Methane, tetrafluoro- $$ Arcton 0 $$ Carbon fluoride (CF4) $$ Freon 14 $$ FC 14 $$ Perfluoro

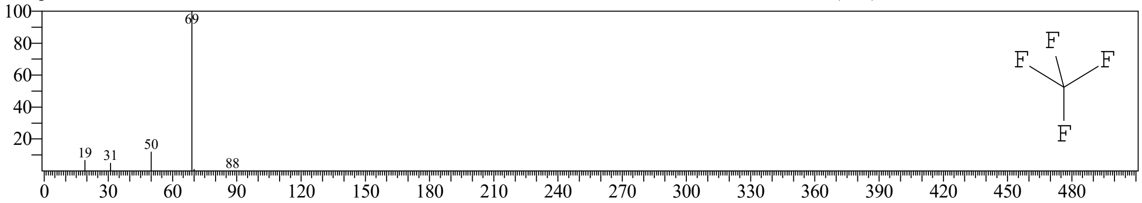

Hit#:3 Entry:10642 Library:NIST17-1.lib
SI:96 Formula:CF5P CAS:1112-04-5 MolWeight:138 RetIndex:0
CompName:Trifluoromethyldifluorophosphine $$ Trifluoromethylphosphonous difluoride # $$

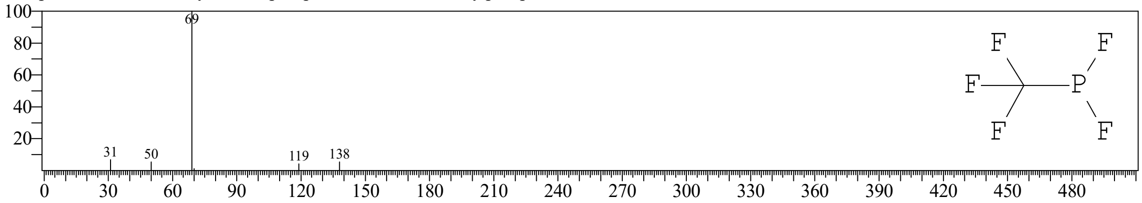

Hit#:4 Entry:10455 Library:NIST17-1.lib
SI:95 Formula:CClF4N CAS:13880-72-3 MolWeight:137 RetIndex:0
CompName:N-Chloro-N-fluoro-trifluoromethylamine $$ N-Chloro-N-tetrafluoromethanamine # $$

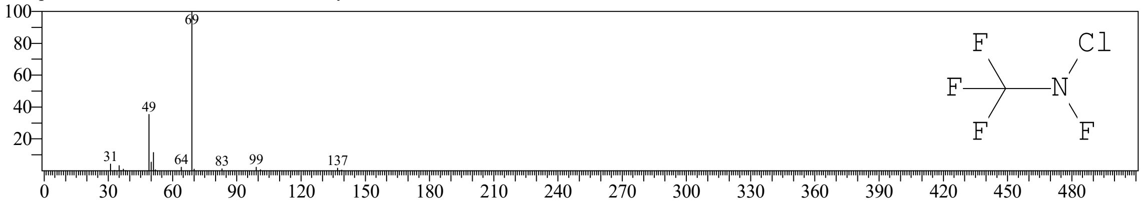

Hit#:5 Entry:7138 Library:NIST17-1.lib
SI:94 Formula:C5H5NOS CAS:60034-28-8 MolWeight:127 RetIndex:0
CompName:Crotonyl isothiocyanate $$ (2E)-2-Butenoyl isothiocyanate # $$

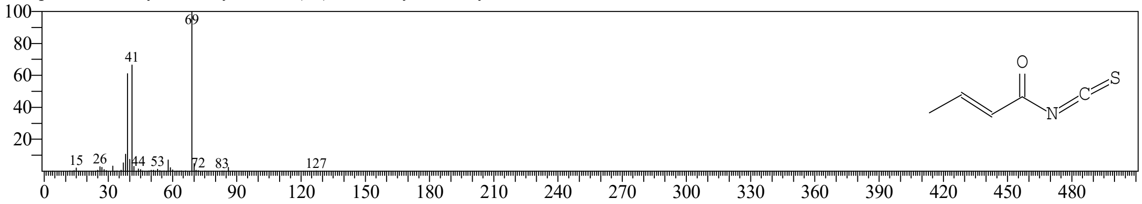

Qualitative Analysis Report

<< Target >>
Line#:53 R.Time:29.337(Scan#:6082) Retention Index:1390 MassPeaks:278
RawMode:Averaged 29.333-29.342(6081-6083) BasePeak:91.10(2506)
BG Mode:Calc. from Peak Group 1 - Event 1 Scan

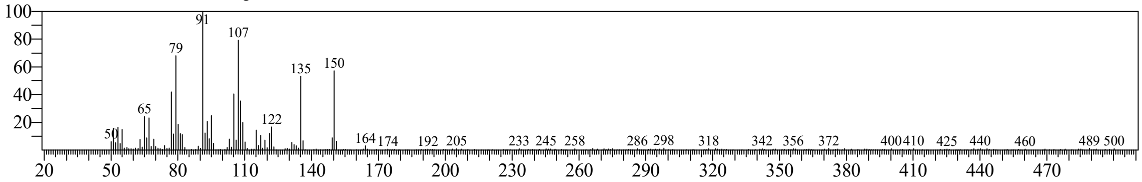

Hit#:1 Entry:31751 Library:NIST17-1.lib
SI:93 Formula:C9H8N2O2 CAS:16844-42-1 MolWeight:176 RetIndex:0
CompName:Sydnone, 3-(phenylmethyl)- $$ Sydnone, 3-benzyl- $$ Benzyl sydnone $$ 3-Benzylsydnone $$ 3-Benzyl-1,2,3-oxadiazol-3-ium-5-olate # $$

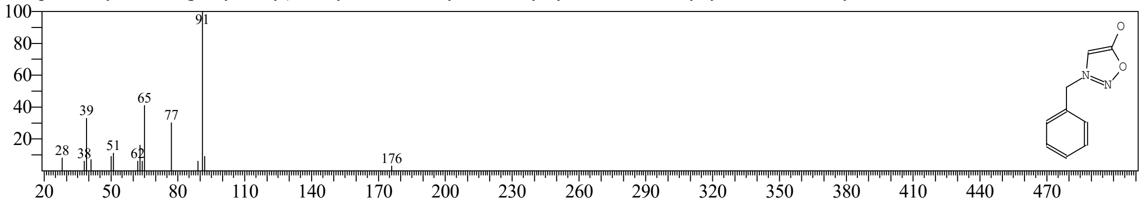

Hit#:2 Entry:33118 Library:NIST17-1.lib
SI:89 Formula:C10H10O3 CAS:6362-58-9 MolWeight:178 RetIndex:1395
CompName:Benzenepropanoic acid, .alpha.-oxo-, methyl ester $$ Pyruvic acid, phenyl-, methyl ester $$ Methyl 2-oxo-3-phenylpropionate $$ Methyl 2-oxo-

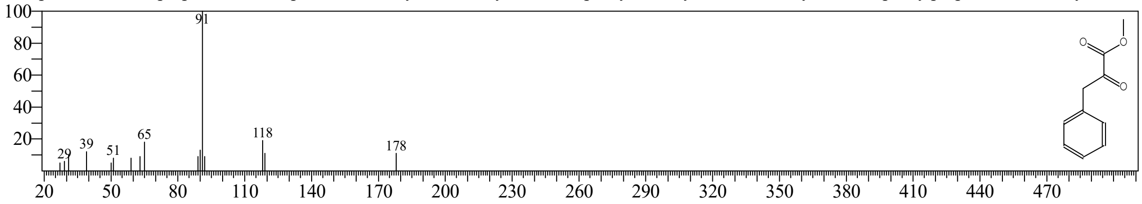

Hit#:3 Entry:231901 Library:NIST17-1.lib
SI:89 Formula:C13H14Fe2O4S2 CAS:12215-56-4 MolWeight:410 RetIndex:0
CompName:Iron, [(2,3,5,6-.eta.)-bicyclo[2.2.1]hepta-2,5-diene]tetracarbonylbis[.mu.-(methanethiolato)]di-, (Fe-Fe)

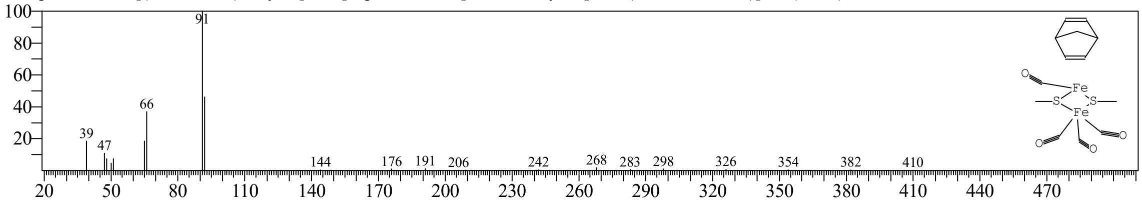

Hit#:4 Entry:5589 Library:NIST17-1.lib
SI:88 Formula:C9H12 CAS:0-00-0 MolWeight:120 RetIndex:0
CompName:Cyclonona-1,2,6-triene $$ 1,2,6-Cyclononatriene # $$

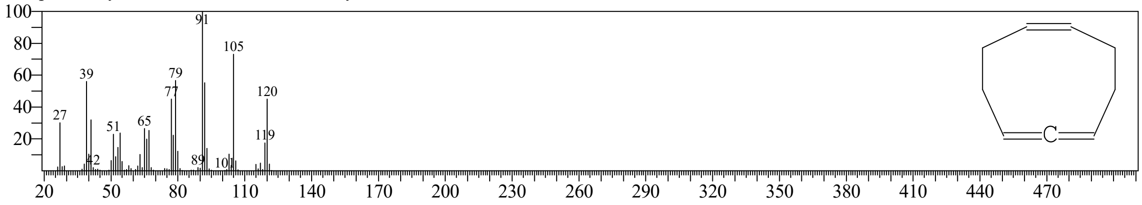

Hit#:5 Entry:168210 Library:NIST17-1.lib
SI:87 Formula:C13H7N7O4 CAS:1394125-25-7 MolWeight:325 RetIndex:0
CompName:12-Benzyl-4,9,15-trioxa-3,5,8,10,12,14,16-heptaazatetracyclo[11.3.0.0(2,6).0(7,11)]hexadeca-1(16),2,5,7,10,13-hexaen-3-one

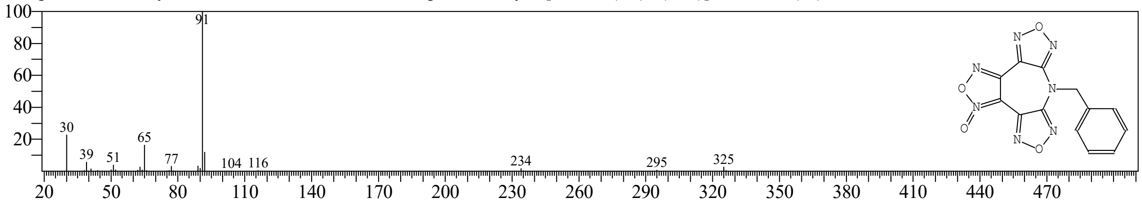

Qualitative Analysis Report

<< Target >>
Line#:54 R.Time:29.596(Scan#:6144) Retention Index:1396 MassPeaks:273
RawMode:Averaged 29.592-29.600(6143-6145) BasePeak:178.15(9227)
BG Mode:Calc. from Peak Group 1 - Event 1 Scan

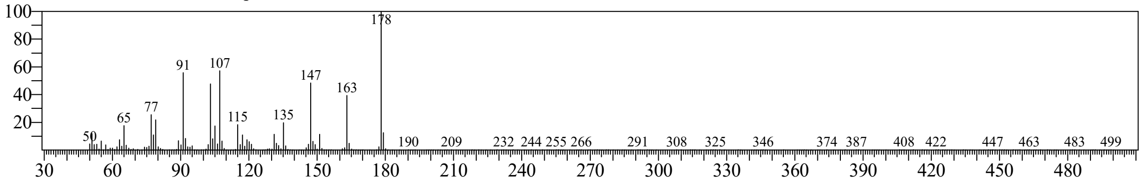

Hit#:1 Entry:532 Library:FFNSC 3.lib
SI:95 Formula:C11 H14 O2 CAS:93-15-2 MolWeight:178 RetIndex:1403
CompName:Eugenol <methyl->

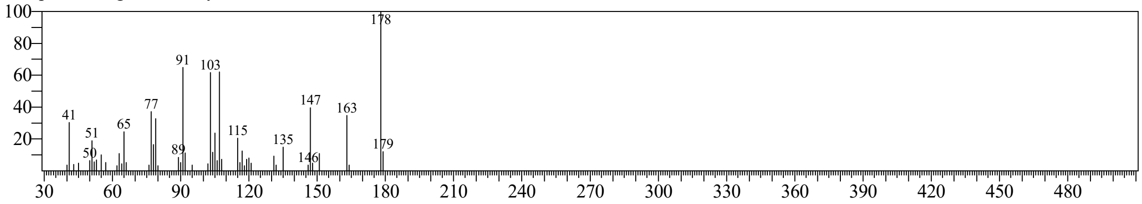

Hit#:2 Entry:32822 Library:NIST17-1.lib
SI:94 Formula:C7H6N4O2 CAS:14209-07-5 MolWeight:178 RetIndex:0
CompName:1H-Benzotriazole, 1-methyl-7-nitro- $$ 1-Methyl-7-nitro-1H-1,2,3-benzotriazole # $$

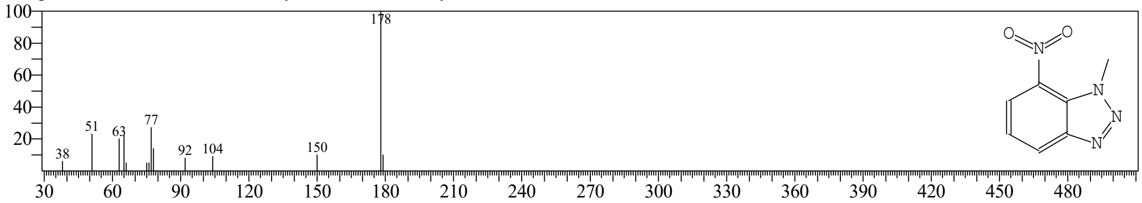

Hit#:3 Entry:32825 Library:NIST17-1.lib
SI:87 Formula:C7H6N4O2 CAS:25877-35-4 MolWeight:178 RetIndex:0
CompName:1H-Benzotriazole, 1-methyl-6-nitro- $$ 1-Methyl-6-nitro-1H-1,2,3-benzotriazole # $$

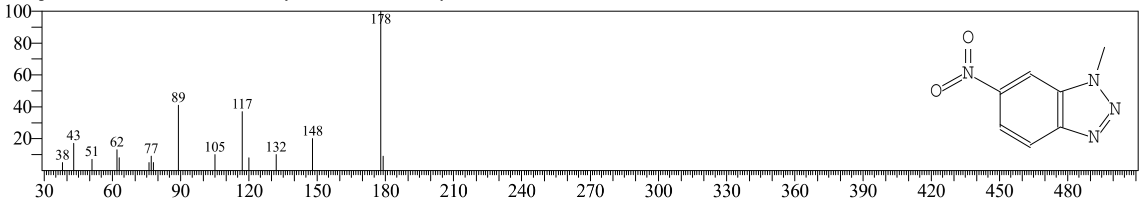

Hit#:4 Entry:32821 Library:NIST17-1.lib
SI:86 Formula:C7H6N4O2 CAS:25877-34-3 MolWeight:178 RetIndex:0
CompName:1H-Benzotriazole, 1-methyl-5-nitro- $$ 1-Methyl-5-nitro-1H-1,2,3-benzotriazole # $$

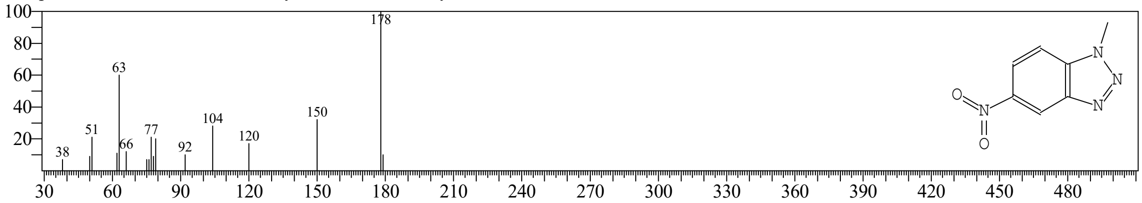

Hit#:5 Entry:32824 Library:NIST17-1.lib
SI:86 Formula:C7H6N4O2 CAS:27799-86-6 MolWeight:178 RetIndex:0
CompName:1H-Benzotriazole, 1-methyl-4-nitro- $$ 1-Methyl-4-nitro-1H-1,2,3-benzotriazole # $$

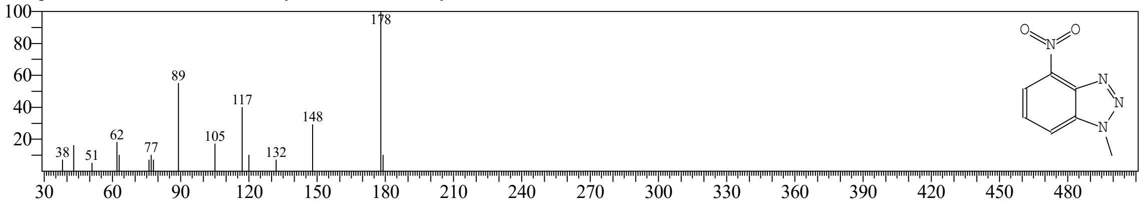

Qualitative Analysis Report

<< Target >>
Line#:55 R.Time:30.575(Scan#:6379) Retention Index:1419 MassPeaks:276
RawMode:Averaged 30.571-30.579(6378-6380) BasePeak:91.10(7966)
BG Mode:Calc. from Peak Group 1 - Event 1 Scan

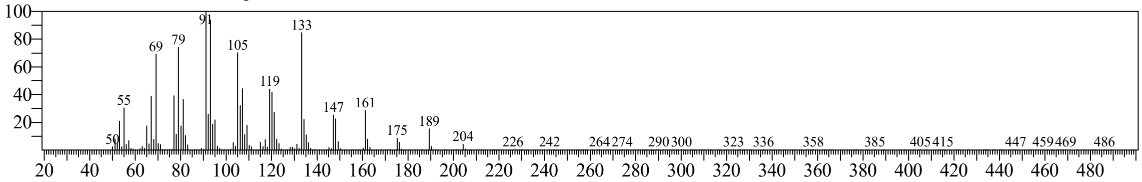

Hit#:1 Entry:1206 Library:FFNSC 3.lib
SI:97 Formula:C15 H24 CAS:13877-93-5 MolWeight:204 RetIndex:1424
CompName:Caryophyllene <(E)->

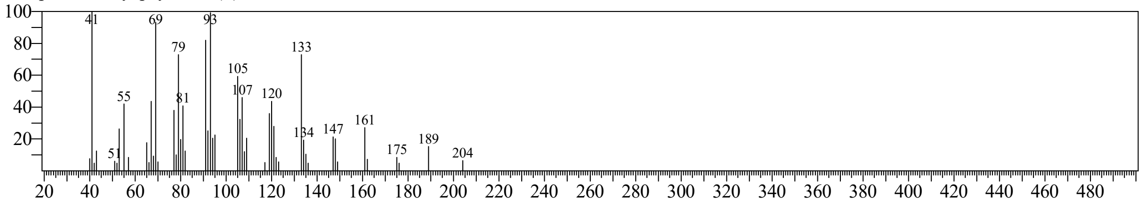

Hit#:2 Entry:2172 Library:FFNSC 3.lib
SI:94 Formula:C15 H24 CAS:118-65-0 MolWeight:204 RetIndex:1413
CompName:Caryophyllene <(Z)->

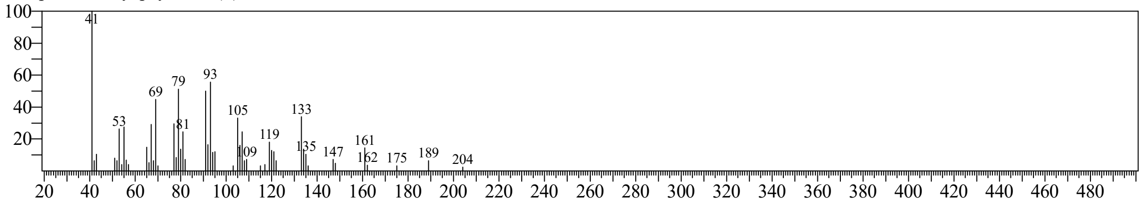

Hit#:3 Entry:52646 Library:NIST17-1.lib
SI:90 Formula:C13H20N2 CAS:0-00-0 MolWeight:204 RetIndex:0
CompName:1,4-Methanocycloocta[d]pyridazine, 1,4,4a,5,6,9,10,10a-octahydro-11,11-dimethyl-, (1.alpha.,4.alpha.,4a.alpha.,10a.alpha.)-

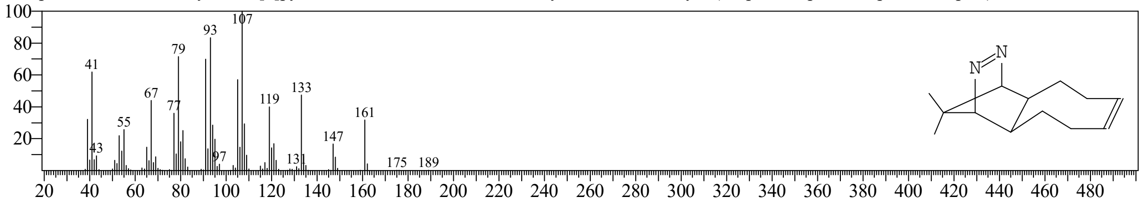

Hit#:4 Entry:231901 Library:NIST17-1.lib
SI:90 Formula:C13H14Fe2O4S2 CAS:12215-56-4 MolWeight:410 RetIndex:0
CompName:Iron, [(2,3,5,6-.eta.)-bicyclo[2.2.1]hepta-2,5-diene]tetracarbonylbis[.mu.-(methanethiolato)]di-, (Fe-Fe)

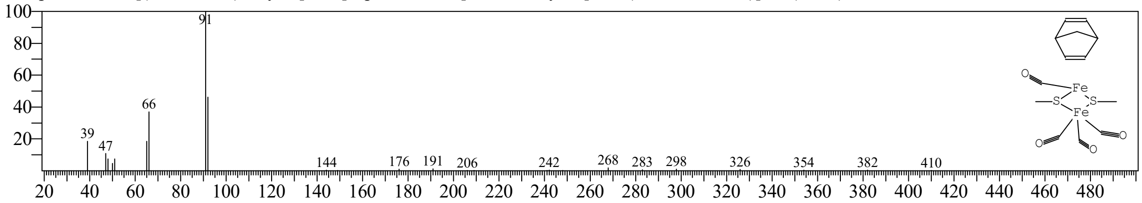

Hit#:5 Entry:31751 Library:NIST17-1.lib
SI:90 Formula:C9H8N2O2 CAS:16844-42-1 MolWeight:176 RetIndex:0
CompName:Sydnone, 3-(phenylmethyl)- $$ Sydnone, 3-benzyl- $$ Benzyl sydnone $$ 3-Benzylsydnone $$ 3-Benzyl-1,2,3-oxadiazol-3-ium-5-olate # $$

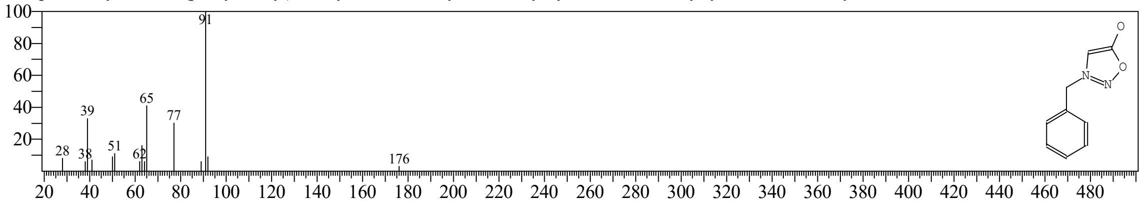

Qualitative Analysis Report

<< Target >>
Line#:56 R.Time:32.071(Scan#:6738) Retention Index:1455 MassPeaks:248
RawMode:Averaged 32.067-32.075(6737-6739) BasePeak:93.10(3278)
BG Mode:Calc. from Peak Group 1 - Event 1 Scan

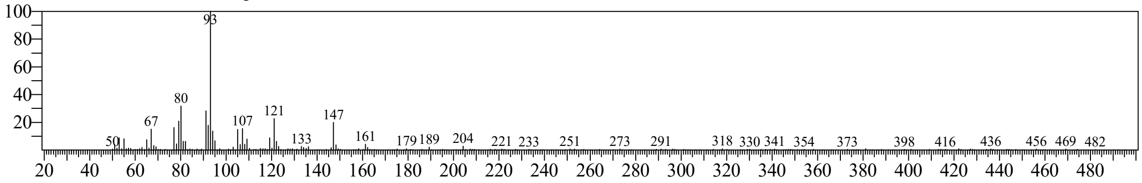

Hit#:1 Entry:1092 Library:FFNSC 3.lib
SI:97 Formula:C15 H24 CAS:6753-98-6 MolWeight:204 RetIndex:1454
CompName:Humulene <alpha->

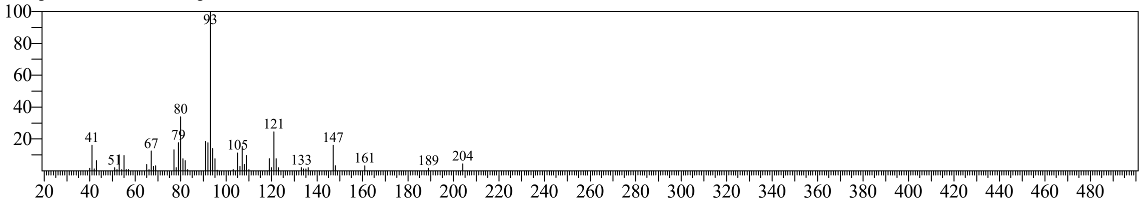

Hit#:2 Entry:24214 Library:NIST17-1.lib
SI:90 Formula:C10H16N2 CAS:87143-58-6 MolWeight:164 RetIndex:0
CompName:3,5-Methanocyclopentapyrazole, 3,3a,4,5,6,6a-hexahydro-3a,4,4-trimethyl-

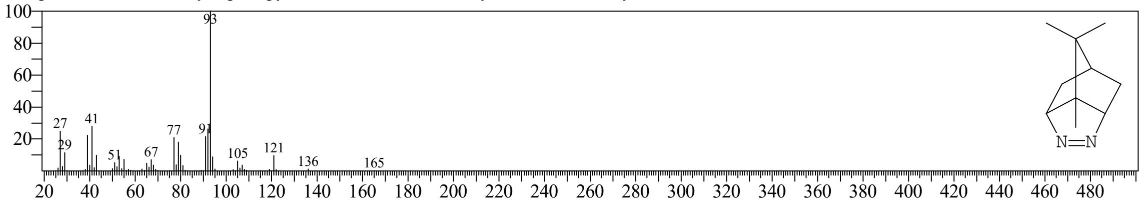

Hit#:3 Entry:2213 Library:FFNSC 3.lib
SI:87 Formula:C15 H24 CAS:135267-19-5 MolWeight:204 RetIndex:1457
CompName:Prenyl limonene <trans->

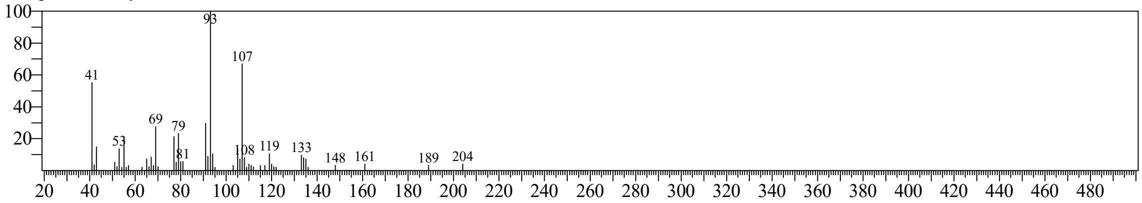

Hit#:4 Entry:58027 Library:NIST17-1.lib
SI:87 Formula:C13H22O2 CAS:0-00-0 MolWeight:210 RetIndex:1451
CompName:(E)-3,7-Dimethyl-2,6-octadien-1-yl propionate $$ Geranyl propionate $$

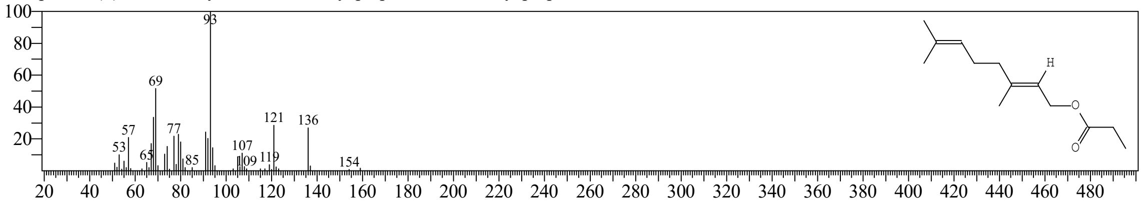

Hit#:5 Entry:2218 Library:FFNSC 3.lib
SI:86 Formula:C15 H24 CAS:135267-20-8 MolWeight:204 RetIndex:1448
CompName:Prenyl limonene < cis->

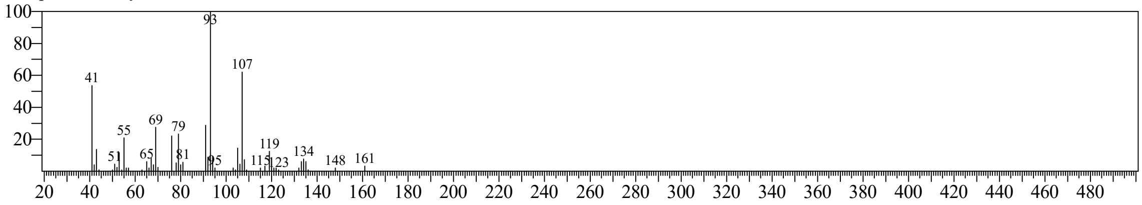

Qualitative Analysis Report

<< Target >>
Line#:57 R.Time:37.158(Scan#:7959) Retention Index:1580 MassPeaks:295
RawMode:Averaged 37.154-37.163(7958-7960) BasePeak:91.10(931)
BG Mode:Calc. from Peak Group 1 - Event 1 Scan

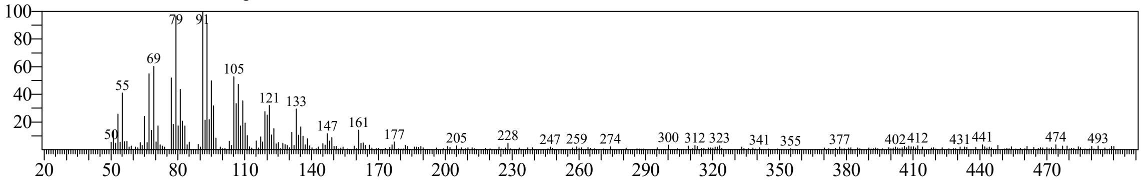

Hit#:1 Entry:145 Library:FFNSC 3.lib
SI:94 Formula:C15 H24 O CAS:1139-30-6 MolWeight:220 RetIndex:1587
CompName:Caryophyllene oxide

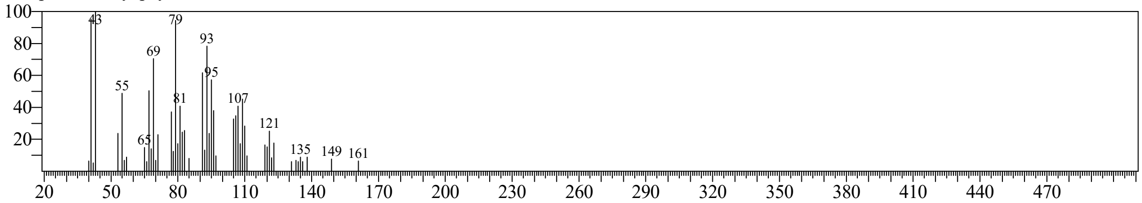

Hit#:2 Entry:31751 Library:NIST17-1.lib
SI:92 Formula:C9H8N2O2 CAS:16844-42-1 MolWeight:176 RetIndex:0
CompName:Sydnone, 3-(phenylmethyl)- $$ Sydnone, 3-benzyl- $$ Benzyl sydnone $$ 3-Benzylsydnone $$ 3-Benzyl-1,2,3-oxadiazol-3-ium-5-olate # $$

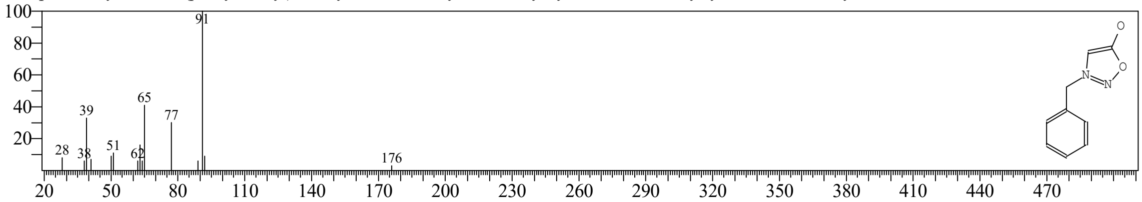

Hit#:3 Entry:903 Library:FFNSC 3.lib
SI:89 Formula:C15 H24 O CAS:72203-24-8 MolWeight:220 RetIndex:1576
CompName:Spathulenol

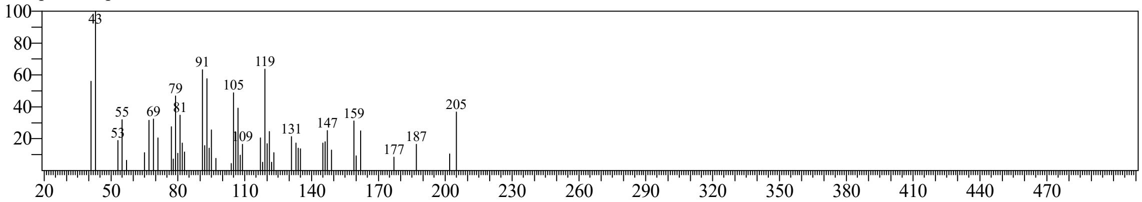

Hit#:4 Entry:231901 Library:NIST17-1.lib
SI:88 Formula:C13H14Fe2O4S2 CAS:12215-56-4 MolWeight:410 RetIndex:0
CompName:Iron, [(2,3,5,6-.eta.)-bicyclo[2.2.1]hepta-2,5-diene]tetracarbonylbis[.mu.-(methanethiolato)]di-, (Fe-Fe)

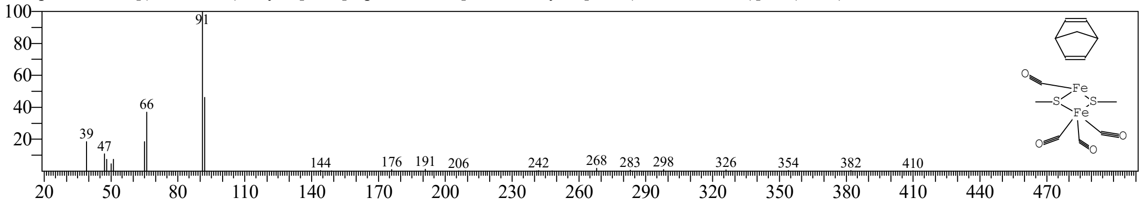

Hit#:5 Entry:2869 Library:FFNSC 3.lib
SI:88 Formula:C15 H26 O CAS:39703-23-6 MolWeight:222 RetIndex:1572
CompName:Longipinanol

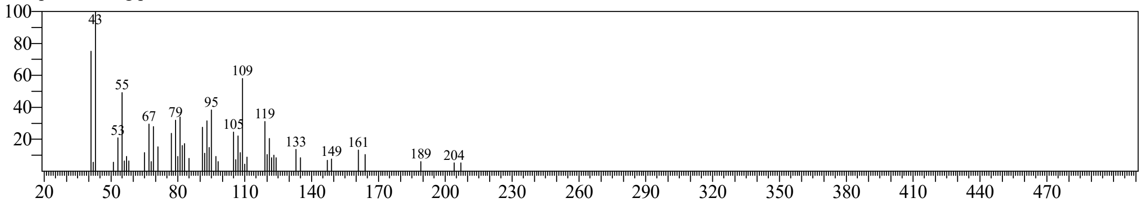

Supplement: Supplementary file 1 [file molecules-31-03346-s001.zip › Supplentary Figure S3_TIC_S rosmarinus.pdf]
